# Association of the Gut Microbiota With Cognitive Function in Midlife

**DOI:** 10.1001/jamanetworkopen.2021.43941

**Published:** 2022-02-08

**Authors:** Katie Meyer, Anju Lulla, Kunal Debroy, James M. Shikany, Kristine Yaffe, Osorio Meirelles, Lenore J. Launer

**Affiliations:** 1 Nutrition Research Institute, University of North Carolina at Chapel Hill, Kannapolis; 2 Department of Nutrition, University of North Carolina at Chapel Hill, Chapel Hill; 3 Intramural Research Program, National Institute on Aging, Bethesda, Maryland; 4 School of Medicine, Division of Preventive Medicine, University of Alabama at Birmingham; 5 Departments of Psychiatry, Neurology and Epidemiology, University of California, San Francisco

## Abstract

**Importance:**

Animal experiments and small clinical studies support a role for the gut microbiota in cognitive functioning. Few studies have investigated gut microbiota and cognition in large community samples.

**Objective:**

To examine associations of gut microbial composition with measures of cognition in an established population-based study of middle-aged adults.

**Design, Setting, and Participants:**

This cross-sectional study analyzed data from the prospective Coronary Artery Risk Development in Young Adults (CARDIA) cohort in 4 US metropolitan centers between 2015 and 2016. Data were analyzed in 2019 and 2020.

**Exposures:**

Stool DNA were sequenced, and the following gut microbial measures were gathered: (1) β-diversity (between-person) derived with multivariate principal coordinates analysis; (2) α-diversity (within-person), defined as richness (genera count) and the Shannon index (integrative measure of genera richness and evenness); and (3) taxonomy (107 genera, after filtering).

**Main Outcomes and Measures:**

Cognitive status was assessed using 6 clinic-administered cognitive tests: Montreal Cognitive Assessment (MoCA), Digit Symbol Substitution Test (DSST), Rey-Auditory Verbal Learning Test (RAVLT), Stroop, category fluency, and letter fluency. A global score measure derived using principal components analysis was also assessed; the first principal component explained 56% of variability.

**Results:**

Microbiome data were available on 597 CARDIA participants; mean (SD) age was 55.2 (3.5) years, 268 participants (44.7%) were men, and 270 (45.2%) were Black. In multivariable-adjusted principal coordinates analysis, permutational multivariate analysis of variance tests for β-diversity were statistically significant for all cognition measures (principal component analysis, *P* = .001; MoCA, *P* = .001; DSST, *P* = .001; RAVLT, *P* = .001; Stroop, *P* = .007; category fluency, *P* = .001) with the exception of letter fluency (*P* = .07). After adjusting for sociodemographic variables (age, race, sex, education), health behaviors (physical activity, diet, smoking, medication use), and clinical covariates (body mass index, diabetes, hypertension), *Barnesiella* was positively associated with the first principal component (β, 0.16; 95% CI, 0.08-0.24), DSST (β, 1.18; 95% CI, 0.35-2.00), and category fluency (β, 0.59; 95% CI, 0.31-0.87); *Lachnospiraceae FCS020 group* was positively associated with DSST (β, 2.67; 95% CI, 1.10-4.23), and *Sutterella* was negatively associated with MoCA (β, −0.27; 95% CI, −0.44 to −0.11).

**Conclusions and Relevance:**

In this cross-sectional study, microbial community composition, based on β-diversity, was associated with all cognitive measures in multivariable-adjusted analysis. These data contribute to a growing body of literature suggesting that the gut microbiota may be associated with cognitive aging, but must be replicated in larger samples and further researched to identify relevant pathways.

## Key Points

**Question:**

Is the gut microbiota associated with cognitive function?

**Findings:**

In this cross-sectional study, β-diversity, a measure of gut microbial community composition, was statistically significantly associated with all measures of cognitive function. Several specific genera were also significantly associated with 1 or more measures of cognitive function after adjustment for multiple comparisons.

**Meaning:**

These results are consistent with an association between the gut microbiota and cognitive function, and support further mechanistic and population work to elucidate the potential for gut microbiota targets for prevention or treatment of cognitive decline.

## Teaser

This cross-sectional study of US adults examines associations of gut microbial measures with the results of several cognition tests.

## Introduction

Communication pathways between gut bacteria and neurologic function (referred to as the “gut-brain axis”) have emerged as a novel area of research into potential mechanisms regulating brain health^1,2^ through immunologic, metabolic, and endocrine pathways.^3,4^ Several studies have shown associations between gut microbial measures and neurological outcomes, including cognitive function and dementia. Mechanisms have not been fully established, but there is growing support for a role in microbiota-generated short-chain fatty acids.^5,6^ In animal experiments, germ-free or antibiotic-treated rodents, which have reduced microbial diversity, have been shown to display cognitive deficits, such as reduced memory, impaired working memory, and changes in brain-derived neurotrophic factor in the hippocampus.^1,2,7,8^ Small-scale human studies have shown associations between microbial features and cognition, or found significant improvements when comparing controls with persons who have been treated with probiotics to increase commensal microbiota.^1^ However, few community-based studies have been conducted with large and diverse populations.

In this study we examine cross-sectional associations of gut microbial diversity and taxonomic composition with cognitive status among middle-aged adults recruited from 4 US centers in the Coronary Artery Risk Development in Young Adults (CARDIA) study. We hypothesized that: (1) gut microbial diversity is positively associated with global and domain-specific cognitive status, and (2) higher cognitive status is associated with specific taxonomic groups that are involved in short-chain fatty acid production. Given our current lack of understanding of the potential role for the microbiota in cognitive functioning, we have applied multiple-comparisons adjustment on the full set of tested genera, rather than a subset of genera related to short-chain fatty acid production.

## Methods

### Study Sample

CARDIA is a population-based, well-characterized, and sociodemographically diverse study of community-dwelling Black and White adults living in 4 metropolitan areas: Birmingham, Alabama; Chicago, Illinois; Minneapolis, Minnesota; and Oakland, California.^9^ At CARDIA’s Year 30 follow-up examination in 2015-16 (age range, 48-60 years; 3358 participants [70.9%] from the surviving cohort), all participants were offered a battery of cognitive assessments as part of an ancillary study, with 3124 (93.0%) completing at least 1 assessment. In addition, 615 Year 30 participants (18.3%) were recruited into a microbiome substudy, details of which have been previously reported.^10^ Analyses for the present study were conducted in 2019 and 2020. All CARDIA field centers received institutional review board approvals (University of Minnesota, University of Alabama at Birmingham, Northwestern University, and Kaiser Permanente Northern California Division of Research) and participants provided written informed consent to all study components. This study followed the Strengthening the Reporting of Observational Studies in Epidemiology (STROBE) reporting guideline.

### Microbiome Data

Standard protocols were followed for collection and processing of stool samples^11,12^ as previously described.^10^ Participants in the microbiome study completed the stool collection in their home. Participants shipped their samples to the Nutrition Research Institute at the University of North Carolina, Chapel Hill, where samples were stored at −80 °C until processing. Samples were collected in tubes containing a stabilizing solution (Thermo Fisher Scientific) and shipped overnight in insulated containers with gel ice packs. DNA was extracted from 0.2 g of stool using a DNA isolation kit (Qiagen). The V3 and V4 hypervariable regions were amplified and sequenced using a sequencer platform (2 × 300 bp) (Illumina), yielding a median (IQR) 165 536 reads (144 058-209 556 reads). Forward sequences were processed (for quality trimming, denoising, and chimera removal) through the divisive amplicon denoising algorithm DADA2 package in R (R Project for Statistical Computing)^13^ for a postprocessing median (IQR) of 115 869 reads (98 550-139 706). The DADA2-formatted Silva database was used to assign taxonomy.^14^

### Cognitive Assessments

Cognitive function was introduced in CARDIA at the Year 25 (2010-2011) examination. At the Year 30 follow-up, participants were administered 6 cognitive tests: the Digit Symbol Substitution Test (DSST), Rey-Auditory Verbal Learning Test (RAVLT); the timed Stroop test, letter fluency, and category fluency; and the Montreal Cognitive Assessment (MoCA) (scales provided in eTable 2 in the Supplement). Higher scores reflect better performance on all assessments except Stroop, for which higher scores reflect poorer performance.

### Covariates and Confounders

We included in our analyses covariates that may confound associations between cognitive test scores and microbiome characteristics. Standard questionnaires were used to obtain demographic and health behavior data at the CARDIA field centers during core examinations. CARDIA participants reported their sex and race at baseline, and age and educational attainment at each examination. Black and White race were self-reported by participants at the baseline examination—we adjusted for race in the analysis to reflect differences in both microbiome and cognition measures by race but did not hypothesize differences in associations. Educational attainment was calculated as the maximum (in years) reported by participants over their 30-year examination history. At each examination, participants reported their use of tobacco products and completed an interviewer-administered CARDIA Physical Activity Questionnaire, from which a total activity score was calculated.^15^ A brief diet assessment was completed by microbiome study participants, from which we derived a summary measure of diet quality as previously reported in CARDIA^16^ with higher scores indicating higher diet quality. Participants reported their use of medications, including for hypertension, elevated lipids, and diabetes. A medication use score was created as the total number of medications reported by participants.

Standardized protocols were used by trained staff for all clinical measures. Height and weight were measured to the nearest 0.5 cm and 0.2 kg, respectively, for body mass index (BMI; calculated as weight in kilograms divided by height in meters squared). Resting systolic and diastolic blood pressure (BP) measures were taken in the seated position. BP values were calculated as the mean of the second and third of 3 measurements taken with oscillometer calibrated to a random-zero sphygmomanometer. Hypertension was defined, based on the clinical standard at the time of the Year 30 examination, as current use of antihypertensive medication, a systolic BP 140 mm Hg or higher, or a diastolic BP 90 mm Hg or higher. Diabetes was defined as having fasting glucose 126 mg/dL (to convert to millimoles per liter, multiply by 0.0555) or greater, 2-hour oral glucose tolerance test 200 mg/dL or greater, hemoglobin A_1c_ 6.5% (to convert to proportion of total hemoglobin, multiply by 0.01) or greater, or the use of hypoglycemic medications.

### Analytical Sample

Of 615 participants who enrolled in the microbiome study, 607 had viable stool DNA for sequencing. Ten participants were missing data on all cognitive tests, yielding an analytic sample of 597. Participants with missing data on specific cognitive tests were excluded for analysis of those respective tests (596 participants for MoCA, 591 for letter fluency, and 589 for Stroop). Participants with additional missing data were excluded from regression models on a covariate basis (3 for physical activity, 7 smoking, 46 diet, and 4 diabetes), yielding sample sizes ranging from 537 to 597 depending on the level of covariate adjustment.

### Statistical Analysis

Statistical analysis was conducted on 3 standard microbial measures: within-person α-diversity, between-person β-diversity, and individual taxa. Primary analysis was at the genus level, the most refined taxonomy from these data. The R package vegan was used to generate measures of microbial diversity.^17^ Measures of α-diversity were derived from raw counts and included richness (the rarified number of genera) and Shannon Diversity Index, a function of both richness and evenness.^18^ Richness was calculated as the number of distinct genera per participant, with total per-participant abundance rarified through random sampling to the minimum count across all participants. We used multivariate principal coordinates analysis (PCoA) to assess β-diversity in microbial community composition.^19^

Prior to β-diversity analysis, raw genera counts were log-transformed as previously described.^20^ PCoA was used to generate orthogonal measures of microbial composition based on a distance matrix of microbial abundance (Bray-Curtis). For regression analysis of distinct taxa, we removed genera with a count of zero in at least 25% of participants to limit the influence of rare taxa and the possibility of spurious findings.^10^ After this filtering, 107 of 375 originally assigned genera (28.5%) remained for analysis.

We generated a summary cognitive measure based on a principal component analysis (PCA) of the 6 assessments. Each assessment was standardized as normal-inverse scores (mean [SD], 0 [1]). To limit exclusions from the PCA because of missing data, we ran PCA on the sample with complete cognitive assessment data (583 participants), set missing cognitive assessment data to zero, and applied factor loadings to nonmissing normal-inverse scores in the remaining sample. Statistical modeling focused on the first factor from PCA (first principal component [PC]), which explained 56% of variance and had loadings of 0.44 for MoCA, 0.43 for RAVLT, 0.40 for DSST, 0.40 for letter fluency, 0.39 for category fluency, and −0.38 for Stroop.

Associations between α-diversity and cognition measures were tested with multivariable-adjusted regression. Differences in β-diversity were tested with permutational multivariate analysis of variance (PERMANOVA). We conducted separate multivariable-adjusted linear regression models for each genus, adjusting for multiple comparisons using the Benjamini-Hochberg method for false discovery rate (FDR).^21^ We considered an FDR-adjusted *P* < .20 in 2-sided tests as significant.

Multivariable-adjusted analysis controlled for covariates that may have confounded associations between microbial and cognitive measures, including variables that have been associated with cognition in CARDIA or other studies. We note that some covariates may reflect mediators in associations between the microbiome and cognition, such as BMI and diabetes. Covariates included in sequential modeling were sequencing batch, age, sex, race, field center, education, physical activity, current smoking, diet quality, number of medications, BMI, hypertension, and diabetes. Statistical interaction by race or sex was tested by including crossproduct term in regression models.

## Results

Participant characteristics are shown in the Table. Participants were aged between 48 and 60 years (mean (SD) age, 55.2 [3.5] years), 267 (44.7%) were men, 270 (45.2%) were Black, and 268 (44.8%) were White. Compared with those not included in the microbiome study, participants in the microbiome study had lower mean BMI, were less likely to have prevalent hypertension, and performed slightly better on cognitive tests (eTable 1 in the Supplement). Distributions of cognitive function measures are shown in eTable 2 in the Supplement. Correlations among cognitive assessments ranged from −0.36 between category fluency and Stroop, to 0.64 between MoCA and RAVLT (eTable 3 in the Supplement). Descriptive statistics for genera included in the analysis are shown in eTable 4 in the Supplement.

**Table.** Participant Characteristics for Individuals Who Completed Cognitive Assessments at Year 30 (2015-2016): CARDIA Microbiome Study

Characteristic	No. (%) (N = 597)^a^
Age, mean (SD), y	55.2 (3.5)
Sex	
Men	267 (44.7)
Women	330 (55.3)
Race	
Black	270 (45.2)
White	327 (54.8)
Education, mean (SD), y	15.9 (2.6)
Field center	
Birmingham, Alabama	96 (16.1)
Chicago, Illinois	292 (48.9)
Minneapolis, Minnesota	108 (18.1)
Oakland, California	101 (16.9)
Current smoking	77 (13.1)
Physical activity, median (IQR), intensity units^b^	269 (127-504)
BMI, mean (SD)	29.4 (6.2)
Hypertension	209 (35.0)
Diabetes	91 (15.4)
Cognitive assessments, median (IQR)^c^	
MoCA	25 (22-27)
DSST, No. correctly substituted	70 (58-82)
Stroop, No. correctly assigned	20 (15-27)
RAVLT, No. of words recalled	9.4 (8-10.6)
Category fluency, No. of unique animals	20 (17-24)
Letter fluency, No. unique words	42 (34-50)

Abbreviations: BMI, body mass index (calculated as weight in kilograms divided by height in meters squared); DSST, the Digit Symbol Substitution Test; MoCA, Montreal Cognitive Assessment; RAVLT, Rey-Auditory Verbal Learning Test.

^a^ Sample size represents the number of participants for whom a microbiome sample and at least 1 cognitive assessment was present.

^b^ Physical activity intensity units were generated from data on the frequency, duration, and intensity for 13 activities.

^c^ See eTable 2 in the Supplement for scoring scales. Mean (SD) for cognitive measures were: MoCA, 24.4 (3.6); DSST, 69.2 (16.9); Stroop, 22.2 (11.5); RAVLT, 9.3 (1.9); category fluency, 20.6 (5.2); letter fluency, 42.6 (12.3).

### Overall Microbial Community Composition

Descriptive data for cognitive assessments and genera with respect to the first 2 PCoA axes are provided in eTables 5 and 6 in the Supplement, respectively. Cognitive function scores were significantly associated with β-diversity based on PCoA (PCA, *P* = .001; MoCA, *P* = .001; DSST, *P* = .001; RAVLT, *P* = .001; Stroop, *P* = .007; category fluency, *P* = .001) with the exception of letter fluency (*P* = .07) (eFigure in the Supplement). We observed a statistically significant PERMANOVA test for interaction by sex with RAVLT (*P* = .04) (eTable 8 in the Supplement), although PCoA differences were significant in both men and women (eTable 9 in the Supplement). PERMANOVA tests for interaction by race were not significant (eTables 8 and 9 in the Supplement).

### α-Diversity

In multivariable-adjusted analysis, Shannon diversity was positively associated with DSST (β, 1.27; 95% CI, 0.05-2.48), indicating a 1.27 higher DSST score (by SD-units) for an SD-unit higher Shannon diversity index score. Other findings for α-diversity and cognition were not significant (eTable 5 in the Supplement). There was no evidence for interaction by race or sex (eTables 10 and 11 in the Supplement).

### Distinct Taxonomies

Accounting for multiple comparisons, genera-specific analysis revealed several associations with cognitive assessments, but few associations remained significant (at the FDR-adjusted threshold of *P* < .20) in fully adjusted models (eTables 12-18 in the Supplement). Assessments were associated with genera at varying levels of multivariable adjustment (FDR-adjusted *P* < .20) (Figure). In these models, β-coefficients indicate the difference in the cognitive assessment measures (see eTable 2 in the Supplement for distributional data) associated with a 1-unit higher log-transformed genera abundance. In fully adjusted analysis controlling for sociodemographics, health behaviors, and clinical measures, *Barnesiella* (median [IQR] log-transform, 0.82 [0-3.02]) was positively associated with DSST (β, 1.18; 95% CI, 0.35-2.00), category fluency (β, 0.59; 95% CI, 0.31-0.87), and the first PC (β, 0.16; 95% CI, 0.08-0.24); *Lachnospiraceae FCS020 group* (median (IQR) log-transform, 1.38 [0-1.75]) was positively associated with DSST (β, 2.67; 95% CI, 1.10-4.23); *Akkermansia* (median [IQR] log-transform, 1.57 [0-3.00]) was positively associated with DSST (β, 1.28; 95% CI, 0.39-2.17), and *Sutterella* (median [IQR] log-transform, 2.80 [0-3.37]) was negatively associated with MoCA (β, −0.27; 95% CI, −0.44 to −0.11). Nine genera were associated with more than 1 cognitive test in sociodemographics-adjusted analysis (without education), including 6 from within Clostridia class (3 of which were from Lachnospirales order). We observed significant statistical tests for interaction by race or sex for several genera, but a large number of tests were conducted, even with FDR-adjustment, and there was a relatively small sample size (eTables 19-60 in the Supplement).

**Figure.**
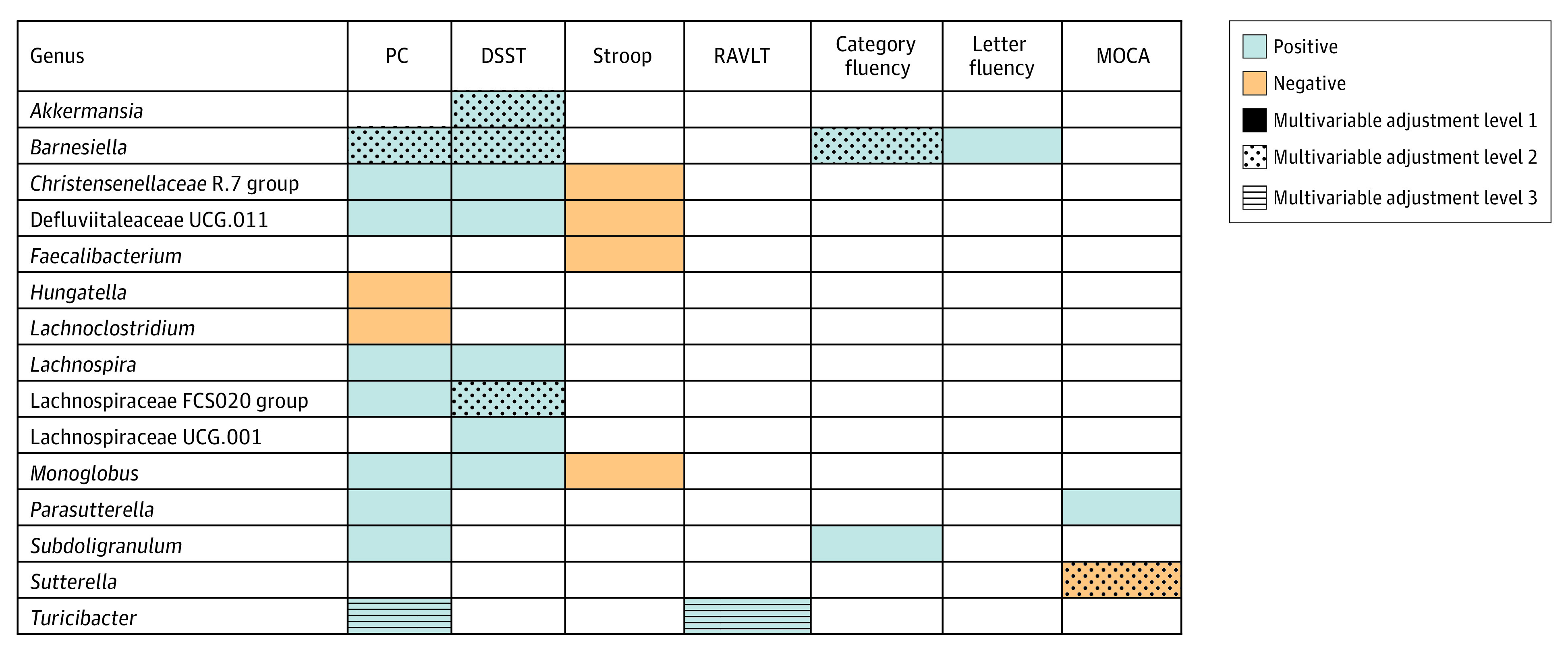
Multivariable-Adjusted Associations Between Specific Genera and Measures of Cognitive Function Genera are restricted to those associated with at least 1 cognitive measure with an FDR-adjusted *P* < .20. Colors reflect direction of β coefficients (blue indicates positive; orange, negative). Stroop is on its natural scale, with lower values reflecting higher cognitive scores. Patterning indicates the highest level of multivariable adjustment for which results were statistically significant, as follows: solid indicates results were significant after adjustment for sequencing batch, age, race, sex, study center; dotted, results remained significant after additional adjustment for education; horizontal lines, results were significant after further adjustment for physical activity, diet, smoking, medication use, body mass index, diabetes, and hypertension.

In secondary analysis of phyla (descriptive data available in eTable 61 in the Supplement), Verrucomicrobia was positively associated with DSST (β, 1.34; 95% CI, 0.43-2.26) (eTables 62-68 in the Supplement) and Proteobacteria was positively associated with category fluency (β, 1.08; 95% CI, 0.34-1.82). The ratio of Firmicutes to Bacteroidetes was not associated with cognitive measures in these data (eTable 69 in the Supplement).

## Discussion

In this cross-sectional analysis of data from a population-based cohort of middle-aged Black and White US adults, gut microbial composition was associated with domain-specific and global measures of cognition. A multivariate measure of between-person differences in microbial community structure was significantly associated with cognitive measures adjusted for sociodemographics, health behaviors, and clinical risk factors. In contrast, within-person microbial diversity was generally not associated with cognition in these data. Genera-specific associations were sensitive to covariate adjustment, and relatively few associations remained significant in fully adjusted models.

Our findings are consistent with results from animal models and small clinical studies. However, direct comparisons across human studies can be difficult given the limited sample sizes of many published reports, the use of case-control designs comparing normal controls with cases of dementia, variation in the taxonomic level of analysis, and the inconsistent adjustment for potential confounders.^22^ Findings from our genera-specific analysis are consistent with proposed pathways through production of the short-chain fatty acid butyrate, many members of which are within class Clostridia.^23^ In animal models, administration of butyrate has been shown to be protective against vascular dementia^24,25^ and cognitive impairment,^26,27^ as well as against metabolic risk factors for cognitive decline and dementia.^28^ In a study of transgenic mice, an antibiotic-induced increase in the relative abundance of genus *Lachnospiraceae*, positively associated with DSST and the first PC in our data, was accompanied by upregulation of FOXP3^+^ regulatory T cells and reduced deposition of β amyloid in brain tissue.^8^ *Akkermansia*, associated with DSST in our data, is a mucin-degrading genus that has been shown to improve gut membrane integrity,^29,30^ associate negatively with inflammation and adverse metabolic outcomes,^30,31,32,33^ and associate positively with cognitive function.^34^ Additional data from larger human studies are needed to confirm our results and test translation of findings from animal models to human samples. We identified a potentially novel positive association between *Barnesiella* and cognitive function. *Barnesiella* remained positively associated with DSST, category fluency, and the first PC in fully adjusted models. *Barnesiella* is involved in carbohydrate fermentation, competitive inhibition of pathogenic bacteria, and immunoregulation^35,36,37^; it has been shown to be enriched in arthritis-resistant mice^38^ and associated with lower levels of active colitis in IL-10^−/−^ mice.^39^

A critical challenge for observational studies of gut microbiota is the potential for confounding, particularly as we continue to learn about variables that may influence the gut microbiota.^40^ In CARDIA, we were able to account for a large set of sociodemographic, behavioral, and clinical variables that have been associated with cognitive function. Significant associations between specific genera and cognitive measures were appreciably attenuated upon adjustment for education, behavioral confounders, and clinical measures. Furthermore, the confounding structure may vary across genera; for example, controlling for education attenuated several genus-cognition associations that were robust to adjustment for other sociodemographic variables or health behaviors; however, other genus-cognition associations were more sensitive to adjustment for health behaviors, as compared with education. These results highlight the importance of adjusting for multiple factors known to be associated with the gut microbiome and the outcome.

### Strengths and Limitations

Our study had several strengths. A population-based, sociodemographically diverse cohort, CARDIA is more representative than patient samples, particularly for the study of cognition among middle-aged adults. The battery of cognitive assessments assessed multiple domain-specific and global measures of cognition. Standardized protocols were used for all data collection and quality control, including participant surveys; clinic-based assessments; and stool collection, processing, and sequencing.

This study also had several limitations. Our sample size, although larger than many human microbiome studies, is relatively small for epidemiologic analysis of multiple comparisons, particularly for assessment of potential effect measure modification by sex and race. We derived gut microbiota measures based on a single stool sample; however, studies of US populations have documented relative stability over 6 to 12 months, with between-person differences consistently and significantly exceeding within-person changes.^41,42^ The cross-sectional design prevents the assessment of temporality, and it is possible that declining health itself influences the gut microbial community. Although we had access to rich covariate data, residual confounding is possible, particularly for self-reported health behaviors that may have greater measurement error. It is unlikely that significant cognitive decline has occurred in our middle-aged sample, and results may not translate to mild cognitive impairment or later disease states. Our analysis relied on 16S rRNA sequence data, which allowed us to characterize the microbial community with respect to composition but not directly to function. Future work is needed using whole-metagenomics sequencing, which would enable direct derivation of gene families and metabolic pathways, as well as refined taxonomic assignments to the species or strain level.

## Conclusions

These data support an association between the gut microbiome and cognitive function. Multivariate analysis illustrated significant differences in microbial community composition according to cognition. Taxa-specific analysis was sensitive to covariate adjustment but revealed patterns and genera-specific associations consistent with mechanistic literature. Specifically, genera within class Clostridia and genus *Akkermansia* have been hypothesized to play a role in mechanisms involving gut membrane integrity and systemic inflammation. These results need to be replicated in larger human samples. Further investigation, validation, and replication are needed to delineate genomic and metabolic profiles of gut microbial signals using whole-metagenomics (shotgun) sequencing. Longitudinal data are required to confirm that gut microbial changes precede relevant physiologic alterations. The gut microbiota is potentially modifiable through health behaviors and targeted treatments. Increments in evidence may lead to new opportunities to reduce cognitive decline in later age through designs of interventions, identification of biomarkers of risk stratification, or modification of chronic diseases that lead to cognitive decline.

## Notes

Supplement. **eTable 1.** CARDIA Participant Characteristics for Individuals Who Completed Cognitive Assessments at Year 30 (2015-16), According to Enrollment in the Microbiome Study **eTable 2.** Descriptive Statistics for the Seven Cognitive Function Measures **eTable 3.** Correlation Coefficients for the Seven Cognitive Function Measures **eTable 4.** Descriptive Statistics for Genera **eTable 5.** Means (SDs) of Seven Cognitive Function Measures According to Quartiles of the First Two PCoA Axes (MDS1 and MDS2) **eTable 6.** Correlations Between Each of the First Two Axes of the PCoA (MDS1 and MDS2) with Genera Used in the Analysis **eTable 7.** Multivariable-adjusted Associations Between Gut Microbial Alpha and Beta Diversity and Cognition Measures in CARDIA **eTable 8.** PERMANOVA Tests (*P* Values) for Interaction Between Beta-diversity (PCoA) and Sex (Panel A) and Race (Panel B) **eTable 9.** PERMANOVA Test (*P* Values) for Sex- (Panel A & B) and Race- (Panel C & D) Stratified Results **eTable 10.** *P* Values for Interaction Between Sex (Panel A) and Race (Panel B) With Alpha Diversity Measures (Shannon and Richness) **eTable 11.** Beta-coefficients (95% CI) and *P* Values for Alpha-diversity Measures (Shannon and Richness) Within Strata of Sex (Panel A) and Race (Panel B) **eTable 12.** Multivariable-adjusted Regression Coefficients, *P* Values, Race Interaction *P* Values, False Discovery Rate (FDR)-adjusted *P* Values for Genera With Respect to DSST **eTable 13.** Multivariable-adjusted Regression Coefficients, *P* Values, Race Interaction *P* Values, False Discovery Rate (FDR)-adjusted *P* Values for Genera With Respect to Stroop **eTable 14.** Multivariable-adjusted Regression Coefficients, *P* Values, Race Interaction *P* Values, False Discovery Rate (FDR)-adjusted *P* Values for Genera With Respect to RAVLT **eTable 15.** Multivariable-adjusted Regression Coefficients, *P* Values, Race Interaction *P* Values, False Discovery Rate (FDR)-adjusted *P* Values for Genera With Respect to Category Fluency **eTable 16.** Multivariable-adjusted Regression Coefficients, *P* Values, Race Interaction *P* Values, False Discovery Rate (FDR)-adjusted *P* Values for Genera With Respect to Letter Fluency **eTable 17.** Multivariable-adjusted Regression Coefficients, *P* Values, Race Interaction *P* Values, False Discovery Rate (FDR)-adjusted *P* Values for Genera With Respect to MOCA **eTable 18.** Multivariable-adjusted Regression Coefficients, *P* Values, Race Interaction *P* Values, False Discovery Rate (FDR)-adjusted *P* Values for Genera With Respect to DSST **eTable 19.** Multivariable-adjusted Regression Coefficients, *P* Values, Race Interaction *P* Values, False Discovery Rate (FDR)-adjusted *P* Values for Race-Genera Interactions With Respect to DSST **eTable 20.** Multivariable-adjusted Regression Coefficients, *P* Values, Race Interaction *P* Values, False Discovery Rate (FDR)-adjusted *P* Values for Race-Genera Interactions With Respect to Stroop **eTable 21.** Multivariable-adjusted Regression Coefficients, *P* Values, Race Interaction *P* Values, False Discovery Rate (FDR)-adjusted *P* Values for Race-Genera Interactions With Respect to RAVLT **eTable 22.** Multivariable-adjusted Regression Coefficients, *P* Values, Race Interaction *P* Values, False Discovery Rate (FDR)-adjusted *P* Values for Race-Genera Interactions With Respect to Category Fluency **eTable 23.** Multivariable-adjusted Regression Coefficients, *P* Values, Race Interaction *P* Values, False Discovery Rate (FDR)-adjusted *P* Values for Race-Genera Interactions With Respect to Letter Fluency **eTable 24.** Multivariable-adjusted Regression Coefficients, *P* Values, Race Interaction *P* Values, False Discovery Rate (FDR)-adjusted *P* Values for Race-Genera Interactions With Respect to MOCA **eTable 25.** Multivariable-adjusted Regression Coefficients, *P* Values, Race Interaction *P* Values, False Discovery Rate (FDR)-adjusted *P* Values for Race-Genera Interactions With Respect to PC (1st) **eTable 26.** Multivariable-adjusted Regression Coefficients, *P* Values, and False Discovery Rate (FDR)-adjusted *P* Values for Genera Among Black Participants With Respect to DSST **eTable 27.** Multivariable-adjusted Regression Coefficients, *P* Values, and False Discovery Rate (FDR)-adjusted *P* Values for Genera Among Black Participants With Respect to Stroop **eTable 28.** Multivariable-adjusted Regression Coefficients, *P* Values, and False Discovery Rate (FDR)-adjusted *P* Values for Genera Among Black Participants With Respect to RAVLT **eTable 29.** Multivariable-adjusted Regression Coefficients, *P* Values, and False Discovery Rate (FDR)-adjusted *P* Values for Genera Among Black Participants With Respect To Category Fluency **eTable 30.** Multivariable-adjusted Regression Coefficients, *P* Values, and False Discovery Rate (FDR)-adjusted *P* Values for Genera Among Black Participants With Respect to Letter Fluency **eTable 31.** Multivariable-adjusted Regression Coefficients, *P* Values, and False Discovery Rate (FDR)-adjusted *P* Values for Genera Among Black Participants With Respect to MOCA **eTable 32.** Multivariable-adjusted Regression Coefficients, *P* Values, and False Discovery Rate (FDR)-adjusted *P* Values for Genera Among Black Participants With Respect to PC (1^st^) **eTable 33.** Multivariable-adjusted Regression Coefficients, *P* Values, and False Discovery Rate (FDR)-adjusted *P* Values for Genera Among White Participants With Respect to DSST **eTable 34.** Multivariable-adjusted Regression Coefficients, *P* Values, and False Discovery Rate (FDR)-adjusted *P* Values for Genera Among White Participants With Respect to Stroop **eTable 35.** Multivariable-adjusted Regression Coefficients, *P* Values, and False Discovery Rate (FDR)-adjusted *P* Values for Genera Among White Participants With Respect to RAVLT **eTable 36.** Multivariable-adjusted Regression Coefficients, *P* Values, and False Discovery Rate (FDR)-adjusted *P* Values for Genera Among White Participants With Respect To Category Fluency **eTable 37.** Multivariable-adjusted Regression Coefficients, *P* Values, and False Discovery Rate (FDR)-adjusted *P* Values for Genera Among White Participants With Respect to Letter Fluency **eTable 38.** Multivariable-adjusted Regression Coefficients, *P* Values, and False Discovery Rate (FDR)-adjusted *P* Values for Genera Among White Participants With Respect to MOCA **eTable 39.** Multivariable-adjusted Regression Coefficients, *P* Values, and False Discovery Rate (FDR)-adjusted *P* Values for Genera Among White Participants With Respect to PC (1^st^) **eTable 40.** Multivariable-adjusted Regression Coefficients, *P* Values, Sex Interaction *P* Values, False Discovery Rate (FDR)-adjusted *P* Values for Sex-Genera Interactions With Respect to DSST **eTable 41.** Multivariable-adjusted Regression Coefficients, *P* Values, Sex Interaction *P* Values, False Discovery Rate (FDR)-adjusted *P* Values for Sex-Genera Interactions With Respect to Stroop **eTable 42.** Multivariable-adjusted Regression Coefficients, *P* Values, Sex Interaction *P* Values, False Discovery Rate (FDR)-adjusted *P* Values for Sex-Genera Interactions With Respect to RAVLT **eTable 43.** Multivariable-adjusted Regression Coefficients, *P* Values, Sex Interaction *P* Values, False Discovery Rate (FDR)-adjusted *P* Values for Sex-Genera Interactions With Respect to Category Fluency **eTable 44.** Multivariable-adjusted Regression Coefficients, *P* Values, Sex Interaction *P* Values, False Discovery Rate (FDR)-adjusted *P* Values for Sex-Genera Interactions With Respect to Letter Fluency **eTable 45.** Multivariable-adjusted Regression Coefficients, *P* Values, Sex Interaction *P* Values, False Discovery Rate (FDR)-adjusted *P* Values for Sex-Genera Interactions With Respect to MOCA **eTable 46.** Multivariable-adjusted Regression Coefficients, *P* Values, Sex Interaction *P* Values, False Discovery Rate (FDR)-adjusted *P* Values for Sex-Genera Interactions With Respect to PC (1^st^) **eTable 47.** Multivariable-adjusted Regression Coefficients, *P* Values, and False Discovery Rate (FDR)-adjusted *P* Values for Genera Among Male Participants With Respect to DSST **eTable 48.** Multivariable-adjusted Regression Coefficients, *P* Values, and False Discovery Rate (FDR)-adjusted *P* Values for Genera Among Male Participants With Respect to Stroop **eTable 49.** Multivariable-adjusted Regression Coefficients, *P* Values, and False Discovery Rate (FDR)-adjusted *P* Values for Genera Among Male Participants With Respect to RAVLT **eTable 50.** Multivariable-adjusted Regression Coefficients, *P* Values, and False Discovery Rate (FDR)-adjusted *P* Values for Genera Among Male Participants With Respect to Category Fluency **eTable 51.** Multivariable-adjusted Regression Coefficients, *P* Values, and False Discovery Rate (FDR)-adjusted *P* Values for Genera Among Male Participants With Respect to Letter Fluency **eTable 52.** Multivariable-adjusted Regression Coefficients, *P* Values, and False Discovery Rate (FDR)-adjusted *P* Values for Genera Among Male Participants With Respect to MOCA **eTable 53.** Multivariable-adjusted Regression Coefficients, *P* Values, and False Discovery Rate (FDR)-adjusted *P* Values for Genera Among Male Participants With Respect to PC (1^st^) **eTable 54.** Multivariable-adjusted Regression Coefficients, *P* Values, and False Discovery Rate (FDR)-adjusted *P* Values for Genera Among Female Participants With Respect to DSST **eTable 55.** Multivariable-adjusted Regression Coefficients, *P* Values, and False Discovery Rate (FDR)-adjusted *P* Values for Genera Among Female Participants With Respect to Stroop **eTable 56.** Multivariable-adjusted Regression Coefficients, *P* Values, and False Discovery Rate (FDR)-adjusted *P* Values for Genera Among Female Participants With Respect to RAVLT **eTable 57.** Multivariable-adjusted Regression Coefficients, *P* Values, and False Discovery Rate (FDR)-adjusted *P* Values for Genera Among Female Participants With Respect to Category Fluency **eTable 58.** Multivariable-adjusted Regression Coefficients, *P* Values, and False Discovery Rate (FDR)-adjusted *P* Values for Genera Among Female Participants With Respect to Letter Fluency **eTable 59.** Multivariable-adjusted Regression Coefficients, *P* Values, and False Discovery Rate (FDR)-adjusted *P* Values for Genera Among Female Participants With Respect to MOCA **eTable 60.** Multivariable-adjusted Regression Coefficients, *P* Values, and False Discovery Rate (FDR)-adjusted *P* Values for Genera Among Female Participants With Respect to PC (1^st^) **eTable 61.** Descriptive Statistics for Phyla **eTable 62.** Multivariable-adjusted Regression Coefficients, *P* Values, and False Discovery Rate (FDR)-adjusted *P* Values for Phyla With Respect to DSST **eTable 63.** Multivariable-adjusted Regression Coefficients, *P* Values, and False Discovery Rate (FDR)-adjusted *P* Values for Phyla With Respect to Stroop **eTable 64.** Multivariable-adjusted Regression Coefficients, *P* Values, and False Discovery Rate (FDR)-adjusted *P* Values for Phyla With Respect to RAVLT **eTable 65.** Multivariable-adjusted Regression Coefficients, *P* Values, and False Discovery Rate (FDR)-adjusted *P* Values for Phyla With Respect to Category Fluency **eTable 66.** Multivariable-adjusted Regression Coefficients, *P* Values, and False Discovery Rate (FDR)-adjusted *P* Values for Phyla With Respect to Letter Fluency **eTable 67.** Multivariable-adjusted Regression Coefficients, *P* Values, and False Discovery Rate (FDR)-adjusted *P* Values for Phyla With Respect to MOCA **eTable 68.** Multivariable-adjusted Regression Coefficients, *P* Values, and False Discovery Rate (FDR)-adjusted *P* Values for Phyla With Respect to PC (1^st^) **eTable 69.** Coefficients and *P* Values for Associations Between Bacteroidota:Firmicutes Ratio With Measures of Cognitive Function **eFigure.** Beta-diversity (PCoA) Differences for Each Cognitive Function Assessment
