# Supplementary material for: Association of the Gut Microbiota With Cognitive Function in Midlife
Source: JAMA Netw Open. 2022 Feb 8;5(2):e2143941. doi: 10.1001/jamanetworkopen.2021.43941 (PMC8826173; doi:10.1001/jamanetworkopen.2021.43941)
Supplement: Supplement. — eTable 1. CARDIA Participant Characteristics for Individuals Who Completed Cognitive Assessments at Year 30 (2015-16), According to Enrollment in the Microbiome Study eTable 2. Descriptive Statistics for the Seven Cognitive Function Measures eTable 3. Correlation Coefficients for the Seven Cognitive Function Measures eTable 4. Descriptive Statistics for Genera eTable 5. Means (SDs) of Seven Cognitive Function Measures According to Quartiles of the First Two PCoA Axes (MDS1 and MDS2) eTable 6. Correlations Between Each of the First Two Axes of the PCoA (MDS1 and MDS2) with Genera Used in the Analysis eTable 7. Multivariable-adjusted Associations Between Gut Microbial Alpha and Beta Diversity and Cognition Measures in CARDIA eTable 8. PERMANOVA Tests (P Values) for Interaction Between Beta-diversity (PCoA) and Sex (Panel A) and Race (Panel B) eTable 9. PERMANOVA Test (P Values) for Sex- (Panel A & B) and Race- (Panel C & D) Stratified Results eTable 10. P Values for Interaction Between Sex (Panel A) and Race (Panel B) With Alpha Diversity Measures (Shannon and Richness) eTable 11. Beta-coefficients (95% CI) and P Values for Alpha-diversity Measures (Shannon and Richness) Within Strata of Sex (Panel A) and Race (Panel B) eTable 12. Multivariable-adjusted Regression Coefficients, P Values, Race Interaction P Values, False Discovery Rate (FDR)-adjusted P Values for Genera With Respect to DSST eTable 13. Multivariable-adjusted Regression Coefficients, P Values, Race Interaction P Values, False Discovery Rate (FDR)-adjusted P Values for Genera With Respect to Stroop eTable 14. Multivariable-adjusted Regression Coefficients, P Values, Race Interaction P Values, False Discovery Rate (FDR)-adjusted P Values for Genera With Respect to RAVLT eTable 15. Multivariable-adjusted Regression Coefficients, P Values, Race Interaction P Values, False Discovery Rate (FDR)-adjusted P Values for Genera With Respect to Category Fluency eTable 16. Multivariable-adjusted Regression Coefficien [file jamanetwopen-e2143941-s001.pdf]

Supplemental Online Content

Meyer K, Lulla A, Debroy K, et al. Association of the gut microbiota and cognitive function in midlife. *JAMA Netw Open.* 2022;5(2):e2143941. doi:10.1001/jamanetworkopen.2021.43941

eTable 1. CARDIA Participant Characteristics for Individuals Who Completed Cognitive Assessments at Year 30 (2015-16), According to Enrollment in the Microbiome Study

eTable 2. Descriptive Statistics for the Seven Cognitive Function Measures

eTable 3. Correlation Coefficients for the Seven Cognitive Function Measures

eTable 4. Descriptive Statistics for Genera

eTable 5. Means (SDs) of Seven Cognitive Function Measures According to Quartiles of the First Two PCoA Axes (MDS1 and MDS2)

eTable 6. Correlations Between Each of the First Two Axes of the PCoA (MDS1 and MDS2) With Genera Used in the Analysis

eTable 7. Multivariable-adjusted Associations Between Gut Microbial Alpha and Beta Diversity and Cognition Measures in CARDIA

eTable 8. PERMANOVA Tests (*P* Values) for Interaction Between Beta-diversity (PCoA) and Sex (Panel A) and Race (Panel B)

eTable 9. PERMANOVA test (*P* Values) for Sex- (panel A & B) and Race- (panel C & D) Stratified Results

eTable 10. *P* values for Interaction Between Sex (Panel A) and Race (Panel B) With Alpha Diversity Measures (Shannon and Richness)

eTable 11. Beta-coefficients (95% CI) and *P* Values for Alpha-diversity Measures (Shannon and Richness) Within Strata of Sex (Panel A) and Race (Panel B)

eTable 12. Multivariable-adjusted Regression Coefficients, *P* Values, Race Interaction *P* Values, False Discovery Rate (FDR)-adjusted *P* Values for Genera With Respect to DSST

© 2022 Meyer K et al. *JAMA Network Open.*

eTable 13. Multivariable-adjusted Regression Coefficients, *P* Values, Race Interaction *P* Values, False Discovery Rate (FDR)-adjusted *P* Values for Genera With Respect to Stroop

eTable 14. Multivariable-adjusted Regression Coefficients, *P* Values, Race Interaction *P* Values, False Discovery Rate (FDR)-adjusted *P* Values for Genera With Respect to RAVLT

eTable 15. Multivariable-adjusted Regression Coefficients, *P* Values, Race Interaction *P* Values, False Discovery Rate (FDR)-adjusted *P* Values for Genera With Respect to Category Fluency

eTable 16. Multivariable-adjusted Regression Coefficients, *P* Values, Race Interaction *P* Values, False Discovery Rate (FDR)-adjusted *P* Values for Genera With Respect to Letter Fluency

eTable 17. Multivariable-adjusted Regression Coefficients, *P* Values, Race Interaction *P* Values, False Discovery Rate (FDR)-adjusted *P* Values for Genera With Respect to MOCA

eTable 18. Multivariable-adjusted Regression Coefficients, *P* Values, Race Interaction *P* Values, False Discovery Rate (FDR)-adjusted *P* Values for Genera With Respect to DSST

eTable 19. Multivariable-adjusted Regression Coefficients, *P* Values, Race Interaction *P* Values, False Discovery Rate (FDR)-adjusted *P* Values for Race-Genera Interactions With Respect to DSST

eTable 20. Multivariable-adjusted Regression Coefficients, *P* Values, Race Interaction *P* Values, False Discovery Rate (FDR)-adjusted *P* Values for Race-Genera Interactions With Respect to Stroop

eTable 21. Multivariable-adjusted Regression Coefficients, *P* Values, Race Interaction *P* Values, False Discovery Rate (FDR)-adjusted *P* Values for Race-Genera Interactions With Respect to RAVLT

eTable 22. Multivariable-adjusted Regression Coefficients, *P* Values, Race Interaction *P* Values, False Discovery Rate (FDR)-adjusted *P* Values for Race-Genera Interactions With Respect to Category Fluency

eTable 23. Multivariable-adjusted Regression Coefficients, *P* Values, Race Interaction *P* Values, False Discovery Rate (FDR)-adjusted *P* Values for Race-Genera Interactions With Respect to Letter Fluency

eTable 24. Multivariable-adjusted Regression Coefficients, *P* Values, Race Interaction *P* Values, False Discovery Rate (FDR)-adjusted *P* Values for Race-Genera Interactions With Respect to MOCA

eTable 25. Multivariable-adjusted Regression Coefficients, *P* Values, Race Interaction *P* Values, False Discovery Rate (FDR)-adjusted *P* Values for Race-Genera Interactions With Respect to PC (1st)

eTable 26. Multivariable-adjusted Regression Coefficients, *P* Values, and False Discovery Rate (FDR)-adjusted *P* Values for Genera Among Black Participants With Respect to DSST

© 2022 Meyer K et al. *JAMA Network Open.*

eTable 27. Multivariable-adjusted Regression Coefficients, *P* Values, and False Discovery Rate (FDR)-adjusted *P* Values for Genera Among Black Participants With Respect to Stroop

eTable 28. Multivariable-adjusted Regression Coefficients, *P* Values, and False Discovery Rate (FDR)-adjusted *P* Values for Genera Among Black Participants With Respect to RAVLT

eTable 29. Multivariable-adjusted Regression Coefficients, *P* Values, and False Discovery Rate (FDR)-adjusted *P* Values for Genera Among Black Participants With Respect To Category Fluency

eTable 30. Multivariable-adjusted Regression Coefficients, *P* Values, and False Discovery Rate (FDR)-adjusted *P* Values for Genera Among Black Participants With Respect to Letter Fluency

eTable 31. Multivariable-adjusted Regression Coefficients, *P* Values, and False Discovery Rate (FDR)-adjusted *P* Values for Genera Among Black Participants With Respect to MOCA

eTable 32. Multivariable-adjusted Regression Coefficients, *P* Values, and False Discovery Rate (FDR)-adjusted *P* Values for Genera Among Black Participants With Respect to PC (1st)

eTable 33. Multivariable-adjusted Regression Coefficients, *P* Values, and False Discovery Rate (FDR)-adjusted *P* Values for Genera Among White Participants With Respect to DSST

eTable 34. Multivariable-adjusted Regression Coefficients, *P* Values, and False Discovery Rate (FDR)-adjusted *P* Values for Genera Among White Participants With Respect to Stroop

eTable 35. Multivariable-adjusted Regression Coefficients, *P* Values, and False Discovery Rate (FDR)-adjusted *P* Values for Genera Among White Participants With Respect to RAVLT

eTable 36. Multivariable-adjusted Regression Coefficients, *P* Values, and False Discovery Rate (FDR)-adjusted *P* Values for Genera Among White Participants With Respect To Category Fluency

eTable 37. Multivariable-adjusted Regression Coefficients, *P* Values, and False Discovery Rate (FDR)-adjusted *P* Values for Genera Among White Participants With Respect to Letter Fluency

eTable 38. Multivariable-adjusted Regression Coefficients, *P* Values, and False Discovery Rate (FDR)-adjusted *P* Values for Genera Among White Participants With Respect to MOCA

eTable 39. Multivariable-adjusted Regression Coefficients, *P* Values, and False Discovery Rate (FDR)-adjusted *P* Values for Genera Among White Participants With Respect to PC (1st)

eTable 40. Multivariable-adjusted Regression Coefficients, *P* Values, Sex Interaction *P* Values, False Discovery Rate (FDR)-adjusted *P* Values for Sex-Genera Interactions With Respect to DSST

© 2022 Meyer K et al. *JAMA Network Open.*

eTable 41. Multivariable-adjusted Regression Coefficients, *P* Values, Sex Interaction *P* Values, False Discovery Rate (FDR)-adjusted *P* Values for Sex-Genera Interactions With Respect to Stroop

eTable 42. Multivariable-adjusted Regression Coefficients, *P* Values, Sex Interaction *P* Values, False Discovery Rate (FDR)-adjusted *P* Values for Sex-Genera Interactions With Respect to RAVLT

eTable 43. Multivariable-adjusted Regression Coefficients, *P* Values, Sex Interaction *P* Values, False Discovery Rate (FDR)-adjusted *P* Values for Sex-Genera Interactions With Respect to Category Fluency

eTable 44. Multivariable-adjusted Regression Coefficients, *P* Values, Sex Interaction *P* Values, False Discovery Rate (FDR)-adjusted *P* Values for Sex-Genera Interactions With Respect to Letter Fluency

eTable 45. Multivariable-adjusted Regression Coefficients, *P* Values, Sex Interaction *P* Values, False Discovery Rate (FDR)-adjusted *P* Values for Sex-Genera Interactions With Respect to MOCA

eTable 46. Multivariable-adjusted Regression Coefficients, *P* Values, Sex Interaction *P* Values, False Discovery Rate (FDR)-adjusted *P* Values for Sex-Genera Interactions With Respect to PC (1st)

eTable 47. Multivariable-adjusted Regression Coefficients, *P* Values, and False Discovery Rate (FDR)-adjusted *P* Values for Genera Among Male Participants With Respect to DSST

eTable 48. Multivariable-adjusted Regression Coefficients, *P* Values, and False Discovery Rate (FDR)-adjusted *P* Values for Genera Among Male Participants With Respect to Stroop

eTable 49. Multivariable-adjusted Regression Coefficients, *P* Values, and False Discovery Rate (FDR)-adjusted *P* Values for Genera Among Male Participants With Respect to RAVLT

eTable 50. Multivariable-adjusted Regression Coefficients, *P* Values, and False Discovery Rate (FDR)-adjusted *P* Values for Genera Among Male Participants With Respect to Category Fluency

eTable 51. Multivariable-adjusted Regression Coefficients, *P* Values, and False Discovery Rate (FDR)-adjusted *P* Values for Genera Among Male Participants With Respect to Letter Fluency

eTable 52. Multivariable-adjusted Regression Coefficients, *P* Values, and False Discovery Rate (FDR)-adjusted *P* Values for Genera Among Male Participants With Respect to MOCA

eTable 53. Multivariable-adjusted Regression Coefficients, *P* Values, and False Discovery Rate (FDR)-adjusted *P* Values for Genera Among Male Participants With Respect to PC (1st)

eTable 54. Multivariable-adjusted Regression Coefficients, *P* Values, and False Discovery Rate (FDR)-adjusted *P* Values for Genera Among Female Participants With Respect to DSST

© 2022 Meyer K et al. *JAMA Network Open.*

eTable 55. Multivariable-adjusted Regression Coefficients, *P* Values, and False Discovery Rate (FDR)-adjusted *P* Values for Genera Among Female Participants With Respect to Stroop

eTable 56. Multivariable-adjusted Regression Coefficients, *P* Values, and False Discovery Rate (FDR)-adjusted *P* Values for Genera Among Female Participants With Respect to RAVLT

eTable 57. Multivariable-adjusted Regression Coefficients, *P* Values, and False Discovery Rate (FDR)-adjusted *P* Values for Genera Among Female Participants With Respect to Category Fluency

eTable 58. Multivariable-adjusted Regression Coefficients, *P* Values, and False Discovery Rate (FDR)-adjusted *P* Values for Genera Among Female Participants With Respect to Letter Fluency

eTable 59. Multivariable-adjusted Regression Coefficients, *P* Values, and False Discovery Rate (FDR)-adjusted *P* Values for Genera Among Female Participants With Respect to MOCA

eTable 60. Multivariable-adjusted Regression Coefficients, *P* Values, and False Discovery Rate (FDR)-adjusted *P* Values for Genera Among Female Participants With Respect to PC (1st)

eTable 61. Descriptive Statistics for Phyla

eTable 62. Multivariable-adjusted Regression Coefficients, *P* Values, and False Discovery Rate (FDR)-adjusted *P* Values for Phyla With Respect to DSST

eTable 63. Multivariable-adjusted Regression Coefficients, *P* Values, and False Discovery Rate (FDR)-adjusted *P* Values for Phyla With Respect to Stroop

eTable 64. Multivariable-adjusted Regression Coefficients, *P* Values, and False Discovery Rate (FDR)-adjusted *P* Values for Phyla With Respect to RAVLT

eTable 65. Multivariable-adjusted Regression Coefficients, *P* Values, and False Discovery Rate (FDR)-adjusted *P* Values for Phyla With Respect to Category Fluency

eTable 66. Multivariable-adjusted Regression Coefficients, *P* Values, and False Discovery Rate (FDR)-adjusted *P* Values for Phyla With Respect to Letter Fluency

eTable 67. Multivariable-adjusted Regression Coefficients, *P* Values, and False Discovery Rate (FDR)-adjusted *P* Values for Phyla With Respect to MOCA

eTable 68. Multivariable-adjusted Regression Coefficients, *P* Values, and False Discovery Rate (FDR)-adjusted *P* Values for Phyla With Respect to PC (1st)

© 2022 Meyer K et al. *JAMA Network Open.*

eTable 69. Coefficients and *P* Values for Associations Between Bacteroidota:Firmicutes Ratio With Measures of Cognitive Function

eFigure. Beta-diversity (PCoA) Differences for Each Cognitive Function Assessment

This supplemental material has been provided by the authors to give readers additional information about their work.

eTable 1: CARDIA participant characteristics for individuals who completed cognitive assessments at Year 30 (2015-16), according to enrollment in the microbiome study

Characteristic	Microbiome Study	Yr 30	p-value[3]
Sample Size[1], n	597	2,550	
Age, years	55.2 (3.5)	55.1 (3.6)	0.42
Males, %	44.7	42.6	0.33
Black Americans, %	45.2	48.2	0.19
Educational Attainment, years	15.9 (2.6)	15.7 (2.6)	0.14
Field Center, %			
Birmingham, AL	16.1	24.4	<0.0001
Chicago, IL	48.9	15.3	
Minneapolis, MN	18.1	27	
Oakland, CA	16.9	33.4	
MoCA	24.4 (3.6)	23.6 (4.0)	<0.0001
DSST, no. correctly substituted	69.2 (16.9)	67.0 (17.0)	0.0068
Stroop, no. correctly assigned	22.2 (11.5)	23.2 (11.7)	0.023

© 2022 Meyer K et al. *JAMA Network Open.*

RAVLT, no. of words recalled	9.3 (1.9)	9.0 (2.0)	0.0044
Category fluency, no. of unique animals	20.6 (5.2)	20.2 (5.6)	0.2
Letter fluency, no. unique words	42.6 (12.3)	41.3 (13.2)	0.018
Current Smoking, %	13.1	14.2	0.62
Physical activity, intensity units, med (IQR)	269 (127-504)	252 (116-456)	0.18
BMI, kg/m^2	29.4 (6.2)	30.8 (7.4)	0.0002
Hypertension, %	35	40	0.026
Diabetes, %	15.4	17.9	0.13

Sample size represents the number of participants for whom a microbiome sample and at least 1 cognitive assessment is present.
Mean (SD) unless noted.
Differences in categorical variables assessed with chi-square and continuous variables with a non-parametric test for comparing means (or medians for physical activity).

© 2022 Meyer K et al. *JAMA Network Open.*

eTable 2: Descriptive statistics for the seven cognitive function measures

Variable	Min.	25th %	Median	75th %	Max.	Mean	SD	Missing, n
DSST	17	58	70	82	112	69.17	16.87	10
Stroop	0	15	20	27	107	22.24	11.54	18
RAVLT	2.8	8	9.4	10.6	14	9.3	1.94	10
Category fluency	6	17	20	24	41	20.56	5.22	10
Letter fluency	7	34	42	50	86	42.63	12.27	16
MOCA	8	22	25	27	30	24.38	3.64	11
PC (1st)	-5.52	-1.21	0.082	1.21	4.24	-0.0021	1.8	10

© 2022 Meyer K et al. *JAMA Network Open.*

eTable 3: Correlation coefficients for the seven cognitive function measures

	DSST	Stroop	RAVLT	Category fluency	Letter fluency	MOCA	PC (1st)
**DSST**	1	-0.47	0.53	0.38	0.41	0.53	0.74
**Stroop**		1	-0.42	-0.36	-0.37	-0.47	-0.66
**RAVLT**			1	0.44	0.44	0.64	0.79
**Category fluency**				1	0.52	0.49	0.71
**Letter fluency**					1	0.48	0.72
**MOCA**						1	0.81
**PC (1st)**							1

© 2022 Meyer K et al. *JAMA Network Open.*

eTable 4: Descriptive statistics for genera

Genus	Min.	25th %	Median	75th %	Max.	Mean	SD	Missing, n
**Acetanaerobacterium**	0.00	0.00	0.00	4.00	54.00	3.57	7.12	377
**Acidaminococcus**	0.00	0.00	0.00	245.50	34487.00	1126.60	3215.34	352
**Actinomyces**	0.00	0.00	0.00	8.00	131.00	6.96	13.70	312
**Adlercreutzia**	0.00	0.00	29.00	91.50	1308.00	81.61	141.96	173
**Agathobacter**	0.00	1496.00	3420.00	6653.00	42267.00	4974.00	5153.25	10
**Akkermansia**	0.00	0.00	39.00	890.50	23786.00	1082.70	2570.36	177
**Alistipes**	0.00	1154.00	2648.00	5553.00	41105.00	4106.00	4570.00	15
**Allisonella**	0.00	0.00	0.00	3.00	607.00	19.30	59.70	451
**Anaerofilum**	0.00	0.00	4.00	13.00	290.00	12.08	26.61	238
**Anaerostipes**	0.00	109.50	262.00	466.50	7313.00	394.30	571.70	15
**Anaerotruncus**	0.00	6.00	17.00	46.00	1977.00	59.48	156.50	86
**Bacteroides**	38.00	20482.00	33655.00	48447.00	125950.00	35733.00	20682.29	0
**Barnesiella**	0.00	0.00	5.00	993.50	10763.00	857.90	1653.97	256
**Bifidobacterium**	0.00	66.50	792.00	1996.50	31271.00	1718.60	3048.20	62
**Bilophila**	0.00	5.00	133.00	352.00	2196.00	230.90	286.50	140
**Blautia**	21.00	879.50	1325.00	2026.00	11968.00	1676.00	1357.75	0
**Butyricicoccus**	0.00	84.00	158.00	321.50	2300.00	266.50	303.55	3
**Butyricimonas**	0.00	0.00	288.00	770.00	4638.00	492.00	658.64	205
**Lachnospiraceae_CAG.56**	0.00	0.00	24.00	142.00	3396.00	111.30	234.98	279
**Candidatus_Soleaferrea**	0.00	0.00	6.00	12.00	141.00	9.59	13.24	167
**Caproiciproducens**	0.00	0.00	6.00	16.00	351.00	14.32	29.21	242
**Christensenellaceae_R.7_group**	0.00	8.00	324.00	1354.00	17455.00	1282.00	2451.28	109
**Clostridium_sensu_stricto_1**	0.00	0.00	6.00	62.50	8329.00	166.10	648.53	244
**Colidextribacter**	0.00	87.00	162.00	312.00	3774.00	267.30	367.58	16
**Collinsella**	0.00	145.50	785.00	1872.00	23889.00	1471.00	2154.67	74
**Coprobacter**	0.00	0.00	0.00	34.50	4093.00	112.60	355.08	411
**Coprococcus**	0.00	308.00	976.00	1750.00	9040.00	1303.00	1372.64	55
**Defluviitaleaceae_UCG.011**	0.00	0.00	4.00	18.00	211.00	15.09	27.80	257
**Desulfovibrio**	0.00	0.00	0.00	124.00	2978.00	190.90	432.29	338
**Dialister**	0.00	0.00	5.00	1033.00	12181.00	787.90	1547.59	260
**Eggerthellaceae_DNF00809**	0.00	0.00	0.00	8.00	284.00	8.23	25.39	417
**Dorea**	0.00	277.50	616.00	1166.50	6138.00	896.50	941.49	31
**Ruminococcaceae_DTU089**	0.00	3.00	17.00	50.00	500.00	44.57	74.59	140
**Eggerthella**	0.00	0.00	13.00	37.00	1324.00	44.71	115.18	214
**Eisenbergiella**	0.00	0.00	0.00	34.00	2094.00	38.29	155.28	340
**Erysipelatoclostridium**	0.00	0.00	9.00	46.50	1298.00	37.95	85.06	217
**Erysipelotrichaceae_UCG.003**	0.00	0.00	35.00	138.50	1585.00	104.00	167.61	215
**Escherichia.Shigella**	0.00	3.50	43.00	379.00	24295.00	619.20	1981.18	142
**Faecalibacterium**	0.00	4523.00	7736.00	11174.00	38698.00	8272.00	5528.14	8
**Anaerovoracaceae_Family_XIII_AD3011**	0.00	5.00	26.00	76.00	1649.00	66.17	140.71	128
**Anaerovoracaceae_Family_XIII_UCG.001**	0.00	0.00	8.00	22.00	179.00	16.41	24.09	223
**Lachnospiraceae_FD2005**	0.00	0.00	0.00	5.00	538.00	13.66	48.09	446
**Flavonifractor**	0.00	20.00	71.00	181.50	6012.00	193.00	466.57	91
**Fournierella**	0.00	0.00	0.00	3.00	984.00	17.13	73.21	449
**Frisingicoccus**	0.00	0.00	0.00	38.00	4251.00	57.55	219.86	377
**Fusicatenibacter**	0.00	263.50	761.00	1641.50	10538.00	1204.70	1445.36	58
**Fusobacterium**	0.00	0.00	0.00	7.50	12808.00	239.80	1032.22	405
**Lachnospiraceae_GCA.900066575**	0.00	0.00	23.00	46.00	576.00	34.34	46.62	154
**Gordonibacter**	0.00	0.00	0.00	18.00	2377.00	18.74	100.45	320
**Granulicatella**	0.00	0.00	0.00	8.00	156.00	6.41	13.17	319
**Haemophilus**	0.00	0.00	7.00	41.00	18435.00	130.10	815.80	244
**Harryflintia**	0.00	0.00	0.00	14.00	192.00	9.58	18.56	339
**Holdemanella**	0.00	0.00	0.00	40.00	1091.00	57.36	137.28	412
**Holdemania**	0.00	0.00	0.00	4.00	41.00	2.38	4.42	391
**Hungatella**	0.00	0.00	11.00	36.50	2844.00	46.88	177.81	265
**Hydrogenoanaerobacterium**	0.00	0.00	0.00	9.00	694.00	16.69	56.61	365
**Ruminococcaceae_Incertae_Sedis**	0.00	92.50	189.00	418.00	7385.00	400.80	671.29	1
**Intestinibacter**	0.00	0.00	4.00	74.50	1101.00	56.18	119.32	291
**Intestinimonas**	0.00	0.00	11.00	42.50	2260.00	58.89	161.23	235
**Lachnoclostridium**	29.00	709.50	1381.00	2502.00	44118.00	2106.00	2808.28	0
**Lachnospira**	0.00	1049.00	2647.00	4865.00	26458.00	3287.00	2999.04	22
**Lachnospiraceae_FCS020_group**	0.00	0.00	21.00	56.00	476.00	39.31	54.65	183
**Lachnospiraceae_ND3007_group**	0.00	11.50	234.00	689.00	6609.00	559.50	869.46	141
**Lachnospiraceae_NK4A136_group**	0.00	159.00	432.00	1061.00	7337.00	803.50	1020.25	34
**Lachnospiraceae_UCG.001**	0.00	0.00	0.00	86.00	2551.00	103.70	242.01	333
**Lachnospiraceae_UCG.004**	0.00	73.00	277.00	632.00	8042.00	474.70	701.26	88
**Lachnospiraceae_UCG.010**	0.00	28.00	78.00	218.50	2266.00	177.80	265.65	32
**Lactobacillus**	0.00	0.00	0.00	6.00	5999.00	90.10	496.58	369
**Lactococcus**	0.00	0.00	0.00	8.00	242.00	9.97	25.17	332
**Marvinbryantia**	0.00	35.50	207.00	483.50	5196.00	375.10	570.50	110
**Megasphaera**	0.00	0.00	0.00	9.00	27568.00	744.80	2777.85	377
**Monoglobus**	0.00	41.50	148.00	390.00	4614.00	317.70	503.83	54
**Moryella**	0.00	0.00	0.00	11.00	152.00	7.93	14.80	350
**Negativibacillus**	0.00	7.00	52.00	179.00	4235.00	162.90	313.92	136
**Oscillospiraceae_NK4A214_group**	0.00	0.00	181.00	717.50	10429.00	570.20	995.69	154
**Odoribacter**	0.00	7.00	527.00	1139.00	22999.00	930.20	1624.81	125
**Oscillibacter**	0.00	5.00	17.00	45.00	2155.00	59.01	169.32	142
**Oscillospira**	0.00	56.50	117.00	253.50	4305.00	234.00	380.56	12

© 2022 Meyer K et al. *JAMA Network Open.*

**Oxalobacter**	0.00	0.00	0.00	12.00	358.00	14.97	37.56	421
**Paludicola**	0.00	0.00	7.00	27.50	3336.00	34.38	154.77	205
**Parabacteroides**	0.00	828.50	1985.00	3795.00	37094.00	2838.40	3302.62	39
**Paraprevotella**	0.00	0.00	0.00	224.00	21406.00	647.40	1770.26	361
**Parasutterella**	0.00	2.00	103.00	1292.00	13304.00	902.90	1512.92	151
**Peptococcus**	0.00	0.00	0.00	6.50	487.00	14.83	49.27	426
**Phascolarctobacterium**	0.00	13.00	2661.00	5914.00	36868.00	4064.00	5104.79	64
**Phocea**	0.00	0.00	10.00	22.00	511.00	16.20	28.70	164
**Prevotella**	0.00	9.00	39.00	6458.00	92382.00	8905.00	17702.44	89
**Romboutsia**	0.00	0.00	14.00	56.00	2272.00	70.38	178.55	211
**Roseburia**	0.00	256.00	707.00	1432.00	11084.00	1107.00	1377.46	27
**Ruminococcus**	0.00	64.50	557.00	1682.50	17447.00	1329.30	2087.47	79
**Sellimonas**	0.00	0.00	0.00	23.00	1526.00	28.26	91.82	382
**Senegalimassilia**	0.00	0.00	0.00	14.00	1355.00	39.32	124.04	428
**Shuttleworthia**	0.00	0.00	0.00	24.00	857.00	26.43	76.44	392
**Slackia**	0.00	0.00	0.00	59.50	1001.00	56.57	124.19	377
**Streptococcus**	0.00	33.00	94.00	279.00	8091.00	394.40	965.16	13
**Subdoligranulum**	0.00	310.00	855.00	1625.00	11638.00	1279.00	1484.05	35
**Sutterella**	0.00	0.00	638.00	2277.00	20418.00	1525.00	2297.98	180
**Saccharimonadaceae_TM7x**	0.00	0.00	7.00	19.00	1370.00	18.41	62.61	189
**Turicibacter**	0.00	0.00	0.00	3.50	1613.00	15.44	86.01	429
**Tuzzerella**	0.00	0.00	0.00	17.00	2915.00	66.49	224.09	396
**Ruminococcaceae_UBA1819**	0.00	9.00	21.00	53.50	3350.00	74.45	221.69	61
**Oscillospiraceae_UCG.002**	0.00	105.00	793.00	2174.00	15158.00	1639.00	2277.40	100
**Oscillospiraceae_UCG.003**	0.00	0.00	146.00	430.50	6113.00	341.60	576.38	155
**Oscillospiraceae_UCG.005**	0.00	22.00	147.00	519.50	9152.00	487.60	900.36	81
**Butyricicoccaceae_UCG.009**	0.00	0.00	0.00	11.00	1702.00	13.88	74.48	360
**Veillonella**	0.00	9.00	38.00	149.50	15224.00	393.20	1398.70	72
**Victivallis**	0.00	0.00	0.00	21.00	2624.00	54.12	184.83	423

© 2022 Meyer K et al. *JAMA Network Open.*

eTable 5: Means (SDs) of seven cognitive function measures according to quartiles of the first two PCoA axes (MDS1 and MDS2).

	MDS1_quart1	MDS1_quart2	MDS1_quart3	MDS1_quart4	MDS2_quart1	MDS2_quart2	MDS2_quart3	MDS2_quart4
**DSST**	71.26(15.58)	69.54(17.26)	68.39(18.04)	67.53(16.39)	63.45(16.18)	67.78(16.02)	71.17(15.69)	74.30(17.74)
**MOCA**	24.71(3.53)	24.71(3.42)	24.09(3.74)	24.02(3.81)	23.35(3.89)	24.03(3.35)	24.64(3.54)	25.50(3.42)
**RAVLT**	9.29(2.14)	9.52(1.85)	9.23(1.98)	9.15(1.77)	8.59(1.74)	9.11(1.92)	9.46(1.82)	10.04(1.99)
**Stroop**	21.02(9.07)	20.92(10.98)	23.19(12.25)	23.80(13.25)	24.65(12.67)	23.33(9.81)	21.37(12.30)	19.62(10.61)
**Category fluency**	21.11(5.04)	21.34(5.29)	20.38(4.93)	19.40(5.46)	20.03(4.91)	20.09(5.30)	20.79(5.46)	21.32(5.15)
**Letter fluency**	43.59(12.04)	43.36(12.62)	41.93(12.93)	41.63(11.39)	40.85(12.25)	42.09(12.31)	43.22(12.64)	44.37(11.68)
**PC (1st)**	0.19(1.78)	0.22(1.75)	-0.14(1.86)	-0.28(1.75)	-0.58(1.72)	-0.25(1.66)	0.18(1.82)	0.64(1.76)

© 2022 Meyer K et al. *JAMA Network Open.*

eTable 6: Correlations between each of the first two axes ofthe PCoA (MDS1 and MDS2) with genera used in the analysis

Taxa	MDS1	MDS2
**Acetanaerobacterium**	0.086	0.29
**Acidaminococcus**	-0.063	-0.25
**Actinomyces**	0.063	-0.13
**Adlercreutzia**	-0.094	0.47
**Agathobacter**	-0.088	-0.14
**Akkermansia**	-0.24	0.47
**Alistipes**	-0.18	0.56
**Allisonella**	-0.25	-0.45
**Anaerofilum**	-0.090	0.39
**Anaerostipes**	0.18	0.14
**Anaerotruncus**	-0.11	0.61
**Bacteroides**	0.63	0.21
**Barnesiella**	-0.44	0.085
**Bifidobacterium**	0.11	-0.14
**Bilophila**	-0.12	0.076
**Blautia**	0.23	0.32
**Butyricicoccus**	-0.15	0.23
**Butyricimonas**	-0.25	-0.096
**Lachnospiraceae_CAG.56**	-0.39	-0.055
**Candidatus_Soleaferrea**	0.094	0.38
**Caproiciproducens**	-0.077	0.47
**Christensenellaceae_R.7_group**	-0.75	0.30
**Clostridium_sensu_stricto_1**	-0.20	-0.11
**Colidextribacter**	0.22	0.40
**Collinsella**	-0.24	-0.21
**Coprobacter**	-0.26	0.010
**Coprococcus**	-0.35	-0.083
**Defluviitaleaceae_UCG.011**	-0.38	0.49
**Desulfovibrio**	-0.49	-0.11
**Dialister**	0.070	0.088
**Eggerthellaceae_DNF00809**	-0.27	0.38
**Dorea**	-0.32	-0.15
**Ruminococcaceae_DTU089**	-0.14	0.60
**Eggerthella**	0.60	0.40
**Eisenbergiella**	0.45	0.27
**Erysipelatoclostridium**	0.53	0.21
**Erysipelotrichaceae_UCG.003**	-0.19	0.18
**Escherichia.Shigella**	0.30	-0.067
**Faecalibacterium**	-0.055	-0.0088
**Anaerovoracaceae_Family_XIII_AD301**	-0.58	0.49
**Anaerovoracaceae_Family_XIII_UCG.0**	-0.54	0.27
**Lachnospiraceae_FD2005**	-0.28	-0.079

© 2022 Meyer K et al. *JAMA Network Open.*

**Flavonifractor**	0.67	0.30
**Fournierella**	-0.30	-0.021
**Frisingicoccus**	0.12	0.48
**Fusicatenibacter**	-0.10	0.032
**Fusobacterium**	0.31	-0.27
**Lachnospiraceae_GCA.900066575**	-0.26	0.16
**Gordonibacter**	0.061	0.51
**Granulicatella**	0.28	-0.19
**Haemophilus**	-0.072	-0.32
**Harryflintia**	-0.15	0.53
**Holdemanella**	-0.48	-0.32
**Holdemania**	0.13	0.34
**Hungatella**	0.59	0.20
**Hydrogenoanaerobacterium**	-0.34	0.31
**Ruminococcaceae_Incertae_Sedis**	0.008	0.48
**Intestinibacter**	0.20	-0.063
**Intestinimonas**	-0.14	0.60
**Lachnoclostridium**	0.66	-0.18
**Lachnospira**	0.058	-0.050
**Lachnospiraceae_FCS020_group**	-0.61	0.12
**Lachnospiraceae_ND3007_group**	-0.51	-0.020
**Lachnospiraceae_NK4A136_group**	-0.46	0.16
**Lachnospiraceae_UCG.001**	-0.37	0.17
**Lachnospiraceae_UCG.004**	-0.0035	-0.28
**Lachnospiraceae_UCG.010**	-0.15	0.066
**Lactobacillus**	-0.035	-0.10
**Lactococcus**	0.033	0.14
**Marvinbryantia**	-0.42	0.082
**Megasphaera**	-0.053	-0.35
**Monoglobus**	-0.19	0.47
**Moryella**	-0.52	0.0087
**Negativibacillus**	-0.17	0.31
**Oscillospiraceae_NK4A214_group**	-0.66	0.36
**Odoribacter**	-0.36	0.20
**Oscillibacter**	-0.19	0.59
**Oscillospira**	0.24	0.54
**Oxalobacter**	-0.50	0.052
**Paludicola**	-0.34	0.56
**Parabacteroides**	0.026	0.16
**Paraprevotella**	-0.28	-0.32
**Parasutterella**	-0.036	0.17
**Peptococcus**	-0.33	0.19
**Phascolarctobacterium**	-0.13	-0.093
**Phocea**	0.35	0.10
**Prevotella**	-0.38	-0.45

© 2022 Meyer K et al. *JAMA Network Open.*

**Romboutsia**	-0.20	-0.075
**Roseburia**	-0.14	-0.011
**Ruminococcus**	-0.45	0.34
**Sellimonas**	-0.26	0.14
**Senegalimassilia**	-0.47	-0.34
**Shuttleworthia**	-0.22	0.35
**Slackia**	-0.46	-0.35
**Streptococcus**	0.046	-0.057
**Subdoligranulum**	-0.41	0.29
**Sutterella**	-0.19	-0.46
**Saccharimonadaceae_TM7x**	0.038	0.049
**Turicibacter**	-0.16	-0.071
**Tuzzerella**	0.26	0.21
**Ruminococcaceae_UBA1819**	0.25	0.61
**Oscillospiraceae_UCG.002**	-0.69	0.30
**Oscillospiraceae_UCG.003**	-0.55	-0.019
**Oscillospiraceae_UCG.005**	-0.70	0.42
**Butyricicoccaceae_UCG.009**	-0.42	0.31
**Veillonella**	0.12	-0.35
**Victivallis**	-0.53	-0.050

© 2022 Meyer K et al. *JAMA Network Open.*

eTable 7 : Multivariable-adjusted associations between gut microbial alpha and beta diversity and cognition measures in CARDIA

	PCA factor					MoCA					DSST					RAVLT			
	Shannon Diversity Index		Richness		PERMANOVA	Shannon Diversity Index		Richness		PERMANOVA	Shannon Diversity Index		Richness		PERMANOVA	Shannon Diversity Index		Richness	
	Beta Coefficient (95% CI)	p-value	Beta Coefficient (95% CI)	p-value	p-value	Beta Coefficient (95% CI)	p-value	Beta Coefficient (95% CI)	p-value	p-value	Beta Coefficient (95% CI)	p-value	Beta Coefficient (95% CI)	p-value	p-value	Beta Coefficient (95% CI)	p-value	Beta Coefficient (95% CI)	p-value
**Model 1**	0.14 (0.02,0.26)	0.024	0.16 (0.04,0.28)	0.009	0.001	0.05 (-0.21,0.31)	0.690	0.18 (-0.08,0.44)	0.186	0.001	1.82 (0.62,3.01)	0.003	1.76 (0.56,2.95)	0.004	0.001	0.14 (0.00,0.28)	0.042	0.11 (-0.03,0.24)	0.124
**Model 2**	0.11 (0.00,0.22)	0.060	0.08(-0.05,0.21)	0.242	0.001	-0.01 (-0.26,0.23)	0.923	0.09(-0.18,0.36)	0.519	0.001	1.56 (0.42,2.70)	0.007	1.00(-0.27,2.27)	0.123	0.001	0.11 (-0.02,0.24)	0.090	0.02(-0.13,0.15)	0.798
**Model 3**	0.08 (-0.04,0.20)	0.193	0.07 (-0.06,0.19)	0.284	0.001	-0.02 (-0.28,0.23)	0.869	0.07 (-0.19,0.33)	0.604	0.002	1.22 (0.01,2.42)	0.048	0.93 (-0.31,2.16)	0.142	0.001	0.08 (-0.06,0.22)	0.278	0.01 (-0.13,0.15)	0.888
**Model 4**	0.08 (-0.04,0.20)	0.207	0.06 (-0.06,0.19)	0.328	0.001	-0.02 (-0.28,0.23)	0.860	0.07 (-0.20,0.33)	0.623	0.001	1.22 (0.01,2.43)	0.048	0.94 (-0.31,2.18)	0.141	0.001	0.08 (-0.06,0.22)	0.286	0.01 (-0.14,0.15)	0.927
**Model 5**	0.08 (-0.04,0.20)	0.203	0.07 (-0.06,0.20)	0.288	0.001	-0.02 (-0.27,0.24)	0.891	0.08 (-0.19,0.35)	0.553	0.001	1.27 (0.05,2.48)	0.041	1.08 (-0.20,2.36)	0.097	0.001	0.08 (-0.06,0.22)	0.273	0.01 (-0.14,0.16)	0.865

Multivariable-adjusted regression models adjusted for:
Model 1: sequencing batch, age, race, gender, study center
Model 2: Model 1, with education
Model 3: Model 2, with physical activity, diet score, medication use, and smoking
Model 4: Model 3 with BMI
Model 5: Model 4 with hypertension and diabetes

© 2022 Meyer K et al. *JAMA Network Open.*

	Stroop					Category Fluency					Letter Fluency				
PERMANOVA	Shannon Diversity Index		Richness		PERMANOVA	Shannon Diversity Index		Richness		PERMANOVA	Shannon Diversity Index		Richness		PERMANOVA
p-value	Beta Coefficient (95% CI)	p-value	Beta Coefficient (95% CI)	p-value	p-value	Beta Coefficient (95% CI)	p-value	Beta Coefficient (95% CI)	p-value	p-value	Beta Coefficient (95% CI)	p-value	Beta Coefficient (95% CI)	p-value	p-value
0.001	-1.01 (-1.89,-0.14)	0.023	-1.14 (-2.02,-0.27)	0.011	0.001	0.41 (0.02,0.80)	0.039	0.28 (-0.12,0.67)	0.170	0.002	0.31 (-0.66,1.28)	0.534	0.84 (-0.13,1.81)	0.090	0.083
0.001	-0.93 (-1.79,-0.06)	0.036	-0.69(-1.60,0.23)	0.138	0.002	0.34 (-0.04,0.72)	0.076	0.10(-0.33,0.54)	0.642	0.003	0.16 (-0.78,1.11)	0.733	0.44(-0.62,1.49)	0.416	0.074
0.001	-0.75 (-1.65,0.14)	0.100	-0.69 (-1.60,0.23)	0.143	0.005	0.34 (-0.07,0.75)	0.106	0.08 (-0.34,0.50)	0.712	0.003	0.06 (-0.94,1.07)	0.906	0.38 (-0.65,1.41)	0.469	0.081
0.001	-0.75 (-1.65,0.15)	0.103	-0.68 (-1.60,0.25)	0.151	0.007	0.33 (-0.08,0.74)	0.118	0.05 (-0.37,0.48)	0.812	0.001	0.04 (-0.96,1.05)	0.930	0.35 (-0.69,1.38)	0.514	0.076
0.001	-0.75 (-1.65,0.16)	0.106	-0.79 (-1.74,0.16)	0.104	0.007	0.32 (-0.10,0.73)	0.133	0.04 (-0.40,0.48)	0.862	0.001	0.05 (-0.96,1.05)	0.928	0.31 (-0.75,1.37)	0.562	0.073

© 2022 Meyer K et al. *JAMA Network Open.*

eTable 8: PERMANOVA tests (p-values) for interaction between beta-diversity (PCoA) and sex (panel a) and race (panel b).

**Panel A: Sex**	**PC**		**DSST**	**MOCA**	**RAVLT**	**Stroop**	**Category fluency**	**Letter fluency**
Model 1		0.054	0.419	0.139	0.007	0.499	0.043	0.029
Model 2		0.052	0.492	0.13	0.011	0.449	0.056	0.034
Model 3		0.079	0.52	0.196	0.009	0.151	0.047	0.022
Model 4		0.172	0.741	0.341	0.029	0.134	0.077	0.062
Model 5		0.249	0.774	0.359	0.041	0.152	0.111	0.056
**Panel B: Race**	**PC**		**DSST**	**MOCA**	**RAVLT**	**Stroop**	**Category fluency**	**Letter fluency**
Model 1		0.193	0.29	0.746	0.293	0.286	0.626	0.35
Model 2		0.208	0.335	0.76	0.256	0.327	0.599	0.352
Model 3		0.179	0.323	0.652	0.264	0.251	0.537	0.321
Model 4		0.152	0.323	0.623	0.279	0.237	0.57	0.296
Model 5		0.185	0.369	0.733	0.342	0.295	0.552	0.279

Multivariable-adjusted regression models adjusted for:
Mode l 1: sequencing batch, age, race, gender, study center
Model 2: Model 1, with education
Model 3: Model 2, with physical activity, diet score, medication use, and smoking
Model 4: Model 3 with BMI
Model 5: Model 4 with hypertension and diabetes

© 2022 Meyer K et al. *JAMA Network Open.*

eTable 9: PERMANOVA test (p-value) for sex- (panel A & B) and race- (panel C & D) stratified results.

**Panel A**	**PC (1st)**	**DSST**	**MOCA**	**Male**<br>**RAVLT**	**Stroop**	**Category fluency**	**Letter fluency**
Model 1	0.01	0.004	0.028	0.002	0.286	0.126	0.11
Model 2	0.009	0.013	0.017	0.003	0.284	0.114	0.118
Model 3	0.011	0.01	0.023	0.003	0.544	0.101	0.114
Model 4	0.015	0.01	0.021	0.006	0.554	0.112	0.103
Model 5	0.01	0.017	0.022	0.003	0.545	0.112	0.107
**Panel B**							
				**Female**			
	**PC (1st)**	**DSST**	**MOCA**	**RAVLT**	**Stroop**	**Category fluency**	**Letter fluency**
Model 1	0.001	0.001	0.001	0.006	0.002	0.002	0.01
Model 2	0.001	0.001	0.002	0.005	0.001	0.001	0.006
Model 3	0.001	0.001	0.001	0.004	0.001	0.001	0.012
Model 4	0.001	0.001	0.001	0.006	0.002	0.001	0.005
Model 5	0.001	0.001	0.001	0.007	0.001	0.001	0.007
**Panel C**							
	**PC (1st)**	**DSST**	**MOCA**	**Black**<br>**RAVLT**	**Stroop**	**Category fluency**	**Letter fluency**
Model 1	0.026	0.007	0.177	0.118	0.125	0.106	0.202
Model 2	0.031	0.005	0.176	0.124	0.129	0.108	0.191
Model 3	0.031	0.007	0.184	0.12	0.253	0.083	0.168
Model 4	0.026	0.011	0.195	0.11	0.229	0.091	0.188
Model 5	0.033	0.001	0.174	0.111	0.322	0.086	0.173
**Panel D**							
	**PC (1st)**	**DSST**	**MOCA**	**White**<br>**RAVLT**	**Stroop**	**Category fluency**	**Letter fluency**
Model 1	0.022	0.012	0.087	0.017	0.098	0.017	0.742
Model 2	0.015	0.015	0.088	0.019	0.127	0.015	0.707
Model 3	0.01	0.012	0.077	0.013	0.042	0.013	0.717
Model 4	0.007	0.015	0.077	0.019	0.058	0.019	0.716
Model 5	0.015	0.007	0.08	0.021	0.047	0.01	0.691

Multivariable-adjusted regression models adjusted for:
Model 1: sequencing batch, age, race, gender, study center
Model 2: Model 1, with education
Model 3: Model 2, with physical activity, diet score, medication use, and smoking
Model 4: Model 3 with BMI
Model 5: Model 4 with hypertension and diabetes

© 2022 Meyer K et al. *JAMA Network Open.*

eTable 10: P-values for interaction between sex (panel A) and race (panel B) with alpha diversity measures (Shannon and richness)

**Panel A: Sex**<br>**PC (1st axis)**<br>Shannon	Richness		**Panel B: Race**<br>Shannon	Richness	
Model 1 0.36		0.88	0.75		0.93
Model 2 0.23		0.75	0.74		0.91
Model 3 0.21		0.78	0.92		0.96
Model 4 0.21		0.78	0.88		0.98
Model 5 0.20		0.70	0.96		0.98
**DSST**					
Shannon	Richness		Shannon	Richness	
Model 1 0.95		0.25	0.13		0.51
Model 2 0.82		0.27	0.12		0.50
Model 3 0.88		0.31	0.18		0.52
Model 4 0.87		0.31	0.18		0.53
Model 5 0.81		0.38	0.18		0.47
**MOCA**					
Shannon	Richness		Shannon	Richness	
Model 1 0.45		0.80	0.62		0.86
Model 2 0.31		0.89	0.59		0.84
Model 3 0.12		0.82	0.62		0.96
Model 4 0.12		0.81	0.63		0.96
Model 5 0.10		0.66	0.59		0.82
**RAVLT**					
Shannon	Richness		Shannon	Richness	
Model 1 0.89		0.50	0.29		0.02
Model 2 0.99		0.41	0.26		0.01
Model 3 0.94		0.66	0.30		0.02
Model 4 0.95		0.66	0.31		0.02
Model 5 0.91		0.61	0.29		0.03
**Stroop**					
Shannon	Richness		Shannon	Richness	
Model 1 0.86		0.76	0.47		0.17
Model 2 0.92		0.82	0.46		0.17
Model 3 0.69		0.77	0.70		0.31
Model 4 0.70		0.77	0.69		0.31
Model 5 0.70		0.67	0.66		0.23
**Category fluency**					
Shannon	Richness		Shannon	Richness	
Model 1 0.27		0.87	0.66		0.47
Model 2 0.21		0.79	0.66		0.46
Model 3 0.25		0.80	0.97		0.56
Model 4 0.25		0.79	0.92		0.54
Model 5 0.27		0.77	0.98		0.60
**Letter fluency**					
Shannon	Richness		Shannon	Richness	
Model 1 0.48		0.92	0.34		0.57
Model 2 0.38		0.83	0.32		0.55
Model 3 0.54		0.98	0.43		0.44
Model 4 0.54		0.99	0.41		0.43
Model 5 0.52		0.97	0.49		0.46

Multivariable-adjusted regression models adjusted for:
Model 1: sequencing batch, age, race, gender, study center
Model 2: Model 1, with education
Model 3: Model 2, with physical activity, diet score, medication use, and smoking

© 2022 Meyer K et al. *JAMA Network Open.*

Model 4: Model 3 with BMI
Model 5: Model 4 with hypertension and diabetes

© 2022 Meyer K et al. *JAMA Network Open.*

eTable 11: Beta-coefficients (95% CI) and p-values for alpha-diversity measures (Shannon and richness) within strata of sex (panel A) and race (panel B)

	Panel A: Female				Panel A: Male				Panel B: White				Panel B: Black			
	Shannon		Richness		Shannon		Richness		Shannon		Richness		Shannon		Richness	
	Beta Coeff	pValue	Beta Coeff	pValue	Beta Coeff	pValue	Beta Coeff	pValue	Beta Coeff	pValue	Beta Coeff	pValue	Beta Coeff	pValue	Beta Coeff	pValue
**PC (1st)**																
Model 1	0.19(0.03,0.36)	0.02	0.19(0.02,0.35)	0.03	0.09(-0.10,0.28)	0.36	0.14(-0.05,0.33)	0.14	0.14(-0.04,0.31)	0.13	0.18(0.00,0.36)	0.05	0.17(-0.01,0.35)	0.07	0.16(-0.01,0.33)	0.06
Model 2	0.18(0.02,0.33)	0.03	0.15(0.00,0.31)	0.06	0.02(-0.15,0.19)	0.83	0.08(-0.09,0.25)	0.38	0.10(-0.06,0.27)	0.21	0.14(-0.03,0.31)	0.10	0.12(-0.04,0.28)	0.14	0.10(-0.06,0.26)	0.21
Model 3	0.16(0.00,0.33)	0.06	0.12(-0.06,0.29)	0.19	-0.05(-0.23,0.14)	0.62	0.02(-0.17,0.20)	0.85	0.06(-0.11,0.23)	0.50	0.06(-0.13,0.24)	0.55	0.09(-0.09,0.27)	0.33	0.06(-0.11,0.24)	0.47
Model 4	0.16(-0.01,0.32)	0.06	0.10(-0.07,0.28)	0.25	-0.04(-0.23,0.14)	0.66	0.03(-0.15,0.22)	0.73	0.04(-0.13,0.22)	0.64	0.03(-0.15,0.21)	0.73	0.09(-0.09,0.27)	0.33	0.07(-0.11,0.25)	0.46
Model 5	0.16(-0.01,0.32)	0.06	0.13(-0.05,0.31)	0.16	-0.04(-0.22,0.15)	0.71	0.04(-0.15,0.22)	0.68	0.05(-0.13,0.22)	0.61	0.02(-0.16,0.21)	0.80	0.09(-0.09,0.27)	0.34	0.11(-0.08,0.29)	0.26
**MOCA**																
Model 1	0.15(-0.20,0.50)	0.39	0.18(-0.17,0.53)	0.32	-0.07(-0.49,0.35)	0.75	0.18(-0.23,0.60)	0.38	0.18(-0.14,0.50)	0.27	0.25(-0.08,0.58)	0.14	-0.03(-0.47,0.41)	0.89	0.13(-0.29,0.55)	0.54
Model 2	0.12(-0.21,0.44)	0.47	0.09(-0.23,0.42)	0.57	-0.19(-0.59,0.20)	0.33	0.07(-0.32,0.46)	0.74	0.12(-0.18,0.42)	0.43	0.17(-0.14,0.48)	0.29	-0.13(-0.54,0.28)	0.54	0.00(-0.39,0.39)	0.99
Model 3	0.17(-0.17,0.50)	0.33	0.11(-0.24,0.46)	0.55	-0.27(-0.69,0.14)	0.20	0.04(-0.38,0.45)	0.87	0.10(-0.23,0.42)	0.56	0.12(-0.22,0.46)	0.47	-0.15(-0.58,0.28)	0.48	0.00(-0.43,0.42)	0.99
Model 4	0.16(-0.18,0.50)	0.35	0.10(-0.26,0.45)	0.59	-0.26(-0.68,0.16)	0.22	0.07(-0.35,0.48)	0.76	0.06(-0.26,0.39)	0.70	0.08(-0.26,0.42)	0.64	-0.16(-0.59,0.27)	0.47	0.01(-0.41,0.44)	0.96
Model 5	0.18(-0.16,0.52)	0.31	0.17(-0.19,0.54)	0.35	-0.26(-0.68,0.15)	0.22	0.04(-0.38,0.46)	0.85	0.06(-0.26,0.39)	0.70	0.04(-0.30,0.39)	0.82	-0.14(-0.57,0.30)	0.53	0.12(-0.32,0.56)	0.60
**DSST**																
Model 1	1.94(0.27,3.60)	0.02	1.45(-0.23,3.13)	0.09	2.05(0.32,3.77)	0.02	2.66(0.97,4.35)	0.00	1.38(-0.29,3.05)	0.11	1.59(-0.14,3.32)	0.07	3.00(1.27,4.73)	0.00	2.45(0.79,4.12)	0.00
Model 2	1.83(0.21,3.46)	0.03	1.20(-0.44,2.85)	0.15	1.49(-0.11,3.10)	0.07	2.14(0.57,3.72)	0.01	1.15(-0.47,2.76)	0.16	1.28(-0.40,2.95)	0.13	2.64(1.01,4.27)	0.00	1.96(0.39,3.54)	0.01
Model 3	1.54(-0.19,3.27)	0.08	0.89(-0.90,2.69)	0.33	0.85(-0.84,2.55)	0.32	1.47(-0.20,3.14)	0.08	0.66(-1.09,2.41)	0.46	0.70(-1.13,2.53)	0.45	2.31(0.58,4.05)	0.01	1.69(-0.03,3.40)	0.05
Model 4	1.48(-0.24,3.19)	0.09	0.73(-1.06,2.52)	0.43	0.98(-0.71,2.66)	0.26	1.79(0.12,3.46)	0.04	0.48(-1.28,2.23)	0.59	0.47(-1.37,2.32)	0.61	2.30(0.57,4.03)	0.01	1.75(0.03,3.47)	0.05
Model 5	1.52(-0.20,3.23)	0.08	1.11(-0.74,2.96)	0.24	1.01(-0.68,2.69)	0.24	1.77(0.08,3.45)	0.04	0.52(-1.25,2.28)	0.57	0.45(-1.41,2.32)	0.63	2.35(0.61,4.10)	0.01	2.16(0.38,3.95)	0.02
**Category fluency**																
Model 1	0.62(0.09,1.15)	0.02	0.30(-0.23,0.84)	0.27	0.24(-0.37,0.86)	0.44	0.26(-0.35,0.87)	0.40	0.29(-0.28,0.86)	0.32	0.12(-0.47,0.72)	0.68	0.42(-0.14,0.98)	0.14	0.35(-0.18,0.89)	0.19
Model 2	0.58(0.07,1.09)	0.03	0.22(-0.30,0.74)	0.41	0.12(-0.49,0.72)	0.71	0.14(-0.45,0.74)	0.64	0.21(-0.34,0.76)	0.46	0.01(-0.56,0.59)	0.96	0.34(-0.20,0.88)	0.22	0.25(-0.28,0.77)	0.35
Model 3	0.62(0.06,1.18)	0.03	0.19(-0.39,0.77)	0.53	0.03(-0.63,0.70)	0.92	-0.04(-0.70,0.62)	0.90	0.28(-0.32,0.87)	0.36	-0.07(-0.70,0.57)	0.82	0.28(-0.33,0.89)	0.37	0.17(-0.43,0.77)	0.59
Model 4	0.61(0.05,1.17)	0.03	0.17(-0.42,0.75)	0.58	0.03(-0.64,0.70)	0.94	-0.06(-0.73,0.61)	0.85	0.25(-0.35,0.85)	0.41	-0.11(-0.74,0.52)	0.73	0.28(-0.33,0.90)	0.36	0.14(-0.46,0.74)	0.64
Model 5	0.59(0.03,1.15)	0.04	0.19(-0.41,0.80)	0.53	0.03(-0.64,0.71)	0.92	-0.07(-0.74,0.61)	0.85	0.25(-0.35,0.86)	0.41	-0.14(-0.77,0.50)	0.68	0.25(-0.37,0.86)	0.43	0.14(-0.49,0.76)	0.66
**Letter fluency**																
Model 1	0.56(-0.73,1.86)	0.39	0.94(-0.37,2.24)	0.16	-0.21(-1.75,1.33)	0.79	0.63(-0.89,2.15)	0.41	-0.18(-1.54,1.17)	0.79	0.58(-0.83,2.00)	0.42	0.66(-0.79,2.11)	0.37	1.09(-0.30,2.47)	0.12
Model 2	0.52(-0.76,1.79)	0.42	0.80(-0.49,2.09)	0.22	-0.59(-2.07,0.90)	0.44	0.28(-1.18,1.75)	0.70	-0.33(-1.66,1.00)	0.63	0.39(-1.01,1.78)	0.58	0.46(-0.95,1.87)	0.52	0.81(-0.54,2.15)	0.24
Model 3	0.37(-0.98,1.72)	0.59	0.57(-0.84,1.97)	0.43	-0.82(-2.44,0.79)	0.32	0.05(-1.55,1.65)	0.95	-0.57(-1.99,0.86)	0.43	-0.30(-1.80,1.19)	0.69	0.58(-0.94,2.10)	0.45	0.87(-0.62,2.36)	0.25
Model 4	0.34(-1.01,1.69)	0.62	0.49(-0.92,1.90)	0.49	-0.82(-2.44,0.80)	0.32	0.07(-1.54,1.69)	0.93	-0.72(-2.15,0.70)	0.32	-0.51(-2.02,1.00)	0.51	0.58(-0.94,2.10)	0.45	0.89(-0.60,2.38)	0.24
Model 5	0.33(-1.01,1.68)	0.63	0.41(-1.04,1.87)	0.58	-0.82(-2.45,0.81)	0.32	0.06(-1.59,1.70)	0.94	-0.66(-2.07,0.76)	0.36	-0.50(-2.01,1.01)	0.52	0.51(-1.03,2.04)	0.51	0.97(-0.58,2.52)	0.22
**Stroop**																
Model 1	-1.05(-2.16,0.06)	0.06	-1.20(-2.32,-0.08)	0.04	-1.30(-2.75,0.15)	0.08	-1.41(-2.84,0.01)	0.05	-0.85(-1.95,0.25)	0.13	-0.62(-1.77,0.52)	0.28	-1.55(-3.01,-0.10)	0.04	-1.94(-3.32,-0.55)	0.01
Model 2	-1.03(-2.14,0.08)	0.07	-1.14(-2.26,-0.03)	0.04	-1.07(-2.51,0.37)	0.14	-1.20(-2.62,0.21)	0.10	-0.81(-1.91,0.30)	0.15	-0.58(-1.72,0.57)	0.32	-1.39(-2.81,0.04)	0.06	-1.71(-3.07,-0.35)	0.01
Model 3	-1.10(-2.31,0.10)	0.07	-1.14(-2.39,0.11)	0.07	-0.58(-2.02,0.86)	0.43	-0.56(-1.98,0.86)	0.44	-0.63(-1.82,0.57)	0.30	-0.25(-1.50,1.00)	0.69	-0.90(-2.39,0.58)	0.23	-1.21(-2.66,0.23)	0.10
Model 4	-1.08(-2.29,0.12)	0.08	-1.07(-2.32,0.18)	0.09	-0.60(-2.04,0.85)	0.42	-0.61(-2.04,0.82)	0.40	-0.60(-1.81,0.61)	0.33	-0.21(-1.47,1.05)	0.74	-0.90(-2.40,0.59)	0.23	-1.22(-2.67,0.24)	0.10
Model 5	-1.07(-2.29,0.15)	0.08	-1.33(-2.64,-0.03)	0.05	-0.64(-2.08,0.81)	0.39	-0.66(-2.11,0.79)	0.37	-0.58(-1.79,0.63)	0.35	-0.15(-1.43,1.12)	0.82	-0.92(-2.42,0.58)	0.23	-1.59(-3.10,-0.09)	0.04
**RAVLT**																
	Shannon		Richness		Shannon		Richness		Shannon		Richness		Shannon		Richness	

© 2022 Meyer K et al. *JAMA Network Open.*

	Beta Coeff	pValue	Beta Coeff	pValue	Beta Coeff	pValue	Beta Coeff	pValue	Beta Coeff	pValue	Beta Coeff	pValue	Beta Coeff	pValue	Beta Coeff	pValue
Model 1	0.12(-0.06,0.31)	0.20	0.14(-0.04,0.33)	0.13	0.16(-0.05,0.36)	0.14	0.03(-0.17,0.24)	0.76	0.20(0.01,0.40)	0.04	0.28(0.08,0.48)	0.01	0.09(-0.11,0.29)	0.38	-0.05(-0.25,0.14)	0.59
Model 2	0.11(-0.07,0.29)	0.23	0.11(-0.07,0.29)	0.23	0.10(-0.10,0.30)	0.32	-0.02(-0.22,0.17)	0.82	0.18(-0.01,0.37)	0.06	0.25(0.05,0.44)	0.01	0.05(-0.14,0.24)	0.61	-0.11(-0.29,0.07)	0.23
Model 3	0.08(-0.11,0.27)	0.43	0.03(-0.17,0.23)	0.74	0.05(-0.16,0.26)	0.64	-0.05(-0.26,0.16)	0.64	0.11(-0.09,0.31)	0.28	0.14(-0.07,0.35)	0.18	0.02(-0.20,0.23)	0.89	-0.17(-0.38,0.04)	0.11
Model 4	0.07(-0.12,0.27)	0.45	0.02(-0.18,0.22)	0.82	0.06(-0.16,0.27)	0.59	-0.03(-0.25,0.18)	0.77	0.10(-0.10,0.30)	0.34	0.13(-0.08,0.33)	0.24	0.01(-0.20,0.23)	0.90	-0.17(-0.37,0.04)	0.12
Model 5	0.07(-0.12,0.27)	0.45	0.04(-0.16,0.25)	0.68	0.07(-0.14,0.29)	0.49	-0.01(-0.22,0.21)	0.96	0.10(-0.10,0.30)	0.33	0.11(-0.10,0.32)	0.31	0.02(-0.19,0.24)	0.83	-0.13(-0.35,0.09)	0.24

Shannon and richness were standardized to mean=0 and SD=1 for analysis. Multivariable-adjusted regression models adjusted for:
Model 1: sequencing batch, age, race, gender, study center
Model 2: Model 1, with education
Model 3: Model 2, with physical activity, diet score, medication use, and smoking
Model 4: Model 3 with BMI
Model 5: Model 4 with hypertension and diabetes

© 2022 Meyer K et al. *JAMA Network Open.*

eTable 12: Multivariable-adjusted regression coefficients, p-values, and false discovery rate (FDR)-adjusted p-values for genera with respect to DSST.

	**Model1**			**Model2**			**Model3**			**Model4**			**Model5**		
	**beta**	**p-value**	**FDR**	**beta**	**p-value**	**FDR**	**beta**	**p-value**	**FDR**	**beta**	**p-value**	**FDR**	**beta**	**p-value**	**FDR**
Acetanaerobacterium	-0.33	0.80	0.91	0.32	0.80	0.95	0.37	0.77	0.93	0.37	0.78	0.93	0.15	0.91	0.97
Acidaminococcus	-0.60	0.16	0.33	-0.16	0.70	0.92	0.11	0.80	0.94	0.11	0.80	0.94	0.11	0.81	0.96
Actinomyces	-0.44	0.70	0.87	-0.08	0.94	0.96	0.78	0.50	0.87	0.78	0.50	0.87	0.97	0.41	0.83
Adlercreutzia	1.29	0.06	0.18	1.14	0.08	0.35	1.12	0.11	0.75	1.12	0.11	0.74	1.06	0.13	0.79
Agathobacter	0.39	0.65	0.83	0.26	0.75	0.94	-0.36	0.67	0.93	-0.36	0.67	0.93	-0.30	0.73	0.96
Akkermansia	1.57	0.00	0.02	1.25	0.00	0.10	1.25	0.01	0.29	1.25	0.01	0.29	1.28	0.00	0.19
Alistipes	0.92	0.22	0.40	0.60	0.40	0.69	0.29	0.69	0.93	0.29	0.69	0.93	0.23	0.76	0.96
Allisonella	-1.91	0.03	0.13	-0.75	0.38	0.69	-0.35	0.70	0.93	-0.35	0.70	0.93	-0.29	0.75	0.96
Anaerofilum	2.05	0.04	0.14	1.03	0.28	0.66	1.04	0.29	0.87	1.05	0.29	0.87	1.13	0.25	0.83
Anaerostipes	0.54	0.61	0.83	0.07	0.94	0.96	0.02	0.98	1.00	0.02	0.98	1.00	0.10	0.93	0.98
Anaerotruncus	1.52	0.09	0.22	1.20	0.17	0.48	0.93	0.30	0.87	0.94	0.30	0.87	0.89	0.33	0.83
Bacteroides	-1.71	0.28	0.49	-2.14	0.16	0.48	-1.50	0.33	0.87	-1.50	0.33	0.87	-1.63	0.29	0.83
Barnesiella	1.37	0.00	0.02	1.37	0.00	0.03	1.11	0.01	0.29	1.11	0.01	0.29	1.18	0.01	0.19
Bifidobacterium	0.00	0.99	0.99	0.53	0.32	0.69	0.32	0.56	0.89	0.32	0.56	0.89	0.36	0.51	0.84
Bilophila	0.15	0.80	0.91	-0.11	0.83	0.95	-0.38	0.50	0.87	-0.38	0.50	0.87	-0.41	0.47	0.83
Blautia	4.54	0.02	0.12	3.75	0.05	0.32	4.44	0.02	0.45	4.44	0.02	0.45	4.88	0.01	0.38
Butyricicoccus	2.89	0.03	0.13	1.57	0.23	0.57	1.35	0.31	0.87	1.36	0.31	0.87	1.47	0.28	0.83
Butyricimonas	0.42	0.39	0.61	0.18	0.71	0.92	0.16	0.75	0.93	0.16	0.75	0.93	0.13	0.79	0.96
CAG.56	0.71	0.21	0.40	0.40	0.46	0.72	-0.11	0.85	0.96	-0.11	0.85	0.97	-0.07	0.91	0.97
Candidatus_Soleaferrea	1.34	0.24	0.44	0.89	0.41	0.70	0.88	0.43	0.87	0.88	0.43	0.87	0.77	0.50	0.84
Caproiciproducens	0.60	0.54	0.79	0.62	0.50	0.75	0.02	0.99	1.00	0.01	0.99	1.00	0.00	1.00	1.00
Christensenellaceae_R.7_group	1.57	0.00	0.02	1.18	0.01	0.14	0.78	0.11	0.75	0.79	0.11	0.74	0.86	0.08	0.65
Clostridium_sensu_stricto_1	0.19	0.75	0.89	0.33	0.57	0.82	0.00	1.00	1.00	0.00	1.00	1.00	0.01	0.99	1.00
Colidextribacter	-1.75	0.14	0.31	-2.25	0.05	0.32	-1.51	0.20	0.87	-1.51	0.20	0.87	-1.39	0.26	0.83
Collinsella	-1.05	0.05	0.16	-0.43	0.40	0.69	-0.35	0.50	0.87	-0.35	0.51	0.87	-0.30	0.57	0.89
Coprobacter	0.02	0.97	0.99	-0.29	0.60	0.84	-0.62	0.28	0.87	-0.63	0.28	0.87	-0.61	0.29	0.83
Coprococcus	1.29	0.04	0.15	1.22	0.04	0.32	1.10	0.08	0.67	1.10	0.08	0.67	1.15	0.07	0.65
Defluviitaleaceae_UCG.011	2.60	0.00	0.05	2.22	0.01	0.14	1.63	0.07	0.67	1.65	0.07	0.67	1.61	0.08	0.65
Desulfovibrio	0.31	0.56	0.81	0.38	0.44	0.72	0.39	0.46	0.87	0.39	0.46	0.87	0.41	0.44	0.83
Dialister	0.09	0.83	0.92	0.12	0.76	0.94	0.30	0.47	0.87	0.30	0.47	0.87	0.32	0.45	0.83
DNF00809	1.72	0.09	0.22	1.46	0.14	0.43	1.33	0.19	0.87	1.33	0.19	0.87	1.35	0.19	0.83
Dorea	-0.01	0.99	0.99	0.17	0.82	0.95	0.28	0.72	0.93	0.28	0.72	0.93	0.25	0.75	0.96
DTU089	0.82	0.33	0.53	0.63	0.43	0.72	0.40	0.63	0.93	0.40	0.63	0.93	0.42	0.62	0.93
Eggerthella	0.28	0.72	0.87	0.11	0.89	0.96	0.60	0.45	0.87	0.60	0.45	0.87	0.67	0.41	0.83
Eisenbergiella	0.05	0.96	0.99	0.07	0.93	0.96	0.36	0.65	0.93	0.37	0.65	0.93	0.46	0.57	0.89
Erysipelatoclostridium	-0.18	0.83	0.92	-0.12	0.88	0.96	0.51	0.55	0.89	0.51	0.55	0.89	0.54	0.53	0.86
Erysipelotrichaceae_UCG.003	0.64	0.31	0.51	0.52	0.38	0.69	0.02	0.97	1.00	0.02	0.98	1.00	0.05	0.94	0.98
Escherichia.Shigella	0.02	0.97	0.99	0.41	0.45	0.72	0.57	0.32	0.87	0.57	0.32	0.87	0.54	0.36	0.83
Faecalibacterium	1.79	0.03	0.13	1.39	0.08	0.35	0.92	0.27	0.87	0.92	0.27	0.87	1.00	0.23	0.83
Family_XIII_AD3011_group	2.29	0.00	0.05	1.64	0.03	0.26	1.19	0.13	0.77	1.21	0.13	0.76	1.27	0.11	0.77
Family_XIII_UCG.001	1.61	0.09	0.22	1.16	0.20	0.51	0.49	0.61	0.93	0.49	0.61	0.93	0.51	0.60	0.91
FD2005	0.34	0.72	0.87	0.66	0.45	0.72	0.70	0.44	0.87	0.70	0.44	0.87	0.70	0.45	0.83
Flavonifractor	-0.94	0.25	0.44	-0.88	0.25	0.62	-0.19	0.82	0.94	-0.19	0.82	0.94	-0.19	0.82	0.96
Fournierella	1.20	0.17	0.35	1.07	0.20	0.51	0.25	0.77	0.93	0.25	0.78	0.93	0.38	0.67	0.95
Frisingicoccus	-0.84	0.21	0.40	-1.07	0.09	0.35	-1.02	0.12	0.75	-1.03	0.12	0.74	-0.98	0.14	0.83
Fusicatenibacter	0.29	0.65	0.83	-0.03	0.96	0.97	-0.23	0.72	0.93	-0.23	0.72	0.93	-0.18	0.78	0.96
Fusobacterium	-0.90	0.35	0.56	-0.43	0.64	0.88	0.40	0.68	0.93	0.40	0.68	0.93	0.30	0.76	0.96
GCA.900066575	0.90	0.30	0.50	0.19	0.82	0.95	-0.61	0.48	0.87	-0.62	0.47	0.87	-0.69	0.43	0.83
Gordonibacter	0.22	0.81	0.91	-0.16	0.85	0.95	-0.36	0.67	0.93	-0.38	0.67	0.93	-0.40	0.65	0.95
Granulicatella	-0.66	0.57	0.81	-0.53	0.63	0.88	-0.40	0.72	0.93	-0.40	0.73	0.93	-0.30	0.79	0.96
Haemophilus	1.24	0.05	0.16	0.95	0.12	0.40	0.75	0.24	0.87	0.75	0.24	0.87	0.84	0.19	0.83
Harryflintia	0.45	0.64	0.83	0.25	0.79	0.95	0.15	0.88	0.99	0.14	0.88	1.00	0.05	0.96	1.00

© 2022 Meyer K et al. *JAMA Network Open.*

Holdemanella	0.22	0.74	0.88	0.41	0.51	0.75	0.52	0.42	0.87	0.52	0.42	0.87	0.52	0.42	0.83
Holdemania	1.02	0.50	0.75	1.04	0.47	0.74	1.54	0.31	0.87	1.54	0.31	0.87	1.34	0.38	0.83
Hungatella	-0.36	0.65	0.83	-0.10	0.90	0.96	0.49	0.54	0.89	0.48	0.54	0.89	0.59	0.46	0.83
Hydrogenoanaerobacterium	1.89	0.03	0.13	1.07	0.20	0.51	0.56	0.52	0.88	0.57	0.52	0.88	0.63	0.47	0.83
Incertae_Sedis	2.80	0.03	0.14	2.97	0.02	0.21	3.04	0.02	0.45	3.04	0.02	0.45	3.06	0.02	0.41
Intestinibacter	0.94	0.22	0.40	1.36	0.06	0.35	1.41	0.06	0.67	1.43	0.06	0.67	1.56	0.04	0.58
Intestinimonas	1.57	0.03	0.13	0.95	0.16	0.48	0.53	0.46	0.87	0.53	0.46	0.87	0.58	0.42	0.83
Lachnoclostridium	-4.19	0.01	0.05	-3.00	0.04	0.32	-1.24	0.42	0.87	-1.24	0.42	0.87	-1.33	0.39	0.83
Lachnospira	3.25	0.00	0.00	2.35	0.00	0.04	1.65	0.03	0.50	1.66	0.03	0.50	1.74	0.02	0.41
Lachnospiraceae_FCS020_group	3.70	0.00	0.00	2.89	0.00	0.01	2.62	0.00	0.10	2.62	0.00	0.10	2.67	0.00	0.09
Lachnospiraceae_ND3007_group	0.26	0.62	0.83	-0.20	0.69	0.92	-0.50	0.34	0.87	-0.50	0.34	0.87	-0.45	0.40	0.83
Lachnospiraceae_NK4A136_group	1.41	0.06	0.18	0.61	0.40	0.69	-0.19	0.81	0.94	-0.19	0.81	0.94	-0.14	0.86	0.97
Lachnospiraceae_UCG.001	1.62	0.00	0.05	1.37	0.01	0.14	1.14	0.04	0.64	1.15	0.04	0.64	1.20	0.03	0.52
Lachnospiraceae_UCG.004	1.41	0.02	0.12	1.12	0.06	0.35	0.89	0.15	0.83	0.90	0.15	0.82	0.85	0.18	0.83
Lachnospiraceae_UCG.010	0.32	0.72	0.87	-0.28	0.74	0.94	-0.46	0.60	0.93	-0.47	0.59	0.93	-0.52	0.56	0.89
Lactobacillus	-1.35	0.06	0.18	-0.60	0.39	0.69	-0.87	0.24	0.87	-0.87	0.24	0.87	-0.76	0.32	0.83
Lactococcus	0.49	0.62	0.83	0.17	0.85	0.95	0.05	0.96	1.00	0.05	0.96	1.00	0.01	0.99	1.00
Marvinbryantia	1.09	0.06	0.18	0.74	0.19	0.51	0.36	0.54	0.89	0.36	0.54	0.89	0.40	0.51	0.84
Megasphaera	-0.21	0.68	0.86	0.33	0.50	0.75	0.62	0.24	0.87	0.62	0.24	0.87	0.61	0.26	0.83
Monoglobus	2.25	0.00	0.05	1.88	0.01	0.14	1.21	0.12	0.75	1.22	0.12	0.74	1.32	0.09	0.66
Moryella	2.38	0.02	0.12	1.68	0.08	0.35	0.77	0.45	0.87	0.77	0.45	0.87	0.83	0.42	0.83
Negativibacillus	-0.64	0.31	0.51	-0.53	0.38	0.69	-0.53	0.40	0.87	-0.53	0.40	0.87	-0.55	0.38	0.83
NK4A214_group	0.80	0.11	0.25	0.45	0.34	0.69	0.05	0.93	1.00	0.05	0.93	1.00	0.13	0.80	0.96
Odoribacter	0.32	0.51	0.75	0.11	0.81	0.95	-0.20	0.68	0.93	-0.20	0.68	0.93	-0.22	0.66	0.95
Oscillibacter	1.96	0.02	0.12	1.61	0.04	0.32	1.47	0.08	0.67	1.48	0.08	0.67	1.53	0.07	0.65
Oscillospira	0.19	0.87	0.94	0.27	0.80	0.95	0.89	0.43	0.87	0.89	0.43	0.87	0.99	0.39	0.83
Oxalobacter	2.31	0.01	0.05	1.37	0.09	0.35	1.11	0.19	0.87	1.11	0.19	0.87	1.11	0.19	0.83
Paludicola	2.09	0.01	0.10	1.32	0.11	0.38	0.64	0.45	0.87	0.65	0.45	0.87	0.81	0.36	0.83
Parabacteroides	-0.44	0.47	0.71	-0.07	0.90	0.96	0.06	0.92	1.00	0.06	0.92	1.00	0.07	0.91	0.97
Paraprevotella	-0.62	0.16	0.33	-0.43	0.31	0.69	-0.36	0.43	0.87	-0.36	0.42	0.87	-0.32	0.47	0.83
Parasutterella	0.80	0.08	0.21	0.65	0.13	0.43	0.37	0.42	0.87	0.38	0.42	0.87	0.33	0.48	0.83
Peptococcus	1.47	0.10	0.23	0.84	0.33	0.69	0.60	0.50	0.87	0.60	0.51	0.87	0.70	0.44	0.83
Phascolarctobacterium	0.02	0.97	0.99	0.08	0.85	0.95	0.03	0.94	1.00	0.03	0.95	1.00	0.00	1.00	1.00
Phocea	0.33	0.76	0.89	0.03	0.98	0.98	0.34	0.75	0.93	0.34	0.75	0.93	0.35	0.75	0.96
Prevotella	-0.82	0.04	0.15	-0.53	0.17	0.48	-0.54	0.17	0.87	-0.54	0.17	0.87	-0.52	0.19	0.83
Romboutsia	0.12	0.87	0.94	0.38	0.57	0.82	0.21	0.77	0.93	0.21	0.76	0.93	0.30	0.67	0.95
Roseburia	1.63	0.05	0.16	1.38	0.08	0.35	0.72	0.38	0.87	0.72	0.38	0.87	0.77	0.36	0.83
Ruminococcus	1.37	0.01	0.07	0.92	0.07	0.35	0.46	0.39	0.87	0.46	0.39	0.87	0.57	0.30	0.83
Sellimonas	0.94	0.21	0.40	0.64	0.37	0.69	0.53	0.48	0.87	0.53	0.48	0.87	0.55	0.47	0.83
Senegalimassilia	0.35	0.63	0.83	0.38	0.57	0.82	-0.17	0.81	0.94	-0.17	0.81	0.94	-0.13	0.85	0.97
Shuttleworthia	1.23	0.12	0.26	0.26	0.74	0.94	0.01	0.99	1.00	0.00	1.00	1.00	0.10	0.90	0.97
Slackia	-0.72	0.25	0.45	-0.54	0.37	0.69	-0.72	0.25	0.87	-0.72	0.25	0.87	-0.80	0.21	0.83
Streptococcus	0.97	0.29	0.50	0.92	0.29	0.68	0.68	0.46	0.87	0.68	0.46	0.87	0.86	0.36	0.83
Subdoligranulum	1.46	0.03	0.13	1.08	0.10	0.36	0.57	0.40	0.87	0.56	0.40	0.87	0.58	0.40	0.83
Sutterella	-1.07	0.01	0.07	-0.71	0.07	0.35	-0.70	0.08	0.67	-0.70	0.08	0.67	-0.70	0.08	0.65
TM7x	0.45	0.64	0.83	-0.10	0.91	0.96	-0.02	0.98	1.00	-0.02	0.98	1.00	0.19	0.84	0.97
Turicibacter	-0.11	0.91	0.97	0.42	0.65	0.88	0.34	0.72	0.93	0.34	0.72	0.93	0.23	0.81	0.96
Tuzzerella	-1.38	0.04	0.14	-1.18	0.06	0.35	-0.89	0.17	0.87	-0.89	0.17	0.87	-0.95	0.15	0.83
UBA1819	0.05	0.96	0.99	-0.10	0.92	0.96	0.42	0.67	0.93	0.42	0.68	0.93	0.35	0.74	0.96
UCG.002	0.85	0.08	0.21	0.44	0.35	0.69	0.07	0.89	1.00	0.06	0.90	1.00	0.06	0.90	0.97
UCG.003	1.01	0.06	0.18	0.52	0.32	0.69	0.16	0.77	0.93	0.16	0.77	0.93	0.18	0.75	0.96
UCG.005	1.40	0.02	0.12	0.97	0.09	0.36	0.23	0.71	0.93	0.23	0.71	0.93	0.30	0.64	0.95
UCG.009	1.59	0.09	0.22	0.75	0.40	0.69	0.08	0.93	1.00	0.08	0.93	1.00	0.15	0.88	0.97
Veillonella	1.01	0.13	0.28	0.98	0.12	0.40	0.78	0.24	0.87	0.78	0.24	0.87	0.89	0.19	0.83
Victivallis	1.93	0.00	0.05	1.50	0.02	0.21	1.19	0.08	0.67	1.19	0.08	0.67	1.27	0.06	0.65

© 2022 Meyer K et al. *JAMA Network Open.*

Multivariable-adjusted regression models adjusted for:
Model 1: sequencing batch, age, race, gender, study center
Model 2: Model 1, with education
Model 3: Model 2, with physical activity, diet score, medication use, and smoking
Model 4: Model 3 with BMI
Model 5: Model 4 with hypertension and diabetes

© 2022 Meyer K et al. *JAMA Network Open.*

eTable 13: Multivariable-adjusted regression coefficients, p-values, and false discovery rate (FDR)-adjusted p-values for genera with respect to Stroop.

	Model1			Model2			Model3			Model4			Model5		
	beta	p-value	FDR	beta	p-value	FDR	beta	p-value	FDR	beta	p-value	FDR	beta	p-value	FDR
Acetanaerobacterium	0.04	0.97	1.00	-0.17	0.86	0.94	0.18	0.85	0.97	0.20	0.84	0.97	0.13	0.90	0.96
Acidaminococcus	0.41	0.18	0.51	0.26	0.41	0.81	-0.06	0.85	0.97	-0.07	0.84	0.97	-0.07	0.83	0.96
Actinomyces	-0.23	0.79	0.94	-0.36	0.67	0.89	-1.11	0.20	0.94	-1.11	0.20	0.94	-1.11	0.21	0.94
Adlercreutzia	0.10	0.85	0.96	0.14	0.79	0.94	0.21	0.68	0.97	0.22	0.67	0.95	0.22	0.68	0.94
Agathobacter	-0.43	0.50	0.80	-0.38	0.55	0.89	0.23	0.71	0.97	0.21	0.74	0.97	0.17	0.79	0.96
Akkermansia	-0.45	0.17	0.51	-0.35	0.29	0.74	-0.24	0.48	0.94	-0.24	0.48	0.94	-0.24	0.48	0.94
Alistipes	-0.36	0.50	0.80	-0.25	0.64	0.89	0.04	0.93	0.97	0.05	0.93	0.97	0.03	0.95	0.97
Allisonella	1.03	0.11	0.48	0.64	0.32	0.78	0.55	0.41	0.94	0.54	0.42	0.94	0.58	0.39	0.94
Anaerofilum	0.03	0.97	1.00	0.38	0.60	0.89	0.40	0.58	0.94	0.42	0.57	0.94	0.39	0.60	0.94
Anaerostipes	0.04	0.96	1.00	0.22	0.78	0.94	0.48	0.54	0.94	0.48	0.54	0.94	0.41	0.61	0.94
Anaerotruncus	-0.20	0.76	0.91	-0.09	0.89	0.96	0.26	0.69	0.97	0.30	0.66	0.95	0.21	0.76	0.95
Bacteroides	-1.03	0.38	0.78	-0.88	0.45	0.84	-0.71	0.53	0.94	-0.74	0.52	0.94	-0.66	0.57	0.94
Barnesiella	-0.64	0.04	0.32	-0.65	0.03	0.35	-0.59	0.06	0.94	-0.59	0.06	0.94	-0.63	0.05	0.94
Bifidobacterium	-0.30	0.46	0.80	-0.48	0.24	0.70	-0.41	0.30	0.94	-0.42	0.29	0.94	-0.42	0.31	0.94
Bilophila	-0.47	0.27	0.64	-0.38	0.36	0.78	-0.28	0.51	0.94	-0.28	0.50	0.94	-0.26	0.54	0.94
Blautia	-0.97	0.50	0.80	-0.69	0.63	0.89	-0.82	0.56	0.94	-0.81	0.57	0.94	-1.14	0.44	0.94
Butyricicoccus	-0.76	0.45	0.80	-0.29	0.77	0.94	0.56	0.57	0.94	0.55	0.58	0.94	0.54	0.60	0.94
Butyricimonas	-0.68	0.06	0.39	-0.60	0.09	0.62	-0.45	0.21	0.94	-0.45	0.21	0.94	-0.46	0.21	0.94
CAG.56	-0.60	0.14	0.51	-0.49	0.23	0.70	-0.10	0.82	0.97	-0.10	0.82	0.97	-0.14	0.75	0.95
Candidatus_Soleaferrea	0.30	0.72	0.90	0.44	0.60	0.89	0.02	0.98	0.98	0.02	0.98	0.98	-0.05	0.96	0.97
Caproiciproducens	-0.76	0.28	0.64	-0.76	0.28	0.73	-0.40	0.57	0.94	-0.40	0.58	0.94	-0.45	0.54	0.94
Christensenellaceae_R.7_group	-1.15	0.00	0.07	-1.03	0.00	0.15	-0.74	0.04	0.94	-0.74	0.04	0.94	-0.79	0.03	0.94
Clostridium_sensu_stricto_1	0.21	0.63	0.87	0.16	0.72	0.92	0.23	0.60	0.94	0.23	0.60	0.94	0.23	0.60	0.94
Colidextribacter	0.02	0.98	1.00	0.21	0.81	0.94	-0.14	0.88	0.97	-0.13	0.88	0.97	-0.33	0.72	0.95
Collinsella	0.05	0.89	0.99	-0.17	0.67	0.89	-0.06	0.89	0.97	-0.06	0.87	0.97	-0.04	0.92	0.97
Coprobacter	0.09	0.84	0.96	0.19	0.65	0.89	0.44	0.30	0.94	0.45	0.29	0.94	0.45	0.29	0.94
Coprococcus	-0.17	0.72	0.90	-0.16	0.74	0.92	-0.12	0.80	0.97	-0.12	0.80	0.97	-0.16	0.74	0.95
Defluviitaleaceae_UCG.011	-1.94	0.00	0.07	-1.82	0.01	0.16	-1.49	0.03	0.94	-1.49	0.03	0.94	-1.60	0.02	0.94
Desulfovibrio	-0.17	0.66	0.88	-0.19	0.61	0.89	-0.12	0.75	0.97	-0.11	0.78	0.97	-0.17	0.67	0.94
Dialister	0.19	0.52	0.80	0.18	0.54	0.89	0.25	0.42	0.94	0.24	0.43	0.94	0.24	0.44	0.94
DNF00809	-1.02	0.17	0.51	-0.94	0.21	0.70	-1.14	0.13	0.94	-1.13	0.14	0.94	-1.15	0.13	0.94
Dorea	-0.91	0.12	0.48	-0.96	0.10	0.62	-0.97	0.09	0.94	-0.98	0.09	0.94	-0.95	0.11	0.94
DTU089	-0.22	0.73	0.90	-0.16	0.80	0.94	-0.09	0.88	0.97	-0.08	0.90	0.97	-0.17	0.79	0.96
Eggerthella	0.61	0.30	0.65	0.68	0.24	0.70	0.28	0.63	0.94	0.29	0.62	0.94	0.28	0.64	0.94
Eisenbergiella	-0.24	0.69	0.90	-0.25	0.68	0.89	-0.59	0.32	0.94	-0.58	0.34	0.94	-0.59	0.33	0.94
Erysipelatoclostridium	-0.04	0.96	1.00	-0.06	0.92	0.96	-0.34	0.60	0.94	-0.32	0.61	0.94	-0.32	0.61	0.94
Erysipelotrichaceae_UCG.003	-0.59	0.19	0.51	-0.54	0.23	0.70	-0.32	0.49	0.94	-0.31	0.50	0.94	-0.35	0.45	0.94
Escherichia.Shigella	0.21	0.61	0.86	0.08	0.84	0.94	-0.02	0.96	0.97	-0.03	0.95	0.97	-0.01	0.99	0.99
Faecalibacterium	-1.94	0.00	0.07	-1.80	0.00	0.15	-1.56	0.01	0.94	-1.57	0.01	0.94	-1.65	0.01	0.89
Family_XIII_AD3011_group	-0.93	0.10	0.48	-0.70	0.21	0.70	-0.08	0.89	0.97	-0.06	0.92	0.97	-0.12	0.85	0.96
Family_XIII_UCG.001	-0.94	0.18	0.51	-0.77	0.27	0.73	-0.47	0.51	0.94	-0.45	0.53	0.94	-0.53	0.46	0.94
FD2005	-0.61	0.36	0.76	-0.72	0.28	0.73	-0.36	0.59	0.94	-0.37	0.59	0.94	-0.44	0.52	0.94
Flavonifractor	0.31	0.60	0.86	0.28	0.63	0.89	-0.30	0.62	0.94	-0.30	0.61	0.94	-0.34	0.58	0.94
Fournierella	-0.35	0.58	0.86	-0.31	0.63	0.89	0.17	0.79	0.97	0.19	0.77	0.97	0.10	0.87	0.96
Frisingicoccus	0.32	0.51	0.80	0.40	0.40	0.81	0.53	0.27	0.94	0.55	0.26	0.94	0.56	0.25	0.94
Fusicatenibacter	-0.54	0.26	0.64	-0.43	0.36	0.78	-0.36	0.46	0.94	-0.38	0.44	0.94	-0.39	0.43	0.94
Fusobacterium	1.35	0.06	0.39	1.18	0.09	0.62	0.93	0.19	0.94	0.95	0.18	0.94	0.90	0.21	0.94
GCA.900066575	-1.33	0.03	0.32	-1.09	0.08	0.62	-0.64	0.31	0.94	-0.63	0.32	0.94	-0.73	0.26	0.94
Gordonibacter	0.16	0.80	0.94	0.28	0.66	0.89	0.52	0.42	0.94	0.55	0.39	0.94	0.56	0.39	0.94
Granulicatella	-0.11	0.89	0.99	-0.18	0.83	0.94	-0.61	0.47	0.94	-0.62	0.47	0.94	-0.61	0.48	0.94
Haemophilus	-0.51	0.27	0.64	-0.41	0.37	0.78	-0.32	0.50	0.94	-0.32	0.50	0.94	-0.36	0.45	0.94
Harryflintia	-0.36	0.62	0.86	-0.29	0.68	0.89	0.04	0.96	0.97	0.05	0.94	0.97	-0.06	0.93	0.97

© 2022 Meyer K et al. *JAMA Network Open.*

Holdemanella	0.03	0.96	1.00	-0.04	0.92	0.96	0.16	0.73	0.97	0.16	0.74	0.97	0.17	0.72	0.95
Holdemania	-0.18	0.87	0.98	-0.20	0.86	0.94	-0.57	0.61	0.94	-0.55	0.62	0.94	-0.62	0.58	0.94
Hungatella	0.90	0.12	0.48	0.82	0.15	0.70	0.24	0.68	0.97	0.25	0.67	0.95	0.26	0.66	0.94
Hydrogenoanaerobacterium	-1.40	0.03	0.28	-1.14	0.07	0.62	-0.93	0.15	0.94	-0.92	0.15	0.94	-0.94	0.15	0.94
Incertae_Sedis	-0.80	0.41	0.80	-0.86	0.37	0.78	-0.93	0.34	0.94	-0.92	0.35	0.94	-1.00	0.31	0.94
Intestinibacter	-0.56	0.32	0.68	-0.70	0.21	0.70	-0.71	0.21	0.94	-0.74	0.19	0.94	-0.74	0.20	0.94
Intestinimonas	-0.72	0.17	0.51	-0.52	0.32	0.78	-0.37	0.49	0.94	-0.35	0.51	0.94	-0.39	0.48	0.94
Lachnoclostridium	2.85	0.01	0.14	2.47	0.03	0.35	1.79	0.11	0.94	1.77	0.12	0.94	1.88	0.10	0.94
Lachnospira	-1.65	0.00	0.07	-1.36	0.01	0.29	-1.01	0.07	0.94	-1.03	0.07	0.94	-1.15	0.04	0.94
Lachnospiraceae_FCS020_group	-1.50	0.01	0.14	-1.23	0.03	0.35	-0.63	0.29	0.94	-0.62	0.29	0.94	-0.63	0.30	0.94
Lachnospiraceae_ND3007_group	-0.29	0.45	0.80	-0.13	0.73	0.92	0.11	0.77	0.97	0.11	0.79	0.97	0.07	0.87	0.96
Lachnospiraceae_NK4A136_group	0.00	0.99	1.00	0.29	0.60	0.89	1.04	0.07	0.94	1.03	0.07	0.94	0.97	0.10	0.94
Lachnospiraceae_UCG.001	-0.58	0.16	0.51	-0.50	0.22	0.70	-0.06	0.88	0.97	-0.05	0.90	0.97	-0.09	0.84	0.96
Lachnospiraceae_UCG.004	-0.17	0.71	0.90	-0.06	0.90	0.96	0.12	0.79	0.97	0.11	0.80	0.97	0.14	0.76	0.95
Lachnospiraceae_UCG.010	-0.20	0.76	0.91	0.02	0.97	0.98	0.57	0.38	0.94	0.58	0.37	0.94	0.60	0.36	0.94
Lactobacillus	0.29	0.59	0.86	0.01	0.98	0.98	-0.04	0.94	0.97	-0.06	0.91	0.97	0.02	0.97	0.98
Lactococcus	-0.25	0.73	0.90	-0.14	0.84	0.94	0.31	0.68	0.97	0.32	0.66	0.95	0.42	0.56	0.94
Marvinbryantia	0.02	0.97	1.00	0.15	0.73	0.92	0.41	0.35	0.94	0.42	0.34	0.94	0.37	0.40	0.94
Megasphaera	0.20	0.59	0.86	0.01	0.98	0.98	-0.41	0.30	0.94	-0.41	0.29	0.94	-0.34	0.39	0.94
Monoglobus	-1.73	0.00	0.07	-1.61	0.00	0.15	-0.72	0.21	0.94	-0.71	0.22	0.94	-0.81	0.17	0.94
Moryella	-1.08	0.14	0.51	-0.83	0.26	0.73	-0.04	0.96	0.97	-0.04	0.96	0.97	-0.10	0.90	0.96
Negativibacillus	-0.20	0.67	0.89	-0.23	0.62	0.89	-0.41	0.38	0.94	-0.41	0.38	0.94	-0.41	0.38	0.94
NK4A214_group	-0.58	0.11	0.48	-0.46	0.21	0.70	-0.12	0.76	0.97	-0.11	0.77	0.97	-0.15	0.69	0.94
Odoribacter	0.00	1.00	1.00	0.07	0.85	0.94	0.20	0.57	0.94	0.21	0.57	0.94	0.15	0.68	0.94
Oscillibacter	-1.09	0.08	0.41	-0.97	0.11	0.62	-0.81	0.19	0.94	-0.80	0.20	0.94	-0.83	0.19	0.94
Oscillospira	-0.62	0.47	0.80	-0.65	0.44	0.84	-0.87	0.30	0.94	-0.86	0.31	0.94	-1.04	0.23	0.94
Oxalobacter	-0.89	0.14	0.51	-0.57	0.35	0.78	0.10	0.88	0.97	0.10	0.87	0.97	0.11	0.87	0.96
Paludicola	-1.11	0.07	0.41	-0.85	0.17	0.70	-0.32	0.62	0.94	-0.29	0.65	0.95	-0.29	0.66	0.94
Parabacteroides	-0.09	0.83	0.96	-0.22	0.62	0.89	-0.25	0.57	0.94	-0.25	0.56	0.94	-0.27	0.54	0.94
Paraprevotella	0.23	0.47	0.80	0.17	0.60	0.89	0.16	0.63	0.94	0.17	0.61	0.94	0.18	0.59	0.94
Parasutterella	-0.82	0.01	0.17	-0.77	0.02	0.32	-0.51	0.14	0.94	-0.51	0.14	0.94	-0.54	0.12	0.94
Peptococcus	-0.71	0.28	0.64	-0.50	0.44	0.84	0.03	0.96	0.97	0.05	0.94	0.97	0.09	0.90	0.96
Phascolarctobacterium	-0.42	0.19	0.51	-0.45	0.16	0.70	-0.42	0.19	0.94	-0.41	0.20	0.94	-0.43	0.19	0.94
Phocea	1.02	0.20	0.51	1.13	0.15	0.70	1.24	0.12	0.94	1.23	0.12	0.94	1.19	0.14	0.94
Prevotella	0.55	0.06	0.39	0.44	0.13	0.66	0.33	0.27	0.94	0.32	0.27	0.94	0.28	0.34	0.94
Romboutsia	-0.40	0.44	0.80	-0.50	0.33	0.78	-0.58	0.27	0.94	-0.59	0.26	0.94	-0.62	0.24	0.94
Roseburia	-0.42	0.49	0.80	-0.33	0.57	0.89	-0.06	0.92	0.97	-0.07	0.91	0.97	-0.18	0.77	0.95
Ruminococcus	-0.40	0.30	0.65	-0.25	0.51	0.89	0.05	0.90	0.97	0.05	0.89	0.97	0.03	0.94	0.97
Sellimonas	-0.45	0.42	0.80	-0.35	0.51	0.89	-0.54	0.32	0.94	-0.53	0.34	0.94	-0.55	0.32	0.94
Senegalimassilia	-0.32	0.54	0.82	-0.34	0.52	0.89	-0.06	0.92	0.97	-0.05	0.92	0.97	-0.09	0.86	0.96
Shuttleworthia	-1.03	0.07	0.41	-0.71	0.22	0.70	-0.41	0.48	0.94	-0.40	0.49	0.94	-0.37	0.53	0.94
Slackia	-0.65	0.16	0.51	-0.72	0.12	0.62	-0.40	0.40	0.94	-0.40	0.39	0.94	-0.42	0.38	0.94
Streptococcus	-1.07	0.11	0.48	-1.06	0.11	0.62	-1.25	0.07	0.94	-1.25	0.07	0.94	-1.19	0.09	0.94
Subdoligranulum	-1.02	0.04	0.32	-0.89	0.07	0.62	-0.84	0.09	0.94	-0.84	0.09	0.94	-0.91	0.07	0.94
Sutterella	0.26	0.39	0.78	0.13	0.66	0.89	-0.08	0.80	0.97	-0.08	0.80	0.97	-0.06	0.83	0.96
TM7x	-0.40	0.57	0.85	-0.22	0.75	0.92	-0.26	0.70	0.97	-0.26	0.71	0.97	-0.30	0.67	0.94
Turicibacter	-0.47	0.51	0.80	-0.66	0.34	0.78	-1.01	0.15	0.94	-1.02	0.15	0.94	-1.04	0.14	0.94
Tuzzerella	0.67	0.16	0.51	0.61	0.20	0.70	0.46	0.35	0.94	0.46	0.34	0.94	0.48	0.34	0.94
UBA1819	-0.50	0.50	0.80	-0.45	0.54	0.89	-1.00	0.17	0.94	-0.99	0.18	0.94	-1.09	0.15	0.94
UCG.002	-0.16	0.64	0.87	-0.01	0.97	0.98	0.32	0.38	0.94	0.34	0.36	0.94	0.28	0.45	0.94
UCG.003	-0.68	0.09	0.45	-0.52	0.19	0.70	-0.32	0.43	0.94	-0.32	0.43	0.94	-0.31	0.45	0.94
UCG.005	-1.18	0.01	0.13	-1.04	0.02	0.31	-0.57	0.22	0.94	-0.56	0.23	0.94	-0.65	0.17	0.94
UCG.009	-0.88	0.20	0.51	-0.60	0.38	0.79	-0.22	0.75	0.97	-0.21	0.76	0.97	-0.28	0.69	0.94
Veillonella	-0.06	0.90	0.99	-0.05	0.91	0.96	0.05	0.92	0.97	0.04	0.93	0.97	0.08	0.88	0.96
Victivallis	-0.96	0.05	0.38	-0.82	0.09	0.62	-0.40	0.42	0.94	-0.39	0.44	0.94	-0.38	0.46	0.94

© 2022 Meyer K et al. *JAMA Network Open.*

Multivariable-adjusted regression models adjusted for:
Model 1: sequencing batch, age, race, gender, study center
Model 2: Model 1, with education
Model 3: Model 2, with physical activity, diet score, medication use, and smoking
Model 4: Model 3 with BMI
Model 5: Model 4 with hypertension and diabetes

© 2022 Meyer K et al. *JAMA Network Open.*

eTable 14: Multivariable-adjusted regression coefficients, p-values, and false discovery rate (FDR)-adjusted p-values for genera with respect to RAVLT.

	Model1			Model2			Model3			Model4			Model5		
	beta	p-value	FDR	beta	p-value	FDR	beta	p-value	FDR	beta	p-value	FDR	beta	p-value	FDR
Acetanaerobacterium	-0.04	0.77	0.89	0.03	0.85	0.94	-0.09	0.53	0.88	-0.10	0.52	0.86	-0.11	0.45	0.79
Acidaminococcus	-0.16	0.00	0.04	-0.11	0.02	0.24	-0.09	0.09	0.53	-0.09	0.09	0.54	-0.09	0.09	0.52
Actinomyces	-0.11	0.39	0.60	-0.07	0.56	0.80	-0.04	0.75	0.93	-0.04	0.75	0.94	-0.04	0.80	0.98
Adlercreutzia	0.18	0.03	0.12	0.16	0.03	0.24	0.13	0.10	0.53	0.13	0.10	0.54	0.13	0.11	0.52
Agathobacter	-0.11	0.26	0.47	-0.13	0.18	0.46	-0.17	0.08	0.53	-0.17	0.09	0.54	-0.16	0.10	0.52
Akkermansia	0.10	0.05	0.16	0.06	0.20	0.49	0.05	0.38	0.76	0.05	0.38	0.75	0.05	0.39	0.79
Alistipes	0.21	0.02	0.09	0.17	0.04	0.24	0.10	0.26	0.76	0.10	0.26	0.75	0.09	0.28	0.76
Allisonella	-0.24	0.02	0.09	-0.11	0.25	0.57	-0.09	0.39	0.76	-0.09	0.40	0.75	-0.08	0.42	0.79
Anaerofilum	0.25	0.03	0.12	0.14	0.21	0.50	0.12	0.28	0.76	0.12	0.29	0.75	0.12	0.28	0.76
Anaerostipes	0.06	0.65	0.81	0.00	0.97	0.99	-0.01	0.94	0.99	-0.01	0.94	0.99	0.00	0.98	1.00
Anaerotruncus	0.04	0.73	0.87	0.00	0.99	0.99	-0.06	0.53	0.88	-0.07	0.49	0.85	-0.07	0.49	0.81
Bacteroides	0.08	0.68	0.83	0.03	0.87	0.94	0.01	0.96	0.99	0.02	0.93	0.99	0.00	0.99	1.00
Barnesiella	0.11	0.02	0.09	0.11	0.01	0.24	0.09	0.06	0.53	0.09	0.06	0.54	0.10	0.05	0.47
Bifidobacterium	0.00	0.95	0.98	0.05	0.37	0.70	0.05	0.43	0.77	0.05	0.42	0.75	0.05	0.41	0.79
Bilophila	0.14	0.04	0.14	0.11	0.09	0.33	0.10	0.13	0.53	0.10	0.13	0.54	0.09	0.15	0.54
Blautia	0.07	0.75	0.89	-0.02	0.93	0.99	0.01	0.96	0.99	0.01	0.97	1.00	0.04	0.85	0.99
Butyricicoccus	0.10	0.50	0.68	-0.05	0.76	0.92	-0.13	0.41	0.76	-0.13	0.41	0.75	-0.12	0.45	0.79
Butyricimonas	0.05	0.35	0.57	0.02	0.64	0.88	0.03	0.62	0.90	0.03	0.61	0.88	0.03	0.63	0.92
CAG.56	0.08	0.21	0.43	0.05	0.46	0.75	0.02	0.76	0.93	0.02	0.76	0.94	0.02	0.71	0.92
Candidatus_Soleaferrea	-0.05	0.68	0.83	-0.10	0.41	0.71	-0.11	0.40	0.76	-0.11	0.39	0.75	-0.12	0.36	0.77
Caproiciproducens	0.02	0.87	0.93	0.02	0.85	0.94	-0.05	0.66	0.90	-0.05	0.65	0.88	-0.05	0.64	0.92
Christensenellaceae_R.7_group	0.10	0.06	0.17	0.06	0.26	0.58	0.02	0.74	0.93	0.02	0.77	0.94	0.02	0.72	0.92
Clostridium_sensu_stricto_1	0.11	0.13	0.31	0.12	0.07	0.30	0.11	0.11	0.53	0.11	0.11	0.54	0.11	0.13	0.52
Colidextribacter	-0.20	0.14	0.33	-0.26	0.05	0.27	-0.32	0.02	0.53	-0.33	0.02	0.54	-0.32	0.02	0.47
Collinsella	-0.20	0.00	0.04	-0.13	0.03	0.24	-0.12	0.04	0.53	-0.12	0.05	0.54	-0.12	0.05	0.47
Coprobacter	0.00	0.96	0.98	-0.03	0.62	0.86	-0.06	0.35	0.76	-0.06	0.34	0.75	-0.06	0.35	0.77
Coprococcus	0.08	0.27	0.47	0.07	0.29	0.63	0.05	0.52	0.88	0.05	0.52	0.86	0.05	0.48	0.81
Defluviitaleaceae_UCG.011	0.27	0.01	0.08	0.23	0.02	0.24	0.22	0.04	0.53	0.22	0.04	0.54	0.22	0.04	0.47
Desulfovibrio	-0.06	0.31	0.54	-0.05	0.36	0.69	-0.07	0.28	0.76	-0.07	0.25	0.75	-0.07	0.29	0.76
Dialister	0.01	0.85	0.92	0.01	0.79	0.92	0.00	0.93	0.99	0.00	0.94	0.99	0.00	0.94	0.99
DNF00809	0.10	0.39	0.60	0.07	0.52	0.78	0.06	0.64	0.90	0.05	0.65	0.88	0.05	0.66	0.92
Dorea	-0.07	0.44	0.64	-0.05	0.57	0.80	-0.05	0.61	0.90	-0.05	0.62	0.88	-0.05	0.59	0.92
DTU089	-0.02	0.84	0.92	-0.04	0.66	0.88	-0.05	0.57	0.90	-0.06	0.55	0.88	-0.05	0.62	0.92
Eggerthella	0.12	0.21	0.42	0.10	0.27	0.59	0.09	0.31	0.76	0.09	0.32	0.75	0.10	0.28	0.76
Eisenbergiella	-0.06	0.51	0.68	-0.06	0.50	0.78	-0.07	0.42	0.77	-0.08	0.39	0.75	-0.07	0.46	0.79
Erysipelatoclostridium	0.08	0.41	0.60	0.09	0.35	0.69	0.10	0.33	0.76	0.09	0.35	0.75	0.09	0.34	0.77
Erysipelotrichaceae_UCG.003	0.01	0.85	0.92	0.00	0.99	0.99	-0.03	0.65	0.90	-0.03	0.64	0.88	-0.03	0.65	0.92
Escherichia.Shigella	-0.04	0.53	0.70	0.00	0.98	0.99	0.01	0.88	0.99	0.01	0.87	0.99	0.01	0.89	0.99
Faecalibacterium	0.20	0.03	0.13	0.16	0.08	0.32	0.16	0.10	0.53	0.16	0.10	0.54	0.17	0.09	0.52
Family_XIII_AD3011_group	0.17	0.05	0.16	0.10	0.25	0.57	0.01	0.88	0.99	0.01	0.92	0.99	0.01	0.89	0.99
Family_XIII_UCG.001	0.12	0.27	0.47	0.07	0.50	0.78	0.04	0.71	0.91	0.04	0.75	0.94	0.04	0.71	0.92
FD2005	-0.06	0.55	0.72	-0.03	0.79	0.92	-0.04	0.70	0.91	-0.04	0.71	0.93	-0.04	0.72	0.92
Flavonifractor	-0.07	0.42	0.62	-0.07	0.44	0.75	-0.09	0.33	0.76	-0.09	0.33	0.75	-0.09	0.33	0.77
Fournierella	0.05	0.60	0.77	0.04	0.68	0.89	0.05	0.61	0.90	0.05	0.63	0.88	0.06	0.54	0.88
Frisingicoccus	0.03	0.72	0.87	0.00	0.99	0.99	-0.07	0.39	0.76	-0.07	0.37	0.75	-0.06	0.41	0.79
Fusicatenibacter	-0.01	0.93	0.98	-0.04	0.55	0.80	-0.08	0.28	0.76	-0.08	0.30	0.75	-0.08	0.30	0.77
Fusobacterium	-0.14	0.20	0.42	-0.09	0.39	0.71	0.00	0.97	0.99	0.00	0.99	1.00	0.00	1.00	1.00
GCA.900066575	0.00	0.98	0.98	-0.08	0.41	0.71	-0.13	0.20	0.64	-0.13	0.18	0.60	-0.14	0.16	0.54
Gordonibacter	0.27	0.01	0.07	0.23	0.02	0.24	0.18	0.08	0.53	0.17	0.09	0.54	0.17	0.09	0.52
Granulicatella	0.10	0.45	0.64	0.12	0.36	0.69	0.11	0.43	0.77	0.11	0.42	0.75	0.12	0.38	0.79
Haemophilus	0.15	0.04	0.14	0.12	0.09	0.33	0.11	0.14	0.53	0.11	0.14	0.54	0.12	0.12	0.52
Harryflintia	0.05	0.68	0.83	0.02	0.83	0.94	0.00	0.98	0.99	0.00	1.00	1.00	-0.01	0.95	0.99
Holdemanella	-0.06	0.40	0.60	-0.04	0.56	0.80	-0.03	0.70	0.91	-0.03	0.72	0.93	-0.03	0.73	0.92
Holdemania	0.16	0.37	0.59	0.16	0.34	0.69	0.04	0.81	0.96	0.04	0.83	0.98	0.01	0.95	0.99

© 2022 Meyer K et al. *JAMA Network Open.*

Hungatella	-0.19	0.04	0.13	-0.16	0.06	0.27	-0.13	0.16	0.55	-0.13	0.16	0.54	-0.12	0.20	0.64
Hydrogenoanaerobacterium	0.27	0.01	0.06	0.19	0.05	0.27	0.15	0.13	0.53	0.15	0.14	0.54	0.15	0.14	0.52
Incertae_Sedis	0.13	0.41	0.60	0.14	0.32	0.67	0.05	0.76	0.93	0.05	0.77	0.94	0.05	0.75	0.94
Intestinibacter	0.13	0.14	0.33	0.18	0.04	0.24	0.15	0.09	0.53	0.15	0.08	0.54	0.16	0.07	0.52
Intestinimonas	0.24	0.00	0.05	0.17	0.03	0.24	0.15	0.06	0.53	0.15	0.07	0.54	0.16	0.05	0.47
Lachnoclostridium	-0.38	0.03	0.13	-0.24	0.15	0.42	-0.10	0.57	0.90	-0.10	0.59	0.88	-0.10	0.57	0.91
Lachnospira	0.26	0.00	0.04	0.16	0.06	0.27	0.11	0.20	0.64	0.12	0.19	0.60	0.13	0.16	0.54
Lachnospiraceae_FCS020_group	0.28	0.00	0.04	0.19	0.03	0.24	0.14	0.13	0.53	0.14	0.13	0.54	0.14	0.14	0.52
Lachnospiraceae_ND3007_group	0.10	0.09	0.23	0.05	0.36	0.69	0.02	0.69	0.91	0.03	0.67	0.90	0.03	0.64	0.92
Lachnospiraceae_NK4A136_group	0.11	0.21	0.43	0.02	0.82	0.94	-0.08	0.41	0.76	-0.08	0.41	0.75	-0.07	0.45	0.79
Lachnospiraceae_UCG.001	0.15	0.02	0.09	0.12	0.04	0.26	0.10	0.15	0.53	0.09	0.15	0.54	0.10	0.14	0.52
Lachnospiraceae_UCG.004	0.03	0.63	0.80	0.00	0.98	0.99	0.00	0.95	0.99	0.01	0.94	0.99	-0.01	0.91	0.99
Lachnospiraceae_UCG.010	-0.12	0.23	0.44	-0.19	0.05	0.27	-0.22	0.03	0.53	-0.22	0.03	0.54	-0.23	0.03	0.47
Lactobacillus	-0.26	0.00	0.04	-0.18	0.03	0.24	-0.20	0.02	0.53	-0.20	0.02	0.54	-0.20	0.02	0.47
Lactococcus	0.08	0.46	0.65	0.05	0.66	0.88	0.06	0.61	0.90	0.06	0.63	0.88	0.04	0.72	0.92
Marvinbryantia	-0.01	0.89	0.95	-0.05	0.45	0.75	-0.02	0.75	0.93	-0.02	0.74	0.94	-0.02	0.80	0.98
Megasphaera	-0.10	0.09	0.23	-0.04	0.46	0.75	0.00	0.95	0.99	0.00	0.94	0.99	0.00	0.96	1.00
Monoglobus	0.25	0.00	0.06	0.21	0.01	0.24	0.18	0.05	0.53	0.18	0.05	0.54	0.19	0.04	0.47
Moryella	0.15	0.21	0.42	0.07	0.54	0.80	-0.01	0.95	0.99	-0.01	0.95	0.99	0.00	0.99	1.00
Negativibacillus	-0.12	0.11	0.27	-0.10	0.14	0.40	-0.11	0.15	0.53	-0.11	0.15	0.54	-0.11	0.13	0.52
NK4A214_group	0.06	0.33	0.56	0.02	0.77	0.92	-0.01	0.81	0.96	-0.02	0.79	0.94	-0.01	0.87	0.99
Odoribacter	0.11	0.05	0.16	0.09	0.11	0.35	0.06	0.28	0.76	0.06	0.29	0.75	0.06	0.28	0.76
Oscillibacter	0.21	0.03	0.12	0.17	0.06	0.27	0.08	0.38	0.76	0.08	0.41	0.75	0.08	0.42	0.79
Oscillospira	0.04	0.77	0.89	0.05	0.71	0.91	0.00	1.00	1.00	0.00	0.98	1.00	0.01	0.95	0.99
Oxalobacter	0.24	0.01	0.08	0.14	0.14	0.40	0.07	0.45	0.80	0.07	0.46	0.81	0.08	0.43	0.79
Paludicola	0.12	0.23	0.44	0.03	0.75	0.92	-0.04	0.66	0.90	-0.05	0.61	0.88	-0.04	0.70	0.92
Parabacteroides	-0.07	0.34	0.56	-0.03	0.70	0.90	-0.01	0.88	0.99	-0.01	0.89	0.99	-0.01	0.90	0.99
Paraprevotella	-0.01	0.84	0.92	0.01	0.82	0.94	0.01	0.87	0.99	0.01	0.89	0.99	0.01	0.88	0.99
Parasutterella	0.15	0.00	0.06	0.13	0.01	0.24	0.07	0.20	0.64	0.07	0.19	0.60	0.07	0.22	0.70
Peptococcus	0.11	0.27	0.47	0.04	0.66	0.88	-0.05	0.65	0.90	-0.05	0.63	0.88	-0.04	0.67	0.92
Phascolarctobacterium	0.03	0.49	0.67	0.04	0.38	0.70	0.04	0.38	0.76	0.04	0.40	0.75	0.04	0.38	0.79
Phocea	-0.24	0.06	0.17	-0.27	0.02	0.24	-0.30	0.01	0.53	-0.30	0.01	0.54	-0.31	0.01	0.47
Prevotella	-0.11	0.02	0.11	-0.07	0.09	0.33	-0.06	0.22	0.69	-0.06	0.23	0.69	-0.05	0.27	0.76
Romboutsia	0.02	0.79	0.90	0.05	0.52	0.78	0.01	0.90	0.99	0.01	0.88	0.99	0.01	0.88	0.99
Roseburia	0.02	0.84	0.92	-0.01	0.92	0.98	-0.06	0.55	0.89	-0.06	0.56	0.88	-0.05	0.62	0.92
Ruminococcus	0.13	0.03	0.12	0.08	0.16	0.43	0.05	0.40	0.76	0.05	0.41	0.75	0.06	0.36	0.77
Sellimonas	0.16	0.06	0.17	0.13	0.11	0.35	0.14	0.11	0.53	0.13	0.12	0.54	0.14	0.12	0.52
Senegalimassilia	-0.13	0.11	0.27	-0.12	0.11	0.35	-0.13	0.11	0.53	-0.13	0.11	0.54	-0.13	0.12	0.52
Shuttleworthia	0.29	0.00	0.04	0.19	0.03	0.24	0.13	0.14	0.53	0.13	0.15	0.54	0.14	0.13	0.52
Slackia	-0.15	0.04	0.13	-0.13	0.06	0.27	-0.14	0.06	0.53	-0.14	0.06	0.54	-0.14	0.05	0.47
Streptococcus	0.14	0.19	0.42	0.13	0.19	0.47	0.18	0.09	0.53	0.18	0.09	0.54	0.19	0.08	0.52
Subdoligranulum	0.16	0.04	0.14	0.12	0.11	0.35	0.08	0.32	0.76	0.08	0.33	0.75	0.08	0.31	0.77
Sutterella	-0.12	0.01	0.08	-0.08	0.08	0.33	-0.05	0.32	0.76	-0.05	0.32	0.75	-0.05	0.32	0.77
TM7x	-0.01	0.96	0.98	-0.07	0.52	0.78	-0.05	0.62	0.90	-0.05	0.62	0.88	-0.04	0.71	0.92
Turicibacter	0.28	0.01	0.08	0.34	0.00	0.14	0.34	0.00	0.19	0.34	0.00	0.18	0.33	0.00	0.28
Tuzzerella	-0.12	0.10	0.25	-0.10	0.16	0.43	-0.14	0.06	0.53	-0.14	0.06	0.54	-0.15	0.05	0.47
UBA1819	0.00	0.98	0.98	-0.02	0.86	0.94	-0.10	0.39	0.76	-0.11	0.35	0.75	-0.11	0.35	0.77
UCG.002	0.12	0.03	0.13	0.07	0.17	0.43	0.04	0.49	0.85	0.04	0.51	0.86	0.04	0.49	0.81
UCG.003	0.03	0.59	0.76	-0.02	0.72	0.91	-0.06	0.34	0.76	-0.06	0.34	0.75	-0.07	0.31	0.77
UCG.005	0.07	0.33	0.56	0.02	0.78	0.92	-0.03	0.66	0.90	-0.04	0.62	0.88	-0.03	0.68	0.92
UCG.009	0.12	0.27	0.47	0.02	0.82	0.94	-0.03	0.80	0.96	-0.03	0.79	0.94	-0.02	0.84	0.99
Veillonella	0.13	0.09	0.24	0.12	0.09	0.33	0.11	0.15	0.53	0.11	0.14	0.54	0.12	0.13	0.52
Victivallis	0.11	0.15	0.34	0.06	0.40	0.71	0.01	0.92	0.99	0.01	0.94	0.99	0.01	0.88	0.99

Multivariable-adjusted regression models adjusted for:
Model 1: sequencing batch, age, race, gender, study center
Model 2: Model 1, with education
Model 3: Model 2, with physical activity, diet score, medication use, and smoking
Model 4: Model 3 with BMI

© 2022 Meyer K et al. *JAMA Network Open.*

Model 5: Model 4 with hypertension and diabetes

© 2022 Meyer K et al. *JAMA Network Open.*

eTable 15: Multivariable-adjusted regression coefficients, p-values, and false discovery rate (FDR)-adjusted p-values for genera with respect to category fluency.

	Model1			Model2			Model3			Model4			Model5		
	beta	p-value	FDR	beta	p-value	FDR	beta	p-value	FDR	beta	p-value	FDR	beta	p-value	FDR
Acetanaerobacterium	-0.16	0.71	0.84	0.01	0.97	0.99	0.00	1.00	1.00	-0.02	0.96	0.97	-0.06	0.90	0.97
Acidaminococcus	-0.31	0.03	0.25	-0.19	0.16	0.60	-0.18	0.23	0.92	-0.17	0.26	0.92	-0.17	0.25	0.91
Actinomyces	-0.39	0.30	0.59	-0.30	0.42	0.82	-0.19	0.63	0.92	-0.20	0.62	0.92	-0.16	0.68	0.91
Adlercreutzia	-0.01	0.97	0.99	-0.05	0.83	0.92	-0.11	0.65	0.93	-0.12	0.62	0.92	-0.14	0.56	0.91
Agathobacter	0.15	0.61	0.83	0.11	0.68	0.90	0.02	0.95	0.98	0.06	0.84	0.96	0.10	0.73	0.93
Akkermansia	0.05	0.73	0.85	-0.04	0.79	0.92	-0.08	0.62	0.92	-0.08	0.62	0.92	-0.09	0.54	0.91
Alistipes	0.08	0.76	0.86	-0.01	0.96	0.99	-0.17	0.50	0.92	-0.17	0.50	0.92	-0.19	0.44	0.91
Allisonella	0.05	0.87	0.92	0.38	0.18	0.60	0.38	0.22	0.92	0.39	0.20	0.89	0.42	0.18	0.89
Anaerofilum	0.37	0.26	0.53	0.09	0.78	0.92	0.06	0.87	0.94	0.03	0.92	0.97	0.01	0.97	0.99
Anaerostipes	-0.08	0.81	0.90	-0.21	0.54	0.85	-0.25	0.48	0.92	-0.25	0.48	0.92	-0.25	0.49	0.91
Anaerotruncus	0.01	0.97	0.99	-0.08	0.79	0.92	-0.11	0.73	0.94	-0.16	0.61	0.92	-0.17	0.59	0.91
Bacteroides	-0.14	0.78	0.87	-0.26	0.61	0.86	-0.27	0.61	0.92	-0.22	0.67	0.93	-0.21	0.69	0.91
Barnesiella	0.54	0.00	0.01	0.54	0.00	0.01	0.58	0.00	0.01	0.57	0.00	0.01	0.59	0.00	0.00
Bifidobacterium	-0.08	0.66	0.83	0.06	0.75	0.91	0.04	0.82	0.94	0.05	0.78	0.95	0.05	0.80	0.93
Bilophila	0.57	0.00	0.06	0.50	0.01	0.20	0.51	0.01	0.44	0.52	0.01	0.40	0.50	0.01	0.51
Blautia	-0.48	0.46	0.75	-0.70	0.27	0.70	-0.62	0.34	0.92	-0.65	0.32	0.92	-0.63	0.35	0.91
Butyricicoccus	0.55	0.22	0.51	0.19	0.66	0.90	0.12	0.79	0.94	0.14	0.77	0.95	0.12	0.79	0.93
Butyricimonas	0.36	0.03	0.25	0.29	0.06	0.50	0.32	0.05	0.64	0.32	0.05	0.54	0.33	0.05	0.56
CAG.56	0.23	0.21	0.50	0.15	0.41	0.82	0.10	0.62	0.92	0.10	0.61	0.92	0.10	0.60	0.91
Candidatus_Soleaferrea	-0.02	0.95	0.99	-0.14	0.70	0.90	-0.10	0.79	0.94	-0.11	0.77	0.95	-0.15	0.71	0.91
Caproiciproducens	-0.49	0.12	0.41	-0.48	0.12	0.57	-0.49	0.14	0.92	-0.50	0.13	0.83	-0.55	0.10	0.76
Christensenellaceae_R.7_group	0.33	0.03	0.27	0.23	0.13	0.59	0.18	0.28	0.92	0.16	0.33	0.92	0.15	0.36	0.91
Clostridium_sensu_stricto_1	0.08	0.70	0.84	0.11	0.56	0.85	0.11	0.60	0.92	0.11	0.61	0.92	0.10	0.64	0.91
Colidextribacter	-0.06	0.88	0.92	-0.19	0.61	0.86	-0.21	0.60	0.92	-0.23	0.57	0.92	-0.19	0.65	0.91
Collinsella	0.25	0.15	0.41	0.43	0.01	0.25	0.43	0.02	0.44	0.44	0.01	0.44	0.43	0.02	0.51
Coprobacter	0.32	0.09	0.38	0.24	0.20	0.63	0.14	0.47	0.92	0.13	0.50	0.92	0.12	0.55	0.91
Coprococcus	0.13	0.52	0.78	0.12	0.57	0.85	0.09	0.67	0.93	0.09	0.67	0.93	0.11	0.60	0.91
Defluviitaleaceae_UCG.011	0.43	0.15	0.41	0.33	0.26	0.70	0.38	0.22	0.92	0.35	0.26	0.92	0.31	0.32	0.91
Desulfovibrio	-0.15	0.37	0.68	-0.13	0.43	0.82	-0.18	0.30	0.92	-0.21	0.24	0.92	-0.23	0.21	0.91
Dialister	-0.06	0.65	0.83	-0.05	0.69	0.90	-0.03	0.81	0.94	-0.03	0.84	0.96	-0.01	0.93	0.98
DNF00809	-0.21	0.52	0.78	-0.29	0.38	0.81	-0.27	0.43	0.92	-0.30	0.39	0.92	-0.30	0.38	0.91
Dorea	-0.05	0.84	0.91	-0.01	0.98	0.99	-0.04	0.87	0.94	-0.03	0.91	0.97	-0.05	0.86	0.96
DTU089	-0.40	0.14	0.41	-0.46	0.09	0.51	-0.49	0.08	0.64	-0.51	0.07	0.62	-0.47	0.10	0.76
Eggerthella	-0.37	0.15	0.41	-0.42	0.10	0.52	-0.36	0.18	0.92	-0.38	0.17	0.83	-0.36	0.19	0.89
Eisenbergiella	-0.31	0.25	0.53	-0.31	0.24	0.67	-0.34	0.21	0.92	-0.39	0.16	0.83	-0.37	0.18	0.89
Erysipelatoclostridium	-0.45	0.11	0.41	-0.43	0.12	0.57	-0.38	0.19	0.92	-0.42	0.15	0.83	-0.41	0.16	0.89
Erysipelotrichaceae_UCG.003	0.16	0.43	0.74	0.13	0.51	0.85	0.12	0.56	0.92	0.12	0.57	0.92	0.10	0.64	0.91
Escherichia.Shigella	-0.26	0.16	0.43	-0.16	0.38	0.81	-0.10	0.63	0.92	-0.09	0.66	0.93	-0.09	0.67	0.91
Faecalibacterium	0.18	0.51	0.78	0.07	0.79	0.92	0.04	0.88	0.94	0.05	0.85	0.96	0.05	0.85	0.96
Family_XIII_AD3011_group	0.21	0.40	0.71	0.03	0.89	0.95	-0.05	0.84	0.94	-0.09	0.73	0.95	-0.13	0.63	0.91
Family_XIII_UCG.001	0.53	0.09	0.38	0.41	0.17	0.60	0.34	0.30	0.92	0.30	0.36	0.92	0.27	0.41	0.91
FD2005	-0.22	0.46	0.75	-0.13	0.65	0.90	-0.11	0.72	0.94	-0.10	0.74	0.95	-0.09	0.77	0.93
Flavonifractor	-0.39	0.14	0.41	-0.37	0.15	0.60	-0.32	0.25	0.92	-0.31	0.25	0.92	-0.30	0.29	0.91
Fournierella	-0.12	0.67	0.83	-0.16	0.57	0.85	-0.21	0.47	0.92	-0.24	0.42	0.92	-0.23	0.45	0.91
Frisingicoccus	0.14	0.52	0.78	0.08	0.71	0.90	0.02	0.93	0.97	0.00	0.99	0.99	0.00	0.99	0.99
Fusicatenibacter	0.37	0.08	0.38	0.28	0.17	0.60	0.20	0.36	0.92	0.23	0.30	0.92	0.19	0.41	0.91
Fusobacterium	-0.18	0.58	0.81	-0.05	0.86	0.93	0.11	0.74	0.94	0.08	0.80	0.96	0.10	0.77	0.93
GCA.900066575	0.33	0.24	0.53	0.15	0.60	0.86	0.13	0.66	0.93	0.09	0.75	0.95	0.06	0.84	0.95
Gordonibacter	0.21	0.48	0.75	0.11	0.70	0.90	0.20	0.50	0.92	0.16	0.59	0.92	0.14	0.64	0.91
Granulicatella	-0.06	0.88	0.92	-0.02	0.95	0.99	0.02	0.96	0.98	0.04	0.93	0.97	0.11	0.78	0.93
Haemophilus	0.37	0.08	0.38	0.29	0.15	0.60	0.17	0.43	0.92	0.17	0.44	0.92	0.19	0.39	0.91
Harryflintia	-0.14	0.66	0.83	-0.19	0.53	0.85	-0.28	0.41	0.92	-0.30	0.37	0.92	-0.33	0.33	0.91
Holdemanella	0.07	0.74	0.85	0.12	0.55	0.85	0.18	0.41	0.92	0.19	0.39	0.92	0.18	0.41	0.91
Holdemania	0.17	0.74	0.85	0.17	0.72	0.91	0.13	0.79	0.94	0.09	0.86	0.96	0.01	0.99	0.99

© 2022 Meyer K et al. *JAMA Network Open.*

Hungatella	-0.74	0.00	0.10	-0.67	0.01	0.21	-0.61	0.02	0.47	-0.62	0.02	0.44	-0.58	0.03	0.56
Hydrogenoanaerobacterium	0.28	0.32	0.61	0.06	0.82	0.92	-0.05	0.87	0.94	-0.09	0.77	0.95	-0.12	0.68	0.91
Incertae_Sedis	-0.13	0.76	0.86	-0.09	0.84	0.92	-0.03	0.95	0.98	-0.04	0.93	0.97	-0.06	0.90	0.97
Intestinibacter	-0.11	0.67	0.83	0.00	0.99	0.99	-0.05	0.86	0.94	-0.01	0.96	0.97	-0.01	0.98	0.99
Intestinimonas	0.39	0.09	0.38	0.23	0.32	0.72	0.14	0.57	0.92	0.11	0.65	0.93	0.13	0.60	0.91
Lachnoclostridium	-0.89	0.08	0.38	-0.57	0.24	0.67	-0.27	0.60	0.92	-0.23	0.66	0.93	-0.20	0.71	0.91
Lachnospira	0.79	0.00	0.04	0.55	0.02	0.36	0.36	0.17	0.92	0.37	0.15	0.83	0.39	0.14	0.89
Lachnospiraceae_FCS020_grou	0.68	0.01	0.15	0.45	0.07	0.50	0.48	0.08	0.64	0.48	0.08	0.62	0.45	0.10	0.76
Lachnospiraceae_ND3007_gro	0.43	0.01	0.17	0.31	0.07	0.50	0.25	0.17	0.92	0.26	0.14	0.83	0.26	0.15	0.89
Lachnospiraceae_NK4A136_gr	0.39	0.11	0.41	0.18	0.45	0.82	-0.04	0.87	0.94	-0.04	0.87	0.96	-0.05	0.87	0.96
Lachnospiraceae_UCG.001	0.30	0.10	0.38	0.24	0.19	0.60	0.14	0.46	0.92	0.13	0.51	0.92	0.12	0.53	0.91
Lachnospiraceae_UCG.004	0.19	0.34	0.66	0.11	0.56	0.85	0.03	0.89	0.94	0.04	0.84	0.96	0.03	0.90	0.97
Lachnospiraceae_UCG.010	-0.39	0.18	0.45	-0.56	0.05	0.49	-0.56	0.06	0.64	-0.59	0.05	0.54	-0.58	0.05	0.56
Lactobacillus	-0.34	0.15	0.41	-0.14	0.55	0.85	-0.16	0.53	0.92	-0.13	0.60	0.92	-0.11	0.68	0.91
Lactococcus	0.56	0.08	0.38	0.48	0.13	0.59	0.49	0.15	0.92	0.47	0.16	0.83	0.45	0.18	0.89
Marvinbryantia	-0.03	0.87	0.92	-0.13	0.49	0.85	-0.10	0.61	0.92	-0.11	0.59	0.92	-0.08	0.69	0.91
Megasphaera	-0.20	0.24	0.53	-0.05	0.74	0.91	0.05	0.79	0.94	0.05	0.77	0.95	0.05	0.80	0.93
Monoglobus	0.55	0.03	0.25	0.45	0.06	0.50	0.36	0.18	0.92	0.35	0.19	0.89	0.34	0.21	0.91
Moryella	0.60	0.07	0.38	0.41	0.20	0.63	0.30	0.40	0.92	0.30	0.40	0.92	0.30	0.40	0.91
Negativibacillus	-0.10	0.64	0.83	-0.07	0.74	0.91	-0.08	0.70	0.94	-0.09	0.69	0.93	-0.11	0.62	0.91
NK4A214_group	0.22	0.19	0.45	0.12	0.44	0.82	0.03	0.88	0.94	0.01	0.96	0.97	0.01	0.97	0.99
Odoribacter	0.33	0.04	0.29	0.27	0.08	0.50	0.18	0.27	0.92	0.18	0.28	0.92	0.16	0.34	0.91
Oscillibacter	0.25	0.37	0.68	0.15	0.57	0.85	0.22	0.44	0.92	0.19	0.51	0.92	0.16	0.58	0.91
Oscillospira	-0.31	0.41	0.73	-0.29	0.43	0.82	-0.23	0.55	0.92	-0.25	0.53	0.92	-0.22	0.58	0.91
Oxalobacter	0.53	0.05	0.35	0.28	0.29	0.72	0.23	0.42	0.92	0.23	0.43	0.92	0.20	0.49	0.91
Paludicola	0.00	0.99	0.99	-0.22	0.42	0.82	-0.34	0.24	0.92	-0.41	0.17	0.83	-0.43	0.15	0.89
Parabacteroides	0.29	0.14	0.41	0.39	0.04	0.49	0.39	0.05	0.64	0.40	0.05	0.54	0.41	0.04	0.56
Paraprevotella	-0.07	0.65	0.83	-0.01	0.92	0.97	-0.03	0.85	0.94	-0.04	0.80	0.96	-0.03	0.84	0.95
Parasutterella	0.38	0.01	0.16	0.35	0.02	0.31	0.29	0.07	0.64	0.30	0.06	0.59	0.26	0.10	0.76
Peptococcus	0.17	0.57	0.81	0.00	0.99	0.99	-0.13	0.66	0.93	-0.16	0.61	0.92	-0.16	0.61	0.91
Phascolarctobacterium	0.22	0.13	0.41	0.23	0.09	0.51	0.26	0.08	0.64	0.24	0.10	0.75	0.24	0.11	0.80
Phocea	-0.61	0.09	0.38	-0.69	0.05	0.49	-0.77	0.03	0.52	-0.75	0.04	0.54	-0.72	0.05	0.56
Prevotella	0.05	0.69	0.84	0.13	0.30	0.72	0.12	0.36	0.92	0.13	0.35	0.92	0.13	0.35	0.91
Romboutsia	0.25	0.29	0.58	0.32	0.16	0.60	0.25	0.31	0.92	0.26	0.28	0.92	0.27	0.28	0.91
Roseburia	0.38	0.15	0.41	0.32	0.22	0.65	0.17	0.54	0.92	0.19	0.50	0.92	0.23	0.41	0.91
Ruminococcus	0.42	0.02	0.19	0.30	0.08	0.50	0.20	0.26	0.92	0.20	0.28	0.92	0.18	0.33	0.91
Sellimonas	0.34	0.17	0.43	0.26	0.28	0.71	0.23	0.37	0.92	0.20	0.43	0.92	0.20	0.43	0.91
Senegalimassilia	0.27	0.24	0.53	0.28	0.21	0.63	0.29	0.22	0.92	0.29	0.22	0.92	0.29	0.23	0.91
Shuttleworthia	0.16	0.54	0.79	-0.11	0.67	0.90	-0.18	0.50	0.92	-0.20	0.45	0.92	-0.18	0.51	0.91
Slackia	-0.10	0.64	0.83	-0.05	0.80	0.92	-0.03	0.89	0.94	-0.03	0.90	0.97	-0.07	0.75	0.93
Streptococcus	0.23	0.45	0.75	0.22	0.46	0.83	0.31	0.33	0.92	0.30	0.35	0.92	0.32	0.32	0.91
Subdoligranulum	0.79	0.00	0.02	0.69	0.00	0.07	0.56	0.02	0.44	0.55	0.02	0.44	0.57	0.01	0.51
Sutterella	-0.07	0.59	0.82	0.03	0.84	0.92	0.02	0.86	0.94	0.02	0.87	0.96	0.04	0.78	0.93
TM7x	-0.15	0.62	0.83	-0.30	0.31	0.72	-0.21	0.51	0.92	-0.21	0.50	0.92	-0.20	0.54	0.91
Turicibacter	0.52	0.10	0.38	0.66	0.03	0.40	0.72	0.03	0.47	0.73	0.02	0.45	0.70	0.03	0.56
Tuzzerella	0.15	0.48	0.75	0.21	0.32	0.72	0.15	0.50	0.92	0.14	0.53	0.92	0.14	0.55	0.91
UBA1819	-0.24	0.46	0.75	-0.28	0.37	0.81	-0.23	0.49	0.92	-0.29	0.40	0.92	-0.30	0.40	0.91
UCG.002	0.14	0.36	0.68	0.03	0.84	0.92	-0.05	0.78	0.94	-0.07	0.70	0.93	-0.07	0.69	0.91
UCG.003	0.31	0.08	0.38	0.18	0.30	0.72	0.11	0.56	0.92	0.11	0.57	0.92	0.08	0.66	0.91
UCG.005	0.38	0.06	0.38	0.26	0.17	0.60	0.16	0.44	0.92	0.14	0.51	0.92	0.12	0.59	0.91
UCG.009	0.00	0.99	0.99	-0.23	0.45	0.82	-0.34	0.28	0.92	-0.35	0.27	0.92	-0.36	0.25	0.91
Veillonella	0.12	0.57	0.81	0.11	0.59	0.86	0.08	0.73	0.94	0.09	0.69	0.93	0.12	0.61	0.91
Victivallis	0.25	0.26	0.53	0.13	0.53	0.85	0.00	1.00	1.00	-0.02	0.94	0.97	-0.01	0.98	0.99

Multivariable-adjusted regression models adjusted for:
Model 1: sequencing batch, age, race, gender, study center
Model 2: Model 1, with education
Model 3: Model 2, with physical activity, diet score, medication use, and smoking
Model 4: Model 3 with BMI

© 2022 Meyer K et al. *JAMA Network Open.*

Model 5: Model 4 with hypertension and diabetes

© 2022 Meyer K et al. *JAMA Network Open.*

eTable 16: Multivariable-adjusted regression coefficients, p-values, and false discovery rate (FDR)-adjusted p-values for genera with respect to letter fluency.

	Model1			Model2			Model3			Model4			Model5		
	beta	p-value	FDR	beta	p-value	FDR	beta	p-value	FDR	beta	p-value	FDR	beta	p-value	FDR
Acetanaerobacterium	0.41	0.70	0.98	0.79	0.45	0.96	0.86	0.43	0.98	0.84	0.44	0.98	0.72	0.51	0.98
Acidaminococcus	-0.70	0.04	0.53	-0.44	0.19	0.84	-0.30	0.41	0.98	-0.29	0.43	0.98	-0.29	0.43	0.98
Actinomyces	-0.09	0.92	0.98	0.12	0.90	0.96	0.66	0.50	0.98	0.66	0.50	0.98	0.77	0.43	0.98
Adlercreutzia	-0.72	0.20	0.68	-0.82	0.14	0.84	-0.89	0.12	0.98	-0.91	0.12	0.98	-0.95	0.10	0.98
Agathobacter	0.16	0.83	0.98	0.10	0.89	0.96	-0.24	0.74	0.98	-0.19	0.80	0.98	-0.11	0.88	0.98
Akkermansia	0.06	0.87	0.98	-0.14	0.69	0.96	-0.24	0.53	0.98	-0.24	0.53	0.98	-0.29	0.45	0.98
Alistipes	0.81	0.18	0.68	0.61	0.30	0.96	0.19	0.76	0.98	0.19	0.76	0.98	0.13	0.83	0.98
Allisonella	0.09	0.90	0.98	0.84	0.23	0.86	1.02	0.17	0.98	1.04	0.16	0.98	1.10	0.14	0.98
Anaerofilum	0.74	0.35	0.79	0.11	0.89	0.96	0.03	0.97	0.98	0.00	1.00	1.00	-0.07	0.93	0.98
Anaerostipes	-0.52	0.55	0.94	-0.80	0.34	0.96	-0.49	0.58	0.98	-0.49	0.57	0.98	-0.40	0.65	0.98
Anaerotruncus	-0.18	0.80	0.98	-0.40	0.58	0.96	-0.37	0.62	0.98	-0.44	0.56	0.98	-0.48	0.53	0.98
Bacteroides	0.16	0.90	0.98	-0.12	0.93	0.96	-0.18	0.89	0.98	-0.12	0.93	0.98	-0.14	0.91	0.98
Barnesiella	1.03	0.00	0.27	1.04	0.00	0.18	0.91	0.01	0.96	0.91	0.01	0.98	0.92	0.01	0.88
Bifidobacterium	-0.08	0.85	0.98	0.25	0.57	0.96	0.16	0.71	0.98	0.18	0.69	0.98	0.17	0.71	0.98
Bilophila	1.04	0.03	0.53	0.87	0.06	0.84	0.84	0.07	0.98	0.86	0.07	0.98	0.78	0.10	0.98
Blautia	-1.97	0.22	0.68	-2.50	0.11	0.84	-2.21	0.17	0.98	-2.25	0.16	0.98	-2.01	0.22	0.98
Butyricicoccus	0.20	0.85	0.98	-0.64	0.55	0.96	-1.16	0.30	0.98	-1.15	0.30	0.98	-1.10	0.32	0.98
Butyricimonas	0.50	0.21	0.68	0.36	0.35	0.96	0.33	0.41	0.98	0.34	0.40	0.98	0.32	0.42	0.98
CAG.56	0.39	0.39	0.83	0.22	0.62	0.96	0.27	0.56	0.98	0.28	0.56	0.98	0.28	0.55	0.98
Candidatus_Soleaferrea	-0.26	0.78	0.98	-0.56	0.54	0.96	-0.35	0.71	0.98	-0.35	0.70	0.98	-0.42	0.65	0.98
Caproiciproducens	0.49	0.53	0.94	0.51	0.51	0.96	0.09	0.92	0.98	0.07	0.93	0.98	-0.01	0.99	1.00
Christensenellaceae_R.7_group	0.55	0.15	0.68	0.32	0.39	0.96	0.06	0.89	0.98	0.03	0.94	0.98	0.01	0.98	1.00
Clostridium_sensu_stricto_1	0.21	0.66	0.98	0.31	0.52	0.96	0.33	0.51	0.98	0.32	0.51	0.98	0.24	0.64	0.98
Colidextribacter	0.32	0.74	0.98	0.00	1.00	1.00	-0.41	0.68	0.98	-0.43	0.66	0.98	-0.34	0.73	0.98
Collinsella	-0.24	0.58	0.94	0.16	0.70	0.96	0.06	0.90	0.98	0.07	0.88	0.98	0.02	0.96	0.99
Coprobacter	0.90	0.06	0.53	0.69	0.13	0.84	0.37	0.45	0.98	0.36	0.46	0.98	0.36	0.46	0.98
Coprococcus	0.78	0.13	0.68	0.74	0.14	0.84	0.71	0.17	0.98	0.71	0.17	0.98	0.73	0.16	0.98
Defluviitaleaceae_UCG.011	0.36	0.63	0.97	0.10	0.89	0.96	0.08	0.91	0.98	0.03	0.96	0.98	-0.08	0.92	0.98
Desulfovibrio	-0.18	0.67	0.98	-0.12	0.77	0.96	-0.20	0.65	0.98	-0.23	0.60	0.98	-0.23	0.61	0.98
Dialister	-0.36	0.28	0.73	-0.34	0.29	0.96	-0.13	0.71	0.98	-0.12	0.73	0.98	-0.11	0.75	0.98
DNF00809	-0.62	0.46	0.87	-0.80	0.32	0.96	-0.89	0.29	0.98	-0.92	0.28	0.98	-0.96	0.26	0.98
Dorea	-0.18	0.78	0.98	-0.07	0.92	0.96	0.02	0.98	0.98	0.03	0.96	0.98	0.00	1.00	1.00
DTU089	0.35	0.61	0.96	0.23	0.73	0.96	0.03	0.97	0.98	0.00	1.00	1.00	0.12	0.87	0.98
Eggerthella	-0.90	0.16	0.68	-1.04	0.10	0.84	-0.69	0.30	0.98	-0.71	0.28	0.98	-0.62	0.35	0.98
Eisenbergiella	-0.94	0.16	0.68	-0.94	0.15	0.84	-1.05	0.12	0.98	-1.13	0.10	0.98	-1.00	0.14	0.98
Erysipelatoclostridium	-0.42	0.55	0.94	-0.40	0.56	0.96	-0.47	0.50	0.98	-0.52	0.47	0.98	-0.50	0.49	0.98
Erysipelotrichaceae_UCG.003	0.40	0.43	0.87	0.33	0.50	0.96	0.11	0.83	0.98	0.10	0.85	0.98	0.04	0.94	0.98
Escherichia.Shigella	-0.64	0.17	0.68	-0.40	0.38	0.96	-0.31	0.52	0.98	-0.30	0.53	0.98	-0.31	0.53	0.98
Faecalibacterium	0.54	0.42	0.87	0.31	0.64	0.96	0.11	0.88	0.98	0.12	0.86	0.98	0.10	0.89	0.98
Family_XIII_AD3011_group	0.80	0.20	0.68	0.41	0.50	0.96	0.18	0.78	0.98	0.13	0.84	0.98	0.09	0.89	0.98
Family_XIII_UCG.001	0.91	0.24	0.68	0.64	0.39	0.96	0.48	0.54	0.98	0.42	0.59	0.98	0.40	0.62	0.98
FD2005	-0.43	0.56	0.94	-0.24	0.74	0.96	-0.14	0.86	0.98	-0.13	0.87	0.98	-0.17	0.82	0.98
Flavonifractor	-0.83	0.20	0.68	-0.80	0.20	0.84	-0.74	0.27	0.98	-0.73	0.27	0.98	-0.71	0.29	0.98
Fournierella	0.11	0.87	0.98	0.05	0.95	0.96	-0.27	0.71	0.98	-0.31	0.67	0.98	-0.29	0.69	0.98
Frisingicoccus	0.07	0.89	0.98	-0.10	0.85	0.96	-0.32	0.56	0.98	-0.34	0.53	0.98	-0.30	0.59	0.98
Fusicatenibacter	0.28	0.59	0.94	0.08	0.88	0.96	-0.13	0.81	0.98	-0.09	0.87	0.98	-0.18	0.75	0.98
Fusobacterium	0.78	0.33	0.76	1.02	0.19	0.84	1.30	0.11	0.98	1.27	0.11	0.98	1.27	0.12	0.98
GCA.900066575	1.07	0.12	0.68	0.66	0.33	0.96	0.39	0.59	0.98	0.34	0.64	0.98	0.21	0.77	0.98
Gordonibacter	0.57	0.43	0.87	0.32	0.64	0.96	0.25	0.73	0.98	0.20	0.79	0.98	0.18	0.81	0.98
Granulicatella	0.07	0.94	0.98	0.14	0.88	0.96	0.60	0.53	0.98	0.63	0.52	0.98	0.77	0.43	0.98
Haemophilus	0.46	0.37	0.81	0.30	0.55	0.96	0.21	0.70	0.98	0.20	0.70	0.98	0.24	0.66	0.98
Harryflintia	-0.86	0.28	0.73	-0.98	0.20	0.84	-1.12	0.17	0.98	-1.16	0.16	0.98	-1.34	0.10	0.98
Holdemanella	0.13	0.80	0.98	0.26	0.61	0.96	0.62	0.24	0.98	0.64	0.23	0.98	0.68	0.20	0.98

© 2022 Meyer K et al. *JAMA Network Open.*

Holdemania	0.10	0.94	0.98	0.07	0.95	0.96	0.23	0.86	0.98	0.17	0.89	0.98	-0.16	0.90	0.98
Hungatella	-1.34	0.04	0.53	-1.20	0.06	0.84	-1.17	0.07	0.98	-1.18	0.07	0.98	-1.05	0.11	0.98
Hydrogenoanaerobacterium	1.48	0.03	0.53	1.00	0.14	0.84	0.83	0.25	0.98	0.78	0.28	0.98	0.75	0.31	0.98
Incertae_Sedis	0.49	0.65	0.98	0.61	0.56	0.96	0.71	0.52	0.98	0.70	0.53	0.98	0.72	0.51	0.98
Intestinibacter	0.02	0.98	0.99	0.27	0.65	0.96	0.17	0.78	0.98	0.22	0.73	0.98	0.27	0.67	0.98
Intestinimonas	0.45	0.44	0.87	0.06	0.91	0.96	-0.03	0.96	0.98	-0.07	0.90	0.98	0.04	0.95	0.98
Lachnoclostridium	-2.04	0.10	0.63	-1.34	0.27	0.93	-0.34	0.79	0.98	-0.29	0.82	0.98	-0.18	0.89	0.98
Lachnospira	1.17	0.05	0.53	0.60	0.32	0.96	0.37	0.56	0.98	0.39	0.53	0.98	0.42	0.51	0.98
Lachnospiraceae_FCS020_group	1.59	0.01	0.53	1.10	0.08	0.84	1.06	0.11	0.98	1.06	0.11	0.98	1.02	0.13	0.98
Lachnospiraceae_ND3007_group	0.78	0.06	0.53	0.52	0.21	0.84	0.43	0.32	0.98	0.45	0.30	0.98	0.42	0.34	0.98
Lachnospiraceae_NK4A136_group	0.44	0.47	0.88	-0.04	0.95	0.96	-0.56	0.39	0.98	-0.56	0.39	0.98	-0.56	0.39	0.98
Lachnospiraceae_UCG.001	-0.01	0.98	0.99	-0.17	0.71	0.96	-0.28	0.54	0.98	-0.31	0.52	0.98	-0.33	0.49	0.98
Lachnospiraceae_UCG.004	0.38	0.46	0.87	0.21	0.67	0.96	0.27	0.60	0.98	0.28	0.58	0.98	0.14	0.79	0.98
Lachnospiraceae_UCG.010	-0.09	0.90	0.98	-0.45	0.52	0.96	-0.50	0.49	0.98	-0.54	0.46	0.98	-0.55	0.45	0.98
Lactobacillus	-0.70	0.23	0.68	-0.23	0.69	0.96	-0.31	0.61	0.98	-0.28	0.65	0.98	-0.26	0.68	0.98
Lactococcus	0.82	0.30	0.76	0.63	0.41	0.96	0.86	0.29	0.98	0.84	0.30	0.98	0.72	0.38	0.98
Marvinbryantia	0.03	0.94	0.98	-0.19	0.68	0.96	-0.28	0.56	0.98	-0.29	0.55	0.98	-0.25	0.62	0.98
Megasphaera	-0.23	0.58	0.94	0.11	0.80	0.96	0.49	0.26	0.98	0.50	0.26	0.98	0.47	0.29	0.98
Monoglobus	1.04	0.09	0.63	0.81	0.18	0.84	0.74	0.25	0.98	0.73	0.26	0.98	0.71	0.28	0.98
Moryella	0.83	0.31	0.76	0.43	0.59	0.96	0.08	0.93	0.98	0.08	0.93	0.98	0.06	0.94	0.98
Negativibacillus	0.05	0.92	0.98	0.11	0.83	0.96	-0.09	0.86	0.98	-0.10	0.86	0.98	-0.18	0.74	0.98
NK4A214_group	0.12	0.76	0.98	-0.08	0.84	0.96	-0.34	0.42	0.98	-0.36	0.39	0.98	-0.37	0.38	0.98
Odoribacter	0.95	0.01	0.53	0.85	0.03	0.78	0.50	0.21	0.98	0.50	0.22	0.98	0.45	0.27	0.98
Oscillibacter	0.17	0.80	0.98	-0.05	0.94	0.96	-0.01	0.98	0.98	-0.06	0.93	0.98	-0.13	0.85	0.98
Oscillospira	1.14	0.23	0.68	1.15	0.21	0.84	1.05	0.26	0.98	1.04	0.27	0.98	1.13	0.24	0.98
Oxalobacter	0.92	0.17	0.68	0.34	0.61	0.96	0.06	0.94	0.98	0.05	0.95	0.98	0.09	0.89	0.98
Paludicola	0.89	0.20	0.68	0.40	0.56	0.96	0.11	0.88	0.98	0.04	0.96	0.98	0.17	0.82	0.98
Parabacteroides	0.70	0.15	0.68	0.93	0.05	0.84	0.77	0.12	0.98	0.78	0.11	0.98	0.79	0.11	0.98
Paraprevotella	0.06	0.86	0.98	0.19	0.59	0.96	0.10	0.78	0.98	0.09	0.81	0.98	0.09	0.80	0.98
Parasutterella	0.67	0.07	0.53	0.59	0.10	0.84	0.38	0.32	0.98	0.39	0.31	0.98	0.30	0.44	0.98
Peptococcus	0.17	0.81	0.98	-0.21	0.77	0.96	-0.73	0.32	0.98	-0.77	0.30	0.98	-0.71	0.34	0.98
Phascolarctobacterium	0.78	0.03	0.53	0.83	0.02	0.78	0.81	0.02	0.98	0.79	0.03	0.98	0.82	0.02	0.98
Phocea	-0.02	0.98	0.99	-0.21	0.81	0.96	-0.25	0.78	0.98	-0.22	0.80	0.98	-0.25	0.78	0.98
Prevotella	-0.23	0.48	0.88	-0.05	0.89	0.96	-0.14	0.66	0.98	-0.14	0.66	0.98	-0.13	0.69	0.98
Romboutsia	0.12	0.83	0.98	0.30	0.59	0.96	0.23	0.69	0.98	0.25	0.66	0.98	0.21	0.73	0.98
Roseburia	0.46	0.49	0.89	0.29	0.65	0.96	0.04	0.96	0.98	0.05	0.94	0.98	0.11	0.87	0.98
Ruminococcus	0.13	0.76	0.98	-0.15	0.71	0.96	-0.35	0.43	0.98	-0.37	0.41	0.98	-0.40	0.38	0.98
Sellimonas	0.69	0.26	0.70	0.52	0.38	0.96	0.22	0.72	0.98	0.18	0.77	0.98	0.20	0.75	0.98
Senegalimassilia	0.70	0.22	0.68	0.73	0.19	0.84	0.70	0.23	0.98	0.69	0.23	0.98	0.70	0.23	0.98
Shuttleworthia	0.00	1.00	1.00	-0.60	0.34	0.96	-0.79	0.23	0.98	-0.83	0.21	0.98	-0.76	0.25	0.98
Slackia	-0.07	0.90	0.98	0.06	0.90	0.96	0.04	0.94	0.98	0.05	0.93	0.98	-0.05	0.93	0.98
Streptococcus	-0.85	0.25	0.70	-0.86	0.23	0.86	-0.31	0.68	0.98	-0.33	0.67	0.98	-0.32	0.68	0.98
Subdoligranulum	0.83	0.13	0.68	0.60	0.27	0.93	0.25	0.66	0.98	0.24	0.68	0.98	0.23	0.68	0.98
Sutterella	-0.18	0.59	0.94	0.06	0.86	0.96	0.02	0.95	0.98	0.02	0.95	0.98	0.03	0.93	0.98
TM7x	-1.27	0.10	0.63	-1.63	0.03	0.78	-1.16	0.14	0.98	-1.16	0.13	0.98	-1.13	0.15	0.98
Turicibacter	0.75	0.33	0.76	1.08	0.15	0.84	1.24	0.11	0.98	1.25	0.11	0.98	1.09	0.17	0.98
Tuzzerella	-0.06	0.90	0.98	0.03	0.95	0.96	-0.27	0.63	0.98	-0.28	0.62	0.98	-0.36	0.51	0.98
UBA1819	0.31	0.70	0.98	0.19	0.81	0.96	0.35	0.67	0.98	0.29	0.73	0.98	0.33	0.70	0.98
UCG.002	0.39	0.32	0.76	0.14	0.71	0.96	-0.23	0.57	0.98	-0.26	0.53	0.98	-0.28	0.50	0.98
UCG.003	0.87	0.05	0.53	0.58	0.18	0.84	0.40	0.39	0.98	0.39	0.39	0.98	0.31	0.50	0.98
UCG.005	0.90	0.07	0.53	0.66	0.17	0.84	0.42	0.41	0.98	0.39	0.45	0.98	0.36	0.49	0.98
UCG.009	0.12	0.87	0.98	-0.38	0.60	0.96	-0.89	0.24	0.98	-0.91	0.24	0.98	-0.92	0.23	0.98
Veillonella	0.04	0.94	0.98	0.04	0.93	0.96	0.19	0.73	0.98	0.21	0.71	0.98	0.22	0.69	0.98
Victivallis	0.53	0.33	0.76	0.27	0.61	0.96	-0.21	0.70	0.98	-0.24	0.67	0.98	-0.19	0.74	0.98

Multivariable-adjusted regression models adjusted for:
Model 1: sequencing batch, age, race, gender, study center
Model 2: Model 1, with education

© 2022 Meyer K et al. *JAMA Network Open.*

Model 3: Model 2, with physical activity, diet score, medication use, and smoking
Model 4: Model 3 with BMI
Model 5: Model 4 with hypertension and diabetes

© 2022 Meyer K et al. *JAMA Network Open.*

eTable 17: Multivariable-adjusted regression coefficients, p-values, and false discovery rate (FDR)-adjusted p-values for genera with respect to MOCA.

	Model1			Model2			Model3			Model4			Model5		
	beta	p-value	FDR	beta	p-value	FDR	beta	p-value	FDR	beta	p-value	FDR	beta	p-value	FDR
Acetanaerobacterium	-0.31	0.28	0.50	-0.15	0.58	0.79	-0.25	0.35	0.76	-0.26	0.35	0.76	-0.28	0.31	0.76
Acidaminococcus	-0.29	0.00	0.03	-0.18	0.04	0.46	-0.17	0.06	0.60	-0.17	0.06	0.62	-0.16	0.08	0.71
Actinomyces	-0.14	0.58	0.71	-0.05	0.84	0.94	0.03	0.91	0.97	0.03	0.92	0.98	0.08	0.74	0.89
Adlercreutzia	0.01	0.95	0.97	-0.03	0.84	0.94	-0.08	0.58	0.80	-0.08	0.58	0.80	-0.08	0.59	0.79
Agathobacter	-0.26	0.18	0.40	-0.29	0.10	0.62	-0.32	0.07	0.61	-0.32	0.08	0.64	-0.30	0.10	0.75
Akkermansia	0.21	0.03	0.17	0.13	0.17	0.63	0.09	0.33	0.76	0.09	0.33	0.76	0.08	0.41	0.76
Alistipes	0.26	0.11	0.31	0.17	0.25	0.65	0.08	0.61	0.80	0.08	0.61	0.80	0.06	0.70	0.88
Allisonella	-0.33	0.09	0.28	-0.03	0.88	0.94	-0.05	0.78	0.89	-0.05	0.79	0.89	-0.06	0.74	0.89
Anaerofilum	0.15	0.48	0.66	-0.12	0.57	0.79	-0.13	0.53	0.80	-0.13	0.52	0.79	-0.13	0.54	0.79
Anaerostipes	0.24	0.30	0.52	0.12	0.57	0.79	0.11	0.60	0.80	0.11	0.60	0.80	0.14	0.51	0.79
Anaerotruncus	0.20	0.32	0.54	0.12	0.53	0.79	0.19	0.31	0.76	0.19	0.32	0.76	0.19	0.32	0.76
Bacteroides	0.02	0.96	0.97	-0.09	0.79	0.91	-0.10	0.75	0.89	-0.10	0.76	0.89	-0.09	0.78	0.91
Barnesiella	0.16	0.08	0.28	0.16	0.06	0.53	0.19	0.04	0.51	0.18	0.04	0.52	0.20	0.03	0.43
Bifidobacterium	-0.11	0.34	0.54	0.02	0.89	0.94	0.01	0.96	0.98	0.01	0.95	0.99	0.00	0.99	0.99
Bilophila	0.15	0.24	0.46	0.08	0.49	0.79	0.01	0.97	0.98	0.01	0.96	0.99	0.01	0.92	0.96
Blautia	-0.04	0.92	0.97	-0.24	0.55	0.79	-0.10	0.80	0.89	-0.11	0.79	0.89	0.05	0.91	0.96
Butyricicoccus	0.30	0.30	0.52	-0.04	0.89	0.94	-0.20	0.48	0.76	-0.20	0.48	0.77	-0.21	0.46	0.79
Butyricimonas	0.15	0.17	0.40	0.09	0.39	0.74	0.11	0.29	0.76	0.11	0.29	0.76	0.09	0.39	0.76
CAG.56	0.10	0.42	0.61	0.02	0.85	0.94	0.02	0.87	0.95	0.02	0.87	0.95	0.03	0.78	0.91
Candidatus_Soleaferrea	-0.19	0.44	0.62	-0.31	0.19	0.63	-0.27	0.25	0.76	-0.27	0.25	0.76	-0.27	0.25	0.76
Caproiciproducens	-0.08	0.69	0.80	-0.08	0.68	0.86	-0.11	0.58	0.80	-0.11	0.57	0.80	-0.11	0.60	0.79
Christensenellaceae_R.7_group	0.28	0.01	0.07	0.18	0.06	0.53	0.18	0.07	0.61	0.18	0.08	0.64	0.19	0.07	0.71
Clostridium_sensu_stricto_1	0.06	0.65	0.77	0.09	0.44	0.76	0.11	0.38	0.76	0.11	0.38	0.76	0.11	0.37	0.76
Colidextribacter	-0.17	0.52	0.69	-0.30	0.22	0.63	-0.21	0.39	0.76	-0.22	0.39	0.76	-0.17	0.50	0.79
Collinsella	-0.09	0.42	0.61	0.07	0.54	0.79	0.05	0.68	0.86	0.05	0.67	0.85	0.04	0.73	0.89
Coprobacter	0.09	0.48	0.66	0.01	0.91	0.94	-0.03	0.80	0.89	-0.03	0.79	0.89	-0.03	0.83	0.91
Coprococcus	0.05	0.70	0.80	0.04	0.77	0.91	0.07	0.60	0.80	0.07	0.60	0.80	0.07	0.60	0.79
Defluviitaleaceae_UCG.011	0.34	0.08	0.28	0.25	0.19	0.63	0.24	0.20	0.76	0.24	0.20	0.76	0.22	0.25	0.76
Desulfovibrio	-0.13	0.25	0.46	-0.11	0.30	0.72	-0.07	0.52	0.80	-0.07	0.50	0.77	-0.07	0.51	0.79
Dialister	-0.05	0.54	0.69	-0.05	0.58	0.79	-0.06	0.49	0.77	-0.06	0.50	0.77	-0.05	0.60	0.79
DNF00809	0.19	0.39	0.58	0.12	0.56	0.79	0.28	0.19	0.76	0.28	0.20	0.76	0.28	0.19	0.76
Dorea	0.02	0.92	0.97	0.06	0.70	0.87	0.10	0.55	0.80	0.10	0.54	0.80	0.09	0.57	0.79
DTU089	0.16	0.38	0.57	0.11	0.52	0.79	0.13	0.45	0.76	0.13	0.46	0.76	0.14	0.42	0.76
Eggerthella	-0.17	0.34	0.54	-0.21	0.19	0.63	-0.14	0.41	0.76	-0.14	0.41	0.76	-0.10	0.54	0.79
Eisenbergiella	0.01	0.95	0.97	0.02	0.92	0.95	0.01	0.93	0.98	0.01	0.96	0.99	0.03	0.85	0.91
Erysipelatoclostridium	-0.13	0.49	0.66	-0.12	0.50	0.79	-0.06	0.76	0.89	-0.06	0.74	0.88	-0.04	0.81	0.91
Erysipelotrichaceae_UCG.003	0.13	0.35	0.54	0.10	0.44	0.76	0.04	0.74	0.88	0.04	0.74	0.88	0.03	0.80	0.91
Escherichia.Shigella	-0.16	0.21	0.43	-0.06	0.62	0.81	-0.09	0.44	0.76	-0.09	0.45	0.76	-0.10	0.42	0.76
Faecalibacterium	0.35	0.05	0.23	0.25	0.14	0.63	0.21	0.22	0.76	0.21	0.22	0.76	0.21	0.24	0.76
Family_XIII_AD3011_group	0.37	0.03	0.16	0.21	0.19	0.63	0.22	0.19	0.76	0.21	0.20	0.76	0.21	0.22	0.76
Family_XIII_UCG.001	0.26	0.20	0.43	0.15	0.45	0.76	0.16	0.43	0.76	0.15	0.45	0.76	0.14	0.48	0.79
FD2005	-0.31	0.12	0.31	-0.23	0.21	0.63	-0.20	0.30	0.76	-0.20	0.30	0.76	-0.21	0.27	0.76
Flavonifractor	-0.16	0.35	0.54	-0.15	0.36	0.74	-0.06	0.71	0.86	-0.06	0.71	0.86	-0.03	0.85	0.91
Fournierella	0.12	0.54	0.69	0.09	0.63	0.81	0.14	0.44	0.76	0.14	0.45	0.76	0.13	0.47	0.79
Frisingicoccus	0.26	0.08	0.28	0.20	0.15	0.63	0.12	0.39	0.76	0.12	0.40	0.76	0.13	0.33	0.76
Fusicatenibacter	-0.02	0.91	0.97	-0.10	0.45	0.76	-0.16	0.22	0.76	-0.16	0.23	0.76	-0.16	0.23	0.76
Fusobacterium	-0.29	0.16	0.40	-0.18	0.37	0.74	-0.11	0.59	0.80	-0.11	0.57	0.80	-0.12	0.54	0.79
GCA.900066575	0.43	0.02	0.15	0.26	0.15	0.63	0.20	0.26	0.76	0.20	0.27	0.76	0.19	0.30	0.76
Gordonibacter	0.01	0.97	0.98	-0.09	0.63	0.81	-0.07	0.70	0.86	-0.08	0.68	0.85	-0.07	0.71	0.88
Granulicatella	-0.24	0.34	0.54	-0.21	0.38	0.74	-0.25	0.29	0.76	-0.25	0.30	0.76	-0.25	0.31	0.76
Haemophilus	0.29	0.04	0.18	0.22	0.09	0.62	0.11	0.42	0.76	0.11	0.42	0.76	0.11	0.42	0.76
Harryflintia	-0.12	0.58	0.71	-0.17	0.39	0.74	-0.16	0.43	0.76	-0.17	0.42	0.76	-0.18	0.39	0.76
Holdemanella	-0.07	0.62	0.76	-0.02	0.88	0.94	0.10	0.47	0.76	0.10	0.46	0.76	0.11	0.40	0.76
Holdemania	-0.36	0.28	0.50	-0.36	0.25	0.65	-0.36	0.25	0.76	-0.37	0.24	0.76	-0.41	0.20	0.76
Hungatella	-0.34	0.05	0.23	-0.27	0.09	0.62	-0.20	0.23	0.76	-0.20	0.23	0.76	-0.19	0.25	0.76

© 2022 Meyer K et al. *JAMA Network Open.*

Hydrogenoanaerobacterium	0.43	0.02	0.15	0.22	0.22	0.63	0.18	0.32	0.76	0.18	0.33	0.76	0.20	0.28	0.76
Incertae_Sedis	-0.06	0.82	0.91	-0.02	0.93	0.95	0.14	0.60	0.80	0.14	0.60	0.80	0.19	0.49	0.79
Intestinibacter	0.16	0.35	0.54	0.26	0.09	0.62	0.26	0.10	0.76	0.27	0.10	0.73	0.29	0.07	0.71
Intestinimonas	0.29	0.06	0.26	0.13	0.38	0.74	0.09	0.55	0.80	0.09	0.56	0.80	0.10	0.50	0.79
Lachnoclostridium	-1.05	0.00	0.03	-0.74	0.02	0.39	-0.69	0.03	0.51	-0.69	0.03	0.52	-0.68	0.03	0.47
Lachnospira	0.58	0.00	0.02	0.34	0.03	0.41	0.13	0.40	0.76	0.14	0.39	0.76	0.15	0.34	0.76
Lachnospiraceae_FCS020_group	0.58	0.00	0.02	0.37	0.02	0.39	0.36	0.03	0.51	0.36	0.03	0.52	0.37	0.03	0.43
Lachnospiraceae_ND3007_group	0.15	0.19	0.42	0.03	0.76	0.91	0.04	0.70	0.86	0.04	0.68	0.85	0.05	0.68	0.87
Lachnospiraceae_NK4A136_group	0.22	0.17	0.40	0.02	0.89	0.94	-0.16	0.31	0.76	-0.16	0.31	0.76	-0.16	0.33	0.76
Lachnospiraceae_UCG.001	0.20	0.10	0.29	0.14	0.22	0.63	0.02	0.85	0.94	0.02	0.87	0.95	0.03	0.82	0.91
Lachnospiraceae_UCG.004	0.26	0.06	0.24	0.18	0.15	0.63	0.12	0.33	0.76	0.13	0.33	0.76	0.12	0.37	0.76
Lachnospiraceae_UCG.010	0.00	1.00	1.00	-0.15	0.40	0.75	-0.19	0.29	0.76	-0.20	0.28	0.76	-0.22	0.24	0.76
Lactobacillus	-0.40	0.01	0.10	-0.21	0.17	0.63	-0.17	0.28	0.76	-0.16	0.29	0.76	-0.18	0.27	0.76
Lactococcus	0.25	0.24	0.46	0.17	0.40	0.75	0.25	0.22	0.76	0.25	0.22	0.76	0.24	0.24	0.76
Marvinbryantia	0.13	0.32	0.54	0.04	0.75	0.91	0.15	0.22	0.76	0.15	0.22	0.76	0.16	0.20	0.76
Megasphaera	-0.34	0.00	0.04	-0.21	0.05	0.53	-0.12	0.29	0.76	-0.12	0.29	0.76	-0.12	0.29	0.76
Monoglobus	0.48	0.00	0.05	0.38	0.01	0.39	0.34	0.04	0.51	0.34	0.04	0.52	0.36	0.03	0.43
Moryella	0.36	0.10	0.30	0.18	0.38	0.74	0.18	0.41	0.76	0.18	0.41	0.76	0.19	0.38	0.76
Negativibacillus	-0.17	0.23	0.46	-0.14	0.29	0.71	-0.10	0.45	0.76	-0.10	0.45	0.76	-0.09	0.50	0.79
NK4A214_group	0.19	0.09	0.28	0.10	0.33	0.74	0.08	0.44	0.76	0.08	0.45	0.76	0.08	0.43	0.76
Odoribacter	0.14	0.18	0.40	0.09	0.36	0.74	0.07	0.50	0.77	0.07	0.50	0.77	0.06	0.55	0.79
Oscillibacter	0.26	0.15	0.38	0.17	0.32	0.74	0.19	0.29	0.76	0.18	0.30	0.76	0.20	0.25	0.76
Oscillospira	0.11	0.66	0.77	0.13	0.58	0.79	0.20	0.40	0.76	0.20	0.41	0.76	0.25	0.30	0.76
Oxalobacter	0.32	0.08	0.28	0.07	0.68	0.86	0.05	0.77	0.89	0.05	0.77	0.89	0.05	0.78	0.91
Paludicola	0.41	0.03	0.16	0.21	0.23	0.63	0.16	0.36	0.76	0.16	0.38	0.76	0.19	0.31	0.76
Parabacteroides	0.02	0.87	0.95	0.12	0.35	0.74	0.14	0.26	0.76	0.14	0.25	0.76	0.14	0.26	0.76
Paraprevotella	-0.08	0.43	0.61	-0.03	0.77	0.91	-0.09	0.34	0.76	-0.09	0.33	0.76	-0.08	0.42	0.76
Parasutterella	0.34	0.00	0.02	0.30	0.00	0.06	0.24	0.01	0.36	0.24	0.01	0.37	0.24	0.01	0.39
Peptococcus	0.35	0.07	0.28	0.19	0.30	0.72	0.18	0.33	0.76	0.18	0.34	0.76	0.18	0.34	0.76
Phascolarctobacterium	0.15	0.12	0.31	0.16	0.06	0.53	0.17	0.06	0.60	0.17	0.06	0.62	0.15	0.10	0.75
Phocea	-0.38	0.11	0.31	-0.46	0.04	0.46	-0.56	0.01	0.36	-0.56	0.01	0.37	-0.56	0.01	0.39
Prevotella	-0.17	0.05	0.23	-0.10	0.22	0.63	-0.11	0.20	0.76	-0.11	0.20	0.76	-0.11	0.20	0.76
Romboutsia	-0.18	0.24	0.46	-0.12	0.42	0.76	-0.08	0.59	0.80	-0.08	0.60	0.80	-0.08	0.59	0.79
Roseburia	0.31	0.08	0.28	0.25	0.14	0.63	0.12	0.48	0.76	0.12	0.47	0.76	0.14	0.43	0.76
Ruminococcus	0.27	0.02	0.15	0.15	0.16	0.63	0.12	0.28	0.76	0.12	0.28	0.76	0.13	0.24	0.76
Sellimonas	0.20	0.23	0.46	0.12	0.44	0.76	0.13	0.40	0.76	0.13	0.41	0.76	0.16	0.32	0.76
Senegalimassilia	-0.09	0.57	0.71	-0.08	0.59	0.79	-0.02	0.91	0.97	-0.02	0.91	0.98	-0.01	0.94	0.96
Shuttleworthia	0.43	0.01	0.10	0.18	0.26	0.67	0.12	0.46	0.76	0.12	0.47	0.76	0.15	0.37	0.76
Slackia	-0.04	0.79	0.89	0.01	0.94	0.95	0.04	0.77	0.89	0.04	0.76	0.89	0.03	0.81	0.91
Streptococcus	0.26	0.19	0.41	0.25	0.18	0.63	0.31	0.11	0.76	0.31	0.12	0.76	0.31	0.12	0.76
Subdoligranulum	0.42	0.00	0.05	0.32	0.02	0.39	0.35	0.01	0.36	0.35	0.01	0.37	0.36	0.01	0.39
Sutterella	-0.36	0.00	0.00	-0.27	0.00	0.06	-0.26	0.00	0.21	-0.26	0.00	0.21	-0.27	0.00	0.13
TM7x	0.09	0.67	0.78	-0.05	0.78	0.91	0.01	0.96	0.98	0.01	0.97	0.99	0.04	0.84	0.91
Turicibacter	0.14	0.49	0.66	0.28	0.16	0.63	0.38	0.06	0.60	0.38	0.06	0.62	0.36	0.07	0.71
Tuzzerella	-0.02	0.88	0.95	0.03	0.83	0.94	0.00	0.98	0.99	0.00	0.99	0.99	-0.01	0.92	0.96
UBA1819	0.05	0.82	0.91	0.01	0.96	0.96	0.09	0.67	0.86	0.08	0.69	0.85	0.12	0.56	0.79
UCG.002	0.27	0.01	0.10	0.16	0.10	0.62	0.14	0.16	0.76	0.14	0.17	0.76	0.14	0.17	0.76
UCG.003	0.19	0.12	0.31	0.06	0.58	0.79	0.01	0.92	0.97	0.01	0.92	0.98	-0.01	0.96	0.97
UCG.005	0.29	0.03	0.16	0.18	0.14	0.63	0.14	0.27	0.76	0.14	0.28	0.76	0.15	0.27	0.76
UCG.009	0.32	0.11	0.31	0.11	0.56	0.79	0.08	0.68	0.86	0.08	0.69	0.85	0.08	0.68	0.87
Veillonella	0.09	0.55	0.70	0.08	0.56	0.79	0.00	0.99	0.99	0.00	0.98	0.99	-0.01	0.93	0.96
Victivallis	0.07	0.65	0.77	-0.04	0.75	0.91	-0.08	0.56	0.80	-0.09	0.55	0.80	-0.08	0.57	0.79

Multivariable-adjusted regression models adjusted for:
Model 1: sequencing batch, age, race, gender, study center
Model 2: Model 1, with education
Model 3: Model 2, with physical activity, diet score, medication use, and smoking
Model 4: Model 3 with BMI
Model 5: Model 4 with hypertension and diabetes

© 2022 Meyer K et al. *JAMA Network Open.*

eTable 18: Multivariable-adjusted regression coefficients, p-values, and false discovery rate (FDR)-adjusted p-values for genera with respect to PC (1st).

	Model1			Model2			Model3			Model4			Model5		
	beta	p-value	FDR	beta	p-value	FDR	beta	p-value	FDR	beta	p-value	FDR	beta	p-value	FDR
Acetanaerobacterium	-0.06	0.65	0.77	0.02	0.85	0.89	-0.02	0.87	0.96	-0.03	0.84	0.96	-0.04	0.76	0.96
Acidaminococcus	-0.14	0.00	0.01	-0.09	0.03	0.24	-0.07	0.12	0.72	-0.07	0.13	0.69	-0.07	0.14	0.74
Actinomyces	-0.10	0.37	0.56	-0.06	0.59	0.83	0.02	0.89	0.97	0.02	0.89	0.97	0.03	0.79	0.96
Adlercreutzia	0.04	0.54	0.72	0.02	0.71	0.89	0.01	0.94	0.99	0.00	0.96	0.99	0.00	0.98	0.99
Agathobacter	-0.01	0.91	0.94	-0.03	0.75	0.89	-0.09	0.28	0.85	-0.08	0.32	0.96	-0.07	0.39	0.96
Akkermansia	0.11	0.02	0.08	0.07	0.10	0.37	0.05	0.23	0.76	0.05	0.23	0.79	0.05	0.26	0.82
Alistipes	0.15	0.04	0.13	0.11	0.11	0.38	0.05	0.54	0.94	0.05	0.54	0.96	0.04	0.60	0.96
Allisonella	-0.17	0.06	0.15	-0.02	0.83	0.89	0.00	0.99	0.99	0.00	0.97	0.99	0.01	0.95	0.98
Anaerofilum	0.20	0.05	0.14	0.06	0.49	0.79	0.05	0.58	0.94	0.05	0.62	0.96	0.05	0.63	0.96
Anaerostipes	0.03	0.81	0.88	-0.03	0.74	0.89	-0.05	0.66	0.94	-0.05	0.66	0.96	-0.03	0.76	0.96
Anaerotruncus	0.08	0.39	0.57	0.04	0.66	0.88	0.01	0.87	0.96	0.00	0.96	0.99	0.00	0.96	0.98
Bacteroides	0.00	0.99	0.99	-0.05	0.73	0.89	-0.06	0.72	0.94	-0.05	0.77	0.96	-0.05	0.73	0.96
Barnesiella	0.17	0.00	0.00	0.17	0.00	0.00	0.15	0.00	0.02	0.15	0.00	0.02	0.16	0.00	0.01
Bifidobacterium	-0.03	0.59	0.73	0.04	0.50	0.79	0.02	0.70	0.94	0.02	0.66	0.96	0.02	0.67	0.96
Bilophila	0.13	0.02	0.10	0.10	0.07	0.32	0.08	0.17	0.73	0.08	0.16	0.69	0.07	0.21	0.78
Blautia	0.03	0.88	0.94	-0.07	0.70	0.89	-0.02	0.92	0.99	-0.02	0.90	0.97	0.02	0.90	0.98
Butyricicoccus	0.20	0.14	0.30	0.03	0.79	0.89	-0.06	0.67	0.94	-0.06	0.68	0.96	-0.05	0.72	0.96
Butyricimonas	0.10	0.05	0.14	0.07	0.16	0.45	0.06	0.20	0.73	0.06	0.20	0.76	0.06	0.22	0.78
CAG.56	0.08	0.16	0.34	0.04	0.45	0.75	0.01	0.85	0.96	0.01	0.85	0.96	0.01	0.79	0.96
Candidatus_Soleaferrea	-0.01	0.95	0.96	-0.06	0.55	0.80	-0.05	0.66	0.94	-0.05	0.65	0.96	-0.06	0.62	0.96
Caproiciproducens	0.01	0.91	0.94	0.01	0.88	0.91	-0.04	0.68	0.94	-0.04	0.65	0.96	-0.05	0.62	0.96
Christensenellaceae_R.7_group	0.16	0.00	0.01	0.11	0.01	0.14	0.08	0.12	0.72	0.07	0.14	0.69	0.08	0.13	0.74
Clostridium_sensu_stricto_1	0.05	0.46	0.64	0.06	0.27	0.62	0.06	0.34	0.93	0.06	0.34	0.96	0.05	0.38	0.96
Colidextribacter	-0.10	0.43	0.61	-0.16	0.15	0.45	-0.16	0.18	0.73	-0.16	0.17	0.69	-0.14	0.24	0.78
Collinsella	-0.07	0.19	0.37	0.01	0.85	0.89	0.01	0.90	0.98	0.01	0.87	0.97	0.00	0.93	0.98
Coprobacter	0.06	0.28	0.48	0.02	0.66	0.88	-0.02	0.70	0.94	-0.02	0.67	0.96	-0.02	0.67	0.96
Coprococcus	0.10	0.12	0.27	0.09	0.12	0.40	0.08	0.19	0.73	0.08	0.19	0.75	0.09	0.16	0.74
Defluviitaleaceae_UCG.011	0.26	0.00	0.04	0.21	0.01	0.14	0.19	0.03	0.62	0.19	0.04	0.57	0.18	0.04	0.57
Desulfovibrio	-0.04	0.51	0.69	-0.03	0.61	0.83	-0.03	0.52	0.94	-0.04	0.45	0.96	-0.04	0.48	0.96
Dialister	-0.02	0.59	0.73	-0.02	0.63	0.85	-0.01	0.73	0.94	-0.01	0.75	0.96	-0.01	0.81	0.96
DNF00809	0.09	0.39	0.57	0.06	0.56	0.81	0.07	0.50	0.94	0.06	0.53	0.96	0.06	0.53	0.96
Dorea	-0.01	0.93	0.95	0.02	0.83	0.89	0.02	0.80	0.95	0.02	0.77	0.96	0.02	0.83	0.98
DTU089	0.03	0.69	0.79	0.01	0.90	0.92	0.00	0.99	0.99	-0.01	0.94	0.99	0.01	0.92	0.98
Eggerthella	-0.04	0.61	0.74	-0.06	0.40	0.70	-0.02	0.80	0.95	-0.02	0.78	0.96	-0.01	0.89	0.98
Eisenbergiella	-0.05	0.56	0.73	-0.05	0.54	0.80	-0.04	0.60	0.94	-0.05	0.52	0.96	-0.04	0.62	0.96
Erysipelatoclostridium	-0.07	0.43	0.61	-0.06	0.45	0.75	-0.03	0.71	0.94	-0.04	0.65	0.96	-0.03	0.69	0.96
Erysipelotrichaceae_UCG.003	0.07	0.24	0.43	0.06	0.31	0.62	0.02	0.73	0.94	0.02	0.74	0.96	0.02	0.77	0.96
Escherichia.Shigella	-0.07	0.24	0.43	-0.02	0.73	0.89	0.00	0.98	0.99	0.00	0.99	0.99	0.00	0.96	0.98
Faecalibacterium	0.20	0.02	0.09	0.15	0.06	0.32	0.12	0.16	0.73	0.12	0.15	0.69	0.13	0.14	0.74
Family_XIII_AD3011_group	0.21	0.01	0.05	0.12	0.09	0.36	0.07	0.39	0.94	0.06	0.45	0.96	0.06	0.46	0.96
Family_XIII_UCG.001	0.18	0.07	0.16	0.12	0.18	0.47	0.08	0.39	0.94	0.07	0.44	0.96	0.07	0.44	0.96
FD2005	-0.06	0.49	0.67	-0.02	0.79	0.89	-0.02	0.82	0.95	-0.02	0.84	0.96	-0.02	0.84	0.98
Flavonifractor	-0.12	0.13	0.30	-0.12	0.12	0.40	-0.07	0.36	0.94	-0.07	0.37	0.96	-0.07	0.41	0.96
Fournierella	0.04	0.66	0.77	0.02	0.78	0.89	-0.01	0.95	0.99	-0.01	0.89	0.97	0.00	0.99	0.99
Frisingicoccus	0.03	0.67	0.77	0.00	1.00	1.00	-0.04	0.57	0.94	-0.04	0.53	0.96	-0.04	0.58	0.96
Fusicatenibacter	0.06	0.38	0.56	0.02	0.79	0.89	-0.02	0.74	0.94	-0.02	0.81	0.96	-0.02	0.74	0.96
Fusobacterium	-0.11	0.28	0.48	-0.05	0.60	0.83	0.03	0.73	0.94	0.03	0.77	0.96	0.03	0.78	0.96
GCA.900066575	0.18	0.05	0.13	0.09	0.29	0.62	0.03	0.74	0.94	0.02	0.81	0.96	0.01	0.87	0.98
Gordonibacter	0.10	0.27	0.47	0.05	0.53	0.80	0.03	0.70	0.94	0.02	0.78	0.96	0.02	0.80	0.96
Granulicatella	-0.04	0.72	0.80	-0.03	0.81	0.89	0.00	0.98	0.99	0.00	0.99	0.99	0.02	0.89	0.98
Haemophilus	0.14	0.03	0.10	0.11	0.07	0.32	0.07	0.27	0.84	0.07	0.27	0.85	0.08	0.22	0.78
Harryflintia	-0.01	0.90	0.94	-0.04	0.67	0.88	-0.05	0.59	0.94	-0.06	0.55	0.96	-0.07	0.49	0.96
Holdemanella	-0.02	0.70	0.79	0.00	1.00	1.00	0.03	0.65	0.94	0.03	0.63	0.96	0.03	0.62	0.96
Holdemania	0.09	0.59	0.73	0.09	0.54	0.80	0.08	0.62	0.94	0.07	0.66	0.96	0.04	0.80	0.96
Hungatella	-0.22	0.01	0.05	-0.18	0.01	0.14	-0.13	0.10	0.72	-0.13	0.09	0.69	-0.12	0.13	0.74

© 2022 Meyer K et al. *JAMA Network Open.*

Hydrogenoanaerobacterium	0.28	0.00	0.02	0.17	0.03	0.27	0.13	0.14	0.72	0.12	0.16	0.69	0.12	0.16	0.74
Incertae_Sedis	0.12	0.37	0.56	0.14	0.25	0.59	0.15	0.24	0.78	0.15	0.25	0.80	0.16	0.23	0.78
Intestinibacter	0.10	0.22	0.42	0.15	0.04	0.28	0.14	0.07	0.66	0.15	0.05	0.57	0.16	0.04	0.57
Intestinimonas	0.20	0.01	0.04	0.12	0.08	0.33	0.09	0.21	0.73	0.08	0.24	0.80	0.10	0.19	0.76
Lachnoclostridium	-0.53	0.00	0.01	-0.37	0.01	0.13	-0.22	0.15	0.73	-0.21	0.16	0.69	-0.21	0.17	0.74
Lachnospira	0.33	0.00	0.00	0.21	0.00	0.07	0.13	0.08	0.72	0.14	0.07	0.64	0.15	0.05	0.57
Lachnospiraceae_FCS020_group	0.35	0.00	0.00	0.24	0.00	0.04	0.21	0.01	0.28	0.21	0.01	0.28	0.21	0.01	0.32
Lachnospiraceae_ND3007_group	0.11	0.04	0.13	0.05	0.31	0.62	0.03	0.63	0.94	0.03	0.59	0.96	0.03	0.57	0.96
Lachnospiraceae_NK4A136_group	0.11	0.14	0.30	0.01	0.88	0.91	-0.11	0.17	0.73	-0.11	0.17	0.69	-0.10	0.19	0.76
Lachnospiraceae_UCG.001	0.12	0.03	0.11	0.09	0.09	0.36	0.04	0.47	0.94	0.04	0.51	0.96	0.04	0.48	0.96
Lachnospiraceae_UCG.004	0.09	0.14	0.30	0.06	0.34	0.66	0.03	0.59	0.94	0.04	0.56	0.96	0.02	0.71	0.96
Lachnospiraceae_UCG.010	-0.05	0.60	0.74	-0.13	0.14	0.43	-0.16	0.07	0.66	-0.17	0.06	0.57	-0.17	0.05	0.57
Lactobacillus	-0.21	0.00	0.04	-0.12	0.09	0.36	-0.12	0.09	0.72	-0.12	0.11	0.69	-0.12	0.11	0.74
Lactococcus	0.12	0.23	0.43	0.08	0.39	0.70	0.09	0.37	0.94	0.08	0.39	0.96	0.07	0.47	0.96
Marvinbryantia	0.03	0.57	0.73	-0.01	0.83	0.89	-0.01	0.87	0.96	-0.01	0.85	0.96	0.00	0.96	0.98
Megasphaera	-0.10	0.06	0.15	-0.03	0.52	0.80	0.03	0.63	0.94	0.03	0.62	0.96	0.02	0.71	0.96
Monoglobus	0.28	0.00	0.01	0.23	0.00	0.04	0.18	0.02	0.56	0.18	0.02	0.57	0.18	0.02	0.51
Moryella	0.23	0.03	0.10	0.14	0.15	0.45	0.06	0.54	0.94	0.06	0.54	0.96	0.07	0.52	0.96
Negativibacillus	-0.06	0.33	0.52	-0.05	0.41	0.71	-0.05	0.46	0.94	-0.05	0.45	0.96	-0.05	0.39	0.96
NK4A214_group	0.10	0.05	0.14	0.05	0.25	0.59	0.01	0.81	0.95	0.01	0.87	0.97	0.01	0.80	0.96
Odoribacter	0.11	0.02	0.10	0.08	0.06	0.32	0.05	0.35	0.93	0.04	0.36	0.96	0.04	0.39	0.96
Oscillibacter	0.19	0.02	0.10	0.15	0.06	0.32	0.13	0.13	0.72	0.12	0.15	0.69	0.12	0.16	0.74
Oscillospira	0.08	0.51	0.69	0.09	0.42	0.73	0.11	0.34	0.93	0.10	0.36	0.96	0.13	0.28	0.84
Oxalobacter	0.23	0.01	0.04	0.11	0.16	0.46	0.06	0.50	0.94	0.05	0.52	0.96	0.05	0.52	0.96
Paludicola	0.20	0.02	0.09	0.10	0.20	0.50	0.03	0.69	0.94	0.02	0.80	0.96	0.03	0.69	0.96
Parabacteroides	0.03	0.66	0.77	0.07	0.19	0.49	0.08	0.17	0.73	0.08	0.16	0.69	0.09	0.15	0.74
Paraprevotella	-0.04	0.37	0.56	-0.02	0.70	0.89	-0.03	0.52	0.94	-0.03	0.49	0.96	-0.03	0.52	0.96
Parasutterella	0.15	0.00	0.01	0.14	0.00	0.04	0.09	0.05	0.63	0.09	0.05	0.57	0.08	0.07	0.63
Peptococcus	0.13	0.17	0.34	0.04	0.60	0.83	-0.02	0.79	0.95	-0.03	0.75	0.96	-0.02	0.79	0.96
Phascolarctobacterium	0.08	0.08	0.19	0.09	0.03	0.27	0.09	0.04	0.62	0.08	0.05	0.57	0.08	0.05	0.57
Phocea	-0.15	0.18	0.35	-0.19	0.07	0.32	-0.21	0.05	0.63	-0.20	0.06	0.57	-0.20	0.06	0.62
Prevotella	-0.09	0.03	0.11	-0.05	0.19	0.49	-0.05	0.21	0.73	-0.05	0.21	0.76	-0.05	0.24	0.78
Romboutsia	0.02	0.73	0.81	0.06	0.39	0.70	0.04	0.55	0.94	0.05	0.52	0.96	0.05	0.50	0.96
Roseburia	0.12	0.17	0.34	0.08	0.28	0.62	0.02	0.81	0.95	0.02	0.78	0.96	0.04	0.66	0.96
Ruminococcus	0.14	0.01	0.05	0.08	0.10	0.37	0.04	0.42	0.94	0.04	0.44	0.96	0.04	0.41	0.96
Sellimonas	0.17	0.03	0.10	0.13	0.07	0.32	0.12	0.11	0.72	0.11	0.12	0.69	0.12	0.11	0.74
Senegalimassilia	0.01	0.90	0.94	0.01	0.84	0.89	0.00	0.96	0.99	0.00	0.97	0.99	0.01	0.93	0.98
Shuttleworthia	0.19	0.02	0.08	0.07	0.35	0.67	0.02	0.79	0.95	0.02	0.83	0.96	0.03	0.75	0.96
Slackia	-0.07	0.30	0.49	-0.04	0.46	0.75	-0.05	0.41	0.94	-0.05	0.42	0.96	-0.06	0.34	0.96
Streptococcus	0.08	0.37	0.56	0.08	0.37	0.69	0.12	0.20	0.73	0.12	0.21	0.76	0.12	0.19	0.76
Subdoligranulum	0.23	0.00	0.01	0.18	0.00	0.08	0.14	0.04	0.62	0.14	0.04	0.57	0.14	0.03	0.57
Sutterella	-0.12	0.00	0.03	-0.07	0.05	0.32	-0.06	0.11	0.72	-0.06	0.11	0.69	-0.06	0.11	0.74
TM7x	-0.02	0.81	0.88	-0.09	0.30	0.62	-0.06	0.51	0.94	-0.06	0.51	0.96	-0.05	0.61	0.96
Turicibacter	0.17	0.07	0.18	0.24	0.01	0.11	0.28	0.00	0.18	0.28	0.00	0.17	0.27	0.01	0.28
Tuzzerella	-0.07	0.29	0.48	-0.05	0.46	0.75	-0.06	0.35	0.93	-0.06	0.34	0.96	-0.07	0.28	0.84
UBA1819	0.04	0.70	0.79	0.02	0.83	0.89	0.04	0.66	0.94	0.03	0.74	0.96	0.04	0.71	0.96
UCG.002	0.11	0.03	0.10	0.05	0.23	0.56	0.01	0.87	0.96	0.00	0.93	0.99	0.01	0.91	0.98
UCG.003	0.12	0.03	0.11	0.05	0.29	0.62	0.01	0.82	0.95	0.01	0.82	0.96	0.00	0.93	0.98
UCG.005	0.17	0.01	0.04	0.12	0.04	0.29	0.06	0.35	0.93	0.05	0.40	0.96	0.06	0.38	0.96
UCG.009	0.13	0.18	0.35	0.02	0.83	0.89	-0.05	0.61	0.94	-0.05	0.59	0.96	-0.05	0.62	0.96
Veillonella	0.07	0.32	0.51	0.06	0.31	0.62	0.04	0.51	0.94	0.05	0.48	0.96	0.05	0.45	0.96
Victivallis	0.14	0.04	0.12	0.09	0.17	0.47	0.03	0.70	0.94	0.02	0.74	0.96	0.03	0.68	0.96

Multivariable-adjusted regression models adjusted for:
Model 1: sequencing batch, age, race, gender, study center
Model 2: Model 1, with education
Model 3: Model 2, with physical activity, diet score, medication use, and smoking
Model 4: Model 3 with BMI
Model 5: Model 4 with hypertension and diabetes

© 2022 Meyer K et al. *JAMA Network Open.*

eTable 19: Multivariable-adjusted regression coefficients, p-values, race interaction p-values, false discovery rate (FDR)-adjusted p-values for race-genera interactions with respect to DSST.

	Model1					Model2					Model3					Model4					Model5				
	beta	p-value	p-val int	FDR	FDR-int	beta	p-value	p-val int	FDR	FDR-int	beta	p-value	p-val int	FDR	FDR-int	beta	p-value	p-val int	FDR	FDR-int	beta	p-value	p-val int	FDR	FDR-int
Acetanaerobacterium	0.67	0.73	0.49	0.86	0.74	0.67	0.72	0.80	0.85	0.93	0.37	0.85	1.00	0.95	1.00	0.37	0.85	1.00	0.94	1.00	0.22	0.91	0.96	0.95	0.97
Acidaminococcus	-0.29	0.62	0.41	0.79	0.69	0.03	0.96	0.60	0.97	0.83	0.48	0.42	0.37	0.84	0.80	0.48	0.42	0.37	0.84	0.80	0.49	0.42	0.36	0.80	0.73
Actinomyces	-2.24	0.14	0.07	0.31	0.33	-1.74	0.23	0.08	0.51	0.44	-1.03	0.51	0.08	0.84	0.59	-1.03	0.51	0.08	0.84	0.59	-0.93	0.55	0.07	0.80	0.62
Adlercreutzia	2.55	0.01	0.10	0.06	0.35	2.10	0.03	0.19	0.18	0.58	2.25	0.03	0.14	0.23	0.59	2.25	0.03	0.14	0.23	0.59	2.20	0.04	0.15	0.27	0.63
Agathobacter	0.12	0.93	0.78	0.97	0.90	-0.10	0.94	0.70	0.96	0.87	-0.84	0.52	0.62	0.84	0.88	-0.84	0.52	0.62	0.84	0.88	-0.77	0.56	0.63	0.80	0.87
Akkermansia	2.42	0.00	0.08	0.01	0.35	1.92	0.00	0.16	0.04	0.56	1.85	0.01	0.24	0.14	0.65	1.85	0.01	0.24	0.14	0.65	1.93	0.01	0.22	0.13	0.63
Alistipes	1.73	0.14	0.36	0.31	0.67	1.16	0.29	0.51	0.55	0.83	0.70	0.54	0.64	0.84	0.89	0.70	0.54	0.64	0.84	0.89	0.67	0.55	0.61	0.80	0.87
Allisonella	-3.15	0.00	0.06	0.03	0.31	-1.79	0.09	0.10	0.31	0.44	-1.10	0.34	0.30	0.77	0.70	-1.11	0.34	0.29	0.77	0.70	-1.04	0.38	0.31	0.80	0.73
Anaerofilum	4.03	0.01	0.09	0.04	0.35	2.83	0.05	0.10	0.22	0.44	3.04	0.05	0.09	0.33	0.59	3.04	0.05	0.09	0.33	0.59	3.17	0.04	0.09	0.30	0.62
Anaerostipes	1.07	0.43	0.53	0.64	0.78	0.52	0.69	0.59	0.84	0.83	1.11	0.41	0.22	0.84	0.64	1.12	0.41	0.22	0.84	0.64	1.22	0.39	0.22	0.80	0.63
Anaerotruncus	4.17	0.00	0.00	0.01	0.07	3.78	0.00	0.00	0.03	0.07	3.16	0.01	0.01	0.14	0.18	3.16	0.01	0.01	0.14	0.18	3.17	0.01	0.01	0.13	0.18
Bacteroides	-0.82	0.67	0.39	0.83	0.69	-1.34	0.46	0.42	0.72	0.77	-0.80	0.66	0.49	0.87	0.87	-0.80	0.66	0.49	0.86	0.87	-0.92	0.62	0.49	0.81	0.83
Barnesiella	1.94	0.00	0.26	0.02	0.56	2.04	0.00	0.16	0.03	0.56	1.66	0.01	0.29	0.14	0.70	1.66	0.01	0.29	0.14	0.70	1.76	0.01	0.27	0.13	0.71
Bifidobacterium	0.20	0.82	0.77	0.90	0.90	0.57	0.49	0.94	0.76	0.98	0.50	0.56	0.78	0.84	0.96	0.51	0.55	0.77	0.84	0.96	0.56	0.52	0.77	0.80	0.95
Bilophila	0.41	0.63	0.68	0.81	0.84	-0.22	0.78	0.86	0.87	0.97	-0.84	0.33	0.48	0.77	0.87	-0.84	0.33	0.48	0.77	0.87	-0.85	0.33	0.51	0.77	0.86
Blautia	5.85	0.03	0.49	0.12	0.74	5.24	0.04	0.41	0.21	0.76	6.78	0.01	0.22	0.14	0.64	6.79	0.01	0.22	0.14	0.64	7.81	0.01	0.17	0.13	0.63
Butyricicoccus	5.07	0.01	0.10	0.04	0.35	3.40	0.06	0.15	0.24	0.56	2.96	0.11	0.22	0.58	0.64	2.98	0.11	0.22	0.58	0.64	3.09	0.10	0.22	0.51	0.63
Butyricimonas	-0.13	0.85	0.29	0.92	0.59	-0.38	0.58	0.26	0.78	0.61	-0.20	0.78	0.50	0.90	0.87	-0.20	0.78	0.49	0.90	0.87	-0.21	0.77	0.52	0.90	0.86
CAG.56	-0.88	0.29	0.01	0.51	0.17	-0.95	0.23	0.02	0.51	0.23	-1.88	0.03	0.00	0.21	0.11	-1.88	0.02	0.00	0.21	0.11	-1.83	0.03	0.01	0.26	0.12
Candidatus_Soleaferrea	5.94	0.00	0.00	0.01	0.03	5.65	0.00	0.00	0.03	0.01	5.25	0.00	0.00	0.11	0.07	5.25	0.00	0.00	0.11	0.07	5.27	0.00	0.00	0.12	0.06
Caproiciproducens	2.13	0.14	0.16	0.31	0.45	1.85	0.18	0.24	0.48	0.59	1.10	0.46	0.34	0.84	0.77	1.10	0.46	0.34	0.84	0.77	1.15	0.45	0.32	0.80	0.73
Christensenellaceae_R.7_group	2.31	0.00	0.17	0.02	0.45	1.69	0.01	0.32	0.09	0.64	1.00	0.17	0.69	0.60	0.94	1.02	0.17	0.68	0.61	0.94	1.16	0.12	0.60	0.53	0.87
Clostridium_sensu_stricto_1	0.57	0.56	0.62	0.74	0.84	0.87	0.35	0.46	0.60	0.80	0.14	0.89	0.86	0.95	0.99	0.14	0.89	0.86	0.95	0.99	0.21	0.84	0.80	0.91	0.96
Colidextribacter	-0.72	0.65	0.34	0.82	0.65	-0.85	0.58	0.17	0.78	0.56	0.28	0.86	0.08	0.95	0.59	0.28	0.86	0.08	0.94	0.59	0.45	0.78	0.08	0.90	0.62
Collinsella	-1.97	0.01	0.09	0.04	0.35	-1.24	0.09	0.12	0.31	0.48	-1.11	0.15	0.17	0.60	0.62	-1.11	0.15	0.17	0.60	0.62	-1.04	0.18	0.20	0.63	0.63
Coprobacter	0.33	0.73	0.69	0.86	0.84	0.06	0.94	0.63	0.96	0.83	-0.65	0.50	0.97	0.84	0.99	-0.66	0.49	0.97	0.84	1.00	-0.67	0.49	0.95	0.80	0.97
Coprococcus	0.28	0.76	0.13	0.88	0.43	0.55	0.53	0.29	0.76	0.63	0.60	0.51	0.46	0.84	0.87	0.60	0.51	0.46	0.84	0.87	0.65	0.49	0.46	0.80	0.83
Defluviitaleaceae_UCG.011	4.83	0.00	0.03	0.01	0.27	4.35	0.00	0.03	0.03	0.31	3.45	0.02	0.10	0.14	0.59	3.47	0.01	0.10	0.14	0.59	3.54	0.01	0.08	0.13	0.62
Desulfovibrio	0.23	0.76	0.87	0.88	0.97	0.31	0.65	0.89	0.81	0.98	0.41	0.58	0.97	0.84	0.99	0.41	0.58	0.97	0.84	1.00	0.44	0.57	0.96	0.80	0.97
Dialister	-0.03	0.96	0.79	0.98	0.90	0.09	0.87	0.95	0.93	0.98	0.60	0.33	0.51	0.77	0.87	0.60	0.33	0.51	0.77	0.87	0.59	0.35	0.56	0.78	0.87
DNF00809	2.27	0.15	0.65	0.31	0.84	2.18	0.15	0.52	0.42	0.83	1.96	0.21	0.60	0.64	0.88	1.96	0.21	0.60	0.64	0.88	2.00	0.20	0.58	0.64	0.87
Dorea	-0.07	0.95	0.93	0.98	0.98	-0.03	0.98	0.77	0.98	0.91	0.38	0.72	0.88	0.87	0.99	0.38	0.72	0.88	0.86	0.99	0.38	0.72	0.85	0.87	0.96
DTU089	3.67	0.00	0.00	0.02	0.06	3.10	0.01	0.00	0.07	0.07	2.92	0.02	0.00	0.14	0.11	2.92	0.02	0.00	0.14	0.11	2.95	0.02	0.00	0.14	0.12
Eggerthella	1.13	0.32	0.30	0.53	0.60	0.68	0.53	0.46	0.76	0.80	1.51	0.19	0.28	0.60	0.70	1.52	0.19	0.27	0.61	0.70	1.48	0.21	0.34	0.64	0.73
Eisenbergiella	0.28	0.80	0.75	0.89	0.90	0.24	0.82	0.81	0.89	0.93	1.29	0.25	0.23	0.66	0.64	1.29	0.25	0.23	0.66	0.64	1.25	0.27	0.31	0.71	0.73
Erysipelatoclostridium	0.46	0.70	0.42	0.86	0.69	0.30	0.79	0.58	0.87	0.83	1.37	0.25	0.30	0.66	0.70	1.37	0.25	0.30	0.66	0.70	1.32	0.27	0.35	0.71	0.73
Erysipelotrichaceae_UCG.003	1.19	0.19	0.39	0.36	0.69	1.16	0.17	0.30	0.47	0.63	0.39	0.66	0.56	0.87	0.88	0.39	0.66	0.57	0.86	0.88	0.51	0.58	0.49	0.80	0.83
Escherichia.Shigella	-0.02	0.98	0.94	1.00	0.98	0.68	0.38	0.63	0.64	0.83	1.19	0.15	0.30	0.60	0.70	1.19	0.15	0.30	0.60	0.70	1.13	0.18	0.33	0.63	0.73
Faecalibacterium	1.92	0.20	0.91	0.37	0.97	0.88	0.53	0.67	0.76	0.86	0.24	0.87	0.57	0.95	0.88	0.24	0.87	0.57	0.95	0.88	0.39	0.79	0.62	0.90	0.87
Family_XIII_AD3011_group	3.47	0.00	0.16	0.02	0.45	2.64	0.02	0.22	0.10	0.58	1.95	0.10	0.39	0.58	0.81	1.97	0.10	0.39	0.58	0.80	2.10	0.08	0.36	0.48	0.73
Family_XIII_UCG.001	3.06	0.03	0.16	0.11	0.45	2.70	0.04	0.12	0.21	0.48	1.04	0.46	0.60	0.84	0.88	1.05	0.46	0.60	0.84	0.88	1.11	0.44	0.58	0.80	0.87
FD2005	-0.48	0.70	0.35	0.86	0.66	0.56	0.65	0.90	0.81	0.98	0.51	0.69	0.83	0.87	0.97	0.51	0.69	0.83	0.86	0.97	0.61	0.64	0.92	0.82	0.97
Flavonifractor	-0.14	0.90	0.30	0.95	0.60	-0.31	0.77	0.44	0.87	0.79	0.85	0.45	0.19	0.84	0.64	0.86	0.45	0.19	0.84	0.64	0.84	0.47	0.22	0.80	0.63
Fournierella	2.03	0.13	0.42	0.31	0.69	1.65	0.20	0.56	0.50	0.83	0.64	0.63	0.70	0.87	0.94	0.64	0.64	0.70	0.86	0.94	0.91	0.51	0.62	0.80	0.87
Frisingicoccus	0.77	0.44	0.03	0.64	0.27	0.37	0.70	0.05	0.84	0.36	0.57	0.58	0.04	0.84	0.42	0.56	0.58	0.04	0.84	0.42	0.58	0.58	0.05	0.80	0.49
Fusicatenibacter	0.62	0.50	0.61	0.67	0.84	0.34	0.70	0.55	0.84	0.83	0.10	0.91	0.62	0.95	0.88	0.10	0.91	0.62	0.95	0.88	0.24	0.80	0.55	0.91	0.87
Fusobacterium	-2.09	0.05	0.02	0.16	0.17	-1.52	0.14	0.02	0.42	0.23	-0.45	0.68	0.11	0.87	0.59	-0.45	0.68	0.11	0.86	0.59	-0.59	0.60	0.10	0.80	0.62
GCA.900066575	1.34	0.30	0.64	0.51	0.84	0.63	0.61	0.64	0.80	0.83	-0.48	0.71	0.89	0.87	0.99	-0.49	0.71	0.89	0.86	0.99	-0.40	0.76	0.78	0.90	0.95
Gordonibacter	3.29	0.02	0.00	0.07	0.08	2.23	0.09	0.02	0.31	0.23	1.83	0.18	0.04	0.60	0.42	1.82	0.19	0.04	0.61	0.42	1.81	0.20	0.04	0.63	0.44
Granulicatella	-2.17	0.15	0.12	0.31	0.42	-1.68	0.25	0.22	0.53	0.58	-1.79	0.24	0.17	0.66	0.62	-1.80	0.24	0.17	0.66	0.62	-1.77	0.25	0.15	0.71	0.63
Haemophilus	1.26	0.17	0.98	0.34	0.99	0.98	0.26	0.97	0.54	0.98	0.55	0.56	0.77	0.84	0.96	0.55	0.56	0.77	0.84	0.96	0.69	0.47	0.82	0.80	0.96
Harryflintia	1.43	0.31	0.33	0.52	0.65	1.27	0.34	0.29	0.60	0.63	0.79	0.58	0.54	0.84	0.88	0.78	0.58	0.54	0.84	0.88	0.83	0.56	0.45	0.80	0.83
Holdemanella	-0.61	0.48	0.15	0.67	0.45	-0.29	0.72	0.20	0.85	0.58	0.22	0.80	0.62	0.92	0.88	0.22	0.81	0.61	0.92	0.88	0.20	0.82	0.60	0.91	0.87
Holdemania	3.59	0.14	0.18	0.31	0.46	3.62	0.12	0.15	0.39	0.56	3.27	0.18	0.37	0.60	0.80	3.27	0.18	0.37	0.61	0.80	3.27	0.19	0.32	0.63	0.73
Hungatella	-0.28	0.80	0.92	0.89	0.98	-0.18	0.87	0.91	0.93	0.98	0.91	0.41	0.59	0.84	0.88	0.92	0.41	0.58	0.84	0.88	0.91	0.42	0.68	0.80	0.91
Hydrogenoanaerobacterium	2.65	0.05	0.46	0.15	0.72	1.44	0.26	0.70	0.54	0.87	0.98	0.48	0.70	0.84	0.94	0.98	0.48	0.70	0.84	0.94	1.04	0.46	0.71	0.80	0.92
Incertae_Sedis	5.14	0.00	0.05	0.03	0.31	4.89	0.00	0.10	0.05	0.44	4.97	0.01	0.12	0.14	0.59	4.98	0.01	0.12	0.14	0.59	5.02	0.01	0.12	0.13	0.63
Intestinibacter	0.62	0.57	0.67	0.74	0.84	0.81	0.43	0.44	0.70	0.79	1.30	0.24	0.89	0.66	0.99	1.33	0.23	0.91	0.66	0.99	1.39	0.22	0.83	0.65	0.96
Intestinimonas	3.46	0.00	0.01	0.01	0.17	2.66	0.01	0.02	0.08	0.23	1.76	0.11	0.13	0.58	0.59	1.76	0.11	0.13	0.58	0.59	1.70	0.12	0.17	0.53	0.63
Lachnoclostridium	-6.07	0.01	0.24	0.03	0.53	-4.51	0.03	0.32	0.18	0.64	-2.03	0.35	0.61	0.78	0.88	-2.03	0.35	0.61	0.79	0.88	-2.20	0.32	0.58	0.76	0.87
Lachnospira	1.83	0.06	0.02	0.17	0.21	1.12	0.23	0.04	0.51	0.31	-0.08	0.94	0.01	0.97	0.14	-0.07	0.94	0.01	0.97	0.15	-0.05	0.96	0.01	0.97	0.16
Lachnospiraceae_FCS020_group	4.80	0.00	0.21	0.00	0.50	3.93	0.00	0.22	0.03	0.58	3.72	0.00	0.22	0.11	0.64	3.72	0.00	0.22	0.11	0.64	3.87	0.00	0.19	0.12	0.63
Lachnospiraceae_ND3007_group	0.64	0.43	0.53	0.64	0.78	0.37	0.63	0.32	0.80	0.64	-0.10	0.90	0.51	0.95	0.87	-0.10	0.90	0.51	0.95	0.87	0.05	0.95	0.42	0.97	0.81
Lachnospiraceae_NK4A136_group	1.99	0.06	0.43	0.17	0.69	1.05	0.30	0.53	0.55	0.83	0.01	0.99	0.79	0.99	0.96	0.01	0.99	0.79	0.99	0.96	0.11	0.92	0.75	0.96	0.94
Lachnospiraceae_UCG.001	1.88	0.02	0.68	0.10	0.84	1.52	0.06	0.80	0.23	0.93	1.05	0.22	0.89	0.66	0.99	1.06	0.22	0.89	0.66	0.99	1.16	0.18	0.94	0.63	0.97
Lachnospiraceae_UCG.004	0.84	0.37	0.41	0.57	0.69	0.57	0.52	0.40	0.76	0.76	0.33	0.72	0.41	0.87	0.81	0.33	0.72	0.41	0.86	0.81	0.33	0.72	0.46	0.87	0.83
Lachnospiraceae_UCG.010	-0.76	0.55	0.23	0.73	0.51	-1.30	0.28	0.23	0.55	0.59	-1.80	0.15	0.14	0.60	0.59	-1.81	0.15	0.14	0.60	0.59	-1.81	0.15	0.16	0.61	0.63
Lactobacillus	-0.33	0.72	0.05	0.86	0.31	0.25	0.77	0.09	0.87	0.44	-0.03	0.98	0.14	0.98	0.59	-0.03	0.97	0.14	0.98	0.59	0.03	0.97	0.18	0.97	0.63
Lactococcus	3.18	0.05	0.03	0.15	0.27	2.14	0.16	0.10	0.44	0.44	2.16	0.19	0.11	0.60	0.59	2.16	0.19	0.11	0.61	0.59	2.18	0.19	0.10	0.63	0.62
Marvinbryantia	1.38	0.11	0.66	0.29	0.84	1.05	0.21	0.62	0.50	0.83	0.43	0.64	0.93	0.87	0.99	0.43	0.64	0.93	0.86	0.99	0.48	0.60	0.90	0.80	0.97
Megasphaera	-0.43	0.49	0.54	0.67	0.79	0.15	0.81	0.58	0.88	0.83	0.52	0.42	0.78	0.84	0.96	0.52	0.42	0.78	0.84	0.96	0.48	0.46	0.74	0.80	0.94

© 2022 Meyer K et al. *JAMA Network Open.*

Monoglobus	3.19	0.00	0.15	0.02	0.45	2.60	0.01	0.24	0.07	0.59	1.40	0.18	0.79	0.60	0.96	1.40	0.18	0.79	0.61	0.96	1.58	0.14	0.72	0.56	0.93
Moryella	1.69	0.28	0.56	0.50	0.79	0.98	0.51	0.54	0.76	0.83	-0.31	0.85	0.38	0.95	0.81	-0.32	0.85	0.38	0.94	0.80	-0.25	0.88	0.39	0.93	0.77
Negativibacillus	0.99	0.28	0.02	0.51	0.17	1.31	0.14	0.00	0.42	0.07	1.37	0.14	0.01	0.60	0.11	1.37	0.14	0.01	0.60	0.11	1.48	0.12	0.00	0.53	0.12
NK4A214_group	0.78	0.30	0.97	0.51	0.99	0.43	0.55	0.96	0.78	0.98	-0.26	0.73	0.59	0.87	0.88	-0.27	0.72	0.58	0.86	0.88	-0.17	0.83	0.61	0.91	0.87
Odoribacter	0.94	0.17	0.20	0.34	0.49	0.42	0.52	0.51	0.76	0.83	0.23	0.74	0.39	0.87	0.81	0.23	0.74	0.39	0.87	0.80	0.29	0.68	0.32	0.85	0.73
Oscillibacter	3.63	0.00	0.06	0.02	0.31	3.48	0.00	0.03	0.04	0.25	3.43	0.01	0.03	0.14	0.39	3.45	0.01	0.03	0.14	0.39	3.62	0.00	0.03	0.13	0.32
Oscillospira	2.40	0.13	0.04	0.31	0.29	2.13	0.16	0.07	0.44	0.44	2.47	0.11	0.14	0.58	0.59	2.48	0.11	0.13	0.58	0.59	2.76	0.09	0.11	0.48	0.62
Oxalobacter	2.20	0.08	0.90	0.22	0.97	1.38	0.25	0.99	0.53	0.99	0.82	0.52	0.76	0.84	0.96	0.82	0.53	0.76	0.84	0.96	0.68	0.60	0.66	0.80	0.90
Paludicola	4.65	0.00	0.01	0.01	0.17	4.09	0.00	0.00	0.03	0.07	3.33	0.01	0.01	0.14	0.16	3.34	0.01	0.01	0.14	0.16	3.41	0.01	0.01	0.13	0.19
Parabacteroides	0.13	0.89	0.41	0.95	0.69	0.12	0.89	0.76	0.93	0.91	0.13	0.89	0.92	0.95	0.99	0.13	0.89	0.92	0.95	0.99	0.17	0.85	0.88	0.91	0.97
Paraprevotella	-0.75	0.25	0.79	0.46	0.90	-0.65	0.30	0.63	0.55	0.83	-0.51	0.45	0.76	0.84	0.96	-0.51	0.44	0.76	0.84	0.96	-0.44	0.51	0.81	0.80	0.96
Parasutterella	1.36	0.05	0.26	0.15	0.56	1.11	0.09	0.34	0.31	0.65	0.75	0.28	0.47	0.70	0.87	0.75	0.28	0.47	0.70	0.87	0.78	0.27	0.40	0.71	0.77
Peptococcus	2.28	0.10	0.45	0.27	0.72	1.41	0.29	0.58	0.55	0.83	0.95	0.50	0.75	0.84	0.96	0.95	0.50	0.75	0.84	0.96	1.00	0.48	0.79	0.80	0.96
Phascolarctobacterium	0.58	0.35	0.21	0.56	0.50	0.56	0.35	0.27	0.60	0.62	0.53	0.40	0.29	0.84	0.70	0.53	0.41	0.29	0.84	0.70	0.52	0.42	0.27	0.80	0.71
Phocea	0.03	0.99	0.77	1.00	0.90	-0.49	0.74	0.61	0.86	0.83	0.36	0.81	0.98	0.92	0.99	0.37	0.81	0.98	0.92	1.00	0.42	0.78	0.95	0.90	0.97
Prevotella	-0.83	0.13	0.98	0.31	0.99	-0.66	0.21	0.70	0.50	0.87	-0.63	0.25	0.81	0.67	0.97	-0.63	0.25	0.81	0.66	0.96	-0.59	0.29	0.85	0.73	0.96
Romboutsia	0.23	0.83	0.89	0.90	0.97	0.54	0.59	0.83	0.79	0.95	0.78	0.47	0.49	0.84	0.87	0.79	0.47	0.48	0.84	0.87	1.05	0.35	0.38	0.78	0.77
Roseburia	2.03	0.07	0.60	0.20	0.84	1.83	0.09	0.54	0.31	0.83	0.62	0.59	0.90	0.84	0.99	0.62	0.59	0.90	0.84	0.99	0.70	0.55	0.94	0.80	0.97
Ruminococcus	2.31	0.00	0.07	0.02	0.35	1.77	0.01	0.09	0.09	0.44	1.16	0.13	0.21	0.60	0.64	1.16	0.13	0.21	0.60	0.64	1.28	0.10	0.20	0.51	0.63
Sellimonas	0.78	0.48	0.85	0.67	0.96	0.60	0.57	0.97	0.78	0.98	0.51	0.64	0.99	0.87	0.99	0.51	0.65	0.99	0.86	1.00	0.52	0.64	0.98	0.82	0.98
Senegalimassilia	-0.74	0.44	0.09	0.64	0.35	-0.45	0.62	0.18	0.80	0.58	-1.07	0.26	0.17	0.67	0.62	-1.08	0.26	0.17	0.67	0.62	-1.00	0.30	0.19	0.73	0.63
Shuttleworthia	4.47	0.00	0.00	0.01	0.06	3.25	0.01	0.00	0.08	0.07	3.28	0.01	0.00	0.14	0.11	3.28	0.01	0.00	0.14	0.11	3.31	0.01	0.00	0.13	0.12
Slackia	-0.63	0.47	0.88	0.67	0.97	-0.62	0.45	0.89	0.72	0.98	-0.66	0.45	0.92	0.84	0.99	-0.66	0.45	0.92	0.84	0.99	-0.67	0.44	0.84	0.80	0.96
Streptococcus	2.71	0.04	0.06	0.13	0.31	2.49	0.05	0.08	0.21	0.44	2.01	0.13	0.15	0.60	0.61	2.03	0.12	0.15	0.60	0.59	2.22	0.10	0.16	0.51	0.63
Subdoligranulum	2.72	0.01	0.09	0.04	0.35	2.38	0.01	0.07	0.09	0.44	1.79	0.08	0.11	0.50	0.59	1.79	0.08	0.11	0.50	0.59	1.89	0.07	0.09	0.44	0.62
Sutterella	-1.67	0.01	0.18	0.03	0.46	-1.28	0.03	0.19	0.16	0.58	-1.25	0.04	0.24	0.31	0.65	-1.25	0.04	0.24	0.31	0.65	-1.22	0.05	0.27	0.34	0.71
TM7x	0.01	1.00	0.67	1.00	0.84	-0.16	0.91	0.96	0.94	0.98	-0.06	0.97	0.97	0.98	0.99	-0.06	0.97	0.97	0.98	1.00	0.08	0.95	0.92	0.97	0.97
Turicibacter	-1.42	0.35	0.27	0.56	0.56	-0.76	0.60	0.30	0.79	0.63	-0.73	0.65	0.41	0.87	0.81	-0.72	0.65	0.41	0.86	0.81	-0.69	0.67	0.48	0.84	0.83
Tuzzerella	0.32	0.77	0.05	0.88	0.31	0.29	0.78	0.07	0.87	0.44	0.43	0.70	0.14	0.87	0.59	0.43	0.70	0.14	0.86	0.59	0.42	0.71	0.13	0.87	0.63
UBA1819	1.32	0.33	0.17	0.54	0.45	1.02	0.43	0.20	0.70	0.58	1.80	0.19	0.14	0.60	0.59	1.79	0.19	0.14	0.61	0.59	1.71	0.22	0.16	0.65	0.63
UCG.002	1.50	0.03	0.20	0.12	0.49	1.00	0.14	0.25	0.42	0.59	0.74	0.30	0.19	0.72	0.64	0.74	0.30	0.19	0.73	0.64	0.78	0.28	0.17	0.72	0.63
UCG.003	1.01	0.22	1.00	0.41	1.00	0.72	0.35	0.73	0.60	0.88	0.32	0.70	0.80	0.87	0.96	0.32	0.70	0.80	0.86	0.96	0.43	0.61	0.69	0.80	0.91
UCG.005	2.32	0.01	0.13	0.04	0.42	1.65	0.05	0.25	0.21	0.59	0.63	0.48	0.53	0.84	0.88	0.63	0.48	0.53	0.84	0.88	0.78	0.39	0.46	0.80	0.83
UCG.009	2.32	0.10	0.49	0.26	0.74	1.40	0.30	0.52	0.55	0.83	0.17	0.90	0.94	0.95	0.99	0.17	0.90	0.93	0.95	0.99	0.28	0.84	0.90	0.91	0.97
Veillonella	0.77	0.41	0.72	0.63	0.87	0.91	0.31	0.92	0.56	0.98	0.40	0.67	0.59	0.87	0.88	0.41	0.67	0.60	0.86	0.88	0.54	0.58	0.64	0.80	0.87
Victivallis	1.49	0.13	0.55	0.31	0.79	1.24	0.19	0.71	0.49	0.87	0.56	0.59	0.42	0.84	0.81	0.56	0.58	0.42	0.84	0.81	0.53	0.61	0.34	0.80	0.73

Multivariable-adjusted regression models adjusted for:

Model 1: sequencing batch, age, race, gender, study center

Model 2: Model 1, with education

Model 3: Model 2, with physical activity, diet score, medication use, and smoking

Model 4: Model 3 with BMI

Model 5: Model 4 with hypertension and diabetes

© 2022 Meyer K et al. *JAMA Network Open.*

eTable 20: Multivariable-adjusted regression coefficients, p-values, race interaction p-values, false discovery rate (FDR)-adjusted p-values for race-genera interactions with respect to Stroop.

	Model1					Model2					Model3					Model4					Model5				
	beta	p-value	p-val int	FDR	FDR-int	beta	p-value	p-val int	FDR	FDR-int	beta	p-value	p-val int	FDR	FDR-int	beta	p-value	p-val int	FDR	FDR-int	beta	p-value	p-val int	FDR	FDR-int
Acetanaerobacterium	-0.89	0.53	0.37	0.76	0.68	-0.88	0.53	0.49	0.79	0.78	-0.21	0.89	0.72	0.99	0.87	-0.20	0.89	0.71	0.99	0.89	-0.24	0.87	0.73	0.96	0.91
Acidaminococcus	0.36	0.39	0.84	0.64	0.91	0.24	0.56	0.95	0.79	0.96	-0.47	0.29	0.18	0.76	0.60	-0.48	0.29	0.18	0.76	0.60	-0.49	0.28	0.17	0.70	0.59
Actinomyces	-0.09	0.94	0.85	0.94	0.91	-0.30	0.79	0.93	0.91	0.96	-1.27	0.27	0.84	0.76	0.94	-1.27	0.27	0.84	0.76	0.94	-1.32	0.26	0.79	0.70	0.91
Adlercreutzia	-0.19	0.80	0.60	0.84	0.82	-0.04	0.96	0.75	0.97	0.92	-0.02	0.98	0.69	0.99	0.87	-0.01	0.98	0.68	1.00	0.89	-0.03	0.97	0.68	0.97	0.88
Agathobacter	-0.69	0.47	0.71	0.69	0.85	-0.61	0.52	0.74	0.79	0.92	0.28	0.77	0.95	0.98	0.98	0.26	0.79	0.95	0.99	0.97	0.10	0.92	0.91	0.96	1.00
Akkermansia	-1.22	0.01	0.04	0.10	0.30	-1.05	0.03	0.05	0.22	0.36	-0.82	0.11	0.14	0.62	0.60	-0.82	0.11	0.14	0.63	0.59	-0.82	0.12	0.14	0.56	0.58
Alistipes	-0.36	0.67	0.99	0.82	0.99	-0.16	0.85	0.89	0.91	0.96	0.27	0.75	0.73	0.98	0.87	0.26	0.76	0.74	0.99	0.89	0.23	0.78	0.76	0.96	0.91
Allisonella	1.29	0.11	0.58	0.32	0.82	0.85	0.29	0.67	0.62	0.90	0.50	0.56	0.92	0.98	0.97	0.50	0.56	0.94	0.98	0.97	0.56	0.53	0.96	0.91	1.00
Anaerofilum	-1.41	0.21	0.09	0.46	0.41	-0.98	0.38	0.11	0.68	0.44	-1.14	0.32	0.08	0.76	0.60	-1.13	0.33	0.08	0.76	0.59	-1.21	0.30	0.07	0.72	0.57
Anaerostipes	0.30	0.77	0.67	0.84	0.85	0.51	0.61	0.63	0.81	0.87	0.93	0.35	0.48	0.79	0.77	0.92	0.36	0.49	0.79	0.78	0.79	0.45	0.57	0.86	0.84
Anaerotruncus	-1.78	0.05	0.01	0.22	0.20	-1.66	0.07	0.01	0.29	0.21	-1.23	0.19	0.03	0.71	0.35	-1.20	0.20	0.03	0.75	0.33	-1.34	0.16	0.02	0.61	0.29
Bacteroides	-2.49	0.07	0.06	0.25	0.36	-2.31	0.10	0.06	0.32	0.39	-2.24	0.10	0.04	0.62	0.49	-2.30	0.09	0.04	0.62	0.46	-2.19	0.11	0.05	0.56	0.47
Barnesiella	-0.38	0.43	0.49	0.66	0.73	-0.42	0.38	0.55	0.68	0.83	-0.27	0.58	0.41	0.98	0.77	-0.27	0.59	0.41	0.98	0.78	-0.33	0.51	0.46	0.90	0.76
Bifidobacterium	0.36	0.57	0.18	0.76	0.51	0.24	0.71	0.14	0.87	0.49	0.08	0.91	0.33	0.99	0.73	0.06	0.93	0.34	1.00	0.72	0.07	0.92	0.34	0.96	0.75
Bilophila	-0.36	0.57	0.81	0.76	0.91	-0.13	0.83	0.59	0.91	0.85	-0.10	0.87	0.72	0.98	0.87	-0.12	0.85	0.73	0.99	0.89	-0.07	0.91	0.70	0.96	0.90
Blautia	-3.89	0.05	0.03	0.22	0.30	-3.70	0.06	0.03	0.29	0.27	-4.04	0.05	0.02	0.51	0.35	-4.09	0.04	0.02	0.51	0.33	-5.33	0.01	0.01	0.40	0.29
Butyricicoccus	-3.58	0.01	0.00	0.07	0.12	-3.04	0.03	0.00	0.19	0.11	-1.61	0.25	0.02	0.76	0.35	-1.64	0.24	0.02	0.76	0.33	-1.75	0.21	0.02	0.65	0.29
Butyricimonas	-0.88	0.09	0.59	0.28	0.82	-0.80	0.12	0.59	0.37	0.85	-0.83	0.12	0.33	0.62	0.73	-0.83	0.12	0.33	0.64	0.72	-0.85	0.11	0.32	0.56	0.75
CAG.56	-0.29	0.64	0.48	0.81	0.73	-0.26	0.67	0.60	0.85	0.86	0.53	0.39	0.18	0.79	0.60	0.53	0.40	0.18	0.79	0.60	0.45	0.48	0.22	0.87	0.66
Candidatus_Soleaferrea	-1.50	0.23	0.06	0.50	0.36	-1.40	0.26	0.05	0.60	0.36	-2.19	0.08	0.02	0.62	0.35	-2.19	0.08	0.02	0.62	0.33	-2.27	0.08	0.02	0.53	0.29
Caproiciproducens	-1.10	0.31	0.68	0.55	0.85	-1.00	0.34	0.76	0.65	0.92	-0.30	0.79	0.90	0.98	0.97	-0.29	0.79	0.90	0.99	0.97	-0.36	0.75	0.92	0.96	1.00
Christensenellaceae_R.7_group	-1.66	0.00	0.19	0.03	0.51	-1.46	0.01	0.27	0.06	0.60	-0.81	0.14	0.88	0.64	0.96	-0.80	0.14	0.88	0.64	0.96	-0.92	0.10	0.76	0.56	0.91
Clostridium_sensu_stricto_1	0.84	0.24	0.26	0.50	0.60	0.72	0.31	0.31	0.62	0.63	1.09	0.14	0.14	0.64	0.60	1.08	0.14	0.14	0.64	0.59	1.12	0.13	0.13	0.57	0.58
Colidextribacter	-0.71	0.54	0.35	0.76	0.66	-0.68	0.56	0.25	0.79	0.60	-1.19	0.31	0.16	0.76	0.60	-1.20	0.30	0.15	0.76	0.59	-1.61	0.18	0.10	0.64	0.58
Collinsella	0.13	0.81	0.84	0.84	0.91	-0.13	0.82	0.92	0.91	0.96	0.09	0.87	0.72	0.98	0.87	0.09	0.87	0.70	0.99	0.89	0.16	0.79	0.64	0.96	0.88
Coprobacter	-0.77	0.27	0.13	0.53	0.48	-0.68	0.33	0.12	0.63	0.46	-0.14	0.85	0.31	0.98	0.73	-0.12	0.86	0.32	0.99	0.72	-0.11	0.87	0.33	0.96	0.75
Coprococcus	0.18	0.79	0.48	0.84	0.73	0.09	0.90	0.62	0.93	0.86	-0.05	0.94	0.90	0.99	0.97	-0.05	0.94	0.90	1.00	0.97	-0.15	0.83	0.99	0.96	1.00
Defluviitaleaceae_UCG.011	-3.76	0.00	0.02	0.01	0.21	-3.61	0.00	0.02	0.02	0.22	-2.74	0.01	0.12	0.49	0.60	-2.73	0.01	0.12	0.51	0.59	-2.97	0.01	0.10	0.29	0.58
Desulfovibrio	-0.08	0.88	0.81	0.89	0.91	-0.12	0.83	0.83	0.91	0.95	-0.09	0.87	0.93	0.98	0.97	-0.07	0.90	0.92	0.99	0.97	-0.18	0.75	0.98	0.96	1.00
Dialister	0.36	0.42	0.62	0.65	0.82	0.31	0.49	0.71	0.79	0.91	0.40	0.39	0.66	0.79	0.87	0.40	0.39	0.66	0.79	0.89	0.39	0.41	0.68	0.85	0.88
DNF00809	-1.98	0.08	0.27	0.27	0.60	-1.96	0.08	0.23	0.31	0.60	-2.10	0.07	0.27	0.62	0.73	-2.10	0.07	0.27	0.62	0.72	-2.13	0.07	0.27	0.53	0.73
Dorea	-1.44	0.06	0.30	0.23	0.63	-1.45	0.06	0.33	0.29	0.64	-1.56	0.04	0.24	0.51	0.70	-1.56	0.04	0.25	0.51	0.71	-1.50	0.05	0.27	0.53	0.73
DTU089	-1.39	0.12	0.07	0.33	0.36	-1.22	0.17	0.10	0.48	0.43	-1.00	0.27	0.17	0.76	0.60	-1.00	0.27	0.17	0.76	0.59	-1.19	0.20	0.13	0.65	0.58
Eggerthella	0.34	0.69	0.65	0.82	0.84	0.51	0.54	0.77	0.79	0.92	-0.38	0.66	0.30	0.98	0.73	-0.39	0.65	0.28	0.99	0.72	-0.43	0.63	0.27	0.95	0.73
Eisenbergiella	0.29	0.72	0.33	0.83	0.64	0.30	0.71	0.31	0.87	0.63	-0.46	0.58	0.81	0.98	0.94	-0.45	0.59	0.82	0.98	0.94	-0.49	0.56	0.86	0.91	0.97
Erysipelatoclostridium	0.19	0.83	0.70	0.84	0.85	0.23	0.79	0.62	0.91	0.86	-0.36	0.68	0.96	0.98	0.98	-0.36	0.69	0.95	0.99	0.97	-0.36	0.69	0.95	0.96	1.00
Erysipelotrichaceae_UCG.003	-0.55	0.40	0.94	0.64	0.97	-0.54	0.41	0.99	0.71	0.99	0.12	0.86	0.37	0.98	0.76	0.13	0.85	0.37	0.99	0.74	0.04	0.95	0.43	0.97	0.75
Escherichia.Shigella	0.25	0.67	0.93	0.82	0.97	0.01	0.99	0.86	0.99	0.96	-0.32	0.60	0.50	0.98	0.77	-0.33	0.59	0.49	0.98	0.78	-0.30	0.63	0.51	0.95	0.77
Faecalibacterium	-1.06	0.33	0.32	0.56	0.63	-0.70	0.51	0.21	0.79	0.59	-0.32	0.76	0.15	0.98	0.60	-0.34	0.75	0.16	0.99	0.59	-0.55	0.61	0.22	0.95	0.66
Family_XIII_AD3011_group	-2.27	0.01	0.03	0.05	0.28	-1.98	0.02	0.04	0.14	0.32	-0.94	0.28	0.19	0.76	0.60	-0.92	0.30	0.19	0.76	0.60	-1.06	0.24	0.16	0.67	0.59
Family_XIII_UCG.001	-2.19	0.03	0.10	0.19	0.42	-2.05	0.05	0.09	0.26	0.43	-0.97	0.36	0.53	0.79	0.77	-0.94	0.38	0.53	0.79	0.78	-1.08	0.32	0.49	0.75	0.76
FD2005	-0.25	0.79	0.58	0.84	0.82	-0.60	0.52	0.85	0.79	0.96	0.28	0.77	0.34	0.98	0.74	0.28	0.76	0.33	0.99	0.72	0.16	0.87	0.37	0.96	0.75
Flavonifractor	0.60	0.46	0.60	0.68	0.82	0.65	0.42	0.51	0.71	0.79	-0.42	0.62	0.84	0.98	0.94	-0.43	0.61	0.82	0.98	0.94	-0.51	0.56	0.78	0.91	0.91
Fournierella	-1.28	0.19	0.22	0.45	0.52	-1.15	0.24	0.26	0.58	0.60	-0.22	0.83	0.61	0.98	0.87	-0.19	0.85	0.62	0.99	0.88	-0.44	0.67	0.49	0.96	0.76
Frisingicoccus	-0.52	0.48	0.13	0.70	0.48	-0.40	0.58	0.15	0.80	0.49	-0.32	0.67	0.15	0.98	0.60	-0.31	0.68	0.14	0.99	0.59	-0.31	0.69	0.14	0.96	0.58
Fusicatenibacter	-0.88	0.19	0.48	0.45	0.73	-0.77	0.25	0.47	0.58	0.77	-0.69	0.33	0.51	0.76	0.77	-0.71	0.31	0.51	0.76	0.78	-0.78	0.28	0.45	0.70	0.76
Fusobacterium	1.58	0.05	0.52	0.21	0.77	1.37	0.08	0.59	0.31	0.85	0.71	0.38	0.58	0.79	0.83	0.73	0.37	0.58	0.79	0.83	0.66	0.43	0.56	0.85	0.83
GCA.900066575	-2.08	0.03	0.28	0.16	0.62	-1.84	0.05	0.28	0.26	0.61	-1.12	0.24	0.51	0.76	0.77	-1.11	0.25	0.51	0.76	0.78	-1.23	0.21	0.49	0.65	0.76
Gordonibacter	-0.85	0.40	0.20	0.64	0.52	-0.49	0.63	0.32	0.82	0.63	-0.18	0.86	0.38	0.98	0.76	-0.15	0.89	0.38	0.99	0.75	-0.16	0.88	0.38	0.96	0.75
Granulicatella	0.40	0.72	0.47	0.83	0.73	0.21	0.85	0.59	0.91	0.85	-0.34	0.77	0.72	0.98	0.87	-0.37	0.75	0.74	0.99	0.89	-0.37	0.75	0.76	0.96	0.91
Haemophilus	-0.29	0.67	0.65	0.82	0.84	-0.20	0.76	0.65	0.91	0.88	0.01	0.99	0.52	0.99	0.77	0.00	1.00	0.53	1.00	0.78	-0.09	0.90	0.60	0.96	0.86
Harryflintia	-2.42	0.02	0.00	0.11	0.13	-2.40	0.02	0.00	0.14	0.11	-1.73	0.10	0.02	0.62	0.35	-1.71	0.11	0.02	0.63	0.33	-1.88	0.08	0.02	0.53	0.29
Holdemanella	0.24	0.70	0.61	0.83	0.82	0.13	0.84	0.69	0.91	0.90	0.29	0.66	0.78	0.98	0.91	0.29	0.66	0.76	0.99	0.91	0.31	0.65	0.77	0.95	0.91
Holdemania	-2.47	0.17	0.10	0.43	0.42	-2.48	0.16	0.10	0.46	0.43	-2.66	0.14	0.15	0.64	0.60	-2.64	0.15	0.14	0.64	0.59	-2.72	0.14	0.15	0.57	0.58
Hungatella	1.37	0.09	0.39	0.28	0.69	1.34	0.09	0.35	0.32	0.64	0.24	0.77	1.00	0.98	1.00	0.23	0.78	0.98	0.99	0.99	0.23	0.79	0.96	0.96	1.00
Hydrogenoanaerobacterium	-0.98	0.32	0.57	0.56	0.82	-0.57	0.56	0.46	0.79	0.77	-0.16	0.87	0.32	0.98	0.73	-0.15	0.89	0.32	0.99	0.72	-0.15	0.88	0.32	0.96	0.75
Incertae_Sedis	-1.54	0.24	0.40	0.50	0.69	-1.49	0.25	0.48	0.59	0.77	-1.53	0.25	0.51	0.76	0.77	-1.55	0.25	0.49	0.76	0.78	-1.66	0.22	0.48	0.65	0.76
Intestinibacter	0.26	0.74	0.14	0.84	0.48	0.21	0.79	0.10	0.91	0.43	-0.09	0.92	0.30	0.99	0.73	-0.13	0.88	0.32	0.99	0.72	-0.13	0.88	0.32	0.96	0.75
Intestinimonas	-1.90	0.01	0.04	0.10	0.30	-1.63	0.03	0.05	0.22	0.36	-1.25	0.12	0.15	0.62	0.60	-1.24	0.13	0.14	0.64	0.59	-1.33	0.10	0.12	0.56	0.58
Lachnoclostridium	4.39	0.01	0.19	0.05	0.51	3.88	0.02	0.22	0.14	0.59	2.61	0.10	0.47	0.62	0.77	2.60	0.11	0.47	0.63	0.78	2.81	0.08	0.43	0.53	0.75
Lachnospira	-0.97	0.17	0.14	0.43	0.48	-0.74	0.29	0.17	0.62	0.53	-0.23	0.76	0.10	0.98	0.60	-0.24	0.74	0.11	0.99	0.59	-0.45	0.56	0.17	0.91	0.59
Lachnospiraceae_FCS020_group	-2.69	0.00	0.07	0.03	0.36	-2.39	0.01	0.07	0.06	0.40	-1.12	0.21	0.47	0.76	0.77	-1.12	0.21	0.46	0.76	0.78	-1.16	0.20	0.44	0.65	0.75
Lachnospiraceae_ND3007_group	-0.79	0.17	0.25	0.43	0.59	-0.70	0.22	0.19	0.57	0.55	-0.22	0.71	0.45	0.98	0.77	-0.24	0.69	0.45	0.99	0.78	-0.36	0.56	0.37	0.91	0.75
Lachnospiraceae_NK4A136_group	0.17	0.82	0.75	0.84	0.88	0.51	0.51	0.68	0.79	0.90	1.59	0.05	0.32	0.51	0.73	1.58	0.05	0.33	0.52	0.72	1.46	0.08	0.39	0.53	0.75
Lachnospiraceae_UCG.001	-0.94	0.13	0.43	0.35	0.70	-0.83	0.17	0.47	0.48	0.77	0.14	0.83	0.68	0.98	0.87	0.16	0.81	0.67	0.99	0.89	0.08	0.91	0.74	0.96	0.91
Lachnospiraceae_UCG.004	-0.28	0.68	0.83	0.82	0.91	-0.17	0.80	0.82	0.91	0.94	0.02	0.98	0.84	0.99	0.94	0.01	0.99	0.84	1.00	0.94	0.11	0.88	0.95	0.96	1.00
Lachnospiraceae_UCG.010	-0.72	0.44	0.43	0.66	0.70	-0.53	0.57	0.40	0.79	0.69	0.32	0.73	0.72	0.98	0.87	0.35	0.71	0.73	0.99	0.89	0.37	0.70	0.73	0.96	0.91
Lactobacillus	0.16	0.81	0.76	0.84	0.88	-0.05	0.94	0.88	0.96	0.96	-0.16	0.82	0.79	0.98	0.92	-0.18	0.80	0.79	0.99	0.91	-0.09	0.90	0.80	0.96	0.91
Lactococcus	-1.85	0.11	0.08	0.33	0.38	-1.49	0.20	0.14	0.53	0.49	-0.61	0.62	0.35	0.98	0.74	-0.62	0.61	0.34	0.98	0.72	-0.36	0.77	0.43	0.96	0.75
Marvinbryantia	-0.69	0.28	0.14	0.53	0.48	-0.56	0.38	0.13	0.68	0.49	0.02	0.98	0.44	0.99	0.77	0.02	0.98	0.44	1.00	0.78	-0.10	0.88	0.36	0.96	0.75
Megasphaera	0.28	0.54	0.76	0.76	0.88	0.08	0.87	0.80	0.92	0.92	-0.49	0.30	0.76	0.76	0.90	-0.49	0.31	0.78	0.76	0.91	-0.39	0.43	0.88	0.85	0.98

© 2022 Meyer K et al. *JAMA Network Open.*

Monoglobus	-2.21	0.00	0.30	0.03	0.63	-2.03	0.01	0.37	0.06	0.66	-0.23	0.77	0.33	0.98	0.73	-0.23	0.77	0.33	0.99	0.72	-0.37	0.64	0.40	0.95	0.75
Moryella	-2.32	0.04	0.16	0.21	0.49	-2.04	0.08	0.17	0.30	0.53	-0.42	0.73	0.69	0.98	0.87	-0.41	0.74	0.69	0.99	0.89	-0.58	0.64	0.62	0.95	0.87
Negativibacillus	-0.86	0.20	0.17	0.45	0.51	-0.98	0.14	0.12	0.43	0.47	-1.22	0.08	0.11	0.62	0.60	-1.21	0.08	0.11	0.62	0.59	-1.24	0.08	0.11	0.53	0.58
NK4A214_group	-1.52	0.01	0.02	0.05	0.23	-1.40	0.01	0.02	0.10	0.22	-0.74	0.19	0.14	0.71	0.60	-0.73	0.20	0.15	0.75	0.59	-0.85	0.14	0.11	0.57	0.58
Odoribacter	-0.51	0.30	0.14	0.55	0.48	-0.33	0.51	0.26	0.79	0.60	-0.38	0.46	0.11	0.87	0.60	-0.38	0.46	0.11	0.87	0.59	-0.49	0.34	0.09	0.78	0.58
Oscillibacter	-1.65	0.07	0.39	0.23	0.69	-1.61	0.07	0.32	0.29	0.63	-1.28	0.17	0.50	0.67	0.77	-1.27	0.17	0.50	0.69	0.78	-1.32	0.17	0.49	0.61	0.76
Oscillospira	-1.25	0.28	0.42	0.53	0.70	-1.19	0.30	0.49	0.62	0.78	-1.38	0.23	0.51	0.76	0.77	-1.39	0.23	0.50	0.76	0.78	-1.75	0.14	0.39	0.57	0.75
Oxalobacter	-1.06	0.25	0.80	0.50	0.91	-0.78	0.40	0.76	0.69	0.92	0.71	0.46	0.40	0.87	0.77	0.72	0.46	0.40	0.87	0.77	0.70	0.48	0.43	0.87	0.75
Paludicola	-2.92	0.00	0.01	0.03	0.20	-2.74	0.00	0.01	0.06	0.18	-1.77	0.08	0.06	0.62	0.59	-1.75	0.08	0.06	0.62	0.59	-1.83	0.07	0.05	0.53	0.47
Parabacteroides	-0.28	0.68	0.72	0.82	0.86	-0.27	0.69	0.92	0.87	0.96	-0.21	0.76	0.93	0.98	0.97	-0.22	0.74	0.94	0.99	0.97	-0.26	0.70	0.98	0.96	1.00
Paraprevotella	0.88	0.07	0.06	0.23	0.36	0.85	0.07	0.05	0.29	0.36	0.84	0.09	0.07	0.62	0.60	0.85	0.09	0.07	0.62	0.59	0.90	0.08	0.06	0.53	0.47
Parasutterella	-1.47	0.00	0.08	0.03	0.38	-1.38	0.00	0.09	0.06	0.43	-1.05	0.04	0.16	0.51	0.60	-1.06	0.04	0.16	0.51	0.59	-1.12	0.03	0.14	0.49	0.58
Peptococcus	-3.11	0.00	0.00	0.03	0.10	-2.83	0.00	0.00	0.06	0.11	-2.22	0.03	0.01	0.51	0.28	-2.21	0.03	0.01	0.51	0.29	-2.21	0.04	0.01	0.49	0.27
Phascolarctobacterium	-0.55	0.23	0.70	0.50	0.85	-0.53	0.24	0.79	0.58	0.92	-0.57	0.23	0.66	0.76	0.87	-0.57	0.23	0.66	0.76	0.89	-0.59	0.22	0.65	0.65	0.88
Phocea	1.03	0.35	0.99	0.58	0.99	1.20	0.27	0.93	0.61	0.96	0.96	0.38	0.72	0.79	0.87	0.95	0.39	0.72	0.79	0.89	0.86	0.44	0.68	0.86	0.88
Prevotella	0.83	0.04	0.32	0.21	0.63	0.76	0.06	0.24	0.29	0.60	0.58	0.16	0.38	0.66	0.76	0.59	0.16	0.36	0.64	0.74	0.52	0.22	0.43	0.65	0.75
Romboutsia	0.37	0.63	0.19	0.81	0.51	0.25	0.75	0.20	0.91	0.55	0.04	0.96	0.31	0.99	0.73	0.02	0.98	0.33	1.00	0.72	-0.04	0.97	0.36	0.97	0.75
Roseburia	-0.48	0.56	0.91	0.76	0.96	-0.42	0.61	0.88	0.81	0.96	0.05	0.96	0.86	0.99	0.95	0.04	0.96	0.86	1.00	0.95	-0.22	0.80	0.95	0.96	1.00
Ruminococcus	-1.10	0.04	0.07	0.21	0.36	-0.93	0.09	0.08	0.31	0.42	-0.40	0.49	0.28	0.88	0.73	-0.39	0.49	0.28	0.89	0.72	-0.48	0.41	0.22	0.85	0.66
Sellimonas	-0.21	0.80	0.70	0.84	0.85	-0.16	0.84	0.75	0.91	0.92	-0.54	0.51	0.99	0.91	1.00	-0.53	0.53	1.00	0.94	1.00	-0.55	0.51	1.00	0.90	1.00
Senegalimassilia	0.21	0.77	0.27	0.84	0.60	0.10	0.88	0.35	0.92	0.64	0.66	0.36	0.14	0.79	0.60	0.68	0.34	0.14	0.78	0.59	0.62	0.39	0.15	0.84	0.58
Shuttleworthia	-3.06	0.00	0.01	0.03	0.16	-2.68	0.00	0.01	0.06	0.18	-2.28	0.02	0.02	0.51	0.35	-2.27	0.02	0.02	0.51	0.33	-2.26	0.02	0.02	0.40	0.29
Slackia	-1.28	0.05	0.15	0.21	0.49	-1.28	0.04	0.19	0.25	0.55	-0.94	0.15	0.23	0.64	0.69	-0.94	0.15	0.24	0.64	0.70	-0.96	0.14	0.24	0.57	0.68
Streptococcus	-1.06	0.27	0.99	0.53	0.99	-1.00	0.29	0.94	0.62	0.96	-0.97	0.32	0.68	0.76	0.87	-0.98	0.32	0.71	0.76	0.89	-0.87	0.39	0.66	0.84	0.88
Subdoligranulum	-2.80	0.00	0.00	0.01	0.10	-2.69	0.00	0.00	0.02	0.09	-2.72	0.00	0.00	0.03	0.09	-2.71	0.00	0.00	0.03	0.09	-2.93	0.00	0.00	0.01	0.05
Sutterella	0.85	0.05	0.07	0.22	0.36	0.72	0.10	0.07	0.32	0.40	0.38	0.41	0.19	0.80	0.60	0.38	0.41	0.19	0.79	0.60	0.41	0.37	0.18	0.83	0.59
TM7x	-1.01	0.33	0.42	0.56	0.70	-0.97	0.34	0.32	0.65	0.63	-0.88	0.40	0.43	0.79	0.77	-0.90	0.39	0.42	0.79	0.78	-1.03	0.33	0.36	0.77	0.75
Turicibacter	0.33	0.77	0.36	0.84	0.68	0.08	0.94	0.39	0.96	0.69	-0.58	0.63	0.65	0.98	0.87	-0.60	0.61	0.67	0.98	0.89	-0.59	0.63	0.64	0.95	0.88
Tuzzerella	0.39	0.62	0.65	0.81	0.84	0.38	0.62	0.72	0.82	0.91	-0.07	0.93	0.43	0.99	0.77	-0.06	0.94	0.43	1.00	0.78	-0.05	0.96	0.44	0.97	0.75
UBA1819	-1.34	0.18	0.21	0.43	0.52	-1.25	0.20	0.22	0.53	0.59	-2.35	0.02	0.05	0.51	0.56	-2.34	0.02	0.05	0.51	0.56	-2.52	0.02	0.05	0.40	0.47
UCG.002	-0.69	0.17	0.16	0.43	0.49	-0.52	0.31	0.18	0.62	0.54	-0.04	0.95	0.35	0.99	0.74	-0.02	0.97	0.36	1.00	0.74	-0.13	0.81	0.29	0.96	0.75
UCG.003	-1.07	0.07	0.37	0.25	0.68	-0.97	0.10	0.30	0.32	0.63	-0.68	0.26	0.43	0.76	0.77	-0.68	0.27	0.43	0.76	0.78	-0.67	0.28	0.43	0.70	0.75
UCG.005	-2.31	0.00	0.01	0.01	0.17	-2.10	0.00	0.02	0.03	0.21	-1.37	0.04	0.08	0.51	0.60	-1.36	0.04	0.08	0.51	0.59	-1.56	0.02	0.06	0.40	0.47
UCG.009	-1.00	0.34	0.87	0.57	0.92	-0.69	0.51	0.90	0.79	0.96	0.31	0.77	0.52	0.98	0.77	0.30	0.78	0.53	0.99	0.78	0.18	0.86	0.58	0.96	0.84
Veillonella	0.56	0.42	0.21	0.65	0.52	0.49	0.47	0.26	0.79	0.60	0.72	0.31	0.20	0.76	0.61	0.71	0.32	0.21	0.76	0.63	0.80	0.28	0.19	0.70	0.61
Victivallis	-0.40	0.58	0.30	0.77	0.63	-0.32	0.66	0.35	0.85	0.64	0.53	0.49	0.11	0.88	0.60	0.54	0.48	0.11	0.89	0.59	0.55	0.48	0.11	0.87	0.58

Multivariable-adjusted regression models adjusted for:

Model 1: sequencing batch, age, race, gender, study center

Model 2: Model 1, with education

Model 3: Model 2, with physical activity, diet score, medication use, and smoking

Model 4: Model 3 with BMI

Model 5: Model 4 with hypertension and diabetes

© 2022 Meyer K et al. *JAMA Network Open.*

eTable 21: Multivariable-adjusted regression coefficients, p-values, race interaction p-values, false discovery rate (FDR)-adjusted p-values for race-genera interactions with respect to RAVLT.

	Model1					Model2					Model3					Model4					Model5				
	beta	p-value	p-val int	FDR	FDR-int	beta	p-value	p-val int	FDR	FDR-int	beta	p-value	p-val int	FDR	FDR-int	beta	p-value	p-val int	FDR	FDR-int	beta	p-value	p-val int	FDR	FDR-int
Acetanaerobacterium	-0.03	0.89	0.93	0.94	0.97	-0.03	0.89	0.71	1.00	0.92	-0.12	0.61	0.90	0.94	0.97	-0.12	0.60	0.91	0.93	0.97	-0.12	0.60	0.97	0.92	0.98
Acidaminococcus	-0.12	0.06	0.45	0.52	0.76	-0.09	0.15	0.65	0.83	0.89	-0.03	0.62	0.26	0.94	0.67	-0.03	0.64	0.26	0.94	0.68	-0.03	0.63	0.27	0.92	0.67
Actinomyces	-0.23	0.18	0.28	0.71	0.70	-0.18	0.28	0.33	0.83	0.71	-0.14	0.43	0.40	0.91	0.73	-0.14	0.43	0.41	0.90	0.73	-0.15	0.42	0.36	0.91	0.74
Adlercreutzia	0.20	0.09	0.83	0.68	0.94	0.15	0.20	0.85	0.83	0.94	0.11	0.38	0.77	0.91	0.95	0.11	0.38	0.77	0.89	0.94	0.10	0.41	0.77	0.91	0.91
Agathobacter	-0.26	0.08	0.17	0.61	0.64	-0.29	0.04	0.13	0.71	0.53	-0.38	0.01	0.07	0.45	0.67	-0.38	0.01	0.07	0.47	0.66	-0.37	0.02	0.08	0.56	0.67
Akkermansia	0.11	0.15	0.84	0.71	0.94	0.05	0.47	0.86	0.94	0.94	0.02	0.84	0.62	0.97	0.88	0.02	0.84	0.63	0.97	0.89	0.01	0.88	0.59	0.97	0.85
Alistipes	0.13	0.33	0.43	0.74	0.76	0.06	0.61	0.27	0.96	0.68	0.02	0.86	0.46	0.97	0.76	0.03	0.85	0.48	0.97	0.77	0.02	0.85	0.50	0.96	0.81
Allisonella	-0.28	0.03	0.59	0.38	0.87	-0.13	0.29	0.82	0.83	0.94	-0.08	0.54	0.92	0.94	0.97	-0.08	0.54	0.95	0.93	0.97	-0.08	0.56	0.95	0.92	0.98
Anaerofilum	0.24	0.17	0.94	0.71	0.97	0.11	0.52	0.81	0.94	0.94	0.08	0.65	0.75	0.94	0.95	0.08	0.67	0.75	0.94	0.94	0.08	0.66	0.75	0.92	0.91
Anaerostipes	0.07	0.65	0.88	0.89	0.97	0.01	0.95	0.96	1.00	0.97	0.00	0.98	0.96	0.99	0.97	0.00	1.00	0.93	1.00	0.97	0.02	0.92	0.91	0.97	0.98
Anaerotruncus	0.05	0.74	0.90	0.93	0.97	0.00	0.98	0.96	1.00	0.97	-0.04	0.79	0.81	0.96	0.95	-0.04	0.76	0.79	0.95	0.95	-0.04	0.78	0.78	0.96	0.91
Bacteroides	0.14	0.51	0.57	0.81	0.87	0.09	0.68	0.61	0.97	0.88	0.04	0.85	0.78	0.97	0.95	0.05	0.81	0.76	0.96	0.94	0.04	0.86	0.76	0.96	0.91
Barnesiella	0.01	0.86	0.08	0.94	0.54	0.02	0.74	0.10	0.99	0.53	0.03	0.70	0.31	0.94	0.70	0.03	0.71	0.30	0.94	0.68	0.04	0.65	0.32	0.92	0.74
Bifidobacterium	0.08	0.44	0.30	0.79	0.70	0.12	0.22	0.38	0.83	0.73	0.09	0.39	0.64	0.91	0.88	0.09	0.37	0.62	0.89	0.89	0.09	0.36	0.61	0.90	0.85
Bilophila	0.15	0.12	0.83	0.70	0.94	0.08	0.38	0.72	0.93	0.92	0.10	0.34	0.96	0.89	0.97	0.10	0.32	0.99	0.86	0.99	0.09	0.36	0.98	0.90	0.98
Blautia	0.16	0.62	0.70	0.89	0.92	0.09	0.77	0.61	0.99	0.88	0.10	0.74	0.68	0.94	0.91	0.11	0.73	0.65	0.94	0.89	0.17	0.61	0.60	0.92	0.85
Butyricicoccus	-0.01	0.97	0.46	0.97	0.76	-0.20	0.33	0.29	0.87	0.68	-0.25	0.25	0.43	0.89	0.73	-0.24	0.26	0.44	0.86	0.75	-0.25	0.26	0.41	0.90	0.76
Butyricimonas	0.06	0.43	0.83	0.79	0.94	0.04	0.64	0.83	0.96	0.94	0.06	0.47	0.59	0.94	0.86	0.06	0.47	0.61	0.93	0.88	0.06	0.44	0.55	0.92	0.83
CAG.56	-0.04	0.64	0.08	0.89	0.54	-0.05	0.57	0.14	0.96	0.53	-0.08	0.41	0.17	0.91	0.67	-0.08	0.42	0.17	0.90	0.67	-0.07	0.45	0.18	0.92	0.67
Candidatus_Soleaferrea	0.19	0.34	0.10	0.74	0.54	0.16	0.41	0.07	0.93	0.53	0.12	0.56	0.13	0.94	0.67	0.12	0.56	0.13	0.93	0.67	0.12	0.56	0.11	0.92	0.67
Caproiciproducens	-0.04	0.82	0.66	0.94	0.89	-0.07	0.66	0.45	0.96	0.82	-0.16	0.34	0.38	0.89	0.73	-0.17	0.34	0.38	0.86	0.73	-0.18	0.32	0.36	0.90	0.74
Christensenellaceae_R.7_group	0.04	0.67	0.27	0.90	0.70	-0.04	0.65	0.11	0.96	0.53	-0.09	0.30	0.10	0.89	0.67	-0.09	0.28	0.09	0.86	0.67	-0.09	0.30	0.10	0.90	0.67
Clostridium_sensu_stricto_1	0.04	0.74	0.43	0.93	0.76	0.07	0.51	0.54	0.94	0.85	0.03	0.79	0.37	0.96	0.73	0.03	0.78	0.38	0.95	0.73	0.03	0.79	0.40	0.96	0.76
Colidextribacter	-0.21	0.26	0.95	0.74	0.97	-0.22	0.21	0.77	0.83	0.94	-0.19	0.29	0.25	0.89	0.67	-0.19	0.30	0.23	0.86	0.68	-0.17	0.35	0.23	0.90	0.67
Collinsella	-0.30	0.00	0.10	0.06	0.54	-0.22	0.01	0.13	0.52	0.53	-0.22	0.01	0.14	0.45	0.67	-0.22	0.01	0.14	0.47	0.67	-0.23	0.01	0.12	0.56	0.67
Coprobacter	-0.05	0.65	0.54	0.89	0.84	-0.08	0.45	0.57	0.94	0.86	-0.07	0.53	0.93	0.94	0.97	-0.07	0.51	0.92	0.93	0.97	-0.08	0.50	0.89	0.92	0.98
Coprococcus	-0.08	0.47	0.04	0.81	0.48	-0.05	0.64	0.10	0.96	0.53	-0.06	0.55	0.16	0.94	0.67	-0.06	0.55	0.16	0.93	0.67	-0.05	0.62	0.19	0.92	0.67
Defluviitaleaceae_UCG.011	0.15	0.34	0.32	0.74	0.70	0.10	0.52	0.25	0.94	0.68	0.05	0.74	0.20	0.94	0.67	0.05	0.75	0.20	0.95	0.68	0.06	0.72	0.22	0.96	0.67
Desulfovibrio	-0.10	0.25	0.53	0.74	0.84	-0.09	0.27	0.53	0.83	0.85	-0.08	0.38	0.88	0.91	0.97	-0.08	0.34	0.85	0.86	0.97	-0.08	0.39	0.87	0.91	0.98
Dialister	-0.05	0.44	0.22	0.79	0.66	-0.04	0.54	0.29	0.94	0.68	-0.08	0.30	0.19	0.89	0.67	-0.08	0.30	0.18	0.86	0.67	-0.08	0.30	0.18	0.90	0.67
DNF00809	-0.21	0.25	0.02	0.74	0.48	-0.22	0.21	0.03	0.83	0.53	-0.26	0.15	0.02	0.89	0.59	-0.26	0.15	0.02	0.86	0.61	-0.26	0.15	0.02	0.90	0.56
Dorea	-0.17	0.17	0.22	0.71	0.66	-0.16	0.16	0.14	0.83	0.53	-0.13	0.28	0.30	0.89	0.70	-0.13	0.29	0.30	0.86	0.68	-0.14	0.26	0.28	0.90	0.67
DTU089	-0.08	0.56	0.54	0.84	0.84	-0.15	0.27	0.27	0.83	0.68	-0.17	0.22	0.26	0.89	0.67	-0.17	0.22	0.27	0.86	0.68	-0.16	0.26	0.28	0.90	0.67
Eggerthella	0.21	0.11	0.31	0.69	0.70	0.16	0.20	0.47	0.83	0.82	0.18	0.18	0.37	0.89	0.73	0.18	0.17	0.35	0.86	0.73	0.18	0.18	0.39	0.90	0.76
Eisenbergiella	0.02	0.86	0.31	0.94	0.70	0.02	0.88	0.33	1.00	0.71	-0.01	0.93	0.47	0.99	0.77	-0.02	0.91	0.45	0.97	0.75	-0.01	0.93	0.50	0.97	0.81
Erysipelatoclostridium	0.26	0.06	0.06	0.52	0.48	0.24	0.06	0.09	0.71	0.53	0.22	0.10	0.18	0.89	0.67	0.22	0.11	0.18	0.86	0.67	0.23	0.10	0.17	0.90	0.67
Erysipelotrichaceae_UCG.003	-0.04	0.68	0.45	0.90	0.76	-0.04	0.65	0.52	0.96	0.85	-0.12	0.25	0.25	0.89	0.67	-0.12	0.25	0.25	0.86	0.68	-0.12	0.27	0.28	0.90	0.67
Escherichia.Shigella	-0.01	0.92	0.62	0.95	0.88	0.07	0.45	0.30	0.94	0.69	0.05	0.59	0.55	0.94	0.84	0.05	0.58	0.54	0.93	0.83	0.05	0.61	0.56	0.92	0.83
Faecalibacterium	0.19	0.27	0.89	0.74	0.97	0.07	0.67	0.50	0.96	0.85	0.01	0.94	0.29	0.99	0.70	0.02	0.93	0.29	0.97	0.68	0.04	0.83	0.35	0.96	0.74
Family_XIII_AD3011_group	0.04	0.77	0.16	0.93	0.64	-0.06	0.64	0.09	0.96	0.53	-0.13	0.36	0.17	0.91	0.67	-0.13	0.34	0.17	0.86	0.67	-0.13	0.35	0.17	0.90	0.67
Family_XIII_UCG.001	-0.11	0.48	0.05	0.81	0.48	-0.15	0.32	0.05	0.85	0.53	-0.16	0.34	0.10	0.89	0.67	-0.16	0.32	0.10	0.86	0.67	-0.16	0.34	0.11	0.90	0.67
FD2005	-0.20	0.17	0.18	0.71	0.64	-0.08	0.55	0.56	0.94	0.86	-0.14	0.33	0.33	0.89	0.72	-0.14	0.33	0.32	0.86	0.70	-0.13	0.38	0.37	0.91	0.74
Flavonifractor	0.05	0.69	0.16	0.90	0.64	0.03	0.79	0.24	1.00	0.68	-0.02	0.91	0.42	0.99	0.73	-0.01	0.93	0.40	0.97	0.73	-0.01	0.93	0.41	0.97	0.76
Fournierella	0.01	0.96	0.70	0.97	0.92	-0.03	0.82	0.51	1.00	0.85	-0.05	0.75	0.39	0.94	0.73	-0.06	0.72	0.38	0.94	0.73	-0.03	0.84	0.45	0.96	0.77
Frisingicoccus	0.17	0.14	0.09	0.71	0.54	0.13	0.24	0.13	0.83	0.53	0.06	0.62	0.18	0.94	0.67	0.06	0.63	0.17	0.94	0.67	0.05	0.66	0.21	0.92	0.67
Fusicatenibacter	-0.17	0.11	0.03	0.69	0.48	-0.20	0.05	0.03	0.71	0.53	-0.23	0.03	0.05	0.65	0.64	-0.23	0.03	0.05	0.70	0.61	-0.24	0.03	0.04	0.62	0.56
Fusobacterium	-0.13	0.29	0.84	0.74	0.94	-0.07	0.58	0.65	0.96	0.89	0.00	0.99	0.92	0.99	0.97	0.00	0.97	0.92	1.00	0.97	0.00	0.99	0.98	0.99	0.98
GCA.900066575	0.05	0.74	0.66	0.93	0.89	-0.03	0.83	0.66	1.00	0.89	-0.08	0.59	0.69	0.94	0.91	-0.09	0.57	0.69	0.93	0.91	-0.08	0.61	0.58	0.92	0.84
Gordonibacter	0.37	0.02	0.40	0.34	0.76	0.25	0.10	0.84	0.83	0.94	0.22	0.18	0.75	0.89	0.95	0.21	0.18	0.74	0.86	0.94	0.21	0.20	0.77	0.90	0.91
Granulicatella	0.05	0.78	0.64	0.93	0.88	0.10	0.53	0.92	0.94	0.97	0.07	0.71	0.73	0.94	0.94	0.07	0.69	0.76	0.94	0.94	0.08	0.68	0.72	0.92	0.91
Haemophilus	0.13	0.23	0.77	0.74	0.94	0.10	0.34	0.77	0.87	0.94	0.07	0.52	0.62	0.94	0.88	0.07	0.51	0.64	0.93	0.89	0.08	0.45	0.68	0.92	0.90
Harryflintia	0.02	0.91	0.82	0.94	0.94	0.00	0.99	0.85	1.00	0.94	-0.01	0.94	0.89	0.99	0.97	-0.02	0.92	0.89	0.97	0.97	-0.01	0.94	0.97	0.97	0.98
Holdemanella	-0.12	0.24	0.42	0.74	0.76	-0.08	0.40	0.53	0.93	0.85	-0.04	0.73	0.91	0.94	0.97	-0.04	0.72	0.89	0.94	0.97	-0.04	0.73	0.89	0.96	0.98
Holdemania	0.35	0.21	0.38	0.74	0.76	0.35	0.19	0.35	0.83	0.71	0.17	0.55	0.57	0.94	0.86	0.16	0.56	0.57	0.93	0.86	0.16	0.58	0.52	0.92	0.82
Hungatella	-0.02	0.85	0.06	0.94	0.48	-0.01	0.91	0.08	1.00	0.53	-0.02	0.85	0.25	0.97	0.67	-0.02	0.87	0.23	0.97	0.68	-0.02	0.91	0.27	0.97	0.67
Hydrogenoanaerobacterium	0.15	0.32	0.30	0.74	0.70	0.02	0.90	0.14	1.00	0.53	0.01	0.97	0.23	0.99	0.67	0.00	0.99	0.23	1.00	0.68	0.00	0.99	0.21	0.99	0.67
Incertae_Sedis	0.05	0.81	0.58	0.94	0.87	0.02	0.91	0.35	1.00	0.71	-0.09	0.65	0.33	0.94	0.72	-0.09	0.66	0.34	0.94	0.73	-0.10	0.65	0.32	0.92	0.74
Intestinibacter	0.12	0.33	0.92	0.74	0.97	0.14	0.23	0.67	0.83	0.90	0.04	0.74	0.26	0.94	0.67	0.05	0.69	0.28	0.94	0.68	0.06	0.65	0.28	0.92	0.67
Intestinimonas	0.15	0.20	0.33	0.74	0.70	0.06	0.58	0.20	0.96	0.62	0.02	0.85	0.17	0.97	0.67	0.02	0.85	0.17	0.97	0.67	0.02	0.85	0.14	0.96	0.67
Lachnoclostridium	-0.20	0.42	0.34	0.79	0.72	-0.03	0.91	0.22	1.00	0.65	0.11	0.67	0.25	0.94	0.67	0.11	0.65	0.24	0.94	0.68	0.11	0.68	0.25	0.92	0.67
Lachnospira	0.08	0.47	0.01	0.81	0.48	0.00	1.00	0.02	1.00	0.53	-0.12	0.29	0.00	0.89	0.11	-0.12	0.31	0.00	0.86	0.12	-0.11	0.35	0.00	0.90	0.16
Lachnospiraceae_FCS020_group	0.20	0.14	0.42	0.71	0.76	0.10	0.45	0.36	0.94	0.72	0.04	0.79	0.34	0.96	0.72	0.04	0.78	0.34	0.95	0.73	0.03	0.81	0.33	0.96	0.74
Lachnospiraceae_ND3007_group	-0.11	0.21	0.00	0.74	0.16	-0.14	0.10	0.00	0.83	0.31	-0.18	0.05	0.00	0.76	0.13	-0.18	0.06	0.00	0.80	0.14	-0.18	0.06	0.00	0.85	0.16
Lachnospiraceae_NK4A136_group	0.08	0.49	0.77	0.81	0.94	-0.02	0.85	0.61	1.00	0.88	-0.14	0.28	0.49	0.89	0.78	-0.13	0.29	0.50	0.86	0.80	-0.13	0.33	0.54	0.90	0.82
Lachnospiraceae_UCG.001	0.06	0.50	0.21	0.81	0.66	0.02	0.80	0.13	1.00	0.53	-0.06	0.57	0.04	0.94	0.64	-0.06	0.54	0.04	0.93	0.61	-0.06	0.57	0.04	0.92	0.56
Lachnospiraceae_UCG.004	0.06	0.56	0.73	0.84	0.93	0.03	0.75	0.69	0.99	0.91	-0.01	0.92	0.85	0.99	0.97	-0.01	0.93	0.84	0.97	0.97	-0.02	0.85	0.88	0.96	0.98
Lachnospiraceae_UCG.010	-0.15	0.30	0.79	0.74	0.94	-0.21	0.13	0.84	0.83	0.94	-0.22	0.14	0.98	0.89	0.98	-0.22	0.13	0.99	0.86	0.99	-0.22	0.13	0.97	0.90	0.98
Lactobacillus	-0.24	0.02	0.81	0.34	0.94	-0.18	0.07	0.94	0.71	0.97	-0.21	0.06	0.90	0.76	0.97	-0.21	0.06	0.90	0.80	0.97	-0.22	0.05	0.77	0.85	0.91
Lactococcus	0.19	0.30	0.46	0.74	0.76	0.07	0.69	0.86	0.97	0.94	0.07	0.72	0.94	0.94	0.97	0.07	0.71	0.93	0.94	0.97	0.04	0.82	0.98	0.96	0.98
Marvinbryantia	-0.05	0.64	0.61	0.89	0.87	-0.08	0.38	0.61	0.93	0.88	-0.06	0.59	0.66	0.94	0.89	-0.06	0.59	0.67	0.93	0.89	-0.05	0.66	0.71	0.92	0.91
Megasphaera	-0.06	0.40	0.32	0.77	0.70	0.00	0.98	0.26	1.00	0.68	0.06	0.41	0.17	0.91	0.67	0.06	0.41	0.18	0.90	0.67	0.05	0.50	0.21	0.92	0.67

© 2022 Meyer K et al. *JAMA Network Open.*

Monoglobus	0.10	0.37	0.05	0.76	0.48	0.04	0.74	0.02	0.99	0.53	-0.06	0.59	0.00	0.94	0.11	-0.06	0.59	0.00	0.93	0.12	-0.06	0.61	0.00	0.92	0.16
Moryella	-0.03	0.87	0.20	0.94	0.66	-0.11	0.52	0.17	0.94	0.59	-0.20	0.29	0.19	0.89	0.67	-0.20	0.29	0.19	0.86	0.67	-0.19	0.31	0.19	0.90	0.67
Negativibacillus	-0.14	0.18	0.73	0.71	0.93	-0.11	0.29	0.96	0.83	0.97	-0.09	0.38	0.89	0.91	0.97	-0.10	0.37	0.91	0.89	0.97	-0.10	0.35	0.91	0.90	0.98
NK4A214_group	-0.08	0.37	0.04	0.76	0.48	-0.12	0.16	0.03	0.83	0.53	-0.13	0.13	0.07	0.89	0.67	-0.14	0.12	0.07	0.86	0.66	-0.13	0.14	0.07	0.90	0.67
Odoribacter	0.14	0.08	0.61	0.61	0.87	0.08	0.30	0.89	0.83	0.96	0.09	0.26	0.59	0.89	0.86	0.09	0.26	0.60	0.86	0.88	0.10	0.23	0.52	0.90	0.82
Oscillibacter	0.11	0.43	0.30	0.79	0.70	0.09	0.49	0.39	0.94	0.73	0.01	0.97	0.46	0.99	0.76	0.00	0.99	0.46	1.00	0.75	-0.01	0.96	0.43	0.99	0.77
Oscillospira	0.04	0.85	0.98	0.94	0.98	0.00	0.98	0.72	1.00	0.92	-0.06	0.75	0.64	0.94	0.88	-0.06	0.75	0.66	0.95	0.89	-0.04	0.83	0.71	0.96	0.91
Oxalobacter	0.02	0.88	0.04	0.94	0.48	-0.07	0.61	0.04	0.96	0.53	-0.11	0.45	0.10	0.92	0.67	-0.12	0.44	0.10	0.90	0.67	-0.12	0.42	0.08	0.91	0.67
Paludicola	0.06	0.68	0.64	0.90	0.88	0.00	0.99	0.78	1.00	0.94	-0.03	0.83	0.94	0.97	0.97	-0.04	0.79	0.94	0.95	0.97	-0.05	0.77	0.95	0.96	0.98
Parabacteroides	0.03	0.77	0.22	0.93	0.66	0.03	0.77	0.47	0.99	0.82	0.05	0.61	0.42	0.94	0.73	0.06	0.59	0.41	0.93	0.73	0.06	0.55	0.37	0.92	0.74
Paraprevotella	-0.09	0.22	0.14	0.74	0.62	-0.08	0.26	0.08	0.83	0.53	-0.06	0.44	0.23	0.91	0.67	-0.06	0.42	0.23	0.90	0.68	-0.06	0.43	0.23	0.91	0.67
Parasutterella	0.18	0.02	0.52	0.34	0.84	0.16	0.03	0.64	0.71	0.89	0.09	0.26	0.73	0.89	0.94	0.09	0.26	0.73	0.86	0.94	0.09	0.25	0.66	0.90	0.89
Peptococcus	0.11	0.50	0.96	0.81	0.97	0.01	0.95	0.78	1.00	0.94	-0.08	0.62	0.78	0.94	0.95	-0.09	0.60	0.78	0.93	0.94	-0.09	0.58	0.71	0.92	0.91
Phascolarctobacterium	0.11	0.12	0.15	0.70	0.62	0.11	0.12	0.19	0.83	0.60	0.11	0.15	0.25	0.89	0.67	0.10	0.16	0.25	0.86	0.68	0.11	0.14	0.22	0.90	0.67
Phocea	-0.33	0.06	0.45	0.52	0.76	-0.38	0.02	0.32	0.69	0.71	-0.41	0.02	0.36	0.45	0.73	-0.41	0.02	0.36	0.47	0.73	-0.40	0.02	0.42	0.56	0.76
Prevotella	-0.06	0.37	0.25	0.76	0.70	-0.04	0.52	0.39	0.94	0.73	-0.01	0.91	0.27	0.99	0.68	-0.01	0.90	0.29	0.97	0.68	0.00	0.98	0.25	0.99	0.67
Romboutsia	-0.03	0.82	0.59	0.94	0.87	0.01	0.96	0.61	1.00	0.88	-0.05	0.66	0.49	0.94	0.78	-0.05	0.69	0.52	0.94	0.81	-0.04	0.74	0.57	0.96	0.84
Roseburia	-0.06	0.63	0.36	0.89	0.74	-0.08	0.49	0.37	0.94	0.72	-0.21	0.12	0.11	0.89	0.67	-0.21	0.12	0.11	0.86	0.67	-0.19	0.16	0.14	0.90	0.67
Ruminococcus	0.06	0.51	0.20	0.81	0.66	0.00	0.96	0.14	1.00	0.53	-0.07	0.42	0.05	0.91	0.64	-0.07	0.41	0.05	0.90	0.61	-0.07	0.42	0.05	0.91	0.56
Sellimonas	-0.02	0.86	0.05	0.94	0.48	-0.04	0.73	0.05	0.99	0.53	-0.05	0.70	0.05	0.94	0.64	-0.05	0.67	0.05	0.94	0.61	-0.06	0.66	0.05	0.92	0.56
Senegalimassilia	-0.24	0.03	0.14	0.39	0.62	-0.21	0.05	0.25	0.71	0.68	-0.22	0.05	0.26	0.76	0.67	-0.22	0.05	0.25	0.80	0.68	-0.21	0.06	0.26	0.85	0.67
Shuttleworthia	0.30	0.05	0.96	0.52	0.97	0.16	0.25	0.85	0.83	0.94	0.17	0.28	0.80	0.89	0.95	0.16	0.29	0.81	0.86	0.96	0.16	0.32	0.89	0.90	0.98
Slackia	-0.25	0.01	0.17	0.34	0.64	-0.25	0.01	0.08	0.52	0.53	-0.25	0.01	0.10	0.45	0.67	-0.25	0.01	0.09	0.47	0.67	-0.26	0.01	0.11	0.56	0.67
Streptococcus	0.17	0.26	0.77	0.74	0.94	0.14	0.32	0.91	0.85	0.97	0.15	0.31	0.79	0.89	0.95	0.16	0.30	0.83	0.86	0.96	0.15	0.32	0.75	0.90	0.91
Subdoligranulum	0.07	0.52	0.32	0.81	0.70	0.04	0.74	0.33	0.99	0.71	0.01	0.95	0.43	0.99	0.73	0.01	0.96	0.43	1.00	0.75	0.02	0.90	0.48	0.97	0.81
Sutterella	-0.17	0.02	0.32	0.34	0.70	-0.12	0.06	0.34	0.71	0.71	-0.08	0.29	0.59	0.89	0.86	-0.08	0.29	0.59	0.86	0.87	-0.07	0.31	0.63	0.90	0.86
TM7x	-0.20	0.21	0.10	0.74	0.54	-0.22	0.15	0.18	0.83	0.60	-0.16	0.31	0.36	0.89	0.73	-0.16	0.32	0.37	0.86	0.73	-0.16	0.34	0.34	0.90	0.74
Turicibacter	0.18	0.31	0.46	0.74	0.76	0.25	0.13	0.51	0.83	0.85	0.23	0.22	0.43	0.89	0.73	0.23	0.21	0.45	0.86	0.75	0.23	0.21	0.50	0.90	0.81
Tuzzerella	0.03	0.79	0.11	0.94	0.58	0.03	0.80	0.16	1.00	0.58	-0.03	0.81	0.29	0.97	0.70	-0.03	0.80	0.30	0.95	0.68	-0.04	0.77	0.28	0.96	0.67
UBA1819	0.01	0.94	0.89	0.96	0.97	-0.02	0.89	0.98	1.00	0.98	-0.08	0.63	0.84	0.94	0.97	-0.08	0.60	0.83	0.93	0.96	-0.09	0.57	0.86	0.92	0.98
UCG.002	0.07	0.39	0.39	0.77	0.76	0.01	0.86	0.29	1.00	0.68	-0.01	0.89	0.40	0.99	0.73	-0.01	0.87	0.39	0.97	0.73	-0.01	0.93	0.44	0.97	0.77
UCG.003	-0.09	0.35	0.08	0.74	0.54	-0.12	0.18	0.14	0.83	0.53	-0.16	0.10	0.18	0.89	0.67	-0.16	0.10	0.18	0.86	0.67	-0.16	0.09	0.18	0.90	0.67
UCG.005	-0.03	0.72	0.14	0.93	0.62	-0.11	0.23	0.05	0.83	0.53	-0.18	0.08	0.04	0.89	0.64	-0.19	0.07	0.04	0.85	0.61	-0.19	0.08	0.04	0.90	0.56
UCG.009	-0.02	0.89	0.24	0.94	0.70	-0.12	0.42	0.20	0.93	0.62	-0.20	0.22	0.17	0.89	0.67	-0.20	0.23	0.17	0.86	0.67	-0.19	0.24	0.17	0.90	0.67
Veillonella	0.10	0.34	0.75	0.74	0.94	0.12	0.25	0.95	0.83	0.97	0.06	0.61	0.51	0.94	0.79	0.06	0.58	0.53	0.93	0.83	0.07	0.57	0.53	0.92	0.82
Victivallis	-0.06	0.60	0.04	0.89	0.48	-0.09	0.42	0.06	0.93	0.53	-0.13	0.28	0.13	0.89	0.67	-0.13	0.27	0.13	0.86	0.67	-0.13	0.27	0.11	0.90	0.67

Multivariable-adjusted regression models adjusted for:

Model 1: sequencing batch, age, race, gender, study center

Model 2: Model 1, with education

Model 3: Model 2, with physical activity, diet score, medication use, and smoking

Model 4: Model 3 with BMI

Model 5: Model 4 with hypertension and diabetes

© 2022 Meyer K et al. *JAMA Network Open.*

eTable 22: Multivariable-adjusted regression coefficients, p-values, race interaction p-values, false discovery rate (FDR)-adjusted p-values for race-genera interactions with respect to category fluency.

	Model1					Model2					Model3					Model4					Model5				
	beta	p-value	p-val int	FDR	FDR-int	beta	p-value	p-val int	FDR	FDR-int	beta	p-value	p-val int	FDR	FDR-int	beta	p-value	p-val int	FDR	FDR-int	beta	p-value	p-val int	FDR	FDR-int
Acetanaerobacterium	-0.58	0.36	0.37	0.69	0.80	-0.58	0.35	0.19	0.79	0.75	-0.42	0.53	0.40	0.91	0.81	-0.43	0.52	0.41	0.92	0.85	-0.38	0.57	0.52	0.97	0.88
Acidaminococcus	-0.25	0.18	0.62	0.51	0.86	-0.16	0.36	0.81	0.79	0.93	-0.18	0.38	1.00	0.91	1.00	-0.17	0.41	0.99	0.92	0.99	-0.17	0.41	0.98	0.97	0.98
Actinomyces	-0.85	0.09	0.15	0.44	0.68	-0.72	0.13	0.18	0.63	0.75	-0.64	0.23	0.20	0.90	0.81	-0.64	0.23	0.20	0.90	0.81	-0.68	0.21	0.16	0.97	0.75
Adlercreutzia	-0.31	0.35	0.22	0.69	0.68	-0.44	0.18	0.11	0.69	0.75	-0.58	0.10	0.07	0.85	0.81	-0.59	0.10	0.08	0.90	0.80	-0.60	0.09	0.08	0.97	0.75
Agathobacter	-0.10	0.82	0.44	0.92	0.81	-0.16	0.70	0.38	0.93	0.80	-0.24	0.58	0.43	0.91	0.83	-0.21	0.64	0.42	0.96	0.85	-0.17	0.70	0.41	0.97	0.88
Akkermansia	0.34	0.13	0.08	0.46	0.68	0.20	0.36	0.14	0.79	0.75	0.18	0.46	0.16	0.91	0.81	0.18	0.45	0.15	0.92	0.80	0.15	0.53	0.18	0.97	0.75
Alistipes	0.45	0.24	0.20	0.56	0.68	0.30	0.42	0.27	0.82	0.77	0.11	0.77	0.33	0.96	0.81	0.13	0.73	0.30	0.96	0.83	0.14	0.72	0.25	0.97	0.87
Allisonella	0.14	0.69	0.65	0.89	0.86	0.54	0.13	0.44	0.63	0.82	0.56	0.15	0.45	0.85	0.84	0.55	0.16	0.53	0.90	0.87	0.59	0.14	0.50	0.97	0.88
Anaerofilum	0.70	0.16	0.38	0.47	0.80	0.37	0.45	0.45	0.82	0.82	0.34	0.52	0.49	0.91	0.87	0.32	0.55	0.49	0.92	0.86	0.25	0.64	0.56	0.97	0.88
Anaerostipes	-0.01	0.99	0.79	0.99	0.90	-0.16	0.72	0.85	0.93	0.94	-0.22	0.64	0.92	0.93	0.99	-0.19	0.68	0.85	0.96	0.95	-0.19	0.70	0.84	0.97	0.96
Anaerotruncus	0.43	0.31	0.15	0.65	0.68	0.32	0.43	0.16	0.82	0.75	0.33	0.45	0.16	0.91	0.81	0.29	0.51	0.15	0.92	0.80	0.32	0.47	0.12	0.97	0.75
Bacteroides	0.24	0.70	0.26	0.89	0.68	0.10	0.86	0.27	0.94	0.77	0.07	0.91	0.31	0.96	0.81	0.15	0.81	0.28	0.96	0.83	0.17	0.78	0.26	0.98	0.87
Barnesiella	0.48	0.02	0.74	0.29	0.90	0.51	0.01	0.86	0.36	0.94	0.57	0.01	0.98	0.65	1.00	0.56	0.01	0.94	0.73	0.99	0.55	0.02	0.86	0.84	0.96
Bifidobacterium	0.41	0.15	0.02	0.47	0.46	0.51	0.06	0.03	0.47	0.59	0.42	0.15	0.10	0.85	0.81	0.45	0.12	0.08	0.90	0.80	0.46	0.12	0.08	0.97	0.75
Bilophila	0.74	0.01	0.40	0.13	0.80	0.58	0.03	0.69	0.37	0.93	0.48	0.10	0.91	0.85	0.99	0.51	0.08	0.98	0.90	0.99	0.45	0.13	0.83	0.97	0.96
Blautia	-0.76	0.40	0.65	0.70	0.86	-0.93	0.29	0.71	0.75	0.93	-1.02	0.27	0.55	0.90	0.89	-0.97	0.30	0.63	0.90	0.93	-0.99	0.32	0.63	0.97	0.88
Butyricicoccus	0.70	0.26	0.72	0.59	0.90	0.24	0.69	0.90	0.93	0.96	0.42	0.52	0.51	0.91	0.87	0.46	0.48	0.48	0.92	0.86	0.42	0.52	0.51	0.97	0.88
Butyricimonas	0.72	0.00	0.03	0.12	0.46	0.66	0.00	0.03	0.36	0.55	0.74	0.00	0.02	0.25	0.62	0.73	0.00	0.02	0.27	0.65	0.76	0.00	0.02	0.21	0.68
CAG.56	0.00	0.99	0.26	0.99	0.68	-0.02	0.95	0.40	0.98	0.80	-0.04	0.90	0.54	0.96	0.89	-0.02	0.94	0.59	0.97	0.92	-0.02	0.94	0.57	0.98	0.88
Candidatus_Soleaferrea	0.22	0.70	0.57	0.89	0.86	0.14	0.80	0.49	0.94	0.83	0.09	0.88	0.67	0.96	0.91	0.09	0.88	0.65	0.96	0.93	0.12	0.84	0.56	0.98	0.88
Caproiciproducens	-0.27	0.57	0.55	0.86	0.84	-0.35	0.45	0.70	0.82	0.93	-0.33	0.52	0.68	0.91	0.91	-0.34	0.50	0.68	0.92	0.93	-0.42	0.41	0.75	0.97	0.91
Christensenellaceae_R.7_group	0.60	0.01	0.13	0.17	0.68	0.44	0.06	0.23	0.47	0.77	0.36	0.15	0.34	0.85	0.81	0.34	0.19	0.37	0.90	0.83	0.32	0.22	0.40	0.97	0.88
Clostridium_sensu_stricto_1	-0.21	0.52	0.26	0.80	0.68	-0.13	0.68	0.32	0.93	0.78	-0.16	0.64	0.32	0.93	0.81	-0.14	0.67	0.35	0.96	0.83	-0.18	0.59	0.30	0.97	0.87
Colidextribacter	-0.13	0.80	0.84	0.92	0.93	-0.17	0.75	0.94	0.93	0.96	-0.11	0.84	0.77	0.96	0.91	-0.10	0.86	0.70	0.96	0.93	-0.05	0.93	0.70	0.98	0.91
Collinsella	0.30	0.24	0.81	0.56	0.91	0.51	0.04	0.66	0.37	0.93	0.44	0.09	0.95	0.85	1.00	0.44	0.09	0.99	0.90	0.99	0.41	0.12	0.93	0.97	0.97
Coprobacter	0.50	0.11	0.46	0.45	0.82	0.43	0.15	0.41	0.63	0.80	0.41	0.21	0.30	0.85	0.81	0.39	0.23	0.32	0.90	0.83	0.34	0.30	0.39	0.97	0.88
Coprococcus	0.10	0.74	0.88	0.91	0.94	0.18	0.55	0.78	0.89	0.93	0.22	0.48	0.58	0.91	0.89	0.22	0.48	0.57	0.92	0.92	0.26	0.41	0.53	0.97	0.88
Defluviitaleaceae_UCG.011	0.38	0.40	0.90	0.70	0.94	0.26	0.57	0.83	0.89	0.94	0.41	0.39	0.92	0.91	0.99	0.38	0.43	0.93	0.92	0.99	0.34	0.49	0.95	0.97	0.97
Desulfovibrio	0.31	0.20	0.01	0.51	0.30	0.33	0.15	0.00	0.63	0.43	0.25	0.33	0.02	0.90	0.62	0.21	0.41	0.02	0.92	0.65	0.19	0.45	0.02	0.97	0.68
Dialister	-0.27	0.17	0.15	0.47	0.68	-0.24	0.21	0.19	0.73	0.75	-0.21	0.31	0.26	0.90	0.81	-0.21	0.32	0.25	0.90	0.83	-0.18	0.40	0.29	0.97	0.87
DNF00809	0.14	0.78	0.36	0.92	0.80	0.12	0.81	0.28	0.94	0.77	0.31	0.56	0.15	0.91	0.81	0.29	0.58	0.15	0.92	0.80	0.25	0.64	0.17	0.97	0.75
Dorea	0.05	0.88	0.64	0.96	0.86	0.06	0.85	0.75	0.94	0.93	0.05	0.88	0.68	0.96	0.91	0.06	0.86	0.69	0.96	0.93	0.04	0.92	0.73	0.98	0.91
DTU089	-0.17	0.68	0.40	0.89	0.80	-0.32	0.40	0.63	0.82	0.93	-0.39	0.34	0.76	0.91	0.91	-0.40	0.34	0.70	0.90	0.93	-0.31	0.45	0.60	0.97	0.88
Eggerthella	-0.82	0.03	0.10	0.29	0.68	-0.94	0.01	0.05	0.36	0.71	-0.86	0.03	0.09	0.76	0.81	-0.84	0.03	0.11	0.89	0.80	-0.82	0.04	0.11	0.87	0.75
Eisenbergiella	0.00	0.99	0.19	0.99	0.68	-0.01	0.98	0.20	0.99	0.75	-0.07	0.85	0.30	0.96	0.81	-0.10	0.79	0.26	0.96	0.83	-0.07	0.85	0.25	0.98	0.87
Erysipelatoclostridium	-0.11	0.77	0.20	0.92	0.68	-0.15	0.68	0.27	0.93	0.77	-0.23	0.58	0.57	0.91	0.89	-0.24	0.55	0.53	0.92	0.87	-0.21	0.61	0.47	0.97	0.88
Erysipelotrichaceae_UCG.003	-0.06	0.83	0.29	0.93	0.72	-0.07	0.81	0.33	0.94	0.78	-0.09	0.78	0.34	0.96	0.81	-0.10	0.75	0.33	0.96	0.83	-0.13	0.68	0.31	0.97	0.87
Escherichia.Shigella	-0.28	0.29	0.91	0.64	0.94	-0.10	0.70	0.75	0.93	0.93	-0.16	0.58	0.77	0.91	0.91	-0.14	0.62	0.79	0.96	0.94	-0.12	0.67	0.86	0.97	0.96
Faecalibacterium	0.48	0.33	0.46	0.69	0.82	0.20	0.68	0.75	0.93	0.93	0.05	0.92	0.99	0.96	1.00	0.08	0.88	0.96	0.96	0.99	0.09	0.85	0.93	0.98	0.97
Family_XIII_AD3011_group	0.70	0.06	0.08	0.40	0.68	0.47	0.20	0.11	0.73	0.75	0.53	0.19	0.05	0.85	0.81	0.49	0.23	0.06	0.90	0.80	0.42	0.30	0.07	0.97	0.75
Family_XIII_UCG.001	0.75	0.10	0.52	0.44	0.84	0.65	0.14	0.46	0.63	0.83	0.63	0.19	0.42	0.85	0.83	0.59	0.23	0.43	0.90	0.85	0.53	0.28	0.48	0.97	0.88
FD2005	-0.71	0.09	0.09	0.44	0.68	-0.44	0.28	0.28	0.74	0.77	-0.40	0.36	0.35	0.91	0.81	-0.41	0.35	0.32	0.91	0.83	-0.37	0.41	0.38	0.97	0.87
Flavonifractor	-0.26	0.47	0.62	0.77	0.86	-0.31	0.38	0.79	0.81	0.93	-0.32	0.42	1.00	0.91	1.00	-0.29	0.45	0.94	0.92	0.99	-0.25	0.52	0.88	0.97	0.97
Fournierella	-0.02	0.96	0.77	0.99	0.90	-0.13	0.77	0.93	0.93	0.96	-0.06	0.89	0.66	0.96	0.91	-0.11	0.81	0.70	0.96	0.93	-0.05	0.91	0.62	0.98	0.88
Frisingicoccus	0.31	0.36	0.51	0.69	0.84	0.20	0.53	0.62	0.89	0.93	0.13	0.72	0.69	0.96	0.91	0.11	0.74	0.68	0.96	0.93	0.10	0.77	0.71	0.98	0.91
Fusicatenibacter	0.07	0.80	0.16	0.92	0.68	0.00	1.00	0.17	1.00	0.75	-0.16	0.62	0.12	0.93	0.81	-0.12	0.70	0.13	0.96	0.80	-0.23	0.47	0.08	0.97	0.75
Fusobacterium	-0.06	0.87	0.46	0.96	0.82	0.10	0.78	0.33	0.93	0.78	0.17	0.64	0.72	0.93	0.91	0.15	0.69	0.72	0.96	0.93	0.21	0.58	0.55	0.97	0.88
GCA.900066575	0.83	0.05	0.11	0.35	0.68	0.64	0.12	0.10	0.63	0.75	0.57	0.20	0.19	0.85	0.81	0.53	0.23	0.19	0.90	0.80	0.56	0.21	0.13	0.97	0.75
Gordonibacter	0.17	0.72	0.91	0.89	0.94	-0.13	0.77	0.49	0.93	0.83	0.08	0.86	0.75	0.96	0.91	0.05	0.92	0.76	0.96	0.94	-0.03	0.96	0.65	0.98	0.88
Granulicatella	-0.44	0.38	0.23	0.69	0.68	-0.31	0.52	0.36	0.89	0.80	-0.36	0.49	0.27	0.91	0.81	-0.32	0.55	0.31	0.92	0.83	-0.21	0.69	0.36	0.97	0.87
Haemophilus	0.22	0.46	0.51	0.76	0.84	0.15	0.61	0.50	0.93	0.83	-0.03	0.93	0.39	0.96	0.81	-0.02	0.96	0.43	0.98	0.85	0.03	0.93	0.50	0.98	0.88
Harryflintia	-0.17	0.72	0.93	0.89	0.94	-0.21	0.64	0.96	0.93	0.97	-0.45	0.35	0.62	0.91	0.91	-0.49	0.32	0.60	0.90	0.93	-0.49	0.32	0.65	0.97	0.88
Holdemanella	0.25	0.37	0.33	0.69	0.78	0.34	0.22	0.24	0.73	0.77	0.48	0.11	0.15	0.85	0.81	0.47	0.12	0.17	0.90	0.80	0.46	0.13	0.19	0.97	0.75
Holdemania	-0.61	0.44	0.21	0.76	0.68	-0.60	0.44	0.20	0.82	0.75	-0.46	0.58	0.36	0.91	0.81	-0.50	0.55	0.37	0.92	0.83	-0.64	0.45	0.33	0.97	0.87
Hungatella	-0.43	0.23	0.23	0.56	0.68	-0.41	0.24	0.29	0.74	0.77	-0.44	0.25	0.51	0.90	0.87	-0.42	0.27	0.45	0.90	0.86	-0.34	0.37	0.36	0.97	0.87
Hydrogenoanaerobacterium	1.25	0.00	0.00	0.12	0.30	0.93	0.03	0.01	0.37	0.46	0.83	0.08	0.02	0.85	0.62	0.78	0.10	0.02	0.90	0.65	0.72	0.13	0.02	0.97	0.68
Incertae_Sedis	-0.26	0.66	0.75	0.89	0.90	-0.33	0.57	0.53	0.89	0.87	-0.27	0.67	0.58	0.95	0.89	-0.24	0.70	0.64	0.96	0.93	-0.29	0.64	0.58	0.97	0.88
Intestinibacter	-0.09	0.80	0.96	0.92	0.96	-0.04	0.90	0.84	0.96	0.94	-0.27	0.48	0.43	0.91	0.83	-0.21	0.57	0.47	0.92	0.86	-0.18	0.64	0.53	0.97	0.88
Intestinimonas	1.03	0.00	0.01	0.12	0.31	0.82	0.02	0.02	0.36	0.49	0.75	0.04	0.03	0.76	0.62	0.74	0.05	0.02	0.89	0.65	0.75	0.04	0.03	0.87	0.68
Lachnoclostridium	-1.41	0.05	0.32	0.35	0.78	-0.99	0.16	0.41	0.64	0.80	-0.77	0.30	0.35	0.90	0.81	-0.72	0.34	0.36	0.90	0.83	-0.65	0.38	0.39	0.97	0.88
Lachnospira	0.54	0.09	0.21	0.44	0.68	0.34	0.27	0.31	0.74	0.78	0.03	0.94	0.14	0.96	0.81	0.06	0.87	0.16	0.96	0.80	0.08	0.83	0.18	0.98	0.75
Lachnospiraceae_FCS020_group	0.59	0.13	0.78	0.46	0.90	0.35	0.35	0.73	0.79	0.93	0.45	0.27	0.92	0.90	0.99	0.46	0.27	0.93	0.90	0.99	0.38	0.37	0.81	0.97	0.95
Lachnospiraceae_ND3007_group	0.37	0.15	0.78	0.47	0.90	0.31	0.23	0.99	0.73	0.99	0.27	0.33	0.93	0.90	0.99	0.29	0.29	0.90	0.90	0.99	0.27	0.33	0.96	0.97	0.97
Lachnospiraceae_NK4A136_group	0.50	0.14	0.65	0.47	0.86	0.25	0.45	0.76	0.82	0.93	0.05	0.89	0.71	0.96	0.91	0.06	0.87	0.69	0.96	0.93	0.08	0.83	0.63	0.98	0.88
Lachnospiraceae_UCG.001	0.29	0.30	0.93	0.65	0.94	0.19	0.48	0.82	0.85	0.93	0.06	0.84	0.71	0.96	0.91	0.03	0.91	0.67	0.96	0.93	0.03	0.92	0.68	0.98	0.91
Lachnospiraceae_UCG.004	0.37	0.22	0.43	0.56	0.81	0.30	0.31	0.40	0.77	0.80	0.16	0.61	0.58	0.93	0.89	0.17	0.59	0.58	0.92	0.92	0.18	0.57	0.51	0.97	0.88
Lachnospiraceae_UCG.010	-0.21	0.62	0.53	0.89	0.84	-0.35	0.38	0.47	0.81	0.83	-0.24	0.58	0.29	0.91	0.81	-0.27	0.53	0.30	0.92	0.83	-0.24	0.57	0.27	0.97	0.87
Lactobacillus	-0.14	0.63	0.26	0.89	0.68	0.01	0.97	0.37	0.99	0.80	-0.03	0.94	0.50	0.96	0.87	0.00	0.99	0.49	0.99	0.86	0.00	1.00	0.60	1.00	0.88
Lactococcus	0.85	0.10	0.48	0.44	0.82	0.58	0.26	0.81	0.74	0.93	0.32	0.57	0.71	0.91	0.91	0.33	0.55	0.75	0.92	0.94	0.22	0.70	0.60	0.97	0.88
Marvinbryantia	-0.07	0.81	0.86	0.92	0.94	-0.16	0.56	0.88	0.89	0.95	-0.07	0.82	0.90	0.96	0.99	-0.07	0.82	0.87	0.96	0.97	-0.02	0.94	0.81	0.98	0.95
Megasphaera	-0.17	0.40	0.84	0.70	0.93	-0.02	0.91	0.78	0.96	0.93	0.03	0.90	0.86	0.96	0.97	0.02	0.93	0.79	0.96	0.94	0.00	0.99	0.70	1.00	0.91

© 2022 Meyer K et al. *JAMA Network Open.*

Monoglobus	0.37	0.26	0.40	0.59	0.80	0.21	0.51	0.25	0.88	0.77	0.04	0.90	0.18	0.96	0.81	0.04	0.91	0.19	0.96	0.80	-0.03	0.93	0.12	0.98	0.75
Moryella	0.27	0.59	0.41	0.87	0.80	0.08	0.87	0.39	0.94	0.80	-0.06	0.91	0.40	0.96	0.81	-0.07	0.90	0.39	0.96	0.83	-0.10	0.86	0.36	0.98	0.87
Negativibacillus	-0.01	0.98	0.69	0.99	0.88	0.08	0.80	0.50	0.94	0.83	0.09	0.78	0.46	0.96	0.84	0.07	0.82	0.50	0.96	0.86	0.04	0.90	0.53	0.98	0.88
NK4A214_group	0.52	0.04	0.10	0.29	0.68	0.42	0.08	0.09	0.49	0.75	0.33	0.20	0.11	0.85	0.81	0.31	0.24	0.13	0.90	0.80	0.29	0.27	0.15	0.97	0.75
Odoribacter	0.61	0.01	0.07	0.13	0.68	0.47	0.03	0.19	0.37	0.75	0.34	0.14	0.33	0.85	0.81	0.34	0.15	0.34	0.90	0.83	0.33	0.17	0.33	0.97	0.87
Oscillibacter	0.22	0.58	0.93	0.86	0.94	0.18	0.64	0.92	0.93	0.96	0.50	0.24	0.38	0.90	0.81	0.46	0.28	0.39	0.90	0.83	0.41	0.35	0.44	0.97	0.88
Oscillospira	-0.49	0.34	0.61	0.69	0.86	-0.57	0.26	0.42	0.74	0.80	-0.54	0.31	0.40	0.90	0.81	-0.53	0.32	0.44	0.90	0.85	-0.50	0.36	0.45	0.97	0.88
Oxalobacter	0.62	0.13	0.78	0.46	0.90	0.40	0.32	0.69	0.77	0.93	0.35	0.42	0.71	0.91	0.91	0.34	0.44	0.74	0.92	0.93	0.25	0.58	0.89	0.97	0.97
Paludicola	0.32	0.46	0.33	0.76	0.78	0.17	0.70	0.24	0.93	0.77	-0.01	0.98	0.36	0.98	0.81	-0.08	0.86	0.36	0.96	0.83	-0.17	0.72	0.48	0.97	0.88
Parabacteroides	0.47	0.12	0.43	0.46	0.81	0.47	0.11	0.73	0.63	0.93	0.34	0.27	0.85	0.90	0.97	0.37	0.23	0.91	0.90	0.99	0.40	0.20	0.96	0.97	0.97
Paraprevotella	0.02	0.93	0.58	0.99	0.86	0.05	0.82	0.68	0.94	0.93	0.04	0.84	0.67	0.96	0.91	0.03	0.90	0.69	0.96	0.93	0.05	0.84	0.65	0.98	0.88
Parasutterella	0.31	0.16	0.67	0.47	0.87	0.25	0.25	0.54	0.74	0.87	0.13	0.57	0.39	0.91	0.81	0.14	0.55	0.39	0.92	0.83	0.09	0.71	0.34	0.97	0.87
Peptococcus	0.68	0.13	0.14	0.46	0.68	0.45	0.31	0.18	0.77	0.75	0.30	0.53	0.25	0.91	0.81	0.27	0.57	0.25	0.92	0.83	0.22	0.66	0.33	0.97	0.87
Phascolarctobacterium	0.48	0.02	0.07	0.25	0.68	0.48	0.02	0.09	0.36	0.75	0.47	0.03	0.18	0.76	0.81	0.46	0.04	0.18	0.89	0.80	0.45	0.04	0.18	0.87	0.75
Phocea	-0.46	0.35	0.67	0.69	0.87	-0.60	0.21	0.79	0.73	0.93	-0.79	0.12	0.96	0.85	1.00	-0.76	0.14	0.98	0.90	0.99	-0.68	0.19	0.91	0.97	0.97
Prevotella	0.13	0.48	0.55	0.77	0.84	0.17	0.33	0.75	0.78	0.93	0.17	0.38	0.75	0.91	0.91	0.15	0.42	0.84	0.92	0.95	0.17	0.38	0.77	0.97	0.92
Romboutsia	-0.05	0.89	0.26	0.96	0.68	0.04	0.92	0.26	0.96	0.77	-0.03	0.93	0.32	0.96	0.81	0.01	0.98	0.37	0.99	0.83	0.01	0.98	0.37	1.00	0.87
Roseburia	0.17	0.63	0.40	0.89	0.80	0.12	0.73	0.41	0.93	0.80	-0.15	0.70	0.24	0.96	0.81	-0.14	0.72	0.24	0.96	0.83	-0.07	0.86	0.29	0.98	0.87
Ruminococcus	0.52	0.03	0.55	0.29	0.84	0.38	0.11	0.62	0.63	0.93	0.27	0.30	0.71	0.90	0.91	0.26	0.32	0.73	0.90	0.93	0.22	0.41	0.84	0.97	0.96
Sellimonas	-0.09	0.81	0.11	0.92	0.68	-0.14	0.70	0.13	0.93	0.75	-0.21	0.57	0.12	0.91	0.81	-0.26	0.49	0.10	0.92	0.80	-0.29	0.44	0.08	0.97	0.75
Senegalimassilia	0.56	0.07	0.17	0.43	0.68	0.65	0.03	0.08	0.37	0.75	0.66	0.04	0.10	0.76	0.81	0.64	0.05	0.12	0.89	0.80	0.65	0.05	0.11	0.87	0.75
Shuttleworthia	0.55	0.20	0.25	0.51	0.68	0.22	0.60	0.33	0.93	0.78	0.38	0.40	0.12	0.91	0.81	0.35	0.44	0.13	0.92	0.80	0.31	0.50	0.18	0.97	0.75
Slackia	0.40	0.16	0.01	0.47	0.31	0.40	0.15	0.02	0.63	0.49	0.42	0.16	0.03	0.85	0.62	0.42	0.16	0.03	0.90	0.67	0.38	0.21	0.03	0.97	0.71
Streptococcus	0.26	0.54	0.91	0.83	0.94	0.20	0.63	0.96	0.93	0.97	0.17	0.70	0.66	0.96	0.91	0.21	0.64	0.78	0.96	0.94	0.21	0.65	0.73	0.97	0.91
Subdoligranulum	0.70	0.03	0.72	0.29	0.90	0.62	0.06	0.75	0.47	0.93	0.51	0.14	0.85	0.85	0.97	0.50	0.15	0.84	0.90	0.95	0.54	0.12	0.92	0.97	0.97
Sutterella	-0.15	0.45	0.60	0.76	0.86	-0.04	0.84	0.64	0.94	0.93	-0.08	0.69	0.50	0.96	0.87	-0.08	0.69	0.50	0.96	0.86	-0.06	0.78	0.54	0.98	0.88
TM7x	-0.77	0.09	0.07	0.44	0.68	-0.81	0.07	0.12	0.47	0.75	-0.64	0.18	0.23	0.85	0.81	-0.62	0.19	0.25	0.90	0.83	-0.67	0.17	0.19	0.97	0.75
Turicibacter	0.21	0.67	0.43	0.89	0.81	0.39	0.42	0.48	0.82	0.83	0.54	0.32	0.68	0.90	0.91	0.58	0.29	0.73	0.90	0.93	0.55	0.31	0.74	0.97	0.91
Tuzzerella	0.24	0.50	0.76	0.79	0.90	0.23	0.50	0.92	0.88	0.96	0.08	0.84	0.82	0.96	0.95	0.07	0.86	0.81	0.96	0.95	0.03	0.93	0.74	0.98	0.91
UBA1819	0.20	0.65	0.14	0.89	0.68	0.12	0.77	0.16	0.93	0.75	0.17	0.71	0.21	0.96	0.81	0.13	0.79	0.19	0.96	0.80	0.14	0.77	0.19	0.98	0.75
UCG.002	0.38	0.10	0.15	0.44	0.68	0.25	0.27	0.18	0.74	0.75	0.19	0.43	0.18	0.91	0.81	0.17	0.49	0.19	0.92	0.80	0.19	0.45	0.16	0.97	0.75
UCG.003	0.53	0.05	0.27	0.35	0.68	0.46	0.08	0.15	0.49	0.75	0.39	0.17	0.18	0.85	0.81	0.38	0.18	0.19	0.90	0.80	0.34	0.24	0.23	0.97	0.86
UCG.005	0.81	0.00	0.03	0.12	0.46	0.64	0.02	0.05	0.36	0.72	0.57	0.06	0.06	0.85	0.81	0.54	0.07	0.06	0.90	0.80	0.52	0.09	0.07	0.97	0.75
UCG.009	0.21	0.64	0.54	0.89	0.84	-0.04	0.93	0.57	0.97	0.90	-0.13	0.78	0.58	0.96	0.89	-0.11	0.82	0.52	0.96	0.87	-0.12	0.80	0.51	0.98	0.88
Veillonella	0.02	0.95	0.64	0.99	0.86	0.06	0.85	0.79	0.94	0.93	0.01	0.98	0.77	0.98	0.91	0.04	0.90	0.84	0.96	0.95	0.09	0.80	0.90	0.98	0.97
Victivallis	-0.03	0.93	0.25	0.99	0.68	-0.10	0.76	0.33	0.93	0.78	-0.29	0.41	0.28	0.91	0.81	-0.30	0.39	0.28	0.92	0.83	-0.35	0.33	0.20	0.97	0.76

Multivariable-adjusted regression models adjusted for:

Model 1: sequencing batch, age, race, gender, study center

Model 2: Model 1, with education

Model 3: Model 2, with physical activity, diet score, medication use, and smoking

Model 4: Model 3 with BMI

Model 5: Model 4 with hypertension and diabetes

© 2022 Meyer K et al. *JAMA Network Open.*

eTable 23: Multivariable-adjusted regression coefficients, p-values, race interaction p-values, false discovery rate (FDR)-adjusted p-values for race-genera interactions with respect to letter fluency.

	Model1					Model2					Model3					Model4					Model5				
	beta	p-value	p-val int	FDR	FDR-int	beta	p-value	p-val int	FDR	FDR-int	beta	p-value	p-val int	FDR	FDR-int	beta	p-value	p-val int	FDR	FDR-int	beta	p-value	p-val int	FDR	FDR-int
Acetanaerobacterium	0.36	0.82	0.97	0.94	0.99	0.33	0.83	0.68	0.98	0.90	0.35	0.83	0.68	1.00	0.97	0.34	0.84	0.69	0.99	0.99	0.50	0.76	0.86	0.98	1.00
Acidaminococcus	-0.28	0.55	0.17	0.82	0.81	-0.10	0.83	0.24	0.98	0.90	-0.01	0.98	0.40	1.00	0.89	0.00	1.00	0.40	1.00	0.89	0.00	0.99	0.41	1.00	0.89
Actinomyces	0.07	0.95	0.84	0.98	0.96	0.38	0.75	0.73	0.98	0.90	0.94	0.47	0.75	0.93	1.00	0.94	0.47	0.75	0.96	1.00	0.90	0.50	0.88	0.98	1.00
Adlercreutzia	0.00	1.00	0.25	1.00	0.82	-0.30	0.71	0.40	0.98	0.90	0.00	1.00	0.17	1.00	0.89	0.00	1.00	0.17	1.00	0.86	-0.02	0.99	0.16	1.00	0.89
Agathobacter	0.41	0.71	0.77	0.88	0.93	0.32	0.77	0.79	0.98	0.90	-0.27	0.81	0.97	1.00	1.00	-0.24	0.84	0.96	0.99	1.00	-0.12	0.91	0.99	1.00	1.00
Akkermansia	0.70	0.20	0.11	0.59	0.81	0.38	0.48	0.19	0.93	0.90	0.30	0.60	0.22	1.00	0.89	0.31	0.59	0.21	0.96	0.86	0.19	0.75	0.29	0.98	0.89
Alistipes	0.82	0.38	0.99	0.76	0.99	0.44	0.63	0.82	0.98	0.92	0.07	0.94	0.87	1.00	1.00	0.10	0.92	0.90	0.99	1.00	0.08	0.93	0.94	1.00	1.00
Allisonella	-0.16	0.85	0.63	0.94	0.92	0.74	0.39	0.84	0.87	0.92	0.87	0.36	0.81	0.93	1.00	0.85	0.38	0.75	0.96	1.00	0.91	0.35	0.75	0.98	1.00
Anaerofilum	1.52	0.22	0.41	0.59	0.86	0.78	0.52	0.47	0.93	0.90	1.10	0.39	0.28	0.93	0.89	1.07	0.41	0.28	0.96	0.86	0.90	0.49	0.33	0.98	0.89
Anaerostipes	-1.03	0.36	0.48	0.76	0.88	-1.38	0.21	0.42	0.78	0.90	-0.89	0.44	0.60	0.93	0.94	-0.86	0.46	0.63	0.96	0.96	-0.77	0.52	0.64	0.98	0.98
Anaerotruncus	0.75	0.47	0.20	0.78	0.81	0.48	0.63	0.22	0.98	0.90	0.93	0.39	0.09	0.93	0.89	0.87	0.41	0.08	0.96	0.86	0.87	0.42	0.08	0.98	0.89
Bacteroides	1.32	0.39	0.17	0.76	0.81	0.98	0.51	0.18	0.93	0.90	0.76	0.61	0.25	1.00	0.89	0.87	0.57	0.24	0.96	0.86	0.92	0.55	0.20	0.98	0.89
Barnesiella	1.16	0.03	0.75	0.42	0.93	1.24	0.02	0.61	0.72	0.90	0.96	0.09	0.92	0.93	1.00	0.94	0.09	0.93	0.96	1.00	0.93	0.10	1.00	0.98	1.00
Bifidobacterium	0.71	0.31	0.15	0.71	0.81	0.98	0.15	0.17	0.74	0.90	0.83	0.25	0.24	0.93	0.89	0.87	0.23	0.22	0.96	0.86	0.89	0.22	0.21	0.98	0.89
Bilophila	1.10	0.11	0.90	0.59	0.98	0.68	0.31	0.71	0.87	0.90	0.57	0.43	0.61	0.93	0.94	0.61	0.40	0.65	0.96	0.96	0.48	0.51	0.58	0.98	0.94
Blautia	0.04	0.99	0.19	1.00	0.81	-0.42	0.85	0.16	0.98	0.90	0.23	0.92	0.13	1.00	0.89	0.32	0.89	0.11	0.99	0.86	0.86	0.72	0.11	0.98	0.89
Butyricicoccus	2.12	0.16	0.07	0.59	0.81	1.04	0.49	0.11	0.93	0.90	0.98	0.53	0.05	0.99	0.89	1.04	0.50	0.05	0.96	0.86	0.96	0.54	0.06	0.98	0.83
Butyricimonas	1.13	0.05	0.14	0.44	0.81	1.01	0.08	0.12	0.72	0.90	1.15	0.05	0.06	0.93	0.89	1.14	0.05	0.06	0.96	0.86	1.20	0.04	0.04	0.98	0.80
CAG.56	0.06	0.92	0.51	0.98	0.88	0.04	0.95	0.72	0.98	0.90	0.25	0.72	0.97	1.00	1.00	0.27	0.70	1.00	0.99	1.00	0.23	0.75	0.92	0.98	1.00
Candidatus_Soleaferrea	0.74	0.60	0.34	0.86	0.86	0.50	0.71	0.30	0.98	0.90	0.53	0.71	0.42	1.00	0.89	0.54	0.71	0.41	0.99	0.89	0.69	0.63	0.31	0.98	0.89
Caproiciproducens	1.80	0.13	0.14	0.59	0.81	1.64	0.15	0.19	0.74	0.90	0.89	0.47	0.40	0.93	0.89	0.87	0.48	0.40	0.96	0.89	0.73	0.56	0.45	0.98	0.89
Christensenellaceae_R.7_group	1.12	0.06	0.19	0.44	0.81	0.75	0.19	0.32	0.74	0.90	0.45	0.46	0.39	0.93	0.89	0.41	0.50	0.41	0.96	0.89	0.33	0.60	0.50	0.98	0.89
Clostridium_sensu_stricto_1	-0.91	0.25	0.07	0.61	0.81	-0.70	0.36	0.09	0.87	0.90	-0.75	0.36	0.10	0.93	0.89	-0.73	0.37	0.10	0.96	0.86	-0.80	0.33	0.11	0.98	0.89
Colidextribacter	0.45	0.73	0.89	0.88	0.98	0.33	0.79	0.69	0.98	0.90	0.31	0.81	0.40	1.00	0.89	0.33	0.80	0.37	0.99	0.89	0.45	0.73	0.37	0.98	0.89
Collinsella	-0.05	0.94	0.67	0.98	0.92	0.43	0.48	0.54	0.93	0.90	0.10	0.88	0.92	1.00	1.00	0.10	0.88	0.95	0.99	1.00	-0.01	0.99	0.95	1.00	1.00
Coprobacter	1.14	0.15	0.70	0.59	0.92	0.92	0.23	0.72	0.83	0.90	0.47	0.57	0.88	1.00	1.00	0.44	0.59	0.89	0.96	1.00	0.39	0.64	0.97	0.98	1.00
Coprococcus	0.28	0.70	0.35	0.88	0.86	0.44	0.54	0.57	0.95	0.90	0.70	0.36	0.99	0.93	1.00	0.70	0.36	0.99	0.96	1.00	0.80	0.30	0.90	0.98	1.00
Defluviitaleaceae_UCG.011	1.40	0.22	0.23	0.59	0.82	1.04	0.35	0.27	0.87	0.90	1.16	0.33	0.24	0.93	0.89	1.11	0.35	0.24	0.96	0.86	0.99	0.41	0.25	0.98	0.89
Desulfovibrio	0.34	0.56	0.20	0.84	0.81	0.41	0.48	0.19	0.93	0.90	0.45	0.46	0.14	0.93	0.89	0.41	0.51	0.15	0.96	0.86	0.40	0.52	0.16	0.98	0.89
Dialister	-0.82	0.10	0.21	0.55	0.81	-0.75	0.12	0.26	0.72	0.90	-0.55	0.29	0.27	0.93	0.89	-0.55	0.29	0.27	0.96	0.86	-0.51	0.33	0.30	0.98	0.89
DNF00809	-0.97	0.45	0.73	0.78	0.92	-1.07	0.40	0.78	0.87	0.90	-0.81	0.54	0.93	0.99	1.00	-0.83	0.53	0.92	0.96	1.00	-0.91	0.50	0.96	0.98	1.00
Dorea	0.42	0.64	0.30	0.87	0.85	0.45	0.60	0.36	0.98	0.90	0.50	0.57	0.41	1.00	0.89	0.51	0.56	0.42	0.96	0.89	0.42	0.63	0.47	0.98	0.89
DTU089	1.15	0.25	0.26	0.61	0.83	0.79	0.41	0.42	0.89	0.90	0.40	0.69	0.61	1.00	0.94	0.40	0.69	0.59	0.99	0.96	0.56	0.58	0.55	0.98	0.93
Eggerthella	-1.15	0.22	0.71	0.59	0.92	-1.48	0.10	0.50	0.72	0.90	-0.92	0.35	0.75	0.93	1.00	-0.89	0.36	0.80	0.96	1.00	-0.86	0.38	0.74	0.98	1.00
Eisenbergiella	-1.21	0.18	0.66	0.59	0.92	-1.23	0.16	0.62	0.74	0.90	-1.17	0.21	0.85	0.93	1.00	-1.21	0.20	0.89	0.96	1.00	-1.11	0.24	0.87	0.98	1.00
Erysipelatoclostridium	0.20	0.83	0.34	0.94	0.86	0.08	0.93	0.45	0.98	0.90	0.31	0.75	0.25	1.00	0.89	0.29	0.77	0.24	0.99	0.86	0.42	0.67	0.18	0.98	0.89
Erysipelotrichaceae_UCG.003	0.98	0.18	0.27	0.59	0.83	0.96	0.18	0.22	0.74	0.90	0.54	0.48	0.43	0.93	0.89	0.52	0.49	0.44	0.96	0.89	0.45	0.55	0.45	0.98	0.89
Escherichia.Shigella	-0.56	0.40	0.86	0.76	0.97	-0.11	0.86	0.54	0.98	0.90	-0.07	0.92	0.63	1.00	0.94	-0.05	0.94	0.62	0.99	0.96	-0.03	0.97	0.58	1.00	0.94
Faecalibacterium	0.78	0.52	0.81	0.80	0.95	0.16	0.89	0.89	0.98	0.93	0.03	0.98	0.94	1.00	1.00	0.06	0.96	0.95	0.99	1.00	0.14	0.91	0.97	1.00	1.00
Family_XIII_AD3011_group	1.31	0.16	0.46	0.59	0.88	0.81	0.38	0.56	0.87	0.90	0.85	0.39	0.37	0.93	0.89	0.79	0.43	0.38	0.96	0.89	0.66	0.51	0.45	0.98	0.89
Family_XIII_UCG.001	1.47	0.20	0.51	0.59	0.88	1.26	0.26	0.45	0.86	0.90	1.36	0.25	0.32	0.93	0.89	1.30	0.28	0.32	0.96	0.89	1.23	0.31	0.36	0.98	0.89
FD2005	-0.75	0.47	0.66	0.78	0.92	-0.13	0.90	0.88	0.98	0.93	0.06	0.95	0.79	1.00	1.00	0.05	0.97	0.82	0.99	1.00	0.08	0.94	0.74	1.00	1.00
Flavonifractor	-0.39	0.66	0.48	0.87	0.88	-0.50	0.57	0.61	0.95	0.90	-0.41	0.66	0.62	1.00	0.94	-0.38	0.68	0.60	0.99	0.96	-0.28	0.77	0.53	0.98	0.90
Fournierella	0.48	0.66	0.65	0.87	0.92	0.28	0.79	0.78	0.98	0.90	0.37	0.74	0.45	1.00	0.90	0.31	0.78	0.47	0.99	0.90	0.32	0.78	0.48	0.98	0.89
Frisingicoccus	0.47	0.57	0.53	0.84	0.89	0.15	0.86	0.69	0.98	0.90	-0.06	0.95	0.70	1.00	0.98	-0.07	0.93	0.69	0.99	0.99	-0.16	0.86	0.84	1.00	1.00
Fusicatenibacter	0.14	0.85	0.78	0.94	0.93	-0.06	0.94	0.79	0.98	0.90	-0.61	0.43	0.39	0.93	0.89	-0.57	0.46	0.39	0.96	0.89	-0.82	0.30	0.27	0.98	0.89
Fusobacterium	0.62	0.49	0.69	0.79	0.92	0.93	0.28	0.82	0.87	0.92	1.44	0.12	0.76	0.93	1.00	1.41	0.13	0.76	0.96	1.00	1.57	0.09	0.52	0.98	0.90
GCA.900066575	1.74	0.10	0.39	0.55	0.86	1.34	0.19	0.38	0.74	0.90	1.14	0.29	0.36	0.93	0.89	1.10	0.32	0.36	0.96	0.89	1.15	0.29	0.25	0.98	0.89
Gordonibacter	1.79	0.12	0.17	0.59	0.81	1.06	0.35	0.40	0.87	0.90	1.31	0.27	0.26	0.93	0.89	1.27	0.29	0.25	0.96	0.86	1.14	0.34	0.30	0.98	0.89
Granulicatella	0.08	0.95	0.99	0.98	0.99	0.41	0.74	0.73	0.98	0.90	0.95	0.46	0.68	0.93	0.97	1.03	0.42	0.64	0.96	0.96	1.14	0.38	0.67	0.98	1.00
Haemophilus	0.20	0.79	0.63	0.92	0.92	0.07	0.93	0.66	0.98	0.90	-0.08	0.92	0.62	1.00	0.94	-0.06	0.94	0.65	0.99	0.96	-0.04	0.96	0.64	1.00	0.98
Harryflintia	-0.92	0.42	0.94	0.76	0.99	-1.00	0.37	0.98	0.87	0.98	-1.27	0.29	0.87	0.93	1.00	-1.31	0.28	0.87	0.96	1.00	-1.44	0.24	0.92	0.98	1.00
Holdemanella	0.45	0.52	0.49	0.80	0.88	0.68	0.32	0.36	0.87	0.90	1.24	0.09	0.22	0.93	0.89	1.23	0.09	0.24	0.96	0.86	1.30	0.08	0.23	0.98	0.89
Holdemania	-2.47	0.22	0.11	0.59	0.81	-2.57	0.19	0.09	0.74	0.90	-2.29	0.28	0.14	0.93	0.89	-2.33	0.27	0.14	0.96	0.86	-2.56	0.23	0.16	0.98	0.89
Hungatella	-1.28	0.15	0.92	0.59	0.98	-1.21	0.16	0.98	0.74	0.98	-1.10	0.24	0.92	0.93	1.00	-1.07	0.25	0.87	0.96	1.00	-0.97	0.30	0.90	0.98	1.00
Hydrogenoanaerobacterium	2.34	0.03	0.30	0.42	0.85	1.65	0.12	0.43	0.72	0.90	1.60	0.16	0.38	0.93	0.89	1.55	0.18	0.39	0.96	0.89	1.44	0.21	0.43	0.98	0.89
Incertae_Sedis	0.67	0.65	0.86	0.87	0.97	0.54	0.70	0.95	0.98	0.97	0.66	0.66	0.96	1.00	1.00	0.70	0.65	1.00	0.99	1.00	0.63	0.68	0.93	0.98	1.00
Intestinibacter	-0.38	0.66	0.52	0.87	0.88	-0.25	0.77	0.38	0.98	0.90	-0.11	0.90	0.67	1.00	0.97	-0.04	0.97	0.70	0.99	0.99	0.10	0.92	0.80	1.00	1.00
Intestinimonas	1.00	0.24	0.38	0.61	0.86	0.50	0.55	0.47	0.95	0.90	0.50	0.58	0.43	1.00	0.89	0.48	0.60	0.41	0.96	0.89	0.50	0.58	0.50	0.98	0.89
Lachnoclostridium	-1.84	0.31	0.88	0.71	0.98	-0.91	0.60	0.74	0.98	0.90	0.59	0.75	0.47	1.00	0.92	0.66	0.72	0.47	0.99	0.90	0.88	0.63	0.42	0.98	0.89
Lachnospira	0.75	0.34	0.40	0.75	0.86	0.29	0.70	0.54	0.98	0.90	0.16	0.85	0.70	1.00	0.98	0.20	0.81	0.73	0.99	1.00	0.28	0.74	0.81	0.98	1.00
Lachnospiraceae_FCS020_group	2.66	0.01	0.14	0.35	0.81	2.13	0.02	0.15	0.72	0.90	2.14	0.03	0.16	0.93	0.89	2.14	0.03	0.16	0.96	0.86	2.06	0.04	0.18	0.98	0.89
Lachnospiraceae_ND3007_group	1.18	0.07	0.42	0.44	0.87	1.02	0.10	0.29	0.72	0.90	1.01	0.13	0.25	0.93	0.89	1.04	0.12	0.25	0.96	0.86	0.98	0.15	0.28	0.98	0.89
Lachnospiraceae_NK4A136_group	0.22	0.79	0.71	0.92	0.92	-0.35	0.68	0.59	0.98	0.90	-0.56	0.53	0.99	0.99	1.00	-0.55	0.54	0.99	0.96	1.00	-0.55	0.55	0.98	0.98	1.00
Lachnospiraceae_UCG.001	0.26	0.70	0.59	0.88	0.92	0.03	0.96	0.69	0.98	0.90	-0.02	0.98	0.63	1.00	0.94	-0.06	0.93	0.66	0.99	0.96	-0.14	0.85	0.74	1.00	1.00
Lachnospiraceae_UCG.004	0.16	0.83	0.70	0.94	0.92	0.02	0.98	0.73	0.98	0.90	0.09	0.91	0.77	1.00	1.00	0.11	0.89	0.76	0.99	1.00	0.03	0.97	0.86	1.00	1.00
Lachnospiraceae_UCG.010	-0.72	0.48	0.38	0.79	0.86	-1.04	0.30	0.40	0.87	0.90	-0.92	0.38	0.58	0.93	0.94	-0.96	0.36	0.57	0.96	0.96	-0.94	0.37	0.60	0.98	0.96
Lactobacillus	-0.49	0.50	0.61	0.79	0.92	-0.11	0.88	0.77	0.98	0.90	-0.24	0.76	0.87	1.00	1.00	-0.20	0.80	0.86	0.99	1.00	-0.31	0.70	0.92	0.98	1.00
Lactococcus	2.50	0.05	0.10	0.44	0.81	1.91	0.13	0.20	0.73	0.90	2.02	0.14	0.28	0.93	0.89	2.04	0.13	0.27	0.96	0.86	1.86	0.17	0.30	0.98	0.89
Marvinbryantia	0.57	0.42	0.30	0.76	0.85	0.35	0.61	0.28	0.98	0.90	0.06	0.93	0.54	1.00	0.94	0.07	0.93	0.53	0.99	0.92	0.16	0.83	0.48	1.00	0.89
Megasphaera	-0.34	0.50	0.68	0.79	0.92	0.01	0.98	0.74	0.98	0.90	0.29	0.59	0.50	1.00	0.93	0.28	0.60	0.47	0.96	0.90	0.22	0.68	0.43	0.98	0.89

© 2022 Meyer K et al. *JAMA Network Open.*

Monoglobus	1.02	0.21	0.97	0.59	0.99	0.63	0.43	0.73	0.90	0.90	0.35	0.69	0.48	1.00	0.92	0.34	0.69	0.50	0.99	0.92	0.18	0.83	0.37	1.00	0.89
Moryella	1.20	0.34	0.70	0.75	0.92	0.80	0.51	0.69	0.93	0.90	0.19	0.89	0.91	1.00	1.00	0.18	0.90	0.92	0.99	1.00	0.06	0.96	1.00	1.00	1.00
Negativibacillus	1.13	0.13	0.05	0.59	0.81	1.30	0.07	0.02	0.72	0.66	1.21	0.12	0.02	0.93	0.77	1.19	0.12	0.02	0.96	0.84	1.14	0.14	0.02	0.98	0.80
NK4A214_group	0.78	0.20	0.15	0.59	0.81	0.57	0.34	0.14	0.87	0.90	0.20	0.76	0.26	1.00	0.89	0.15	0.81	0.28	0.99	0.86	0.08	0.90	0.35	1.00	0.89
Odoribacter	1.26	0.02	0.44	0.42	0.87	0.97	0.07	0.75	0.72	0.90	0.50	0.38	1.00	0.93	1.00	0.49	0.39	0.99	0.96	1.00	0.44	0.45	1.00	0.98	1.00
Oscillibacter	0.40	0.69	0.76	0.88	0.93	0.28	0.77	0.64	0.98	0.90	0.45	0.66	0.54	1.00	0.94	0.40	0.70	0.55	0.99	0.95	0.25	0.82	0.63	1.00	0.98
Oscillospira	1.86	0.15	0.41	0.59	0.86	1.64	0.19	0.56	0.74	0.90	1.59	0.22	0.55	0.93	0.94	1.60	0.22	0.53	0.96	0.92	1.79	0.18	0.47	0.98	0.89
Oxalobacter	1.35	0.19	0.58	0.59	0.92	0.84	0.40	0.50	0.87	0.90	0.85	0.43	0.33	0.93	0.89	0.83	0.44	0.34	0.96	0.89	0.77	0.48	0.41	0.98	0.89
Paludicola	1.44	0.18	0.50	0.59	0.88	1.04	0.32	0.43	0.87	0.90	0.68	0.55	0.52	0.99	0.94	0.61	0.59	0.52	0.96	0.92	0.50	0.67	0.71	0.98	1.00
Parabacteroides	1.38	0.07	0.23	0.44	0.82	1.34	0.07	0.45	0.72	0.90	0.87	0.25	0.86	0.93	1.00	0.91	0.23	0.83	0.96	1.00	0.96	0.21	0.77	0.98	1.00
Paraprevotella	-0.05	0.93	0.77	0.98	0.93	0.03	0.95	0.66	0.98	0.90	-0.21	0.71	0.45	1.00	0.90	-0.23	0.68	0.44	0.99	0.89	-0.23	0.69	0.44	0.98	0.89
Parasutterella	0.78	0.16	0.79	0.59	0.93	0.63	0.24	0.91	0.83	0.95	0.47	0.41	0.83	0.93	1.00	0.48	0.40	0.83	0.96	1.00	0.40	0.50	0.83	0.98	1.00
Peptococcus	2.55	0.02	0.01	0.42	0.31	2.05	0.06	0.01	0.72	0.47	1.63	0.16	0.01	0.93	0.68	1.59	0.17	0.01	0.96	0.78	1.50	0.20	0.01	0.98	0.79
Phascolarctobacterium	1.39	0.01	0.10	0.35	0.81	1.40	0.01	0.12	0.56	0.90	1.55	0.00	0.06	0.38	0.89	1.53	0.00	0.06	0.43	0.86	1.62	0.00	0.04	0.29	0.80
Phocea	0.22	0.86	0.78	0.94	0.93	-0.07	0.96	0.87	0.98	0.93	0.18	0.88	0.62	1.00	0.94	0.22	0.86	0.61	0.99	0.96	0.39	0.75	0.47	0.98	0.89
Prevotella	-0.36	0.42	0.67	0.76	0.92	-0.25	0.57	0.49	0.95	0.90	-0.22	0.63	0.80	1.00	1.00	-0.24	0.60	0.75	0.96	1.00	-0.21	0.65	0.81	0.98	1.00
Romboutsia	-1.56	0.07	0.01	0.44	0.31	-1.33	0.11	0.01	0.72	0.47	-1.46	0.10	0.01	0.93	0.68	-1.42	0.11	0.01	0.96	0.78	-1.51	0.10	0.01	0.98	0.79
Roseburia	0.46	0.61	0.99	0.87	0.99	0.32	0.72	0.97	0.98	0.98	-0.02	0.99	0.94	1.00	1.00	0.00	1.00	0.93	1.00	1.00	0.16	0.87	0.95	1.00	1.00
Ruminococcus	0.54	0.37	0.34	0.76	0.86	0.20	0.73	0.39	0.98	0.90	0.14	0.82	0.27	1.00	0.89	0.13	0.84	0.28	0.99	0.86	0.00	1.00	0.39	1.00	0.89
Sellimonas	0.12	0.90	0.39	0.97	0.86	0.04	0.96	0.46	0.98	0.90	-0.65	0.48	0.20	0.93	0.89	-0.72	0.44	0.19	0.96	0.86	-0.77	0.40	0.16	0.98	0.89
Senegalimassilia	0.63	0.42	0.90	0.76	0.98	0.84	0.27	0.83	0.86	0.92	0.78	0.33	0.88	0.93	1.00	0.75	0.35	0.92	0.96	1.00	0.75	0.35	0.93	0.98	1.00
Shuttleworthia	0.79	0.46	0.35	0.78	0.86	0.05	0.96	0.43	0.98	0.90	-0.31	0.78	0.59	1.00	0.94	-0.36	0.74	0.60	0.99	0.96	-0.46	0.68	0.74	0.98	1.00
Slackia	0.25	0.72	0.51	0.88	0.88	0.28	0.68	0.63	0.98	0.90	0.38	0.61	0.51	1.00	0.94	0.37	0.61	0.52	0.96	0.92	0.33	0.65	0.46	0.98	0.89
Streptococcus	0.02	0.99	0.25	1.00	0.82	-0.09	0.93	0.29	0.98	0.90	0.50	0.65	0.30	1.00	0.89	0.57	0.60	0.25	0.96	0.86	0.44	0.69	0.34	0.98	0.89
Subdoligranulum	1.54	0.06	0.25	0.44	0.82	1.32	0.10	0.23	0.72	0.90	1.02	0.23	0.23	0.93	0.89	1.01	0.24	0.23	0.96	0.86	1.05	0.22	0.20	0.98	0.89
Sutterella	-0.16	0.75	0.96	0.89	0.99	0.11	0.82	0.88	0.98	0.93	-0.05	0.92	0.85	1.00	1.00	-0.05	0.92	0.85	0.99	1.00	0.01	0.99	0.96	1.00	1.00
TM7x	-0.92	0.42	0.68	0.76	0.92	-1.06	0.34	0.49	0.87	0.90	-0.21	0.86	0.28	1.00	0.89	-0.17	0.88	0.26	0.99	0.86	-0.32	0.79	0.36	0.98	0.89
Turicibacter	-0.52	0.67	0.18	0.87	0.81	-0.10	0.94	0.21	0.98	0.90	0.37	0.78	0.41	1.00	0.89	0.42	0.75	0.43	0.99	0.89	0.41	0.76	0.52	0.98	0.90
Tuzzerella	1.80	0.04	0.01	0.44	0.31	1.73	0.04	0.01	0.72	0.47	1.37	0.15	0.03	0.93	0.87	1.36	0.15	0.03	0.96	0.86	1.28	0.17	0.03	0.98	0.80
UBA1819	0.99	0.37	0.37	0.76	0.86	0.77	0.48	0.44	0.93	0.90	1.15	0.32	0.32	0.93	0.89	1.10	0.34	0.31	0.96	0.89	1.21	0.30	0.28	0.98	0.89
UCG.002	1.25	0.03	0.04	0.42	0.81	0.96	0.08	0.04	0.72	0.90	0.56	0.35	0.07	0.93	0.89	0.53	0.38	0.07	0.96	0.86	0.55	0.36	0.06	0.98	0.83
UCG.003	1.25	0.06	0.44	0.44	0.87	1.08	0.09	0.30	0.72	0.90	0.92	0.18	0.31	0.93	0.89	0.91	0.19	0.31	0.96	0.89	0.78	0.26	0.36	0.98	0.89
UCG.005	1.67	0.02	0.11	0.42	0.81	1.28	0.06	0.19	0.72	0.90	1.14	0.12	0.17	0.93	0.89	1.10	0.13	0.17	0.96	0.86	1.02	0.17	0.22	0.98	0.89
UCG.009	0.43	0.70	0.72	0.88	0.92	-0.13	0.91	0.76	0.98	0.90	-0.96	0.42	0.94	0.93	1.00	-0.93	0.43	0.98	0.96	1.00	-1.04	0.38	0.89	0.98	1.00
Veillonella	-0.81	0.29	0.12	0.70	0.81	-0.67	0.37	0.18	0.87	0.90	-0.63	0.44	0.17	0.93	0.89	-0.59	0.47	0.18	0.96	0.86	-0.62	0.45	0.17	0.98	0.89
Victivallis	-0.40	0.62	0.12	0.87	0.81	-0.53	0.50	0.17	0.93	0.90	-1.06	0.21	0.19	0.93	0.89	-1.08	0.20	0.19	0.96	0.86	-1.13	0.19	0.14	0.98	0.89

Multivariable-adjusted regression models adjusted for:

Model 1: sequencing batch, age, race, gender, study center

Model 2: Model 1, with education

Model 3: Model 2, with physical activity, diet score, medication use, and smoking

Model 4: Model 3 with BMI

Model 5: Model 4 with hypertension and diabetes

© 2022 Meyer K et al. *JAMA Network Open.*

eTable 24: Multivariable-adjusted regression coefficients, p-values, race interaction p-values, false discovery rate (FDR)-adjusted p-values for race-genera interactions with respect to MOCA.

	Model1					Model2					Model3					Model4					Model5				
	beta	p-value	p-val int	FDR	FDR-int	beta	p-value	p-val int	FDR	FDR-int	beta	p-value	p-val int	FDR	FDR-int	beta	p-value	p-val int	FDR	FDR-int	beta	p-value	p-val int	FDR	FDR-int
Acetanaerobacterium	-0.13	0.76	0.56	0.84	0.85	-0.13	0.75	0.94	0.97	0.98	-0.24	0.56	0.97	0.87	1.00	-0.24	0.55	0.96	0.87	0.99	-0.20	0.64	0.79	0.90	0.91
Acidaminococcus	-0.21	0.09	0.31	0.25	0.76	-0.13	0.25	0.50	0.60	0.89	-0.08	0.52	0.27	0.87	0.87	-0.08	0.52	0.27	0.87	0.87	-0.05	0.66	0.22	0.90	0.83
Actinomyces	-0.43	0.19	0.17	0.40	0.76	-0.30	0.32	0.20	0.65	0.86	-0.31	0.35	0.13	0.77	0.79	-0.31	0.35	0.13	0.76	0.79	-0.24	0.46	0.14	0.83	0.74
Adlercreutzia	0.21	0.35	0.23	0.57	0.76	0.09	0.66	0.44	0.94	0.89	0.01	0.95	0.57	0.99	0.87	0.01	0.95	0.56	0.98	0.87	0.04	0.84	0.45	0.97	0.85
Agathobacter	-0.47	0.10	0.30	0.26	0.76	-0.53	0.05	0.22	0.39	0.86	-0.54	0.04	0.27	0.37	0.87	-0.54	0.05	0.27	0.38	0.87	-0.53	0.06	0.27	0.41	0.85
Akkermansia	0.40	0.01	0.07	0.07	0.59	0.27	0.04	0.14	0.39	0.79	0.20	0.18	0.35	0.61	0.87	0.20	0.18	0.34	0.61	0.87	0.17	0.24	0.40	0.67	0.85
Alistipes	0.32	0.20	0.74	0.40	0.92	0.18	0.45	0.99	0.73	1.00	0.09	0.70	0.94	0.91	0.99	0.09	0.69	0.93	0.90	0.99	0.10	0.69	0.84	0.92	0.94
Allisonella	-0.42	0.07	0.48	0.23	0.80	-0.07	0.75	0.74	0.97	0.97	-0.08	0.73	0.84	0.91	0.94	-0.08	0.73	0.82	0.91	0.93	-0.11	0.65	0.76	0.90	0.91
Anaerofilum	0.36	0.28	0.41	0.48	0.76	0.05	0.88	0.49	0.99	0.89	0.05	0.89	0.49	0.98	0.87	0.04	0.90	0.49	0.98	0.87	0.05	0.89	0.49	0.97	0.85
Anaerostipes	0.12	0.68	0.53	0.81	0.83	-0.02	0.95	0.42	0.99	0.89	0.00	0.99	0.55	0.99	0.87	0.01	0.98	0.56	0.99	0.87	0.05	0.86	0.63	0.97	0.87
Anaerotruncus	0.53	0.05	0.08	0.20	0.59	0.43	0.09	0.08	0.39	0.70	0.65	0.02	0.02	0.23	0.58	0.64	0.02	0.02	0.24	0.59	0.69	0.01	0.01	0.17	0.48
Bacteroides	0.14	0.73	0.58	0.83	0.86	0.01	0.97	0.64	1.00	0.93	0.01	0.98	0.59	0.99	0.87	0.02	0.96	0.58	0.98	0.87	0.04	0.91	0.53	0.97	0.85
Barnesiella	-0.02	0.89	0.11	0.91	0.63	0.00	0.97	0.14	1.00	0.79	0.09	0.53	0.38	0.87	0.87	0.09	0.54	0.38	0.87	0.87	0.08	0.57	0.30	0.89	0.85
Bifidobacterium	-0.08	0.68	0.80	0.81	0.93	0.02	0.93	1.00	0.99	1.00	0.02	0.89	0.90	0.98	0.97	0.03	0.87	0.88	0.98	0.96	0.00	0.99	0.98	0.99	0.98
Bilophila	0.29	0.11	0.28	0.28	0.76	0.14	0.43	0.67	0.73	0.93	-0.04	0.84	0.76	0.98	0.91	-0.03	0.85	0.77	0.98	0.93	-0.01	0.96	0.88	0.97	0.95
Blautia	-0.34	0.57	0.47	0.75	0.78	-0.49	0.37	0.51	0.69	0.89	-0.26	0.65	0.69	0.87	0.89	-0.26	0.65	0.71	0.88	0.90	-0.02	0.97	0.88	0.98	0.95
Butyricicoccus	0.28	0.50	0.93	0.70	1.00	-0.16	0.68	0.65	0.95	0.93	-0.31	0.43	0.68	0.81	0.89	-0.31	0.43	0.69	0.82	0.90	-0.32	0.42	0.68	0.83	0.88
Butyricimonas	0.13	0.39	0.90	0.59	0.99	0.07	0.62	0.89	0.92	0.98	0.18	0.22	0.49	0.61	0.87	0.18	0.22	0.50	0.63	0.87	0.16	0.29	0.51	0.70	0.85
CAG.56	-0.11	0.54	0.12	0.73	0.63	-0.13	0.44	0.23	0.73	0.86	-0.09	0.60	0.40	0.87	0.87	-0.09	0.61	0.41	0.88	0.87	-0.08	0.66	0.40	0.90	0.85
Candidatus_Soleaferrea	0.08	0.84	0.33	0.88	0.76	0.00	0.99	0.23	1.00	0.86	0.04	0.91	0.25	0.98	0.87	0.04	0.91	0.25	0.98	0.87	0.08	0.84	0.20	0.97	0.83
Caproiciproducens	0.13	0.69	0.38	0.81	0.76	0.05	0.86	0.55	0.99	0.89	-0.02	0.95	0.70	0.99	0.89	-0.02	0.94	0.70	0.98	0.90	0.02	0.94	0.59	0.97	0.87
Christensenellaceae_R.7_group	0.42	0.01	0.23	0.08	0.76	0.27	0.07	0.46	0.39	0.89	0.27	0.08	0.45	0.48	0.87	0.27	0.08	0.45	0.49	0.87	0.30	0.06	0.38	0.41	0.85
Clostridium_sensu_stricto_1	-0.20	0.34	0.11	0.56	0.63	-0.13	0.52	0.15	0.80	0.79	-0.16	0.44	0.10	0.81	0.77	-0.16	0.44	0.10	0.82	0.76	-0.14	0.51	0.13	0.85	0.74
Colidextribacter	-0.30	0.40	0.58	0.59	0.86	-0.33	0.32	0.88	0.65	0.98	-0.15	0.65	0.76	0.87	0.91	-0.15	0.65	0.74	0.88	0.92	-0.07	0.83	0.65	0.97	0.87
Collinsella	-0.19	0.25	0.42	0.46	0.76	0.00	1.00	0.54	1.00	0.89	-0.02	0.91	0.58	0.98	0.87	-0.02	0.90	0.57	0.98	0.87	-0.03	0.86	0.57	0.97	0.85
Coprobacter	0.33	0.11	0.15	0.28	0.69	0.26	0.18	0.11	0.47	0.79	0.28	0.16	0.05	0.60	0.72	0.28	0.16	0.05	0.59	0.72	0.28	0.17	0.06	0.58	0.68
Coprococcus	-0.13	0.53	0.22	0.73	0.76	-0.06	0.76	0.48	0.97	0.89	0.11	0.58	0.79	0.87	0.93	0.11	0.58	0.79	0.88	0.93	0.11	0.56	0.77	0.89	0.91
Defluviitaleaceae_UCG.011	0.75	0.01	0.07	0.11	0.59	0.63	0.03	0.07	0.29	0.70	0.61	0.04	0.11	0.37	0.77	0.61	0.04	0.11	0.38	0.76	0.62	0.04	0.09	0.39	0.68
Desulfovibrio	-0.04	0.80	0.40	0.85	0.76	-0.02	0.91	0.36	0.99	0.89	0.07	0.66	0.20	0.87	0.84	0.06	0.69	0.21	0.90	0.87	0.06	0.72	0.24	0.93	0.85
Dialister	-0.25	0.06	0.05	0.22	0.59	-0.21	0.08	0.07	0.39	0.70	-0.20	0.12	0.14	0.59	0.79	-0.20	0.12	0.14	0.59	0.79	-0.19	0.16	0.16	0.58	0.74
DNF00809	-0.15	0.66	0.19	0.81	0.76	-0.17	0.59	0.22	0.90	0.86	0.15	0.65	0.60	0.87	0.87	0.15	0.65	0.60	0.88	0.87	0.16	0.63	0.62	0.90	0.87
Dorea	0.17	0.45	0.30	0.65	0.76	0.19	0.39	0.39	0.70	0.89	0.26	0.24	0.27	0.62	0.87	0.26	0.24	0.27	0.63	0.87	0.24	0.27	0.31	0.70	0.85
DTU089	0.44	0.10	0.15	0.26	0.69	0.29	0.24	0.31	0.60	0.89	0.24	0.35	0.56	0.77	0.87	0.24	0.35	0.55	0.76	0.87	0.27	0.30	0.50	0.70	0.85
Eggerthella	-0.35	0.17	0.32	0.36	0.76	-0.46	0.05	0.14	0.39	0.79	-0.28	0.24	0.40	0.62	0.87	-0.28	0.25	0.42	0.63	0.87	-0.25	0.32	0.43	0.70	0.85
Eisenbergiella	-0.09	0.70	0.51	0.81	0.81	-0.11	0.64	0.42	0.94	0.89	-0.07	0.76	0.60	0.94	0.87	-0.07	0.75	0.61	0.93	0.87	-0.05	0.82	0.60	0.97	0.87
Erysipelatoclostridium	-0.07	0.79	0.71	0.85	0.92	-0.11	0.65	0.95	0.94	0.98	0.00	0.99	0.76	0.99	0.91	0.00	0.99	0.75	0.99	0.92	0.02	0.93	0.71	0.97	0.90
Erysipelotrichaceae_UCG.003	0.30	0.13	0.23	0.30	0.76	0.29	0.11	0.14	0.39	0.79	0.11	0.55	0.61	0.87	0.87	0.11	0.55	0.61	0.87	0.87	0.11	0.55	0.56	0.89	0.85
Escherichia.Shigella	-0.21	0.24	0.67	0.44	0.92	-0.04	0.83	0.85	0.99	0.98	-0.09	0.59	0.99	0.87	1.00	-0.09	0.60	0.99	0.88	0.99	-0.10	0.56	0.98	0.89	0.98
Faecalibacterium	0.59	0.07	0.37	0.23	0.76	0.33	0.28	0.76	0.63	0.97	0.31	0.31	0.70	0.75	0.89	0.31	0.30	0.69	0.73	0.90	0.33	0.29	0.65	0.70	0.87
Family_XIII_AD3011_group	0.58	0.02	0.26	0.12	0.76	0.36	0.12	0.36	0.40	0.89	0.49	0.05	0.14	0.37	0.79	0.49	0.05	0.14	0.38	0.79	0.50	0.05	0.12	0.41	0.74
Family_XIII_UCG.001	0.46	0.13	0.38	0.30	0.76	0.37	0.20	0.29	0.51	0.89	0.36	0.22	0.35	0.61	0.87	0.36	0.23	0.35	0.63	0.87	0.36	0.23	0.33	0.67	0.85
FD2005	-0.51	0.06	0.30	0.22	0.76	-0.25	0.33	0.91	0.65	0.98	-0.21	0.44	0.96	0.81	0.99	-0.21	0.43	0.95	0.82	0.99	-0.20	0.45	0.97	0.83	0.98
Flavonifractor	-0.22	0.37	0.75	0.59	0.92	-0.26	0.25	0.48	0.60	0.89	-0.15	0.54	0.62	0.87	0.87	-0.14	0.55	0.63	0.87	0.88	-0.09	0.72	0.74	0.93	0.91
Fournierella	0.12	0.68	0.98	0.81	1.00	0.03	0.93	0.78	0.99	0.97	0.21	0.45	0.73	0.81	0.91	0.21	0.46	0.74	0.83	0.92	0.19	0.51	0.79	0.85	0.91
Frisingicoccus	0.61	0.01	0.03	0.07	0.49	0.51	0.01	0.04	0.23	0.58	0.38	0.08	0.11	0.48	0.77	0.38	0.08	0.11	0.49	0.76	0.41	0.06	0.10	0.41	0.69
Fusicatenibacter	-0.06	0.75	0.73	0.83	0.92	-0.14	0.46	0.77	0.73	0.97	-0.27	0.17	0.47	0.61	0.87	-0.26	0.18	0.47	0.61	0.87	-0.26	0.19	0.51	0.61	0.85
Fusobacterium	-0.53	0.02	0.03	0.13	0.49	-0.38	0.08	0.04	0.39	0.58	-0.32	0.16	0.05	0.60	0.72	-0.33	0.15	0.05	0.59	0.72	-0.34	0.14	0.06	0.58	0.68
GCA.900066575	0.63	0.02	0.34	0.13	0.76	0.45	0.09	0.32	0.39	0.89	0.39	0.16	0.37	0.60	0.87	0.38	0.16	0.37	0.59	0.87	0.40	0.15	0.30	0.58	0.85
Gordonibacter	0.32	0.29	0.18	0.49	0.76	0.05	0.87	0.54	0.99	0.89	0.06	0.84	0.57	0.98	0.87	0.05	0.85	0.56	0.98	0.87	0.07	0.81	0.54	0.97	0.85
Granulicatella	-0.29	0.38	0.82	0.59	0.95	-0.16	0.60	0.82	0.90	0.97	-0.35	0.27	0.64	0.67	0.87	-0.35	0.28	0.65	0.69	0.88	-0.35	0.29	0.64	0.70	0.87
Haemophilus	0.33	0.10	0.77	0.26	0.92	0.26	0.16	0.74	0.47	0.97	0.02	0.90	0.56	0.98	0.87	0.03	0.90	0.57	0.98	0.87	0.02	0.92	0.54	0.97	0.85
Harryflintia	-0.08	0.80	0.86	0.85	0.95	-0.12	0.68	0.79	0.95	0.97	-0.14	0.63	0.94	0.87	0.99	-0.15	0.62	0.94	0.88	0.99	-0.14	0.65	0.85	0.90	0.95
Holdemanella	-0.09	0.62	0.84	0.80	0.95	-0.01	0.95	0.94	0.99	0.98	0.24	0.20	0.28	0.61	0.87	0.23	0.21	0.29	0.62	0.87	0.26	0.17	0.27	0.58	0.85
Holdemania	-0.37	0.49	0.98	0.69	1.00	-0.36	0.46	0.99	0.73	1.00	-0.36	0.48	1.00	0.86	1.00	-0.37	0.48	0.99	0.85	0.99	-0.37	0.47	0.93	0.83	0.98
Hungatella	-0.38	0.11	0.79	0.28	0.93	-0.36	0.11	0.59	0.39	0.89	-0.28	0.24	0.63	0.62	0.87	-0.27	0.24	0.65	0.63	0.88	-0.27	0.25	0.63	0.68	0.87
Hydrogenoanaerobacterium	0.71	0.02	0.21	0.12	0.76	0.40	0.14	0.38	0.45	0.89	0.38	0.18	0.36	0.61	0.87	0.38	0.19	0.36	0.62	0.87	0.40	0.17	0.38	0.58	0.85
Incertae_Sedis	0.17	0.66	0.37	0.81	0.76	0.11	0.76	0.58	0.97	0.89	0.33	0.39	0.48	0.78	0.87	0.33	0.38	0.47	0.80	0.87	0.38	0.33	0.48	0.70	0.85
Intestinibacter	0.02	0.92	0.42	0.94	0.76	0.07	0.75	0.21	0.97	0.86	0.02	0.92	0.16	0.98	0.83	0.03	0.88	0.17	0.98	0.86	0.04	0.86	0.15	0.97	0.74
Intestinimonas	0.40	0.08	0.49	0.24	0.80	0.20	0.36	0.66	0.68	0.93	0.12	0.59	0.86	0.87	0.94	0.12	0.60	0.85	0.88	0.94	0.13	0.58	0.88	0.89	0.95
Lachnoclostridium	-1.40	0.00	0.30	0.06	0.76	-1.01	0.02	0.41	0.29	0.89	-0.89	0.05	0.54	0.37	0.87	-0.89	0.05	0.54	0.38	0.87	-0.89	0.05	0.53	0.41	0.85
Lachnospira	0.46	0.03	0.40	0.14	0.76	0.28	0.17	0.61	0.47	0.90	-0.04	0.84	0.20	0.98	0.84	-0.04	0.85	0.20	0.98	0.87	-0.02	0.94	0.24	0.97	0.85
Lachnospiraceae_FCS020_group	0.93	0.00	0.07	0.01	0.59	0.70	0.00	0.06	0.10	0.70	0.66	0.01	0.11	0.19	0.77	0.66	0.01	0.11	0.19	0.76	0.71	0.01	0.08	0.12	0.68
Lachnospiraceae_ND3007_group	0.21	0.23	0.66	0.44	0.92	0.14	0.39	0.38	0.70	0.89	0.21	0.21	0.19	0.61	0.84	0.21	0.21	0.19	0.62	0.87	0.23	0.18	0.16	0.58	0.74
Lachnospiraceae_NK4A136_group	0.29	0.21	0.70	0.41	0.92	0.05	0.83	0.87	0.99	0.98	-0.19	0.41	0.89	0.81	0.97	-0.19	0.41	0.89	0.82	0.96	-0.17	0.48	0.98	0.83	0.98
Lachnospiraceae_UCG.001	0.33	0.07	0.36	0.23	0.76	0.23	0.17	0.45	0.47	0.89	0.06	0.72	0.75	0.91	0.91	0.06	0.74	0.76	0.91	0.93	0.07	0.70	0.75	0.92	0.91
Lachnospiraceae_UCG.004	0.39	0.05	0.37	0.20	0.76	0.33	0.09	0.32	0.39	0.89	0.25	0.20	0.40	0.61	0.87	0.25	0.20	0.40	0.62	0.87	0.24	0.22	0.39	0.67	0.85
Lachnospiraceae_UCG.010	0.10	0.72	0.61	0.83	0.88	-0.04	0.88	0.53	0.99	0.89	-0.03	0.90	0.40	0.98	0.87	-0.04	0.88	0.40	0.98	0.87	-0.05	0.84	0.39	0.97	0.85
Lactobacillus	-0.40	0.04	0.99	0.18	1.00	-0.25	0.17	0.68	0.47	0.93	-0.25	0.21	0.50	0.61	0.87	-0.24	0.22	0.51	0.62	0.87	-0.29	0.15	0.36	0.58	0.85
Lactococcus	0.74	0.03	0.07	0.15	0.59	0.48	0.14	0.23	0.45	0.86	0.50	0.15	0.38	0.60	0.87	0.50	0.15	0.37	0.59	0.87	0.47	0.18	0.42	0.58	0.85
Marvinbryantia	0.27	0.16	0.32	0.36	0.76	0.18	0.31	0.27	0.65	0.89	0.45	0.02	0.04	0.24	0.72	0.45	0.02	0.04	0.24	0.72	0.49	0.01	0.03	0.17	0.68
Megasphaera	-0.36	0.01	0.77	0.08	0.92	-0.22	0.08	0.84	0.39	0.98	-0.13	0.32	0.84	0.77	0.94	-0.13	0.32	0.83	0.76	0.93	-0.14	0.29	0.76	0.70	0.91

© 2022 Meyer K et al. *JAMA Network Open.*

Monoglobus	0.49	0.02	0.93	0.13	1.00	0.34	0.10	0.74	0.39	0.97	0.14	0.52	0.16	0.87	0.83	0.14	0.53	0.16	0.87	0.86	0.16	0.47	0.17	0.83	0.74
Moryella	0.26	0.45	0.69	0.65	0.92	0.07	0.81	0.66	0.99	0.93	0.17	0.61	0.99	0.87	1.00	0.17	0.61	0.98	0.88	0.99	0.17	0.62	0.93	0.90	0.98
Negativibacillus	-0.06	0.77	0.46	0.84	0.78	0.02	0.91	0.24	0.99	0.87	0.09	0.65	0.19	0.87	0.84	0.09	0.66	0.20	0.88	0.87	0.14	0.48	0.12	0.83	0.74
NK4A214_group	0.25	0.12	0.60	0.29	0.88	0.16	0.29	0.58	0.65	0.89	0.15	0.34	0.55	0.77	0.87	0.15	0.35	0.56	0.76	0.87	0.16	0.31	0.51	0.70	0.85
Odoribacter	0.37	0.01	0.03	0.11	0.49	0.23	0.09	0.14	0.39	0.79	0.26	0.07	0.06	0.47	0.73	0.26	0.07	0.06	0.48	0.74	0.27	0.07	0.05	0.41	0.68
Oscillibacter	0.44	0.09	0.33	0.26	0.76	0.41	0.10	0.19	0.39	0.86	0.53	0.04	0.08	0.37	0.74	0.52	0.05	0.08	0.38	0.74	0.59	0.03	0.05	0.29	0.68
Oscillospira	0.03	0.93	0.72	0.94	0.92	-0.04	0.90	0.44	0.99	0.89	-0.02	0.96	0.33	0.99	0.87	-0.02	0.96	0.34	0.98	0.87	0.09	0.80	0.47	0.97	0.85
Oxalobacter	0.16	0.56	0.44	0.75	0.78	-0.05	0.84	0.51	0.99	0.89	-0.06	0.82	0.58	0.98	0.87	-0.06	0.82	0.58	0.98	0.87	-0.07	0.79	0.55	0.97	0.85
Paludicola	0.57	0.05	0.45	0.20	0.78	0.43	0.11	0.29	0.39	0.89	0.26	0.36	0.66	0.77	0.88	0.26	0.37	0.66	0.79	0.88	0.28	0.33	0.67	0.70	0.88
Parabacteroides	0.18	0.38	0.31	0.59	0.76	0.17	0.36	0.68	0.68	0.93	0.17	0.36	0.82	0.77	0.93	0.18	0.35	0.80	0.76	0.93	0.19	0.33	0.75	0.70	0.91
Paraprevotella	-0.13	0.36	0.61	0.57	0.88	-0.10	0.43	0.42	0.73	0.89	-0.20	0.16	0.30	0.60	0.87	-0.20	0.16	0.30	0.59	0.87	-0.17	0.23	0.38	0.67	0.85
Parasutterella	0.60	0.00	0.01	0.00	0.49	0.54	0.00	0.02	0.01	0.58	0.43	0.00	0.08	0.11	0.74	0.43	0.00	0.08	0.11	0.74	0.43	0.00	0.09	0.11	0.68
Peptococcus	0.90	0.00	0.02	0.06	0.49	0.69	0.02	0.02	0.23	0.58	0.73	0.01	0.02	0.23	0.58	0.73	0.01	0.02	0.24	0.59	0.72	0.02	0.02	0.21	0.68
Phascolarctobacterium	0.29	0.03	0.15	0.15	0.69	0.28	0.03	0.20	0.29	0.86	0.26	0.05	0.35	0.37	0.87	0.26	0.05	0.35	0.38	0.87	0.24	0.08	0.38	0.45	0.85
Phocea	-0.75	0.02	0.10	0.13	0.63	-0.88	0.00	0.04	0.10	0.58	-0.99	0.00	0.05	0.08	0.72	-0.99	0.00	0.05	0.08	0.72	-0.96	0.00	0.07	0.11	0.68
Prevotella	-0.15	0.21	0.77	0.41	0.92	-0.11	0.34	0.92	0.66	0.98	-0.09	0.45	0.81	0.81	0.93	-0.09	0.44	0.83	0.82	0.93	-0.09	0.47	0.79	0.83	0.91
Romboutsia	-0.31	0.18	0.45	0.37	0.78	-0.24	0.28	0.46	0.63	0.89	-0.20	0.39	0.50	0.78	0.87	-0.19	0.40	0.51	0.81	0.87	-0.18	0.43	0.56	0.83	0.85
Roseburia	0.31	0.20	1.00	0.40	1.00	0.26	0.25	0.93	0.60	0.98	-0.03	0.91	0.38	0.98	0.87	-0.03	0.91	0.38	0.98	0.87	0.01	0.96	0.48	0.97	0.85
Ruminococcus	0.38	0.02	0.32	0.12	0.76	0.24	0.11	0.39	0.39	0.89	0.20	0.20	0.48	0.61	0.87	0.20	0.21	0.48	0.62	0.87	0.22	0.18	0.45	0.58	0.85
Sellimonas	0.12	0.62	0.67	0.80	0.92	0.07	0.74	0.79	0.97	0.97	0.14	0.55	0.95	0.87	0.99	0.14	0.56	0.96	0.87	0.99	0.16	0.48	0.96	0.83	0.98
Senegalimassilia	-0.27	0.20	0.20	0.40	0.76	-0.19	0.32	0.39	0.65	0.89	-0.11	0.58	0.48	0.87	0.87	-0.12	0.56	0.47	0.87	0.87	-0.11	0.60	0.49	0.90	0.85
Shuttleworthia	1.01	0.00	0.01	0.01	0.49	0.70	0.01	0.01	0.18	0.58	0.79	0.00	0.00	0.12	0.30	0.78	0.00	0.00	0.13	0.30	0.80	0.00	0.00	0.11	0.36
Slackia	-0.06	0.74	0.84	0.83	0.95	-0.06	0.73	0.57	0.97	0.89	0.01	0.96	0.81	0.99	0.93	0.01	0.97	0.81	0.98	0.93	0.02	0.93	0.91	0.97	0.97
Streptococcus	0.26	0.36	0.99	0.57	1.00	0.21	0.44	0.80	0.73	0.97	0.10	0.71	0.29	0.91	0.87	0.10	0.71	0.29	0.91	0.87	0.06	0.83	0.21	0.97	0.83
Subdoligranulum	0.43	0.05	0.94	0.20	1.00	0.35	0.09	0.88	0.39	0.98	0.44	0.04	0.58	0.37	0.87	0.44	0.04	0.59	0.38	0.87	0.49	0.02	0.44	0.29	0.85
Sutterella	-0.55	0.00	0.04	0.00	0.59	-0.45	0.00	0.04	0.01	0.58	-0.44	0.00	0.07	0.08	0.74	-0.44	0.00	0.07	0.08	0.74	-0.45	0.00	0.07	0.05	0.68
TM7x	0.01	0.98	0.72	0.98	0.92	-0.03	0.91	0.93	0.99	0.98	0.11	0.72	0.66	0.91	0.88	0.11	0.71	0.65	0.91	0.88	0.13	0.66	0.69	0.90	0.89
Turicibacter	0.14	0.67	1.00	0.81	1.00	0.31	0.31	0.88	0.65	0.98	0.54	0.10	0.54	0.58	0.87	0.55	0.10	0.53	0.57	0.87	0.56	0.10	0.47	0.50	0.85
Tuzzerella	0.40	0.09	0.02	0.25	0.49	0.39	0.07	0.04	0.39	0.58	0.38	0.11	0.05	0.58	0.72	0.37	0.11	0.05	0.59	0.72	0.39	0.10	0.03	0.50	0.68
UBA1819	0.13	0.66	0.68	0.81	0.92	0.06	0.84	0.81	0.99	0.97	0.19	0.51	0.61	0.87	0.87	0.19	0.52	0.61	0.87	0.87	0.27	0.37	0.49	0.76	0.85
UCG.002	0.36	0.02	0.37	0.12	0.76	0.24	0.09	0.46	0.39	0.89	0.23	0.12	0.41	0.59	0.87	0.23	0.12	0.42	0.59	0.87	0.25	0.10	0.33	0.50	0.85
UCG.003	0.20	0.26	0.91	0.46	0.99	0.13	0.44	0.58	0.73	0.89	0.04	0.81	0.81	0.98	0.93	0.04	0.81	0.82	0.98	0.93	0.03	0.88	0.80	0.97	0.91
UCG.005	0.50	0.01	0.12	0.08	0.63	0.32	0.06	0.25	0.39	0.88	0.27	0.14	0.32	0.60	0.87	0.27	0.14	0.33	0.59	0.87	0.29	0.13	0.29	0.58	0.85
UCG.009	0.19	0.53	0.55	0.73	0.85	-0.04	0.88	0.47	0.99	0.89	-0.13	0.66	0.35	0.87	0.87	-0.13	0.66	0.36	0.88	0.87	-0.14	0.65	0.34	0.90	0.85
Veillonella	-0.04	0.85	0.39	0.88	0.76	0.00	0.98	0.55	1.00	0.89	-0.18	0.39	0.23	0.78	0.87	-0.17	0.39	0.23	0.81	0.87	-0.21	0.31	0.19	0.70	0.82
Victivallis	-0.21	0.32	0.08	0.55	0.59	-0.28	0.17	0.12	0.47	0.79	-0.30	0.16	0.18	0.60	0.84	-0.31	0.16	0.18	0.59	0.86	-0.31	0.16	0.16	0.58	0.74

Multivariable-adjusted regression models adjusted for:

Model 1: sequencing batch, age, race, gender, study center

Model 2: Model 1, with education

Model 3: Model 2, with physical activity, diet score, medication use, and smoking

Model 4: Model 3 with BMI

Model 5: Model 4 with hypertension and diabetes

© 2022 Meyer K et al. *JAMA Network Open.*

eTable 25: Multivariable-adjusted regression coefficients, p-values, race interaction p-values, false discovery rate (FDR)-adjusted p-values for race- genera interactions with respect to PC (1st).

	Model1					Model2					Model3					Model4					Model5				
	beta	p-value	p-val int	FDR	FDR-int	beta	p-value	p-val int	FDR	FDR-int	beta	p-value	p-val int	FDR	FDR-int	beta	p-value	p-val int	FDR	FDR-int	beta	p-value	p-val int	FDR	FDR-int
Acetanaerobacterium	-0.02	0.91	0.79	0.94	0.89	-0.02	0.91	0.74	0.94	0.88	-0.05	0.81	0.85	0.94	0.96	-0.05	0.80	0.87	0.94	0.96	-0.04	0.84	1.00	0.99	1.00
Acidaminococcus	-0.09	0.11	0.17	0.30	0.62	-0.05	0.32	0.30	0.65	0.82	-0.01	0.89	0.15	1.00	0.74	-0.01	0.91	0.15	0.99	0.73	0.00	0.94	0.14	1.00	0.72
Actinomyces	-0.23	0.14	0.23	0.33	0.68	-0.16	0.26	0.27	0.65	0.82	-0.10	0.54	0.28	0.92	0.77	-0.10	0.54	0.28	0.93	0.76	-0.09	0.55	0.23	0.94	0.72
Adlercreutzia	0.12	0.26	0.33	0.45	0.71	0.06	0.54	0.62	0.80	0.86	0.05	0.64	0.58	0.93	0.85	0.05	0.65	0.57	0.93	0.84	0.05	0.66	0.55	0.94	0.87
Agathobacter	-0.10	0.45	0.36	0.62	0.71	-0.13	0.30	0.27	0.65	0.82	-0.22	0.09	0.18	0.66	0.74	-0.22	0.10	0.18	0.74	0.73	-0.20	0.13	0.20	0.81	0.72
Akkermansia	0.21	0.00	0.05	0.04	0.48	0.14	0.02	0.11	0.27	0.82	0.12	0.08	0.21	0.66	0.74	0.12	0.08	0.21	0.68	0.73	0.11	0.11	0.24	0.81	0.72
Alistipes	0.17	0.15	0.86	0.34	0.92	0.10	0.38	0.85	0.65	0.93	0.03	0.76	0.89	0.93	0.96	0.04	0.74	0.93	0.94	0.97	0.04	0.74	1.00	0.94	1.00
Allisonella	-0.22	0.05	0.44	0.19	0.72	-0.04	0.69	0.70	0.84	0.88	0.00	0.99	1.00	1.00	1.00	0.00	0.98	0.93	0.99	0.97	0.00	0.99	0.92	1.00	0.98
Anaerofilum	0.34	0.03	0.21	0.13	0.68	0.19	0.19	0.26	0.59	0.82	0.19	0.21	0.25	0.81	0.74	0.19	0.23	0.25	0.82	0.73	0.18	0.24	0.27	0.82	0.72
Anaerostipes	0.04	0.79	0.90	0.85	0.94	-0.03	0.79	0.99	0.85	0.99	-0.03	0.82	0.87	0.94	0.96	-0.03	0.85	0.82	0.97	0.95	0.00	0.97	0.77	1.00	0.94
Anaerotruncus	0.27	0.04	0.04	0.16	0.42	0.22	0.07	0.03	0.36	0.63	0.23	0.07	0.02	0.66	0.48	0.22	0.08	0.02	0.68	0.43	0.23	0.07	0.01	0.80	0.44
Bacteroides	0.15	0.43	0.15	0.62	0.62	0.09	0.62	0.15	0.84	0.82	0.08	0.66	0.17	0.93	0.74	0.10	0.60	0.16	0.93	0.73	0.09	0.61	0.15	0.94	0.72
Barnesiella	0.12	0.07	0.36	0.23	0.71	0.13	0.03	0.47	0.27	0.82	0.12	0.07	0.53	0.66	0.84	0.12	0.07	0.51	0.68	0.84	0.12	0.07	0.48	0.80	0.87
Bifidobacterium	0.05	0.57	0.24	0.72	0.69	0.10	0.24	0.33	0.65	0.82	0.07	0.39	0.43	0.88	0.84	0.08	0.35	0.39	0.89	0.82	0.08	0.35	0.39	0.83	0.80
Bilophila	0.16	0.07	0.67	0.23	0.86	0.08	0.33	0.75	0.65	0.88	0.04	0.68	0.52	0.93	0.84	0.04	0.63	0.56	0.93	0.84	0.03	0.73	0.52	0.94	0.87
Blautia	0.18	0.53	0.45	0.68	0.72	0.10	0.71	0.34	0.84	0.82	0.17	0.52	0.32	0.92	0.78	0.19	0.49	0.28	0.93	0.76	0.29	0.32	0.21	0.82	0.72
Butyricicoccus	0.38	0.05	0.19	0.19	0.67	0.16	0.37	0.31	0.65	0.82	0.09	0.63	0.25	0.92	0.74	0.10	0.59	0.24	0.93	0.73	0.10	0.60	0.26	0.94	0.72
Butyricimonas	0.14	0.06	0.44	0.21	0.72	0.11	0.12	0.41	0.45	0.82	0.13	0.07	0.19	0.66	0.74	0.13	0.07	0.20	0.68	0.73	0.13	0.06	0.16	0.80	0.72
CAG.56	-0.06	0.51	0.03	0.66	0.42	-0.07	0.40	0.07	0.67	0.72	-0.10	0.23	0.07	0.81	0.72	-0.10	0.24	0.08	0.82	0.73	-0.10	0.26	0.08	0.82	0.72
Candidatus_Soleaferrea	0.31	0.08	0.02	0.25	0.42	0.27	0.09	0.01	0.43	0.58	0.27	0.11	0.01	0.77	0.45	0.27	0.11	0.01	0.78	0.43	0.29	0.09	0.01	0.81	0.44
Caproiciproducens	0.12	0.42	0.33	0.62	0.71	0.08	0.55	0.51	0.81	0.84	0.00	0.99	0.72	1.00	0.90	0.00	0.99	0.72	0.99	0.91	-0.01	0.95	0.74	1.00	0.94
Christensenellaceae_R.7_group	0.22	0.00	0.28	0.04	0.69	0.14	0.04	0.57	0.28	0.85	0.09	0.24	0.85	0.81	0.96	0.08	0.27	0.88	0.82	0.96	0.09	0.26	0.87	0.82	0.94
Clostridium_sensu_stricto_1	-0.07	0.49	0.14	0.65	0.62	-0.03	0.73	0.19	0.84	0.82	-0.07	0.46	0.10	0.91	0.74	-0.07	0.48	0.10	0.93	0.73	-0.07	0.46	0.11	0.89	0.72
Colidextribacter	-0.05	0.74	0.69	0.84	0.86	-0.07	0.64	0.37	0.84	0.82	-0.01	0.95	0.14	1.00	0.74	-0.01	0.97	0.12	0.99	0.73	0.03	0.86	0.10	0.99	0.72
Collinsella	-0.12	0.11	0.35	0.30	0.71	-0.03	0.69	0.46	0.84	0.82	-0.04	0.57	0.37	0.92	0.83	-0.04	0.56	0.34	0.93	0.78	-0.05	0.48	0.30	0.89	0.72
Coprobacter	0.12	0.24	0.50	0.43	0.76	0.08	0.36	0.42	0.65	0.82	0.04	0.67	0.41	0.93	0.84	0.04	0.70	0.43	0.93	0.83	0.03	0.76	0.48	0.94	0.87
Coprococcus	-0.01	0.94	0.11	0.96	0.62	0.03	0.75	0.30	0.84	0.82	0.06	0.55	0.70	0.92	0.90	0.06	0.55	0.70	0.93	0.91	0.07	0.45	0.79	0.89	0.94
Defluviitaleaceae_UCG.011	0.41	0.00	0.17	0.04	0.62	0.35	0.01	0.17	0.21	0.82	0.32	0.02	0.25	0.65	0.74	0.31	0.03	0.25	0.68	0.73	0.32	0.03	0.23	0.59	0.72
Desulfovibrio	0.02	0.82	0.32	0.87	0.71	0.03	0.68	0.26	0.84	0.82	0.04	0.62	0.18	0.92	0.74	0.03	0.71	0.20	0.93	0.73	0.03	0.68	0.20	0.94	0.72
Dialister	-0.10	0.12	0.10	0.30	0.62	-0.08	0.16	0.14	0.53	0.82	-0.07	0.25	0.23	0.81	0.74	-0.07	0.26	0.22	0.82	0.73	-0.07	0.30	0.24	0.82	0.72
DNF00809	0.03	0.87	0.60	0.90	0.83	0.01	0.92	0.71	0.95	0.88	0.06	0.69	0.96	0.93	0.98	0.06	0.70	0.97	0.93	0.99	0.06	0.72	0.96	0.94	0.99
Dorea	0.03	0.79	0.61	0.85	0.83	0.03	0.73	0.78	0.84	0.89	0.06	0.56	0.55	0.92	0.84	0.06	0.54	0.55	0.93	0.84	0.05	0.61	0.60	0.94	0.88
DTU089	0.18	0.14	0.10	0.33	0.62	0.11	0.35	0.23	0.65	0.82	0.08	0.53	0.39	0.92	0.84	0.08	0.53	0.36	0.93	0.78	0.10	0.42	0.31	0.87	0.72
Eggerthella	-0.05	0.65	0.89	0.78	0.94	-0.11	0.30	0.54	0.65	0.85	-0.02	0.83	0.95	0.94	0.98	-0.02	0.86	0.99	0.97	0.99	-0.02	0.90	0.96	1.00	0.99
Eisenbergiella	-0.03	0.80	0.79	0.85	0.89	-0.03	0.74	0.86	0.84	0.93	0.00	0.97	0.62	1.00	0.86	-0.01	0.93	0.57	0.99	0.84	0.00	1.00	0.61	1.00	0.88
Erysipelatoclostridium	0.05	0.66	0.14	0.78	0.62	0.03	0.77	0.21	0.85	0.82	0.08	0.51	0.19	0.92	0.74	0.07	0.53	0.17	0.93	0.73	0.08	0.48	0.15	0.89	0.72
Erysipelotrichaceae_UCG.003	0.09	0.33	0.81	0.52	0.89	0.09	0.31	0.65	0.65	0.88	0.00	0.96	0.79	1.00	0.96	0.00	0.98	0.78	0.99	0.95	0.00	0.96	0.83	1.00	0.94
Escherichia.Shigella	-0.07	0.39	0.96	0.61	0.97	0.02	0.82	0.51	0.87	0.84	0.03	0.73	0.62	0.93	0.86	0.03	0.70	0.60	0.93	0.85	0.03	0.73	0.60	0.94	0.88
Faecalibacterium	0.24	0.11	0.77	0.30	0.88	0.11	0.45	0.70	0.72	0.88	0.04	0.78	0.51	0.93	0.84	0.05	0.75	0.54	0.94	0.84	0.07	0.64	0.64	0.94	0.90
Family_XIII_AD3011_group	0.30	0.01	0.30	0.07	0.70	0.19	0.08	0.41	0.40	0.82	0.16	0.17	0.29	0.81	0.77	0.15	0.20	0.30	0.82	0.77	0.15	0.21	0.31	0.82	0.72
Family_XIII_UCG.001	0.24	0.10	0.57	0.28	0.81	0.19	0.15	0.47	0.53	0.82	0.13	0.37	0.66	0.88	0.87	0.12	0.41	0.67	0.92	0.89	0.12	0.41	0.68	0.87	0.93
FD2005	-0.20	0.12	0.13	0.30	0.62	-0.07	0.56	0.57	0.81	0.85	-0.09	0.50	0.46	0.92	0.84	-0.09	0.49	0.43	0.93	0.83	-0.08	0.55	0.52	0.94	0.87
Flavonifractor	-0.05	0.65	0.36	0.78	0.71	-0.07	0.48	0.55	0.75	0.85	-0.01	0.90	0.46	1.00	0.84	-0.01	0.94	0.43	0.99	0.83	0.01	0.96	0.39	1.00	0.80
Fournierella	0.11	0.44	0.53	0.62	0.78	0.06	0.66	0.73	0.84	0.88	0.04	0.76	0.65	0.93	0.87	0.03	0.81	0.67	0.95	0.89	0.05	0.69	0.60	0.94	0.88
Frisingicoccus	0.19	0.07	0.04	0.23	0.43	0.14	0.15	0.05	0.53	0.65	0.11	0.30	0.07	0.82	0.72	0.10	0.31	0.07	0.85	0.73	0.10	0.32	0.08	0.83	0.72
Fusicatenibacter	0.00	0.98	0.36	0.98	0.71	-0.04	0.65	0.36	0.84	0.82	-0.10	0.29	0.25	0.82	0.74	-0.09	0.32	0.25	0.85	0.73	-0.11	0.25	0.21	0.82	0.72
Fusobacterium	-0.16	0.16	0.31	0.34	0.71	-0.08	0.42	0.44	0.68	0.82	0.00	1.00	0.53	1.00	0.84	-0.01	0.96	0.53	0.99	0.84	0.00	0.97	0.68	1.00	0.93
GCA.900066575	0.28	0.03	0.28	0.15	0.69	0.19	0.12	0.25	0.45	0.82	0.11	0.40	0.41	0.90	0.84	0.10	0.44	0.41	0.93	0.83	0.12	0.38	0.30	0.86	0.72
Gordonibacter	0.30	0.03	0.07	0.15	0.54	0.16	0.21	0.28	0.64	0.82	0.16	0.24	0.23	0.81	0.74	0.16	0.26	0.22	0.82	0.73	0.15	0.29	0.25	0.82	0.72
Granulicatella	-0.12	0.45	0.45	0.62	0.72	-0.05	0.70	0.76	0.84	0.88	-0.06	0.67	0.54	0.93	0.84	-0.05	0.72	0.58	0.93	0.84	-0.04	0.79	0.58	0.95	0.88
Haemophilus	0.11	0.23	0.63	0.42	0.83	0.08	0.37	0.62	0.65	0.86	0.00	0.99	0.31	1.00	0.78	0.00	0.97	0.33	0.99	0.78	0.02	0.87	0.37	0.99	0.80
Harryflintia	0.06	0.65	0.45	0.78	0.72	0.04	0.74	0.38	0.84	0.82	0.00	0.99	0.62	1.00	0.86	-0.01	0.95	0.63	0.99	0.87	-0.01	0.97	0.55	1.00	0.87
Holdemanella	-0.05	0.60	0.71	0.74	0.88	-0.01	0.94	0.92	0.95	0.97	0.07	0.44	0.51	0.90	0.84	0.07	0.45	0.55	0.93	0.84	0.07	0.44	0.55	0.89	0.87
Holdemania	0.14	0.56	0.76	0.72	0.88	0.15	0.52	0.74	0.79	0.88	0.12	0.61	0.80	0.92	0.96	0.12	0.63	0.79	0.93	0.95	0.10	0.68	0.75	0.94	0.94
Hungatella	-0.16	0.16	0.47	0.34	0.72	-0.15	0.16	0.61	0.53	0.86	-0.09	0.44	0.57	0.90	0.84	-0.08	0.47	0.52	0.93	0.84	-0.07	0.53	0.53	0.94	0.87
Hydrogenoanaerobacterium	0.37	0.01	0.40	0.06	0.72	0.21	0.10	0.69	0.43	0.88	0.17	0.21	0.70	0.81	0.90	0.16	0.24	0.71	0.82	0.91	0.16	0.26	0.76	0.82	0.94
Incertae_Sedis	0.18	0.32	0.62	0.52	0.83	0.15	0.37	0.95	0.65	0.97	0.16	0.37	0.94	0.88	0.98	0.17	0.35	0.90	0.89	0.96	0.17	0.36	0.94	0.83	0.99
Intestinibacter	0.03	0.75	0.43	0.84	0.72	0.06	0.57	0.20	0.81	0.82	0.03	0.78	0.18	0.93	0.74	0.04	0.69	0.20	0.93	0.73	0.06	0.61	0.22	0.94	0.72
Intestinimonas	0.32	0.00	0.11	0.04	0.62	0.22	0.03	0.17	0.27	0.82	0.17	0.12	0.34	0.77	0.78	0.16	0.13	0.32	0.82	0.78	0.17	0.12	0.36	0.81	0.80
Lachnoclostridium	-0.59	0.01	0.68	0.06	0.86	-0.39	0.06	0.90	0.35	0.95	-0.20	0.36	0.88	0.88	0.96	-0.19	0.39	0.87	0.92	0.96	-0.19	0.40	0.86	0.87	0.94
Lachnospira	0.19	0.05	0.03	0.19	0.42	0.10	0.29	0.05	0.65	0.65	-0.03	0.74	0.01	0.93	0.45	-0.03	0.79	0.01	0.94	0.43	-0.01	0.92	0.02	1.00	0.55
Lachnospiraceae_FCS020_group	0.43	0.00	0.37	0.03	0.71	0.31	0.01	0.39	0.21	0.82	0.27	0.02	0.51	0.65	0.84	0.27	0.02	0.50	0.68	0.84	0.27	0.03	0.51	0.59	0.87
Lachnospiraceae_ND3007_group	0.09	0.28	0.73	0.47	0.88	0.05	0.47	0.95	0.73	0.97	0.03	0.69	0.92	0.93	0.96	0.04	0.65	0.90	0.93	0.96	0.04	0.62	0.86	0.94	0.94
Lachnospiraceae_NK4A136_group	0.12	0.28	0.98	0.47	0.98	-0.01	0.95	0.81	0.95	0.90	-0.13	0.22	0.72	0.81	0.90	-0.13	0.22	0.74	0.82	0.92	-0.12	0.27	0.81	0.82	0.94
Lachnospiraceae_UCG.001	0.13	0.12	0.86	0.30	0.92	0.09	0.27	0.96	0.65	0.97	0.00	0.97	0.56	1.00	0.84	0.00	0.97	0.53	0.99	0.84	0.00	1.00	0.54	1.00	0.87
Lachnospiraceae_UCG.004	0.11	0.24	0.80	0.43	0.89	0.08	0.38	0.75	0.65	0.88	0.04	0.70	0.97	0.93	0.98	0.04	0.68	0.98	0.93	0.99	0.03	0.76	0.93	0.94	0.99
Lachnospiraceae_UCG.010	-0.05	0.69	0.96	0.79	0.97	-0.12	0.31	0.96	0.65	0.97	-0.14	0.26	0.83	0.81	0.96	-0.15	0.24	0.85	0.82	0.96	-0.15	0.24	0.80	0.82	0.94
Lactobacillus	-0.15	0.10	0.26	0.28	0.69	-0.08	0.35	0.44	0.65	0.82	-0.09	0.33	0.57	0.85	0.84	-0.09	0.36	0.56	0.89	0.84	-0.10	0.30	0.72	0.82	0.94
Lactococcus	0.40	0.01	0.03	0.08	0.42	0.27	0.08	0.11	0.39	0.82	0.24	0.14	0.25	0.81	0.74	0.24	0.14	0.23	0.82	0.73	0.21	0.20	0.29	0.82	0.72
Marvinbryantia	0.06	0.47	0.64	0.63	0.83	0.02	0.79	0.57	0.85	0.85	0.02	0.78	0.61	0.93	0.86	0.03	0.78	0.59	0.94	0.85	0.04	0.64	0.51	0.94	0.87
Megasphaera	-0.10	0.13	0.91	0.32	0.95	-0.02	0.69	0.82	0.84	0.90	0.03	0.61	0.84	0.92	0.96	0.03	0.63	0.89	0.93	0.96	0.02	0.76	1.00	0.94	1.00

© 2022 Meyer K et al. *JAMA Network Open.*

Monoglobus	0.26	0.01	0.75	0.07	0.88	0.18	0.06	0.42	0.35	0.82	0.05	0.60	0.07	0.92	0.72	0.05	0.60	0.07	0.93	0.73	0.05	0.61	0.06	0.94	0.72
Moryella	0.15	0.33	0.54	0.52	0.78	0.06	0.67	0.50	0.84	0.84	-0.04	0.79	0.40	0.93	0.84	-0.04	0.78	0.39	0.94	0.82	-0.04	0.79	0.38	0.95	0.80
Negativibacillus	0.04	0.67	0.13	0.78	0.62	0.08	0.35	0.04	0.65	0.65	0.10	0.29	0.03	0.82	0.63	0.09	0.31	0.04	0.85	0.63	0.09	0.31	0.03	0.82	0.59
NK4A214_group	0.13	0.10	0.63	0.28	0.83	0.08	0.25	0.61	0.65	0.86	0.03	0.71	0.77	0.93	0.95	0.02	0.78	0.82	0.94	0.95	0.03	0.74	0.83	0.94	0.94
Odoribacter	0.20	0.00	0.07	0.04	0.54	0.13	0.04	0.29	0.28	0.82	0.11	0.12	0.21	0.77	0.74	0.11	0.13	0.22	0.82	0.73	0.11	0.12	0.18	0.81	0.72
Oscillibacter	0.24	0.05	0.60	0.19	0.83	0.22	0.05	0.38	0.35	0.82	0.23	0.06	0.25	0.66	0.74	0.23	0.07	0.25	0.68	0.73	0.23	0.08	0.26	0.80	0.72
Oscillospira	0.16	0.33	0.46	0.52	0.72	0.12	0.41	0.73	0.67	0.88	0.12	0.42	0.87	0.90	0.96	0.13	0.42	0.83	0.92	0.96	0.17	0.30	0.71	0.82	0.94
Oxalobacter	0.17	0.18	0.51	0.38	0.76	0.06	0.59	0.60	0.82	0.86	0.00	0.97	0.53	1.00	0.84	-0.01	0.95	0.51	0.99	0.84	-0.02	0.85	0.42	0.99	0.84
Paludicola	0.34	0.01	0.17	0.07	0.62	0.27	0.03	0.08	0.27	0.77	0.18	0.18	0.15	0.81	0.74	0.17	0.21	0.16	0.82	0.73	0.17	0.23	0.21	0.82	0.72
Parabacteroides	0.11	0.22	0.22	0.42	0.68	0.11	0.19	0.56	0.59	0.85	0.09	0.30	0.86	0.82	0.96	0.10	0.28	0.82	0.82	0.95	0.11	0.24	0.75	0.82	0.94
Paraprevotella	-0.08	0.23	0.41	0.42	0.72	-0.07	0.27	0.25	0.65	0.82	-0.08	0.23	0.30	0.81	0.77	-0.08	0.21	0.28	0.82	0.76	-0.08	0.23	0.30	0.82	0.72
Parasutterella	0.21	0.00	0.26	0.04	0.69	0.18	0.00	0.34	0.21	0.82	0.13	0.07	0.49	0.66	0.84	0.13	0.07	0.49	0.68	0.84	0.12	0.08	0.47	0.80	0.87
Peptococcus	0.38	0.01	0.02	0.06	0.42	0.27	0.04	0.02	0.28	0.63	0.20	0.15	0.04	0.81	0.63	0.20	0.16	0.04	0.82	0.63	0.19	0.18	0.05	0.82	0.70
Phascolarctobacterium	0.16	0.01	0.07	0.07	0.54	0.16	0.01	0.09	0.21	0.82	0.16	0.01	0.12	0.65	0.74	0.16	0.01	0.12	0.68	0.73	0.16	0.01	0.11	0.59	0.72
Phocea	-0.20	0.20	0.65	0.39	0.84	-0.26	0.06	0.45	0.36	0.82	-0.27	0.07	0.56	0.66	0.84	-0.26	0.08	0.57	0.68	0.84	-0.24	0.11	0.69	0.81	0.93
Prevotella	-0.08	0.18	0.77	0.38	0.88	-0.05	0.29	0.89	0.65	0.95	-0.04	0.42	0.90	0.90	0.96	-0.05	0.39	0.96	0.92	0.99	-0.04	0.47	0.87	0.89	0.94
Romboutsia	-0.09	0.41	0.16	0.62	0.62	-0.05	0.62	0.15	0.84	0.82	-0.06	0.58	0.21	0.92	0.74	-0.05	0.64	0.24	0.93	0.73	-0.04	0.69	0.28	0.94	0.72
Roseburia	0.09	0.43	0.74	0.62	0.88	0.07	0.54	0.79	0.80	0.89	-0.06	0.61	0.33	0.92	0.78	-0.06	0.62	0.33	0.93	0.78	-0.03	0.81	0.44	0.96	0.85
Ruminococcus	0.18	0.02	0.42	0.09	0.72	0.11	0.11	0.52	0.45	0.84	0.06	0.43	0.75	0.90	0.93	0.06	0.45	0.76	0.93	0.93	0.06	0.47	0.82	0.89	0.94
Sellimonas	0.05	0.67	0.15	0.78	0.62	0.03	0.80	0.18	0.86	0.82	0.00	0.99	0.16	1.00	0.74	-0.01	0.95	0.14	0.99	0.73	-0.01	0.93	0.13	1.00	0.72
Senegalimassilia	-0.06	0.51	0.26	0.66	0.69	-0.03	0.77	0.50	0.85	0.84	-0.05	0.62	0.44	0.92	0.84	-0.05	0.58	0.40	0.93	0.82	-0.05	0.63	0.42	0.94	0.84
Shuttleworthia	0.45	0.00	0.01	0.03	0.42	0.30	0.02	0.02	0.27	0.63	0.31	0.02	0.01	0.65	0.45	0.30	0.02	0.01	0.68	0.43	0.29	0.03	0.01	0.59	0.44
Slackia	-0.03	0.74	0.54	0.84	0.78	-0.03	0.72	0.79	0.84	0.89	-0.02	0.79	0.65	0.93	0.87	-0.02	0.78	0.66	0.94	0.89	-0.03	0.74	0.61	0.94	0.88
Streptococcus	0.18	0.19	0.33	0.38	0.71	0.15	0.23	0.43	0.65	0.82	0.14	0.28	0.82	0.82	0.96	0.15	0.26	0.72	0.82	0.91	0.14	0.29	0.85	0.82	0.94
Subdoligranulum	0.29	0.01	0.46	0.05	0.72	0.25	0.01	0.38	0.22	0.82	0.21	0.04	0.33	0.66	0.78	0.21	0.04	0.34	0.68	0.78	0.23	0.03	0.26	0.59	0.72
Sutterella	-0.18	0.00	0.17	0.04	0.62	-0.13	0.02	0.17	0.27	0.82	-0.13	0.04	0.19	0.66	0.74	-0.13	0.04	0.18	0.68	0.73	-0.12	0.05	0.21	0.80	0.72
TM7x	-0.11	0.43	0.39	0.62	0.72	-0.13	0.31	0.68	0.65	0.88	-0.07	0.61	0.92	0.92	0.96	-0.07	0.63	0.96	0.93	0.99	-0.07	0.63	0.86	0.94	0.94
Turicibacter	0.04	0.79	0.27	0.85	0.69	0.13	0.37	0.31	0.65	0.82	0.19	0.22	0.52	0.81	0.84	0.20	0.20	0.56	0.82	0.84	0.20	0.21	0.62	0.82	0.89
Tuzzerella	0.14	0.19	0.01	0.38	0.42	0.14	0.17	0.02	0.54	0.63	0.11	0.31	0.05	0.82	0.68	0.11	0.31	0.05	0.85	0.69	0.11	0.34	0.05	0.83	0.70
UBA1819	0.16	0.25	0.20	0.44	0.68	0.12	0.34	0.25	0.65	0.82	0.18	0.18	0.14	0.81	0.74	0.17	0.21	0.13	0.82	0.73	0.19	0.18	0.12	0.82	0.72
UCG.002	0.17	0.02	0.23	0.09	0.68	0.11	0.11	0.28	0.45	0.82	0.06	0.38	0.29	0.88	0.77	0.06	0.42	0.30	0.92	0.77	0.07	0.35	0.24	0.83	0.72
UCG.003	0.12	0.14	0.95	0.33	0.97	0.09	0.27	0.59	0.65	0.86	0.04	0.63	0.65	0.92	0.87	0.04	0.64	0.67	0.93	0.89	0.03	0.72	0.69	0.94	0.93
UCG.005	0.27	0.00	0.10	0.04	0.62	0.18	0.02	0.23	0.27	0.82	0.12	0.18	0.34	0.81	0.78	0.11	0.21	0.35	0.82	0.78	0.12	0.20	0.35	0.82	0.79
UCG.009	0.11	0.45	0.85	0.62	0.92	-0.01	0.93	0.76	0.95	0.88	-0.12	0.38	0.48	0.88	0.84	-0.12	0.40	0.52	0.92	0.84	-0.12	0.41	0.50	0.87	0.87
Veillonella	-0.01	0.96	0.28	0.96	0.69	0.01	0.89	0.42	0.93	0.82	-0.05	0.62	0.19	0.92	0.74	-0.04	0.67	0.22	0.93	0.73	-0.04	0.69	0.21	0.94	0.72

Multivariable-adjusted regression models adjusted for:

Model 1: sequencing batch, age, race, gender, study center

Model 2: Model 1, with education

Model 3: Model 2, with physical activity, diet score, medication use, and smoking

Model 4: Model 3 with BMI

Model 5: Model 4 with hypertension and diabetes

© 2022 Meyer K et al. *JAMA Network Open.*

eTable 26: Multivariable-adjusted regression coefficients, p-values, and false discovery rate (FDR)-adjusted p-values for genera among Black participants with respect to DSST.

	Model1			Model2			Model3			Model4			Model5		
	beta	p-value	FDR	beta	p-value	FDR	beta	p-value	FDR	beta	p-value	FDR	beta	p-value	FDR
Acetanaerobacterium	0.13	0.95	0.96	0.16	0.93	0.97	-0.13	0.95	0.98	-0.08	0.97	0.98	-0.31	0.87	0.93
Acidaminococcus	-0.19	0.74	0.85	0.17	0.76	0.88	0.61	0.31	0.71	0.57	0.33	0.68	0.59	0.33	0.65
Actinomyces	-2.86	0.06	0.18	-2.24	0.12	0.39	-1.73	0.26	0.71	-1.70	0.27	0.68	-1.46	0.36	0.65
Adlercreutzia	2.29	0.03	0.12	1.83	0.06	0.30	2.07	0.04	0.33	2.10	0.04	0.29	2.08	0.05	0.31
Agathobacter	0.29	0.82	0.88	0.05	0.97	0.97	-0.49	0.70	0.89	-0.59	0.64	0.90	-0.44	0.74	0.89
Akkermansia	2.08	0.00	0.03	1.51	0.02	0.16	1.58	0.02	0.23	1.59	0.02	0.21	1.72	0.01	0.16
Alistipes	1.67	0.15	0.35	1.08	0.33	0.61	0.66	0.55	0.84	0.65	0.56	0.84	0.66	0.55	0.77
Allisonella	-2.44	0.03	0.12	-0.76	0.49	0.74	-0.25	0.83	0.96	-0.26	0.83	0.95	-0.25	0.83	0.93
Anaerofilum	3.78	0.01	0.07	2.42	0.10	0.38	2.62	0.08	0.53	2.75	0.07	0.45	2.95	0.05	0.31
Anaerostipes	1.40	0.33	0.60	0.75	0.58	0.78	1.37	0.33	0.71	1.25	0.38	0.68	1.35	0.35	0.65
Anaerotruncus	3.88	0.00	0.03	3.38	0.01	0.10	2.90	0.02	0.23	3.08	0.02	0.20	3.27	0.01	0.15
Atopobium	1.94	0.39	0.63	2.76	0.19	0.52	2.15	0.34	0.71	1.98	0.38	0.68	2.67	0.25	0.64
Bacteroides	-1.49	0.44	0.67	-1.99	0.27	0.60	-1.72	0.34	0.71	-1.92	0.29	0.68	-2.05	0.26	0.64
Barnesiella	1.90	0.00	0.04	2.00	0.00	0.05	1.75	0.01	0.18	1.77	0.01	0.14	1.85	0.01	0.11
Bifidobacterium	0.31	0.73	0.85	0.69	0.41	0.66	0.61	0.47	0.78	0.53	0.54	0.84	0.63	0.47	0.74
Bilophila	0.24	0.78	0.85	-0.49	0.55	0.76	-1.13	0.18	0.59	-1.20	0.15	0.61	-1.16	0.17	0.61
Blautia	5.44	0.05	0.16	4.87	0.06	0.30	6.06	0.03	0.24	5.93	0.03	0.27	6.79	0.02	0.20
Butyricicoccus	4.75	0.01	0.07	2.82	0.12	0.39	2.49	0.18	0.59	2.41	0.19	0.61	2.75	0.14	0.58
Butyricimonas	0.50	0.51	0.73	0.16	0.82	0.91	0.29	0.70	0.89	0.30	0.68	0.92	0.32	0.67	0.86
CAG.56	-0.76	0.38	0.61	-0.84	0.30	0.60	-1.66	0.05	0.33	-1.73	0.04	0.29	-1.58	0.06	0.33
Candidatus_Soleaferrea	5.83	0.00	0.02	5.43	0.00	0.05	4.83	0.00	0.16	4.84	0.00	0.12	4.89	0.00	0.11
Caproiciproducens	1.60	0.29	0.56	1.18	0.40	0.66	0.80	0.59	0.88	0.84	0.57	0.84	1.09	0.47	0.74
Catenibacterium	0.29	0.72	0.85	0.52	0.49	0.74	0.21	0.79	0.94	0.17	0.83	0.95	0.21	0.80	0.93
Christensenellaceae_R.7_group	2.40	0.00	0.02	1.67	0.02	0.16	1.14	0.12	0.59	1.23	0.09	0.51	1.51	0.04	0.31
Clostridium_sensu_stricto_1	0.50	0.61	0.79	0.83	0.37	0.66	0.03	0.97	0.98	-0.02	0.98	0.98	0.07	0.94	0.97
Colidextribacter	-0.73	0.66	0.82	-1.02	0.51	0.74	0.21	0.89	0.96	0.22	0.89	0.97	0.57	0.73	0.89
Collinsella	-1.80	0.02	0.09	-1.00	0.18	0.51	-0.74	0.33	0.71	-0.74	0.33	0.68	-0.67	0.39	0.68
Coprobacter	0.57	0.56	0.76	0.20	0.83	0.91	-0.43	0.66	0.88	-0.35	0.71	0.93	-0.41	0.67	0.86
Coprococcus	0.60	0.53	0.74	0.90	0.32	0.61	0.95	0.30	0.71	0.92	0.32	0.68	0.98	0.29	0.64
Defluviitaleaceae_UCG.011	4.34	0.00	0.03	3.76	0.00	0.10	3.11	0.03	0.24	3.26	0.02	0.21	3.33	0.02	0.19
Desulfovibrio	0.79	0.30	0.56	0.82	0.25	0.60	1.00	0.18	0.59	1.11	0.14	0.58	1.08	0.16	0.59
Dialister	-0.06	0.92	0.96	0.12	0.83	0.91	0.70	0.25	0.70	0.69	0.26	0.68	0.77	0.22	0.64
DNF00809	1.48	0.36	0.60	1.33	0.38	0.66	1.34	0.39	0.71	1.39	0.37	0.68	1.42	0.37	0.65
Dorea	-0.02	0.99	0.99	0.01	0.99	0.99	0.47	0.64	0.88	0.44	0.67	0.91	0.48	0.64	0.85
DTU089	3.44	0.01	0.05	2.74	0.02	0.16	2.88	0.02	0.23	2.91	0.02	0.20	3.00	0.02	0.16
Eggerthella	0.75	0.53	0.74	0.21	0.85	0.92	0.84	0.48	0.78	0.83	0.48	0.80	0.84	0.48	0.74
Eisenbergiella	0.73	0.55	0.75	0.55	0.63	0.81	1.29	0.27	0.71	1.40	0.23	0.67	1.33	0.26	0.64
Enterorhabdus	1.91	0.14	0.32	1.59	0.19	0.52	1.16	0.37	0.71	1.18	0.37	0.68	1.45	0.28	0.64
Erysipelatoclostridium	1.25	0.35	0.60	0.98	0.43	0.68	1.46	0.25	0.70	1.51	0.23	0.67	1.39	0.28	0.64
Erysipelotrichaceae_UCG.003	0.93	0.31	0.56	0.96	0.26	0.60	0.42	0.63	0.88	0.45	0.61	0.88	0.65	0.47	0.74
Escherichia.Shigella	0.37	0.67	0.82	1.18	0.15	0.46	1.20	0.16	0.59	1.15	0.18	0.61	1.03	0.25	0.64
Faecalibacterium	2.50	0.10	0.25	1.33	0.35	0.64	0.60	0.68	0.89	0.50	0.73	0.93	0.81	0.58	0.79
Family_XIII_AD3011_group	3.50	0.00	0.03	2.46	0.03	0.17	2.13	0.07	0.49	2.28	0.06	0.38	2.46	0.04	0.31
Family_XIII_UCG.001	2.96	0.04	0.14	2.64	0.05	0.29	1.29	0.36	0.71	1.42	0.32	0.68	1.41	0.33	0.65
FD2005	-0.35	0.78	0.85	0.83	0.50	0.74	0.79	0.54	0.84	0.79	0.54	0.84	0.88	0.50	0.74
Flavonifractor	-0.32	0.79	0.85	-0.53	0.64	0.81	0.36	0.77	0.94	0.34	0.77	0.95	0.43	0.73	0.89
Frisingicoccus	0.52	0.61	0.79	0.08	0.93	0.97	0.14	0.89	0.96	0.22	0.83	0.95	0.33	0.75	0.89
Fusicatenibacter	0.48	0.60	0.79	0.20	0.82	0.91	0.15	0.87	0.96	0.07	0.94	0.98	0.24	0.80	0.93
Fusobacterium	-2.74	0.03	0.12	-1.87	0.12	0.39	-1.08	0.39	0.71	-1.01	0.42	0.74	-1.32	0.30	0.64
GCA.900066575	2.06	0.12	0.30	1.09	0.39	0.66	-0.17	0.89	0.96	-0.07	0.96	0.98	0.24	0.86	0.93
Gordonibacter	2.58	0.07	0.19	1.43	0.29	0.60	1.25	0.36	0.71	1.44	0.30	0.68	1.42	0.31	0.64
Granulicatella	-2.63	0.09	0.22	-1.94	0.18	0.51	-2.33	0.12	0.59	-2.44	0.11	0.53	-2.14	0.16	0.59
Haemophilus	1.21	0.19	0.39	0.92	0.29	0.60	0.45	0.62	0.88	0.43	0.64	0.90	0.73	0.44	0.73
Harryflintia	0.96	0.50	0.73	0.75	0.57	0.78	0.35	0.80	0.95	0.47	0.74	0.94	0.64	0.66	0.86

© 2022 Meyer K et al. *JAMA Network Open.*

Holdemanella	-0.07	0.94	0.96	0.28	0.74	0.88	0.94	0.30	0.71	0.93	0.30	0.68	0.73	0.43	0.73
Holdemania	2.02	0.42	0.64	1.96	0.40	0.66	1.61	0.51	0.82	1.75	0.47	0.79	1.67	0.50	0.74
Howardella	0.34	0.78	0.85	0.14	0.91	0.97	-0.15	0.90	0.96	-0.04	0.98	0.98	0.05	0.97	0.97
Hungatella	-0.46	0.70	0.84	-0.45	0.69	0.85	0.21	0.86	0.96	0.19	0.87	0.97	0.22	0.85	0.93
Hydrogenoanaerobacterium	2.70	0.05	0.15	1.23	0.35	0.64	1.18	0.38	0.71	1.39	0.31	0.68	1.61	0.25	0.64
Incertae_Sedis	5.00	0.01	0.05	4.61	0.01	0.11	4.71	0.01	0.18	4.68	0.01	0.14	4.73	0.01	0.14
Intestinibacter	1.18	0.34	0.60	1.26	0.28	0.60	1.55	0.20	0.63	1.40	0.25	0.68	1.49	0.23	0.64
Intestinimonas	3.32	0.00	0.03	2.33	0.02	0.16	1.55	0.16	0.59	1.64	0.14	0.58	1.64	0.14	0.58
Lachnoclostridium	-5.76	0.01	0.07	-4.00	0.06	0.30	-2.74	0.21	0.65	-2.89	0.19	0.61	-3.17	0.15	0.59
Lachnospira	2.10	0.04	0.13	1.20	0.21	0.54	-0.08	0.94	0.98	-0.20	0.84	0.95	-0.04	0.97	0.97
Lachnospiraceae_FCS020_group	4.84	0.00	0.01	3.82	0.00	0.05	3.93	0.00	0.10	3.90	0.00	0.11	4.11	0.00	0.07
Lachnospiraceae_ND3007_group	0.56	0.48	0.71	0.27	0.72	0.86	0.05	0.95	0.98	-0.03	0.97	0.98	0.25	0.75	0.89
Lachnospiraceae_NK4A136_group	2.40	0.02	0.10	1.29	0.20	0.53	0.37	0.74	0.93	0.33	0.76	0.95	0.63	0.57	0.79
Lachnospiraceae_UCG.001	1.95	0.02	0.09	1.52	0.05	0.29	1.23	0.14	0.59	1.33	0.12	0.55	1.62	0.06	0.33
Lachnospiraceae_UCG.004	1.26	0.19	0.39	0.95	0.29	0.60	0.76	0.41	0.71	0.73	0.42	0.74	0.92	0.33	0.65
Lachnospiraceae_UCG.010	-0.58	0.65	0.82	-1.23	0.31	0.60	-1.78	0.15	0.59	-1.71	0.16	0.61	-1.58	0.20	0.64
Lactobacillus	-0.40	0.66	0.82	0.24	0.78	0.89	0.00	1.00	1.00	-0.13	0.89	0.97	0.04	0.96	0.97
Lactococcus	2.94	0.07	0.19	1.86	0.22	0.54	2.16	0.18	0.59	2.13	0.18	0.61	2.15	0.19	0.64
Marvinbryantia	1.27	0.15	0.35	0.86	0.30	0.60	0.37	0.68	0.89	0.34	0.71	0.93	0.47	0.60	0.81
Megasphaera	-0.21	0.74	0.85	0.47	0.44	0.68	0.69	0.28	0.71	0.71	0.27	0.68	0.66	0.31	0.64
Monoglobus	2.85	0.01	0.05	2.19	0.02	0.16	1.28	0.21	0.65	1.31	0.20	0.63	1.56	0.14	0.58
Moryella	1.43	0.37	0.60	0.56	0.71	0.86	-0.44	0.78	0.94	-0.45	0.78	0.95	-0.33	0.84	0.93
Negativibacillus	1.03	0.27	0.53	1.37	0.12	0.39	1.31	0.15	0.59	1.37	0.13	0.58	1.53	0.10	0.50
NK4A214_group	0.94	0.22	0.45	0.44	0.55	0.76	-0.03	0.97	0.98	0.08	0.92	0.97	0.30	0.70	0.89
Odoribacter	1.19	0.09	0.22	0.51	0.44	0.68	0.45	0.52	0.82	0.46	0.51	0.81	0.54	0.44	0.73
Oscillibacter	3.15	0.01	0.08	2.97	0.01	0.16	3.17	0.01	0.22	3.40	0.01	0.14	3.74	0.01	0.11
Oscillospira	2.24	0.17	0.36	1.96	0.20	0.52	2.11	0.17	0.59	2.12	0.17	0.61	2.59	0.11	0.50
Oxalobacter	2.37	0.06	0.18	1.26	0.30	0.60	1.17	0.36	0.71	1.23	0.34	0.68	0.87	0.51	0.75
Paludicola	4.61	0.00	0.02	3.83	0.00	0.08	3.41	0.01	0.20	3.66	0.01	0.14	3.79	0.01	0.11
Parabacteroides	0.09	0.92	0.96	0.06	0.94	0.97	0.17	0.85	0.96	0.10	0.91	0.97	0.16	0.86	0.93
Paraprevotella	-0.48	0.46	0.69	-0.36	0.56	0.78	-0.33	0.62	0.88	-0.28	0.67	0.91	-0.14	0.83	0.93
Parasutterella	1.24	0.07	0.20	1.02	0.11	0.39	0.93	0.18	0.59	0.90	0.19	0.61	0.85	0.22	0.64
Phascolarctobacterium	1.00	0.12	0.29	0.93	0.12	0.39	0.73	0.24	0.70	0.78	0.22	0.65	0.66	0.31	0.64
Phocea	0.44	0.78	0.85	-0.09	0.95	0.97	0.46	0.76	0.94	0.40	0.79	0.95	0.53	0.73	0.89
Prevotella	-0.31	0.58	0.79	-0.16	0.76	0.88	-0.16	0.78	0.94	-0.13	0.81	0.95	-0.14	0.81	0.93
Romboutsia	-0.07	0.95	0.96	0.40	0.70	0.85	0.91	0.40	0.71	0.75	0.50	0.81	1.15	0.31	0.64
Roseburia	2.39	0.03	0.12	2.10	0.05	0.27	1.04	0.36	0.71	1.00	0.38	0.68	1.18	0.31	0.64
Rothia	-5.02	0.02	0.08	-5.04	0.01	0.13	-5.83	0.00	0.16	-6.05	0.00	0.12	-5.82	0.01	0.11
Ruminococcus	2.29	0.00	0.03	1.66	0.02	0.16	1.22	0.11	0.58	1.27	0.09	0.51	1.50	0.05	0.31
Sellimonas	0.95	0.40	0.63	0.70	0.51	0.74	0.82	0.45	0.75	0.97	0.37	0.68	1.03	0.35	0.65
Senegalimassilia	-0.48	0.62	0.80	-0.21	0.82	0.91	-0.76	0.42	0.71	-0.71	0.46	0.78	-0.61	0.53	0.76
Slackia	-0.37	0.68	0.82	-0.43	0.61	0.80	-0.46	0.59	0.88	-0.47	0.58	0.84	-0.51	0.55	0.77
Streptococcus	1.89	0.16	0.35	1.73	0.16	0.50	1.33	0.32	0.71	1.24	0.35	0.68	1.66	0.23	0.64
Subdoligranulum	2.81	0.01	0.05	2.36	0.02	0.16	2.01	0.04	0.33	2.06	0.04	0.29	2.28	0.03	0.21
Sutterella	-1.45	0.02	0.09	-1.01	0.08	0.35	-1.00	0.10	0.58	-1.01	0.10	0.51	-0.96	0.12	0.54
TM7x	-0.40	0.78	0.85	-0.57	0.67	0.84	-0.31	0.82	0.96	-0.34	0.80	0.95	-0.16	0.91	0.95
Turicibacter	-1.51	0.33	0.60	-0.71	0.63	0.81	-0.77	0.63	0.88	-0.95	0.55	0.84	-1.01	0.53	0.76
Tuzzerella	0.17	0.88	0.93	0.11	0.91	0.97	0.16	0.88	0.96	0.21	0.85	0.95	0.17	0.88	0.93
UBA1819	1.17	0.41	0.64	0.70	0.60	0.80	1.37	0.32	0.71	1.61	0.25	0.68	1.54	0.28	0.64
UCG.002	1.87	0.01	0.05	1.24	0.07	0.30	1.18	0.10	0.58	1.26	0.08	0.48	1.42	0.05	0.31
UCG.003	0.97	0.24	0.47	0.64	0.41	0.66	0.41	0.61	0.88	0.44	0.58	0.84	0.65	0.43	0.73
UCG.005	2.34	0.01	0.05	1.50	0.07	0.30	0.72	0.41	0.71	0.84	0.34	0.68	1.14	0.21	0.64
UCG.009	1.99	0.17	0.36	0.89	0.51	0.74	-0.11	0.94	0.98	-0.17	0.91	0.97	0.11	0.94	0.97
Veillonella	0.86	0.36	0.60	1.08	0.22	0.54	0.42	0.66	0.88	0.33	0.72	0.93	0.69	0.48	0.74
Victivallis	2.04	0.05	0.15	1.64	0.09	0.36	1.05	0.31	0.71	1.08	0.30	0.68	0.98	0.35	0.65

Multivariable-adjusted regression models adjusted for:
Model 1: sequencing batch, age, race, gender, study center
Model 2: Model 1, with education

© 2022 Meyer K et al. *JAMA Network Open.*

Model 3: Model 2, with physical activity, diet score, medication use, and smoking
Model 4: Model 3 with BMI
Model 5: Model 4 with hypertension and diabetes

© 2022 Meyer K et al. *JAMA Network Open.*

eTable 27: Multivariable-adjusted regression coefficients, p-values, and false discovery rate (FDR)-adjusted p-values for genera among Black participants with respect to Stroop.

	Model1			Model2			Model3			Model4			Model5		
	beta	p-value	FDR	beta	p-value	FDR	beta	p-value	FDR	beta	p-value	FDR	beta	p-value	FDR
Acetanaerobacterium	-0.60	0.71	0.86	-0.59	0.71	0.92	-0.08	0.96	0.99	-0.08	0.96	0.99	-0.09	0.95	0.99
Acidaminococcus	0.36	0.44	0.76	0.17	0.72	0.92	-0.51	0.31	0.89	-0.51	0.31	0.89	-0.56	0.27	0.81
Actinomyces	0.20	0.88	0.97	-0.19	0.88	0.96	-1.24	0.35	0.89	-1.24	0.35	0.89	-1.56	0.25	0.81
Adlercreutzia	-0.11	0.90	0.97	0.12	0.89	0.96	0.15	0.86	0.99	0.15	0.86	0.99	0.14	0.87	0.99
Agathobacter	-0.77	0.48	0.76	-0.63	0.55	0.88	0.16	0.88	0.99	0.16	0.88	0.99	-0.07	0.95	0.99
Akkermansia	-1.12	0.05	0.28	-0.83	0.14	0.56	-0.71	0.22	0.89	-0.71	0.22	0.89	-0.74	0.21	0.81
Alistipes	-0.36	0.71	0.86	-0.05	0.96	0.98	0.27	0.78	0.99	0.26	0.78	0.99	0.23	0.81	0.99
Allisonella	1.00	0.29	0.61	0.14	0.88	0.96	-0.23	0.82	0.99	-0.23	0.82	0.99	-0.20	0.85	0.99
Anaerofilum	-1.39	0.27	0.61	-0.68	0.59	0.88	-0.99	0.44	0.99	-0.99	0.44	0.99	-1.10	0.40	0.92
Anaerostipes	0.13	0.91	0.97	0.49	0.67	0.92	1.20	0.31	0.89	1.21	0.31	0.89	1.15	0.35	0.89
Anaerotruncus	-1.77	0.10	0.41	-1.54	0.14	0.56	-1.21	0.26	0.89	-1.22	0.26	0.89	-1.49	0.17	0.75
Atopobium	3.30	0.08	0.36	2.88	0.11	0.56	2.48	0.19	0.89	2.50	0.19	0.89	1.98	0.31	0.83
Bacteroides	-2.47	0.12	0.47	-2.20	0.16	0.56	-2.20	0.15	0.89	-2.24	0.14	0.89	-2.20	0.15	0.75
Barnesiella	-0.37	0.51	0.76	-0.44	0.42	0.84	-0.26	0.64	0.99	-0.26	0.64	0.99	-0.32	0.58	0.99
Bifidobacterium	0.26	0.72	0.86	0.08	0.91	0.97	0.05	0.94	0.99	0.05	0.95	0.99	0.04	0.95	0.99
Bilophila	-0.33	0.64	0.83	0.05	0.94	0.97	-0.01	0.99	0.99	-0.01	0.99	0.99	-0.02	0.97	0.99
Blautia	-4.37	0.06	0.32	-4.13	0.07	0.40	-4.06	0.08	0.84	-4.09	0.08	0.83	-5.36	0.03	0.71
Butyricicoccus	-3.73	0.02	0.16	-2.83	0.07	0.40	-1.47	0.34	0.89	-1.48	0.34	0.89	-1.68	0.29	0.81
Butyricimonas	-1.42	0.02	0.19	-1.26	0.04	0.40	-1.17	0.06	0.84	-1.17	0.06	0.83	-1.17	0.06	0.74
CAG.56	-0.36	0.61	0.83	-0.31	0.65	0.90	0.49	0.48	0.99	0.49	0.48	0.99	0.27	0.71	0.99
Candidatus_Soleaferrea	-1.48	0.30	0.62	-1.27	0.36	0.83	-2.09	0.13	0.89	-2.09	0.13	0.89	-2.16	0.13	0.74
Caproiciproducens	-0.95	0.45	0.76	-0.76	0.53	0.88	-0.25	0.84	0.99	-0.24	0.85	0.99	-0.51	0.69	0.99
Catenibacterium	-0.42	0.53	0.76	-0.53	0.42	0.84	-0.29	0.67	0.99	-0.29	0.67	0.99	-0.27	0.69	0.99
Christensenellaceae_R.7_group	-1.77	0.00	0.08	-1.41	0.02	0.30	-0.75	0.22	0.89	-0.76	0.22	0.89	-0.99	0.12	0.74
Clostridium_sensu_stricto_1	0.89	0.27	0.61	0.69	0.39	0.84	1.14	0.16	0.89	1.14	0.16	0.89	1.12	0.17	0.75
Colidextribacter	-0.86	0.53	0.76	-0.74	0.58	0.88	-1.49	0.26	0.89	-1.49	0.26	0.89	-2.14	0.12	0.74
Collinsella	0.03	0.96	0.98	-0.41	0.52	0.88	-0.11	0.87	0.99	-0.11	0.87	0.99	-0.09	0.89	0.99
Coprobacter	-1.02	0.20	0.55	-0.85	0.28	0.82	-0.31	0.70	0.99	-0.31	0.70	0.99	-0.22	0.79	0.99
Coprococcus	-0.10	0.90	0.97	-0.26	0.74	0.92	-0.10	0.90	0.99	-0.10	0.90	0.99	-0.23	0.78	0.99
Defluviitaleaceae_UCG.011	-3.59	0.00	0.08	-3.34	0.00	0.14	-2.52	0.03	0.84	-2.54	0.03	0.83	-2.68	0.03	0.71
Desulfovibrio	-0.31	0.62	0.83	-0.34	0.58	0.88	-0.23	0.71	0.99	-0.23	0.72	0.99	-0.26	0.69	0.99
Dialister	0.38	0.45	0.76	0.27	0.59	0.88	0.33	0.53	0.99	0.33	0.53	0.99	0.25	0.64	0.99
DNF00809	-1.94	0.15	0.47	-1.89	0.15	0.56	-2.22	0.09	0.88	-2.22	0.09	0.89	-2.24	0.09	0.74
Dorea	-1.57	0.07	0.36	-1.58	0.07	0.40	-1.69	0.05	0.84	-1.69	0.05	0.83	-1.67	0.05	0.74
DTU089	-1.33	0.21	0.55	-1.01	0.33	0.83	-0.93	0.37	0.90	-0.93	0.37	0.90	-1.16	0.27	0.81
Eggerthella	0.48	0.63	0.83	0.78	0.42	0.84	-0.17	0.87	0.99	-0.17	0.87	0.99	-0.28	0.78	0.99
Eisenbergiella	0.22	0.83	0.93	0.30	0.76	0.92	-0.34	0.73	0.99	-0.33	0.74	0.99	-0.34	0.73	0.99
Enterorhabdus	-1.79	0.09	0.41	-1.62	0.12	0.56	-0.62	0.57	0.99	-0.62	0.58	0.99	-0.87	0.44	0.96
Erysipelatoclostridium	-0.14	0.90	0.97	-0.03	0.98	0.98	-0.34	0.75	0.99	-0.34	0.75	0.99	-0.26	0.81	0.99
Erysipelotrichaceae_UCG.003	-0.51	0.50	0.76	-0.51	0.49	0.88	0.04	0.96	0.99	0.04	0.95	0.99	-0.10	0.90	0.99
Escherichia.Shigella	0.00	1.00	1.00	-0.42	0.55	0.88	-0.44	0.54	0.99	-0.45	0.54	0.99	-0.36	0.63	0.99
Faecalibacterium	-1.39	0.26	0.61	-0.79	0.52	0.88	-0.12	0.92	0.99	-0.12	0.92	0.99	-0.37	0.77	0.99
Family_XIII_AD3011_group	-2.35	0.01	0.14	-1.83	0.06	0.40	-1.02	0.31	0.89	-1.02	0.31	0.89	-1.16	0.25	0.81
Family_XIII_UCG.001	-2.45	0.04	0.28	-2.27	0.05	0.40	-1.28	0.29	0.89	-1.28	0.29	0.89	-1.34	0.27	0.81
FD2005	-0.37	0.73	0.86	-0.97	0.36	0.83	0.11	0.92	0.99	0.11	0.92	0.99	-0.09	0.93	0.99
Flavonifractor	0.68	0.49	0.76	0.76	0.43	0.84	-0.52	0.60	0.99	-0.53	0.60	0.99	-0.73	0.48	0.98
Frisingicoccus	-0.45	0.60	0.83	-0.25	0.76	0.92	-0.37	0.67	0.99	-0.36	0.67	0.99	-0.45	0.60	0.99
Fusicatenibacter	-0.83	0.28	0.61	-0.67	0.37	0.83	-0.73	0.35	0.89	-0.74	0.34	0.89	-0.87	0.28	0.81
Fusobacterium	0.83	0.43	0.76	0.36	0.73	0.92	0.09	0.93	0.99	0.10	0.93	0.99	0.21	0.85	0.99
GCA.900066575	-2.73	0.01	0.14	-2.27	0.04	0.40	-1.20	0.28	0.89	-1.21	0.28	0.89	-1.50	0.18	0.77
Gordonibacter	-0.49	0.67	0.85	0.09	0.93	0.97	0.02	0.99	0.99	0.03	0.98	0.99	0.07	0.95	0.99
Granulicatella	0.64	0.61	0.83	0.25	0.84	0.96	-0.42	0.74	0.99	-0.43	0.74	0.99	-0.73	0.57	0.99
Haemophilus	-0.24	0.75	0.86	-0.11	0.88	0.96	0.08	0.92	0.99	0.08	0.92	0.99	-0.14	0.86	0.99
Harryflintia	-2.33	0.05	0.28	-2.28	0.05	0.40	-1.63	0.17	0.89	-1.64	0.17	0.89	-1.90	0.11	0.74

© 2022 Meyer K et al. *JAMA Network Open.*

Holdemanella	-0.02	0.97	0.98	-0.21	0.77	0.92	0.07	0.93	0.99	0.07	0.93	0.99	0.20	0.79	0.99
Holdemania	-1.94	0.35	0.68	-1.93	0.34	0.83	-2.56	0.21	0.89	-2.56	0.21	0.89	-2.43	0.24	0.81
Howardella	-0.40	0.69	0.86	-0.30	0.76	0.92	0.56	0.58	0.99	0.58	0.57	0.99	0.46	0.66	0.99
Hungatella	1.46	0.14	0.47	1.44	0.13	0.56	0.58	0.56	0.99	0.58	0.56	0.99	0.51	0.61	0.99
Hydrogenoanaerobacterium	-0.98	0.38	0.72	-0.22	0.84	0.96	0.33	0.77	0.99	0.35	0.77	0.99	0.27	0.82	0.99
Incertae_Sedis	-1.50	0.32	0.65	-1.34	0.36	0.83	-1.41	0.35	0.89	-1.41	0.35	0.89	-1.49	0.33	0.88
Intestinibacter	-0.07	0.95	0.98	-0.08	0.94	0.97	-0.03	0.98	0.99	-0.04	0.97	0.99	-0.03	0.98	0.99
Intestinimonas	-1.92	0.03	0.23	-1.43	0.11	0.56	-1.20	0.19	0.89	-1.21	0.19	0.89	-1.29	0.17	0.75
Lachnoclostridium	4.23	0.02	0.19	3.36	0.07	0.40	2.67	0.15	0.89	2.67	0.15	0.89	2.97	0.11	0.74
Lachnospira	-1.25	0.13	0.47	-0.79	0.34	0.83	0.03	0.97	0.99	0.02	0.98	0.99	-0.23	0.79	0.99
Lachnospiraceae_FCS020_group	-2.84	0.00	0.10	-2.33	0.02	0.30	-1.19	0.24	0.89	-1.19	0.24	0.89	-1.29	0.21	0.81
Lachnospiraceae_ND3007_group	-0.80	0.23	0.56	-0.66	0.31	0.83	-0.25	0.70	0.99	-0.26	0.69	0.99	-0.51	0.46	0.96
Lachnospiraceae_NK4A136_group	-0.04	0.96	0.98	0.57	0.51	0.88	1.76	0.05	0.84	1.76	0.05	0.83	1.51	0.11	0.74
Lachnospiraceae_UCG.001	-0.93	0.18	0.51	-0.73	0.28	0.82	0.23	0.74	0.99	0.24	0.74	0.99	0.02	0.98	0.99
Lachnospiraceae_UCG.004	-0.49	0.53	0.76	-0.31	0.69	0.92	-0.12	0.87	0.99	-0.13	0.87	0.99	-0.18	0.82	0.99
Lachnospiraceae_UCG.010	-0.83	0.43	0.76	-0.49	0.64	0.90	0.40	0.70	0.99	0.41	0.69	0.99	0.34	0.75	0.99
Lactobacillus	0.21	0.79	0.89	-0.15	0.84	0.96	-0.19	0.81	0.99	-0.21	0.80	0.99	-0.23	0.78	0.99
Lactococcus	-1.64	0.22	0.56	-1.05	0.42	0.84	-0.18	0.89	0.99	-0.19	0.89	0.99	0.12	0.93	0.99
Marvinbryantia	-0.67	0.36	0.69	-0.43	0.55	0.88	0.13	0.86	0.99	0.13	0.86	0.99	-0.09	0.91	0.99
Megasphaera	0.22	0.67	0.85	-0.14	0.79	0.94	-0.61	0.26	0.89	-0.61	0.26	0.89	-0.49	0.38	0.92
Monoglobus	-2.09	0.01	0.14	-1.78	0.03	0.38	-0.09	0.92	0.99	-0.08	0.92	0.99	-0.26	0.77	0.99
Moryella	-2.23	0.09	0.41	-1.72	0.18	0.57	-0.10	0.94	0.99	-0.09	0.95	0.99	-0.28	0.84	0.99
Negativibacillus	-0.95	0.22	0.56	-1.12	0.14	0.56	-1.37	0.07	0.84	-1.37	0.07	0.83	-1.52	0.05	0.74
NK4A214_group	-1.66	0.01	0.13	-1.42	0.02	0.30	-0.80	0.20	0.89	-0.81	0.20	0.89	-1.06	0.10	0.74
Odoribacter	-0.62	0.28	0.61	-0.25	0.66	0.91	-0.35	0.55	0.99	-0.35	0.55	0.99	-0.48	0.42	0.93
Oscillibacter	-1.49	0.16	0.47	-1.45	0.17	0.56	-1.40	0.20	0.89	-1.42	0.20	0.89	-1.60	0.16	0.75
Oscillospira	-1.27	0.34	0.68	-1.19	0.36	0.83	-1.46	0.26	0.89	-1.46	0.26	0.89	-2.05	0.13	0.74
Oxalobacter	-1.13	0.28	0.61	-0.56	0.60	0.88	0.85	0.43	0.99	0.86	0.43	0.99	1.18	0.29	0.81
Paludicola	-3.06	0.01	0.12	-2.69	0.01	0.30	-1.86	0.10	0.89	-1.89	0.10	0.89	-2.01	0.08	0.74
Parabacteroides	-0.25	0.74	0.86	-0.23	0.76	0.92	-0.28	0.71	0.99	-0.28	0.70	0.99	-0.38	0.61	0.99
Paraprevotella	0.78	0.15	0.47	0.73	0.17	0.56	0.70	0.21	0.89	0.70	0.21	0.89	0.61	0.29	0.81
Parasutterella	-1.42	0.01	0.14	-1.31	0.02	0.30	-1.05	0.07	0.84	-1.05	0.07	0.83	-1.01	0.09	0.74
Phascolarctobacterium	-0.77	0.15	0.47	-0.72	0.17	0.56	-0.52	0.33	0.89	-0.52	0.33	0.89	-0.40	0.46	0.96
Phocea	0.52	0.70	0.86	0.77	0.55	0.88	0.81	0.53	0.99	0.81	0.53	0.99	0.58	0.65	0.99
Prevotella	0.66	0.16	0.47	0.57	0.22	0.66	0.50	0.29	0.89	0.50	0.29	0.89	0.45	0.34	0.89
Romboutsia	0.60	0.51	0.76	0.33	0.72	0.92	0.07	0.94	0.99	0.06	0.95	0.99	-0.14	0.89	0.99
Roseburia	-0.67	0.47	0.76	-0.53	0.55	0.88	0.01	0.99	0.99	0.01	0.99	0.99	-0.30	0.76	0.99
Rothia	2.51	0.15	0.47	2.51	0.14	0.56	0.79	0.65	0.99	0.79	0.65	0.99	0.19	0.91	0.99
Ruminococcus	-1.12	0.07	0.36	-0.82	0.18	0.57	-0.42	0.51	0.99	-0.42	0.51	0.99	-0.58	0.38	0.92
Sellimonas	-0.17	0.85	0.95	-0.06	0.95	0.97	-0.53	0.56	0.99	-0.53	0.56	0.99	-0.60	0.51	0.99
Senegalimassilia	0.03	0.97	0.98	-0.11	0.89	0.96	0.54	0.50	0.99	0.55	0.50	0.99	0.45	0.58	0.99
Slackia	-1.37	0.06	0.32	-1.35	0.06	0.40	-1.02	0.15	0.89	-1.02	0.15	0.89	-1.00	0.17	0.75
Streptococcus	-0.71	0.52	0.76	-0.66	0.54	0.88	-0.74	0.51	0.99	-0.75	0.51	0.99	-0.79	0.50	0.98
Subdoligranulum	-2.91	0.00	0.03	-2.69	0.00	0.12	-2.91	0.00	0.05	-2.91	0.00	0.05	-3.29	0.00	0.01
Sutterella	0.75	0.14	0.47	0.53	0.30	0.83	0.24	0.64	0.99	0.24	0.64	0.99	0.23	0.66	0.99
TM7x	-0.83	0.48	0.76	-0.76	0.51	0.88	-0.85	0.46	0.99	-0.86	0.46	0.99	-1.01	0.39	0.92
Turicibacter	0.41	0.75	0.86	-0.03	0.98	0.98	-0.59	0.66	0.99	-0.61	0.65	0.99	-0.54	0.69	0.99
Tuzzerella	0.42	0.64	0.83	0.43	0.63	0.90	-0.05	0.95	0.99	-0.05	0.96	0.99	0.00	1.00	1.00
UBA1819	-1.40	0.23	0.56	-1.17	0.30	0.83	-2.31	0.04	0.84	-2.36	0.04	0.83	-2.56	0.03	0.71
UCG.002	-0.83	0.16	0.47	-0.49	0.40	0.84	-0.02	0.97	0.99	-0.02	0.97	0.99	-0.22	0.71	0.99
UCG.003	-1.10	0.10	0.41	-0.93	0.16	0.56	-0.69	0.30	0.89	-0.69	0.30	0.89	-0.78	0.26	0.81
UCG.005	-2.42	0.00	0.03	-2.03	0.00	0.14	-1.37	0.06	0.84	-1.39	0.06	0.83	-1.70	0.02	0.71
UCG.009	-0.82	0.50	0.76	-0.25	0.83	0.96	0.67	0.58	0.99	0.67	0.58	0.99	0.42	0.73	0.99
Veillonella	0.56	0.47	0.76	0.41	0.59	0.88	0.71	0.37	0.90	0.71	0.37	0.90	0.60	0.47	0.96
Victivallis	-0.62	0.47	0.76	-0.41	0.62	0.90	0.63	0.47	0.99	0.63	0.47	0.99	0.71	0.42	0.93

Multivariable-adjusted regression models adjusted for:

Model 1: sequencing batch, age, race, gender, study center

Model 2: Model 1, with education

Model 3: Model 2, with physical activity, diet score, medication use, and smoking

© 2022 Meyer K et al. *JAMA Network Open.*

Model 4: Model 3 with BMI
Model 5: Model 4 with hypertension and diabetes

© 2022 Meyer K et al. *JAMA Network Open.*

eTable 28: Multivariable-adjusted regression coefficients, p-values, and false discovery rate (FDR)-adjusted p-values for genera among Black participants with respect to RAVLT.

	Model1			Model2			Model3			Model4			Model5		
	beta	p-value	FDR	beta	p-value	FDR	beta	p-value	FDR	beta	p-value	FDR	beta	p-value	FDR
Acetanaerobacterium	0.00	0.99	0.99	0.01	0.97	1.00	-0.08	0.73	0.89	-0.07	0.75	0.92	-0.09	0.70	0.91
Acidaminococcus	-0.13	0.05	0.49	-0.09	0.15	0.68	-0.06	0.40	0.86	-0.06	0.37	0.85	-0.07	0.35	0.86
Actinomyces	-0.23	0.19	0.63	-0.16	0.34	0.77	-0.18	0.35	0.85	-0.17	0.36	0.85	-0.13	0.49	0.86
Adlercreutzia	0.23	0.05	0.49	0.18	0.11	0.68	0.15	0.23	0.76	0.15	0.22	0.76	0.15	0.22	0.81
Agathobacter	-0.28	0.06	0.50	-0.31	0.03	0.45	-0.35	0.02	0.54	-0.36	0.02	0.47	-0.33	0.03	0.59
Akkermansia	0.13	0.09	0.51	0.07	0.37	0.77	0.05	0.54	0.87	0.05	0.54	0.88	0.06	0.51	0.86
Alistipes	0.17	0.21	0.63	0.10	0.42	0.77	0.07	0.62	0.87	0.06	0.63	0.88	0.07	0.63	0.91
Allisonella	-0.31	0.02	0.33	-0.12	0.33	0.77	-0.08	0.58	0.87	-0.08	0.57	0.88	-0.10	0.49	0.86
Anaerofilum	0.26	0.14	0.63	0.10	0.55	0.88	0.07	0.69	0.88	0.08	0.66	0.88	0.11	0.56	0.89
Anaerostipes	0.06	0.72	0.87	-0.01	0.93	1.00	0.05	0.77	0.91	0.04	0.82	0.95	0.08	0.63	0.91
Anaerotruncus	0.11	0.48	0.79	0.05	0.73	0.91	0.01	0.95	0.96	0.02	0.89	0.97	0.04	0.78	0.93
Atopobium	0.00	0.99	0.99	0.09	0.72	0.91	0.06	0.83	0.95	0.04	0.88	0.97	0.12	0.67	0.91
Bacteroides	0.16	0.47	0.79	0.10	0.62	0.91	0.10	0.65	0.87	0.09	0.69	0.88	0.06	0.80	0.93
Barnesiella	0.02	0.78	0.87	0.03	0.65	0.91	0.05	0.50	0.87	0.05	0.49	0.88	0.06	0.43	0.86
Bifidobacterium	0.09	0.36	0.69	0.14	0.15	0.68	0.14	0.18	0.76	0.13	0.20	0.75	0.15	0.16	0.80
Bilophila	0.16	0.10	0.51	0.08	0.37	0.77	0.10	0.32	0.85	0.10	0.34	0.85	0.11	0.30	0.86
Blautia	0.17	0.59	0.84	0.11	0.72	0.91	0.14	0.66	0.87	0.13	0.69	0.88	0.25	0.49	0.86
Butyricicoccus	0.04	0.86	0.90	-0.20	0.35	0.77	-0.21	0.34	0.85	-0.22	0.32	0.85	-0.17	0.46	0.86
Butyricimonas	0.07	0.42	0.74	0.03	0.70	0.91	0.07	0.45	0.87	0.07	0.44	0.88	0.07	0.42	0.86
CAG.56	-0.04	0.70	0.87	-0.05	0.61	0.91	-0.06	0.53	0.87	-0.07	0.50	0.88	-0.05	0.64	0.91
Candidatus_Soleaferrea	0.21	0.29	0.69	0.16	0.38	0.77	0.14	0.49	0.87	0.14	0.49	0.88	0.16	0.43	0.86
Caproiciproducens	0.05	0.77	0.87	0.00	0.98	1.00	-0.09	0.60	0.87	-0.09	0.62	0.88	-0.06	0.76	0.93
Catenibacterium	-0.16	0.07	0.50	-0.14	0.11	0.68	-0.07	0.49	0.87	-0.07	0.47	0.88	-0.07	0.48	0.86
Christensenellaceae_R.7_group	0.04	0.65	0.85	-0.05	0.52	0.85	-0.10	0.25	0.79	-0.10	0.28	0.85	-0.07	0.41	0.86
Clostridium_sensu_stricto_1	0.00	0.99	0.99	0.04	0.73	0.91	0.03	0.83	0.95	0.02	0.86	0.97	0.03	0.78	0.93
Colidextribacter	-0.18	0.34	0.69	-0.21	0.23	0.68	-0.17	0.37	0.85	-0.17	0.37	0.85	-0.10	0.60	0.91
Collinsella	-0.30	0.00	0.07	-0.21	0.01	0.45	-0.23	0.01	0.54	-0.23	0.01	0.47	-0.24	0.01	0.59
Coprobacter	-0.04	0.70	0.87	-0.08	0.42	0.77	-0.07	0.53	0.87	-0.07	0.57	0.88	-0.07	0.56	0.89
Coprococcus	-0.12	0.28	0.69	-0.09	0.40	0.77	-0.10	0.37	0.85	-0.10	0.36	0.85	-0.09	0.42	0.86
Defluviitaleaceae_UCG.011	0.18	0.27	0.69	0.11	0.46	0.77	0.06	0.72	0.89	0.07	0.67	0.88	0.09	0.60	0.91
Desulfovibrio	-0.11	0.21	0.63	-0.10	0.20	0.68	-0.11	0.24	0.76	-0.10	0.27	0.85	-0.10	0.26	0.83
Dialister	-0.05	0.49	0.80	-0.03	0.67	0.91	-0.06	0.45	0.87	-0.06	0.44	0.88	-0.05	0.51	0.86
DNF00809	-0.20	0.27	0.69	-0.22	0.21	0.68	-0.27	0.16	0.76	-0.26	0.16	0.75	-0.25	0.19	0.80
Dorea	-0.17	0.17	0.63	-0.17	0.15	0.68	-0.16	0.20	0.76	-0.16	0.19	0.75	-0.17	0.18	0.80
DTU089	-0.05	0.75	0.87	-0.13	0.35	0.77	-0.20	0.18	0.76	-0.20	0.18	0.75	-0.19	0.21	0.80
Eggerthella	0.23	0.09	0.51	0.17	0.18	0.68	0.19	0.17	0.76	0.19	0.17	0.75	0.21	0.15	0.80
Eisenbergiella	0.04	0.76	0.87	0.02	0.87	0.99	0.03	0.85	0.95	0.03	0.81	0.95	0.04	0.79	0.93
Enterorhabdus	-0.14	0.33	0.69	-0.18	0.19	0.68	-0.24	0.13	0.75	-0.24	0.13	0.75	-0.20	0.21	0.80
Erysipelatoclostridium	0.33	0.03	0.37	0.30	0.04	0.45	0.33	0.03	0.54	0.33	0.03	0.47	0.33	0.03	0.59
Erysipelotrichaceae_UCG.003	-0.04	0.71	0.87	-0.04	0.71	0.91	-0.10	0.33	0.85	-0.10	0.34	0.85	-0.08	0.48	0.86
Escherichia.Shigella	-0.02	0.84	0.90	0.07	0.46	0.77	0.06	0.60	0.87	0.05	0.63	0.88	0.02	0.86	0.94
Faecalibacterium	0.18	0.30	0.69	0.04	0.79	0.95	0.02	0.92	0.96	0.01	0.96	0.98	0.07	0.68	0.91
Family_XIII_AD3011_group	0.04	0.74	0.87	-0.08	0.53	0.86	-0.15	0.28	0.85	-0.15	0.31	0.85	-0.12	0.41	0.86
Family_XIII_UCG.001	-0.09	0.59	0.84	-0.13	0.42	0.77	-0.11	0.54	0.87	-0.10	0.58	0.88	-0.09	0.62	0.91
FD2005	-0.23	0.11	0.55	-0.11	0.44	0.77	-0.20	0.20	0.76	-0.20	0.21	0.75	-0.20	0.20	0.80
Flavonifractor	0.13	0.36	0.69	0.10	0.43	0.77	0.08	0.60	0.87	0.08	0.60	0.88	0.11	0.46	0.86
Frisingicoccus	0.20	0.08	0.51	0.15	0.16	0.68	0.10	0.43	0.87	0.11	0.39	0.86	0.12	0.35	0.86
Fusicatenibacter	-0.17	0.10	0.51	-0.21	0.04	0.45	-0.24	0.03	0.54	-0.25	0.03	0.47	-0.24	0.04	0.59
Fusobacterium	-0.15	0.31	0.69	-0.05	0.71	0.91	-0.05	0.72	0.89	-0.05	0.76	0.92	-0.07	0.64	0.91
GCA.900066575	0.05	0.76	0.87	-0.06	0.67	0.91	-0.07	0.65	0.87	-0.06	0.69	0.88	-0.02	0.90	0.96
Gordonibacter	0.41	0.01	0.25	0.28	0.06	0.63	0.26	0.12	0.71	0.28	0.10	0.72	0.29	0.09	0.80
Granulicatella	0.07	0.71	0.87	0.14	0.38	0.77	0.15	0.42	0.87	0.14	0.45	0.88	0.18	0.35	0.86
Haemophilus	0.12	0.26	0.69	0.09	0.38	0.77	0.10	0.37	0.85	0.10	0.38	0.85	0.14	0.23	0.81
Harryflintia	0.03	0.85	0.90	0.01	0.96	1.00	-0.02	0.91	0.96	-0.01	0.95	0.98	0.01	0.95	0.98

© 2022 Meyer K et al. *JAMA Network Open.*

Holdemanella	-0.13	0.21	0.63	-0.09	0.36	0.77	-0.07	0.52	0.87	-0.07	0.52	0.88	-0.10	0.39	0.86
Holdemania	0.34	0.23	0.66	0.33	0.21	0.68	0.19	0.52	0.87	0.20	0.50	0.88	0.23	0.45	0.86
Howardella	-0.25	0.07	0.50	-0.28	0.04	0.45	-0.25	0.09	0.71	-0.24	0.10	0.72	-0.24	0.10	0.80
Hungatella	0.03	0.82	0.90	0.03	0.80	0.95	0.01	0.94	0.96	0.01	0.95	0.98	0.00	0.98	0.98
Hydrogenoanaerobacterium	0.16	0.30	0.69	-0.01	0.95	1.00	-0.01	0.93	0.96	0.00	1.00	1.00	0.03	0.87	0.94
Incertae_Sedis	0.10	0.62	0.84	0.06	0.77	0.94	-0.10	0.66	0.87	-0.10	0.65	0.88	-0.08	0.70	0.91
Intestinibacter	0.09	0.51	0.81	0.10	0.45	0.77	0.06	0.68	0.87	0.05	0.76	0.92	0.07	0.66	0.91
Intestinimonas	0.18	0.15	0.63	0.06	0.59	0.91	0.05	0.70	0.88	0.06	0.66	0.88	0.07	0.62	0.91
Lachnoclostridium	-0.20	0.43	0.74	0.00	1.00	1.00	0.15	0.58	0.87	0.14	0.61	0.88	0.11	0.69	0.91
Lachnospira	0.06	0.60	0.84	-0.04	0.69	0.91	-0.15	0.23	0.76	-0.16	0.20	0.75	-0.12	0.34	0.86
Lachnospiraceae_FCS020_group	0.19	0.17	0.63	0.07	0.60	0.91	0.01	0.95	0.96	0.01	0.97	0.98	0.03	0.85	0.94
Lachnospiraceae_ND3007_group	-0.12	0.20	0.63	-0.15	0.08	0.68	-0.19	0.04	0.55	-0.20	0.03	0.47	-0.17	0.07	0.80
Lachnospiraceae_NK4A136_group	0.08	0.51	0.81	-0.05	0.66	0.91	-0.13	0.32	0.85	-0.13	0.31	0.85	-0.09	0.49	0.86
Lachnospiraceae_UCG.001	0.06	0.54	0.82	0.01	0.92	1.00	-0.06	0.59	0.87	-0.05	0.63	0.88	-0.02	0.83	0.94
Lachnospiraceae_UCG.004	0.06	0.60	0.84	0.02	0.83	0.97	0.02	0.85	0.95	0.02	0.87	0.97	0.01	0.92	0.96
Lachnospiraceae_UCG.010	-0.14	0.35	0.69	-0.21	0.13	0.68	-0.21	0.16	0.76	-0.20	0.17	0.75	-0.19	0.21	0.80
Lactobacillus	-0.23	0.02	0.33	-0.16	0.10	0.68	-0.22	0.05	0.60	-0.24	0.04	0.47	-0.24	0.04	0.59
Lactococcus	0.17	0.36	0.69	0.05	0.79	0.95	0.05	0.79	0.92	0.05	0.80	0.95	0.03	0.88	0.94
Marvinbryantia	-0.05	0.62	0.84	-0.10	0.31	0.77	-0.09	0.43	0.87	-0.09	0.41	0.87	-0.07	0.53	0.88
Megasphaera	-0.06	0.37	0.70	0.01	0.89	1.00	0.05	0.52	0.87	0.05	0.51	0.88	0.03	0.69	0.91
Monoglobus	0.13	0.28	0.69	0.05	0.64	0.91	-0.02	0.86	0.95	-0.02	0.88	0.97	0.01	0.91	0.96
Moryella	-0.07	0.71	0.87	-0.17	0.33	0.77	-0.26	0.17	0.76	-0.27	0.17	0.75	-0.26	0.18	0.80
Negativibacillus	-0.14	0.18	0.63	-0.11	0.29	0.77	-0.10	0.36	0.85	-0.10	0.38	0.85	-0.09	0.41	0.86
NK4A214_group	-0.07	0.43	0.74	-0.13	0.12	0.68	-0.15	0.09	0.71	-0.15	0.11	0.72	-0.12	0.19	0.80
Odoribacter	0.15	0.05	0.49	0.08	0.31	0.77	0.08	0.34	0.85	0.08	0.33	0.85	0.09	0.29	0.86
Oscillibacter	0.15	0.32	0.69	0.13	0.36	0.77	0.07	0.65	0.87	0.09	0.58	0.88	0.12	0.45	0.86
Oscillospira	0.05	0.77	0.87	0.02	0.90	1.00	-0.02	0.90	0.96	-0.02	0.91	0.98	0.04	0.82	0.93
Oxalobacter	0.02	0.88	0.92	-0.11	0.43	0.77	-0.12	0.45	0.87	-0.11	0.46	0.88	-0.15	0.34	0.86
Paludicola	0.08	0.60	0.84	-0.01	0.95	1.00	-0.02	0.89	0.96	-0.01	0.97	0.98	0.02	0.93	0.96
Parabacteroides	0.05	0.64	0.85	0.05	0.64	0.91	0.06	0.56	0.87	0.06	0.60	0.88	0.06	0.56	0.89
Paraprevotella	-0.10	0.17	0.63	-0.09	0.21	0.68	-0.08	0.34	0.85	-0.07	0.36	0.85	-0.07	0.40	0.86
Parasutterella	0.20	0.01	0.25	0.18	0.01	0.45	0.15	0.08	0.71	0.14	0.08	0.72	0.15	0.09	0.80
Phascolarctobacterium	0.11	0.13	0.62	0.10	0.13	0.68	0.11	0.16	0.76	0.11	0.15	0.75	0.11	0.18	0.80
Phocea	-0.33	0.07	0.50	-0.39	0.02	0.45	-0.40	0.03	0.54	-0.41	0.03	0.47	-0.39	0.04	0.59
Prevotella	-0.06	0.32	0.69	-0.05	0.44	0.77	-0.02	0.75	0.90	-0.02	0.78	0.93	-0.02	0.81	0.93
Romboutsia	-0.06	0.61	0.84	-0.01	0.92	1.00	-0.06	0.64	0.87	-0.08	0.55	0.88	-0.04	0.79	0.93
Roseburia	-0.07	0.60	0.84	-0.10	0.41	0.77	-0.25	0.07	0.71	-0.25	0.07	0.72	-0.22	0.12	0.80
Rothia	-0.32	0.19	0.63	-0.32	0.16	0.68	-0.40	0.11	0.71	-0.42	0.09	0.72	-0.37	0.15	0.80
Ruminococcus	0.07	0.41	0.73	0.00	0.96	1.00	-0.06	0.54	0.87	-0.05	0.57	0.88	-0.03	0.77	0.93
Sellimonas	-0.02	0.86	0.90	-0.05	0.67	0.91	-0.06	0.64	0.87	-0.05	0.70	0.88	-0.04	0.74	0.93
Senegalimassilia	-0.26	0.02	0.33	-0.23	0.03	0.45	-0.24	0.03	0.54	-0.24	0.04	0.47	-0.22	0.05	0.72
Slackia	-0.25	0.01	0.25	-0.26	0.01	0.45	-0.27	0.01	0.54	-0.28	0.01	0.47	-0.27	0.01	0.59
Streptococcus	0.20	0.18	0.63	0.18	0.20	0.68	0.20	0.21	0.76	0.19	0.23	0.76	0.23	0.17	0.80
Subdoligranulum	0.07	0.54	0.82	0.02	0.85	0.99	0.01	0.95	0.96	0.01	0.93	0.98	0.03	0.79	0.93
Sutterella	-0.18	0.01	0.25	-0.13	0.05	0.52	-0.09	0.23	0.76	-0.09	0.23	0.76	-0.09	0.24	0.82
TM7x	-0.20	0.21	0.63	-0.22	0.14	0.68	-0.15	0.37	0.85	-0.15	0.36	0.85	-0.13	0.44	0.86
Turicibacter	0.11	0.53	0.82	0.20	0.23	0.68	0.14	0.46	0.87	0.13	0.50	0.88	0.13	0.50	0.86
Tuzzerella	0.05	0.71	0.87	0.04	0.73	0.91	0.00	0.98	0.98	0.00	0.99	1.00	0.00	0.98	0.98
UBA1819	0.04	0.83	0.90	-0.02	0.91	1.00	-0.07	0.68	0.87	-0.05	0.75	0.92	-0.05	0.76	0.93
UCG.002	0.07	0.36	0.69	0.00	0.99	1.00	-0.02	0.79	0.92	-0.02	0.84	0.96	0.00	0.98	0.98
UCG.003	-0.08	0.38	0.70	-0.12	0.18	0.68	-0.15	0.12	0.71	-0.15	0.12	0.75	-0.14	0.16	0.80
UCG.005	-0.02	0.86	0.90	-0.12	0.21	0.68	-0.19	0.08	0.71	-0.18	0.09	0.72	-0.15	0.17	0.80
UCG.009	0.00	0.98	0.99	-0.12	0.43	0.77	-0.22	0.19	0.76	-0.23	0.18	0.75	-0.20	0.25	0.83
Veillonella	0.10	0.36	0.69	0.12	0.22	0.68	0.10	0.38	0.85	0.09	0.42	0.87	0.13	0.29	0.86
Victivallis	-0.10	0.38	0.70	-0.15	0.18	0.68	-0.20	0.11	0.71	-0.20	0.11	0.72	-0.21	0.10	0.80

Multivariable-adjusted regression models adjusted for:
Model 1: sequencing batch, age, race, gender, study center
Model 2: Model 1, with education

© 2022 Meyer K et al. *JAMA Network Open.*

Model 3: Model 2, with physical activity, diet score, medication use, and smoking
Model 4: Model 3 with BMI
Model 5: Model 4 with hypertension and diabetes

© 2022 Meyer K et al. *JAMA Network Open.*

eTable 29: Multivariable-adjusted regression coefficients, p-values, and false discovery rate (FDR)-adjusted p-values for genera among Black participants with respect to category fluency.

	Model1			Model2			Model3			Model4			Model5		
	beta	p-value	FDR	beta	p-value	FDR	beta	p-value	FDR	beta	p-value	FDR	beta	p-value	FDR
Acetanaerobacterium	-0.53	0.39	0.67	-0.53	0.38	0.72	-0.46	0.49	0.79	-0.48	0.47	0.79	-0.44	0.51	0.86
Acidaminococcus	-0.25	0.16	0.44	-0.18	0.31	0.71	-0.19	0.35	0.75	-0.18	0.39	0.79	-0.17	0.42	0.81
Actinomyces	-0.85	0.08	0.36	-0.72	0.13	0.52	-0.75	0.16	0.69	-0.76	0.16	0.72	-0.78	0.16	0.77
Adlercreutzia	-0.31	0.36	0.65	-0.41	0.20	0.60	-0.59	0.10	0.69	-0.60	0.09	0.72	-0.62	0.09	0.77
Agathobacter	-0.08	0.85	0.94	-0.13	0.74	0.90	-0.29	0.51	0.79	-0.25	0.57	0.86	-0.21	0.64	0.88
Akkermansia	0.37	0.09	0.38	0.24	0.26	0.67	0.23	0.33	0.75	0.23	0.33	0.74	0.20	0.40	0.81
Alistipes	0.44	0.23	0.52	0.32	0.38	0.72	0.07	0.85	0.99	0.08	0.83	0.98	0.09	0.81	0.93
Allisonella	0.16	0.65	0.85	0.58	0.11	0.52	0.61	0.14	0.69	0.61	0.14	0.72	0.65	0.12	0.77
Anaerofilum	0.68	0.16	0.44	0.38	0.43	0.76	0.39	0.46	0.78	0.34	0.52	0.81	0.28	0.60	0.88
Anaerostipes	-0.02	0.97	0.99	-0.16	0.72	0.90	-0.18	0.71	0.95	-0.12	0.81	0.98	-0.13	0.79	0.93
Anaerotruncus	0.40	0.34	0.65	0.29	0.48	0.77	0.27	0.54	0.80	0.22	0.63	0.91	0.27	0.55	0.88
Atopobium	-0.88	0.22	0.52	-0.71	0.31	0.71	-0.90	0.25	0.69	-0.83	0.29	0.72	-0.77	0.33	0.81
Bacteroides	0.33	0.59	0.79	0.22	0.71	0.90	0.16	0.80	0.99	0.25	0.70	0.94	0.23	0.72	0.91
Barnesiella	0.45	0.03	0.30	0.47	0.02	0.33	0.54	0.02	0.66	0.53	0.02	0.71	0.52	0.03	0.77
Bifidobacterium	0.38	0.18	0.47	0.46	0.09	0.52	0.48	0.10	0.69	0.53	0.07	0.71	0.52	0.08	0.77
Bilophila	0.79	0.00	0.08	0.65	0.02	0.33	0.53	0.07	0.69	0.56	0.05	0.71	0.52	0.08	0.77
Blautia	-0.84	0.35	0.65	-0.96	0.27	0.67	-1.10	0.25	0.69	-1.03	0.28	0.72	-1.13	0.27	0.80
Butyricicoccus	0.72	0.23	0.52	0.29	0.63	0.88	0.48	0.45	0.77	0.52	0.41	0.79	0.51	0.43	0.83
Butyricimonas	0.70	0.00	0.08	0.63	0.01	0.33	0.74	0.00	0.38	0.73	0.00	0.41	0.76	0.00	0.33
CAG.56	-0.01	0.98	0.99	-0.02	0.93	0.96	-0.04	0.89	0.99	-0.01	0.96	0.98	-0.01	0.96	0.99
Candidatus_Soleaferrea	0.10	0.85	0.94	0.01	0.98	0.98	-0.03	0.95	0.99	-0.04	0.95	0.98	0.00	1.00	1.00
Caproiciproducens	-0.33	0.48	0.75	-0.43	0.36	0.72	-0.47	0.37	0.76	-0.49	0.34	0.74	-0.54	0.30	0.80
Catenibacterium	0.01	0.95	0.99	0.07	0.79	0.91	0.01	0.97	0.99	0.03	0.91	0.98	0.02	0.94	0.98
Christensenellaceae_R.7_group	0.62	0.01	0.12	0.46	0.04	0.43	0.41	0.10	0.69	0.39	0.13	0.72	0.38	0.14	0.77
Clostridium_sensu_stricto_1	-0.18	0.57	0.79	-0.11	0.73	0.90	-0.13	0.70	0.95	-0.11	0.75	0.96	-0.15	0.66	0.88
Colidextribacter	-0.44	0.40	0.67	-0.51	0.32	0.72	-0.51	0.35	0.75	-0.51	0.35	0.74	-0.49	0.39	0.81
Collinsella	0.25	0.31	0.63	0.45	0.06	0.47	0.39	0.14	0.69	0.39	0.14	0.72	0.36	0.19	0.77
Coprobacter	0.43	0.16	0.44	0.35	0.24	0.65	0.28	0.40	0.76	0.25	0.46	0.79	0.19	0.57	0.88
Coprococcus	0.09	0.77	0.92	0.16	0.60	0.88	0.29	0.37	0.76	0.30	0.34	0.74	0.34	0.29	0.80
Defluviitaleaceae_UCG.011	0.54	0.23	0.52	0.41	0.35	0.72	0.56	0.25	0.69	0.51	0.30	0.72	0.49	0.32	0.81
Desulfovibrio	0.29	0.23	0.52	0.29	0.21	0.60	0.23	0.38	0.76	0.19	0.47	0.79	0.19	0.48	0.85
Dialister	-0.28	0.15	0.44	-0.24	0.20	0.60	-0.23	0.28	0.72	-0.22	0.29	0.72	-0.19	0.37	0.81
DNF00809	0.09	0.86	0.94	0.06	0.91	0.95	0.35	0.51	0.79	0.33	0.54	0.82	0.30	0.58	0.88
Dorea	0.06	0.86	0.94	0.07	0.84	0.94	0.06	0.87	0.99	0.08	0.83	0.98	0.06	0.86	0.95
DTU089	-0.25	0.54	0.79	-0.41	0.30	0.71	-0.50	0.24	0.69	-0.52	0.22	0.72	-0.40	0.35	0.81
Eggerthella	-0.98	0.01	0.13	-1.10	0.00	0.29	-1.03	0.01	0.61	-1.03	0.01	0.63	-1.03	0.01	0.69
Eisenbergiella	-0.09	0.82	0.94	-0.13	0.73	0.90	-0.14	0.73	0.95	-0.18	0.65	0.92	-0.19	0.63	0.88
Enterorhabdus	0.61	0.13	0.44	0.55	0.17	0.56	0.95	0.04	0.66	0.94	0.04	0.71	0.99	0.03	0.77
Erysipelatoclostridium	-0.07	0.87	0.94	-0.13	0.75	0.90	-0.16	0.72	0.95	-0.18	0.68	0.94	-0.21	0.64	0.88
Erysipelotrichaceae_UCG.003	0.02	0.94	0.98	0.03	0.92	0.95	0.01	0.99	0.99	-0.01	0.99	0.99	-0.03	0.91	0.96
Escherichia.Shigella	-0.41	0.13	0.44	-0.25	0.35	0.72	-0.35	0.24	0.69	-0.33	0.27	0.72	-0.36	0.25	0.79
Faecalibacterium	0.45	0.35	0.65	0.19	0.68	0.90	0.18	0.72	0.95	0.22	0.65	0.92	0.25	0.62	0.88
Family_XIII_AD3011_group	0.76	0.04	0.30	0.53	0.15	0.52	0.54	0.19	0.69	0.49	0.24	0.72	0.46	0.27	0.80
Family_XIII_UCG.001	0.81	0.08	0.36	0.74	0.10	0.52	0.74	0.13	0.69	0.69	0.16	0.72	0.65	0.19	0.77
FD2005	-0.76	0.06	0.36	-0.52	0.20	0.60	-0.51	0.25	0.69	-0.51	0.25	0.72	-0.47	0.30	0.80
Flavonifractor	-0.42	0.27	0.58	-0.46	0.21	0.60	-0.50	0.23	0.69	-0.49	0.24	0.72	-0.49	0.25	0.79
Frisingicoccus	0.31	0.34	0.65	0.22	0.49	0.79	0.05	0.88	0.99	0.02	0.96	0.98	0.00	1.00	1.00
Fusicatenibacter	0.16	0.58	0.79	0.10	0.72	0.90	-0.08	0.80	0.99	-0.04	0.90	0.98	-0.14	0.67	0.88
Fusobacterium	-0.07	0.86	0.94	0.12	0.76	0.90	0.20	0.65	0.93	0.16	0.71	0.95	0.20	0.65	0.88
GCA.900066575	0.80	0.06	0.36	0.59	0.15	0.52	0.58	0.20	0.69	0.54	0.24	0.72	0.58	0.22	0.79
Gordonibacter	0.31	0.49	0.75	0.06	0.90	0.95	0.25	0.59	0.86	0.19	0.70	0.94	0.12	0.81	0.93
Granulicatella	-0.41	0.39	0.67	-0.27	0.58	0.87	-0.36	0.49	0.79	-0.32	0.54	0.82	-0.22	0.67	0.88
Haemophilus	0.27	0.36	0.65	0.21	0.46	0.77	0.06	0.86	0.99	0.07	0.83	0.98	0.12	0.72	0.91
Harryflintia	-0.17	0.71	0.89	-0.21	0.63	0.88	-0.50	0.31	0.74	-0.55	0.26	0.72	-0.54	0.28	0.80

© 2022 Meyer K et al. *JAMA Network Open.*

Holdemanella	0.23	0.42	0.69	0.31	0.27	0.67	0.49	0.11	0.69	0.50	0.11	0.72	0.50	0.12	0.77
Holdemania	-0.39	0.62	0.81	-0.40	0.60	0.88	-0.22	0.80	0.99	-0.28	0.74	0.96	-0.39	0.65	0.88
Howardella	-0.01	0.99	0.99	-0.05	0.90	0.95	0.04	0.93	0.99	-0.02	0.97	0.98	0.00	1.00	1.00
Hungatella	-0.57	0.13	0.44	-0.57	0.12	0.52	-0.57	0.16	0.69	-0.56	0.16	0.72	-0.53	0.20	0.77
Hydrogenoanaerobacterium	1.20	0.00	0.09	0.91	0.03	0.39	0.99	0.04	0.66	0.93	0.05	0.71	0.89	0.06	0.77
Incertae_Sedis	-0.42	0.48	0.75	-0.50	0.37	0.72	-0.54	0.39	0.76	-0.52	0.40	0.79	-0.55	0.38	0.81
Intestinibacter	-0.30	0.44	0.71	-0.29	0.45	0.76	-0.35	0.40	0.76	-0.28	0.52	0.81	-0.28	0.51	0.86
Intestinimonas	1.01	0.00	0.08	0.80	0.02	0.33	0.74	0.05	0.66	0.70	0.06	0.71	0.73	0.06	0.77
Lachnoclostridium	-1.66	0.02	0.25	-1.29	0.07	0.47	-1.11	0.15	0.69	-1.06	0.17	0.72	-1.08	0.16	0.77
Lachnospira	0.45	0.16	0.44	0.26	0.42	0.75	-0.02	0.95	0.99	0.03	0.93	0.98	0.06	0.87	0.95
Lachnospiraceae_FCS020_group	0.68	0.08	0.36	0.45	0.24	0.65	0.53	0.21	0.69	0.54	0.19	0.72	0.49	0.25	0.79
Lachnospiraceae_ND3007_grou	0.43	0.09	0.38	0.37	0.14	0.52	0.32	0.23	0.69	0.36	0.18	0.72	0.37	0.19	0.77
Lachnospiraceae_NK4A136_gro	0.49	0.15	0.44	0.25	0.46	0.76	0.07	0.86	0.99	0.08	0.83	0.98	0.12	0.76	0.93
Lachnospiraceae_UCG.001	0.31	0.25	0.54	0.21	0.41	0.75	0.09	0.76	0.97	0.05	0.85	0.98	0.07	0.82	0.93
Lachnospiraceae_UCG.004	0.39	0.19	0.47	0.33	0.27	0.67	0.25	0.43	0.77	0.26	0.41	0.79	0.27	0.40	0.81
Lachnospiraceae_UCG.010	-0.22	0.59	0.79	-0.36	0.36	0.72	-0.28	0.52	0.79	-0.31	0.46	0.79	-0.29	0.50	0.86
Lactobacillus	-0.19	0.52	0.78	-0.05	0.87	0.94	-0.11	0.73	0.95	-0.05	0.87	0.98	-0.05	0.88	0.95
Lactococcus	1.01	0.05	0.31	0.78	0.12	0.52	0.57	0.31	0.74	0.58	0.30	0.72	0.48	0.40	0.81
Marvinbryantia	0.01	0.96	0.99	-0.08	0.77	0.90	0.00	0.99	0.99	0.01	0.97	0.98	0.08	0.79	0.93
Megasphaera	-0.20	0.31	0.63	-0.06	0.76	0.90	-0.02	0.93	0.99	-0.03	0.91	0.98	-0.05	0.83	0.93
Monoglobus	0.34	0.30	0.63	0.19	0.55	0.85	-0.04	0.91	0.99	-0.05	0.88	0.98	-0.13	0.73	0.91
Moryella	0.41	0.41	0.68	0.23	0.64	0.88	0.08	0.88	0.99	0.09	0.88	0.98	0.09	0.87	0.95
Negativibacillus	-0.06	0.83	0.94	0.01	0.97	0.98	-0.02	0.96	0.99	-0.04	0.89	0.98	-0.08	0.80	0.93
NK4A214_group	0.49	0.04	0.30	0.38	0.11	0.52	0.29	0.27	0.71	0.25	0.34	0.74	0.26	0.33	0.81
Odoribacter	0.63	0.00	0.08	0.50	0.02	0.33	0.38	0.12	0.69	0.37	0.12	0.72	0.38	0.12	0.77
Oscillibacter	0.16	0.70	0.89	0.12	0.76	0.90	0.47	0.30	0.74	0.40	0.38	0.79	0.36	0.45	0.83
Oscillospira	-0.68	0.18	0.47	-0.74	0.14	0.52	-0.73	0.18	0.69	-0.73	0.17	0.72	-0.75	0.18	0.77
Oxalobacter	0.59	0.14	0.44	0.35	0.37	0.72	0.34	0.44	0.77	0.32	0.47	0.79	0.26	0.57	0.88
Paludicola	0.25	0.57	0.79	0.07	0.87	0.94	-0.07	0.87	0.99	-0.16	0.74	0.96	-0.22	0.65	0.88
Parabacteroides	0.46	0.12	0.44	0.45	0.11	0.52	0.29	0.34	0.75	0.33	0.28	0.72	0.37	0.23	0.79
Paraprevotella	0.02	0.92	0.97	0.05	0.81	0.92	0.04	0.87	0.99	0.02	0.95	0.98	0.03	0.91	0.96
Parasutterella	0.36	0.10	0.39	0.31	0.14	0.52	0.22	0.35	0.75	0.23	0.33	0.74	0.18	0.46	0.83
Phascolarctobacterium	0.44	0.03	0.30	0.43	0.03	0.39	0.48	0.03	0.66	0.46	0.04	0.71	0.45	0.05	0.77
Phocea	-0.73	0.15	0.44	-0.84	0.09	0.52	-0.96	0.07	0.69	-0.93	0.08	0.71	-0.90	0.09	0.77
Prevotella	0.10	0.58	0.79	0.13	0.45	0.76	0.17	0.39	0.76	0.16	0.42	0.79	0.17	0.37	0.81
Romboutsia	0.06	0.86	0.94	0.17	0.63	0.88	0.16	0.67	0.95	0.26	0.51	0.81	0.27	0.49	0.86
Roseburia	0.13	0.72	0.89	0.06	0.85	0.94	-0.30	0.45	0.77	-0.28	0.48	0.79	-0.20	0.63	0.88
Rothia	-0.40	0.55	0.79	-0.40	0.53	0.83	-0.93	0.19	0.69	-0.87	0.23	0.72	-0.63	0.39	0.81
Ruminococcus	0.49	0.04	0.30	0.35	0.14	0.52	0.21	0.43	0.77	0.19	0.48	0.79	0.16	0.56	0.88
Sellimonas	-0.11	0.77	0.92	-0.16	0.64	0.88	-0.25	0.50	0.79	-0.32	0.40	0.79	-0.32	0.39	0.81
Senegalimassilia	0.55	0.08	0.36	0.61	0.04	0.43	0.66	0.05	0.66	0.63	0.05	0.71	0.64	0.05	0.77
Slackia	0.41	0.15	0.44	0.39	0.15	0.52	0.37	0.21	0.69	0.37	0.21	0.72	0.35	0.25	0.79
Streptococcus	0.37	0.38	0.67	0.34	0.41	0.75	0.29	0.53	0.80	0.34	0.47	0.79	0.36	0.46	0.83
Subdoligranulum	0.70	0.03	0.30	0.60	0.06	0.47	0.46	0.19	0.69	0.44	0.21	0.72	0.51	0.15	0.77
Sutterella	-0.17	0.37	0.67	-0.08	0.69	0.90	-0.09	0.68	0.95	-0.08	0.69	0.94	-0.06	0.78	0.93
TM7x	-0.79	0.08	0.36	-0.83	0.06	0.47	-0.63	0.19	0.69	-0.61	0.20	0.72	-0.67	0.16	0.77
Turicibacter	0.28	0.57	0.79	0.46	0.34	0.72	0.69	0.22	0.69	0.78	0.17	0.72	0.77	0.17	0.77
Tuzzerella	0.18	0.60	0.79	0.17	0.61	0.88	-0.03	0.93	0.99	-0.05	0.89	0.98	-0.10	0.79	0.93
UBA1819	0.09	0.84	0.94	-0.01	0.97	0.98	0.02	0.96	0.99	-0.07	0.89	0.98	-0.05	0.92	0.96
UCG.002	0.37	0.10	0.39	0.23	0.30	0.71	0.19	0.45	0.77	0.16	0.51	0.81	0.21	0.41	0.81
UCG.003	0.54	0.04	0.30	0.47	0.06	0.47	0.41	0.14	0.69	0.39	0.16	0.72	0.36	0.20	0.77
UCG.005	0.82	0.00	0.08	0.65	0.02	0.33	0.58	0.05	0.66	0.55	0.07	0.71	0.55	0.08	0.77
UCG.009	0.15	0.74	0.91	-0.09	0.83	0.94	-0.27	0.58	0.86	-0.25	0.62	0.91	-0.24	0.64	0.88
Veillonella	0.04	0.90	0.96	0.09	0.77	0.90	0.07	0.84	0.99	0.11	0.75	0.96	0.15	0.65	0.88
Victivallis	-0.12	0.71	0.89	-0.21	0.50	0.80	-0.39	0.28	0.72	-0.40	0.27	0.72	-0.43	0.24	0.79

Multivariable-adjusted regression models adjusted for:
Model 1: sequencing batch, age, race, gender, study center
Model 2: Model 1, with education

© 2022 Meyer K et al. *JAMA Network Open.*

Model 3: Model 2, with physical activity, diet score, medication use, and smoking
Model 4: Model 3 with BMI
Model 5: Model 4 with hypertension and diabetes

© 2022 Meyer K et al. *JAMA Network Open.*

eTable 30: Multivariable-adjusted regression coefficients, p-values, and false discovery rate (FDR)-adjusted p-values for genera among Black participants with respect to letter fluency.

	Model1			Model2			Model3			Model4			Model5		
	beta	p-value	FDR	beta	p-value	FDR	beta	p-value	FDR	beta	p-value	FDR	beta	p-value	FDR
Acetanaerobacterium	0.16	0.92	0.99	0.15	0.92	0.99	0.65	0.70	0.93	0.66	0.70	0.94	0.93	0.58	0.91
Acidaminococcus	-0.34	0.47	0.79	-0.14	0.75	0.97	-0.17	0.74	0.93	-0.18	0.73	0.94	-0.09	0.86	0.96
Actinomyces	0.04	0.98	0.99	0.44	0.72	0.97	0.81	0.55	0.90	0.82	0.54	0.91	1.02	0.46	0.89
Adlercreutzia	0.10	0.91	0.99	-0.19	0.82	0.99	0.12	0.89	0.99	0.13	0.88	0.99	0.24	0.79	0.93
Agathobacter	0.46	0.69	0.88	0.35	0.75	0.97	-0.27	0.82	0.96	-0.30	0.80	0.95	-0.22	0.86	0.96
Akkermansia	0.68	0.23	0.66	0.31	0.57	0.97	0.52	0.39	0.90	0.52	0.39	0.89	0.42	0.49	0.89
Alistipes	0.80	0.40	0.77	0.41	0.66	0.97	-0.05	0.96	0.99	-0.05	0.96	0.99	-0.02	0.98	0.99
Allisonella	-0.15	0.87	0.99	0.98	0.30	0.82	1.21	0.24	0.89	1.21	0.24	0.85	1.27	0.23	0.89
Anaerofilum	1.39	0.27	0.69	0.56	0.65	0.97	0.95	0.47	0.90	0.99	0.45	0.89	0.87	0.51	0.90
Anaerostipes	-1.04	0.39	0.77	-1.44	0.22	0.82	-0.81	0.51	0.90	-0.86	0.49	0.89	-0.91	0.48	0.89
Anaerotruncus	0.67	0.53	0.79	0.34	0.75	0.97	1.08	0.33	0.90	1.13	0.31	0.89	1.38	0.22	0.89
Atopobium	0.94	0.61	0.82	1.46	0.42	0.84	0.31	0.87	0.99	0.26	0.89	0.99	0.39	0.84	0.96
Bacteroides	1.64	0.30	0.69	1.30	0.40	0.82	0.98	0.53	0.90	0.94	0.55	0.91	1.01	0.52	0.90
Barnesiella	1.20	0.03	0.45	1.29	0.01	0.71	1.19	0.04	0.89	1.20	0.04	0.85	1.15	0.05	0.89
Bifidobacterium	0.61	0.41	0.77	0.89	0.21	0.82	0.95	0.20	0.89	0.93	0.21	0.85	0.85	0.26	0.89
Bilophila	1.16	0.10	0.54	0.70	0.31	0.82	0.74	0.31	0.90	0.73	0.32	0.89	0.67	0.37	0.89
Blautia	0.03	0.99	0.99	-0.38	0.87	0.99	0.47	0.84	0.98	0.43	0.86	0.99	1.13	0.66	0.93
Butyricicoccus	2.03	0.19	0.65	0.81	0.60	0.97	1.11	0.49	0.90	1.09	0.50	0.89	0.97	0.56	0.91
Butyricimonas	1.37	0.03	0.45	1.20	0.05	0.71	1.15	0.07	0.89	1.15	0.07	0.85	1.13	0.08	0.89
CAG.56	-0.01	0.99	0.99	-0.04	0.96	0.99	0.32	0.66	0.93	0.31	0.67	0.94	0.36	0.63	0.91
Candidatus_Soleaferrea	0.53	0.71	0.89	0.22	0.87	0.99	0.47	0.75	0.93	0.47	0.75	0.94	0.55	0.71	0.93
Caproiciproducens	1.83	0.14	0.59	1.61	0.18	0.79	0.85	0.51	0.90	0.87	0.50	0.89	0.96	0.47	0.89
Catenibacterium	0.03	0.96	0.99	0.20	0.76	0.97	0.35	0.61	0.92	0.34	0.62	0.92	0.34	0.62	0.91
Christensenellaceae_R.7_group	1.22	0.04	0.47	0.79	0.18	0.79	0.70	0.27	0.89	0.73	0.25	0.85	0.77	0.24	0.89
Clostridium_sensu_stricto_1	-0.89	0.27	0.69	-0.66	0.40	0.82	-0.38	0.65	0.93	-0.40	0.64	0.94	-0.47	0.58	0.91
Colidextribacter	-0.07	0.96	0.99	-0.29	0.83	0.99	-0.43	0.75	0.93	-0.43	0.76	0.94	-0.26	0.85	0.96
Collinsella	-0.17	0.79	0.93	0.38	0.55	0.97	-0.01	0.99	0.99	-0.01	0.99	1.00	-0.10	0.88	0.97
Coprobacter	1.15	0.16	0.64	0.85	0.28	0.82	0.46	0.59	0.92	0.49	0.57	0.92	0.30	0.73	0.93
Coprococcus	0.49	0.54	0.79	0.65	0.40	0.82	0.71	0.38	0.90	0.70	0.38	0.89	0.78	0.33	0.89
Defluviitaleaceae_UCG.011	1.63	0.17	0.64	1.22	0.29	0.82	1.68	0.17	0.89	1.73	0.16	0.85	1.71	0.17	0.89
Desulfovibrio	0.41	0.52	0.79	0.44	0.47	0.88	0.67	0.30	0.90	0.71	0.28	0.89	0.73	0.27	0.89
Dialister	-0.86	0.09	0.51	-0.76	0.12	0.71	-0.62	0.24	0.89	-0.62	0.24	0.85	-0.51	0.34	0.89
DNF00809	-1.40	0.30	0.69	-1.52	0.25	0.82	-1.01	0.47	0.90	-1.00	0.47	0.89	-1.04	0.46	0.89
Dorea	0.48	0.59	0.82	0.49	0.57	0.97	0.55	0.54	0.90	0.54	0.55	0.91	0.48	0.59	0.91
DTU089	0.79	0.45	0.79	0.36	0.73	0.97	-0.06	0.96	0.99	-0.05	0.96	0.99	0.30	0.78	0.93
Eggerthella	-1.45	0.14	0.59	-1.87	0.05	0.71	-1.57	0.13	0.89	-1.57	0.13	0.85	-1.48	0.16	0.89
Eisenbergiella	-1.35	0.18	0.64	-1.45	0.14	0.72	-1.50	0.14	0.89	-1.48	0.15	0.85	-1.49	0.15	0.89
Enterorhabdus	1.79	0.09	0.51	1.64	0.11	0.71	2.62	0.02	0.73	2.62	0.02	0.74	2.62	0.02	0.83
Erysipelatoclostridium	0.60	0.58	0.82	0.41	0.70	0.97	0.30	0.78	0.95	0.31	0.78	0.94	0.31	0.78	0.93
Erysipelotrichaceae_UCG.003	1.13	0.13	0.59	1.14	0.12	0.71	0.76	0.32	0.90	0.77	0.31	0.89	0.68	0.38	0.89
Escherichia.Shigella	-0.46	0.51	0.79	0.03	0.96	0.99	-0.06	0.94	0.99	-0.07	0.92	0.99	-0.02	0.98	0.99
Faecalibacterium	0.96	0.45	0.79	0.25	0.84	0.99	0.09	0.95	0.99	0.06	0.96	0.99	-0.03	0.98	0.99
Family_XIII_AD3011_group	1.43	0.14	0.59	0.82	0.39	0.82	1.21	0.24	0.89	1.26	0.23	0.85	1.19	0.26	0.89
Family_XIII_UCG.001	1.39	0.25	0.69	1.21	0.30	0.82	1.77	0.15	0.89	1.83	0.14	0.85	1.76	0.16	0.89
FD2005	-0.79	0.46	0.79	-0.13	0.90	0.99	0.13	0.91	0.99	0.13	0.91	0.99	0.32	0.78	0.93
Flavonifractor	-0.68	0.49	0.79	-0.82	0.39	0.82	-1.03	0.32	0.90	-1.04	0.32	0.89	-0.93	0.38	0.89
Frisingicoccus	0.43	0.62	0.82	0.09	0.92	0.99	-0.06	0.95	0.99	-0.04	0.97	0.99	-0.02	0.98	0.99
Fusicatenibacter	0.22	0.77	0.93	0.03	0.96	0.99	-0.51	0.52	0.90	-0.54	0.50	0.89	-0.75	0.36	0.89
Fusobacterium	0.55	0.61	0.82	1.03	0.33	0.82	0.70	0.53	0.90	0.72	0.51	0.89	0.80	0.48	0.89
GCA.900066575	2.01	0.07	0.47	1.47	0.17	0.79	1.35	0.24	0.89	1.40	0.23	0.85	1.46	0.21	0.89
Gordonibacter	1.79	0.13	0.59	1.03	0.38	0.82	1.47	0.23	0.89	1.54	0.21	0.85	1.44	0.25	0.89
Granulicatella	-0.02	0.99	0.99	0.44	0.72	0.97	1.00	0.45	0.90	0.97	0.46	0.89	1.24	0.36	0.89
Haemophilus	0.25	0.74	0.91	0.12	0.87	0.99	0.22	0.78	0.95	0.21	0.79	0.94	0.26	0.75	0.93
Harryflintia	-1.15	0.33	0.69	-1.25	0.27	0.82	-1.17	0.35	0.90	-1.15	0.36	0.89	-1.03	0.41	0.89

© 2022 Meyer K et al. *JAMA Network Open.*

Holdemanella	0.53	0.48	0.79	0.78	0.28	0.82	1.45	0.06	0.89	1.45	0.06	0.85	1.54	0.05	0.89
Holdemania	-2.36	0.27	0.69	-2.50	0.22	0.82	-1.49	0.50	0.90	-1.46	0.51	0.89	-1.80	0.42	0.89
Howardella	-1.21	0.23	0.67	-1.32	0.18	0.79	-0.90	0.39	0.90	-0.88	0.41	0.89	-0.74	0.49	0.89
Hungatella	-1.54	0.12	0.59	-1.53	0.11	0.71	-1.71	0.09	0.89	-1.71	0.09	0.85	-1.62	0.11	0.89
Hydrogenoanaerobacterium	2.25	0.04	0.47	1.42	0.20	0.82	1.39	0.24	0.89	1.47	0.22	0.85	1.39	0.25	0.89
Incertae_Sedis	0.27	0.86	0.99	0.08	0.96	0.99	0.52	0.74	0.93	0.51	0.75	0.94	0.57	0.72	0.93
Intestinibacter	-0.66	0.52	0.79	-0.59	0.55	0.97	-0.69	0.51	0.90	-0.77	0.47	0.89	-0.83	0.44	0.89
Intestinimonas	0.90	0.31	0.69	0.27	0.76	0.97	0.67	0.48	0.90	0.70	0.47	0.89	0.77	0.42	0.89
Lachnoclostridium	-1.85	0.33	0.69	-0.82	0.66	0.97	0.24	0.90	0.99	0.21	0.91	0.99	0.15	0.94	0.99
Lachnospira	0.78	0.35	0.71	0.24	0.77	0.98	0.04	0.97	0.99	0.00	1.00	1.00	0.06	0.95	0.99
Lachnospiraceae_FCS020_group	2.88	0.00	0.24	2.27	0.02	0.71	2.66	0.01	0.59	2.66	0.01	0.61	2.63	0.01	0.73
Lachnospiraceae_ND3007_group	1.28	0.05	0.47	1.12	0.08	0.71	1.07	0.11	0.89	1.06	0.12	0.85	1.08	0.12	0.89
Lachnospiraceae_NK4A136_group	0.31	0.72	0.89	-0.39	0.65	0.97	-0.38	0.69	0.93	-0.38	0.69	0.94	-0.31	0.75	0.93
Lachnospiraceae_UCG.001	0.30	0.67	0.87	0.02	0.98	0.99	0.19	0.80	0.96	0.21	0.78	0.94	0.26	0.73	0.93
Lachnospiraceae_UCG.004	0.23	0.77	0.93	0.06	0.94	0.99	0.18	0.82	0.96	0.18	0.83	0.97	0.21	0.80	0.93
Lachnospiraceae_UCG.010	-0.57	0.58	0.82	-0.96	0.35	0.82	-0.82	0.44	0.90	-0.80	0.45	0.89	-0.83	0.44	0.89
Lactobacillus	-0.54	0.47	0.79	-0.13	0.86	0.99	-0.28	0.72	0.93	-0.33	0.69	0.94	-0.36	0.67	0.93
Lactococcus	2.77	0.04	0.47	2.16	0.09	0.71	2.60	0.06	0.89	2.59	0.06	0.85	2.33	0.10	0.89
Marvinbryantia	0.78	0.28	0.69	0.52	0.46	0.88	0.27	0.72	0.93	0.27	0.73	0.94	0.46	0.56	0.91
Megasphaera	-0.43	0.41	0.77	-0.02	0.96	0.99	0.05	0.93	0.99	0.05	0.93	0.99	0.03	0.96	0.99
Monoglobus	0.84	0.32	0.69	0.41	0.62	0.97	0.46	0.61	0.92	0.46	0.60	0.92	0.34	0.71	0.93
Moryella	1.61	0.22	0.65	1.14	0.37	0.82	0.69	0.62	0.92	0.69	0.62	0.92	0.76	0.59	0.91
Negativibacillus	1.03	0.18	0.64	1.21	0.11	0.71	1.23	0.12	0.89	1.25	0.11	0.85	1.34	0.10	0.89
NK4A214_group	0.81	0.20	0.65	0.52	0.40	0.82	0.21	0.75	0.93	0.24	0.71	0.94	0.27	0.69	0.93
Odoribacter	1.43	0.01	0.41	1.08	0.05	0.71	0.70	0.24	0.89	0.70	0.24	0.85	0.77	0.21	0.89
Oscillibacter	-0.09	0.93	0.99	-0.22	0.83	0.99	0.60	0.60	0.92	0.66	0.56	0.92	0.66	0.58	0.91
Oscillospira	1.42	0.29	0.69	1.19	0.36	0.82	1.16	0.39	0.90	1.16	0.39	0.89	1.45	0.30	0.89
Oxalobacter	1.42	0.18	0.64	0.76	0.46	0.88	1.25	0.26	0.89	1.27	0.26	0.85	1.23	0.28	0.89
Paludicola	1.42	0.21	0.65	0.86	0.44	0.87	0.96	0.42	0.90	1.03	0.39	0.89	0.94	0.44	0.89
Parabacteroides	1.37	0.07	0.47	1.31	0.08	0.71	0.89	0.24	0.89	0.88	0.25	0.85	0.96	0.21	0.89
Paraprevotella	-0.03	0.95	0.99	0.05	0.92	0.99	-0.10	0.87	0.99	-0.08	0.89	0.99	0.01	0.99	0.99
Parasutterella	0.72	0.20	0.65	0.59	0.28	0.82	0.64	0.28	0.90	0.64	0.29	0.89	0.51	0.40	0.89
Phascolarctobacterium	1.51	0.00	0.24	1.48	0.00	0.42	1.70	0.00	0.22	1.72	0.00	0.20	1.66	0.00	0.37
Phocea	-0.15	0.91	0.99	-0.45	0.72	0.97	-0.69	0.60	0.92	-0.71	0.59	0.92	-0.56	0.68	0.93
Prevotella	-0.35	0.46	0.79	-0.25	0.58	0.97	-0.23	0.64	0.93	-0.22	0.65	0.94	-0.18	0.71	0.93
Romboutsia	-1.64	0.07	0.47	-1.31	0.14	0.72	-1.24	0.19	0.89	-1.34	0.17	0.85	-1.44	0.15	0.89
Roseburia	0.55	0.55	0.80	0.35	0.70	0.97	-0.15	0.88	0.99	-0.16	0.87	0.99	0.05	0.96	0.99
Rothia	-0.67	0.70	0.88	-0.64	0.70	0.97	-1.34	0.45	0.90	-1.40	0.44	0.89	-0.93	0.61	0.91
Ruminococcus	0.49	0.43	0.79	0.08	0.89	0.99	0.22	0.74	0.93	0.23	0.73	0.94	0.16	0.81	0.94
Sellimonas	0.10	0.91	0.99	-0.01	0.99	0.99	-0.54	0.56	0.91	-0.51	0.58	0.92	-0.46	0.62	0.91
Senegalimassilia	0.67	0.41	0.77	0.87	0.27	0.82	0.98	0.23	0.89	1.00	0.22	0.85	1.04	0.21	0.89
Slackia	0.31	0.67	0.87	0.31	0.66	0.97	0.55	0.45	0.90	0.55	0.46	0.89	0.50	0.50	0.89
Streptococcus	0.02	0.99	0.99	-0.03	0.98	0.99	0.93	0.42	0.90	0.91	0.43	0.89	0.82	0.50	0.89
Subdoligranulum	1.54	0.07	0.47	1.25	0.13	0.72	1.14	0.19	0.89	1.15	0.19	0.85	1.41	0.11	0.89
Sutterella	-0.14	0.79	0.93	0.16	0.75	0.97	-0.01	0.98	0.99	-0.02	0.98	0.99	0.01	0.99	0.99
TM7x	-1.23	0.29	0.69	-1.36	0.23	0.82	-0.51	0.67	0.93	-0.53	0.66	0.94	-0.60	0.62	0.91
Turicibacter	-0.66	0.61	0.82	-0.17	0.89	0.99	0.56	0.68	0.93	0.52	0.71	0.94	0.49	0.72	0.93
Tuzzerella	1.64	0.07	0.47	1.54	0.08	0.71	1.21	0.21	0.89	1.22	0.20	0.85	1.15	0.23	0.89
UBA1819	0.77	0.51	0.79	0.43	0.71	0.97	0.91	0.45	0.90	0.99	0.42	0.89	1.25	0.32	0.89
UCG.002	1.32	0.02	0.45	0.95	0.10	0.71	0.68	0.27	0.89	0.71	0.26	0.85	0.86	0.18	0.89
UCG.003	1.26	0.06	0.47	1.06	0.11	0.71	0.97	0.17	0.89	0.98	0.16	0.85	0.87	0.22	0.89
UCG.005	1.73	0.02	0.41	1.28	0.07	0.71	1.33	0.08	0.89	1.39	0.07	0.85	1.42	0.07	0.89
UCG.009	0.13	0.91	0.99	-0.54	0.64	0.97	-0.98	0.43	0.90	-1.00	0.42	0.89	-0.99	0.43	0.89
Veillonella	-0.84	0.28	0.69	-0.66	0.38	0.82	-0.51	0.53	0.90	-0.53	0.52	0.89	-0.59	0.49	0.89
Victivallis	-0.52	0.54	0.79	-0.76	0.35	0.82	-1.07	0.23	0.89	-1.06	0.24	0.85	-1.10	0.22	0.89

Multivariable-adjusted regression models adjusted for:
Model 1: sequencing batch, age, race, gender, study center
Model 2: Model 1, with education

© 2022 Meyer K et al. *JAMA Network Open.*

Model 3: Model 2, with physical activity, diet score, medication use, and smoking
Model 4: Model 3 with BMI
Model 5: Model 4 with hypertension and diabetes

© 2022 Meyer K et al. *JAMA Network Open.*

eTable 31: Multivariable-adjusted regression coefficients, p-values, and false discovery rate (FDR)-adjusted p-values for genera among Black participants with respect to MOCA.

	Model2			Model2			Model3			Model4			Model5		
	beta	p-value	FDR	beta	p-value	FDR	beta	p-value	FDR	beta	p-value	FDR	beta	p-value	FDR
Acetanaerobacterium	-0.26	0.59	0.77	-0.25	0.57	0.80	-0.40	0.39	0.85	-0.39	0.41	0.87	-0.37	0.44	0.83
Acidaminococcus	-0.23	0.11	0.37	-0.13	0.31	0.79	-0.08	0.59	0.89	-0.09	0.55	0.88	-0.06	0.67	0.91
Actinomyces	-0.53	0.16	0.43	-0.37	0.30	0.79	-0.38	0.32	0.85	-0.37	0.33	0.83	-0.23	0.55	0.87
Adlercreutzia	0.27	0.30	0.57	0.15	0.54	0.80	0.01	0.97	0.99	0.02	0.95	0.98	0.06	0.83	0.97
Agathobacter	-0.50	0.12	0.38	-0.57	0.06	0.75	-0.60	0.05	0.70	-0.63	0.04	0.60	-0.57	0.07	0.62
Akkermansia	0.41	0.01	0.24	0.26	0.11	0.79	0.20	0.24	0.85	0.20	0.24	0.78	0.19	0.25	0.79
Alistipes	0.37	0.20	0.47	0.22	0.43	0.80	0.09	0.73	0.97	0.09	0.75	0.94	0.09	0.73	0.96
Allisonella	-0.52	0.07	0.30	-0.07	0.79	0.96	-0.08	0.77	0.99	-0.09	0.77	0.94	-0.16	0.59	0.87
Anaerofilum	0.33	0.38	0.62	-0.04	0.91	0.98	-0.07	0.85	0.99	-0.04	0.91	0.98	0.00	1.00	1.00
Anaerostipes	0.13	0.71	0.83	-0.04	0.90	0.98	-0.08	0.81	0.99	-0.12	0.73	0.94	-0.02	0.95	1.00
Anaerotruncus	0.59	0.07	0.30	0.46	0.13	0.79	0.69	0.03	0.56	0.73	0.02	0.40	0.82	0.01	0.27
Atopobium	0.08	0.88	0.93	0.29	0.57	0.80	-0.05	0.93	0.99	-0.10	0.86	0.98	0.00	1.00	1.00
Bacteroides	0.36	0.46	0.70	0.23	0.61	0.80	0.20	0.65	0.94	0.16	0.73	0.94	0.15	0.74	0.96
Barnesiella	-0.03	0.84	0.91	-0.01	0.97	1.00	0.10	0.55	0.89	0.10	0.53	0.88	0.10	0.54	0.87
Bifidobacterium	-0.02	0.93	0.95	0.08	0.70	0.87	0.11	0.60	0.89	0.09	0.68	0.94	0.08	0.69	0.92
Bilophila	0.29	0.18	0.45	0.10	0.62	0.80	-0.07	0.72	0.97	-0.09	0.66	0.93	-0.04	0.86	0.99
Blautia	-0.33	0.64	0.79	-0.48	0.46	0.80	-0.36	0.59	0.89	-0.40	0.55	0.88	-0.08	0.91	1.00
Butyricicoccus	0.26	0.58	0.77	-0.28	0.53	0.80	-0.43	0.35	0.85	-0.45	0.32	0.83	-0.37	0.43	0.83
Butyricimonas	0.24	0.21	0.47	0.15	0.40	0.80	0.25	0.16	0.84	0.25	0.16	0.71	0.23	0.21	0.76
CAG.56	-0.11	0.60	0.77	-0.13	0.50	0.80	-0.08	0.70	0.96	-0.10	0.64	0.93	-0.05	0.80	0.97
Candidatus_Soleaferrea	0.04	0.92	0.95	-0.07	0.87	0.98	-0.03	0.95	0.99	-0.02	0.95	0.98	0.04	0.93	1.00
Caproiciproducens	0.06	0.87	0.93	-0.05	0.89	0.98	-0.05	0.90	0.99	-0.03	0.93	0.98	0.10	0.80	0.97
Catenibacterium	-0.43	0.03	0.24	-0.37	0.04	0.68	-0.27	0.16	0.84	-0.29	0.14	0.71	-0.27	0.17	0.69
Christensenellaceae_R.7_group	0.41	0.02	0.24	0.21	0.21	0.79	0.23	0.20	0.84	0.25	0.16	0.71	0.33	0.08	0.62
Clostridium_sensu_stricto_1	-0.25	0.30	0.57	-0.17	0.46	0.80	-0.19	0.41	0.85	-0.21	0.37	0.87	-0.16	0.49	0.87
Colidextribacter	-0.37	0.37	0.62	-0.45	0.24	0.79	-0.27	0.49	0.89	-0.27	0.49	0.87	-0.12	0.76	0.97
Collinsella	-0.25	0.19	0.45	-0.03	0.85	0.98	-0.04	0.84	0.99	-0.04	0.84	0.98	-0.05	0.81	0.97
Coprobacter	0.35	0.15	0.41	0.25	0.26	0.79	0.30	0.19	0.84	0.33	0.16	0.71	0.32	0.17	0.69
Coprococcus	-0.05	0.84	0.91	0.03	0.89	0.98	0.19	0.41	0.85	0.18	0.43	0.87	0.19	0.40	0.83
Defluviitaleaceae_UCG.011	0.72	0.04	0.25	0.56	0.09	0.79	0.55	0.11	0.84	0.58	0.09	0.71	0.62	0.08	0.62
Desulfovibrio	-0.09	0.64	0.79	-0.08	0.64	0.81	0.00	0.99	0.99	0.02	0.91	0.98	0.00	0.99	1.00
Dialister	-0.25	0.09	0.34	-0.21	0.15	0.79	-0.21	0.16	0.84	-0.21	0.16	0.71	-0.18	0.24	0.78
DNF00809	-0.28	0.49	0.71	-0.32	0.39	0.80	0.13	0.74	0.97	0.14	0.71	0.94	0.19	0.63	0.87
Dorea	0.16	0.56	0.76	0.16	0.51	0.80	0.22	0.38	0.85	0.21	0.40	0.87	0.20	0.43	0.83
DTU089	0.34	0.28	0.55	0.15	0.60	0.80	0.12	0.69	0.96	0.13	0.66	0.93	0.16	0.60	0.87
Eggerthella	-0.30	0.32	0.59	-0.44	0.11	0.79	-0.27	0.35	0.85	-0.27	0.35	0.84	-0.22	0.45	0.84
Eisenbergiella	0.07	0.82	0.91	0.02	0.95	1.00	0.03	0.93	0.99	0.05	0.86	0.98	0.07	0.82	0.97
Enterorhabdus	0.30	0.35	0.62	0.21	0.47	0.80	0.43	0.18	0.84	0.43	0.18	0.71	0.46	0.16	0.69
Erysipelatoclostridium	0.24	0.47	0.71	0.17	0.59	0.80	0.28	0.36	0.85	0.30	0.34	0.84	0.32	0.30	0.79
Erysipelotrichaceae_UCG.003	0.31	0.17	0.45	0.31	0.13	0.79	0.17	0.44	0.86	0.17	0.42	0.87	0.22	0.32	0.79
Escherichia.Shigella	-0.08	0.70	0.83	0.13	0.53	0.80	0.04	0.84	0.99	0.03	0.89	0.98	-0.03	0.90	1.00
Faecalibacterium	0.70	0.06	0.30	0.39	0.27	0.79	0.37	0.29	0.85	0.35	0.32	0.83	0.44	0.22	0.76
Family_XIII_AD3011_group	0.50	0.08	0.34	0.22	0.42	0.80	0.35	0.23	0.85	0.39	0.19	0.71	0.44	0.14	0.69
Family_XIII_UCG.001	0.36	0.32	0.59	0.27	0.42	0.80	0.33	0.35	0.85	0.36	0.30	0.83	0.38	0.28	0.79
FD2005	-0.57	0.07	0.32	-0.27	0.37	0.80	-0.26	0.41	0.85	-0.26	0.41	0.87	-0.27	0.39	0.82
Flavonifractor	-0.10	0.74	0.85	-0.16	0.57	0.80	-0.02	0.95	0.99	-0.02	0.94	0.98	0.09	0.77	0.97
Frisingicoccus	0.64	0.01	0.24	0.53	0.03	0.53	0.42	0.09	0.84	0.44	0.07	0.71	0.50	0.04	0.58
Fusicatenibacter	-0.13	0.56	0.76	-0.21	0.33	0.79	-0.35	0.12	0.84	-0.38	0.09	0.71	-0.33	0.15	0.69
Fusobacterium	-0.47	0.14	0.41	-0.23	0.43	0.80	-0.27	0.39	0.85	-0.25	0.42	0.87	-0.32	0.30	0.79
GCA.900066575	0.78	0.02	0.24	0.53	0.09	0.79	0.41	0.20	0.84	0.44	0.17	0.71	0.54	0.10	0.68
Gordonibacter	0.31	0.37	0.62	0.00	1.00	1.00	0.02	0.96	0.99	0.06	0.86	0.98	0.10	0.78	0.97
Granulicatella	-0.24	0.53	0.73	-0.05	0.88	0.98	-0.23	0.53	0.89	-0.26	0.48	0.87	-0.21	0.58	0.87
Haemophilus	0.32	0.16	0.43	0.25	0.25	0.79	-0.01	0.96	0.99	-0.02	0.94	0.98	0.02	0.93	1.00
Harryflintia	-0.13	0.71	0.83	-0.19	0.57	0.80	-0.25	0.47	0.87	-0.22	0.53	0.88	-0.17	0.62	0.87

© 2022 Meyer K et al. *JAMA Network Open.*

Holdemanella	-0.14	0.52	0.73	-0.05	0.81	0.96	0.23	0.30	0.85	0.23	0.30	0.83	0.21	0.34	0.82
Holdemania	-0.49	0.43	0.67	-0.51	0.38	0.80	-0.47	0.43	0.86	-0.44	0.46	0.87	-0.41	0.50	0.87
Howardella	-0.33	0.28	0.55	-0.39	0.18	0.79	-0.23	0.45	0.86	-0.20	0.50	0.87	-0.15	0.61	0.87
Hungatella	-0.22	0.45	0.70	-0.22	0.42	0.80	-0.17	0.55	0.89	-0.18	0.54	0.88	-0.19	0.51	0.87
Hydrogenoanaerobacterium	0.79	0.02	0.24	0.41	0.20	0.79	0.35	0.30	0.85	0.40	0.23	0.78	0.48	0.16	0.69
Incertae_Sedis	0.13	0.77	0.87	0.03	0.95	1.00	0.23	0.61	0.89	0.22	0.62	0.93	0.29	0.51	0.87
Intestinibacter	0.21	0.49	0.71	0.23	0.42	0.80	0.18	0.54	0.89	0.13	0.65	0.93	0.17	0.56	0.87
Intestinimonas	0.44	0.10	0.35	0.16	0.52	0.80	0.03	0.91	0.99	0.05	0.84	0.98	0.07	0.79	0.97
Lachnoclostridium	-1.19	0.04	0.25	-0.72	0.17	0.79	-0.62	0.25	0.85	-0.66	0.22	0.78	-0.70	0.20	0.76
Lachnospira	0.56	0.02	0.24	0.33	0.17	0.79	-0.05	0.83	0.99	-0.09	0.73	0.94	0.01	0.97	1.00
Lachnospiraceae_FCS020_group	0.90	0.00	0.11	0.62	0.03	0.53	0.60	0.04	0.70	0.59	0.04	0.60	0.68	0.02	0.43
Lachnospiraceae_ND3007_group	0.18	0.38	0.62	0.10	0.59	0.80	0.16	0.39	0.85	0.14	0.45	0.87	0.22	0.26	0.79
Lachnospiraceae_NK4A136_grou	0.31	0.24	0.50	0.01	0.97	1.00	-0.31	0.25	0.85	-0.32	0.23	0.78	-0.24	0.38	0.82
Lachnospiraceae_UCG.001	0.32	0.12	0.38	0.21	0.29	0.79	0.03	0.90	0.99	0.05	0.82	0.98	0.10	0.62	0.87
Lachnospiraceae_UCG.004	0.49	0.04	0.25	0.41	0.06	0.75	0.33	0.14	0.84	0.33	0.14	0.71	0.34	0.13	0.69
Lachnospiraceae_UCG.010	0.13	0.67	0.82	-0.03	0.91	0.98	-0.03	0.92	0.99	-0.01	0.97	0.98	0.00	0.99	1.00
Lactobacillus	-0.43	0.06	0.29	-0.27	0.21	0.79	-0.27	0.23	0.85	-0.31	0.17	0.71	-0.34	0.15	0.69
Lactococcus	0.81	0.04	0.25	0.52	0.16	0.79	0.54	0.17	0.84	0.54	0.17	0.71	0.51	0.20	0.76
Marvinbryantia	0.24	0.28	0.55	0.13	0.53	0.80	0.41	0.06	0.72	0.40	0.07	0.71	0.47	0.03	0.50
Megasphaera	-0.38	0.02	0.24	-0.21	0.16	0.79	-0.12	0.45	0.86	-0.11	0.47	0.87	-0.14	0.37	0.82
Monoglobus	0.45	0.08	0.32	0.27	0.25	0.79	0.07	0.78	0.99	0.08	0.76	0.94	0.15	0.56	0.87
Moryella	0.21	0.59	0.77	-0.02	0.96	1.00	0.04	0.92	0.99	0.04	0.93	0.98	0.04	0.93	1.00
Negativibacillus	-0.09	0.70	0.83	0.00	1.00	1.00	0.09	0.69	0.96	0.10	0.64	0.93	0.18	0.42	0.83
NK4A214_group	0.25	0.19	0.45	0.12	0.51	0.80	0.08	0.66	0.94	0.11	0.56	0.88	0.17	0.38	0.82
Odoribacter	0.37	0.03	0.24	0.20	0.22	0.79	0.18	0.29	0.85	0.18	0.28	0.83	0.20	0.24	0.78
Oscillibacter	0.28	0.39	0.63	0.23	0.44	0.80	0.44	0.16	0.84	0.50	0.12	0.71	0.65	0.05	0.58
Oscillospira	-0.01	0.98	0.98	-0.08	0.83	0.98	0.01	0.98	0.99	0.01	0.97	0.98	0.19	0.63	0.87
Oxalobacter	0.16	0.62	0.79	-0.15	0.61	0.80	-0.18	0.56	0.89	-0.17	0.59	0.90	-0.23	0.48	0.87
Paludicola	0.52	0.13	0.38	0.31	0.33	0.79	0.18	0.58	0.89	0.24	0.48	0.87	0.30	0.38	0.82
Parabacteroides	0.15	0.53	0.73	0.14	0.52	0.80	0.14	0.51	0.89	0.12	0.57	0.88	0.13	0.55	0.87
Paraprevotella	-0.15	0.34	0.61	-0.12	0.42	0.80	-0.25	0.13	0.84	-0.23	0.15	0.71	-0.20	0.23	0.78
Parasutterella	0.59	0.00	0.03	0.53	0.00	0.04	0.45	0.01	0.33	0.44	0.01	0.30	0.44	0.01	0.27
Phascolarctobacterium	0.34	0.03	0.24	0.33	0.03	0.53	0.30	0.05	0.70	0.32	0.04	0.60	0.28	0.08	0.62
Phocea	-0.66	0.10	0.34	-0.80	0.03	0.53	-0.88	0.02	0.50	-0.89	0.02	0.40	-0.85	0.02	0.43
Prevotella	-0.17	0.23	0.49	-0.13	0.32	0.79	-0.12	0.37	0.85	-0.11	0.40	0.87	-0.12	0.38	0.82
Romboutsia	-0.46	0.09	0.34	-0.34	0.18	0.79	-0.29	0.27	0.85	-0.36	0.19	0.71	-0.29	0.31	0.79
Roseburia	0.34	0.22	0.48	0.27	0.30	0.79	-0.07	0.80	0.99	-0.08	0.77	0.94	-0.01	0.97	1.00
Rothia	-0.86	0.10	0.34	-0.87	0.07	0.78	-1.31	0.01	0.33	-1.37	0.01	0.30	-1.35	0.01	0.27
Ruminococcus	0.36	0.06	0.29	0.18	0.30	0.79	0.13	0.47	0.87	0.15	0.43	0.87	0.21	0.27	0.79
Sellimonas	0.06	0.83	0.91	-0.01	0.98	1.00	0.06	0.83	0.99	0.09	0.73	0.94	0.14	0.60	0.87
Senegalimassilia	-0.30	0.22	0.48	-0.23	0.31	0.79	-0.14	0.55	0.89	-0.12	0.60	0.91	-0.09	0.69	0.92
Slackia	-0.10	0.65	0.79	-0.11	0.57	0.80	-0.05	0.81	0.99	-0.05	0.80	0.97	-0.04	0.86	0.99
Streptococcus	0.24	0.48	0.71	0.19	0.53	0.80	0.03	0.93	0.99	0.01	0.99	0.99	0.01	0.96	1.00
Subdoligranulum	0.38	0.14	0.40	0.26	0.27	0.79	0.37	0.13	0.84	0.39	0.12	0.71	0.47	0.06	0.62
Sutterella	-0.59	0.00	0.01	-0.48	0.00	0.04	-0.46	0.00	0.21	-0.46	0.00	0.20	-0.48	0.00	0.15
TM7x	-0.05	0.90	0.94	-0.09	0.78	0.95	0.12	0.72	0.97	0.11	0.73	0.94	0.18	0.60	0.87
Turicibacter	-0.02	0.96	0.97	0.19	0.59	0.80	0.44	0.25	0.85	0.41	0.30	0.83	0.41	0.30	0.79
Tuzzerella	0.36	0.18	0.45	0.35	0.17	0.79	0.36	0.18	0.84	0.37	0.16	0.71	0.39	0.14	0.69
UBA1819	0.24	0.49	0.71	0.12	0.72	0.89	0.20	0.54	0.89	0.26	0.44	0.87	0.35	0.31	0.79
UCG.002	0.37	0.04	0.25	0.20	0.24	0.79	0.18	0.29	0.85	0.20	0.25	0.81	0.25	0.16	0.69
UCG.003	0.18	0.38	0.62	0.09	0.63	0.81	0.00	0.99	0.99	0.01	0.96	0.98	0.03	0.88	1.00
UCG.005	0.48	0.02	0.24	0.26	0.20	0.79	0.21	0.34	0.85	0.24	0.27	0.83	0.32	0.15	0.69
UCG.009	0.10	0.77	0.87	-0.19	0.56	0.80	-0.33	0.33	0.85	-0.35	0.31	0.83	-0.31	0.38	0.82
Veillonella	-0.01	0.96	0.97	0.05	0.83	0.98	-0.13	0.56	0.89	-0.16	0.49	0.87	-0.15	0.52	0.87
Victivallis	-0.26	0.32	0.59	-0.37	0.12	0.79	-0.42	0.10	0.84	-0.42	0.10	0.71	-0.43	0.09	0.67

Multivariable-adjusted regression models adjusted for:
Model 1: sequencing batch, age, race, gender, study center
Model 2: Model 1, with education

© 2022 Meyer K et al. *JAMA Network Open.*

Model 3: Model 2, with physical activity, diet score, medication use, and smoking
Model 4: Model 3 with BMI
Model 5: Model 4 with hypertension and diabetes

© 2022 Meyer K et al. *JAMA Network Open.*

eTable 32: Multivariable-adjusted regression coefficients, p-values, and false discovery rate (FDR)-adjusted p-values for genera among Black participants with respect to PC (1st).

	Model1			Model2			Model3			Model4			Model5		
	beta	p-value	FDR	beta	p-value	FDR	beta	p-value	FDR	beta	p-value	FDR	beta	p-value	FDR
Acetanaerobacterium	-0.05	0.81	0.86	-0.04	0.81	0.87	-0.06	0.74	0.89	-0.06	0.75	0.89	-0.06	0.77	0.92
Acidaminococcus	-0.09	0.11	0.29	-0.05	0.34	0.67	-0.02	0.73	0.89	-0.02	0.72	0.89	-0.01	0.81	0.93
Actinomyces	-0.26	0.10	0.29	-0.18	0.21	0.61	-0.15	0.33	0.75	-0.15	0.34	0.74	-0.12	0.47	0.86
Adlercreutzia	0.13	0.24	0.44	0.07	0.48	0.75	0.05	0.64	0.89	0.05	0.63	0.89	0.06	0.60	0.87
Agathobacter	-0.10	0.47	0.64	-0.13	0.30	0.67	-0.21	0.11	0.75	-0.21	0.11	0.74	-0.18	0.17	0.73
Akkermansia	0.21	0.00	0.05	0.14	0.03	0.36	0.14	0.06	0.60	0.14	0.06	0.60	0.14	0.06	0.55
Alistipes	0.18	0.12	0.31	0.11	0.31	0.67	0.04	0.74	0.89	0.04	0.74	0.89	0.04	0.72	0.92
Allisonella	-0.21	0.07	0.26	0.01	0.96	0.96	0.05	0.70	0.89	0.05	0.70	0.89	0.04	0.75	0.92
Anaerofilum	0.33	0.04	0.17	0.15	0.29	0.67	0.16	0.31	0.75	0.16	0.29	0.74	0.17	0.27	0.77
Anaerostipes	0.04	0.77	0.85	-0.04	0.77	0.86	-0.01	0.93	0.96	-0.02	0.90	0.94	0.00	0.98	1.00
Anaerotruncus	0.28	0.04	0.17	0.21	0.07	0.45	0.24	0.07	0.65	0.25	0.06	0.61	0.29	0.03	0.49
Atopobium	-0.07	0.75	0.85	0.03	0.90	0.92	-0.09	0.70	0.89	-0.10	0.68	0.89	-0.03	0.89	0.97
Bacteroides	0.19	0.35	0.54	0.12	0.49	0.76	0.12	0.54	0.89	0.11	0.55	0.89	0.10	0.59	0.87
Barnesiella	0.12	0.09	0.28	0.13	0.03	0.36	0.14	0.05	0.60	0.14	0.05	0.60	0.14	0.04	0.49
Bifidobacterium	0.06	0.49	0.67	0.11	0.18	0.58	0.12	0.18	0.75	0.12	0.19	0.74	0.12	0.19	0.73
Bilophila	0.16	0.06	0.23	0.08	0.34	0.67	0.04	0.66	0.89	0.04	0.68	0.89	0.04	0.66	0.90
Blautia	0.17	0.55	0.71	0.10	0.70	0.86	0.15	0.60	0.89	0.14	0.61	0.89	0.27	0.38	0.82
Butyricicoccus	0.39	0.05	0.20	0.14	0.44	0.74	0.10	0.61	0.89	0.10	0.62	0.89	0.12	0.53	0.87
Butyricimonas	0.18	0.02	0.10	0.14	0.04	0.37	0.16	0.03	0.51	0.16	0.03	0.49	0.16	0.03	0.49
CAG.56	-0.05	0.56	0.71	-0.06	0.44	0.74	-0.08	0.34	0.75	-0.08	0.33	0.74	-0.06	0.46	0.86
Candidatus_Soleaferrea	0.29	0.10	0.29	0.24	0.14	0.58	0.25	0.15	0.75	0.25	0.15	0.74	0.27	0.12	0.66
Caproiciproducens	0.11	0.46	0.63	0.06	0.65	0.84	0.00	0.98	0.98	0.00	0.99	0.99	0.03	0.86	0.97
Catenibacterium	-0.07	0.37	0.55	-0.05	0.54	0.77	-0.03	0.72	0.89	-0.03	0.71	0.89	-0.03	0.72	0.92
Christensenellaceae_R.7_group	0.23	0.00	0.05	0.14	0.04	0.37	0.10	0.20	0.75	0.10	0.19	0.74	0.13	0.10	0.66
Clostridium_sensu_stricto_1	-0.08	0.41	0.59	-0.04	0.64	0.84	-0.07	0.51	0.89	-0.07	0.50	0.89	-0.06	0.52	0.87
Colidextribacter	-0.10	0.57	0.72	-0.13	0.39	0.70	-0.07	0.69	0.89	-0.07	0.69	0.89	0.00	0.99	1.00
Collinsella	-0.13	0.10	0.29	-0.03	0.73	0.86	-0.04	0.58	0.89	-0.04	0.58	0.89	-0.05	0.52	0.87
Coprobacter	0.13	0.20	0.43	0.08	0.36	0.67	0.05	0.64	0.89	0.05	0.62	0.89	0.04	0.72	0.92
Coprococcus	0.01	0.89	0.92	0.05	0.57	0.79	0.07	0.44	0.86	0.07	0.45	0.88	0.09	0.36	0.81
Defluviitaleaceae_UCG.011	0.42	0.00	0.05	0.35	0.01	0.23	0.33	0.02	0.51	0.33	0.02	0.49	0.35	0.02	0.49
Desulfovibrio	0.03	0.72	0.84	0.03	0.65	0.84	0.04	0.57	0.89	0.05	0.54	0.89	0.05	0.55	0.87
Dialister	-0.10	0.11	0.29	-0.08	0.18	0.58	-0.07	0.29	0.75	-0.07	0.29	0.74	-0.05	0.42	0.85
DNF00809	-0.03	0.88	0.92	-0.04	0.76	0.86	0.04	0.78	0.90	0.05	0.77	0.89	0.05	0.76	0.92
Dorea	0.03	0.75	0.85	0.04	0.71	0.86	0.06	0.58	0.89	0.06	0.59	0.89	0.05	0.63	0.87
DTU089	0.15	0.23	0.44	0.06	0.59	0.80	0.02	0.85	0.91	0.03	0.84	0.90	0.06	0.63	0.87
Eggerthella	-0.08	0.51	0.68	-0.15	0.18	0.58	-0.08	0.52	0.89	-0.08	0.52	0.89	-0.06	0.60	0.87
Eisenbergiella	-0.01	0.95	0.98	-0.03	0.79	0.87	0.00	0.98	0.98	0.01	0.96	0.98	0.01	0.96	1.00
Enterorhabdus	0.19	0.15	0.35	0.15	0.22	0.62	0.19	0.16	0.75	0.19	0.16	0.74	0.22	0.12	0.66
Erysipelatoclostridium	0.15	0.28	0.47	0.11	0.36	0.67	0.15	0.25	0.75	0.15	0.25	0.74	0.15	0.26	0.77
Erysipelotrichaceae_UCG.003	0.10	0.29	0.48	0.10	0.23	0.63	0.03	0.74	0.89	0.03	0.73	0.89	0.04	0.63	0.87
Escherichia.Shigella	-0.05	0.55	0.71	0.05	0.57	0.79	0.03	0.73	0.89	0.03	0.74	0.89	0.01	0.89	0.97
Faecalibacterium	0.27	0.07	0.26	0.13	0.37	0.67	0.08	0.60	0.89	0.07	0.61	0.89	0.11	0.46	0.86
Family_XIII_AD3011_group	0.30	0.01	0.09	0.16	0.14	0.58	0.15	0.21	0.75	0.16	0.19	0.74	0.17	0.16	0.73
Family_XIII_UCG.001	0.24	0.11	0.29	0.20	0.14	0.58	0.17	0.25	0.75	0.18	0.23	0.74	0.18	0.24	0.77
FD2005	-0.22	0.10	0.29	-0.08	0.52	0.77	-0.11	0.38	0.78	-0.11	0.39	0.78	-0.10	0.46	0.86
Flavonifractor	-0.04	0.72	0.84	-0.07	0.52	0.77	-0.01	0.91	0.96	-0.01	0.91	0.94	0.02	0.90	0.97
Frisingicoccus	0.19	0.07	0.25	0.14	0.15	0.58	0.10	0.32	0.75	0.11	0.30	0.74	0.12	0.25	0.77
Fusicatenibacter	0.00	0.96	0.98	-0.04	0.64	0.84	-0.10	0.31	0.75	-0.10	0.29	0.74	-0.10	0.29	0.77
Fusobacterium	-0.15	0.24	0.45	-0.04	0.73	0.86	-0.03	0.81	0.90	-0.03	0.82	0.90	-0.04	0.74	0.92
GCA.900066575	0.33	0.01	0.09	0.21	0.08	0.49	0.14	0.30	0.75	0.15	0.29	0.74	0.18	0.19	0.73
Gordonibacter	0.30	0.04	0.18	0.15	0.25	0.63	0.17	0.23	0.75	0.18	0.21	0.74	0.17	0.23	0.77
Granulicatella	-0.12	0.44	0.62	-0.03	0.81	0.87	-0.04	0.80	0.90	-0.04	0.79	0.89	0.00	1.00	1.00
Haemophilus	0.11	0.22	0.44	0.08	0.36	0.67	0.02	0.81	0.90	0.02	0.82	0.90	0.05	0.60	0.87
Harryflintia	0.04	0.77	0.85	0.02	0.90	0.92	-0.03	0.83	0.91	-0.03	0.85	0.90	0.00	0.99	1.00

© 2022 Meyer K et al. *JAMA Network Open.*

Holdemanella	-0.03	0.71	0.84	0.01	0.91	0.92	0.09	0.35	0.75	0.09	0.35	0.74	0.08	0.43	0.85
Holdemania	0.10	0.70	0.84	0.09	0.69	0.86	0.12	0.64	0.89	0.12	0.63	0.89	0.11	0.68	0.91
Howardella	-0.13	0.31	0.50	-0.15	0.18	0.58	-0.13	0.29	0.75	-0.13	0.31	0.74	-0.11	0.37	0.81
Hungatella	-0.15	0.21	0.44	-0.15	0.17	0.58	-0.12	0.33	0.75	-0.12	0.33	0.74	-0.11	0.36	0.81
Hydrogenoanaerobacterium	0.37	0.01	0.06	0.19	0.13	0.58	0.17	0.23	0.75	0.18	0.21	0.74	0.19	0.18	0.73
Incertae_Sedis	0.16	0.38	0.57	0.11	0.50	0.77	0.11	0.54	0.89	0.11	0.54	0.89	0.13	0.50	0.87
Intestinibacter	0.04	0.75	0.85	0.05	0.66	0.84	0.04	0.74	0.89	0.04	0.78	0.89	0.04	0.74	0.92
Intestinimonas	0.32	0.00	0.05	0.20	0.05	0.38	0.17	0.13	0.75	0.17	0.12	0.74	0.19	0.10	0.66
Lachnoclostridium	-0.57	0.01	0.09	-0.35	0.10	0.53	-0.21	0.36	0.76	-0.21	0.35	0.74	-0.24	0.29	0.77
Lachnospira	0.20	0.05	0.20	0.09	0.34	0.67	-0.04	0.67	0.89	-0.05	0.64	0.89	-0.02	0.87	0.97
Lachnospiraceae_FCS020_group	0.44	0.00	0.04	0.31	0.01	0.22	0.29	0.02	0.51	0.29	0.02	0.49	0.31	0.01	0.49
Lachnospiraceae_ND3007_group	0.09	0.27	0.46	0.06	0.46	0.74	0.03	0.66	0.89	0.03	0.68	0.89	0.06	0.47	0.86
Lachnospiraceae_NK4A136_group	0.13	0.22	0.44	-0.01	0.91	0.92	-0.13	0.26	0.75	-0.13	0.26	0.74	-0.09	0.43	0.85
Lachnospiraceae_UCG.001	0.14	0.11	0.29	0.08	0.30	0.67	0.01	0.88	0.93	0.02	0.85	0.90	0.04	0.63	0.87
Lachnospiraceae_UCG.004	0.14	0.14	0.33	0.10	0.24	0.63	0.09	0.35	0.75	0.09	0.35	0.74	0.10	0.32	0.77
Lachnospiraceae_UCG.010	-0.03	0.80	0.86	-0.11	0.33	0.67	-0.14	0.27	0.75	-0.14	0.28	0.74	-0.13	0.32	0.77
Lactobacillus	-0.16	0.08	0.28	-0.08	0.33	0.67	-0.11	0.25	0.75	-0.12	0.23	0.74	-0.12	0.23	0.77
Lactococcus	0.41	0.01	0.09	0.28	0.06	0.44	0.27	0.10	0.75	0.27	0.11	0.74	0.23	0.16	0.73
Marvinbryantia	0.07	0.44	0.62	0.02	0.83	0.88	0.02	0.84	0.91	0.02	0.85	0.90	0.05	0.59	0.87
Megasphaera	-0.10	0.13	0.32	-0.02	0.80	0.87	0.02	0.76	0.90	0.02	0.76	0.89	0.01	0.94	0.99
Monoglobus	0.24	0.02	0.11	0.16	0.10	0.53	0.05	0.62	0.89	0.05	0.61	0.89	0.07	0.53	0.87
Moryella	0.15	0.36	0.55	0.04	0.79	0.87	-0.06	0.72	0.89	-0.06	0.71	0.89	-0.05	0.77	0.92
Negativibacillus	0.03	0.75	0.85	0.07	0.41	0.71	0.09	0.36	0.76	0.09	0.35	0.74	0.10	0.28	0.77
NK4A214_group	0.13	0.09	0.28	0.07	0.31	0.67	0.02	0.78	0.90	0.03	0.74	0.89	0.05	0.54	0.87
Odoribacter	0.22	0.00	0.05	0.14	0.04	0.36	0.11	0.12	0.75	0.11	0.12	0.74	0.12	0.09	0.66
Oscillibacter	0.19	0.14	0.33	0.17	0.15	0.58	0.24	0.08	0.65	0.25	0.07	0.61	0.28	0.05	0.49
Oscillospira	0.13	0.44	0.62	0.09	0.53	0.77	0.10	0.52	0.89	0.10	0.52	0.89	0.17	0.29	0.77
Oxalobacter	0.18	0.17	0.38	0.04	0.76	0.86	0.01	0.96	0.98	0.01	0.95	0.98	-0.03	0.84	0.95
Paludicola	0.34	0.01	0.09	0.24	0.06	0.43	0.19	0.17	0.75	0.20	0.15	0.74	0.21	0.14	0.71
Parabacteroides	0.11	0.23	0.44	0.11	0.20	0.61	0.09	0.32	0.75	0.09	0.33	0.74	0.10	0.26	0.77
Paraprevotella	-0.08	0.26	0.46	-0.06	0.32	0.67	-0.08	0.26	0.75	-0.08	0.27	0.74	-0.06	0.37	0.81
Parasutterella	0.21	0.00	0.05	0.19	0.00	0.19	0.16	0.03	0.51	0.16	0.03	0.49	0.15	0.04	0.49
Phascolarctobacterium	0.18	0.01	0.06	0.17	0.00	0.19	0.17	0.01	0.51	0.18	0.01	0.49	0.16	0.01	0.49
Phocea	-0.20	0.23	0.44	-0.26	0.07	0.45	-0.28	0.07	0.65	-0.28	0.07	0.61	-0.26	0.11	0.66
Prevotella	-0.06	0.27	0.46	-0.05	0.39	0.70	-0.04	0.51	0.89	-0.04	0.52	0.89	-0.03	0.58	0.87
Romboutsia	-0.12	0.28	0.47	-0.06	0.53	0.77	-0.05	0.67	0.89	-0.06	0.63	0.89	-0.03	0.81	0.93
Roseburia	0.10	0.36	0.55	0.07	0.51	0.77	-0.08	0.50	0.89	-0.08	0.49	0.89	-0.04	0.73	0.92
Rothia	-0.42	0.05	0.21	-0.42	0.03	0.36	-0.55	0.01	0.51	-0.56	0.01	0.49	-0.49	0.03	0.49
Ruminococcus	0.18	0.02	0.10	0.10	0.16	0.58	0.06	0.44	0.86	0.06	0.43	0.84	0.08	0.33	0.77
Sellimonas	0.04	0.70	0.84	0.01	0.90	0.92	0.00	0.98	0.98	0.00	0.99	0.99	0.01	0.91	0.98
Senegalimassilia	-0.06	0.54	0.71	-0.03	0.77	0.86	-0.04	0.69	0.89	-0.04	0.70	0.89	-0.02	0.81	0.93
Slackia	-0.02	0.79	0.86	-0.03	0.70	0.86	-0.02	0.78	0.90	-0.02	0.78	0.89	-0.03	0.74	0.92
Streptococcus	0.16	0.23	0.44	0.14	0.24	0.63	0.14	0.30	0.75	0.14	0.31	0.74	0.15	0.28	0.77
Subdoligranulum	0.29	0.01	0.06	0.23	0.02	0.33	0.22	0.03	0.51	0.22	0.03	0.49	0.26	0.01	0.49
Sutterella	-0.18	0.00	0.05	-0.13	0.03	0.36	-0.12	0.05	0.60	-0.12	0.05	0.60	-0.12	0.06	0.55
TM7x	-0.14	0.32	0.51	-0.16	0.21	0.61	-0.08	0.56	0.89	-0.08	0.56	0.89	-0.07	0.63	0.87
Turicibacter	0.00	0.98	0.99	0.10	0.47	0.75	0.18	0.29	0.75	0.17	0.30	0.74	0.17	0.31	0.77
Tuzzerella	0.13	0.25	0.45	0.12	0.23	0.63	0.10	0.37	0.77	0.10	0.37	0.75	0.10	0.40	0.84
UBA1819	0.16	0.28	0.47	0.10	0.45	0.74	0.16	0.27	0.75	0.17	0.25	0.74	0.19	0.20	0.73
UCG.002	0.18	0.01	0.09	0.10	0.12	0.58	0.07	0.33	0.75	0.07	0.31	0.74	0.10	0.17	0.73
UCG.003	0.12	0.14	0.33	0.08	0.28	0.67	0.05	0.57	0.89	0.05	0.56	0.89	0.05	0.53	0.87
UCG.005	0.28	0.00	0.05	0.17	0.03	0.36	0.12	0.18	0.75	0.13	0.16	0.74	0.16	0.09	0.66
UCG.009	0.08	0.61	0.76	-0.07	0.63	0.84	-0.17	0.23	0.75	-0.18	0.23	0.74	-0.15	0.31	0.77
Veillonella	0.00	1.00	1.00	0.03	0.75	0.86	-0.02	0.85	0.91	-0.02	0.83	0.90	0.00	0.97	1.00
Victivallis	-0.03	0.80	0.86	-0.08	0.41	0.71	-0.16	0.14	0.75	-0.16	0.14	0.74	-0.17	0.12	0.66

Multivariable-adjusted regression models adjusted for:
Model 1: sequencing batch, age, race, gender, study center
Model 2: Model 1, with education

© 2022 Meyer K et al. *JAMA Network Open.*

Model 3: Model 2, with physical activity, diet score, medication use, and smoking
Model 4: Model 3 with BMI
Model 5: Model 4 with hypertension and diabetes

© 2022 Meyer K et al. *JAMA Network Open.*

eTable 33: Multivariable-adjusted regression coefficients, p-values, and false discovery rate (FDR)-adjusted p-values for genera among White participants with respect to DSST.

	Model1			Model2			Model3			Model4			Model5		
	beta	p-value	FDR	beta	p-value	FDR	beta	p-value	FDR	beta	p-value	FDR	beta	p-value	FDR
Acetanaerobacterium	-0.41	0.82	0.94	0.61	0.73	0.91	0.77	0.67	0.97	0.58	0.75	1.00	0.23	0.90	0.98
Acidaminococcus	-1.24	0.05	0.34	-0.75	0.23	0.77	-0.64	0.33	0.97	-0.61	0.36	0.94	-0.60	0.37	0.95
Actinomyces	2.00	0.26	0.61	2.04	0.23	0.77	2.86	0.11	0.83	2.82	0.11	0.80	3.02	0.09	0.78
Adlercreutzia	0.72	0.44	0.74	0.85	0.35	0.86	0.45	0.64	0.97	0.41	0.67	0.99	0.39	0.69	0.98
Agathobacter	0.19	0.87	0.95	0.16	0.89	0.96	-0.53	0.66	0.97	-0.27	0.82	1.00	-0.17	0.89	0.98
Akkermansia	1.14	0.06	0.34	0.99	0.09	0.53	0.96	0.12	0.83	0.94	0.12	0.80	0.94	0.13	0.78
Alistipes	0.56	0.57	0.77	0.43	0.65	0.91	0.05	0.96	0.99	0.00	1.00	1.00	-0.08	0.93	0.98
Anaerofilum	1.01	0.44	0.74	0.23	0.86	0.96	0.06	0.96	0.99	-0.04	0.98	1.00	-0.01	1.00	1.00
Anaerostipes	-0.06	0.97	0.99	-0.34	0.83	0.96	-1.02	0.54	0.97	-1.33	0.43	0.94	-1.24	0.46	0.97
Anaerotruncus	-0.63	0.62	0.79	-0.87	0.48	0.88	-0.82	0.53	0.97	-1.21	0.36	0.94	-1.25	0.35	0.95
Angelakisella	-2.18	0.24	0.61	-2.99	0.09	0.53	-3.03	0.11	0.83	-2.97	0.12	0.80	-3.21	0.09	0.78
Bacteroides	-4.35	0.13	0.51	-4.49	0.10	0.53	-3.77	0.20	0.93	-3.74	0.20	0.90	-3.86	0.19	0.89
Barnesiella	1.20	0.03	0.23	1.14	0.03	0.35	0.90	0.11	0.83	0.90	0.11	0.80	0.91	0.11	0.78
Bifidobacterium	-0.40	0.58	0.77	0.15	0.84	0.96	0.09	0.90	0.99	0.05	0.95	1.00	0.04	0.95	0.98
Bilophila	-0.04	0.96	0.99	0.01	0.99	0.99	-0.01	0.99	0.99	-0.09	0.91	1.00	-0.12	0.88	0.98
Blautia	3.27	0.25	0.61	2.29	0.41	0.88	2.23	0.44	0.97	1.62	0.57	0.94	1.88	0.51	0.97
Butyricicoccus	1.03	0.60	0.78	0.20	0.91	0.96	-0.30	0.88	0.99	-0.32	0.87	1.00	-0.13	0.95	0.98
Butyricimonas	0.62	0.34	0.66	0.42	0.50	0.88	0.35	0.60	0.97	0.39	0.55	0.94	0.38	0.57	0.97
CAG.56	2.27	0.00	0.08	1.87	0.01	0.25	1.57	0.05	0.78	1.52	0.06	0.80	1.55	0.05	0.78
Candidatus_Soleaferrea	-1.87	0.23	0.61	-2.39	0.11	0.53	-1.94	0.21	0.93	-2.01	0.20	0.90	-2.23	0.16	0.84
Caproiciproducens	-0.13	0.92	0.97	0.13	0.92	0.96	-0.48	0.72	0.97	-0.57	0.67	0.99	-0.60	0.65	0.97
Christensenellaceae_R.7_group	1.41	0.03	0.23	1.24	0.05	0.38	0.89	0.19	0.93	0.79	0.24	0.94	0.81	0.23	0.95
Clostridium_sensu_stricto_1	-0.08	0.92	0.97	-0.03	0.97	0.99	-0.10	0.89	0.99	-0.24	0.76	1.00	-0.37	0.64	0.97
Colidextribacter	-2.90	0.10	0.46	-3.81	0.03	0.35	-3.61	0.06	0.78	-3.82	0.04	0.80	-3.78	0.05	0.78
Collinsella	-0.25	0.74	0.88	0.19	0.79	0.93	0.20	0.79	0.97	0.32	0.67	0.99	0.30	0.70	0.98
Coprobacter	-0.19	0.80	0.93	-0.50	0.48	0.88	-0.78	0.31	0.97	-0.81	0.28	0.94	-0.80	0.29	0.95
Coprococcus	2.01	0.02	0.21	1.72	0.04	0.36	1.49	0.09	0.83	1.43	0.10	0.80	1.40	0.11	0.78
Defluviitaleaceae_UCG.011	1.85	0.12	0.51	1.58	0.17	0.66	1.02	0.40	0.97	0.84	0.49	0.94	0.78	0.52	0.97
Desulfovibrio	0.44	0.56	0.77	0.47	0.51	0.88	0.40	0.60	0.97	0.31	0.68	1.00	0.35	0.65	0.97
Dialister	0.31	0.57	0.77	0.27	0.62	0.91	0.21	0.71	0.97	0.30	0.61	0.96	0.26	0.66	0.97
DNF00809	2.36	0.08	0.42	1.98	0.13	0.62	1.39	0.32	0.97	1.28	0.36	0.94	1.27	0.36	0.95
Dorea	-0.15	0.90	0.97	0.15	0.90	0.96	-0.30	0.81	0.97	-0.30	0.81	1.00	-0.25	0.84	0.98
DTU089	-1.31	0.25	0.61	-1.20	0.27	0.82	-1.61	0.16	0.93	-1.87	0.11	0.80	-1.82	0.12	0.78
Eggerthella	-0.55	0.62	0.79	-0.49	0.65	0.91	-0.22	0.84	0.97	-0.40	0.72	1.00	-0.29	0.80	0.98
Eisenbergiella	-0.53	0.66	0.81	-0.44	0.70	0.91	-0.64	0.59	0.97	-1.01	0.40	0.94	-0.83	0.49	0.97
Erysipelatoclostridium	-1.36	0.25	0.61	-1.05	0.36	0.86	-0.32	0.79	0.97	-0.60	0.62	0.97	-0.62	0.61	0.97
Erysipelotrichaceae_UCG.003	0.53	0.54	0.77	0.36	0.67	0.91	-0.20	0.83	0.97	-0.24	0.79	1.00	-0.26	0.78	0.98
Escherichia.Shigella	-0.26	0.74	0.88	-0.18	0.81	0.95	0.05	0.95	0.99	0.09	0.91	1.00	0.10	0.90	0.98
Faecalibacterium	1.50	0.14	0.51	1.42	0.14	0.64	0.94	0.38	0.97	0.97	0.36	0.94	0.96	0.37	0.95
Family_XIII_AD3011_group	1.83	0.08	0.42	1.39	0.17	0.66	0.97	0.38	0.97	0.77	0.48	0.94	0.78	0.48	0.97
Family_XIII_UCG.001	0.98	0.44	0.74	0.52	0.67	0.91	0.26	0.84	0.97	-0.03	0.98	1.00	-0.08	0.95	0.98
FD2005	1.16	0.38	0.71	0.80	0.53	0.89	0.73	0.59	0.97	0.99	0.46	0.94	0.88	0.52	0.97
Flavonifractor	-2.20	0.05	0.34	-1.94	0.07	0.52	-1.53	0.18	0.93	-1.50	0.19	0.90	-1.49	0.19	0.89
Fournierella	0.59	0.61	0.79	0.65	0.56	0.89	0.27	0.82	0.97	0.16	0.89	1.00	0.19	0.87	0.98
Frisingicoccus	-1.66	0.06	0.34	-1.76	0.04	0.36	-1.92	0.03	0.78	-1.99	0.02	0.80	-1.89	0.03	0.78
Fusicatenibacter	0.00	1.00	1.00	-0.33	0.70	0.91	-0.64	0.48	0.97	-0.52	0.57	0.94	-0.50	0.59	0.97
Fusobacterium	1.09	0.47	0.74	1.16	0.42	0.88	2.00	0.18	0.93	1.83	0.22	0.94	1.70	0.26	0.95
GCA.900066575	0.69	0.55	0.77	0.15	0.89	0.96	-0.39	0.74	0.97	-0.61	0.60	0.96	-0.87	0.46	0.97
GCA.900066755	1.22	0.52	0.77	0.84	0.64	0.91	1.33	0.50	0.97	1.09	0.58	0.94	1.02	0.61	0.97
Gordonibacter	-1.13	0.32	0.65	-1.07	0.33	0.86	-1.45	0.21	0.93	-1.75	0.13	0.80	-1.72	0.14	0.81
Granulicatella	1.45	0.42	0.74	1.03	0.55	0.89	1.54	0.39	0.97	1.39	0.44	0.94	1.55	0.39	0.95
Haemophilus	1.26	0.16	0.53	0.96	0.26	0.82	0.96	0.29	0.97	0.82	0.36	0.94	0.85	0.35	0.95
Harryflintia	-0.27	0.85	0.94	-0.48	0.72	0.91	-0.15	0.91	0.99	-0.23	0.87	1.00	-0.49	0.73	0.98
Holdemanella	1.05	0.27	0.61	1.06	0.25	0.81	0.72	0.46	0.97	0.89	0.36	0.94	0.94	0.33	0.95

© 2022 Meyer K et al. *JAMA Network Open.*

Holdemania	0.39	0.84	0.94	0.25	0.90	0.96	0.96	0.63	0.97	0.68	0.74	1.00	0.33	0.87	0.98
Hungatella	-0.66	0.54	0.77	-0.31	0.77	0.93	0.25	0.82	0.97	0.12	0.91	1.00	0.29	0.80	0.98
Hydrogenoanaerobacterium	1.59	0.15	0.53	1.12	0.30	0.84	0.34	0.77	0.97	0.11	0.92	1.00	0.13	0.92	0.98
Incertae_Sedis	0.87	0.65	0.81	1.44	0.44	0.88	1.37	0.49	0.97	1.15	0.56	0.94	1.19	0.55	0.97
Intestinibacter	0.65	0.51	0.77	1.24	0.20	0.71	1.23	0.23	0.94	1.30	0.20	0.90	1.35	0.19	0.89
Intestinimonas	0.56	0.56	0.77	0.13	0.89	0.96	0.02	0.99	0.99	-0.19	0.85	1.00	-0.07	0.95	0.98
Lachnoclostridium	-4.41	0.03	0.26	-3.54	0.08	0.53	-1.60	0.47	0.97	-1.37	0.53	0.94	-1.29	0.56	0.97
Lachnospira	4.90	0.00	0.00	4.00	0.00	0.03	3.91	0.00	0.07	3.83	0.00	0.08	3.83	0.00	0.08
Lachnospiraceae_FCS020_group	3.36	0.00	0.06	2.69	0.01	0.25	2.13	0.05	0.78	2.06	0.06	0.80	2.07	0.06	0.78
Lachnospiraceae_ND3007_group	0.16	0.82	0.94	-0.40	0.56	0.89	-0.80	0.27	0.97	-0.79	0.28	0.94	-0.82	0.27	0.95
Lachnospiraceae_NK4A136_group	1.07	0.33	0.65	0.54	0.61	0.91	-0.28	0.80	0.97	-0.32	0.78	1.00	-0.30	0.79	0.98
Lachnospiraceae_UCG.001	1.76	0.02	0.21	1.62	0.03	0.35	1.45	0.06	0.78	1.45	0.06	0.80	1.45	0.06	0.78
Lachnospiraceae_UCG.004	1.60	0.06	0.34	1.32	0.11	0.53	1.09	0.20	0.93	1.15	0.18	0.90	1.01	0.25	0.95
Lachnospiraceae_UCG.010	1.40	0.26	0.61	0.85	0.48	0.88	0.81	0.52	0.97	0.76	0.55	0.94	0.78	0.54	0.97
Lactobacillus	-3.23	0.01	0.17	-2.36	0.05	0.42	-2.25	0.08	0.82	-2.13	0.09	0.80	-1.96	0.13	0.78
Lactococcus	-1.26	0.31	0.64	-1.17	0.33	0.86	-1.19	0.35	0.97	-1.43	0.26	0.94	-1.48	0.25	0.95
Marvinbryantia	1.00	0.21	0.61	0.69	0.37	0.86	0.36	0.66	0.97	0.24	0.77	1.00	0.27	0.74	0.98
Megasphaera	-0.04	0.97	0.99	0.33	0.70	0.91	0.60	0.52	0.97	0.74	0.43	0.94	0.71	0.45	0.97
Monoglobus	1.68	0.15	0.53	1.61	0.15	0.65	1.24	0.30	0.97	1.06	0.37	0.94	1.16	0.33	0.95
Moryella	3.46	0.01	0.17	2.91	0.02	0.35	2.06	0.13	0.89	2.09	0.13	0.80	2.09	0.13	0.78
Negativibacillus	-2.07	0.02	0.21	-2.13	0.01	0.25	-2.14	0.02	0.78	-2.03	0.02	0.80	-2.08	0.02	0.78
NK4A214_group	1.15	0.09	0.42	0.87	0.18	0.66	0.47	0.50	0.97	0.46	0.51	0.94	0.49	0.49	0.97
Odoribacter	-0.20	0.76	0.90	-0.09	0.89	0.96	-0.50	0.47	0.97	-0.56	0.42	0.94	-0.62	0.38	0.95
Oscillibacter	1.21	0.29	0.62	0.74	0.50	0.88	0.35	0.76	0.97	0.25	0.83	1.00	0.19	0.87	0.98
Oscillospira	-2.22	0.20	0.61	-1.75	0.30	0.84	-1.06	0.55	0.97	-1.19	0.50	0.94	-1.19	0.50	0.97
Oxalobacter	2.63	0.02	0.21	1.77	0.10	0.53	1.58	0.17	0.93	1.63	0.15	0.88	1.76	0.13	0.78
Paludicola	1.30	0.25	0.61	0.38	0.73	0.91	-0.34	0.77	0.97	-0.71	0.55	0.94	-0.50	0.68	0.98
Parabacteroides	-0.62	0.44	0.74	-0.05	0.94	0.97	0.29	0.72	0.97	0.24	0.77	1.00	0.24	0.78	0.98
Paraprevotella	-0.57	0.35	0.66	-0.32	0.58	0.91	-0.26	0.68	0.97	-0.29	0.64	0.99	-0.29	0.65	0.97
Parasutterella	0.35	0.57	0.77	0.31	0.61	0.91	-0.05	0.93	0.99	-0.02	0.97	1.00	-0.10	0.88	0.98
Peptococcus	0.86	0.46	0.74	0.45	0.69	0.91	0.27	0.82	0.97	0.15	0.90	1.00	0.30	0.80	0.98
Phascolarctobacterium	-0.65	0.27	0.61	-0.53	0.35	0.86	-0.52	0.38	0.97	-0.65	0.28	0.94	-0.64	0.29	0.95
Phocea	-0.59	0.70	0.85	-0.59	0.68	0.91	-0.37	0.81	0.97	-0.19	0.90	1.00	-0.33	0.83	0.98
Prevotella	-0.87	0.13	0.51	-0.50	0.37	0.86	-0.48	0.42	0.97	-0.37	0.53	0.94	-0.36	0.55	0.97
Romboutsia	0.09	0.93	0.97	0.24	0.78	0.93	-0.21	0.82	0.97	-0.34	0.71	1.00	-0.44	0.65	0.97
Roseburia	1.15	0.34	0.66	0.87	0.45	0.88	0.72	0.55	0.97	0.81	0.50	0.94	0.80	0.51	0.97
Ruminococcus	0.81	0.28	0.62	0.48	0.51	0.88	0.03	0.97	0.99	0.00	1.00	1.00	0.04	0.96	0.98
Sellimonas	1.71	0.10	0.45	1.38	0.16	0.66	0.97	0.36	0.97	0.94	0.37	0.94	0.97	0.36	0.95
Shuttleworthia	0.18	0.85	0.94	-0.57	0.55	0.89	-1.11	0.28	0.97	-1.14	0.26	0.94	-1.04	0.31	0.95
Slackia	-0.97	0.29	0.62	-0.61	0.49	0.88	-0.83	0.37	0.97	-0.81	0.39	0.94	-0.91	0.33	0.95
Streptococcus	-0.44	0.73	0.88	-0.41	0.74	0.91	-0.49	0.72	0.97	-0.94	0.49	0.94	-0.83	0.54	0.97
Subdoligranulum	0.66	0.47	0.74	0.30	0.74	0.91	-0.27	0.77	0.97	-0.29	0.75	1.00	-0.35	0.70	0.98
Sutterella	-0.66	0.22	0.61	-0.37	0.49	0.88	-0.38	0.49	0.97	-0.41	0.45	0.94	-0.44	0.43	0.97
TM7x	0.86	0.50	0.77	0.12	0.92	0.96	0.02	0.99	0.99	-0.16	0.90	1.00	-0.05	0.97	0.98
Turicibacter	0.75	0.54	0.77	1.10	0.35	0.86	0.92	0.45	0.97	0.79	0.51	0.94	0.58	0.63	0.97
Tuzzerella	-2.53	0.00	0.08	-2.22	0.01	0.25	-1.89	0.03	0.78	-1.88	0.03	0.80	-1.98	0.02	0.78
Tyzzerella	0.03	0.98	0.99	0.02	0.99	0.99	-0.08	0.95	0.99	0.11	0.93	1.00	0.41	0.74	0.98
UBA1819	-1.10	0.46	0.74	-1.07	0.45	0.88	-0.92	0.54	0.97	-1.23	0.41	0.94	-1.28	0.39	0.95
UCG.002	0.51	0.44	0.74	0.23	0.72	0.91	-0.46	0.51	0.97	-0.55	0.44	0.94	-0.62	0.39	0.95
UCG.003	1.09	0.14	0.51	0.52	0.47	0.88	0.01	0.99	0.99	0.00	1.00	1.00	-0.03	0.97	0.98
UCG.005	1.06	0.22	0.61	0.92	0.26	0.82	0.18	0.84	0.97	0.03	0.97	1.00	0.06	0.95	0.98
UCG.009	1.69	0.17	0.57	0.97	0.42	0.88	0.52	0.68	0.97	0.28	0.83	1.00	0.27	0.83	0.98
Veillonella	1.15	0.23	0.61	0.94	0.30	0.84	1.01	0.29	0.97	0.94	0.32	0.94	0.97	0.31	0.95
Victivallis	2.26	0.01	0.17	1.83	0.03	0.36	1.69	0.07	0.78	1.56	0.09	0.80	1.67	0.07	0.78

Multivariable-adjusted regression models adjusted for:
Model 1: sequencing batch, age, race, gender, study center
Model 2: Model 1, with education
Model 3: Model 2, with physical activity, diet score, medication use, and smoking

© 2022 Meyer K et al. *JAMA Network Open.*

Model 4: Model 3 with BMI
Model 5: Model 4 with hypertension and diabetes

© 2022 Meyer K et al. *JAMA Network Open.*

eTable 34: Multivariable-adjusted regression coefficients, p-values, and false discovery rate (FDR)-adjusted p-values for genera among White participants with respect to Stroop.

	Model1			Model2			Model3			Model4			Model5		
	beta	p-value	FDR	beta	p-value	FDR	beta	p-value	FDR	beta	p-value	FDR	beta	p-value	FDR
Acetanaerobacterium	0.26	0.83	0.97	0.09	0.94	0.99	0.35	0.78	0.95	0.39	0.76	0.95	0.19	0.88	0.97
Acidaminococcus	0.58	0.17	0.67	0.50	0.24	0.75	0.66	0.14	0.64	0.66	0.15	0.66	0.64	0.16	0.74
Actinomyces	-0.60	0.60	0.94	-0.60	0.61	0.93	-1.20	0.33	0.85	-1.19	0.33	0.87	-0.99	0.42	0.88
Adlercreutzia	0.22	0.73	0.96	0.19	0.76	0.97	0.45	0.50	0.88	0.45	0.50	0.89	0.37	0.58	0.88
Agathobacter	-0.05	0.94	0.99	-0.04	0.95	0.99	0.37	0.65	0.92	0.33	0.69	0.94	0.47	0.57	0.88
Akkermansia	0.01	0.97	0.99	0.04	0.93	0.99	0.06	0.88	0.97	0.07	0.88	0.97	0.04	0.93	0.97
Alistipes	-0.45	0.48	0.85	-0.43	0.50	0.89	-0.12	0.86	0.97	-0.11	0.87	0.97	-0.30	0.67	0.91
Anaerofilum	1.02	0.24	0.70	1.17	0.18	0.72	1.36	0.13	0.64	1.38	0.13	0.66	1.47	0.11	0.73
Anaerostipes	-0.24	0.83	0.97	-0.17	0.88	0.98	-0.41	0.72	0.95	-0.36	0.76	0.95	-0.30	0.80	0.97
Anaerotruncus	1.17	0.17	0.67	1.21	0.15	0.70	1.53	0.09	0.64	1.63	0.07	0.66	1.46	0.11	0.73
Angelakisella	0.58	0.63	0.94	0.73	0.55	0.91	1.04	0.42	0.88	1.03	0.43	0.89	1.17	0.37	0.88
Bacteroides	2.43	0.20	0.67	2.45	0.19	0.72	2.47	0.22	0.80	2.46	0.22	0.80	2.45	0.22	0.74
Barnesiella	-0.91	0.01	0.33	-0.90	0.01	0.37	-0.93	0.01	0.53	-0.93	0.01	0.54	-0.89	0.02	0.52
Bifidobacterium	-0.60	0.21	0.67	-0.71	0.14	0.70	-0.71	0.15	0.64	-0.71	0.16	0.66	-0.67	0.18	0.74
Bilophila	-0.56	0.28	0.77	-0.57	0.26	0.77	-0.38	0.48	0.88	-0.37	0.49	0.89	-0.35	0.52	0.88
Blautia	2.28	0.22	0.67	2.48	0.19	0.72	2.24	0.25	0.83	2.39	0.22	0.80	2.32	0.24	0.74
Butyricicoccus	2.19	0.09	0.67	2.38	0.06	0.70	3.10	0.02	0.62	3.10	0.02	0.57	3.10	0.02	0.52
Butyricimonas	-0.31	0.47	0.84	-0.28	0.51	0.89	-0.13	0.77	0.95	-0.14	0.76	0.95	-0.20	0.67	0.91
CAG.56	-0.92	0.07	0.67	-0.86	0.09	0.70	-0.66	0.23	0.80	-0.65	0.23	0.80	-0.69	0.21	0.74
Candidatus_Soleaferrea	1.46	0.15	0.67	1.54	0.13	0.70	1.53	0.15	0.64	1.55	0.15	0.66	1.42	0.19	0.74
Caproiciproducens	-0.79	0.35	0.82	-0.83	0.32	0.83	-0.70	0.43	0.88	-0.69	0.44	0.89	-0.77	0.39	0.88
Christensenellaceae_R.7_group	-0.95	0.02	0.38	-0.92	0.03	0.45	-0.78	0.09	0.64	-0.77	0.10	0.66	-0.81	0.08	0.73
Clostridium_sensu_stricto_1	-0.18	0.73	0.96	-0.19	0.71	0.97	-0.26	0.63	0.92	-0.24	0.66	0.94	-0.13	0.82	0.97
Colidextribacter	0.90	0.44	0.84	1.08	0.36	0.83	1.35	0.30	0.85	1.39	0.29	0.83	1.69	0.20	0.74
Collinsella	-0.01	0.99	0.99	-0.08	0.87	0.98	-0.19	0.71	0.95	-0.21	0.68	0.94	-0.22	0.67	0.91
Coprobacter	0.59	0.22	0.67	0.64	0.18	0.72	0.81	0.12	0.64	0.81	0.11	0.66	0.80	0.12	0.74
Coprococcus	-0.33	0.57	0.94	-0.29	0.62	0.94	-0.14	0.81	0.96	-0.13	0.83	0.96	-0.07	0.91	0.97
Defluviitaleaceae_UCG.011	-0.97	0.22	0.67	-0.92	0.24	0.75	-0.88	0.29	0.85	-0.86	0.30	0.86	-0.99	0.24	0.74
Desulfovibrio	-0.26	0.60	0.94	-0.26	0.60	0.93	-0.04	0.93	0.97	-0.03	0.96	0.98	-0.04	0.93	0.97
Dialister	-0.01	0.98	0.99	0.00	0.99	0.99	0.03	0.95	0.97	0.01	0.98	0.98	0.09	0.82	0.97
DNF00809	-0.70	0.44	0.84	-0.64	0.48	0.89	-0.51	0.60	0.92	-0.49	0.61	0.92	-0.43	0.65	0.91
Dorea	-0.12	0.88	0.97	-0.16	0.84	0.98	0.04	0.96	0.97	0.04	0.96	0.98	-0.06	0.95	0.97
DTU089	0.63	0.40	0.84	0.61	0.41	0.88	0.68	0.39	0.85	0.73	0.36	0.87	0.69	0.38	0.88
Eggerthella	0.89	0.22	0.67	0.88	0.22	0.75	0.86	0.26	0.83	0.90	0.24	0.80	0.95	0.21	0.74
Eisenbergiella	-0.77	0.32	0.77	-0.78	0.31	0.83	-0.97	0.23	0.80	-0.93	0.25	0.80	-0.82	0.31	0.87
Erysipelatoclostridium	0.05	0.95	0.99	-0.01	0.99	0.99	-0.36	0.66	0.92	-0.31	0.70	0.94	-0.14	0.87	0.97
Erysipelotrichaceae_UCG.003	-0.82	0.15	0.67	-0.79	0.17	0.72	-0.69	0.26	0.83	-0.68	0.27	0.80	-0.68	0.27	0.78
Escherichia.Shigella	0.38	0.45	0.84	0.37	0.46	0.89	0.17	0.76	0.95	0.16	0.77	0.95	0.11	0.84	0.97
Faecalibacterium	-2.21	0.00	0.05	-2.19	0.00	0.09	-2.06	0.00	0.23	-2.07	0.00	0.23	-2.03	0.01	0.28
Family_XIII_AD3011_group	-0.09	0.90	0.97	-0.01	0.98	0.99	0.52	0.49	0.88	0.56	0.46	0.89	0.48	0.53	0.88
Family_XIII_UCG.001	-0.15	0.86	0.97	-0.06	0.94	0.99	-0.02	0.98	0.98	0.03	0.97	0.98	-0.05	0.95	0.97
FD2005	-0.90	0.30	0.77	-0.85	0.33	0.83	-0.88	0.34	0.85	-0.93	0.31	0.87	-1.02	0.27	0.78
Flavonifractor	0.18	0.81	0.96	0.13	0.86	0.98	-0.13	0.87	0.97	-0.14	0.86	0.97	-0.04	0.96	0.97
Fournierella	0.43	0.57	0.94	0.42	0.58	0.92	0.56	0.49	0.88	0.58	0.47	0.89	0.59	0.47	0.88
Frisingicoccus	0.81	0.16	0.67	0.83	0.15	0.70	1.11	0.07	0.64	1.12	0.06	0.66	1.12	0.07	0.73
Fusicatenibacter	-0.20	0.74	0.96	-0.15	0.80	0.97	0.07	0.92	0.97	0.04	0.95	0.98	0.02	0.98	0.98
Fusobacterium	2.42	0.01	0.33	2.40	0.01	0.37	2.22	0.03	0.62	2.26	0.03	0.57	2.24	0.03	0.52
GCA.900066575	-0.76	0.32	0.77	-0.67	0.38	0.85	-0.50	0.53	0.88	-0.46	0.56	0.90	-0.63	0.44	0.88
GCA.900066755	-0.40	0.75	0.96	-0.32	0.80	0.97	-0.82	0.54	0.88	-0.78	0.56	0.90	-0.71	0.60	0.89
Gordonibacter	0.55	0.46	0.84	0.54	0.47	0.89	0.85	0.28	0.85	0.92	0.25	0.80	0.90	0.26	0.77
Granulicatella	-0.87	0.46	0.84	-0.81	0.49	0.89	-1.06	0.39	0.85	-1.03	0.40	0.87	-1.01	0.41	0.88
Haemophilus	-0.73	0.21	0.67	-0.69	0.24	0.75	-0.55	0.37	0.85	-0.53	0.39	0.87	-0.54	0.38	0.88
Harryflintia	1.39	0.12	0.67	1.43	0.11	0.70	1.40	0.14	0.64	1.41	0.13	0.66	1.29	0.17	0.74
Holdemanella	-0.16	0.80	0.96	-0.16	0.80	0.97	0.20	0.76	0.95	0.17	0.80	0.95	0.19	0.78	0.97

© 2022 Meyer K et al. *JAMA Network Open.*

Holdemania	0.85	0.52	0.90	0.86	0.51	0.89	0.80	0.56	0.89	0.85	0.54	0.90	0.93	0.50	0.88
Hungatella	0.58	0.42	0.84	0.53	0.46	0.89	0.08	0.92	0.97	0.10	0.89	0.98	0.15	0.84	0.97
Hydrogenoanaerobacterium	-1.74	0.02	0.33	-1.67	0.02	0.45	-1.55	0.05	0.64	-1.53	0.06	0.66	-1.53	0.06	0.73
Incertae_Sedis	-0.34	0.79	0.96	-0.43	0.74	0.97	-0.42	0.76	0.95	-0.38	0.78	0.95	-0.41	0.77	0.97
Intestinibacter	-0.94	0.15	0.67	-1.05	0.11	0.70	-1.30	0.06	0.64	-1.31	0.06	0.66	-1.20	0.09	0.73
Intestinimonas	0.02	0.97	0.99	0.09	0.89	0.98	0.11	0.87	0.97	0.15	0.83	0.96	0.11	0.87	0.97
Lachnoclostridium	2.30	0.09	0.67	2.17	0.11	0.70	1.17	0.43	0.88	1.13	0.45	0.89	1.09	0.47	0.88
Lachnospira	-2.47	0.00	0.05	-2.37	0.00	0.09	-2.26	0.00	0.23	-2.25	0.00	0.23	-2.23	0.00	0.28
Lachnospiraceae_FCS020_group	-0.74	0.28	0.77	-0.63	0.36	0.83	-0.30	0.69	0.93	-0.29	0.71	0.94	-0.33	0.67	0.91
Lachnospiraceae_ND3007_group	0.07	0.89	0.97	0.16	0.73	0.97	0.35	0.49	0.88	0.35	0.49	0.89	0.32	0.52	0.88
Lachnospiraceae_NK4A136_grou	-0.22	0.76	0.96	-0.12	0.86	0.98	0.52	0.50	0.88	0.53	0.50	0.89	0.48	0.54	0.88
Lachnospiraceae_UCG.001	-0.45	0.37	0.84	-0.43	0.39	0.85	-0.27	0.61	0.92	-0.27	0.61	0.92	-0.31	0.55	0.88
Lachnospiraceae_UCG.004	0.10	0.85	0.97	0.16	0.78	0.97	0.35	0.56	0.89	0.33	0.57	0.90	0.33	0.57	0.88
Lachnospiraceae_UCG.010	0.29	0.72	0.96	0.40	0.63	0.94	0.84	0.33	0.85	0.85	0.33	0.87	0.77	0.38	0.88
Lactobacillus	0.37	0.66	0.96	0.21	0.80	0.97	0.11	0.90	0.97	0.09	0.92	0.98	0.41	0.65	0.91
Lactococcus	0.72	0.38	0.84	0.70	0.39	0.85	0.92	0.29	0.85	0.97	0.27	0.80	1.13	0.20	0.74
Marvinbryantia	0.53	0.31	0.77	0.59	0.26	0.77	0.71	0.20	0.79	0.74	0.18	0.73	0.78	0.16	0.74
Megasphaera	0.08	0.89	0.97	0.02	0.97	0.99	-0.21	0.74	0.95	-0.23	0.71	0.94	-0.15	0.81	0.97
Monoglobus	-1.37	0.08	0.67	-1.36	0.08	0.70	-1.50	0.07	0.64	-1.48	0.07	0.66	-1.44	0.08	0.73
Moryella	-0.41	0.64	0.94	-0.32	0.72	0.97	0.07	0.94	0.97	0.07	0.94	0.98	0.10	0.91	0.97
Negativibacillus	0.32	0.57	0.94	0.34	0.56	0.91	0.27	0.66	0.92	0.25	0.69	0.94	0.23	0.71	0.94
NK4A214_group	0.04	0.93	0.99	0.09	0.83	0.98	0.32	0.50	0.88	0.32	0.50	0.89	0.34	0.47	0.88
Odoribacter	0.45	0.32	0.77	0.42	0.34	0.83	0.70	0.14	0.64	0.71	0.14	0.66	0.61	0.20	0.74
Oscillibacter	-1.03	0.17	0.67	-0.95	0.21	0.73	-0.69	0.38	0.85	-0.68	0.39	0.87	-0.72	0.36	0.88
Oscillospira	-0.03	0.98	0.99	-0.09	0.94	0.99	-0.24	0.84	0.97	-0.22	0.86	0.97	-0.20	0.87	0.97
Oxalobacter	-0.91	0.20	0.67	-0.78	0.28	0.79	-0.49	0.53	0.88	-0.50	0.53	0.90	-0.57	0.47	0.88
Paludicola	-0.24	0.75	0.96	-0.08	0.91	0.99	0.36	0.65	0.92	0.44	0.59	0.91	0.51	0.53	0.88
Parabacteroides	-0.14	0.79	0.96	-0.24	0.65	0.95	-0.32	0.58	0.90	-0.31	0.59	0.91	-0.24	0.67	0.91
Paraprevotella	-0.33	0.42	0.84	-0.38	0.35	0.83	-0.29	0.49	0.88	-0.29	0.50	0.89	-0.27	0.53	0.88
Parasutterella	-0.30	0.47	0.84	-0.29	0.48	0.89	-0.05	0.91	0.97	-0.05	0.90	0.98	-0.12	0.79	0.97
Peptococcus	1.06	0.17	0.67	1.13	0.14	0.70	1.68	0.04	0.64	1.71	0.04	0.65	1.81	0.03	0.52
Phascolarctobacterium	-0.16	0.68	0.96	-0.19	0.63	0.94	-0.17	0.67	0.92	-0.15	0.71	0.94	-0.22	0.60	0.89
Phocea	1.60	0.10	0.67	1.61	0.10	0.70	1.72	0.10	0.64	1.70	0.10	0.66	1.79	0.09	0.73
Prevotella	0.33	0.39	0.84	0.27	0.48	0.89	0.27	0.51	0.88	0.25	0.54	0.90	0.24	0.55	0.88
Romboutsia	-1.04	0.09	0.67	-1.07	0.08	0.70	-1.06	0.10	0.64	-1.04	0.11	0.66	-0.99	0.13	0.74
Roseburia	-0.31	0.69	0.96	-0.27	0.74	0.97	-0.17	0.84	0.97	-0.18	0.82	0.96	-0.07	0.93	0.97
Ruminococcus	0.15	0.76	0.96	0.20	0.68	0.96	0.34	0.52	0.88	0.34	0.51	0.89	0.40	0.45	0.88
Sellimonas	-0.92	0.17	0.67	-0.87	0.20	0.72	-0.61	0.39	0.85	-0.60	0.40	0.87	-0.62	0.39	0.88
Shuttleworthia	-0.27	0.67	0.96	-0.15	0.82	0.98	0.32	0.64	0.92	0.33	0.64	0.94	0.50	0.48	0.88
Slackia	0.14	0.81	0.96	0.08	0.89	0.98	0.30	0.64	0.92	0.29	0.64	0.94	0.22	0.73	0.95
Streptococcus	-1.25	0.14	0.67	-1.25	0.14	0.70	-1.51	0.10	0.64	-1.49	0.11	0.66	-1.30	0.16	0.74
Subdoligranulum	0.30	0.61	0.94	0.37	0.53	0.90	0.54	0.40	0.85	0.54	0.39	0.87	0.52	0.41	0.88
Sutterella	-0.19	0.59	0.94	-0.25	0.48	0.89	-0.33	0.38	0.85	-0.32	0.39	0.87	-0.30	0.43	0.88
TM7x	0.13	0.88	0.97	0.25	0.77	0.97	0.10	0.91	0.97	0.14	0.88	0.97	0.34	0.70	0.94
Turicibacter	-1.01	0.21	0.67	-1.07	0.18	0.72	-1.18	0.15	0.64	-1.16	0.16	0.66	-1.19	0.15	0.74
Tuzzerella	0.86	0.12	0.67	0.81	0.15	0.70	0.92	0.12	0.64	0.92	0.13	0.66	0.97	0.11	0.73
Tyzzerella	-1.30	0.09	0.67	-1.30	0.09	0.70	-1.29	0.12	0.64	-1.34	0.11	0.66	-1.23	0.15	0.74
UBA1819	0.33	0.73	0.96	0.33	0.73	0.97	0.24	0.81	0.96	0.30	0.77	0.95	0.23	0.82	0.97
UCG.002	0.23	0.60	0.94	0.28	0.52	0.89	0.61	0.21	0.80	0.63	0.20	0.76	0.58	0.23	0.74
UCG.003	-0.37	0.44	0.84	-0.28	0.56	0.91	-0.02	0.97	0.97	-0.02	0.97	0.98	-0.06	0.92	0.97
UCG.005	-0.27	0.63	0.94	-0.25	0.66	0.95	0.17	0.79	0.95	0.20	0.75	0.95	0.11	0.85	0.97
UCG.009	-1.01	0.22	0.67	-0.90	0.27	0.77	-0.79	0.36	0.85	-0.75	0.38	0.87	-0.85	0.33	0.88
Veillonella	-0.64	0.31	0.77	-0.60	0.34	0.83	-0.57	0.38	0.85	-0.56	0.40	0.87	-0.51	0.44	0.88
Victivallis	-1.39	0.02	0.33	-1.32	0.02	0.45	-1.12	0.07	0.64	-1.10	0.08	0.66	-1.02	0.11	0.73

Multivariable-adjusted regression models adjusted for:

Model 1: sequencing batch, age, race, gender, study center

Model 2: Model 1, with education

Model 3: Model 2, with physical activity, diet score, medication use, and smoking

© 2022 Meyer K et al. *JAMA Network Open.*

Model 4: Model 3 with BMI
Model 5: Model 4 with hypertension and diabetes

© 2022 Meyer K et al. *JAMA Network Open.*

eTable 35: Multivariable-adjusted regression coefficients, p-values, and false discovery rate (FDR)-adjusted p-values for genera among White participants with respect to RAVLT.

	Model1			Model2			Model3			Model4			Model5		
	beta	p-value	FDR	beta	p-value	FDR	beta	p-value	FDR	beta	p-value	FDR	beta	p-value	FDR
Acetanaerobacterium	-0.08	0.69	0.85	0.04	0.86	0.96	-0.13	0.53	0.85	-0.14	0.49	0.81	-0.19	0.35	0.72
Acidaminococcus	-0.20	0.01	0.09	-0.14	0.05	0.25	-0.16	0.03	0.41	-0.16	0.03	0.42	-0.16	0.04	0.40
Actinomyces	0.09	0.67	0.85	0.09	0.64	0.83	0.15	0.46	0.78	0.15	0.47	0.79	0.16	0.44	0.81
Adlercreutzia	0.16	0.15	0.32	0.17	0.10	0.29	0.12	0.27	0.66	0.12	0.28	0.72	0.12	0.28	0.72
Agathobacter	0.02	0.89	0.94	0.01	0.91	0.97	-0.02	0.87	0.96	0.00	0.98	0.98	0.00	1.00	1.00
Akkermansia	0.09	0.19	0.38	0.08	0.27	0.50	0.06	0.36	0.75	0.06	0.37	0.75	0.06	0.39	0.77
Alistipes	0.25	0.02	0.12	0.24	0.03	0.22	0.11	0.34	0.73	0.10	0.36	0.75	0.11	0.36	0.72
Anaerofilum	0.23	0.13	0.30	0.15	0.33	0.59	0.13	0.39	0.76	0.12	0.41	0.78	0.12	0.43	0.81
Anaerostipes	0.05	0.79	0.89	0.02	0.92	0.97	-0.02	0.92	0.96	-0.04	0.83	0.96	-0.04	0.85	1.00
Anaerotruncus	-0.02	0.90	0.94	-0.05	0.75	0.91	-0.13	0.39	0.76	-0.16	0.29	0.72	-0.15	0.33	0.72
Angelakisella	0.33	0.12	0.30	0.24	0.24	0.48	0.11	0.60	0.88	0.12	0.59	0.88	0.07	0.73	0.95
Bacteroides	-0.08	0.80	0.89	-0.10	0.76	0.91	-0.01	0.97	0.97	-0.01	0.98	0.98	-0.02	0.95	1.00
Barnesiella	0.19	0.00	0.05	0.18	0.00	0.08	0.13	0.04	0.41	0.13	0.04	0.42	0.13	0.04	0.41
Bifidobacterium	-0.04	0.63	0.80	0.02	0.77	0.91	0.02	0.85	0.96	0.01	0.88	0.98	0.01	0.93	1.00
Bilophila	0.12	0.18	0.37	0.13	0.14	0.34	0.10	0.25	0.66	0.10	0.28	0.72	0.09	0.30	0.72
Blautia	-0.04	0.90	0.94	-0.16	0.62	0.81	-0.14	0.66	0.91	-0.19	0.56	0.86	-0.14	0.67	0.92
Butyricicoccus	0.17	0.46	0.65	0.07	0.75	0.91	-0.06	0.79	0.96	-0.06	0.78	0.96	-0.04	0.85	1.00
Butyricimonas	0.06	0.43	0.62	0.04	0.62	0.81	0.01	0.94	0.96	0.01	0.90	0.98	0.01	0.90	1.00
CAG.56	0.19	0.03	0.14	0.14	0.10	0.29	0.11	0.22	0.64	0.11	0.24	0.70	0.12	0.20	0.60
Candidatus_Soleaferrea	-0.26	0.15	0.32	-0.32	0.06	0.25	-0.25	0.15	0.53	-0.26	0.14	0.55	-0.28	0.12	0.53
Caproiciproducens	0.02	0.87	0.94	0.05	0.70	0.90	-0.02	0.88	0.96	-0.03	0.85	0.96	-0.03	0.84	1.00
Christensenellaceae_R.7_group	0.16	0.03	0.13	0.14	0.05	0.25	0.09	0.25	0.66	0.08	0.30	0.72	0.08	0.30	0.72
Clostridium_sensu_stricto_1	0.16	0.07	0.21	0.17	0.05	0.25	0.17	0.06	0.44	0.16	0.08	0.45	0.13	0.15	0.55
Colidextribacter	-0.24	0.25	0.46	-0.34	0.09	0.29	-0.53	0.01	0.32	-0.55	0.01	0.26	-0.57	0.01	0.20
Collinsella	-0.11	0.20	0.39	-0.06	0.47	0.72	-0.05	0.55	0.85	-0.04	0.61	0.88	-0.05	0.53	0.85
Coprobacter	0.03	0.72	0.86	-0.01	0.95	0.98	-0.06	0.47	0.78	-0.06	0.45	0.79	-0.06	0.47	0.82
Coprococcus	0.24	0.02	0.10	0.21	0.04	0.24	0.14	0.15	0.53	0.14	0.17	0.55	0.13	0.21	0.62
Defluviitaleaceae_UCG.011	0.34	0.02	0.10	0.31	0.02	0.22	0.29	0.03	0.41	0.28	0.04	0.42	0.28	0.05	0.41
Desulfovibrio	-0.03	0.75	0.88	-0.02	0.78	0.91	-0.07	0.41	0.78	-0.08	0.37	0.75	-0.07	0.43	0.81
Dialister	0.06	0.35	0.57	0.05	0.38	0.63	0.05	0.48	0.79	0.05	0.43	0.78	0.04	0.57	0.87
DNF00809	0.29	0.07	0.21	0.24	0.11	0.30	0.24	0.13	0.53	0.23	0.15	0.55	0.22	0.16	0.55
Dorea	0.06	0.70	0.85	0.09	0.51	0.74	0.03	0.81	0.96	0.03	0.81	0.96	0.06	0.68	0.92
DTU089	0.00	0.98	0.98	0.02	0.90	0.97	-0.01	0.95	0.96	-0.03	0.85	0.96	-0.01	0.91	1.00
Eggerthella	0.00	0.98	0.98	0.01	0.93	0.98	-0.03	0.80	0.96	-0.04	0.72	0.94	-0.03	0.81	1.00
Eisenbergiella	-0.19	0.16	0.34	-0.18	0.17	0.37	-0.20	0.14	0.53	-0.23	0.09	0.49	-0.21	0.12	0.53
Erysipelatoclostridium	-0.13	0.36	0.57	-0.09	0.50	0.74	-0.03	0.80	0.96	-0.06	0.69	0.94	-0.08	0.58	0.87
Erysipelotrichaceae_UCG.003	0.07	0.46	0.65	0.05	0.58	0.80	0.05	0.62	0.89	0.05	0.64	0.89	0.04	0.71	0.94
Escherichia.Shigella	-0.05	0.55	0.73	-0.04	0.61	0.81	-0.02	0.79	0.96	-0.02	0.81	0.96	-0.01	0.91	1.00
Faecalibacterium	0.22	0.06	0.18	0.22	0.06	0.25	0.21	0.08	0.44	0.21	0.08	0.45	0.20	0.10	0.49
Family_XIII_AD3011_group	0.26	0.03	0.13	0.21	0.07	0.25	0.07	0.55	0.85	0.06	0.63	0.89	0.06	0.62	0.90
Family_XIII_UCG.001	0.29	0.05	0.18	0.24	0.10	0.29	0.17	0.25	0.66	0.15	0.31	0.72	0.15	0.33	0.72
FD2005	0.05	0.75	0.88	0.01	0.97	1.00	0.06	0.69	0.94	0.08	0.60	0.88	0.06	0.69	0.92
Flavonifractor	-0.26	0.05	0.16	-0.23	0.07	0.25	-0.22	0.08	0.44	-0.22	0.08	0.45	-0.23	0.08	0.48
Fournierella	0.10	0.47	0.65	0.10	0.42	0.67	0.13	0.33	0.73	0.12	0.36	0.75	0.12	0.36	0.72
Frisingicoccus	-0.11	0.29	0.52	-0.12	0.22	0.46	-0.20	0.05	0.41	-0.20	0.04	0.42	-0.19	0.06	0.48
Fusicatenibacter	0.16	0.13	0.30	0.12	0.23	0.46	0.06	0.57	0.85	0.07	0.51	0.82	0.07	0.51	0.83
Fusobacterium	-0.15	0.39	0.59	-0.14	0.40	0.65	0.02	0.88	0.96	0.01	0.94	0.98	0.00	0.99	1.00
GCA.900066575	-0.03	0.83	0.91	-0.09	0.47	0.72	-0.16	0.21	0.64	-0.18	0.17	0.55	-0.21	0.11	0.53
GCA.900066755	0.38	0.08	0.24	0.33	0.12	0.30	0.37	0.10	0.49	0.35	0.12	0.52	0.34	0.13	0.54
Gordonibacter	0.17	0.20	0.39	0.18	0.17	0.37	0.11	0.42	0.78	0.09	0.51	0.82	0.10	0.45	0.82
Granulicatella	0.20	0.33	0.56	0.15	0.45	0.70	0.21	0.31	0.73	0.20	0.33	0.75	0.22	0.29	0.72
Haemophilus	0.20	0.06	0.18	0.16	0.11	0.30	0.15	0.13	0.53	0.15	0.16	0.55	0.15	0.15	0.55
Harryflintia	0.05	0.76	0.88	0.02	0.88	0.97	0.02	0.90	0.96	0.01	0.92	0.98	-0.01	0.95	1.00
Holdemanella	0.02	0.83	0.91	0.03	0.82	0.95	-0.02	0.82	0.96	-0.01	0.91	0.98	-0.01	0.96	1.00

© 2022 Meyer K et al. *JAMA Network Open.*

Holdemania	0.02	0.95	0.96	0.00	1.00	1.00	-0.04	0.87	0.96	-0.06	0.80	0.96	-0.12	0.61	0.90
Hungatella	-0.38	0.00	0.05	-0.34	0.00	0.10	-0.24	0.06	0.43	-0.25	0.05	0.42	-0.24	0.06	0.48
Hydrogenoanaerobacterium	0.35	0.01	0.09	0.30	0.02	0.20	0.20	0.13	0.53	0.18	0.17	0.55	0.19	0.16	0.55
Incertae_Sedis	0.16	0.49	0.66	0.22	0.31	0.56	0.13	0.56	0.85	0.12	0.61	0.88	0.14	0.53	0.85
Intestinibacter	0.14	0.22	0.41	0.21	0.06	0.25	0.20	0.08	0.44	0.21	0.07	0.45	0.20	0.09	0.48
Intestinimonas	0.29	0.01	0.09	0.24	0.03	0.22	0.23	0.04	0.41	0.21	0.06	0.43	0.24	0.04	0.40
Lachnoclostridium	-0.53	0.03	0.13	-0.43	0.07	0.25	-0.20	0.43	0.78	-0.18	0.47	0.79	-0.15	0.56	0.87
Lachnospira	0.55	0.00	0.00	0.44	0.00	0.07	0.46	0.00	0.03	0.45	0.00	0.04	0.44	0.00	0.03
Lachnospiraceae_FCS020_group	0.35	0.00	0.05	0.27	0.02	0.20	0.18	0.14	0.53	0.18	0.15	0.55	0.18	0.16	0.55
Lachnospiraceae_ND3007_group	0.28	0.00	0.04	0.22	0.01	0.11	0.18	0.03	0.41	0.18	0.03	0.42	0.18	0.03	0.40
Lachnospiraceae_NK4A136_grou	0.14	0.28	0.50	0.08	0.54	0.76	-0.05	0.71	0.96	-0.05	0.69	0.94	-0.05	0.69	0.92
Lachnospiraceae_UCG.001	0.23	0.01	0.09	0.21	0.01	0.16	0.20	0.02	0.41	0.20	0.02	0.42	0.20	0.02	0.37
Lachnospiraceae_UCG.004	0.03	0.74	0.88	0.00	0.99	1.00	0.02	0.83	0.96	0.03	0.79	0.96	0.00	0.98	1.00
Lachnospiraceae_UCG.010	-0.10	0.52	0.69	-0.16	0.25	0.48	-0.21	0.14	0.53	-0.21	0.13	0.55	-0.20	0.16	0.55
Lactobacillus	-0.29	0.04	0.16	-0.19	0.18	0.39	-0.20	0.16	0.53	-0.19	0.18	0.56	-0.19	0.19	0.60
Lactococcus	0.01	0.93	0.96	0.02	0.87	0.96	0.05	0.74	0.96	0.03	0.83	0.96	0.02	0.88	1.00
Marvinbryantia	0.02	0.80	0.89	-0.01	0.89	0.97	0.01	0.95	0.96	0.00	0.98	0.98	-0.01	0.95	1.00
Megasphaera	-0.18	0.09	0.24	-0.14	0.17	0.38	-0.10	0.35	0.73	-0.09	0.40	0.78	-0.10	0.34	0.72
Monoglobus	0.41	0.00	0.05	0.40	0.00	0.08	0.46	0.00	0.03	0.45	0.00	0.04	0.47	0.00	0.03
Moryella	0.30	0.05	0.18	0.23	0.12	0.31	0.12	0.45	0.78	0.12	0.45	0.79	0.11	0.46	0.82
Negativibacillus	-0.11	0.30	0.52	-0.11	0.25	0.48	-0.11	0.27	0.66	-0.11	0.31	0.72	-0.11	0.28	0.72
NK4A214_group	0.16	0.04	0.16	0.12	0.10	0.29	0.06	0.45	0.78	0.06	0.45	0.79	0.05	0.49	0.82
Odoribacter	0.08	0.33	0.56	0.09	0.24	0.48	0.01	0.93	0.96	0.00	0.97	0.98	0.00	0.98	1.00
Oscillibacter	0.28	0.03	0.14	0.23	0.08	0.27	0.11	0.40	0.77	0.10	0.43	0.78	0.10	0.46	0.82
Oscillospira	-0.02	0.92	0.95	0.03	0.86	0.96	0.04	0.85	0.96	0.03	0.89	0.98	0.03	0.87	1.00
Oxalobacter	0.42	0.00	0.04	0.32	0.01	0.16	0.21	0.11	0.49	0.21	0.10	0.49	0.24	0.07	0.48
Paludicola	0.14	0.30	0.52	0.03	0.83	0.96	-0.12	0.38	0.76	-0.15	0.28	0.72	-0.13	0.35	0.72
Parabacteroides	-0.13	0.18	0.37	-0.06	0.51	0.74	-0.03	0.75	0.96	-0.03	0.72	0.94	-0.04	0.66	0.92
Paraprevotella	0.07	0.36	0.57	0.10	0.16	0.37	0.07	0.33	0.73	0.07	0.35	0.75	0.07	0.35	0.72
Parasutterella	0.12	0.10	0.26	0.11	0.10	0.29	0.04	0.58	0.85	0.04	0.56	0.86	0.04	0.58	0.87
Peptococcus	0.11	0.40	0.59	0.07	0.61	0.81	-0.02	0.86	0.96	-0.03	0.81	0.96	-0.02	0.88	1.00
Phascolarctobacterium	-0.03	0.63	0.80	-0.02	0.77	0.91	-0.01	0.85	0.96	-0.02	0.75	0.96	-0.01	0.87	1.00
Phocea	-0.15	0.39	0.59	-0.15	0.37	0.63	-0.21	0.21	0.64	-0.20	0.24	0.70	-0.23	0.19	0.60
Prevotella	-0.16	0.02	0.10	-0.12	0.07	0.25	-0.12	0.07	0.44	-0.12	0.08	0.45	-0.12	0.09	0.48
Romboutsia	0.08	0.48	0.65	0.10	0.36	0.63	0.05	0.64	0.91	0.04	0.70	0.94	0.02	0.89	1.00
Roseburia	0.12	0.38	0.59	0.09	0.50	0.74	0.11	0.43	0.78	0.11	0.40	0.78	0.10	0.49	0.82
Ruminococcus	0.21	0.01	0.10	0.18	0.04	0.24	0.15	0.08	0.44	0.15	0.08	0.45	0.15	0.09	0.48
Sellimonas	0.30	0.01	0.09	0.26	0.02	0.22	0.27	0.02	0.41	0.27	0.02	0.42	0.28	0.02	0.37
Shuttleworthia	0.29	0.01	0.09	0.21	0.06	0.25	0.11	0.33	0.73	0.11	0.34	0.75	0.12	0.29	0.72
Slackia	-0.04	0.70	0.85	0.00	0.99	1.00	-0.01	0.93	0.96	-0.01	0.94	0.98	-0.02	0.87	1.00
Streptococcus	0.12	0.43	0.62	0.12	0.39	0.64	0.20	0.19	0.61	0.17	0.26	0.72	0.18	0.26	0.72
Subdoligranulum	0.23	0.03	0.14	0.18	0.07	0.25	0.11	0.28	0.68	0.11	0.29	0.72	0.10	0.34	0.72
Sutterella	-0.07	0.27	0.49	-0.03	0.57	0.80	-0.02	0.74	0.96	-0.02	0.71	0.94	-0.03	0.62	0.90
TM7x	0.14	0.37	0.57	0.05	0.73	0.91	0.02	0.90	0.96	0.00	0.97	0.98	0.00	0.99	1.00
Turicibacter	0.36	0.01	0.09	0.40	0.00	0.08	0.41	0.00	0.08	0.40	0.00	0.10	0.38	0.01	0.20
Tuzzerella	-0.23	0.02	0.11	-0.19	0.04	0.25	-0.19	0.05	0.43	-0.19	0.05	0.43	-0.21	0.03	0.40
Tyzzerella	0.20	0.14	0.32	0.20	0.13	0.32	0.19	0.18	0.58	0.20	0.15	0.55	0.24	0.09	0.48
UBA1819	-0.09	0.62	0.80	-0.08	0.61	0.81	-0.20	0.24	0.66	-0.22	0.19	0.59	-0.22	0.20	0.60
UCG.002	0.16	0.04	0.15	0.13	0.09	0.29	0.05	0.55	0.85	0.04	0.60	0.88	0.03	0.68	0.92
UCG.003	0.15	0.09	0.24	0.08	0.35	0.61	0.01	0.92	0.96	0.01	0.93	0.98	0.00	0.98	1.00
UCG.005	0.16	0.10	0.26	0.15	0.12	0.31	0.07	0.47	0.78	0.06	0.54	0.85	0.07	0.49	0.82
UCG.009	0.21	0.15	0.32	0.13	0.37	0.63	0.06	0.66	0.91	0.05	0.75	0.96	0.05	0.75	0.96
Veillonella	0.17	0.12	0.29	0.15	0.16	0.37	0.18	0.10	0.49	0.17	0.11	0.50	0.17	0.12	0.53
Victivallis	0.26	0.01	0.09	0.21	0.04	0.24	0.12	0.27	0.66	0.11	0.31	0.72	0.11	0.30	0.72

Multivariable-adjusted regression models adjusted for:

Model 1: sequencing batch, age, race, gender, study center

Model 2: Model 1, with education

Model 3: Model 2, with physical activity, diet score, medication use, and smoking

© 2022 Meyer K et al. *JAMA Network Open.*

Model 4: Model 3 with BMI

Model 5: Model 4 with hypertension and diabetes

© 2022 Meyer K et al. *JAMA Network Open.*

eTable 36: Multivariable-adjusted regression coefficients, p-values, and false discovery rate (FDR)-adjusted p-values for genera among White participants with respect to category fluency.

	Model1			Model2			Model3			Model4			Model5		
	beta	p-value	FDR	beta	p-value	FDR	beta	p-value	FDR	beta	p-value	FDR	beta	p-value	FDR
Acetanaerobacterium	0.11	0.86	0.98	0.46	0.43	0.83	0.30	0.62	0.88	0.27	0.66	0.87	0.11	0.86	0.92
Acidaminococcus	-0.40	0.06	0.53	-0.23	0.28	0.77	-0.17	0.46	0.85	-0.16	0.48	0.86	-0.17	0.47	0.85
Actinomyces	0.22	0.72	0.94	0.23	0.68	0.87	0.34	0.57	0.88	0.34	0.58	0.87	0.42	0.49	0.86
Adlercreutzia	0.26	0.41	0.89	0.30	0.32	0.79	0.31	0.34	0.77	0.31	0.35	0.79	0.28	0.40	0.82
Agathobacter	0.34	0.39	0.89	0.33	0.39	0.83	0.19	0.65	0.88	0.23	0.57	0.87	0.29	0.48	0.86
Akkermansia	-0.21	0.32	0.87	-0.26	0.19	0.77	-0.30	0.15	0.72	-0.31	0.14	0.68	-0.33	0.12	0.59
Alistipes	-0.22	0.51	0.89	-0.26	0.41	0.83	-0.41	0.23	0.76	-0.42	0.23	0.75	-0.48	0.17	0.59
Anaerofilum	0.22	0.63	0.93	-0.05	0.90	0.95	-0.13	0.77	0.90	-0.15	0.74	0.88	-0.13	0.78	0.90
Anaerostipes	-0.18	0.75	0.94	-0.28	0.61	0.87	-0.51	0.37	0.79	-0.56	0.32	0.78	-0.56	0.33	0.80
Anaerotruncus	-0.36	0.41	0.89	-0.44	0.29	0.77	-0.51	0.25	0.76	-0.58	0.20	0.69	-0.63	0.17	0.59
Angelakisella	-0.17	0.78	0.94	-0.45	0.46	0.86	-0.61	0.35	0.77	-0.60	0.36	0.79	-0.62	0.35	0.80
Bacteroides	-0.88	0.37	0.89	-0.92	0.33	0.79	-1.04	0.30	0.76	-1.04	0.30	0.77	-1.04	0.30	0.80
Barnesiella	0.57	0.00	0.12	0.54	0.00	0.24	0.56	0.00	0.36	0.56	0.00	0.36	0.57	0.00	0.31
Bifidobacterium	-0.43	0.08	0.54	-0.25	0.30	0.78	-0.24	0.32	0.77	-0.25	0.31	0.78	-0.25	0.32	0.80
Bilophila	0.42	0.12	0.58	0.43	0.09	0.73	0.54	0.04	0.70	0.53	0.05	0.61	0.55	0.04	0.59
Blautia	-0.08	0.93	0.99	-0.42	0.65	0.87	-0.33	0.74	0.89	-0.43	0.66	0.87	-0.39	0.69	0.88
Butyricicoccus	0.42	0.53	0.89	0.13	0.84	0.90	-0.17	0.80	0.90	-0.17	0.80	0.88	-0.19	0.78	0.90
Butyricimonas	0.07	0.74	0.94	0.00	0.98	0.99	-0.03	0.90	0.95	-0.02	0.93	0.96	-0.02	0.94	0.96
CAG.56	0.41	0.11	0.58	0.27	0.29	0.77	0.21	0.45	0.85	0.20	0.46	0.86	0.20	0.46	0.85
Candidatus_Soleaferrea	-0.25	0.63	0.93	-0.43	0.39	0.83	-0.31	0.56	0.88	-0.32	0.55	0.87	-0.43	0.43	0.84
Caproiciproducens	-0.62	0.15	0.64	-0.54	0.20	0.77	-0.58	0.19	0.76	-0.59	0.18	0.68	-0.63	0.16	0.59
Christensenellaceae_R.7_group	0.07	0.77	0.94	0.01	0.98	0.99	-0.02	0.92	0.95	-0.04	0.86	0.93	-0.06	0.80	0.90
Clostridium_sensu_stricto_1	0.27	0.31	0.87	0.28	0.27	0.77	0.27	0.31	0.76	0.25	0.34	0.79	0.24	0.38	0.82
Colidextribacter	0.17	0.78	0.94	-0.13	0.82	0.89	-0.17	0.80	0.90	-0.20	0.76	0.88	-0.16	0.80	0.90
Collinsella	0.20	0.43	0.89	0.36	0.14	0.77	0.42	0.10	0.70	0.44	0.09	0.61	0.41	0.11	0.59
Coprobacter	0.18	0.48	0.89	0.07	0.77	0.89	-0.10	0.69	0.88	-0.11	0.67	0.87	-0.11	0.66	0.86
Coprococcus	0.20	0.51	0.89	0.10	0.73	0.87	-0.03	0.92	0.95	-0.04	0.90	0.96	-0.04	0.90	0.95
Defluviitaleaceae_UCG.011	0.45	0.27	0.87	0.36	0.36	0.82	0.31	0.46	0.85	0.28	0.50	0.87	0.25	0.56	0.86
Desulfovibrio	-0.69	0.01	0.20	-0.68	0.01	0.31	-0.66	0.01	0.57	-0.68	0.01	0.48	-0.67	0.01	0.54
Dialister	0.09	0.62	0.93	0.08	0.68	0.87	0.09	0.66	0.88	0.10	0.61	0.87	0.09	0.65	0.86
DNF00809	-0.51	0.27	0.87	-0.64	0.15	0.77	-0.78	0.10	0.70	-0.80	0.09	0.61	-0.79	0.10	0.59
Dorea	-0.24	0.56	0.89	-0.14	0.73	0.87	-0.22	0.59	0.88	-0.22	0.60	0.87	-0.21	0.62	0.86
DTU089	-0.60	0.12	0.58	-0.56	0.13	0.77	-0.56	0.15	0.72	-0.60	0.13	0.68	-0.60	0.13	0.59
Eggerthella	0.16	0.67	0.94	0.18	0.62	0.87	0.13	0.72	0.89	0.11	0.78	0.88	0.14	0.71	0.89
Eisenbergiella	-0.57	0.16	0.64	-0.54	0.17	0.77	-0.61	0.13	0.72	-0.68	0.09	0.61	-0.63	0.13	0.59
Erysipelatoclostridium	-0.71	0.08	0.54	-0.60	0.12	0.77	-0.52	0.20	0.76	-0.57	0.16	0.68	-0.57	0.17	0.59
Erysipelotrichaceae_UCG.003	0.29	0.33	0.87	0.23	0.42	0.83	0.25	0.42	0.85	0.24	0.43	0.86	0.21	0.49	0.86
Escherichia.Shigella	-0.20	0.46	0.89	-0.17	0.50	0.87	-0.01	0.96	0.96	-0.01	0.98	0.98	0.02	0.93	0.96
Faecalibacterium	0.05	0.88	0.99	0.03	0.93	0.96	0.08	0.82	0.90	0.09	0.81	0.88	0.08	0.83	0.91
Family_XIII_AD3011_group	-0.22	0.54	0.89	-0.38	0.27	0.77	-0.53	0.15	0.72	-0.57	0.13	0.68	-0.60	0.11	0.59
Family_XIII_UCG.001	0.31	0.48	0.89	0.15	0.73	0.87	0.07	0.87	0.93	0.03	0.95	0.97	-0.02	0.97	0.97
FD2005	0.39	0.39	0.89	0.26	0.54	0.87	0.32	0.48	0.86	0.37	0.43	0.86	0.31	0.51	0.86
Flavonifractor	-0.33	0.38	0.89	-0.24	0.51	0.87	-0.19	0.63	0.88	-0.18	0.63	0.87	-0.16	0.68	0.88
Fournierella	-0.20	0.62	0.93	-0.17	0.65	0.87	-0.31	0.45	0.85	-0.32	0.43	0.86	-0.35	0.39	0.82
Frisingicoccus	0.03	0.92	0.99	-0.01	0.99	0.99	-0.06	0.84	0.91	-0.07	0.81	0.88	-0.05	0.88	0.93
Fusicatenibacter	0.60	0.05	0.52	0.49	0.09	0.73	0.43	0.17	0.74	0.45	0.15	0.68	0.44	0.16	0.59
Fusobacterium	-0.12	0.82	0.95	-0.09	0.85	0.90	0.16	0.76	0.90	0.13	0.80	0.88	0.13	0.80	0.90
GCA.900066575	0.01	0.99	0.99	-0.18	0.63	0.87	-0.18	0.65	0.88	-0.22	0.59	0.87	-0.33	0.41	0.82
GCA.900066755	0.19	0.76	0.94	0.06	0.92	0.96	0.24	0.72	0.89	0.21	0.76	0.88	0.20	0.77	0.90
Gordonibacter	0.24	0.53	0.89	0.26	0.49	0.87	0.24	0.55	0.88	0.20	0.62	0.87	0.23	0.56	0.86
Granulicatella	0.45	0.46	0.89	0.31	0.60	0.87	0.46	0.45	0.85	0.44	0.47	0.86	0.48	0.44	0.84
Haemophilus	0.46	0.13	0.58	0.36	0.22	0.77	0.32	0.30	0.76	0.30	0.33	0.78	0.29	0.35	0.80
Harryflintia	-0.04	0.94	0.99	-0.11	0.80	0.89	-0.03	0.94	0.96	-0.05	0.92	0.96	-0.12	0.80	0.90
Holdemanella	-0.19	0.55	0.89	-0.19	0.54	0.87	-0.19	0.56	0.88	-0.17	0.61	0.87	-0.15	0.65	0.86

© 2022 Meyer K et al. *JAMA Network Open.*

Holdemania	0.48	0.48	0.89	0.43	0.51	0.87	0.27	0.70	0.88	0.22	0.74	0.88	0.16	0.82	0.90
Hungatella	-0.96	0.01	0.20	-0.84	0.02	0.42	-0.74	0.05	0.70	-0.76	0.04	0.61	-0.75	0.05	0.59
Hydrogenoanaerobacterium	-0.36	0.34	0.87	-0.53	0.15	0.77	-0.69	0.08	0.70	-0.73	0.07	0.61	-0.75	0.06	0.59
Incertae_Sedis	0.09	0.89	0.99	0.28	0.66	0.87	0.30	0.66	0.88	0.26	0.70	0.88	0.30	0.65	0.86
Intestinibacter	0.00	0.99	0.99	0.20	0.53	0.87	0.15	0.66	0.88	0.16	0.64	0.87	0.15	0.66	0.86
Intestinimonas	-0.13	0.69	0.94	-0.28	0.38	0.83	-0.37	0.27	0.76	-0.41	0.23	0.75	-0.39	0.25	0.77
Lachnoclostridium	-0.16	0.82	0.95	0.15	0.82	0.89	0.52	0.48	0.86	0.56	0.45	0.86	0.65	0.39	0.82
Lachnospira	1.22	0.00	0.12	0.89	0.02	0.42	0.83	0.03	0.70	0.82	0.04	0.61	0.81	0.04	0.59
Lachnospiraceae_FCS020_group	0.67	0.06	0.52	0.43	0.21	0.77	0.41	0.27	0.76	0.40	0.28	0.77	0.40	0.29	0.80
Lachnospiraceae_ND3007_group	0.46	0.06	0.52	0.27	0.25	0.77	0.19	0.45	0.85	0.19	0.44	0.86	0.18	0.47	0.85
Lachnospiraceae_NK4A136_group	0.29	0.43	0.89	0.11	0.76	0.89	-0.15	0.71	0.89	-0.15	0.70	0.88	-0.18	0.64	0.86
Lachnospiraceae_UCG.001	0.27	0.29	0.87	0.23	0.36	0.82	0.17	0.52	0.88	0.17	0.53	0.87	0.14	0.59	0.86
Lachnospiraceae_UCG.004	0.01	0.99	0.99	-0.10	0.73	0.87	-0.14	0.64	0.88	-0.13	0.67	0.87	-0.18	0.55	0.86
Lachnospiraceae_UCG.010	-0.56	0.19	0.73	-0.76	0.07	0.73	-0.81	0.06	0.70	-0.82	0.06	0.61	-0.81	0.06	0.59
Lactobacillus	-0.75	0.08	0.54	-0.44	0.29	0.77	-0.37	0.39	0.83	-0.35	0.42	0.86	-0.24	0.59	0.86
Lactococcus	0.40	0.35	0.87	0.43	0.29	0.77	0.62	0.15	0.72	0.60	0.17	0.68	0.66	0.13	0.59
Marvinbryantia	0.00	0.99	0.99	-0.11	0.67	0.87	-0.12	0.67	0.88	-0.14	0.62	0.87	-0.13	0.63	0.86
Megasphaera	-0.29	0.34	0.87	-0.16	0.58	0.87	0.08	0.81	0.90	0.10	0.76	0.88	0.11	0.73	0.89
Monoglobus	0.79	0.05	0.52	0.77	0.04	0.63	0.72	0.07	0.70	0.70	0.09	0.61	0.77	0.06	0.59
Moryella	0.78	0.08	0.54	0.58	0.18	0.77	0.49	0.29	0.76	0.49	0.29	0.77	0.48	0.31	0.80
Negativibacillus	-0.18	0.54	0.89	-0.20	0.48	0.87	-0.21	0.50	0.86	-0.19	0.54	0.87	-0.20	0.52	0.86
NK4A214_group	-0.05	0.82	0.95	-0.15	0.50	0.87	-0.26	0.28	0.76	-0.26	0.28	0.77	-0.27	0.25	0.77
Odoribacter	0.02	0.94	0.99	0.06	0.80	0.89	0.02	0.92	0.95	0.02	0.95	0.97	-0.01	0.97	0.97
Oscillibacter	0.30	0.45	0.89	0.13	0.72	0.87	0.02	0.95	0.96	0.01	0.98	0.98	-0.03	0.95	0.96
Oscillospira	0.07	0.91	0.99	0.23	0.69	0.87	0.35	0.55	0.88	0.34	0.58	0.87	0.36	0.55	0.86
Oxalobacter	0.41	0.27	0.87	0.10	0.79	0.89	0.11	0.78	0.90	0.12	0.77	0.88	0.14	0.72	0.89
Paludicola	-0.30	0.43	0.89	-0.64	0.08	0.73	-0.75	0.06	0.70	-0.83	0.04	0.61	-0.81	0.05	0.59
Parabacteroides	0.08	0.77	0.94	0.28	0.29	0.77	0.41	0.14	0.72	0.40	0.15	0.68	0.41	0.14	0.59
Paraprevotella	-0.13	0.52	0.89	-0.05	0.81	0.89	-0.06	0.78	0.90	-0.06	0.76	0.88	-0.07	0.75	0.90
Parasutterella	0.44	0.04	0.52	0.42	0.04	0.63	0.40	0.07	0.70	0.40	0.06	0.61	0.38	0.08	0.59
Peptococcus	-0.17	0.66	0.94	-0.32	0.41	0.83	-0.41	0.31	0.76	-0.43	0.29	0.77	-0.39	0.34	0.80
Phascolarctobacterium	0.01	0.95	0.99	0.05	0.78	0.89	0.10	0.62	0.88	0.08	0.68	0.87	0.10	0.64	0.86
Phocea	-0.49	0.34	0.87	-0.49	0.32	0.79	-0.53	0.30	0.76	-0.51	0.33	0.78	-0.50	0.33	0.80
Prevotella	-0.05	0.79	0.94	0.08	0.68	0.87	0.08	0.68	0.88	0.10	0.62	0.87	0.10	0.64	0.86
Romboutsia	0.42	0.18	0.70	0.48	0.12	0.77	0.36	0.26	0.76	0.34	0.29	0.77	0.32	0.33	0.80
Roseburia	0.63	0.13	0.58	0.54	0.18	0.77	0.47	0.25	0.76	0.48	0.24	0.76	0.49	0.24	0.77
Ruminococcus	0.29	0.25	0.87	0.18	0.46	0.86	0.09	0.74	0.89	0.08	0.75	0.88	0.07	0.79	0.90
Sellimonas	0.71	0.04	0.52	0.60	0.08	0.73	0.66	0.07	0.70	0.65	0.07	0.61	0.68	0.06	0.59
Shuttleworthia	-0.14	0.68	0.94	-0.41	0.21	0.77	-0.57	0.10	0.70	-0.58	0.10	0.61	-0.51	0.14	0.59
Slackia	-0.67	0.03	0.52	-0.55	0.07	0.73	-0.48	0.13	0.72	-0.47	0.14	0.68	-0.51	0.11	0.59
Streptococcus	0.14	0.75	0.94	0.15	0.72	0.87	0.44	0.34	0.77	0.38	0.41	0.86	0.43	0.36	0.80
Subdoligranulum	0.82	0.01	0.20	0.69	0.02	0.42	0.54	0.08	0.70	0.54	0.09	0.61	0.52	0.10	0.59
Sutterella	-0.01	0.98	0.99	0.10	0.57	0.87	0.12	0.52	0.88	0.12	0.54	0.87	0.12	0.53	0.86
TM7x	0.41	0.35	0.87	0.16	0.71	0.87	0.08	0.86	0.93	0.05	0.91	0.96	0.08	0.85	0.92
Turicibacter	0.68	0.10	0.58	0.80	0.05	0.63	0.76	0.06	0.70	0.74	0.07	0.61	0.70	0.09	0.59
Tuzzerella	0.15	0.59	0.93	0.27	0.34	0.79	0.32	0.28	0.76	0.32	0.28	0.77	0.31	0.29	0.80
Tyzzerella	0.58	0.15	0.64	0.57	0.14	0.77	0.54	0.19	0.76	0.58	0.17	0.68	0.74	0.08	0.59
UBA1819	-0.62	0.22	0.79	-0.61	0.21	0.77	-0.62	0.22	0.76	-0.67	0.19	0.68	-0.67	0.19	0.65
UCG.002	-0.07	0.74	0.94	-0.17	0.43	0.83	-0.30	0.21	0.76	-0.32	0.19	0.68	-0.36	0.14	0.59
UCG.003	0.11	0.66	0.94	-0.10	0.70	0.87	-0.16	0.54	0.88	-0.16	0.54	0.87	-0.17	0.51	0.86
UCG.005	-0.10	0.72	0.94	-0.15	0.59	0.87	-0.30	0.33	0.77	-0.33	0.29	0.77	-0.35	0.26	0.77
UCG.009	-0.17	0.68	0.94	-0.43	0.29	0.77	-0.58	0.17	0.74	-0.63	0.14	0.68	-0.69	0.11	0.59
Veillonella	0.23	0.47	0.89	0.16	0.60	0.87	0.17	0.59	0.88	0.16	0.62	0.87	0.15	0.64	0.86
Victivallis	0.50	0.10	0.58	0.35	0.24	0.77	0.25	0.42	0.85	0.23	0.46	0.86	0.27	0.40	0.82

Multivariable-adjusted regression models adjusted for:
Model 1: sequencing batch, age, race, gender, study center
Model 2: Model 1, with education
Model 3: Model 2, with physical activity, diet score, medication use, and smoking

© 2022 Meyer K et al. *JAMA Network Open.*

Model 4: Model 3 with BMI

Model 5: Model 4 with hypertension and diabetes

© 2022 Meyer K et al. *JAMA Network Open.*

eTable 37: Multivariable-adjusted regression coefficients, p-values, and false discovery rate (FDR)-adjusted p-values for genera among White participants with respect to letter fluency.

	Model1			Model2			Model3			Model4			Model5		
	beta	p-value	FDR	beta	p-value	FDR	beta	p-value	FDR	beta	p-value	FDR	beta	p-value	FDR
Acetanaerobacterium	0.69	0.64	0.94	1.33	0.35	0.87	1.21	0.41	0.94	1.07	0.47	0.92	0.83	0.57	0.93
Acidaminococcus	-1.21	0.02	0.89	-0.91	0.08	0.87	-0.69	0.20	0.94	-0.66	0.22	0.92	-0.63	0.24	0.93
Actinomyces	-0.22	0.88	0.99	-0.20	0.89	1.00	0.66	0.65	0.94	0.63	0.66	0.92	0.76	0.60	0.93
Adlercreutzia	-1.27	0.09	0.89	-1.20	0.11	0.87	-1.67	0.03	0.82	-1.70	0.03	0.82	-1.66	0.03	0.92
Agathobacter	-0.11	0.91	0.99	-0.13	0.89	1.00	-0.46	0.64	0.94	-0.26	0.79	0.96	-0.22	0.82	0.97
Akkermansia	-0.45	0.37	0.94	-0.54	0.27	0.87	-0.69	0.17	0.94	-0.71	0.16	0.84	-0.67	0.18	0.93
Alistipes	0.79	0.32	0.93	0.71	0.36	0.87	0.19	0.82	0.94	0.14	0.86	0.98	0.16	0.85	0.97
Anaerofilum	0.46	0.67	0.94	-0.04	0.97	1.00	-0.42	0.70	0.94	-0.50	0.65	0.92	-0.48	0.66	0.93
Anaerostipes	-0.15	0.91	0.99	-0.34	0.79	1.00	0.03	0.98	0.99	-0.20	0.89	0.98	-0.04	0.98	0.99
Anaerotruncus	-0.81	0.44	0.94	-0.95	0.35	0.87	-1.41	0.18	0.94	-1.73	0.10	0.82	-1.69	0.11	0.92
Angelakisella	2.87	0.05	0.89	2.39	0.10	0.87	1.73	0.26	0.94	1.78	0.25	0.92	1.47	0.34	0.93
Bacteroides	-2.17	0.35	0.94	-2.25	0.32	0.87	-1.29	0.59	0.94	-1.26	0.60	0.92	-1.49	0.53	0.93
Barnesiella	0.96	0.03	0.89	0.93	0.03	0.87	0.79	0.08	0.82	0.79	0.08	0.82	0.77	0.09	0.92
Bifidobacterium	-0.65	0.27	0.93	-0.31	0.59	1.00	-0.32	0.59	0.94	-0.35	0.55	0.92	-0.36	0.54	0.93
Bilophila	0.96	0.13	0.89	0.99	0.11	0.87	1.04	0.10	0.82	0.98	0.13	0.82	0.91	0.15	0.93
Blautia	-4.13	0.07	0.89	-4.78	0.04	0.87	-4.06	0.08	0.82	-4.62	0.05	0.82	-4.34	0.06	0.92
Butyricicoccus	-1.81	0.25	0.93	-2.35	0.13	0.87	-3.45	0.03	0.82	-3.47	0.03	0.82	-3.08	0.06	0.92
Butyricimonas	-0.11	0.83	0.99	-0.24	0.65	1.00	-0.32	0.55	0.94	-0.28	0.60	0.92	-0.32	0.56	0.93
CAG.56	0.69	0.27	0.93	0.43	0.48	1.00	0.32	0.62	0.94	0.27	0.67	0.92	0.29	0.65	0.93
Candidatus_Soleaferrea	-0.95	0.45	0.94	-1.28	0.30	0.87	-0.82	0.52	0.94	-0.87	0.49	0.92	-1.02	0.42	0.93
Caproiciproducens	-0.40	0.70	0.97	-0.25	0.81	1.00	-0.58	0.59	0.94	-0.65	0.54	0.92	-0.61	0.56	0.93
Christensenellaceae_R.7_group	0.03	0.95	0.99	-0.07	0.90	1.00	-0.56	0.31	0.94	-0.66	0.23	0.92	-0.57	0.30	0.93
Clostridium_sensu_stricto_1	0.89	0.16	0.89	0.92	0.13	0.87	0.80	0.21	0.94	0.70	0.27	0.92	0.57	0.38	0.93
Colidextribacter	0.39	0.79	0.99	-0.17	0.91	1.00	-0.91	0.55	0.94	-1.07	0.49	0.92	-1.02	0.51	0.93
Collinsella	-0.47	0.44	0.94	-0.19	0.76	1.00	-0.14	0.82	0.94	-0.05	0.94	0.98	-0.01	0.99	0.99
Coprobacter	0.79	0.19	0.93	0.61	0.30	0.87	0.33	0.59	0.94	0.31	0.62	0.92	0.33	0.59	0.93
Coprococcus	1.08	0.12	0.89	0.91	0.19	0.87	0.62	0.39	0.94	0.57	0.43	0.92	0.51	0.48	0.93
Defluviitaleaceae_UCG.011	-0.18	0.85	0.99	-0.34	0.72	1.00	-0.71	0.47	0.94	-0.87	0.38	0.92	-0.88	0.38	0.93
Desulfovibrio	-0.71	0.24	0.93	-0.68	0.26	0.87	-0.97	0.12	0.82	-1.05	0.09	0.82	-1.02	0.10	0.92
Dialister	0.01	0.99	0.99	-0.02	0.96	1.00	0.15	0.76	0.94	0.21	0.65	0.92	0.16	0.74	0.94
DNF00809	-0.12	0.91	0.99	-0.37	0.73	1.00	-0.75	0.51	0.94	-0.84	0.46	0.92	-0.90	0.43	0.93
Dorea	-1.09	0.27	0.93	-0.90	0.36	0.87	-0.80	0.42	0.94	-0.80	0.42	0.92	-0.77	0.44	0.93
DTU089	-0.05	0.95	0.99	0.01	0.99	1.00	-0.23	0.81	0.94	-0.42	0.66	0.92	-0.30	0.75	0.94
Eggerthella	-0.44	0.62	0.94	-0.41	0.64	1.00	-0.04	0.96	0.99	-0.18	0.84	0.98	-0.08	0.93	0.99
Eisenbergiella	-0.45	0.64	0.94	-0.41	0.67	1.00	-0.38	0.69	0.94	-0.67	0.49	0.92	-0.47	0.63	0.93
Erysipelatoclostridium	-1.10	0.25	0.93	-0.92	0.33	0.87	-0.81	0.41	0.94	-1.04	0.29	0.92	-1.11	0.26	0.93
Erysipelotrichaceae_UCG.003	-0.26	0.71	0.97	-0.37	0.59	1.00	-0.51	0.49	0.94	-0.54	0.46	0.92	-0.48	0.51	0.93
Escherichia.Shigella	-0.76	0.23	0.93	-0.72	0.24	0.87	-0.37	0.57	0.94	-0.34	0.60	0.92	-0.41	0.53	0.93
Faecalibacterium	0.26	0.75	0.98	0.21	0.80	1.00	-0.12	0.89	0.96	-0.09	0.91	0.98	-0.08	0.93	0.99
Family_XIII_AD3011_group	0.46	0.59	0.94	0.18	0.83	1.00	-0.61	0.49	0.94	-0.78	0.38	0.92	-0.68	0.44	0.93
Family_XIII_UCG.001	0.56	0.59	0.94	0.25	0.80	1.00	-0.46	0.67	0.94	-0.70	0.51	0.92	-0.65	0.54	0.93
FD2005	-0.07	0.95	0.99	-0.30	0.78	1.00	-0.36	0.74	0.94	-0.17	0.88	0.98	-0.22	0.84	0.97
Flavonifractor	-0.94	0.30	0.93	-0.78	0.38	0.87	-0.29	0.75	0.94	-0.27	0.77	0.95	-0.32	0.73	0.94
Fournierella	-0.31	0.74	0.98	-0.28	0.76	1.00	-1.02	0.29	0.94	-1.12	0.25	0.92	-1.02	0.29	0.93
Frisingicoccus	0.00	0.99	0.99	-0.07	0.92	1.00	-0.42	0.56	0.94	-0.47	0.51	0.92	-0.30	0.67	0.93
Fusicatenibacter	0.32	0.66	0.94	0.12	0.87	1.00	0.09	0.90	0.96	0.19	0.80	0.96	0.30	0.68	0.93
Fusobacterium	1.52	0.21	0.93	1.56	0.19	0.87	2.44	0.04	0.82	2.32	0.06	0.82	2.07	0.09	0.92
GCA.900066575	0.54	0.56	0.94	0.20	0.83	1.00	-0.11	0.91	0.96	-0.28	0.77	0.95	-0.47	0.62	0.93
GCA.900066755	-1.07	0.48	0.94	-1.33	0.37	0.87	-0.75	0.64	0.94	-0.95	0.55	0.92	-1.12	0.48	0.93
Gordonibacter	0.08	0.93	0.99	0.11	0.90	1.00	-0.20	0.83	0.94	-0.41	0.66	0.92	-0.42	0.66	0.93
Granulicatella	0.03	0.98	0.99	-0.29	0.84	1.00	0.21	0.89	0.96	0.10	0.95	0.98	0.29	0.84	0.97
Haemophilus	0.55	0.44	0.94	0.36	0.61	1.00	0.12	0.87	0.96	0.02	0.98	0.98	0.08	0.92	0.99
Harryflintia	-0.48	0.67	0.94	-0.62	0.57	1.00	-0.84	0.46	0.94	-0.90	0.42	0.92	-1.21	0.28	0.93
Holdemanella	-0.26	0.74	0.98	-0.25	0.74	1.00	-0.23	0.77	0.94	-0.10	0.90	0.98	-0.05	0.95	0.99

© 2022 Meyer K et al. *JAMA Network Open.*

Holdemania	1.83	0.26	0.93	1.73	0.27	0.87	1.56	0.34	0.94	1.35	0.41	0.92	0.87	0.59	0.93
Hungatella	-1.31	0.14	0.89	-1.10	0.20	0.87	-0.86	0.34	0.94	-0.96	0.29	0.92	-0.71	0.43	0.93
Hydrogenoanaerobacterium	0.92	0.31	0.93	0.62	0.49	1.00	0.21	0.82	0.94	0.03	0.97	0.98	0.07	0.94	0.99
Incertae_Sedis	0.97	0.54	0.94	1.32	0.39	0.87	1.01	0.53	0.94	0.83	0.60	0.92	0.75	0.64	0.93
Intestinibacter	0.42	0.60	0.94	0.78	0.32	0.87	0.52	0.53	0.94	0.58	0.48	0.92	0.63	0.45	0.93
Intestinimonas	0.19	0.81	0.99	-0.08	0.91	1.00	-0.35	0.66	0.94	-0.52	0.52	0.92	-0.33	0.68	0.93
Lachnoclostridium	-2.43	0.15	0.89	-1.88	0.26	0.87	-0.12	0.95	0.99	0.07	0.97	0.98	0.03	0.99	0.99
Lachnospira	1.53	0.10	0.89	0.90	0.33	0.87	0.58	0.54	0.94	0.51	0.58	0.92	0.53	0.57	0.93
Lachnospiraceae_FCS020_group	0.66	0.43	0.94	0.21	0.80	1.00	-0.28	0.75	0.94	-0.35	0.70	0.92	-0.33	0.71	0.94
Lachnospiraceae_ND3007_group	0.33	0.57	0.94	-0.02	0.98	1.00	-0.24	0.69	0.94	-0.23	0.70	0.92	-0.23	0.70	0.93
Lachnospiraceae_NK4A136_group	0.63	0.48	0.94	0.30	0.73	1.00	-0.72	0.44	0.94	-0.75	0.42	0.92	-0.62	0.50	0.93
Lachnospiraceae_UCG.001	-0.27	0.66	0.94	-0.36	0.55	1.00	-0.63	0.32	0.94	-0.63	0.31	0.92	-0.60	0.34	0.93
Lachnospiraceae_UCG.004	0.30	0.66	0.94	0.12	0.86	1.00	0.19	0.79	0.94	0.24	0.73	0.94	0.03	0.96	0.99
Lachnospiraceae_UCG.010	0.56	0.58	0.94	0.21	0.83	1.00	-0.12	0.91	0.96	-0.16	0.88	0.98	-0.20	0.84	0.97
Lactobacillus	-1.01	0.33	0.93	-0.44	0.67	1.00	-0.51	0.62	0.94	-0.41	0.70	0.92	-0.29	0.79	0.97
Lactococcus	-0.17	0.87	0.99	-0.12	0.91	1.00	0.23	0.83	0.94	0.05	0.96	0.98	-0.16	0.88	0.99
Marvinbryantia	-0.39	0.55	0.94	-0.59	0.36	0.87	-0.57	0.39	0.94	-0.67	0.31	0.92	-0.63	0.34	0.93
Megasphaera	0.03	0.96	0.99	0.27	0.71	1.00	0.84	0.27	0.94	0.95	0.21	0.92	0.86	0.26	0.93
Monoglobus	1.25	0.19	0.93	1.22	0.19	0.87	1.18	0.22	0.94	1.04	0.28	0.92	1.06	0.28	0.93
Moryella	0.33	0.76	0.98	-0.03	0.97	1.00	-0.60	0.59	0.94	-0.58	0.60	0.92	-0.58	0.60	0.93
Negativibacillus	-0.91	0.20	0.93	-0.95	0.17	0.87	-1.39	0.06	0.82	-1.30	0.08	0.82	-1.37	0.06	0.92
NK4A214_group	-0.39	0.47	0.94	-0.57	0.29	0.87	-0.94	0.10	0.82	-0.95	0.09	0.82	-0.88	0.12	0.92
Odoribacter	0.55	0.32	0.93	0.63	0.24	0.87	0.19	0.74	0.94	0.15	0.80	0.96	0.12	0.83	0.97
Oscillibacter	0.24	0.80	0.99	-0.06	0.95	1.00	-0.33	0.73	0.94	-0.41	0.67	0.92	-0.41	0.67	0.93
Oscillospira	0.85	0.55	0.94	1.13	0.41	0.91	1.37	0.34	0.94	1.27	0.38	0.92	1.23	0.39	0.93
Oxalobacter	0.53	0.56	0.94	-0.04	0.96	1.00	-0.69	0.46	0.94	-0.66	0.48	0.92	-0.51	0.59	0.93
Paludicola	0.74	0.42	0.94	0.15	0.86	1.00	-0.54	0.57	0.94	-0.84	0.39	0.92	-0.51	0.60	0.93
Parabacteroides	0.10	0.88	0.99	0.49	0.46	0.99	0.58	0.39	0.94	0.53	0.44	0.92	0.52	0.44	0.93
Paraprevotella	0.13	0.79	0.99	0.31	0.53	1.00	0.26	0.61	0.94	0.23	0.65	0.92	0.25	0.63	0.93
Parasutterella	0.56	0.27	0.93	0.53	0.28	0.87	0.27	0.60	0.94	0.30	0.57	0.92	0.22	0.68	0.93
Peptococcus	-1.44	0.13	0.89	-1.71	0.06	0.87	-2.36	0.01	0.82	-2.47	0.01	0.82	-2.32	0.02	0.92
Phascolarctobacterium	0.24	0.62	0.94	0.32	0.49	1.00	0.24	0.62	0.94	0.15	0.75	0.95	0.19	0.69	0.93
Phocea	0.02	0.99	0.99	0.01	1.00	1.00	0.05	0.97	0.99	0.20	0.87	0.98	-0.08	0.95	0.99
Prevotella	-0.22	0.65	0.94	0.03	0.95	1.00	-0.25	0.61	0.94	-0.17	0.73	0.94	-0.12	0.80	0.97
Romboutsia	1.40	0.06	0.89	1.51	0.04	0.87	1.21	0.11	0.82	1.12	0.14	0.82	1.08	0.16	0.93
Roseburia	0.45	0.65	0.94	0.28	0.77	1.00	0.02	0.98	0.99	0.09	0.93	0.98	0.09	0.93	0.99
Ruminococcus	-0.30	0.62	0.94	-0.52	0.39	0.87	-1.00	0.11	0.82	-1.03	0.10	0.82	-0.92	0.14	0.93
Sellimonas	1.29	0.12	0.89	1.07	0.19	0.87	0.93	0.28	0.94	0.91	0.29	0.92	0.88	0.30	0.93
Shuttleworthia	-0.55	0.49	0.94	-1.05	0.18	0.87	-1.32	0.11	0.82	-1.34	0.11	0.82	-1.33	0.11	0.92
Slackia	-0.57	0.44	0.94	-0.34	0.64	1.00	-0.56	0.46	0.94	-0.54	0.48	0.92	-0.63	0.40	0.93
Streptococcus	-1.68	0.11	0.89	-1.65	0.11	0.87	-1.24	0.26	0.94	-1.62	0.14	0.82	-1.54	0.16	0.93
Subdoligranulum	0.24	0.75	0.98	0.01	0.99	1.00	-0.57	0.46	0.94	-0.59	0.44	0.92	-0.61	0.42	0.93
Sutterella	-0.20	0.66	0.94	0.00	1.00	1.00	0.01	0.99	0.99	-0.02	0.97	0.98	-0.06	0.90	0.99
TM7x	-1.54	0.14	0.89	-2.03	0.05	0.87	-1.79	0.09	0.82	-1.95	0.07	0.82	-1.85	0.08	0.92
Turicibacter	1.59	0.11	0.89	1.80	0.06	0.87	1.55	0.11	0.82	1.45	0.14	0.82	1.20	0.22	0.93
Tuzzerella	-1.08	0.11	0.89	-0.88	0.19	0.87	-0.83	0.24	0.94	-0.82	0.25	0.92	-0.92	0.19	0.93
Tyzzerella	0.86	0.37	0.94	0.85	0.36	0.87	1.35	0.18	0.94	1.51	0.13	0.82	1.82	0.07	0.92
UBA1819	0.25	0.83	0.99	0.27	0.82	1.00	0.31	0.80	0.94	0.09	0.94	0.98	-0.02	0.99	0.99
UCG.002	-0.35	0.52	0.94	-0.53	0.32	0.87	-1.24	0.03	0.82	-1.31	0.02	0.82	-1.33	0.02	0.92
UCG.003	0.51	0.39	0.94	0.15	0.80	1.00	-0.24	0.70	0.94	-0.25	0.69	0.92	-0.25	0.69	0.93
UCG.005	0.11	0.87	0.99	0.04	0.96	1.00	-0.70	0.35	0.94	-0.83	0.26	0.92	-0.71	0.34	0.93
UCG.009	0.01	0.99	0.99	-0.45	0.65	1.00	-1.03	0.31	0.94	-1.24	0.22	0.92	-1.14	0.26	0.93
Veillonella	0.82	0.29	0.93	0.69	0.36	0.87	0.81	0.30	0.94	0.75	0.33	0.92	0.82	0.29	0.93
Victivallis	1.24	0.09	0.89	0.96	0.18	0.87	0.25	0.74	0.94	0.14	0.85	0.98	0.25	0.74	0.94

Multivariable-adjusted regression models adjusted for:
Model 1: sequencing batch, age, race, gender, study center
Model 2: Model 1, with education
Model 3: Model 2, with physical activity, diet score, medication use, and smoking

© 2022 Meyer K et al. *JAMA Network Open.*

Model 4: Model 3 with BMI
Model 5: Model 4 with hypertension and diabetes

© 2022 Meyer K et al. *JAMA Network Open.*

eTable 38: Multivariable-adjusted regression coefficients, p-values, and false discovery rate (FDR)-adjusted p-values for genera among White participants with respect to MOCA.

	Model1			Model2			Model3			Model4			Model5		
	beta	p-value	FDR	beta	p-value	FDR	beta	p-value	FDR	beta	p-value	FDR	beta	p-value	FDR
Acetanaerobacterium	-0.41	0.23	0.60	-0.16	0.61	0.87	-0.29	0.39	0.84	-0.33	0.33	0.77	-0.42	0.21	0.72
Acidaminococcus	-0.37	0.00	0.08	-0.25	0.03	0.44	-0.28	0.02	0.66	-0.28	0.02	0.74	-0.28	0.02	0.72
Actinomyces	0.33	0.32	0.62	0.35	0.27	0.77	0.48	0.15	0.79	0.47	0.15	0.74	0.49	0.13	0.72
Adlercreutzia	-0.18	0.32	0.62	-0.15	0.38	0.77	-0.18	0.32	0.81	-0.18	0.30	0.74	-0.20	0.27	0.72
Agathobacter	0.00	1.00	1.00	-0.01	0.97	0.98	-0.07	0.75	0.95	-0.02	0.92	0.98	-0.01	0.95	0.98
Akkermansia	0.07	0.54	0.77	0.03	0.76	0.92	0.02	0.83	0.95	0.02	0.86	0.98	0.01	0.93	0.98
Alistipes	0.19	0.30	0.62	0.16	0.36	0.77	0.06	0.73	0.95	0.05	0.77	0.98	0.04	0.83	0.98
Anaerofilum	-0.03	0.91	0.93	-0.23	0.33	0.77	-0.25	0.31	0.81	-0.27	0.27	0.74	-0.28	0.25	0.72
Anaerostipes	0.32	0.31	0.62	0.24	0.41	0.77	0.21	0.50	0.86	0.15	0.62	0.98	0.15	0.63	0.98
Anaerotruncus	-0.19	0.43	0.71	-0.25	0.28	0.77	-0.32	0.19	0.79	-0.39	0.11	0.74	-0.39	0.12	0.72
Angelakisella	0.24	0.50	0.74	0.04	0.91	0.96	0.05	0.88	0.95	0.07	0.85	0.98	0.00	0.99	0.99
Bacteroides	-0.55	0.32	0.62	-0.58	0.26	0.77	-0.65	0.23	0.80	-0.64	0.24	0.74	-0.65	0.23	0.72
Barnesiella	0.33	0.00	0.08	0.31	0.00	0.13	0.30	0.00	0.24	0.30	0.00	0.35	0.30	0.00	0.22
Bifidobacterium	-0.17	0.21	0.60	-0.04	0.79	0.92	-0.05	0.72	0.95	-0.06	0.68	0.98	-0.07	0.62	0.98
Bilophila	0.04	0.80	0.92	0.05	0.71	0.89	0.05	0.75	0.95	0.03	0.82	0.98	0.02	0.87	0.98
Blautia	0.13	0.81	0.92	-0.11	0.83	0.93	-0.12	0.82	0.95	-0.25	0.65	0.98	-0.15	0.78	0.98
Butyricicoccus	0.35	0.35	0.62	0.14	0.68	0.88	-0.02	0.95	0.97	-0.03	0.94	0.98	-0.02	0.95	0.98
Butyricimonas	0.12	0.35	0.62	0.07	0.56	0.84	0.03	0.83	0.95	0.03	0.78	0.98	0.03	0.80	0.98
CAG.56	0.32	0.03	0.30	0.22	0.12	0.74	0.19	0.20	0.79	0.18	0.23	0.74	0.20	0.18	0.72
Candidatus_Soleaferrea	-0.43	0.14	0.51	-0.56	0.04	0.52	-0.52	0.07	0.79	-0.53	0.07	0.74	-0.56	0.05	0.72
Caproiciproducens	-0.26	0.29	0.62	-0.20	0.38	0.77	-0.26	0.29	0.81	-0.27	0.26	0.74	-0.28	0.24	0.72
Christensenellaceae_R.7_group	0.24	0.05	0.34	0.20	0.09	0.71	0.17	0.17	0.79	0.15	0.22	0.74	0.15	0.24	0.72
Clostridium_sensu_stricto_1	0.29	0.05	0.34	0.30	0.03	0.44	0.31	0.03	0.66	0.29	0.04	0.74	0.25	0.09	0.72
Colidextribacter	-0.13	0.70	0.87	-0.35	0.27	0.77	-0.36	0.30	0.81	-0.40	0.26	0.74	-0.43	0.22	0.72
Collinsella	0.03	0.86	0.92	0.14	0.30	0.77	0.12	0.39	0.84	0.14	0.30	0.74	0.12	0.39	0.81
Coprobacter	-0.03	0.83	0.92	-0.11	0.43	0.78	-0.20	0.16	0.79	-0.20	0.15	0.74	-0.20	0.16	0.72
Coprococcus	0.19	0.26	0.62	0.12	0.45	0.81	0.05	0.76	0.95	0.04	0.82	0.98	0.01	0.95	0.98
Defluviitaleaceae_UCG.011	0.09	0.68	0.86	0.03	0.90	0.96	0.03	0.91	0.95	-0.01	0.97	0.98	-0.03	0.89	0.98
Desulfovibrio	-0.20	0.17	0.51	-0.19	0.16	0.77	-0.20	0.16	0.79	-0.22	0.12	0.74	-0.20	0.16	0.72
Dialister	0.12	0.27	0.62	0.10	0.29	0.77	0.08	0.47	0.85	0.09	0.38	0.79	0.07	0.51	0.92
DNF00809	0.55	0.03	0.30	0.46	0.06	0.57	0.45	0.08	0.79	0.43	0.10	0.74	0.42	0.10	0.72
Dorea	-0.12	0.62	0.82	-0.04	0.85	0.94	-0.08	0.71	0.95	-0.08	0.71	0.98	-0.04	0.85	0.98
DTU089	-0.04	0.86	0.92	-0.02	0.94	0.97	0.03	0.87	0.95	-0.01	0.97	0.98	0.00	0.99	0.99
Eggerthella	-0.14	0.49	0.74	-0.13	0.50	0.83	-0.16	0.43	0.85	-0.20	0.34	0.77	-0.17	0.41	0.81
Eisenbergiella	-0.12	0.58	0.80	-0.11	0.62	0.87	-0.11	0.63	0.95	-0.17	0.43	0.79	-0.15	0.50	0.92
Erysipelatoclostridium	-0.47	0.04	0.30	-0.40	0.06	0.57	-0.34	0.13	0.79	-0.39	0.08	0.74	-0.43	0.05	0.72
Erysipelotrichaceae_UCG.003	0.03	0.84	0.92	-0.01	0.96	0.98	0.03	0.88	0.95	0.02	0.91	0.98	-0.01	0.95	0.98
Escherichia.Shigella	-0.20	0.17	0.51	-0.18	0.18	0.77	-0.18	0.23	0.80	-0.17	0.25	0.74	-0.15	0.32	0.80
Faecalibacterium	0.21	0.26	0.62	0.20	0.28	0.77	0.21	0.30	0.81	0.21	0.28	0.74	0.18	0.35	0.81
Family_XIII_AD3011_group	0.27	0.17	0.51	0.16	0.38	0.77	0.06	0.75	0.95	0.03	0.89	0.98	0.02	0.94	0.98
Family_XIII_UCG.001	0.23	0.35	0.62	0.11	0.63	0.87	0.11	0.66	0.95	0.05	0.82	0.98	0.03	0.89	0.98
FD2005	-0.04	0.89	0.92	-0.13	0.59	0.86	-0.06	0.82	0.95	-0.01	0.97	0.98	-0.05	0.84	0.98
Flavonifractor	-0.30	0.16	0.51	-0.23	0.24	0.77	-0.18	0.40	0.84	-0.17	0.41	0.79	-0.18	0.39	0.81
Fournierella	0.11	0.60	0.82	0.13	0.53	0.83	0.12	0.59	0.95	0.10	0.65	0.98	0.09	0.67	0.98
Frisingicoccus	-0.03	0.85	0.92	-0.06	0.71	0.89	-0.12	0.48	0.85	-0.13	0.43	0.79	-0.10	0.53	0.92
Fusicatenibacter	0.12	0.50	0.74	0.04	0.82	0.93	0.00	0.98	0.98	0.03	0.87	0.98	0.02	0.92	0.98
Fusobacterium	-0.03	0.91	0.93	-0.01	0.96	0.98	0.11	0.70	0.95	0.07	0.79	0.98	0.07	0.81	0.98
GCA.900066575	0.19	0.39	0.66	0.05	0.79	0.92	0.04	0.87	0.95	0.00	0.98	0.98	-0.05	0.82	0.98
GCA.900066755	-0.13	0.72	0.87	-0.23	0.50	0.83	-0.25	0.49	0.85	-0.30	0.41	0.79	-0.31	0.40	0.81
Gordonibacter	-0.19	0.38	0.66	-0.18	0.39	0.77	-0.20	0.34	0.82	-0.26	0.23	0.74	-0.24	0.28	0.72
Granulicatella	-0.23	0.49	0.74	-0.35	0.28	0.77	-0.19	0.58	0.95	-0.22	0.52	0.89	-0.19	0.56	0.96
Haemophilus	0.31	0.07	0.37	0.23	0.15	0.77	0.25	0.13	0.79	0.23	0.17	0.74	0.23	0.17	0.72
Harryflintia	-0.10	0.70	0.87	-0.15	0.53	0.83	-0.13	0.62	0.95	-0.14	0.58	0.95	-0.18	0.49	0.92
Holdemanella	0.05	0.78	0.92	0.05	0.76	0.92	0.02	0.90	0.95	0.06	0.76	0.98	0.07	0.71	0.98

© 2022 Meyer K et al. *JAMA Network Open.*

Holdemania	-0.26	0.50	0.74	-0.30	0.40	0.77	-0.37	0.32	0.81	-0.42	0.26	0.74	-0.51	0.17	0.72
Hungatella	-0.51	0.01	0.22	-0.42	0.03	0.44	-0.31	0.13	0.79	-0.33	0.10	0.74	-0.32	0.12	0.72
Hydrogenoanaerobacterium	0.21	0.33	0.62	0.09	0.65	0.87	0.01	0.95	0.97	-0.03	0.89	0.98	-0.03	0.91	0.98
Incertae_Sedis	-0.30	0.42	0.70	-0.16	0.64	0.87	-0.09	0.81	0.95	-0.13	0.72	0.98	-0.07	0.85	0.98
Intestinibacter	0.19	0.32	0.62	0.33	0.06	0.57	0.36	0.05	0.66	0.37	0.05	0.74	0.36	0.06	0.72
Intestinimonas	0.19	0.29	0.62	0.09	0.63	0.87	0.08	0.67	0.95	0.04	0.83	0.98	0.07	0.71	0.98
Lachnoclostridium	-1.09	0.01	0.14	-0.88	0.02	0.44	-0.78	0.05	0.66	-0.74	0.07	0.74	-0.67	0.10	0.72
Lachnospira	0.69	0.00	0.08	0.44	0.03	0.44	0.43	0.04	0.66	0.42	0.05	0.74	0.40	0.06	0.72
Lachnospiraceae_FCS020_group	0.39	0.05	0.34	0.21	0.25	0.77	0.21	0.31	0.81	0.19	0.34	0.77	0.18	0.38	0.81
Lachnospiraceae_ND3007_group	0.17	0.22	0.60	0.03	0.81	0.93	-0.01	0.94	0.97	-0.01	0.96	0.98	-0.02	0.88	0.98
Lachnospiraceae_NK4A136_grou	0.16	0.45	0.72	0.03	0.89	0.96	-0.11	0.59	0.95	-0.12	0.57	0.95	-0.13	0.53	0.92
Lachnospiraceae_UCG.001	0.15	0.29	0.62	0.12	0.37	0.77	0.05	0.71	0.95	0.05	0.71	0.98	0.05	0.72	0.98
Lachnospiraceae_UCG.004	0.14	0.40	0.67	0.07	0.66	0.87	0.05	0.75	0.95	0.06	0.70	0.98	0.03	0.86	0.98
Lachnospiraceae_UCG.010	-0.09	0.71	0.87	-0.23	0.31	0.77	-0.30	0.21	0.79	-0.31	0.19	0.74	-0.29	0.23	0.72
Lactobacillus	-0.36	0.13	0.51	-0.14	0.55	0.84	-0.06	0.80	0.95	-0.03	0.88	0.98	-0.02	0.94	0.98
Lactococcus	-0.05	0.84	0.92	-0.03	0.91	0.96	0.10	0.66	0.95	0.06	0.79	0.98	0.06	0.80	0.98
Marvinbryantia	0.07	0.63	0.82	0.00	0.99	0.99	-0.01	0.97	0.98	-0.03	0.85	0.98	-0.04	0.79	0.98
Megasphaera	-0.25	0.14	0.51	-0.16	0.32	0.77	-0.05	0.76	0.95	-0.03	0.87	0.98	-0.04	0.83	0.98
Monoglobus	0.55	0.01	0.22	0.53	0.01	0.44	0.62	0.00	0.24	0.60	0.01	0.35	0.63	0.00	0.22
Moryella	0.54	0.03	0.30	0.39	0.10	0.71	0.30	0.24	0.80	0.31	0.23	0.74	0.30	0.23	0.72
Negativibacillus	-0.23	0.18	0.51	-0.24	0.13	0.74	-0.21	0.21	0.79	-0.19	0.26	0.74	-0.20	0.23	0.72
NK4A214_group	0.18	0.15	0.51	0.11	0.34	0.77	0.06	0.63	0.95	0.06	0.64	0.98	0.04	0.75	0.98
Odoribacter	-0.06	0.62	0.82	-0.04	0.77	0.92	-0.09	0.47	0.85	-0.10	0.43	0.79	-0.11	0.39	0.81
Oscillibacter	0.16	0.47	0.74	0.04	0.84	0.94	-0.03	0.90	0.95	-0.04	0.84	0.98	-0.06	0.77	0.98
Oscillospira	0.12	0.72	0.87	0.23	0.46	0.81	0.36	0.27	0.81	0.34	0.30	0.74	0.35	0.29	0.74
Oxalobacter	0.46	0.03	0.30	0.24	0.22	0.77	0.21	0.33	0.81	0.22	0.31	0.74	0.25	0.25	0.72
Paludicola	0.40	0.06	0.35	0.18	0.39	0.77	0.16	0.47	0.85	0.10	0.67	0.98	0.12	0.60	0.98
Parabacteroides	-0.06	0.68	0.86	0.08	0.59	0.86	0.13	0.39	0.84	0.12	0.43	0.79	0.11	0.48	0.91
Paraprevotella	-0.02	0.88	0.92	0.05	0.66	0.87	0.01	0.91	0.95	0.01	0.96	0.98	0.01	0.96	0.98
Parasutterella	0.14	0.22	0.60	0.13	0.23	0.77	0.09	0.43	0.85	0.10	0.40	0.79	0.10	0.40	0.81
Peptococcus	-0.04	0.85	0.92	-0.14	0.49	0.83	-0.17	0.44	0.85	-0.20	0.38	0.79	-0.18	0.41	0.81
Phascolarctobacterium	-0.02	0.86	0.92	0.01	0.91	0.96	0.05	0.66	0.95	0.03	0.81	0.98	0.04	0.70	0.98
Phocea	-0.18	0.53	0.76	-0.19	0.49	0.83	-0.25	0.37	0.84	-0.22	0.44	0.79	-0.26	0.37	0.81
Prevotella	-0.20	0.07	0.36	-0.11	0.29	0.77	-0.14	0.19	0.79	-0.12	0.26	0.74	-0.12	0.26	0.72
Romboutsia	0.03	0.88	0.92	0.07	0.68	0.88	0.06	0.73	0.95	0.04	0.84	0.98	-0.01	0.94	0.98
Roseburia	0.34	0.14	0.51	0.27	0.21	0.77	0.29	0.19	0.79	0.31	0.16	0.74	0.27	0.23	0.72
Ruminococcus	0.20	0.15	0.51	0.12	0.36	0.77	0.10	0.49	0.85	0.09	0.51	0.89	0.08	0.59	0.98
Sellimonas	0.32	0.11	0.46	0.23	0.21	0.77	0.22	0.26	0.81	0.22	0.27	0.74	0.25	0.21	0.72
Shuttleworthia	0.12	0.50	0.74	-0.06	0.72	0.89	-0.19	0.31	0.81	-0.20	0.29	0.74	-0.18	0.35	0.81
Slackia	0.02	0.92	0.93	0.11	0.52	0.83	0.10	0.56	0.94	0.11	0.54	0.90	0.09	0.61	0.98
Streptococcus	0.29	0.23	0.60	0.30	0.19	0.77	0.51	0.04	0.66	0.44	0.08	0.74	0.45	0.08	0.72
Subdoligranulum	0.47	0.01	0.14	0.38	0.02	0.44	0.33	0.05	0.66	0.33	0.05	0.74	0.31	0.07	0.72
Sutterella	-0.17	0.10	0.46	-0.10	0.33	0.77	-0.10	0.32	0.81	-0.11	0.30	0.74	-0.12	0.23	0.72
TM7x	0.12	0.62	0.82	-0.06	0.79	0.92	-0.07	0.78	0.95	-0.10	0.67	0.98	-0.12	0.62	0.98
Turicibacter	0.27	0.25	0.62	0.35	0.11	0.73	0.37	0.09	0.79	0.35	0.12	0.74	0.30	0.18	0.72
Tuzzerella	-0.30	0.06	0.34	-0.23	0.13	0.74	-0.22	0.17	0.79	-0.22	0.17	0.74	-0.26	0.11	0.72
Tyzzerella	0.26	0.25	0.62	0.25	0.23	0.77	0.28	0.22	0.80	0.32	0.16	0.74	0.38	0.10	0.72
UBA1819	-0.17	0.55	0.77	-0.16	0.54	0.83	-0.19	0.49	0.85	-0.25	0.37	0.79	-0.24	0.40	0.81
UCG.002	0.22	0.08	0.38	0.15	0.19	0.77	0.10	0.44	0.85	0.09	0.51	0.89	0.06	0.63	0.98
UCG.003	0.23	0.10	0.46	0.09	0.51	0.83	0.04	0.80	0.95	0.03	0.81	0.98	0.02	0.89	0.98
UCG.005	0.16	0.34	0.62	0.12	0.42	0.78	0.08	0.65	0.95	0.05	0.78	0.98	0.05	0.76	0.98
UCG.009	0.55	0.02	0.28	0.37	0.10	0.71	0.30	0.19	0.79	0.26	0.26	0.74	0.26	0.27	0.72
Veillonella	0.26	0.15	0.51	0.21	0.21	0.77	0.24	0.18	0.79	0.23	0.20	0.74	0.21	0.23	0.72
Victivallis	0.33	0.05	0.34	0.22	0.17	0.77	0.16	0.35	0.82	0.13	0.44	0.79	0.13	0.45	0.87

Multivariable-adjusted regression models adjusted for:
Model 1: sequencing batch, age, race, gender, study center
Model 2: Model 1, with education
Model 3: Model 2, with physical activity, diet score, medication use, and smoking

© 2022 Meyer K et al. *JAMA Network Open.*

Model 4: Model 3 with BMI
Model 5: Model 4 with hypertension and diabetes

© 2022 Meyer K et al. *JAMA Network Open.*

eTable 39: Multivariable-adjusted regression coefficients, p-values, and false discovery rate (FDR)-adjusted p-values for genera among White participants with respect to PC (1st).

	Model1			Model2			Model3			Model4			Model5		
	beta	p-value	FDR	beta	p-value	FDR	beta	p-value	FDR	beta	p-value	FDR	beta	p-value	FDR
Acetanaerobacterium	-0.06	0.73	0.85	0.08	0.67	0.91	0.00	0.98	0.98	-0.02	0.90	0.95	-0.06	0.74	0.92
Acidaminococcus	-0.22	0.00	0.04	-0.15	0.02	0.22	-0.15	0.02	0.41	-0.15	0.02	0.43	-0.15	0.02	0.39
Actinomyces	0.09	0.64	0.84	0.09	0.59	0.88	0.20	0.26	0.78	0.19	0.27	0.79	0.21	0.24	0.82
Adlercreutzia	-0.01	0.93	0.96	0.01	0.93	0.96	-0.04	0.69	0.94	-0.04	0.66	0.92	-0.04	0.66	0.92
Agathobacter	0.06	0.62	0.84	0.06	0.63	0.88	-0.01	0.92	0.96	0.01	0.90	0.95	0.02	0.87	0.94
Akkermansia	0.04	0.51	0.75	0.02	0.72	0.91	0.01	0.91	0.96	0.00	0.94	0.96	0.00	0.94	0.98
Alistipes	0.14	0.16	0.45	0.13	0.18	0.55	0.03	0.73	0.94	0.03	0.77	0.92	0.03	0.78	0.92
Anaerofilum	0.10	0.46	0.71	-0.01	0.96	0.97	-0.03	0.80	0.94	-0.04	0.74	0.92	-0.05	0.73	0.92
Anaerostipes	-0.01	0.96	0.96	-0.05	0.77	0.91	-0.08	0.64	0.94	-0.11	0.52	0.89	-0.10	0.55	0.91
Anaerotruncus	-0.10	0.43	0.70	-0.14	0.27	0.73	-0.19	0.14	0.70	-0.23	0.07	0.63	-0.23	0.08	0.68
Angelakisella	0.19	0.31	0.57	0.09	0.62	0.88	0.02	0.93	0.96	0.02	0.90	0.95	-0.01	0.95	0.98
Bacteroides	-0.38	0.20	0.48	-0.40	0.15	0.49	-0.36	0.21	0.74	-0.36	0.22	0.75	-0.38	0.19	0.78
Barnesiella	0.21	0.00	0.01	0.21	0.00	0.01	0.18	0.00	0.07	0.18	0.00	0.09	0.18	0.00	0.09
Bifidobacterium	-0.10	0.20	0.48	-0.02	0.74	0.91	-0.03	0.68	0.94	-0.03	0.64	0.92	-0.04	0.61	0.92
Bilophila	0.11	0.17	0.47	0.12	0.13	0.44	0.11	0.15	0.70	0.10	0.18	0.75	0.10	0.20	0.79
Blautia	-0.15	0.62	0.84	-0.28	0.31	0.74	-0.24	0.41	0.86	-0.30	0.29	0.79	-0.27	0.35	0.83
Butyricicoccus	0.02	0.93	0.96	-0.10	0.61	0.88	-0.23	0.23	0.77	-0.24	0.23	0.77	-0.21	0.28	0.83
Butyricimonas	0.05	0.44	0.70	0.03	0.69	0.91	0.00	0.98	0.98	0.00	0.96	0.97	0.00	0.98	0.98
CAG.56	0.21	0.01	0.10	0.15	0.05	0.35	0.12	0.13	0.70	0.11	0.15	0.75	0.12	0.13	0.71
Candidatus_Soleaferrea	-0.25	0.12	0.39	-0.32	0.03	0.31	-0.27	0.08	0.69	-0.28	0.07	0.63	-0.30	0.06	0.60
Caproiciproducens	-0.06	0.65	0.84	-0.03	0.83	0.94	-0.08	0.53	0.92	-0.09	0.48	0.87	-0.09	0.48	0.88
Christensenellaceae_R.7_group	0.13	0.04	0.24	0.11	0.08	0.37	0.06	0.36	0.85	0.05	0.45	0.86	0.05	0.42	0.87
Clostridium_sensu_stricto_1	0.13	0.11	0.38	0.14	0.07	0.37	0.14	0.08	0.69	0.12	0.11	0.67	0.11	0.18	0.78
Colidextribacter	-0.16	0.40	0.67	-0.28	0.11	0.42	-0.34	0.07	0.69	-0.36	0.05	0.61	-0.37	0.05	0.57
Collinsella	-0.02	0.76	0.86	0.04	0.62	0.88	0.04	0.57	0.94	0.05	0.46	0.87	0.05	0.49	0.88
Coprobacter	0.03	0.68	0.85	-0.01	0.88	0.94	-0.07	0.35	0.85	-0.07	0.32	0.79	-0.07	0.34	0.83
Coprococcus	0.19	0.03	0.22	0.16	0.07	0.37	0.10	0.26	0.78	0.09	0.29	0.79	0.08	0.34	0.83
Defluviitaleaceae_UCG.011	0.18	0.14	0.43	0.15	0.21	0.61	0.11	0.36	0.85	0.09	0.44	0.86	0.09	0.46	0.88
Desulfovibrio	-0.09	0.24	0.49	-0.09	0.24	0.67	-0.12	0.13	0.70	-0.13	0.10	0.63	-0.12	0.11	0.70
Dialister	0.04	0.45	0.70	0.04	0.50	0.87	0.03	0.57	0.94	0.04	0.47	0.87	0.03	0.58	0.92
DNF00809	0.17	0.22	0.49	0.12	0.36	0.74	0.08	0.58	0.94	0.07	0.63	0.92	0.06	0.66	0.92
Dorea	-0.06	0.64	0.84	-0.02	0.87	0.94	-0.06	0.60	0.94	-0.06	0.60	0.92	-0.05	0.68	0.92
DTU089	-0.08	0.51	0.75	-0.06	0.57	0.88	-0.08	0.49	0.92	-0.10	0.37	0.79	-0.09	0.42	0.87
Eggerthella	-0.04	0.73	0.85	-0.03	0.76	0.91	-0.03	0.78	0.94	-0.05	0.66	0.92	-0.04	0.74	0.92
Eisenbergiella	-0.11	0.38	0.66	-0.10	0.41	0.78	-0.10	0.39	0.86	-0.14	0.24	0.79	-0.12	0.31	0.83
Erysipelatoclostridium	-0.25	0.04	0.23	-0.21	0.06	0.37	-0.15	0.19	0.74	-0.18	0.12	0.70	-0.20	0.10	0.70
Erysipelotrichaceae_UCG.003	0.07	0.43	0.70	0.05	0.57	0.88	0.03	0.72	0.94	0.03	0.75	0.92	0.03	0.78	0.92
Escherichia.Shigella	-0.08	0.31	0.57	-0.07	0.35	0.74	-0.03	0.72	0.94	-0.02	0.76	0.92	-0.02	0.79	0.92
Faecalibacterium	0.17	0.10	0.38	0.16	0.10	0.41	0.13	0.21	0.74	0.13	0.20	0.75	0.13	0.22	0.79
Family_XIII_AD3011_group	0.15	0.16	0.45	0.09	0.36	0.74	-0.02	0.88	0.95	-0.04	0.73	0.92	-0.03	0.76	0.92
Family_XIII_UCG.001	0.15	0.25	0.49	0.09	0.46	0.84	0.04	0.76	0.94	0.01	0.94	0.96	0.01	0.94	0.98
FD2005	0.09	0.53	0.77	0.04	0.78	0.91	0.06	0.64	0.94	0.09	0.51	0.88	0.08	0.57	0.91
Flavonifractor	-0.23	0.05	0.24	-0.20	0.07	0.37	-0.15	0.19	0.74	-0.15	0.20	0.75	-0.15	0.18	0.78
Fournierella	-0.01	0.93	0.96	0.00	0.98	0.98	-0.04	0.77	0.94	-0.05	0.69	0.92	-0.04	0.72	0.92
Frisingicoccus	-0.08	0.37	0.66	-0.10	0.26	0.72	-0.15	0.08	0.69	-0.16	0.07	0.63	-0.14	0.10	0.70
Fusicatenibacter	0.12	0.18	0.47	0.08	0.36	0.74	0.04	0.67	0.94	0.05	0.57	0.91	0.06	0.54	0.91
Fusobacterium	-0.04	0.80	0.88	-0.03	0.84	0.94	0.10	0.48	0.92	0.09	0.55	0.91	0.07	0.63	0.92
GCA.900066575	0.09	0.43	0.70	0.02	0.86	0.94	-0.02	0.84	0.95	-0.05	0.69	0.92	-0.07	0.55	0.91
GCA.900066755	0.09	0.64	0.84	0.04	0.83	0.94	0.11	0.58	0.94	0.08	0.66	0.92	0.07	0.71	0.92
Gordonibacter	-0.01	0.91	0.96	-0.01	0.96	0.97	-0.06	0.62	0.94	-0.08	0.47	0.87	-0.08	0.50	0.88
Granulicatella	0.07	0.72	0.85	0.01	0.96	0.97	0.09	0.60	0.94	0.08	0.66	0.92	0.10	0.59	0.92
Haemophilus	0.18	0.05	0.24	0.14	0.10	0.41	0.13	0.16	0.70	0.11	0.20	0.75	0.12	0.19	0.78
Harryflintia	-0.06	0.67	0.85	-0.09	0.50	0.87	-0.07	0.61	0.94	-0.08	0.57	0.91	-0.10	0.45	0.88
Holdemanella	0.01	0.92	0.96	0.01	0.91	0.96	-0.02	0.80	0.94	-0.01	0.94	0.96	0.00	0.98	0.98

© 2022 Meyer K et al. *JAMA Network Open.*

Holdemania	0.07	0.73	0.85	0.05	0.78	0.91	0.03	0.87	0.95	0.01	0.98	0.98	-0.04	0.82	0.93
Hungatella	-0.31	0.01	0.08	-0.26	0.01	0.20	-0.17	0.11	0.70	-0.19	0.09	0.63	-0.17	0.12	0.70
Hydrogenoanaerobacterium	0.22	0.06	0.26	0.16	0.15	0.48	0.07	0.53	0.92	0.05	0.66	0.92	0.05	0.65	0.92
Incertae_Sedis	0.09	0.64	0.84	0.17	0.37	0.74	0.15	0.43	0.87	0.13	0.50	0.88	0.14	0.47	0.88
Intestinibacter	0.13	0.20	0.48	0.21	0.03	0.31	0.20	0.04	0.63	0.21	0.03	0.46	0.21	0.04	0.49
Intestinimonas	0.11	0.27	0.53	0.05	0.57	0.88	0.03	0.74	0.94	0.01	0.89	0.95	0.03	0.75	0.92
Lachnoclostridium	-0.56	0.01	0.10	-0.45	0.03	0.31	-0.21	0.33	0.85	-0.19	0.38	0.79	-0.18	0.42	0.87
Lachnospira	0.51	0.00	0.00	0.38	0.00	0.03	0.36	0.00	0.07	0.36	0.00	0.09	0.35	0.00	0.09
Lachnospiraceae_FCS020_group	0.30	0.00	0.08	0.21	0.04	0.34	0.14	0.19	0.74	0.14	0.21	0.75	0.14	0.22	0.79
Lachnospiraceae_ND3007_group	0.14	0.06	0.29	0.06	0.37	0.74	0.01	0.84	0.95	0.02	0.82	0.95	0.01	0.85	0.94
Lachnospiraceae_NK4A136_group	0.12	0.29	0.56	0.05	0.66	0.91	-0.10	0.39	0.86	-0.10	0.37	0.79	-0.10	0.39	0.87
Lachnospiraceae_UCG.001	0.13	0.11	0.38	0.11	0.14	0.48	0.07	0.35	0.85	0.07	0.36	0.79	0.07	0.34	0.83
Lachnospiraceae_UCG.004	0.06	0.48	0.73	0.02	0.78	0.91	0.01	0.91	0.96	0.02	0.85	0.95	0.00	0.96	0.98
Lachnospiraceae_UCG.010	-0.04	0.77	0.87	-0.11	0.35	0.74	-0.17	0.18	0.74	-0.17	0.17	0.75	-0.17	0.17	0.78
Lactobacillus	-0.32	0.01	0.12	-0.20	0.11	0.41	-0.19	0.14	0.70	-0.17	0.17	0.75	-0.17	0.19	0.78
Lactococcus	-0.05	0.69	0.85	-0.04	0.74	0.91	0.00	0.98	0.98	-0.02	0.88	0.95	-0.03	0.80	0.92
Marvinbryantia	0.02	0.80	0.88	-0.02	0.78	0.91	-0.03	0.71	0.94	-0.04	0.60	0.92	-0.04	0.60	0.92
Megasphaera	-0.11	0.24	0.49	-0.06	0.49	0.87	0.01	0.88	0.95	0.03	0.76	0.92	0.02	0.83	0.93
Monoglobus	0.33	0.01	0.08	0.32	0.00	0.15	0.33	0.00	0.15	0.32	0.01	0.23	0.33	0.00	0.18
Moryella	0.31	0.02	0.19	0.23	0.07	0.37	0.14	0.32	0.85	0.14	0.31	0.79	0.14	0.31	0.83
Negativibacillus	-0.15	0.09	0.36	-0.16	0.06	0.37	-0.17	0.06	0.69	-0.16	0.08	0.63	-0.16	0.07	0.60
NK4A214_group	0.09	0.20	0.48	0.05	0.43	0.82	-0.01	0.88	0.95	-0.01	0.87	0.95	-0.01	0.88	0.94
Odoribacter	0.02	0.74	0.85	0.04	0.56	0.88	-0.02	0.73	0.94	-0.03	0.67	0.92	-0.03	0.64	0.92
Oscillibacter	0.18	0.12	0.39	0.12	0.28	0.73	0.05	0.65	0.94	0.04	0.71	0.92	0.04	0.74	0.92
Oscillospira	-0.01	0.95	0.96	0.05	0.76	0.91	0.11	0.51	0.92	0.10	0.56	0.91	0.10	0.56	0.91
Oxalobacter	0.29	0.01	0.11	0.17	0.12	0.44	0.10	0.36	0.85	0.11	0.34	0.79	0.13	0.27	0.83
Paludicola	0.15	0.21	0.48	0.02	0.85	0.94	-0.08	0.52	0.92	-0.11	0.33	0.79	-0.09	0.45	0.88
Parabacteroides	-0.03	0.74	0.85	0.05	0.53	0.88	0.09	0.27	0.78	0.09	0.30	0.79	0.08	0.32	0.83
Paraprevotella	-0.01	0.93	0.96	0.03	0.63	0.88	0.01	0.83	0.95	0.01	0.88	0.95	0.01	0.88	0.94
Parasutterella	0.11	0.08	0.35	0.11	0.08	0.38	0.05	0.42	0.86	0.05	0.39	0.79	0.05	0.43	0.88
Peptococcus	-0.05	0.65	0.84	-0.11	0.33	0.74	-0.18	0.13	0.70	-0.19	0.10	0.63	-0.18	0.12	0.70
Phascolarctobacterium	-0.01	0.93	0.96	0.01	0.84	0.94	0.02	0.76	0.94	0.01	0.91	0.95	0.01	0.83	0.93
Phocea	-0.14	0.38	0.66	-0.14	0.34	0.74	-0.15	0.32	0.85	-0.13	0.38	0.79	-0.16	0.30	0.83
Prevotella	-0.11	0.07	0.29	-0.06	0.29	0.74	-0.07	0.21	0.74	-0.06	0.28	0.79	-0.06	0.30	0.83
Romboutsia	0.13	0.18	0.47	0.15	0.09	0.41	0.11	0.25	0.78	0.10	0.31	0.79	0.08	0.38	0.87
Roseburia	0.15	0.23	0.49	0.12	0.33	0.74	0.10	0.41	0.86	0.11	0.37	0.79	0.10	0.41	0.87
Ruminococcus	0.11	0.14	0.43	0.07	0.34	0.74	0.02	0.77	0.94	0.02	0.80	0.94	0.02	0.77	0.92
Sellimonas	0.29	0.01	0.08	0.25	0.01	0.20	0.24	0.02	0.41	0.23	0.02	0.43	0.24	0.02	0.37
Shuttleworthia	0.07	0.51	0.75	-0.03	0.73	0.91	-0.12	0.22	0.74	-0.13	0.21	0.75	-0.12	0.23	0.80
Slackia	-0.12	0.23	0.49	-0.07	0.46	0.84	-0.08	0.37	0.85	-0.08	0.38	0.79	-0.09	0.32	0.83
Streptococcus	0.01	0.96	0.96	0.01	0.93	0.96	0.09	0.51	0.92	0.05	0.72	0.92	0.05	0.69	0.92
Subdoligranulum	0.19	0.04	0.23	0.15	0.10	0.41	0.07	0.43	0.87	0.07	0.45	0.86	0.06	0.49	0.88
Sutterella	-0.07	0.24	0.49	-0.03	0.62	0.88	-0.02	0.74	0.94	-0.02	0.70	0.92	-0.03	0.63	0.92
TM7x	0.05	0.72	0.85	-0.05	0.67	0.91	-0.06	0.64	0.94	-0.08	0.54	0.91	-0.08	0.55	0.91
Turicibacter	0.28	0.03	0.19	0.33	0.01	0.15	0.32	0.01	0.17	0.31	0.01	0.23	0.29	0.02	0.34
Tuzzerella	-0.20	0.02	0.16	-0.16	0.05	0.35	-0.15	0.09	0.70	-0.14	0.09	0.63	-0.16	0.07	0.60
Tyzzerella	0.23	0.05	0.26	0.23	0.04	0.34	0.24	0.05	0.63	0.26	0.03	0.46	0.30	0.01	0.34
UBA1819	-0.09	0.57	0.80	-0.09	0.55	0.88	-0.11	0.46	0.91	-0.14	0.35	0.79	-0.14	0.34	0.83
UCG.002	0.06	0.36	0.64	0.03	0.70	0.91	-0.06	0.37	0.85	-0.07	0.31	0.79	-0.08	0.27	0.83
UCG.003	0.12	0.10	0.38	0.05	0.52	0.88	-0.02	0.78	0.94	-0.02	0.77	0.92	-0.03	0.74	0.92
UCG.005	0.09	0.31	0.57	0.07	0.39	0.76	-0.02	0.84	0.95	-0.03	0.71	0.92	-0.03	0.77	0.92
UCG.009	0.18	0.17	0.47	0.08	0.53	0.88	0.01	0.95	0.98	-0.02	0.89	0.95	-0.01	0.92	0.97
Veillonella	0.15	0.12	0.39	0.12	0.18	0.55	0.14	0.15	0.70	0.13	0.17	0.75	0.13	0.17	0.78
Victivallis	0.28	0.00	0.07	0.22	0.01	0.20	0.15	0.11	0.70	0.13	0.14	0.75	0.14	0.12	0.70

Multivariable-adjusted regression models adjusted for:
Model 1: sequencing batch, age, race, gender, study center
Model 2: Model 1, with education
Model 3: Model 2, with physical activity, diet score, medication use, and smoking

© 2022 Meyer K et al. *JAMA Network Open.*

Model 4: Model 3 with BMI
Model 5: Model 4 with hypertension and diabetes

© 2022 Meyer K et al. *JAMA Network Open.*

eTable 40: Multivariable-adjusted regression coefficients, p-values, sex interaction p-values, false discovery rate (FDR)-adjusted p-values for sex-genera interaction with respect to DSST.

	Model1					Model2					Model3					Model4					Model5				
	beta	p-value	p-val int	FDR	FDR-int	beta	p-value	p-val int	FDR	FDR-int	beta	p-value	p-val int	FDR	FDR-int	beta	p-value	p-val int	FDR	FDR-int	beta	p-value	p-val int	FDR	FDR-int
Acetanaerobacterium	0.51	0.81	0.61	0.88	0.98	0.52	0.79	0.90	0.90	0.99	0.46	0.83	0.96	0.95	0.98	0.46	0.83	0.96	0.95	0.98	0.39	0.85	0.88	0.94	0.96
Acidaminococcus	-0.09	0.89	0.25	0.92	0.74	0.34	0.57	0.24	0.73	0.81	0.48	0.45	0.41	0.79	0.89	0.48	0.45	0.42	0.79	0.90	0.51	0.42	0.37	0.75	0.89
Actinomyces	-1.54	0.38	0.40	0.57	0.91	-1.42	0.39	0.28	0.63	0.81	-0.78	0.66	0.24	0.92	0.80	-0.83	0.64	0.23	0.90	0.79	-0.70	0.69	0.21	0.94	0.78
Adlercreutzia	0.70	0.48	0.41	0.64	0.92	0.82	0.39	0.64	0.63	0.99	1.16	0.24	0.95	0.63	0.98	1.16	0.24	0.95	0.63	0.98	1.11	0.26	0.94	0.65	0.96
Agathobacter	1.00	0.49	0.59	0.64	0.98	0.46	0.74	0.85	0.86	0.99	-0.12	0.93	0.83	0.95	0.98	-0.12	0.93	0.83	0.95	0.98	-0.13	0.93	0.88	0.96	0.96
Akkermansia	2.20	0.00	0.25	0.07	0.74	1.68	0.01	0.41	0.24	0.84	1.63	0.02	0.50	0.51	0.96	1.63	0.02	0.49	0.51	0.96	1.61	0.03	0.56	0.48	0.96
Alistipes	2.82	0.02	0.06	0.12	0.74	1.97	0.10	0.15	0.31	0.81	1.61	0.20	0.19	0.63	0.78	1.61	0.20	0.19	0.63	0.78	1.51	0.23	0.21	0.65	0.78
Allisonella	-1.04	0.35	0.22	0.56	0.74	-0.08	0.94	0.32	0.96	0.81	-0.10	0.93	0.73	0.95	0.98	-0.10	0.93	0.73	0.95	0.98	-0.05	0.96	0.75	0.97	0.96
Anaerofilum	1.74	0.26	0.79	0.49	1.00	1.04	0.48	0.99	0.67	0.99	1.47	0.34	0.72	0.72	0.98	1.47	0.34	0.72	0.72	0.98	1.45	0.35	0.79	0.71	0.96
Anaerostipes	2.16	0.19	0.20	0.41	0.74	1.39	0.38	0.28	0.63	0.81	1.49	0.37	0.26	0.72	0.80	1.51	0.36	0.25	0.72	0.80	1.60	0.34	0.25	0.71	0.81
Anaerotruncus	1.56	0.26	0.97	0.49	1.00	0.95	0.48	0.80	0.67	0.99	0.68	0.63	0.81	0.91	0.98	0.69	0.62	0.81	0.90	0.98	0.68	0.63	0.84	0.91	0.96
Bacteroides	-0.23	0.91	0.29	0.94	0.74	-0.62	0.76	0.26	0.86	0.81	0.18	0.93	0.22	0.95	0.79	0.19	0.93	0.22	0.95	0.79	0.11	0.96	0.21	0.97	0.78
Barnesiella	1.74	0.01	0.46	0.08	0.94	1.81	0.00	0.35	0.24	0.84	1.32	0.04	0.66	0.51	0.98	1.32	0.04	0.66	0.51	0.98	1.34	0.04	0.75	0.48	0.96
Bifidobacterium	-0.92	0.26	0.12	0.49	0.74	-0.38	0.63	0.11	0.77	0.81	-0.48	0.54	0.16	0.84	0.78	-0.48	0.54	0.16	0.85	0.78	-0.44	0.57	0.16	0.86	0.78
Bilophila	0.51	0.58	0.62	0.72	0.98	-0.13	0.89	0.98	0.94	0.99	-0.44	0.64	0.94	0.91	0.98	-0.43	0.65	0.95	0.90	0.98	-0.48	0.62	0.93	0.90	0.96
Blautia	7.64	0.01	0.17	0.08	0.74	5.68	0.05	0.37	0.28	0.84	6.32	0.04	0.41	0.51	0.89	6.32	0.04	0.41	0.51	0.90	6.33	0.04	0.52	0.48	0.96
Butyricicoccus	6.08	0.01	0.08	0.08	0.74	4.31	0.05	0.12	0.28	0.81	4.57	0.04	0.07	0.51	0.78	4.58	0.04	0.07	0.51	0.78	4.67	0.04	0.08	0.48	0.78
Butyricimonas	0.49	0.52	0.91	0.66	1.00	0.14	0.84	0.95	0.93	0.99	0.22	0.77	0.91	0.95	0.98	0.22	0.77	0.91	0.95	0.98	0.19	0.80	0.91	0.94	0.96
CAG.56	0.75	0.37	0.95	0.57	1.00	0.31	0.69	0.88	0.82	0.99	-0.17	0.83	0.91	0.95	0.98	-0.17	0.83	0.91	0.95	0.98	-0.22	0.79	0.80	0.94	0.96
Candidatus_Soleaferrea	1.35	0.42	0.99	0.61	1.00	0.49	0.76	0.72	0.86	0.99	0.20	0.90	0.57	0.95	0.98	0.20	0.90	0.57	0.95	0.98	0.19	0.91	0.63	0.96	0.96
Caproiciproducens	0.51	0.74	0.94	0.84	1.00	0.27	0.85	0.75	0.93	0.99	0.22	0.89	0.86	0.95	0.98	0.22	0.89	0.86	0.95	0.98	0.28	0.86	0.82	0.94	0.96
Christensenellaceae_R.7_group	1.69	0.02	0.82	0.11	1.00	1.35	0.05	0.75	0.28	0.99	1.16	0.11	0.49	0.58	0.96	1.16	0.11	0.49	0.58	0.96	1.15	0.12	0.59	0.60	0.96
Clostridium_sensu_stricto_1	0.99	0.25	0.19	0.49	0.74	1.31	0.11	0.09	0.34	0.81	0.93	0.28	0.14	0.63	0.78	0.93	0.28	0.14	0.63	0.78	1.02	0.24	0.11	0.65	0.78
Colidextribacter	-1.24	0.47	0.68	0.64	0.98	-2.20	0.18	0.96	0.46	0.99	-1.22	0.47	0.81	0.81	0.98	-1.22	0.47	0.80	0.81	0.98	-1.14	0.50	0.83	0.83	0.96
Collinsella	-0.76	0.38	0.66	0.57	0.98	-0.44	0.59	0.99	0.75	0.99	-0.74	0.39	0.56	0.74	0.98	-0.74	0.39	0.56	0.74	0.98	-0.74	0.39	0.51	0.73	0.96
Coprobacter	0.82	0.34	0.21	0.56	0.74	0.56	0.49	0.16	0.68	0.81	0.02	0.98	0.29	0.98	0.80	0.02	0.98	0.29	0.98	0.80	-0.04	0.97	0.35	0.97	0.87
Coprococcus	2.42	0.02	0.16	0.11	0.74	1.65	0.10	0.58	0.31	0.97	1.42	0.16	0.69	0.63	0.98	1.42	0.16	0.69	0.63	0.98	1.41	0.17	0.74	0.65	0.96
Defluviitaleaceae_UCG.011	2.99	0.04	0.72	0.15	0.99	3.03	0.03	0.43	0.24	0.84	3.27	0.02	0.13	0.51	0.78	3.27	0.02	0.14	0.51	0.78	3.25	0.02	0.13	0.48	0.78
Desulfovibrio	0.23	0.76	0.88	0.85	1.00	0.29	0.68	0.86	0.82	0.99	0.28	0.71	0.85	0.95	0.98	0.29	0.71	0.85	0.95	0.98	0.27	0.73	0.80	0.94	0.96
Dialister	0.04	0.95	0.92	0.97	1.00	0.10	0.87	0.96	0.93	0.99	0.41	0.51	0.81	0.82	0.98	0.41	0.51	0.81	0.82	0.98	0.47	0.46	0.75	0.80	0.96
DNF00809	2.24	0.16	0.67	0.36	0.98	1.89	0.21	0.71	0.51	0.99	1.86	0.24	0.66	0.63	0.98	1.87	0.24	0.66	0.63	0.98	1.89	0.24	0.65	0.65	0.96
Dorea	1.75	0.18	0.09	0.40	0.74	1.38	0.27	0.22	0.58	0.81	1.75	0.17	0.14	0.63	0.78	1.75	0.17	0.14	0.63	0.78	1.71	0.18	0.14	0.65	0.78
DTU089	1.90	0.13	0.26	0.33	0.74	1.82	0.13	0.19	0.39	0.81	1.35	0.28	0.32	0.63	0.80	1.35	0.28	0.32	0.63	0.80	1.35	0.29	0.33	0.65	0.87
Eggerthella	-0.76	0.53	0.24	0.66	0.74	-1.19	0.30	0.13	0.58	0.81	-0.70	0.57	0.16	0.86	0.78	-0.70	0.57	0.16	0.86	0.78	-0.69	0.57	0.14	0.86	0.78
Eisenbergiella	-1.95	0.11	0.03	0.31	0.74	-1.99	0.09	0.02	0.31	0.74	-1.61	0.19	0.04	0.63	0.68	-1.60	0.20	0.04	0.63	0.68	-1.54	0.22	0.03	0.65	0.73
Erysipelatoclostridium	-1.22	0.32	0.22	0.54	0.74	-1.07	0.36	0.24	0.63	0.81	-0.39	0.75	0.30	0.95	0.80	-0.39	0.75	0.30	0.95	0.80	-0.32	0.79	0.32	0.94	0.87
Erysipelotrichaceae_UCG.003	0.93	0.32	0.67	0.54	0.98	0.76	0.39	0.72	0.63	0.99	0.53	0.57	0.46	0.86	0.95	0.53	0.57	0.46	0.86	0.94	0.53	0.57	0.48	0.86	0.96
Escherichia.Shigella	-1.42	0.10	0.02	0.29	0.74	-0.88	0.28	0.03	0.58	0.74	-1.09	0.21	0.01	0.63	0.53	-1.09	0.21	0.01	0.63	0.53	-1.11	0.20	0.01	0.65	0.56
Faecalibacterium	2.47	0.09	0.56	0.27	0.98	2.52	0.07	0.32	0.31	0.81	2.83	0.06	0.12	0.51	0.78	2.83	0.06	0.12	0.51	0.78	2.92	0.05	0.12	0.48	0.78
Family_XIII_AD3011_group	3.57	0.00	0.16	0.07	0.74	2.48	0.03	0.34	0.24	0.84	2.22	0.07	0.27	0.51	0.80	2.24	0.07	0.27	0.51	0.80	2.22	0.07	0.31	0.55	0.87
Family_XIII_UCG.001	2.98	0.03	0.16	0.13	0.74	2.47	0.06	0.16	0.30	0.81	1.88	0.17	0.16	0.63	0.78	1.89	0.17	0.16	0.63	0.78	1.86	0.18	0.18	0.65	0.78
FD2005	0.43	0.73	0.91	0.84	1.00	0.73	0.54	0.93	0.72	0.99	0.62	0.62	0.92	0.91	0.98	0.62	0.61	0.92	0.90	0.98	0.62	0.62	0.93	0.90	0.96
Flavonifractor	-1.14	0.29	0.77	0.52	1.00	-1.11	0.28	0.74	0.58	0.99	-0.30	0.78	0.88	0.95	0.98	-0.30	0.78	0.88	0.95	0.98	-0.24	0.83	0.95	0.94	0.96
Fournierella	1.23	0.30	0.97	0.52	1.00	1.18	0.30	0.89	0.58	0.99	0.28	0.81	0.97	0.95	0.98	0.28	0.81	0.97	0.95	0.98	0.27	0.82	0.89	0.94	0.96
Frisingicoccus	-1.77	0.10	0.28	0.29	0.74	-1.49	0.15	0.61	0.41	0.99	-1.30	0.22	0.74	0.63	0.98	-1.30	0.23	0.75	0.63	0.98	-1.33	0.22	0.68	0.65	0.96
Fusicatenibacter	0.78	0.48	0.58	0.64	0.98	0.61	0.56	0.45	0.73	0.84	0.14	0.90	0.66	0.95	0.98	0.14	0.90	0.66	0.95	0.98	0.12	0.91	0.73	0.96	0.96
Fusobacterium	-1.94	0.09	0.07	0.27	0.74	-1.51	0.16	0.05	0.42	0.74	-1.04	0.36	0.02	0.72	0.55	-1.04	0.36	0.02	0.72	0.56	-1.11	0.33	0.02	0.70	0.64
GCA.900066575	1.60	0.21	0.45	0.43	0.94	0.93	0.44	0.40	0.67	0.84	0.36	0.77	0.29	0.95	0.80	0.35	0.78	0.28	0.95	0.80	0.36	0.77	0.25	0.94	0.81
Gordonibacter	-0.52	0.71	0.51	0.83	0.95	-1.02	0.45	0.42	0.67	0.84	-0.13	0.93	0.83	0.95	0.98	-0.14	0.92	0.83	0.95	0.98	-0.18	0.90	0.84	0.96	0.96
Granulicatella	-2.97	0.10	0.10	0.29	0.74	-2.49	0.15	0.15	0.41	0.81	-1.73	0.35	0.36	0.72	0.84	-1.75	0.35	0.36	0.72	0.84	-1.68	0.37	0.35	0.72	0.87
Haemophilus	1.58	0.08	0.60	0.26	0.98	1.48	0.08	0.38	0.31	0.84	1.73	0.06	0.13	0.51	0.78	1.73	0.06	0.13	0.51	0.78	1.79	0.05	0.15	0.48	0.78
Harryflintia	1.41	0.35	0.40	0.56	0.91	0.86	0.55	0.58	0.73	0.97	0.27	0.86	0.91	0.95	0.98	0.27	0.86	0.91	0.95	0.98	0.15	0.92	0.93	0.96	0.96
Holdemanella	0.24	0.78	0.96	0.85	1.00	0.33	0.69	0.88	0.82	0.99	0.29	0.74	0.68	0.95	0.98	0.28	0.74	0.68	0.95	0.98	0.27	0.76	0.65	0.94	0.96
Holdemania	1.31	0.59	0.88	0.72	1.00	1.74	0.45	0.70	0.67	0.99	3.47	0.15	0.31	0.63	0.80	3.47	0.15	0.31	0.63	0.80	3.39	0.16	0.28	0.65	0.85
Hungatella	-2.60	0.03	0.01	0.13	0.74	-2.53	0.03	0.01	0.24	0.54	-2.18	0.07	0.00	0.51	0.37	-2.18	0.07	0.00	0.51	0.37	-2.11	0.08	0.00	0.55	0.35
Hydrogenoanaerobacterium	4.40	0.00	0.04	0.07	0.74	3.36	0.02	0.06	0.24	0.74	3.01	0.05	0.05	0.51	0.68	3.01	0.05	0.05	0.51	0.68	3.05	0.05	0.05	0.48	0.73
Incertae_Sedis	4.02	0.06	0.46	0.19	0.94	4.44	0.03	0.35	0.24	0.84	4.55	0.03	0.36	0.51	0.84	4.55	0.03	0.36	0.51	0.84	4.50	0.03	0.38	0.48	0.89
Intestinibacter	0.04	0.97	0.18	0.97	0.74	0.49	0.61	0.17	0.77	0.81	0.23	0.83	0.09	0.95	0.78	0.25	0.81	0.09	0.95	0.78	0.36	0.73	0.09	0.94	0.78
Intestinimonas	2.95	0.01	0.12	0.08	0.74	1.84	0.09	0.30	0.31	0.81	1.45	0.20	0.30	0.63	0.80	1.45	0.20	0.30	0.63	0.80	1.37	0.23	0.38	0.65	0.89
Lachnoclostridium	-5.34	0.02	0.51	0.11	0.95	-4.76	0.03	0.28	0.24	0.81	-2.91	0.20	0.32	0.63	0.80	-2.91	0.20	0.32	0.63	0.80	-2.92	0.20	0.35	0.65	0.87
Lachnospira	3.68	0.00	0.63	0.07	0.98	2.69	0.02	0.69	0.24	0.99	2.06	0.08	0.66	0.51	0.98	2.09	0.08	0.65	0.51	0.98	2.08	0.08	0.71	0.55	0.96
Lachnospiraceae_FCS020_group	3.54	0.00	0.84	0.07	1.00	2.95	0.01	0.94	0.24	0.99	2.93	0.01	0.70	0.51	0.98	2.93	0.01	0.70	0.51	0.98	2.95	0.01	0.73	0.48	0.96
Lachnospiraceae_ND3007_group	1.21	0.13	0.11	0.33	0.74	0.74	0.33	0.10	0.62	0.81	0.72	0.37	0.04	0.72	0.68	0.72	0.37	0.04	0.72	0.68	0.71	0.38	0.05	0.72	0.73
Lachnospiraceae_NK4A136_group	2.66	0.02	0.14	0.11	0.74	1.83	0.09	0.13	0.31	0.81	1.22	0.28	0.08	0.63	0.78	1.22	0.28	0.08	0.63	0.78	1.22	0.28	0.09	0.65	0.78
Lachnospiraceae_UCG.001	2.09	0.01	0.43	0.08	0.94	1.82	0.02	0.43	0.24	0.84	1.64	0.05	0.42	0.51	0.89	1.64	0.05	0.42	0.51	0.90	1.63	0.05	0.49	0.48	0.96
Lachnospiraceae_UCG.004	2.59	0.01	0.15	0.08	0.74	1.96	0.05	0.28	0.28	0.81	1.90	0.06	0.20	0.51	0.78	1.91	0.06	0.20	0.51	0.78	1.95	0.05	0.17	0.48	0.78
Lachnospiraceae_UCG.010	0.26	0.85	0.95	0.91	1.00	-0.42	0.75	0.89	0.86	0.99	-0.38	0.78	0.94	0.95	0.98	-0.39	0.77	0.94	0.95	0.98	-0.49	0.72	0.98	0.94	0.98
Lactobacillus	-1.73	0.15	0.69	0.35	0.98	-0.94	0.41	0.71	0.65	0.99	-1.44	0.24	0.56	0.63	0.98	-1.44	0.24	0.56	0.63	0.98	-1.33	0.28	0.55	0.65	0.96
Lactococcus	0.81	0.59	0.78	0.72	1.00	0.03	0.98	0.90	0.98	0.99	0.30	0.84	0.82	0.95	0.98	0.30	0.84	0.82	0.95	0.98	0.28	0.85	0.81	0.94	0.96
Marvinbryantia	1.33	0.14	0.73	0.35	0.99	0.83	0.34	0.90	0.63	0.99	0.61	0.50	0.72	0.82	0.98	0.61	0.51	0.72	0.82	0.98	0.61	0.51	0.76	0.83	0.96
Megasphaera	-0.66	0.35	0.35	0.56	0.84	-0.12	0.86	0.32	0.93	0.81	-0.05	0.94	0.17	0.95	0.78	-0.06	0.94	0.17	0.95	0.78	-0.04	0.95	0.18	0.97	0.78

© 2022 Meyer K et al. *JAMA Network Open.*

Monoglobus	2.25	0.04	1.00	0.15	1.00	1.82	0.08	0.94	0.31	0.99	1.26	0.26	0.96	0.63	0.98	1.26	0.27	0.96	0.63	0.98	1.19	0.29	0.87	0.66	0.96
Moryella	3.66	0.02	0.25	0.10	0.74	3.12	0.03	0.18	0.24	0.81	2.20	0.14	0.20	0.63	0.78	2.20	0.15	0.20	0.63	0.78	2.19	0.15	0.22	0.65	0.78
Negativibacillus	-0.33	0.75	0.70	0.85	0.99	-0.66	0.51	0.87	0.69	0.99	-0.88	0.40	0.67	0.74	0.98	-0.88	0.40	0.67	0.74	0.98	-0.92	0.38	0.66	0.72	0.96
NK4A214_group	1.24	0.12	0.48	0.31	0.94	0.72	0.35	0.66	0.63	0.99	0.18	0.82	0.82	0.95	0.98	0.18	0.82	0.82	0.95	0.98	0.18	0.83	0.94	0.94	0.96
Odoribacter	0.61	0.44	0.64	0.62	0.98	0.55	0.47	0.46	0.67	0.85	0.22	0.78	0.50	0.95	0.96	0.22	0.78	0.50	0.95	0.96	0.16	0.84	0.54	0.94	0.96
Oscillibacter	2.46	0.06	0.61	0.19	0.98	2.13	0.08	0.57	0.31	0.97	2.14	0.10	0.50	0.53	0.96	2.15	0.10	0.50	0.53	0.96	2.18	0.10	0.51	0.55	0.96
Oscillospira	1.69	0.36	0.29	0.56	0.74	1.38	0.43	0.41	0.66	0.84	2.41	0.18	0.27	0.63	0.80	2.41	0.18	0.27	0.63	0.80	2.42	0.18	0.30	0.65	0.87
Oxalobacter	3.41	0.01	0.26	0.08	0.74	2.54	0.04	0.21	0.26	0.81	2.21	0.09	0.26	0.51	0.80	2.21	0.09	0.26	0.51	0.80	2.09	0.11	0.32	0.58	0.87
Paludicola	3.61	0.01	0.12	0.07	0.74	2.31	0.07	0.30	0.31	0.81	1.89	0.15	0.22	0.63	0.79	1.89	0.16	0.22	0.63	0.79	1.92	0.15	0.27	0.65	0.85
Parabacteroides	0.50	0.60	0.19	0.72	0.74	0.94	0.29	0.14	0.58	0.81	1.00	0.27	0.18	0.63	0.78	1.02	0.27	0.17	0.63	0.78	0.99	0.28	0.19	0.65	0.78
Paraprevotella	0.31	0.62	0.04	0.74	0.74	0.52	0.39	0.03	0.63	0.74	0.59	0.36	0.04	0.72	0.68	0.59	0.37	0.05	0.72	0.68	0.65	0.33	0.04	0.70	0.73
Parasutterella	1.03	0.15	0.67	0.35	0.98	1.02	0.14	0.49	0.39	0.88	0.49	0.50	0.84	0.82	0.98	0.49	0.50	0.84	0.82	0.98	0.43	0.55	0.85	0.86	0.96
Peptococcus	2.44	0.05	0.27	0.18	0.74	1.71	0.15	0.30	0.41	0.81	1.44	0.25	0.33	0.63	0.80	1.44	0.25	0.33	0.63	0.80	1.44	0.25	0.39	0.65	0.89
Phascolarctobacterium	-0.03	0.96	0.92	0.97	1.00	-0.06	0.92	0.76	0.95	0.99	-0.07	0.91	0.84	0.95	0.98	-0.07	0.91	0.84	0.95	0.98	-0.10	0.88	0.84	0.96	0.96
Phocea	0.46	0.77	0.91	0.85	1.00	-0.30	0.85	0.77	0.93	0.99	-0.54	0.73	0.45	0.95	0.95	-0.54	0.73	0.45	0.95	0.94	-0.53	0.74	0.45	0.94	0.96
Prevotella	-0.71	0.19	0.77	0.41	1.00	-0.56	0.28	0.93	0.58	0.99	-0.62	0.26	0.83	0.63	0.98	-0.62	0.26	0.83	0.63	0.98	-0.60	0.27	0.83	0.65	0.96
Romboutsia	0.83	0.42	0.33	0.61	0.83	1.22	0.21	0.23	0.51	0.81	1.17	0.25	0.19	0.63	0.78	1.17	0.25	0.19	0.63	0.78	1.26	0.22	0.19	0.65	0.78
Roseburia	3.80	0.00	0.04	0.07	0.74	3.00	0.02	0.11	0.24	0.81	2.30	0.10	0.16	0.53	0.78	2.30	0.10	0.16	0.53	0.78	2.31	0.10	0.17	0.55	0.78
Ruminococcus	1.26	0.12	0.85	0.31	1.00	0.95	0.22	0.95	0.51	0.99	0.47	0.57	1.00	0.86	1.00	0.47	0.57	1.00	0.86	1.00	0.49	0.55	0.90	0.86	0.96
Sellimonas	0.72	0.52	0.79	0.66	1.00	0.91	0.39	0.73	0.63	0.99	0.76	0.49	0.78	0.82	0.98	0.76	0.49	0.78	0.82	0.98	0.75	0.50	0.80	0.83	0.96
Senegalimassilia	0.73	0.47	0.60	0.64	0.98	0.70	0.47	0.65	0.67	0.99	-0.19	0.85	0.97	0.95	0.98	-0.20	0.85	0.97	0.95	0.98	-0.19	0.85	0.93	0.94	0.96
Shuttleworthia	2.90	0.03	0.11	0.13	0.74	2.29	0.07	0.04	0.31	0.74	2.28	0.09	0.03	0.51	0.68	2.30	0.08	0.03	0.51	0.68	2.28	0.09	0.04	0.55	0.73
Slackia	-0.34	0.71	0.55	0.83	0.98	-0.03	0.98	0.40	0.98	0.84	-0.41	0.65	0.63	0.91	0.98	-0.41	0.65	0.63	0.90	0.98	-0.43	0.64	0.57	0.91	0.96
Streptococcus	0.23	0.87	0.48	0.92	0.94	0.17	0.90	0.45	0.94	0.84	0.47	0.74	0.84	0.95	0.98	0.46	0.75	0.84	0.95	0.98	0.60	0.68	0.81	0.94	0.96
Subdoligranulum	1.27	0.24	0.82	0.48	1.00	0.93	0.37	0.84	0.63	0.99	0.88	0.40	0.69	0.74	0.98	0.88	0.40	0.69	0.74	0.98	0.87	0.41	0.71	0.75	0.96
Sutterella	-1.27	0.04	0.67	0.15	0.98	-0.98	0.10	0.55	0.31	0.97	-0.87	0.16	0.72	0.63	0.98	-0.87	0.16	0.72	0.63	0.98	-0.85	0.17	0.75	0.65	0.96
TM7x	-1.06	0.47	0.18	0.64	0.74	-1.54	0.27	0.18	0.58	0.81	-1.10	0.45	0.33	0.79	0.80	-1.10	0.45	0.33	0.79	0.80	-1.08	0.46	0.25	0.80	0.81
Turicibacter	-0.20	0.88	0.92	0.92	1.00	0.63	0.62	0.80	0.77	0.99	0.41	0.75	0.93	0.95	0.98	0.42	0.75	0.93	0.95	0.98	0.46	0.72	0.79	0.94	0.96
Tuzzerella	-1.19	0.24	0.81	0.48	1.00	-1.17	0.23	0.99	0.52	0.99	-0.82	0.42	0.93	0.77	0.98	-0.82	0.43	0.92	0.77	0.98	-0.81	0.43	0.86	0.78	0.96
UBA1819	-1.01	0.50	0.35	0.66	0.84	-1.48	0.30	0.20	0.58	0.81	-1.07	0.47	0.18	0.81	0.78	-1.07	0.48	0.18	0.81	0.78	-1.09	0.47	0.20	0.80	0.78
UCG.002	0.62	0.44	0.72	0.62	0.99	0.09	0.91	0.57	0.94	0.97	-0.13	0.87	0.76	0.95	0.98	-0.13	0.87	0.76	0.95	0.98	-0.18	0.82	0.70	0.94	0.96
UCG.003	1.13	0.17	0.85	0.38	1.00	0.56	0.48	0.95	0.67	0.99	0.39	0.64	0.71	0.91	0.98	0.39	0.64	0.71	0.90	0.98	0.37	0.65	0.75	0.92	0.96
UCG.005	1.91	0.04	0.46	0.15	0.94	1.55	0.08	0.38	0.31	0.84	0.87	0.35	0.37	0.72	0.84	0.87	0.36	0.37	0.72	0.84	0.88	0.35	0.41	0.71	0.92
UCG.009	3.28	0.02	0.09	0.10	0.74	2.69	0.04	0.04	0.26	0.74	1.74	0.20	0.09	0.63	0.78	1.73	0.20	0.09	0.63	0.78	1.78	0.19	0.10	0.65	0.78
Veillonella	1.00	0.28	0.99	0.51	1.00	1.12	0.21	0.82	0.51	0.99	1.03	0.27	0.71	0.63	0.98	1.03	0.27	0.71	0.63	0.98	1.10	0.24	0.74	0.65	0.96
Victivallis	2.59	0.00	0.28	0.07	0.74	2.24	0.01	0.21	0.24	0.81	1.56	0.08	0.53	0.51	0.98	1.56	0.08	0.53	0.51	0.98	1.57	0.08	0.61	0.55	0.96

Multivariable-adjusted regression models adjusted for:

Model 1: sequencing batch, age, race, gender, study center

Model 2: Model 1, with education

Model 3: Model 2, with physical activity, diet score, medication use, and smoking

Model 4: Model 3 with BMI

Model 5: Model 4 with hypertension and diabetes

© 2022 Meyer K et al. *JAMA Network Open.*

eTable 41: Multivariable-adjusted regression coefficients, p-values, sex interaction p-values, false discovery rate (FDR)-adjusted p-values sex-genera interaction with respect to Stroop.

	Model1					Model2					Model3					Model4					Model5				
	beta	p-value	p-val int	FDR	FDR-int	beta	p-value	p-val int	FDR	FDR-int	beta	p-value	p-val int	FDR	FDR-int	beta	p-value	p-val int	FDR	FDR-int	beta	p-value	p-val int	FDR	FDR-int
Acetanaerobacterium	0.02	0.99	0.99	1.00	0.99	0.01	1.00	0.88	1.00	0.99	1.11	0.48	0.45	0.88	0.80	1.12	0.48	0.46	0.88	0.79	1.11	0.48	0.42	0.91	0.77
Acidaminococcus	0.36	0.42	0.87	0.69	0.98	0.21	0.63	0.89	0.84	0.99	-0.24	0.61	0.60	0.90	0.80	-0.24	0.61	0.60	0.91	0.79	-0.24	0.61	0.61	0.91	0.82
Actinomyces	0.07	0.95	0.75	0.99	0.96	0.03	0.98	0.68	0.99	0.87	-0.75	0.56	0.71	0.90	0.81	-0.69	0.60	0.67	0.91	0.79	-0.72	0.59	0.70	0.91	0.83
Adlercreutzia	1.82	0.01	0.00	0.25	0.11	1.77	0.01	0.00	0.25	0.11	1.78	0.02	0.00	0.26	0.08	1.78	0.02	0.00	0.25	0.08	1.77	0.02	0.00	0.25	0.07
Agathobacter	-1.57	0.14	0.17	0.48	0.62	-1.38	0.19	0.23	0.56	0.62	-0.63	0.54	0.30	0.90	0.70	-0.67	0.53	0.29	0.88	0.71	-0.63	0.55	0.33	0.91	0.75
Akkermansia	0.26	0.62	0.08	0.83	0.50	0.44	0.40	0.05	0.68	0.39	0.80	0.13	0.01	0.76	0.12	0.80	0.14	0.01	0.73	0.13	0.79	0.14	0.01	0.78	0.14
Alistipes	0.23	0.80	0.42	0.88	0.74	0.53	0.56	0.29	0.81	0.64	1.01	0.27	0.19	0.88	0.57	1.00	0.28	0.20	0.88	0.59	0.99	0.29	0.21	0.88	0.56
Allisonella	0.25	0.76	0.13	0.87	0.61	-0.06	0.94	0.16	0.99	0.59	-0.02	0.98	0.30	0.99	0.70	-0.02	0.98	0.31	0.99	0.72	-0.02	0.98	0.27	0.99	0.68
Anaerofilum	2.61	0.02	0.00	0.25	0.12	2.87	0.01	0.00	0.25	0.11	3.14	0.01	0.00	0.24	0.08	3.14	0.01	0.00	0.24	0.08	3.14	0.01	0.00	0.25	0.07
Anaerostipes	-0.07	0.95	0.91	0.99	0.98	0.23	0.85	0.99	0.94	0.99	0.13	0.92	0.73	0.99	0.81	0.10	0.94	0.71	0.99	0.79	0.15	0.91	0.79	0.99	0.88
Anaerotruncus	1.35	0.18	0.04	0.54	0.43	1.57	0.12	0.03	0.49	0.33	2.66	0.01	0.00	0.25	0.08	2.69	0.01	0.00	0.24	0.08	2.63	0.01	0.00	0.25	0.07
Bacteroides	1.01	0.52	0.05	0.78	0.43	1.13	0.47	0.05	0.71	0.39	1.11	0.47	0.08	0.88	0.41	1.07	0.49	0.08	0.88	0.41	1.07	0.49	0.10	0.91	0.46
Barnesiella	-0.43	0.36	0.57	0.66	0.83	-0.46	0.33	0.61	0.68	0.82	-0.35	0.47	0.50	0.88	0.80	-0.35	0.47	0.50	0.88	0.79	-0.34	0.48	0.44	0.91	0.77
Bifidobacterium	-0.35	0.56	0.90	0.78	0.98	-0.55	0.36	0.87	0.68	0.99	-0.62	0.29	0.62	0.88	0.81	-0.64	0.28	0.62	0.88	0.79	-0.62	0.30	0.64	0.88	0.82
Bilophila	-0.24	0.72	0.69	0.87	0.91	-0.02	0.98	0.51	0.99	0.80	0.27	0.70	0.33	0.90	0.75	0.26	0.72	0.34	0.91	0.78	0.26	0.72	0.36	0.92	0.77
Blautia	0.23	0.92	0.48	0.97	0.76	0.93	0.67	0.33	0.86	0.66	1.27	0.57	0.23	0.90	0.61	1.26	0.57	0.23	0.91	0.62	1.28	0.57	0.15	0.91	0.56
Butyricicoccus	-0.96	0.56	0.88	0.78	0.98	-0.36	0.83	0.96	0.94	0.99	1.06	0.53	0.71	0.88	0.81	1.04	0.53	0.71	0.88	0.79	0.98	0.56	0.74	0.91	0.83
Butyricimonas	0.23	0.68	0.03	0.85	0.42	0.35	0.53	0.02	0.77	0.30	0.20	0.72	0.12	0.91	0.53	0.20	0.71	0.12	0.91	0.52	0.20	0.72	0.12	0.92	0.50
CAG.56	-0.63	0.30	0.96	0.60	0.98	-0.48	0.43	0.97	0.70	0.99	0.21	0.74	0.50	0.92	0.80	0.21	0.74	0.50	0.92	0.79	0.19	0.76	0.47	0.92	0.77
Candidatus_Soleaferrea	1.29	0.29	0.26	0.60	0.64	1.61	0.18	0.18	0.56	0.59	1.25	0.30	0.17	0.88	0.57	1.24	0.31	0.17	0.88	0.57	1.24	0.31	0.14	0.88	0.55
Caproiciproducens	-0.29	0.80	0.58	0.88	0.83	-0.22	0.84	0.52	0.94	0.81	0.76	0.50	0.18	0.88	0.57	0.76	0.50	0.19	0.88	0.57	0.69	0.54	0.19	0.91	0.56
Christensenellaceae_R.7_group	-1.21	0.02	0.89	0.25	0.98	-1.09	0.04	0.87	0.36	0.99	-0.59	0.27	0.71	0.88	0.81	-0.59	0.27	0.71	0.88	0.79	-0.61	0.26	0.65	0.88	0.82
Clostridium_sensu_stricto_1	-0.21	0.74	0.34	0.87	0.70	-0.33	0.59	0.27	0.83	0.64	-0.13	0.84	0.44	0.95	0.80	-0.13	0.84	0.43	0.95	0.79	-0.12	0.85	0.44	0.96	0.77
Colidextribacter	1.04	0.41	0.26	0.69	0.64	1.37	0.28	0.20	0.64	0.60	0.94	0.45	0.23	0.88	0.61	0.94	0.46	0.23	0.88	0.62	0.90	0.48	0.17	0.91	0.56
Collinsella	-0.70	0.27	0.13	0.60	0.61	-0.81	0.19	0.19	0.56	0.59	-0.53	0.41	0.35	0.88	0.77	-0.53	0.41	0.36	0.88	0.79	-0.55	0.40	0.32	0.91	0.75
Coprobacter	-0.38	0.55	0.32	0.78	0.69	-0.29	0.65	0.30	0.84	0.66	0.11	0.86	0.47	0.96	0.80	0.12	0.85	0.46	0.96	0.79	0.11	0.87	0.45	0.96	0.77
Coprococcus	-0.70	0.36	0.38	0.66	0.74	-0.42	0.58	0.66	0.82	0.87	-0.53	0.49	0.50	0.88	0.80	-0.52	0.50	0.51	0.88	0.79	-0.54	0.49	0.54	0.91	0.77
Defluviitaleaceae_UCG.011	-1.96	0.06	0.98	0.37	0.99	-1.98	0.05	0.83	0.43	0.99	-1.06	0.31	0.60	0.88	0.80	-1.06	0.31	0.60	0.88	0.79	-1.10	0.30	0.54	0.88	0.77
Desulfovibrio	-0.38	0.49	0.59	0.75	0.83	-0.41	0.46	0.59	0.71	0.82	-0.30	0.59	0.66	0.90	0.81	-0.28	0.62	0.68	0.91	0.79	-0.31	0.60	0.74	0.91	0.83
Dialister	-0.07	0.87	0.44	0.94	0.74	-0.10	0.82	0.41	0.94	0.70	-0.05	0.92	0.41	0.99	0.80	-0.05	0.91	0.40	0.99	0.79	-0.04	0.93	0.43	0.99	0.77
DNF00809	-2.10	0.07	0.23	0.39	0.64	-1.97	0.09	0.24	0.45	0.64	-1.92	0.10	0.38	0.73	0.80	-1.91	0.11	0.39	0.73	0.79	-1.94	0.10	0.38	0.73	0.77
Dorea	-1.54	0.11	0.40	0.45	0.74	-1.41	0.14	0.55	0.52	0.81	-1.39	0.14	0.56	0.76	0.80	-1.39	0.14	0.58	0.73	0.79	-1.39	0.14	0.55	0.78	0.77
DTU089	0.32	0.73	0.44	0.87	0.74	0.35	0.71	0.47	0.89	0.77	0.95	0.31	0.14	0.88	0.56	0.98	0.30	0.14	0.88	0.55	0.96	0.31	0.12	0.88	0.50
Eggerthella	1.47	0.09	0.19	0.45	0.64	1.64	0.06	0.14	0.43	0.59	1.15	0.20	0.20	0.88	0.59	1.16	0.20	0.20	0.88	0.59	1.16	0.20	0.20	0.88	0.56
Eisenbergiella	1.07	0.23	0.05	0.60	0.43	1.09	0.22	0.04	0.56	0.37	0.27	0.77	0.22	0.94	0.61	0.28	0.76	0.22	0.93	0.62	0.29	0.76	0.21	0.92	0.56
Erysipelatoclostridium	1.70	0.05	0.01	0.37	0.12	1.65	0.06	0.01	0.43	0.11	0.92	0.31	0.05	0.88	0.31	0.92	0.31	0.05	0.88	0.31	0.96	0.29	0.05	0.88	0.29
Erysipelotrichaceae_UCG.003	-0.30	0.66	0.57	0.84	0.83	-0.24	0.72	0.55	0.89	0.81	0.00	0.99	0.54	0.99	0.80	-0.01	0.99	0.56	0.99	0.79	-0.01	0.98	0.51	0.99	0.77
Escherichia.Shigella	0.68	0.28	0.32	0.60	0.69	0.49	0.43	0.38	0.70	0.68	0.31	0.63	0.49	0.90	0.80	0.31	0.63	0.49	0.91	0.79	0.32	0.63	0.50	0.91	0.77
Faecalibacterium	-3.20	0.00	0.14	0.13	0.61	-3.21	0.00	0.10	0.11	0.57	-3.94	0.00	0.01	0.04	0.11	-3.94	0.00	0.01	0.04	0.12	-3.96	0.00	0.01	0.04	0.13
Family_XIII_AD3011_group	-0.82	0.35	0.87	0.66	0.98	-0.44	0.62	0.70	0.84	0.88	0.84	0.35	0.19	0.88	0.57	0.86	0.34	0.18	0.88	0.57	0.82	0.37	0.18	0.91	0.56
Family_XIII_UCG.001	-0.52	0.60	0.56	0.81	0.83	-0.34	0.74	0.55	0.89	0.81	0.40	0.70	0.25	0.90	0.63	0.42	0.69	0.25	0.91	0.63	0.34	0.75	0.25	0.92	0.65
FD2005	-0.99	0.28	0.54	0.60	0.82	-1.09	0.22	0.53	0.56	0.81	-0.41	0.66	0.94	0.90	0.94	-0.43	0.64	0.92	0.91	0.92	-0.41	0.65	0.96	0.91	0.96
Flavonifractor	1.02	0.20	0.18	0.57	0.62	1.00	0.20	0.17	0.56	0.59	0.03	0.97	0.55	0.99	0.80	0.03	0.97	0.55	0.99	0.79	0.02	0.98	0.51	0.99	0.77
Fournierella	-0.74	0.39	0.50	0.68	0.78	-0.72	0.40	0.48	0.68	0.78	-0.22	0.81	0.52	0.95	0.80	-0.20	0.82	0.51	0.95	0.79	-0.18	0.84	0.64	0.96	0.82
Frisingicoccus	2.04	0.01	0.01	0.25	0.12	1.93	0.01	0.02	0.25	0.24	2.32	0.00	0.00	0.18	0.08	2.32	0.00	0.00	0.18	0.08	2.32	0.00	0.01	0.19	0.09
Fusicatenibacter	-1.13	0.17	0.38	0.53	0.74	-1.07	0.19	0.34	0.56	0.66	-0.53	0.52	0.80	0.88	0.85	-0.55	0.51	0.80	0.88	0.86	-0.53	0.52	0.83	0.91	0.88
Fusobacterium	1.69	0.04	0.41	0.36	0.74	1.55	0.06	0.38	0.43	0.68	0.74	0.37	0.66	0.88	0.81	0.76	0.36	0.66	0.88	0.79	0.74	0.38	0.72	0.91	0.83
GCA.900066575	-1.27	0.17	0.94	0.53	0.98	-1.05	0.26	0.95	0.61	0.99	-0.95	0.31	0.65	0.88	0.81	-0.95	0.31	0.64	0.88	0.79	-0.98	0.30	0.72	0.88	0.83
Gordonibacter	1.58	0.13	0.08	0.48	0.50	1.75	0.09	0.07	0.45	0.44	2.05	0.05	0.06	0.51	0.31	2.07	0.05	0.06	0.49	0.32	2.05	0.05	0.07	0.53	0.36
Granulicatella	0.43	0.74	0.60	0.87	0.83	0.26	0.85	0.67	0.94	0.87	-0.88	0.52	0.80	0.88	0.85	-0.86	0.54	0.83	0.88	0.87	-0.87	0.53	0.81	0.91	0.88
Haemophilus	-1.07	0.11	0.23	0.45	0.64	-1.03	0.11	0.18	0.49	0.59	-1.14	0.09	0.09	0.73	0.44	-1.14	0.09	0.09	0.73	0.44	-1.13	0.10	0.11	0.73	0.50
Harryflintia	1.12	0.31	0.08	0.60	0.50	1.31	0.23	0.06	0.56	0.39	2.66	0.02	0.00	0.26	0.08	2.67	0.02	0.00	0.25	0.08	2.64	0.02	0.00	0.25	0.07
Holdemanella	-0.42	0.50	0.29	0.77	0.65	-0.45	0.48	0.34	0.72	0.66	-0.03	0.96	0.66	0.99	0.81	-0.02	0.97	0.67	0.99	0.79	-0.01	0.99	0.68	0.99	0.83
Holdemania	1.85	0.30	0.14	0.60	0.61	1.71	0.33	0.16	0.68	0.59	1.75	0.33	0.10	0.88	0.44	1.78	0.32	0.10	0.88	0.44	1.77	0.33	0.09	0.88	0.46
Hungatella	2.84	0.00	0.00	0.13	0.12	2.81	0.00	0.00	0.11	0.11	2.08	0.02	0.01	0.26	0.10	2.09	0.02	0.01	0.25	0.10	2.12	0.02	0.01	0.25	0.10
Hydrogenoanaerobacterium	-1.53	0.17	0.88	0.53	0.98	-1.17	0.29	0.97	0.65	0.99	-1.03	0.36	0.92	0.88	0.93	-1.03	0.36	0.91	0.88	0.92	-1.05	0.36	0.90	0.91	0.91
Incertae_Sedis	-0.07	0.96	0.55	0.99	0.83	-0.23	0.88	0.60	0.96	0.82	0.05	0.97	0.43	0.99	0.80	0.05	0.97	0.43	0.99	0.79	0.04	0.98	0.40	0.99	0.77
Intestinibacter	-0.47	0.53	0.85	0.78	0.98	-0.61	0.41	0.86	0.68	0.99	-1.11	0.15	0.44	0.78	0.80	-1.16	0.14	0.43	0.73	0.79	-1.14	0.15	0.45	0.78	0.77
Intestinimonas	0.64	0.44	0.04	0.71	0.42	1.05	0.21	0.02	0.56	0.24	1.30	0.13	0.01	0.76	0.12	1.30	0.13	0.01	0.73	0.13	1.28	0.13	0.01	0.78	0.13
Lachnoclostridium	3.82	0.02	0.45	0.25	0.74	3.62	0.03	0.36	0.36	0.66	2.11	0.21	0.80	0.88	0.85	2.09	0.22	0.80	0.88	0.86	2.15	0.20	0.83	0.88	0.88
Lachnospira	-2.38	0.01	0.27	0.20	0.64	-2.06	0.02	0.29	0.25	0.64	-1.99	0.02	0.15	0.29	0.57	-2.03	0.02	0.14	0.27	0.55	-2.02	0.02	0.21	0.28	0.56
Lachnospiraceae_FCS020_group	-1.65	0.05	0.81	0.37	0.98	-1.44	0.08	0.72	0.45	0.90	-0.37	0.67	0.68	0.90	0.81	-0.36	0.67	0.68	0.91	0.79	-0.38	0.66	0.69	0.91	0.83
Lachnospiraceae_ND3007_group	-0.89	0.13	0.17	0.48	0.62	-0.73	0.21	0.17	0.56	0.59	-0.25	0.67	0.42	0.90	0.80	-0.26	0.67	0.42	0.91	0.79	-0.27	0.65	0.46	0.91	0.77
Lachnospiraceae_NK4A136_group	-0.76	0.36	0.22	0.66	0.64	-0.46	0.58	0.22	0.82	0.62	0.34	0.69	0.25	0.90	0.63	0.34	0.69	0.25	0.91	0.63	0.33	0.70	0.29	0.92	0.70
Lachnospiraceae_UCG.001	-1.09	0.07	0.25	0.39	0.64	-1.01	0.09	0.25	0.45	0.64	-0.24	0.70	0.69	0.90	0.81	-0.24	0.70	0.68	0.91	0.79	-0.26	0.68	0.71	0.91	0.83
Lachnospiraceae_UCG.004	-0.93	0.22	0.20	0.57	0.64	-0.71	0.34	0.28	0.68	0.64	-0.66	0.38	0.19	0.88	0.57	-0.67	0.37	0.19	0.88	0.57	-0.65	0.38	0.18	0.91	0.56
Lachnospiraceae_UCG.010	-0.57	0.57	0.63	0.78	0.85	-0.34	0.73	0.63	0.89	0.84	0.69	0.49	0.87	0.88	0.89	0.72	0.47	0.86	0.88	0.89	0.72	0.48	0.88	0.91	0.91
Lactobacillus	-0.63	0.48	0.20	0.75	0.64	-0.93	0.30	0.19	0.66	0.59	-0.51	0.58	0.53	0.90	0.80	-0.50	0.59	0.55	0.91	0.79	-0.48	0.61	0.50	0.91	0.77
Lactococcus	-1.18	0.27	0.25	0.60	0.64	-0.93	0.39	0.33	0.68	0.66	-0.08	0.94	0.63	0.99	0.81	-0.07	0.95	0.63	0.99	0.79	-0.07	0.95	0.54	0.99	0.77
Marvinbryantia	-0.75	0.26	0.13	0.60	0.61	-0.57	0.39	0.16	0.68	0.59	0.02	0.97	0.46	0.99	0.80	0.03	0.96	0.46	0.99	0.79	0.02	0.98	0.50	0.99	0.77
Megasphaera	0.29	0.58	0.81	0.78	0.98	0.10	0.84	0.79	0.94	0.96	-0.28	0.60	0.73	0.90	0.81	-0.28	0.61	0.71	0.91	0.79	-0.27	0.62	0.83	0.91	0.88

© 2022 Meyer K et al. *JAMA Network Open.*

Monoglobus	-1.22	0.13	0.38	0.48	0.74	-1.08	0.18	0.36	0.56	0.66	0.68	0.42	0.02	0.88	0.21	0.68	0.42	0.02	0.88	0.22	0.66	0.44	0.02	0.91	0.16
Moryella	-1.79	0.11	0.39	0.45	0.74	-1.60	0.15	0.34	0.52	0.66	-0.28	0.81	0.77	0.95	0.84	-0.27	0.81	0.78	0.95	0.85	-0.28	0.81	0.83	0.96	0.88
Negativibacillus	0.45	0.55	0.28	0.78	0.64	0.57	0.44	0.18	0.71	0.59	0.60	0.43	0.10	0.88	0.44	0.60	0.44	0.10	0.88	0.44	0.61	0.43	0.09	0.91	0.46
NK4A214_group	-0.21	0.71	0.41	0.87	0.74	-0.03	0.95	0.34	0.99	0.66	0.84	0.15	0.04	0.78	0.26	0.85	0.15	0.04	0.77	0.27	0.82	0.17	0.04	0.85	0.27
Odoribacter	0.47	0.41	0.29	0.69	0.65	0.50	0.38	0.34	0.68	0.66	0.61	0.29	0.37	0.88	0.80	0.61	0.30	0.38	0.88	0.79	0.59	0.31	0.34	0.88	0.75
Oscillibacter	-0.29	0.76	0.27	0.87	0.64	-0.18	0.85	0.27	0.94	0.64	0.61	0.53	0.06	0.88	0.31	0.62	0.53	0.06	0.88	0.32	0.58	0.56	0.06	0.91	0.33
Oscillospira	1.15	0.39	0.08	0.68	0.50	1.24	0.35	0.06	0.68	0.42	1.11	0.40	0.05	0.88	0.31	1.12	0.40	0.05	0.88	0.31	1.07	0.42	0.04	0.91	0.27
Oxalobacter	-1.92	0.04	0.15	0.36	0.61	-1.62	0.08	0.14	0.45	0.59	-0.68	0.48	0.29	0.88	0.70	-0.67	0.48	0.29	0.88	0.71	-0.71	0.47	0.27	0.91	0.68
Paludicola	-1.31	0.17	0.78	0.53	0.98	-0.86	0.37	0.99	0.68	0.99	0.47	0.63	0.30	0.90	0.70	0.49	0.62	0.30	0.91	0.71	0.46	0.65	0.32	0.91	0.75
Parabacteroides	0.00	1.00	0.85	1.00	0.98	-0.15	0.82	0.90	0.94	0.99	-0.20	0.77	0.92	0.94	0.93	-0.18	0.80	0.88	0.95	0.90	-0.20	0.77	0.90	0.93	0.91
Paraprevotella	-0.48	0.31	0.04	0.60	0.42	-0.56	0.23	0.03	0.56	0.33	-0.78	0.11	0.01	0.75	0.11	-0.77	0.12	0.01	0.73	0.12	-0.75	0.13	0.01	0.78	0.13
Parasutterella	-0.77	0.14	0.91	0.48	0.98	-0.77	0.14	0.99	0.52	0.99	-0.31	0.56	0.64	0.90	0.81	-0.32	0.55	0.64	0.90	0.79	-0.33	0.54	0.61	0.91	0.82
Peptococcus	-0.93	0.31	0.73	0.60	0.95	-0.69	0.45	0.77	0.71	0.95	0.14	0.88	0.86	0.97	0.89	0.16	0.86	0.86	0.96	0.89	0.17	0.86	0.90	0.96	0.91
Phascolarctobacterium	-0.07	0.88	0.34	0.94	0.70	-0.06	0.89	0.29	0.97	0.64	-0.21	0.68	0.57	0.90	0.80	-0.20	0.68	0.57	0.91	0.79	-0.22	0.66	0.58	0.91	0.79
Phocea	1.44	0.22	0.63	0.57	0.85	1.70	0.14	0.51	0.52	0.80	1.44	0.22	0.82	0.88	0.86	1.44	0.22	0.81	0.88	0.86	1.45	0.22	0.76	0.88	0.85
Prevotella	0.07	0.85	0.08	0.93	0.50	0.02	0.95	0.12	0.99	0.59	-0.28	0.49	0.03	0.88	0.24	-0.28	0.49	0.03	0.88	0.25	-0.28	0.49	0.04	0.91	0.29
Romboutsia	-0.64	0.39	0.65	0.68	0.87	-0.78	0.29	0.60	0.65	0.82	-0.87	0.25	0.60	0.88	0.80	-0.88	0.25	0.59	0.88	0.79	-0.88	0.25	0.64	0.88	0.82
Roseburia	-1.66	0.09	0.11	0.45	0.60	-1.38	0.16	0.18	0.54	0.59	-1.25	0.23	0.16	0.88	0.57	-1.26	0.22	0.15	0.88	0.57	-1.26	0.22	0.20	0.88	0.56
Ruminococcus	-0.37	0.53	0.94	0.78	0.98	-0.26	0.65	0.98	0.84	0.99	0.39	0.52	0.46	0.88	0.80	0.39	0.52	0.46	0.88	0.79	0.37	0.54	0.45	0.91	0.77
Sellimonas	-0.62	0.45	0.77	0.71	0.97	-0.70	0.39	0.57	0.68	0.81	-0.95	0.24	0.49	0.88	0.80	-0.95	0.25	0.49	0.88	0.79	-0.95	0.25	0.51	0.88	0.77
Senegalimassilia	-0.94	0.21	0.26	0.57	0.64	-0.92	0.22	0.28	0.56	0.64	-0.33	0.66	0.62	0.90	0.81	-0.32	0.68	0.63	0.91	0.79	-0.32	0.68	0.69	0.91	0.83
Shuttleworthia	-0.45	0.64	0.46	0.83	0.74	-0.27	0.78	0.56	0.94	0.81	0.21	0.83	0.43	0.95	0.80	0.20	0.84	0.44	0.95	0.79	0.19	0.85	0.48	0.96	0.77
Slackia	-1.30	0.05	0.17	0.37	0.62	-1.41	0.03	0.14	0.36	0.59	-0.70	0.30	0.53	0.88	0.80	-0.69	0.31	0.55	0.88	0.79	-0.70	0.30	0.56	0.88	0.77
Streptococcus	-0.47	0.64	0.43	0.83	0.74	-0.45	0.65	0.42	0.84	0.72	-0.22	0.84	0.19	0.95	0.57	-0.16	0.88	0.17	0.97	0.57	-0.17	0.87	0.21	0.96	0.56
Subdoligranulum	-0.88	0.26	0.81	0.60	0.98	-0.76	0.32	0.83	0.68	0.99	-0.52	0.50	0.58	0.88	0.80	-0.51	0.51	0.58	0.88	0.79	-0.52	0.51	0.50	0.91	0.77
Sutterella	-0.19	0.68	0.20	0.85	0.64	-0.29	0.52	0.22	0.77	0.62	-0.83	0.07	0.03	0.69	0.24	-0.83	0.07	0.03	0.71	0.25	-0.83	0.07	0.03	0.73	0.25
TM7x	0.31	0.77	0.39	0.88	0.74	0.49	0.65	0.39	0.84	0.68	0.21	0.84	0.57	0.95	0.80	0.22	0.84	0.56	0.95	0.79	0.21	0.85	0.54	0.96	0.77
Turicibacter	-1.22	0.20	0.25	0.57	0.64	-1.52	0.11	0.19	0.49	0.59	-2.29	0.02	0.05	0.26	0.31	-2.32	0.02	0.05	0.25	0.31	-2.31	0.02	0.06	0.25	0.33
Tuzzerella	1.83	0.01	0.04	0.25	0.43	1.81	0.01	0.03	0.25	0.33	1.28	0.09	0.16	0.73	0.57	1.28	0.10	0.17	0.73	0.57	1.27	0.10	0.18	0.73	0.56
UBA1819	1.77	0.11	0.01	0.45	0.12	1.92	0.08	0.00	0.45	0.11	1.40	0.21	0.00	0.88	0.08	1.40	0.21	0.00	0.88	0.08	1.35	0.23	0.00	0.88	0.07
UCG.002	-0.15	0.80	0.97	0.88	0.99	0.04	0.95	0.91	0.99	0.99	0.52	0.38	0.67	0.88	0.81	0.53	0.37	0.67	0.88	0.79	0.51	0.40	0.63	0.91	0.82
UCG.003	-1.44	0.02	0.09	0.25	0.51	-1.25	0.04	0.10	0.36	0.57	-1.00	0.10	0.14	0.73	0.56	-0.99	0.10	0.14	0.73	0.55	-1.00	0.10	0.13	0.73	0.50
UCG.005	-1.23	0.07	0.93	0.39	0.98	-1.11	0.10	0.90	0.45	0.99	-0.19	0.79	0.47	0.95	0.80	-0.18	0.80	0.47	0.95	0.79	-0.22	0.76	0.41	0.92	0.77
UCG.009	-1.12	0.27	0.75	0.60	0.96	-0.90	0.37	0.68	0.68	0.87	0.04	0.97	0.73	0.99	0.81	0.05	0.96	0.73	0.99	0.80	0.03	0.98	0.69	0.99	0.83
Veillonella	0.02	0.98	0.87	1.00	0.98	-0.02	0.98	0.94	0.99	0.99	-0.23	0.74	0.57	0.92	0.80	-0.23	0.74	0.58	0.92	0.79	-0.23	0.75	0.54	0.92	0.77
Victivallis	-1.28	0.06	0.49	0.37	0.77	-1.16	0.08	0.46	0.45	0.76	-0.66	0.33	0.57	0.88	0.80	-0.66	0.33	0.55	0.88	0.79	-0.67	0.32	0.51	0.88	0.77

Multivariable-adjusted regression models adjusted for:

Model 1: sequencing batch, age, race, gender, study center

Model 2: Model 1, with education

Model 3: Model 2, with physical activity, diet score, medication use, and smoking

Model 4: Model 3 with BMI

Model 5: Model 4 with hypertension and diabetes

© 2022 Meyer K et al. *JAMA Network Open.*

eTable 42: Multivariable-adjusted regression coefficients, p-values, sex interaction p-values, false discovery rate (FDR)-adjusted p-values sex-genera interaction with respect to RAVLT.

	Model1					Model2					Model3					Model4					Model5				
	beta	p-value	p-val int	FDR	FDR-int	beta	p-value	p-val int	FDR	FDR-int	beta	p-value	p-val int	FDR	FDR-int	beta	p-value	p-val int	FDR	FDR-int	beta	p-value	p-val int	FDR	FDR-int
Acetanaerobacterium	0.03	0.91	0.70	0.96	0.99	0.03	0.90	0.99	0.95	1.00	-0.14	0.56	0.80	0.83	0.94	-0.14	0.56	0.82	0.84	0.94	-0.14	0.55	0.87	0.84	0.95
Acidaminococcus	-0.17	0.01	0.74	0.23	0.99	-0.13	0.06	0.69	0.57	1.00	-0.12	0.09	0.48	0.53	0.94	-0.12	0.09	0.48	0.54	0.94	-0.12	0.10	0.51	0.56	0.95
Actinomyces	-0.12	0.55	0.96	0.71	0.99	-0.11	0.58	0.82	0.82	1.00	-0.08	0.71	0.83	0.94	0.94	-0.09	0.67	0.77	0.91	0.94	-0.09	0.68	0.75	0.92	0.95
Adlercreutzia	0.08	0.46	0.25	0.67	0.99	0.10	0.37	0.41	0.70	1.00	0.08	0.47	0.55	0.83	0.94	0.09	0.46	0.57	0.84	0.94	0.08	0.47	0.59	0.84	0.95
Agathobacter	0.03	0.83	0.26	0.90	0.99	-0.03	0.87	0.41	0.94	1.00	-0.13	0.43	0.72	0.81	0.94	-0.12	0.45	0.72	0.83	0.94	-0.13	0.43	0.78	0.81	0.95
Akkermansia	0.09	0.26	0.89	0.54	0.99	0.03	0.68	0.60	0.93	1.00	0.01	0.94	0.55	0.98	0.94	0.01	0.92	0.57	0.97	0.94	0.01	0.93	0.56	0.98	0.95
Alistipes	0.26	0.07	0.64	0.38	0.99	0.17	0.22	0.97	0.58	1.00	0.09	0.52	0.97	0.83	0.98	0.10	0.51	0.99	0.84	0.99	0.09	0.52	1.00	0.84	1.00
Allisonella	-0.06	0.63	0.03	0.76	0.99	0.04	0.72	0.04	0.93	0.88	0.04	0.77	0.13	0.94	0.94	0.04	0.78	0.14	0.95	0.94	0.04	0.74	0.13	0.95	0.95
Anaerofilum	0.17	0.32	0.58	0.60	0.99	0.10	0.57	0.76	0.82	1.00	0.11	0.54	0.92	0.83	0.98	0.11	0.54	0.94	0.84	0.99	0.11	0.54	0.92	0.84	0.98
Anaerostipes	0.14	0.48	0.58	0.69	0.99	0.05	0.78	0.74	0.94	1.00	0.09	0.62	0.49	0.88	0.94	0.10	0.60	0.47	0.86	0.94	0.10	0.61	0.53	0.85	0.95
Anaerotruncus	0.06	0.72	0.86	0.81	0.99	-0.01	0.94	0.93	0.95	1.00	-0.03	0.85	0.78	0.96	0.94	-0.04	0.82	0.77	0.95	0.94	-0.04	0.83	0.76	0.95	0.95
Bacteroides	0.30	0.21	0.16	0.50	0.99	0.26	0.26	0.13	0.61	1.00	0.21	0.37	0.20	0.81	0.94	0.22	0.36	0.20	0.82	0.94	0.22	0.36	0.18	0.81	0.95
Barnesiella	0.10	0.18	0.80	0.50	0.99	0.11	0.13	0.91	0.57	1.00	0.10	0.20	0.94	0.66	0.98	0.10	0.20	0.94	0.66	0.99	0.10	0.20	0.99	0.65	1.00
Bifidobacterium	-0.04	0.68	0.61	0.79	0.99	0.02	0.81	0.62	0.94	1.00	0.00	0.98	0.48	0.99	0.94	0.00	0.96	0.48	0.98	0.94	0.00	0.97	0.47	0.99	0.95
Bilophila	0.14	0.20	0.98	0.50	0.99	0.07	0.52	0.62	0.79	1.00	0.07	0.52	0.74	0.83	0.94	0.07	0.50	0.77	0.84	0.94	0.07	0.51	0.81	0.84	0.95
Blautia	0.33	0.34	0.33	0.61	0.99	0.11	0.75	0.62	0.93	1.00	0.10	0.78	0.75	0.94	0.94	0.10	0.77	0.73	0.95	0.94	0.10	0.78	0.84	0.95	0.95
Butyricicoccus	0.17	0.52	0.76	0.71	0.99	-0.04	0.88	0.96	0.94	1.00	-0.07	0.78	0.79	0.94	0.94	-0.07	0.79	0.79	0.95	0.94	-0.07	0.79	0.81	0.95	0.95
Butyricimonas	-0.08	0.38	0.05	0.62	0.99	-0.11	0.17	0.03	0.57	0.88	-0.11	0.18	0.03	0.64	0.82	-0.11	0.18	0.03	0.64	0.80	-0.12	0.18	0.03	0.65	0.78
CAG.56	0.17	0.07	0.17	0.38	0.99	0.13	0.16	0.23	0.57	1.00	0.11	0.26	0.20	0.81	0.94	0.11	0.26	0.21	0.81	0.94	0.11	0.26	0.23	0.81	0.95
Candidatus_Soleaferrea	-0.12	0.54	0.64	0.71	0.99	-0.21	0.24	0.39	0.58	1.00	-0.27	0.16	0.25	0.64	0.94	-0.27	0.16	0.26	0.64	0.94	-0.27	0.16	0.28	0.65	0.95
Caproiciproducens	-0.17	0.33	0.16	0.60	0.99	-0.20	0.24	0.09	0.58	1.00	-0.31	0.08	0.06	0.53	0.94	-0.31	0.08	0.06	0.54	0.94	-0.31	0.08	0.07	0.55	0.95
Christensenellaceae_R.7_group	0.06	0.49	0.47	0.69	0.99	0.02	0.81	0.50	0.94	1.00	0.00	0.96	0.82	0.99	0.94	0.00	0.97	0.83	0.98	0.94	0.00	0.97	0.79	0.99	0.95
Clostridium_sensu_stricto_1	0.05	0.64	0.40	0.76	0.99	0.08	0.38	0.56	0.70	1.00	0.09	0.38	0.76	0.81	0.94	0.09	0.37	0.76	0.82	0.94	0.09	0.36	0.84	0.81	0.95
Colidextribacter	-0.22	0.26	0.88	0.54	0.99	-0.33	0.08	0.59	0.57	1.00	-0.35	0.07	0.83	0.53	0.94	-0.35	0.07	0.85	0.54	0.95	-0.36	0.07	0.79	0.54	0.95
Collinsella	-0.15	0.12	0.54	0.44	0.99	-0.12	0.21	0.84	0.58	1.00	-0.15	0.14	0.76	0.62	0.94	-0.15	0.14	0.74	0.61	0.94	-0.15	0.14	0.78	0.62	0.95
Coprobacter	0.05	0.63	0.54	0.76	0.99	0.02	0.84	0.47	0.94	1.00	-0.01	0.93	0.44	0.98	0.94	-0.01	0.93	0.44	0.97	0.94	-0.01	0.93	0.45	0.98	0.95
Coprococcus	0.27	0.02	0.04	0.29	0.99	0.18	0.10	0.21	0.57	1.00	0.16	0.16	0.21	0.64	0.94	0.16	0.17	0.21	0.64	0.94	0.16	0.17	0.24	0.65	0.95
Defluviitaleaceae_UCG.011	0.34	0.04	0.59	0.35	0.99	0.34	0.03	0.34	0.53	1.00	0.41	0.01	0.14	0.37	0.94	0.41	0.01	0.13	0.37	0.94	0.41	0.01	0.13	0.37	0.95
Desulfovibrio	-0.08	0.36	0.77	0.62	0.99	-0.07	0.39	0.75	0.70	1.00	-0.07	0.42	0.95	0.81	0.98	-0.08	0.38	0.91	0.82	0.98	-0.08	0.39	0.87	0.81	0.95
Dialister	0.09	0.18	0.11	0.50	0.99	0.10	0.14	0.08	0.57	1.00	0.07	0.32	0.16	0.81	0.94	0.07	0.31	0.16	0.82	0.94	0.07	0.32	0.17	0.81	0.95
DNF00809	0.22	0.23	0.40	0.51	0.99	0.18	0.30	0.42	0.66	1.00	0.16	0.39	0.47	0.81	0.94	0.15	0.40	0.48	0.82	0.94	0.16	0.39	0.46	0.81	0.95
Dorea	0.03	0.86	0.41	0.92	0.99	-0.01	0.92	0.76	0.95	1.00	-0.02	0.91	0.79	0.98	0.94	-0.02	0.91	0.80	0.97	0.94	-0.02	0.90	0.78	0.97	0.95
DTU089	0.00	0.99	0.88	1.00	0.99	-0.01	0.93	0.78	0.95	1.00	-0.03	0.86	0.79	0.96	0.94	-0.03	0.83	0.81	0.95	0.94	-0.03	0.85	0.86	0.96	0.95
Eggerthella	0.14	0.29	0.78	0.59	0.99	0.10	0.47	1.00	0.78	1.00	0.14	0.33	0.68	0.81	0.94	0.14	0.34	0.68	0.82	0.94	0.13	0.34	0.75	0.81	0.95
Eisenbergiella	-0.19	0.18	0.23	0.50	0.99	-0.19	0.15	0.19	0.57	1.00	-0.22	0.13	0.18	0.61	0.94	-0.23	0.12	0.19	0.60	0.94	-0.23	0.12	0.16	0.61	0.95
Erysipelatoclostridium	0.08	0.58	0.95	0.73	0.99	0.09	0.49	0.97	0.79	1.00	0.14	0.33	0.68	0.81	0.94	0.14	0.34	0.67	0.82	0.94	0.14	0.34	0.69	0.81	0.95
Erysipelotrichaceae_UCG.003	0.04	0.73	0.78	0.82	0.99	0.02	0.86	0.83	0.94	1.00	-0.02	0.87	0.86	0.96	0.95	-0.02	0.88	0.83	0.96	0.94	-0.01	0.89	0.82	0.97	0.95
Escherichia.Shigella	-0.15	0.13	0.15	0.45	0.99	-0.09	0.35	0.20	0.70	1.00	-0.11	0.29	0.12	0.81	0.94	-0.11	0.29	0.12	0.82	0.94	-0.11	0.28	0.11	0.81	0.95
Faecalibacterium	0.26	0.11	0.66	0.44	0.99	0.27	0.09	0.40	0.57	1.00	0.34	0.05	0.20	0.53	0.94	0.34	0.05	0.20	0.54	0.94	0.35	0.05	0.21	0.54	0.95
Family_XIII_AD3011_group	0.27	0.05	0.36	0.35	0.99	0.14	0.27	0.64	0.61	1.00	0.09	0.54	0.50	0.83	0.94	0.08	0.57	0.50	0.84	0.94	0.08	0.57	0.54	0.84	0.95
Family_XIII_UCG.001	0.00	0.99	0.29	1.00	0.99	-0.06	0.71	0.24	0.93	1.00	-0.05	0.76	0.44	0.94	0.94	-0.05	0.74	0.44	0.95	0.94	-0.05	0.75	0.43	0.95	0.95
FD2005	-0.08	0.59	0.89	0.73	0.99	-0.04	0.75	0.86	0.93	1.00	-0.09	0.53	0.62	0.83	0.94	-0.09	0.55	0.64	0.84	0.94	-0.09	0.54	0.61	0.84	0.95
Flavonifractor	-0.11	0.39	0.69	0.62	0.99	-0.10	0.38	0.65	0.70	1.00	-0.15	0.24	0.50	0.78	0.94	-0.15	0.24	0.50	0.78	0.94	-0.15	0.24	0.50	0.79	0.95
Fournierella	0.05	0.69	0.99	0.79	0.99	0.05	0.71	0.92	0.93	1.00	0.11	0.42	0.52	0.81	0.94	0.11	0.43	0.52	0.82	0.94	0.11	0.43	0.62	0.81	0.95
Frisingicoccus	-0.01	0.96	0.74	1.00	0.99	0.03	0.83	0.80	0.94	1.00	-0.06	0.65	0.93	0.89	0.98	-0.06	0.65	0.90	0.90	0.98	-0.05	0.67	0.92	0.91	0.98
Fusicatenibacter	0.10	0.43	0.30	0.66	0.99	0.08	0.51	0.21	0.79	1.00	0.02	0.89	0.33	0.96	0.94	0.02	0.88	0.33	0.96	0.94	0.02	0.89	0.33	0.97	0.95
Fusobacterium	-0.20	0.12	0.39	0.44	0.99	-0.15	0.22	0.33	0.58	1.00	-0.07	0.59	0.27	0.85	0.94	-0.07	0.57	0.28	0.84	0.94	-0.08	0.56	0.26	0.84	0.95
GCA.900066575	-0.14	0.33	0.18	0.60	0.99	-0.22	0.12	0.17	0.57	1.00	-0.21	0.15	0.42	0.62	0.94	-0.21	0.14	0.45	0.61	0.94	-0.21	0.14	0.48	0.62	0.95
Gordonibacter	0.20	0.23	0.56	0.51	0.99	0.14	0.36	0.48	0.70	1.00	0.14	0.38	0.80	0.81	0.94	0.14	0.38	0.81	0.82	0.94	0.14	0.38	0.82	0.81	0.95
Granulicatella	0.13	0.54	0.87	0.71	0.99	0.18	0.36	0.67	0.70	1.00	0.18	0.41	0.67	0.81	0.94	0.17	0.43	0.71	0.82	0.94	0.18	0.42	0.74	0.81	0.95
Haemophilus	0.19	0.06	0.56	0.38	0.99	0.18	0.07	0.35	0.57	1.00	0.20	0.06	0.25	0.53	0.94	0.20	0.06	0.25	0.54	0.94	0.20	0.06	0.28	0.54	0.95
Harryflintia	0.00	1.00	0.72	1.00	0.99	-0.06	0.70	0.49	0.93	1.00	-0.09	0.62	0.50	0.88	0.94	-0.09	0.62	0.51	0.86	0.94	-0.09	0.60	0.52	0.85	0.95
Holdemanella	-0.04	0.68	0.75	0.79	0.99	-0.03	0.73	0.89	0.93	1.00	-0.04	0.71	0.88	0.94	0.98	-0.04	0.70	0.86	0.93	0.95	-0.04	0.71	0.86	0.95	0.95
Holdemania	0.17	0.54	0.95	0.71	0.99	0.22	0.41	0.77	0.72	1.00	0.09	0.74	0.82	0.94	0.94	0.09	0.76	0.83	0.95	0.94	0.09	0.75	0.72	0.95	0.95
Hungatella	-0.21	0.12	0.83	0.44	0.99	-0.21	0.12	0.66	0.57	1.00	-0.22	0.12	0.39	0.61	0.94	-0.22	0.11	0.38	0.60	0.94	-0.22	0.12	0.33	0.61	0.95
Hydrogenoanaerobacterium	0.24	0.16	0.82	0.50	0.99	0.13	0.44	0.68	0.75	1.00	0.15	0.41	0.96	0.81	0.98	0.15	0.41	0.97	0.82	0.99	0.14	0.42	0.95	0.81	0.99
Incertae_Sedis	0.23	0.35	0.60	0.61	0.99	0.27	0.24	0.48	0.58	1.00	0.15	0.54	0.58	0.83	0.94	0.15	0.54	0.58	0.84	0.94	0.15	0.53	0.59	0.84	0.95
Intestinibacter	0.10	0.41	0.67	0.65	0.99	0.15	0.19	0.69	0.58	1.00	0.11	0.37	0.61	0.81	0.94	0.11	0.34	0.63	0.82	0.94	0.12	0.34	0.57	0.81	0.95
Intestinimonas	0.21	0.11	0.75	0.44	0.99	0.08	0.51	0.36	0.79	1.00	0.10	0.45	0.61	0.82	0.94	0.10	0.44	0.62	0.83	0.94	0.10	0.46	0.53	0.84	0.95
Lachnoclostridium	-0.46	0.08	0.68	0.44	0.99	-0.39	0.12	0.43	0.57	1.00	-0.26	0.33	0.42	0.81	0.94	-0.25	0.34	0.43	0.82	0.94	-0.26	0.33	0.43	0.81	0.95
Lachnospira	0.26	0.05	0.99	0.35	0.99	0.15	0.26	0.92	0.61	1.00	0.11	0.42	1.00	0.81	1.00	0.12	0.39	0.97	0.82	0.99	0.12	0.41	0.93	0.81	0.98
Lachnospiraceae_FCS020_group	0.31	0.02	0.72	0.23	0.99	0.25	0.05	0.51	0.56	1.00	0.23	0.08	0.32	0.53	0.94	0.23	0.08	0.32	0.54	0.94	0.23	0.08	0.31	0.55	0.95
Lachnospiraceae_ND3007_group	0.23	0.01	0.06	0.23	0.99	0.18	0.04	0.05	0.56	0.98	0.18	0.05	0.02	0.53	0.82	0.18	0.05	0.03	0.54	0.80	0.18	0.05	0.03	0.54	0.78
Lachnospiraceae_NK4A136_group	0.20	0.13	0.37	0.45	0.99	0.10	0.41	0.37	0.72	1.00	0.03	0.81	0.26	0.94	0.94	0.03	0.81	0.26	0.95	0.94	0.03	0.81	0.28	0.95	0.95
Lachnospiraceae_UCG.001	0.27	0.00	0.10	0.23	0.99	0.24	0.01	0.09	0.30	1.00	0.21	0.03	0.11	0.53	0.94	0.21	0.03	0.11	0.54	0.94	0.21	0.03	0.13	0.54	0.95
Lachnospiraceae_UCG.004	0.11	0.37	0.45	0.62	0.99	0.03	0.77	0.72	0.94	1.00	0.03	0.77	0.74	0.94	0.94	0.04	0.76	0.74	0.95	0.94	0.03	0.77	0.65	0.95	0.95
Lachnospiraceae_UCG.010	-0.04	0.79	0.49	0.87	0.99	-0.12	0.43	0.51	0.74	1.00	-0.14	0.36	0.51	0.81	0.94	-0.15	0.35	0.52	0.82	0.94	-0.15	0.34	0.51	0.81	0.95
Lactobacillus	-0.37	0.01	0.30	0.23	0.99	-0.29	0.03	0.30	0.53	1.00	-0.35	0.01	0.17	0.37	0.94	-0.35	0.01	0.16	0.37	0.94	-0.36	0.01	0.16	0.37	0.95
Lactococcus	0.02	0.92	0.61	0.96	0.99	-0.07	0.67	0.34	0.93	1.00	-0.03	0.85	0.48	0.96	0.94	-0.03	0.84	0.48	0.95	0.94	-0.03	0.84	0.55	0.96	0.95
Marvinbryantia	0.12	0.25	0.10	0.54	0.99	0.06	0.52	0.14	0.79	1.00	0.09	0.42	0.18	0.81	0.94	0.09	0.42	0.19	0.82	0.94	0.09	0.42	0.20	0.81	0.95
Megasphaera	-0.21	0.01	0.05	0.23	0.99	-0.15	0.05	0.04	0.56	0.88	-0.13	0.11	0.01	0.61	0.82	-0.14	0.10	0.01	0.58	0.80	-0.14	0.10	0.02	0.56	0.78

© 2022 Meyer K et al. *JAMA Network Open.*

Monoglobus	0.34	0.01	0.30	0.23	0.99	0.29	0.01	0.31	0.38	1.00	0.27	0.04	0.32	0.53	0.94	0.27	0.04	0.31	0.54	0.94	0.28	0.04	0.36	0.54	0.95
Moryella	0.17	0.33	0.85	0.60	0.99	0.11	0.51	0.73	0.79	1.00	0.00	0.98	0.93	0.99	0.98	0.00	0.99	0.94	0.99	0.99	0.00	1.00	0.99	1.00	1.00
Negativibacillus	-0.15	0.20	0.70	0.50	0.99	-0.19	0.10	0.34	0.57	1.00	-0.21	0.09	0.29	0.53	0.94	-0.20	0.09	0.30	0.54	0.94	-0.21	0.09	0.33	0.55	0.95
NK4A214_group	0.11	0.22	0.43	0.51	0.99	0.05	0.54	0.59	0.81	1.00	0.05	0.57	0.36	0.84	0.94	0.05	0.59	0.36	0.85	0.94	0.05	0.58	0.40	0.84	0.95
Odoribacter	0.11	0.20	0.92	0.50	0.99	0.11	0.21	0.74	0.58	1.00	0.08	0.36	0.74	0.81	0.94	0.08	0.36	0.73	0.82	0.94	0.08	0.36	0.75	0.81	0.95
Oscillibacter	0.25	0.09	0.73	0.44	0.99	0.22	0.13	0.70	0.57	1.00	0.09	0.56	0.97	0.83	0.98	0.09	0.57	0.97	0.84	0.99	0.09	0.57	0.95	0.84	0.99
Oscillospira	0.07	0.74	0.85	0.82	0.99	0.03	0.86	0.94	0.94	1.00	-0.05	0.81	0.75	0.94	0.94	-0.05	0.80	0.75	0.95	0.94	-0.05	0.81	0.72	0.95	0.95
Oxalobacter	0.23	0.11	0.94	0.44	0.99	0.14	0.33	1.00	0.69	1.00	0.15	0.31	0.48	0.81	0.94	0.15	0.31	0.48	0.82	0.94	0.15	0.32	0.53	0.81	0.95
Paludicola	0.20	0.18	0.46	0.50	0.99	0.05	0.72	0.84	0.93	1.00	0.04	0.79	0.47	0.94	0.94	0.03	0.83	0.46	0.95	0.94	0.04	0.82	0.52	0.95	0.95
Parabacteroides	-0.09	0.40	0.77	0.64	0.99	-0.04	0.69	0.85	0.93	1.00	-0.06	0.55	0.51	0.83	0.94	-0.07	0.52	0.46	0.84	0.94	-0.07	0.53	0.47	0.84	0.95
Paraprevotella	0.00	0.97	0.89	1.00	0.99	0.02	0.77	0.86	0.94	1.00	0.05	0.54	0.49	0.83	0.94	0.04	0.56	0.50	0.84	0.94	0.05	0.54	0.49	0.84	0.95
Parasutterella	0.13	0.12	0.76	0.44	0.99	0.13	0.11	0.94	0.57	1.00	0.02	0.78	0.48	0.94	0.94	0.03	0.76	0.49	0.95	0.94	0.02	0.77	0.52	0.95	0.95
Peptococcus	-0.08	0.56	0.05	0.72	0.99	-0.16	0.23	0.03	0.58	0.88	-0.27	0.06	0.02	0.53	0.82	-0.28	0.05	0.02	0.54	0.80	-0.28	0.05	0.02	0.54	0.78
Phascolarctobacterium	-0.07	0.37	0.07	0.62	0.99	-0.07	0.33	0.04	0.68	0.88	-0.07	0.35	0.05	0.81	0.94	-0.07	0.34	0.05	0.82	0.94	-0.07	0.36	0.05	0.81	0.95
Phocea	-0.38	0.04	0.28	0.35	0.99	-0.47	0.01	0.12	0.30	1.00	-0.52	0.00	0.11	0.37	0.94	-0.52	0.00	0.11	0.37	0.94	-0.52	0.00	0.11	0.37	0.95
Prevotella	-0.12	0.05	0.72	0.35	0.99	-0.10	0.08	0.45	0.57	1.00	-0.08	0.18	0.52	0.64	0.94	-0.08	0.18	0.50	0.64	0.94	-0.08	0.19	0.47	0.65	0.95
Romboutsia	0.08	0.49	0.49	0.69	0.99	0.12	0.27	0.37	0.61	1.00	0.06	0.63	0.58	0.88	0.94	0.06	0.62	0.58	0.86	0.94	0.06	0.61	0.58	0.85	0.95
Roseburia	0.12	0.45	0.42	0.67	0.99	0.03	0.86	0.76	0.94	1.00	-0.11	0.51	0.70	0.83	0.94	-0.10	0.52	0.71	0.84	0.94	-0.11	0.51	0.65	0.84	0.95
Ruminococcus	0.15	0.10	0.75	0.44	0.99	0.12	0.17	0.56	0.57	1.00	0.08	0.38	0.67	0.81	0.94	0.08	0.38	0.66	0.82	0.94	0.08	0.38	0.71	0.81	0.95
Sellimonas	0.15	0.25	0.86	0.54	0.99	0.17	0.17	0.68	0.57	1.00	0.17	0.18	0.71	0.64	0.94	0.17	0.19	0.71	0.64	0.94	0.17	0.19	0.73	0.65	0.95
Senegalimassilia	-0.16	0.18	0.74	0.50	0.99	-0.16	0.15	0.66	0.57	1.00	-0.17	0.15	0.63	0.62	0.94	-0.18	0.14	0.61	0.61	0.94	-0.18	0.14	0.57	0.62	0.95
Shuttleworthia	0.30	0.04	0.91	0.35	0.99	0.24	0.09	0.64	0.57	1.00	0.24	0.13	0.41	0.61	0.94	0.24	0.12	0.39	0.60	0.94	0.24	0.13	0.43	0.61	0.95
Slackia	-0.23	0.02	0.27	0.29	0.99	-0.20	0.04	0.35	0.56	1.00	-0.22	0.03	0.25	0.53	0.94	-0.23	0.03	0.23	0.54	0.94	-0.23	0.03	0.27	0.54	0.95
Streptococcus	0.09	0.55	0.73	0.71	0.99	0.09	0.56	0.70	0.82	1.00	0.16	0.33	0.85	0.81	0.95	0.15	0.36	0.81	0.82	0.94	0.16	0.34	0.81	0.81	0.95
Subdoligranulum	0.16	0.20	0.98	0.50	0.99	0.12	0.31	0.98	0.66	1.00	0.08	0.51	0.97	0.83	0.98	0.08	0.52	0.98	0.84	0.99	0.08	0.51	1.00	0.84	1.00
Sutterella	-0.11	0.11	0.92	0.44	0.99	-0.08	0.24	0.96	0.58	1.00	-0.02	0.77	0.64	0.94	0.94	-0.02	0.75	0.66	0.95	0.94	-0.02	0.78	0.62	0.95	0.95
TM7x	0.07	0.69	0.57	0.79	0.99	0.01	0.93	0.51	0.95	1.00	0.10	0.54	0.22	0.83	0.94	0.10	0.55	0.23	0.84	0.94	0.10	0.54	0.27	0.84	0.95
Turicibacter	0.30	0.04	0.79	0.35	0.99	0.40	0.01	0.53	0.30	1.00	0.38	0.01	0.69	0.37	0.94	0.39	0.01	0.67	0.37	0.94	0.39	0.01	0.58	0.37	0.95
Tuzzerella	-0.17	0.15	0.62	0.49	0.99	-0.16	0.14	0.46	0.57	1.00	-0.23	0.06	0.34	0.53	0.94	-0.23	0.06	0.35	0.54	0.94	-0.23	0.06	0.41	0.54	0.95
UBA1819	-0.17	0.33	0.20	0.60	0.99	-0.22	0.18	0.10	0.57	1.00	-0.31	0.07	0.10	0.53	0.94	-0.32	0.07	0.11	0.54	0.94	-0.32	0.07	0.11	0.54	0.95
UCG.002	0.07	0.43	0.52	0.66	0.99	0.01	0.87	0.39	0.94	1.00	0.00	0.99	0.58	0.99	0.94	0.00	0.98	0.58	0.98	0.94	0.00	0.98	0.56	0.99	0.95
UCG.003	0.07	0.46	0.61	0.67	0.99	0.01	0.95	0.69	0.95	1.00	-0.02	0.81	0.59	0.94	0.94	-0.02	0.80	0.61	0.95	0.94	-0.03	0.79	0.57	0.95	0.95
UCG.005	0.06	0.55	0.95	0.71	0.99	0.02	0.83	0.97	0.94	1.00	0.02	0.89	0.56	0.96	0.94	0.01	0.92	0.56	0.97	0.94	0.01	0.90	0.58	0.97	0.95
UCG.009	0.16	0.30	0.70	0.60	0.99	0.10	0.52	0.51	0.79	1.00	0.01	0.96	0.76	0.99	0.94	0.01	0.97	0.76	0.98	0.94	0.01	0.96	0.79	0.99	0.95
Veillonella	0.13	0.21	0.92	0.50	0.99	0.15	0.15	0.74	0.57	1.00	0.15	0.17	0.63	0.64	0.94	0.15	0.18	0.65	0.64	0.94	0.15	0.17	0.67	0.65	0.95
Victivallis	0.05	0.63	0.39	0.76	0.99	0.01	0.92	0.44	0.95	1.00	-0.02	0.87	0.72	0.96	0.94	-0.02	0.87	0.74	0.96	0.94	-0.02	0.88	0.68	0.97	0.95

Multivariable-adjusted regression models adjusted for:

Model 1: sequencing batch, age, race, gender, study center

Model 2: Model 1, with education

Model 3: Model 2, with physical activity, diet score, medication use, and smoking

Model 4: Model 3 with BMI

Model 5: Model 4 with hypertension and diabetes

© 2022 Meyer K et al. *JAMA Network Open.*

eTable 43: Multivariable-adjusted regression coefficients, p-values, sex interaction p-values, false discovery rate (FDR)-adjusted p-values for sex-genera interaction with respect to category fluency.

	Model1					Model2					Model3					Model4					Model5				
	beta	p-value	p-val int	FDR	FDR-int	beta	p-value	p-val int	FDR	FDR-int	beta	p-value	p-val int	FDR	FDR-int	beta	p-value	p-val int	FDR	FDR-int	beta	p-value	p-val int	FDR	FDR-int
Acetanaerobacterium	-0.16	0.82	1.00	0.89	1.00	-0.15	0.82	0.75	0.91	0.92	-0.39	0.58	0.48	0.76	0.69	-0.39	0.58	0.51	0.76	0.71	-0.44	0.54	0.50	0.73	0.71
Acidaminococcus	-0.20	0.31	0.47	0.55	0.80	-0.09	0.63	0.48	0.81	0.72	-0.14	0.50	0.82	0.70	0.92	-0.14	0.52	0.84	0.71	0.94	-0.13	0.55	0.79	0.73	0.89
Actinomyces	0.44	0.44	0.05	0.65	0.45	0.47	0.39	0.06	0.57	0.40	0.56	0.35	0.10	0.57	0.42	0.49	0.41	0.13	0.64	0.55	0.55	0.37	0.12	0.61	0.52
Adlercreutzia	-0.01	0.97	0.98	0.97	1.00	0.02	0.96	0.77	0.96	0.92	-0.06	0.86	0.85	0.89	0.92	-0.04	0.90	0.76	0.92	0.88	-0.05	0.89	0.71	0.93	0.85
Agathobacter	0.87	0.06	0.05	0.36	0.45	0.73	0.11	0.09	0.35	0.45	0.77	0.11	0.05	0.36	0.34	0.84	0.08	0.04	0.33	0.35	0.84	0.08	0.05	0.32	0.37
Akkermansia	-0.22	0.35	0.14	0.58	0.56	-0.36	0.11	0.06	0.35	0.40	-0.44	0.08	0.06	0.34	0.38	-0.42	0.09	0.07	0.33	0.45	-0.43	0.08	0.08	0.32	0.45
Alistipes	-0.01	0.99	0.80	0.99	0.94	-0.24	0.55	0.48	0.73	0.72	-0.42	0.33	0.47	0.56	0.68	-0.40	0.35	0.51	0.59	0.71	-0.46	0.28	0.44	0.49	0.66
Allisonella	0.09	0.80	0.83	0.88	0.96	0.37	0.31	0.98	0.54	0.99	0.33	0.41	0.85	0.63	0.92	0.31	0.43	0.75	0.64	0.88	0.30	0.45	0.64	0.69	0.79
Anaerofilum	-0.30	0.55	0.09	0.71	0.50	-0.49	0.32	0.13	0.55	0.55	-0.56	0.29	0.13	0.51	0.50	-0.57	0.28	0.14	0.51	0.55	-0.60	0.26	0.13	0.47	0.55
Anaerostipes	-0.54	0.32	0.27	0.56	0.71	-0.75	0.15	0.18	0.38	0.58	-0.67	0.24	0.34	0.45	0.68	-0.63	0.27	0.39	0.49	0.71	-0.64	0.26	0.37	0.47	0.66
Anaerotruncus	-0.73	0.11	0.03	0.42	0.36	-0.89	0.04	0.02	0.28	0.22	-0.94	0.05	0.02	0.34	0.32	-0.98	0.04	0.02	0.33	0.32	-1.01	0.03	0.02	0.31	0.31
Bacteroides	0.29	0.67	0.34	0.81	0.72	0.19	0.78	0.32	0.90	0.71	0.20	0.78	0.32	0.88	0.68	0.26	0.71	0.30	0.82	0.69	0.25	0.72	0.33	0.84	0.66
Barnesiella	0.55	0.01	0.95	0.17	0.99	0.57	0.01	0.85	0.15	0.94	0.58	0.01	0.99	0.17	0.99	0.58	0.01	0.98	0.17	0.98	0.60	0.01	0.95	0.16	0.98
Bifidobacterium	0.29	0.28	0.06	0.53	0.45	0.43	0.10	0.05	0.35	0.40	0.37	0.16	0.09	0.41	0.42	0.39	0.15	0.08	0.38	0.45	0.38	0.16	0.09	0.41	0.45
Bilophila	0.84	0.01	0.27	0.16	0.71	0.67	0.02	0.48	0.28	0.72	0.69	0.03	0.46	0.34	0.68	0.73	0.02	0.39	0.30	0.71	0.75	0.02	0.33	0.27	0.66
Blautia	-0.79	0.42	0.68	0.63	0.90	-1.34	0.17	0.38	0.38	0.71	-1.20	0.24	0.46	0.45	0.68	-1.18	0.25	0.50	0.48	0.71	-1.16	0.26	0.49	0.47	0.71
Butyricicoccus	0.66	0.37	0.84	0.58	0.96	0.18	0.80	0.99	0.91	0.99	0.18	0.81	0.93	0.88	0.97	0.21	0.79	0.90	0.88	0.96	0.20	0.80	0.90	0.89	0.94
Butyricimonas	0.36	0.15	0.99	0.42	1.00	0.27	0.27	0.90	0.52	0.98	0.31	0.23	0.94	0.45	0.97	0.30	0.23	0.92	0.47	0.96	0.29	0.26	0.84	0.47	0.94
CAG.56	0.53	0.05	0.14	0.33	0.56	0.41	0.12	0.17	0.35	0.58	0.39	0.17	0.16	0.41	0.57	0.38	0.17	0.16	0.42	0.58	0.38	0.18	0.17	0.42	0.61
Candidatus_Soleaferrea	-0.67	0.22	0.10	0.46	0.54	-0.90	0.09	0.05	0.34	0.40	-0.97	0.08	0.03	0.34	0.34	-0.96	0.08	0.04	0.33	0.35	-0.98	0.08	0.04	0.32	0.34
Caproiciproducens	-1.20	0.02	0.06	0.18	0.45	-1.27	0.01	0.03	0.15	0.36	-1.32	0.01	0.04	0.17	0.34	-1.32	0.01	0.04	0.17	0.35	-1.33	0.01	0.05	0.16	0.37
Christensenellaceae_R.7_group	0.28	0.24	0.77	0.46	0.94	0.19	0.42	0.81	0.59	0.93	0.15	0.56	0.85	0.75	0.92	0.14	0.58	0.90	0.76	0.96	0.13	0.61	0.88	0.79	0.94
Clostridium_sensu_stricto_1	0.36	0.21	0.16	0.46	0.63	0.44	0.11	0.09	0.35	0.45	0.54	0.07	0.04	0.34	0.34	0.54	0.07	0.04	0.33	0.35	0.59	0.05	0.02	0.31	0.31
Colidextribacter	-0.69	0.22	0.12	0.46	0.56	-0.95	0.08	0.06	0.34	0.40	-0.94	0.10	0.07	0.36	0.42	-0.93	0.11	0.09	0.36	0.45	-0.91	0.11	0.07	0.37	0.45
Collinsella	0.40	0.16	0.51	0.43	0.80	0.49	0.07	0.79	0.31	0.92	0.48	0.10	0.84	0.36	0.92	0.46	0.11	0.92	0.36	0.96	0.45	0.12	0.90	0.38	0.94
Coprobacter	0.51	0.07	0.36	0.38	0.72	0.44	0.11	0.31	0.35	0.71	0.39	0.17	0.23	0.41	0.64	0.39	0.18	0.22	0.42	0.67	0.38	0.19	0.22	0.44	0.65
Coprococcus	0.55	0.10	0.12	0.42	0.56	0.35	0.29	0.37	0.54	0.71	0.37	0.29	0.31	0.51	0.68	0.36	0.30	0.33	0.53	0.71	0.33	0.35	0.43	0.58	0.66
Defluviitaleaceae_UCG.011	-0.19	0.69	0.09	0.82	0.50	-0.17	0.70	0.15	0.85	0.58	-0.13	0.79	0.18	0.88	0.61	-0.13	0.79	0.20	0.88	0.65	-0.14	0.77	0.22	0.86	0.65
Desulfovibrio	-0.32	0.19	0.34	0.46	0.72	-0.30	0.20	0.32	0.43	0.71	-0.40	0.12	0.24	0.36	0.64	-0.46	0.08	0.20	0.33	0.65	-0.48	0.07	0.18	0.32	0.61
Dialister	-0.07	0.73	0.95	0.83	0.99	-0.06	0.78	0.98	0.90	0.99	-0.09	0.66	0.71	0.82	0.86	-0.08	0.70	0.74	0.82	0.88	-0.07	0.74	0.72	0.85	0.85
DNF00809	-0.62	0.23	0.31	0.46	0.71	-0.72	0.16	0.26	0.38	0.71	-0.61	0.26	0.41	0.47	0.68	-0.66	0.23	0.39	0.46	0.71	-0.65	0.24	0.41	0.47	0.66
Dorea	0.32	0.46	0.27	0.65	0.71	0.22	0.60	0.50	0.78	0.73	0.17	0.70	0.53	0.83	0.73	0.16	0.71	0.57	0.82	0.77	0.16	0.71	0.54	0.84	0.74
DTU089	-0.54	0.20	0.67	0.46	0.90	-0.56	0.16	0.73	0.38	0.92	-0.69	0.11	0.53	0.36	0.73	-0.74	0.09	0.49	0.33	0.71	-0.73	0.09	0.43	0.33	0.66
Eggerthella	-0.64	0.11	0.37	0.42	0.72	-0.75	0.05	0.24	0.28	0.70	-0.72	0.08	0.25	0.34	0.64	-0.73	0.08	0.25	0.33	0.67	-0.74	0.07	0.21	0.32	0.65
Eisenbergiella	-1.02	0.01	0.02	0.17	0.27	-1.03	0.01	0.01	0.15	0.22	-1.23	0.00	0.01	0.14	0.13	-1.26	0.00	0.01	0.11	0.15	-1.27	0.00	0.01	0.10	0.12
Erysipelatoclostridium	-0.81	0.04	0.19	0.32	0.67	-0.77	0.05	0.21	0.28	0.62	-0.73	0.08	0.25	0.34	0.64	-0.75	0.07	0.27	0.33	0.68	-0.74	0.07	0.26	0.32	0.66
Erysipelotrichaceae_UCG.003	0.41	0.18	0.27	0.46	0.71	0.37	0.22	0.29	0.45	0.71	0.32	0.31	0.41	0.54	0.68	0.33	0.29	0.36	0.51	0.71	0.33	0.30	0.33	0.51	0.66
Escherichia.Shigella	-0.34	0.22	0.70	0.46	0.90	-0.20	0.46	0.84	0.65	0.94	-0.24	0.42	0.51	0.63	0.72	-0.23	0.43	0.51	0.64	0.71	-0.26	0.39	0.43	0.62	0.66
Faecalibacterium	0.43	0.37	0.52	0.58	0.80	0.44	0.34	0.33	0.55	0.71	0.77	0.13	0.09	0.39	0.42	0.77	0.13	0.09	0.38	0.45	0.75	0.14	0.10	0.39	0.45
Family_XIII_AD3011_group	0.24	0.54	0.93	0.71	0.99	-0.06	0.87	0.74	0.92	0.92	-0.12	0.78	0.84	0.88	0.92	-0.16	0.70	0.83	0.82	0.93	-0.19	0.65	0.85	0.79	0.94
Family_XIII_UCG.001	0.20	0.66	0.30	0.80	0.71	0.06	0.89	0.26	0.92	0.71	-0.02	0.97	0.30	0.97	0.68	-0.06	0.90	0.30	0.92	0.69	-0.07	0.89	0.32	0.93	0.66
FD2005	-0.63	0.12	0.14	0.42	0.56	-0.54	0.17	0.12	0.38	0.55	-0.60	0.15	0.09	0.41	0.42	-0.57	0.18	0.10	0.42	0.48	-0.59	0.17	0.08	0.42	0.45
Flavonifractor	-0.32	0.37	0.76	0.58	0.94	-0.31	0.37	0.77	0.55	0.92	-0.28	0.46	0.87	0.66	0.93	-0.27	0.46	0.88	0.66	0.96	-0.26	0.48	0.89	0.70	0.94
Fournierella	-0.06	0.87	0.82	0.92	0.95	-0.08	0.84	0.75	0.92	0.92	-0.16	0.70	0.84	0.83	0.92	-0.18	0.67	0.81	0.82	0.92	-0.19	0.63	0.90	0.79	0.94
Frisingicoccus	-0.38	0.28	0.06	0.53	0.45	-0.31	0.37	0.15	0.55	0.58	-0.31	0.39	0.25	0.63	0.64	-0.31	0.40	0.29	0.64	0.69	-0.31	0.40	0.28	0.63	0.66
Fusicatenibacter	0.52	0.14	0.58	0.42	0.87	0.48	0.17	0.48	0.38	0.72	0.52	0.15	0.27	0.41	0.64	0.55	0.13	0.26	0.38	0.68	0.54	0.14	0.22	0.39	0.65
Fusobacterium	-0.25	0.51	0.72	0.70	0.92	-0.13	0.71	0.67	0.86	0.88	-0.05	0.90	0.44	0.92	0.68	-0.07	0.86	0.45	0.91	0.71	-0.08	0.83	0.38	0.90	0.66
GCA.900066575	0.55	0.19	0.48	0.46	0.80	0.37	0.35	0.44	0.55	0.72	0.35	0.41	0.47	0.63	0.68	0.34	0.42	0.42	0.64	0.71	0.30	0.48	0.43	0.70	0.66
Gordonibacter	-0.30	0.52	0.17	0.70	0.63	-0.43	0.34	0.13	0.55	0.55	-0.26	0.58	0.21	0.76	0.64	-0.29	0.54	0.23	0.73	0.67	-0.30	0.53	0.24	0.73	0.66
Granulicatella	-0.12	0.85	0.90	0.92	0.99	0.02	0.98	0.93	0.98	0.99	0.13	0.83	0.82	0.89	0.92	0.09	0.89	0.91	0.92	0.96	0.12	0.85	0.98	0.90	0.99
Haemophilus	0.61	0.04	0.24	0.32	0.71	0.58	0.04	0.14	0.28	0.58	0.56	0.07	0.08	0.34	0.42	0.55	0.08	0.08	0.33	0.45	0.55	0.08	0.10	0.32	0.45
Harryflintia	-0.70	0.15	0.13	0.42	0.56	-0.86	0.07	0.07	0.31	0.40	-1.01	0.05	0.06	0.34	0.38	-1.02	0.05	0.06	0.33	0.42	-1.05	0.04	0.06	0.31	0.42
Holdemanella	0.41	0.15	0.08	0.42	0.50	0.43	0.12	0.09	0.35	0.46	0.53	0.07	0.08	0.34	0.42	0.52	0.08	0.09	0.33	0.45	0.51	0.08	0.09	0.32	0.45
Holdemania	-1.29	0.10	0.02	0.42	0.27	-1.18	0.12	0.02	0.35	0.28	-1.29	0.12	0.03	0.36	0.32	-1.34	0.10	0.03	0.36	0.32	-1.35	0.10	0.03	0.36	0.34
Hungatella	-1.45	0.00	0.02	0.03	0.27	-1.43	0.00	0.01	0.01	0.17	-1.47	0.00	0.01	0.02	0.13	-1.49	0.00	0.01	0.02	0.15	-1.48	0.00	0.00	0.02	0.12
Hydrogenoanaerobacterium	0.18	0.72	0.79	0.83	0.94	-0.11	0.82	0.67	0.91	0.88	-0.37	0.48	0.45	0.67	0.68	-0.37	0.47	0.50	0.67	0.71	-0.40	0.45	0.52	0.69	0.72
Incertae_Sedis	-0.72	0.30	0.28	0.55	0.71	-0.61	0.37	0.32	0.55	0.71	-0.55	0.45	0.36	0.66	0.68	-0.55	0.45	0.37	0.65	0.71	-0.53	0.47	0.40	0.70	0.66
Intestinibacter	0.17	0.61	0.21	0.75	0.69	0.29	0.37	0.18	0.55	0.58	0.22	0.53	0.26	0.72	0.64	0.27	0.45	0.24	0.65	0.67	0.25	0.48	0.28	0.70	0.66
Intestinimonas	-0.35	0.35	0.01	0.58	0.27	-0.66	0.07	0.00	0.31	0.09	-0.80	0.04	0.00	0.34	0.13	-0.80	0.04	0.00	0.33	0.15	-0.83	0.03	0.00	0.31	0.09
Lachnoclostridium	-1.13	0.14	0.67	0.42	0.90	-0.98	0.19	0.46	0.41	0.72	-0.74	0.34	0.42	0.57	0.68	-0.69	0.37	0.42	0.61	0.71	-0.67	0.39	0.41	0.62	0.66
Lachnospira	1.06	0.01	0.37	0.16	0.72	0.79	0.04	0.40	0.28	0.71	0.51	0.21	0.63	0.44	0.83	0.56	0.17	0.56	0.42	0.76	0.53	0.19	0.65	0.44	0.79
Lachnospiraceae_FCS020_group	0.54	0.15	0.60	0.42	0.88	0.37	0.30	0.76	0.54	0.92	0.46	0.24	0.94	0.45	0.97	0.46	0.24	0.93	0.48	0.96	0.45	0.25	1.00	0.47	1.00
Lachnospiraceae_ND3007_group	0.62	0.02	0.32	0.19	0.72	0.50	0.05	0.31	0.28	0.71	0.55	0.04	0.14	0.34	0.53	0.56	0.04	0.15	0.33	0.55	0.55	0.04	0.15	0.31	0.57
Lachnospiraceae_NK4A136_group	0.28	0.45	0.69	0.65	0.90	0.06	0.87	0.65	0.92	0.88	-0.16	0.68	0.67	0.82	0.83	-0.16	0.68	0.68	0.82	0.85	-0.19	0.63	0.61	0.79	0.79
Lachnospiraceae_UCG.001	0.04	0.88	0.18	0.92	0.67	-0.03	0.90	0.16	0.92	0.58	-0.12	0.68	0.22	0.82	0.64	-0.12	0.69	0.25	0.82	0.67	-0.13	0.66	0.24	0.80	0.66
Lachnospiraceae_UCG.004	0.31	0.36	0.66	0.58	0.90	0.14	0.67	0.93	0.84	0.99	0.16	0.65	0.63	0.82	0.83	0.18	0.61	0.62	0.79	0.81	0.18	0.59	0.56	0.77	0.75
Lachnospiraceae_UCG.010	-0.68	0.13	0.39	0.42	0.72	-0.87	0.05	0.34	0.28	0.71	-0.81	0.08	0.47	0.34	0.68	-0.85	0.06	0.45	0.33	0.71	-0.88	0.06	0.39	0.31	0.66
Lactobacillus	-0.48	0.23	0.67	0.46	0.90	-0.26	0.49	0.69	0.68	0.89	-0.31	0.46	0.65	0.66	0.83	-0.33	0.43	0.55	0.64	0.75	-0.29	0.50	0.59	0.70	0.78
Lactococcus	0.93	0.05	0.30	0.33	0.71	0.73	0.12	0.47	0.35	0.72	0.81	0.10	0.38	0.36	0.68	0.79	0.11	0.38	0.36	0.71	0.81	0.10	0.33	0.36	0.66
Marvinbryantia	0.24	0.42	0.23	0.63	0.71	0.10	0.72	0.30	0.86	0.71	0.15	0.63	0.29	0.81	0.67	0.14	0.65	0.29	0.82	0.69	0.13	0.67	0.37	0.80	0.66
Megasphaera	-0.18	0.42	0.93	0.63	0.99	-0.04	0.85	0.94	0.92	0.99	0.06	0.81	0.95	0.88	0.97	0.05	0.85	0.97	0.91	0.98	0.05	0.83	0.97	0.90	0.99

© 2022 Meyer K et al. *JAMA Network Open.*

Monoglobus	0.34	0.35	0.40	0.58	0.72	0.22	0.52	0.36	0.72	0.71	0.12	0.76	0.39	0.88	0.68	0.12	0.76	0.41	0.87	0.71	0.13	0.74	0.45	0.85	0.66
Moryella	0.88	0.08	0.44	0.39	0.78	0.73	0.13	0.36	0.35	0.71	0.65	0.20	0.34	0.44	0.68	0.64	0.21	0.36	0.46	0.71	0.63	0.22	0.37	0.46	0.66
Negativibacillus	-0.45	0.19	0.19	0.46	0.67	-0.54	0.10	0.07	0.35	0.40	-0.66	0.06	0.04	0.34	0.34	-0.65	0.07	0.05	0.33	0.35	-0.66	0.06	0.05	0.32	0.37
NK4A214_group	-0.13	0.60	0.08	0.75	0.50	-0.28	0.27	0.04	0.52	0.40	-0.39	0.15	0.05	0.41	0.34	-0.40	0.14	0.05	0.38	0.35	-0.43	0.11	0.04	0.37	0.34
Odoribacter	0.50	0.05	0.39	0.33	0.72	0.48	0.05	0.28	0.28	0.71	0.42	0.12	0.27	0.36	0.64	0.42	0.12	0.25	0.36	0.67	0.41	0.13	0.25	0.39	0.66
Oscillibacter	-0.03	0.95	0.40	0.97	0.72	-0.11	0.78	0.40	0.90	0.71	-0.08	0.85	0.37	0.89	0.68	-0.11	0.81	0.39	0.89	0.71	-0.10	0.83	0.45	0.90	0.66
Oscillospira	-0.19	0.76	0.79	0.84	0.94	-0.27	0.65	0.96	0.82	0.99	-0.36	0.56	0.79	0.75	0.92	-0.37	0.54	0.79	0.73	0.91	-0.36	0.56	0.77	0.74	0.89
Oxalobacter	0.69	0.10	0.63	0.42	0.90	0.45	0.27	0.58	0.52	0.81	0.35	0.42	0.71	0.63	0.86	0.35	0.43	0.71	0.64	0.86	0.30	0.50	0.76	0.70	0.89
Paludicola	-0.26	0.54	0.43	0.71	0.76	-0.63	0.13	0.19	0.35	0.59	-0.87	0.05	0.13	0.34	0.50	-0.92	0.04	0.14	0.33	0.55	-0.93	0.04	0.14	0.31	0.55
Parabacteroides	0.45	0.14	0.50	0.42	0.80	0.57	0.05	0.43	0.28	0.72	0.65	0.04	0.27	0.34	0.64	0.62	0.05	0.34	0.33	0.71	0.62	0.05	0.38	0.31	0.66
Paraprevotella	0.07	0.76	0.38	0.84	0.72	0.12	0.55	0.36	0.73	0.71	0.10	0.65	0.42	0.82	0.68	0.08	0.71	0.46	0.82	0.71	0.11	0.63	0.40	0.79	0.66
Parasutterella	0.58	0.01	0.26	0.17	0.71	0.58	0.01	0.18	0.16	0.58	0.50	0.04	0.26	0.34	0.64	0.52	0.04	0.25	0.33	0.67	0.50	0.04	0.21	0.31	0.65
Peptococcus	-0.55	0.18	0.01	0.46	0.27	-0.75	0.06	0.01	0.29	0.17	-0.79	0.06	0.02	0.34	0.32	-0.82	0.05	0.02	0.33	0.32	-0.84	0.05	0.02	0.31	0.31
Phascolarctobacterium	0.27	0.21	0.74	0.46	0.93	0.26	0.21	0.85	0.43	0.94	0.31	0.17	0.74	0.41	0.89	0.30	0.18	0.74	0.42	0.88	0.28	0.21	0.78	0.46	0.89
Phocea	-0.85	0.11	0.53	0.42	0.80	-1.05	0.04	0.33	0.28	0.71	-1.40	0.01	0.11	0.17	0.49	-1.39	0.01	0.11	0.17	0.48	-1.38	0.01	0.09	0.16	0.45
Prevotella	0.06	0.72	0.92	0.83	0.99	0.11	0.54	0.83	0.73	0.94	0.08	0.68	0.70	0.82	0.86	0.07	0.71	0.66	0.82	0.84	0.07	0.72	0.63	0.84	0.79
Romboutsia	0.42	0.21	0.48	0.46	0.80	0.52	0.11	0.39	0.35	0.71	0.43	0.22	0.46	0.44	0.68	0.45	0.20	0.45	0.43	0.71	0.48	0.17	0.40	0.42	0.66
Roseburia	1.18	0.01	0.02	0.16	0.30	0.97	0.02	0.06	0.28	0.40	0.67	0.16	0.20	0.41	0.64	0.69	0.15	0.19	0.38	0.65	0.69	0.15	0.23	0.39	0.66
Ruminococcus	0.43	0.11	0.95	0.42	0.99	0.35	0.18	0.79	0.39	0.92	0.30	0.29	0.66	0.51	0.83	0.30	0.29	0.63	0.51	0.81	0.30	0.29	0.58	0.49	0.77
Sellimonas	0.35	0.34	0.97	0.58	1.00	0.40	0.26	0.59	0.52	0.82	0.45	0.23	0.42	0.45	0.68	0.43	0.25	0.40	0.48	0.71	0.44	0.24	0.38	0.47	0.66
Senegalimassilia	0.48	0.15	0.38	0.42	0.72	0.47	0.14	0.41	0.37	0.72	0.54	0.12	0.33	0.36	0.68	0.51	0.14	0.38	0.38	0.71	0.51	0.14	0.37	0.39	0.66
Shuttleworthia	-0.25	0.56	0.24	0.72	0.71	-0.41	0.33	0.36	0.55	0.71	-0.61	0.18	0.24	0.42	0.64	-0.59	0.19	0.29	0.43	0.69	-0.56	0.22	0.31	0.46	0.66
Slackia	-0.04	0.89	0.79	0.92	0.94	0.04	0.88	0.65	0.92	0.88	0.09	0.78	0.59	0.88	0.80	0.06	0.84	0.69	0.91	0.85	0.03	0.91	0.65	0.93	0.79
Streptococcus	0.46	0.31	0.49	0.55	0.80	0.45	0.31	0.48	0.55	0.72	0.62	0.20	0.40	0.44	0.68	0.56	0.26	0.49	0.48	0.71	0.60	0.22	0.45	0.46	0.66
Subdoligranulum	0.77	0.03	0.94	0.24	0.99	0.68	0.04	0.97	0.28	0.99	0.56	0.12	0.99	0.36	0.99	0.54	0.13	0.96	0.38	0.98	0.53	0.14	0.88	0.39	0.94
Sutterella	0.09	0.65	0.29	0.80	0.71	0.17	0.39	0.33	0.57	0.71	0.27	0.20	0.12	0.44	0.50	0.26	0.22	0.14	0.46	0.55	0.27	0.20	0.14	0.45	0.55
TM7x	0.08	0.86	0.52	0.92	0.80	-0.04	0.93	0.47	0.94	0.72	0.12	0.81	0.38	0.88	0.68	0.10	0.84	0.40	0.91	0.71	0.10	0.84	0.43	0.90	0.66
Turicibacter	1.35	0.00	0.00	0.09	0.27	1.58	0.00	0.00	0.01	0.09	1.60	0.00	0.00	0.02	0.13	1.63	0.00	0.00	0.02	0.15	1.66	0.00	0.00	0.02	0.09
Tuzzerella	0.05	0.89	0.69	0.92	0.90	0.06	0.87	0.55	0.92	0.78	-0.07	0.85	0.42	0.89	0.68	-0.06	0.87	0.46	0.92	0.71	-0.04	0.90	0.51	0.93	0.71
UBA1819	-1.13	0.02	0.02	0.21	0.27	-1.26	0.01	0.01	0.15	0.17	-1.30	0.01	0.01	0.17	0.13	-1.35	0.01	0.01	0.17	0.15	-1.35	0.01	0.00	0.16	0.12
UCG.002	-0.09	0.73	0.26	0.83	0.71	-0.23	0.36	0.19	0.55	0.59	-0.25	0.35	0.33	0.57	0.68	-0.27	0.32	0.33	0.54	0.71	-0.30	0.27	0.28	0.49	0.66
UCG.003	0.41	0.13	0.63	0.42	0.90	0.26	0.33	0.71	0.55	0.91	0.21	0.47	0.65	0.66	0.83	0.19	0.50	0.69	0.70	0.85	0.18	0.53	0.65	0.73	0.79
UCG.005	0.22	0.46	0.50	0.65	0.80	0.13	0.66	0.53	0.84	0.77	0.04	0.90	0.61	0.92	0.82	0.01	0.96	0.61	0.96	0.80	0.00	1.00	0.63	1.00	0.79
UCG.009	-0.29	0.52	0.37	0.70	0.72	-0.45	0.30	0.49	0.54	0.72	-0.59	0.20	0.44	0.44	0.68	-0.61	0.19	0.44	0.42	0.71	-0.64	0.17	0.42	0.42	0.66
Veillonella	0.59	0.06	0.03	0.33	0.36	0.62	0.04	0.02	0.28	0.22	0.61	0.06	0.02	0.34	0.32	0.61	0.06	0.02	0.33	0.32	0.62	0.06	0.03	0.31	0.33
Victivallis	0.21	0.48	0.86	0.66	0.96	0.12	0.68	0.94	0.84	0.99	0.02	0.94	0.92	0.95	0.97	0.02	0.94	0.85	0.95	0.94	0.02	0.95	0.89	0.96	0.94

Multivariable-adjusted regression models adjusted for:

Model 1: sequencing batch, age, race, gender, study center

Model 2: Model 1, with education

Model 3: Model 2, with physical activity, diet score, medication use, and smoking

Model 4: Model 3 with BMI

Model 5: Model 4 with hypertension and diabetes

© 2022 Meyer K et al. *JAMA Network Open.*

eTable 44: Multivariable-adjusted regression coefficients, p-values, sex interaction p-values, false discovery rate (FDR)-adjusted p-values for sex-genera interaction with respect to letter fluency.

	Model1					Model2					Model3					Model4					Model5				
	beta	p-value	p-val int	FDR	FDR-int	beta	p-value	p-val int	FDR	FDR-int	beta	p-value	p-val int	FDR	FDR-int	beta	p-value	p-val int	FDR	FDR-int	beta	p-value	p-val int	FDR	FDR-int
Acetanaerobacterium	1.50	0.38	0.42	0.89	0.94	1.49	0.38	0.60	0.95	0.91	1.33	0.45	0.74	0.98	0.98	1.33	0.45	0.72	0.97	0.95	1.33	0.45	0.66	0.98	0.98
Acidaminococcus	-0.94	0.06	0.49	0.55	0.94	-0.69	0.15	0.47	0.95	0.91	-0.59	0.25	0.43	0.98	0.95	-0.59	0.26	0.42	0.97	0.94	-0.57	0.27	0.44	0.98	0.93
Actinomyces	1.06	0.45	0.28	0.89	0.94	1.12	0.42	0.33	0.95	0.91	0.80	0.59	0.90	0.98	0.98	0.69	0.64	0.97	0.97	1.00	0.73	0.63	0.97	0.98	1.00
Adlercreutzia	-0.59	0.46	0.82	0.89	0.94	-0.53	0.50	0.61	0.95	0.92	-0.43	0.60	0.44	0.98	0.95	-0.40	0.63	0.39	0.97	0.94	-0.41	0.62	0.36	0.98	0.92
Agathobacter	0.75	0.54	0.55	0.89	0.94	0.44	0.72	0.73	0.95	0.93	0.07	0.96	0.76	0.98	0.98	0.14	0.91	0.74	0.97	0.95	0.10	0.93	0.83	0.98	0.98
Akkermansia	-0.23	0.69	0.51	0.92	0.94	-0.56	0.31	0.33	0.95	0.91	-0.65	0.27	0.37	0.98	0.95	-0.63	0.29	0.40	0.97	0.94	-0.66	0.27	0.42	0.98	0.93
Alistipes	1.76	0.08	0.24	0.58	0.94	1.24	0.21	0.43	0.95	0.91	0.85	0.42	0.43	0.98	0.95	0.88	0.40	0.41	0.97	0.94	0.84	0.42	0.41	0.98	0.93
Allisonella	-0.19	0.83	0.63	0.94	0.94	0.44	0.62	0.47	0.95	0.91	0.71	0.46	0.61	0.98	0.95	0.68	0.48	0.55	0.97	0.95	0.72	0.46	0.52	0.98	0.93
Anaerofilum	0.49	0.70	0.79	0.92	0.94	0.06	0.96	0.96	0.97	0.98	0.25	0.85	0.83	0.98	0.98	0.25	0.85	0.80	0.97	0.95	0.23	0.86	0.76	0.98	0.98
Anaerostipes	1.29	0.35	0.09	0.89	0.90	0.83	0.54	0.12	0.95	0.91	1.93	0.18	0.03	0.98	0.54	2.00	0.16	0.03	0.97	0.52	1.93	0.18	0.04	0.98	0.57
Anaerotruncus	-0.66	0.56	0.59	0.89	0.94	-1.07	0.34	0.43	0.95	0.91	-0.70	0.55	0.71	0.98	0.98	-0.76	0.51	0.72	0.97	0.95	-0.81	0.49	0.71	0.98	0.98
Bacteroides	1.46	0.40	0.25	0.89	0.94	1.21	0.47	0.23	0.95	0.91	1.27	0.46	0.20	0.98	0.88	1.36	0.43	0.20	0.97	0.88	1.36	0.43	0.19	0.98	0.84
Barnesiella	1.14	0.03	0.77	0.48	0.94	1.19	0.02	0.70	0.71	0.93	0.79	0.14	0.78	0.98	0.98	0.79	0.14	0.78	0.97	0.95	0.81	0.13	0.78	0.98	0.98
Bifidobacterium	-0.09	0.89	0.99	0.95	1.00	0.24	0.71	0.99	0.95	0.99	0.01	0.99	0.74	0.99	0.98	0.03	0.97	0.74	0.98	0.95	0.00	1.00	0.72	1.00	0.98
Bilophila	0.86	0.25	0.77	0.89	0.94	0.47	0.52	0.50	0.95	0.91	0.83	0.29	0.98	0.98	0.99	0.88	0.26	0.97	0.97	1.00	0.89	0.26	0.87	0.98	0.98
Blautia	0.52	0.83	0.17	0.94	0.94	-0.70	0.77	0.31	0.95	0.91	0.15	0.95	0.22	0.98	0.88	0.18	0.94	0.20	0.98	0.88	0.14	0.95	0.25	0.98	0.84
Butyricicoccus	1.50	0.41	0.37	0.89	0.94	0.39	0.83	0.47	0.95	0.91	0.67	0.72	0.22	0.98	0.88	0.71	0.70	0.21	0.97	0.88	0.64	0.73	0.24	0.98	0.84
Butyricimonas	0.30	0.63	0.67	0.92	0.94	0.09	0.88	0.55	0.96	0.91	0.07	0.91	0.58	0.98	0.95	0.07	0.91	0.57	0.97	0.95	0.03	0.96	0.54	0.98	0.93
CAG.56	0.31	0.64	0.86	0.92	0.95	0.05	0.94	0.72	0.97	0.93	-0.04	0.95	0.53	0.98	0.95	-0.04	0.95	0.52	0.98	0.95	-0.05	0.94	0.51	0.98	0.93
Candidatus_Soleaferrea	0.55	0.68	0.40	0.92	0.94	0.01	0.99	0.55	0.99	0.91	0.33	0.81	0.50	0.98	0.95	0.34	0.80	0.48	0.97	0.95	0.31	0.82	0.46	0.98	0.93
Caproiciproducens	1.42	0.25	0.32	0.89	0.94	1.27	0.29	0.40	0.95	0.91	1.12	0.37	0.29	0.98	0.95	1.13	0.37	0.28	0.97	0.94	1.14	0.37	0.24	0.98	0.84
Christensenellaceae_R.7_group	0.39	0.51	0.71	0.89	0.94	0.18	0.75	0.74	0.95	0.93	0.01	0.99	0.92	0.99	0.98	0.00	1.00	0.95	1.00	1.00	0.00	1.00	0.97	1.00	1.00
Clostridium_sensu_stricto_1	0.64	0.36	0.39	0.89	0.94	0.84	0.22	0.27	0.95	0.91	0.98	0.17	0.21	0.98	0.88	0.98	0.17	0.21	0.97	0.88	0.99	0.17	0.14	0.98	0.84
Colidextribacter	-0.26	0.85	0.56	0.94	0.94	-0.85	0.53	0.39	0.95	0.91	-0.81	0.57	0.69	0.98	0.98	-0.79	0.57	0.72	0.97	0.95	-0.85	0.54	0.60	0.98	0.95
Collinsella	0.54	0.43	0.15	0.89	0.94	0.74	0.27	0.27	0.95	0.91	0.76	0.28	0.20	0.98	0.88	0.75	0.29	0.22	0.97	0.88	0.77	0.28	0.17	0.98	0.84
Coprobacter	1.87	0.01	0.06	0.45	0.90	1.71	0.01	0.04	0.71	0.91	1.48	0.03	0.03	0.89	0.54	1.47	0.04	0.03	0.91	0.52	1.49	0.03	0.02	0.91	0.57
Coprococcus	2.02	0.02	0.06	0.48	0.90	1.57	0.05	0.20	0.80	0.91	1.54	0.07	0.21	0.89	0.88	1.53	0.07	0.22	0.91	0.88	1.47	0.08	0.26	0.91	0.84
Defluviitaleaceae_UCG.011	0.65	0.57	0.74	0.89	0.94	0.67	0.55	0.51	0.95	0.91	1.04	0.38	0.29	0.98	0.95	1.05	0.38	0.27	0.97	0.94	1.03	0.38	0.22	0.98	0.84
Desulfovibrio	-0.23	0.70	0.91	0.92	0.96	-0.19	0.74	0.86	0.95	0.96	-0.29	0.65	0.84	0.98	0.98	-0.35	0.58	0.80	0.97	0.95	-0.35	0.58	0.78	0.98	0.98
Dialister	-0.14	0.78	0.55	0.94	0.94	-0.11	0.83	0.52	0.95	0.91	0.23	0.66	0.36	0.98	0.95	0.25	0.63	0.35	0.97	0.94	0.23	0.67	0.40	0.98	0.93
DNF00809	0.03	0.98	0.52	0.99	0.94	-0.22	0.86	0.55	0.95	0.91	0.24	0.86	0.27	0.98	0.95	0.19	0.89	0.28	0.97	0.94	0.21	0.87	0.26	0.98	0.84
Dorea	0.82	0.45	0.25	0.89	0.94	0.62	0.56	0.42	0.95	0.91	1.34	0.21	0.12	0.98	0.88	1.32	0.22	0.13	0.97	0.88	1.34	0.22	0.12	0.98	0.84
DTU089	1.71	0.10	0.07	0.60	0.90	1.65	0.10	0.06	0.80	0.91	1.67	0.11	0.04	0.89	0.54	1.63	0.12	0.04	0.97	0.52	1.66	0.11	0.05	0.92	0.59
Eggerthella	0.06	0.95	0.18	0.99	0.94	-0.22	0.82	0.24	0.95	0.91	0.23	0.82	0.22	0.98	0.88	0.21	0.83	0.22	0.97	0.88	0.18	0.86	0.28	0.98	0.85
Eisenbergiella	-1.75	0.08	0.28	0.58	0.94	-1.79	0.07	0.24	0.80	0.91	-1.75	0.09	0.38	0.89	0.95	-1.80	0.08	0.39	0.91	0.94	-1.87	0.07	0.28	0.91	0.84
Erysipelatoclostridium	-0.66	0.50	0.73	0.89	0.94	-0.58	0.55	0.79	0.95	0.96	-0.88	0.38	0.57	0.98	0.95	-0.90	0.37	0.59	0.97	0.95	-0.93	0.35	0.54	0.98	0.93
Erysipelotrichaceae_UCG.003	0.62	0.41	0.69	0.89	0.94	0.53	0.47	0.72	0.95	0.93	0.30	0.69	0.73	0.98	0.98	0.32	0.68	0.70	0.97	0.95	0.34	0.66	0.59	0.98	0.95
Escherichia.Shigella	-0.29	0.67	0.50	0.92	0.94	0.03	0.96	0.39	0.97	0.91	-0.14	0.85	0.74	0.98	0.98	-0.12	0.86	0.74	0.97	0.95	-0.19	0.79	0.82	0.98	0.98
Faecalibacterium	0.55	0.64	0.99	0.92	1.00	0.61	0.60	0.75	0.95	0.93	0.38	0.76	0.80	0.98	0.98	0.38	0.76	0.81	0.97	0.95	0.37	0.77	0.80	0.98	0.98
Family_XIII_AD3011_group	1.40	0.15	0.42	0.70	0.94	0.72	0.45	0.67	0.95	0.93	0.70	0.49	0.51	0.98	0.95	0.65	0.53	0.51	0.97	0.95	0.60	0.56	0.51	0.98	0.93
Family_XIII_UCG.001	0.69	0.53	0.78	0.89	0.94	0.39	0.72	0.74	0.95	0.93	0.38	0.74	0.91	0.98	0.98	0.32	0.78	0.90	0.97	0.98	0.31	0.79	0.91	0.98	0.99
FD2005	-1.99	0.05	0.02	0.49	0.90	-1.80	0.06	0.02	0.80	0.61	-1.67	0.10	0.03	0.89	0.54	-1.63	0.11	0.03	0.97	0.52	-1.67	0.10	0.03	0.92	0.57
Flavonifractor	0.08	0.93	0.12	0.98	0.94	0.09	0.91	0.11	0.97	0.91	0.28	0.76	0.10	0.98	0.88	0.28	0.76	0.10	0.97	0.82	0.25	0.78	0.11	0.98	0.84
Fournierella	-0.02	0.98	0.83	0.99	0.94	-0.05	0.96	0.87	0.97	0.96	-0.54	0.59	0.69	0.98	0.98	-0.56	0.57	0.71	0.97	0.95	-0.55	0.58	0.70	0.98	0.98
Frisingicoccus	0.00	1.00	0.92	1.00	0.96	0.16	0.85	0.71	0.95	0.93	0.23	0.80	0.44	0.98	0.95	0.25	0.78	0.41	0.97	0.94	0.28	0.75	0.41	0.98	0.93
Fusicatenibacter	0.29	0.74	0.99	0.93	1.00	0.19	0.82	0.87	0.95	0.96	0.57	0.52	0.33	0.98	0.95	0.60	0.50	0.32	0.97	0.94	0.60	0.50	0.27	0.98	0.84
Fusobacterium	0.21	0.82	0.21	0.94	0.94	0.44	0.62	0.19	0.95	0.91	0.40	0.67	0.06	0.98	0.73	0.37	0.69	0.06	0.97	0.74	0.32	0.73	0.05	0.98	0.59
GCA.900066575	0.95	0.36	0.87	0.89	0.95	0.56	0.57	0.89	0.95	0.96	0.50	0.63	0.89	0.98	0.98	0.48	0.64	0.85	0.97	0.97	0.41	0.69	0.78	0.98	0.98
Gordonibacter	0.83	0.48	0.78	0.89	0.94	0.49	0.67	0.86	0.95	0.96	0.76	0.52	0.58	0.98	0.95	0.73	0.54	0.57	0.97	0.95	0.76	0.52	0.53	0.98	0.93
Granulicatella	-1.30	0.39	0.24	0.89	0.94	-1.02	0.48	0.30	0.95	0.91	-1.12	0.47	0.16	0.98	0.88	-1.19	0.45	0.14	0.97	0.88	-1.18	0.45	0.11	0.98	0.84
Haemophilus	0.46	0.53	1.00	0.89	1.00	0.41	0.57	0.83	0.95	0.96	0.20	0.79	0.99	0.98	0.99	0.20	0.80	0.99	0.97	1.00	0.20	0.79	0.94	0.98	1.00
Harryflintia	-0.40	0.75	0.63	0.93	0.94	-0.77	0.52	0.82	0.95	0.96	-0.74	0.56	0.69	0.98	0.98	-0.75	0.55	0.68	0.97	0.95	-0.81	0.52	0.58	0.98	0.95
Holdemanella	0.23	0.74	0.83	0.93	0.94	0.29	0.67	0.95	0.95	0.98	0.77	0.28	0.76	0.98	0.98	0.76	0.29	0.80	0.97	0.95	0.80	0.27	0.80	0.98	0.98
Holdemania	-1.53	0.44	0.29	0.89	0.94	-1.31	0.50	0.36	0.95	0.91	-0.21	0.92	0.78	0.98	0.98	-0.27	0.90	0.78	0.97	0.95	-0.15	0.94	0.99	0.98	1.00
Hungatella	-2.28	0.02	0.20	0.48	0.94	-2.24	0.02	0.14	0.71	0.91	-2.71	0.01	0.04	0.72	0.55	-2.74	0.01	0.04	0.66	0.52	-2.80	0.01	0.02	0.56	0.57
Hydrogenoanaerobacterium	2.17	0.08	0.49	0.58	0.94	1.54	0.20	0.58	0.95	0.91	0.80	0.53	0.98	0.98	0.99	0.79	0.53	1.00	0.97	1.00	0.71	0.58	0.97	0.98	1.00
Incertae_Sedis	2.70	0.12	0.10	0.68	0.90	2.93	0.08	0.08	0.80	0.91	3.21	0.07	0.07	0.89	0.76	3.21	0.07	0.07	0.91	0.74	3.30	0.06	0.06	0.91	0.66
Intestinibacter	0.15	0.86	0.81	0.94	0.94	0.42	0.60	0.78	0.95	0.96	0.25	0.77	0.90	0.98	0.98	0.31	0.72	0.88	0.97	0.97	0.26	0.76	0.98	0.98	1.00
Intestinimonas	0.87	0.35	0.55	0.89	0.94	0.17	0.85	0.88	0.95	0.96	0.13	0.89	0.82	0.98	0.98	0.14	0.88	0.77	0.97	0.95	0.11	0.91	0.92	0.98	1.00
Lachnoclostridium	-2.46	0.19	0.77	0.82	0.94	-2.08	0.26	0.59	0.95	0.91	-1.08	0.57	0.60	0.98	0.95	-1.03	0.59	0.60	0.97	0.95	-1.02	0.59	0.55	0.98	0.93
Lachnospira	1.63	0.09	0.53	0.58	0.94	1.01	0.28	0.56	0.95	0.91	0.66	0.51	0.71	0.98	0.98	0.72	0.47	0.67	0.97	0.95	0.65	0.51	0.76	0.98	0.98
Lachnospiraceae_FCS020_group	0.88	0.33	0.28	0.89	0.94	0.53	0.55	0.37	0.95	0.91	0.70	0.46	0.60	0.98	0.95	0.69	0.47	0.59	0.97	0.95	0.70	0.47	0.64	0.98	0.96
Lachnospiraceae_ND3007_group	1.32	0.04	0.27	0.49	0.94	1.05	0.10	0.26	0.80	0.91	1.15	0.09	0.16	0.89	0.88	1.16	0.08	0.16	0.91	0.88	1.15	0.09	0.15	0.91	0.84
Lachnospiraceae_NK4A136_group	0.80	0.39	0.61	0.89	0.94	0.29	0.75	0.63	0.95	0.93	-0.11	0.90	0.51	0.98	0.95	-0.11	0.91	0.51	0.97	0.95	-0.14	0.88	0.53	0.98	0.93
Lachnospiraceae_UCG.001	0.06	0.93	0.89	0.98	0.96	-0.11	0.87	0.91	0.95	0.97	-0.20	0.78	0.86	0.98	0.98	-0.20	0.78	0.83	0.97	0.97	-0.25	0.72	0.88	0.98	0.98
Lachnospiraceae_UCG.004	0.15	0.86	0.73	0.94	0.94	-0.24	0.77	0.49	0.95	0.91	-0.26	0.76	0.43	0.98	0.95	-0.24	0.78	0.44	0.97	0.95	-0.28	0.74	0.53	0.98	0.93
Lachnospiraceae_UCG.010	-0.94	0.40	0.31	0.89	0.94	-1.34	0.21	0.28	0.95	0.91	-1.07	0.34	0.50	0.98	0.95	-1.12	0.32	0.49	0.97	0.95	-1.15	0.31	0.48	0.98	0.93
Lactobacillus	-2.08	0.03	0.07	0.48	0.90	-1.60	0.09	0.07	0.80	0.91	-2.14	0.03	0.02	0.89	0.54	-2.19	0.03	0.02	0.91	0.52	-2.21	0.03	0.01	0.91	0.57
Lactococcus	2.06	0.08	0.16	0.58	0.94	1.60	0.17	0.27	0.95	0.91	1.70	0.16	0.35	0.98	0.95	1.68	0.17	0.35	0.97	0.94	1.70	0.16	0.27	0.98	0.84
Marvinbryantia	0.83	0.26	0.15	0.89	0.94	0.53	0.46	0.19	0.95	0.91	0.54	0.48	0.16	0.98	0.88	0.52	0.49	0.16	0.97	0.88	0.53	0.48	0.18	0.98	0.84
Megasphaera	-0.25	0.66	0.95	0.92	0.98	0.07	0.91	0.92	0.97	0.97	0.35	0.56	0.72	0.98	0.98	0.33	0.58	0.68	0.97	0.95	0.32	0.59	0.71	0.98	0.98

© 2022 Meyer K et al. *JAMA Network Open.*

Monoglobus	1.30	0.14	0.68	0.68	0.94	1.04	0.23	0.71	0.95	0.93	0.89	0.34	0.83	0.98	0.98	0.89	0.34	0.81	0.97	0.95	0.91	0.33	0.76	0.98	0.98
Moryella	0.73	0.55	0.91	0.89	0.96	0.42	0.73	0.99	0.95	0.99	0.26	0.84	0.84	0.98	0.98	0.24	0.85	0.86	0.97	0.97	0.20	0.87	0.88	0.98	0.98
Negativibacillus	-1.23	0.14	0.05	0.68	0.90	-1.44	0.08	0.02	0.80	0.61	-1.61	0.06	0.03	0.89	0.54	-1.60	0.06	0.03	0.91	0.52	-1.62	0.06	0.03	0.91	0.57
NK4A214_group	0.46	0.48	0.50	0.89	0.94	0.13	0.84	0.66	0.95	0.93	-0.07	0.92	0.60	0.98	0.95	-0.09	0.89	0.59	0.97	0.95	-0.12	0.85	0.62	0.98	0.96
Odoribacter	0.54	0.39	0.40	0.89	0.94	0.50	0.42	0.47	0.95	0.91	0.08	0.91	0.40	0.98	0.95	0.08	0.90	0.42	0.97	0.94	0.07	0.91	0.47	0.98	0.93
Oscillibacter	1.06	0.31	0.26	0.89	0.94	0.86	0.40	0.24	0.95	0.91	1.01	0.35	0.21	0.98	0.88	0.98	0.37	0.21	0.97	0.88	0.97	0.37	0.18	0.98	0.84
Oscillospira	3.09	0.04	0.08	0.48	0.90	2.89	0.04	0.12	0.80	0.91	2.79	0.06	0.13	0.89	0.88	2.77	0.06	0.13	0.91	0.88	2.79	0.06	0.14	0.91	0.84
Oxalobacter	2.75	0.01	0.02	0.45	0.90	2.19	0.03	0.02	0.80	0.61	1.93	0.07	0.02	0.89	0.54	1.93	0.07	0.02	0.91	0.52	1.89	0.08	0.03	0.91	0.57
Paludicola	2.27	0.03	0.08	0.48	0.90	1.46	0.16	0.18	0.95	0.91	1.49	0.18	0.10	0.98	0.88	1.43	0.20	0.10	0.97	0.82	1.41	0.20	0.14	0.98	0.84
Parabacteroides	0.43	0.57	0.63	0.89	0.94	0.70	0.34	0.69	0.95	0.93	0.63	0.40	0.80	0.98	0.98	0.58	0.44	0.73	0.97	0.95	0.60	0.43	0.73	0.98	0.98
Paraprevotella	-0.26	0.61	0.38	0.92	0.94	-0.13	0.80	0.38	0.95	0.91	-0.12	0.83	0.57	0.98	0.95	-0.15	0.79	0.55	0.97	0.95	-0.12	0.83	0.60	0.98	0.95
Parasutterella	0.59	0.31	0.85	0.89	0.95	0.58	0.30	0.98	0.95	0.99	0.37	0.54	0.98	0.98	0.99	0.39	0.52	1.00	0.97	1.00	0.38	0.53	0.86	0.98	0.98
Peptococcus	-0.91	0.37	0.13	0.89	0.94	-1.35	0.17	0.10	0.95	0.91	-1.45	0.16	0.31	0.98	0.95	-1.48	0.15	0.31	0.97	0.94	-1.50	0.14	0.26	0.98	0.84
Phascolarctobacterium	0.57	0.28	0.60	0.89	0.94	0.56	0.27	0.49	0.95	0.91	0.43	0.43	0.36	0.98	0.95	0.41	0.45	0.36	0.97	0.94	0.44	0.43	0.35	0.98	0.92
Phocea	-0.40	0.76	0.69	0.93	0.94	-0.85	0.50	0.48	0.95	0.91	-1.34	0.31	0.26	0.98	0.95	-1.33	0.31	0.26	0.97	0.94	-1.39	0.29	0.24	0.98	0.84
Prevotella	-0.49	0.27	0.38	0.89	0.94	-0.39	0.37	0.23	0.95	0.91	-0.44	0.33	0.34	0.98	0.95	-0.45	0.32	0.33	0.97	0.94	-0.43	0.34	0.33	0.98	0.91
Romboutsia	0.58	0.48	0.44	0.89	0.94	0.82	0.31	0.37	0.95	0.91	0.78	0.36	0.37	0.98	0.95	0.80	0.34	0.37	0.97	0.94	0.81	0.34	0.31	0.98	0.91
Roseburia	1.62	0.14	0.18	0.68	0.94	1.13	0.29	0.32	0.95	0.91	0.51	0.66	0.62	0.98	0.95	0.54	0.64	0.61	0.97	0.95	0.48	0.68	0.69	0.98	0.98
Ruminococcus	-0.43	0.52	0.26	0.89	0.94	-0.61	0.34	0.34	0.95	0.91	-0.68	0.32	0.53	0.98	0.95	-0.68	0.32	0.54	0.97	0.95	-0.70	0.31	0.56	0.98	0.93
Sellimonas	0.30	0.74	0.55	0.93	0.94	0.42	0.63	0.88	0.95	0.96	0.11	0.90	0.87	0.98	0.98	0.09	0.93	0.88	0.97	0.97	0.09	0.92	0.88	0.98	0.98
Senegalimassilia	0.23	0.78	0.43	0.94	0.94	0.21	0.79	0.36	0.95	0.91	0.06	0.94	0.30	0.98	0.95	0.02	0.98	0.27	0.99	0.94	0.02	0.98	0.26	1.00	0.84
Shuttleworthia	0.65	0.54	0.44	0.89	0.94	0.28	0.79	0.29	0.95	0.91	0.41	0.71	0.17	0.98	0.88	0.44	0.69	0.15	0.97	0.88	0.45	0.68	0.17	0.98	0.84
Slackia	-1.01	0.17	0.07	0.74	0.90	-0.81	0.25	0.08	0.95	0.91	-0.81	0.28	0.11	0.98	0.88	-0.86	0.25	0.09	0.97	0.82	-0.88	0.24	0.12	0.98	0.84
Streptococcus	-0.04	0.97	0.34	0.99	0.94	-0.09	0.93	0.35	0.97	0.91	0.20	0.87	0.57	0.98	0.95	0.12	0.92	0.62	0.97	0.95	0.12	0.92	0.63	0.98	0.96
Subdoligranulum	0.41	0.64	0.52	0.92	0.94	0.20	0.81	0.53	0.95	0.91	0.26	0.77	0.99	0.98	0.99	0.23	0.79	1.00	0.97	1.00	0.25	0.77	0.97	0.98	1.00
Sutterella	-0.10	0.85	0.83	0.94	0.94	0.09	0.86	0.93	0.95	0.98	0.25	0.63	0.55	0.98	0.95	0.23	0.65	0.58	0.97	0.95	0.27	0.60	0.53	0.98	0.93
TM7x	-1.52	0.20	0.79	0.84	0.94	-1.83	0.11	0.82	0.82	0.96	-1.27	0.29	0.90	0.98	0.98	-1.29	0.29	0.89	0.97	0.97	-1.31	0.28	0.85	0.98	0.98
Turicibacter	1.16	0.27	0.57	0.89	0.94	1.68	0.10	0.40	0.80	0.91	1.76	0.10	0.49	0.89	0.95	1.80	0.10	0.46	0.94	0.95	1.81	0.09	0.33	0.92	0.91
Tuzzerella	0.13	0.88	0.76	0.95	0.94	0.14	0.86	0.86	0.95	0.96	-0.26	0.76	0.99	0.98	0.99	-0.25	0.77	0.96	0.97	1.00	-0.23	0.78	0.84	0.98	0.98
UBA1819	-0.31	0.80	0.50	0.94	0.94	-0.63	0.60	0.36	0.95	0.91	-0.35	0.78	0.46	0.98	0.95	-0.40	0.75	0.46	0.97	0.95	-0.44	0.73	0.41	0.98	0.93
UCG.002	-0.14	0.83	0.30	0.94	0.94	-0.47	0.46	0.22	0.95	0.91	-0.65	0.32	0.42	0.98	0.95	-0.68	0.31	0.42	0.97	0.94	-0.70	0.29	0.42	0.98	0.93
UCG.003	0.65	0.33	0.65	0.89	0.94	0.31	0.63	0.58	0.95	0.91	0.17	0.80	0.66	0.98	0.98	0.15	0.83	0.64	0.97	0.95	0.13	0.85	0.73	0.98	0.98
UCG.005	1.10	0.14	0.72	0.68	0.94	0.88	0.22	0.67	0.95	0.93	0.83	0.29	0.49	0.98	0.95	0.80	0.31	0.49	0.97	0.95	0.81	0.30	0.44	0.98	0.93
UCG.009	0.58	0.60	0.57	0.92	0.94	0.21	0.85	0.45	0.95	0.91	-0.09	0.94	0.32	0.98	0.95	-0.10	0.93	0.32	0.97	0.94	-0.13	0.90	0.34	0.98	0.91
Veillonella	0.52	0.50	0.38	0.89	0.94	0.59	0.42	0.29	0.95	0.91	0.39	0.62	0.73	0.98	0.98	0.38	0.63	0.76	0.97	0.95	0.37	0.64	0.80	0.98	0.98
Victivallis	0.29	0.70	0.63	0.92	0.94	0.08	0.92	0.69	0.97	0.93	-0.17	0.82	0.93	0.98	0.98	-0.17	0.82	0.89	0.97	0.97	-0.19	0.80	1.00	0.98	1.00

Multivariable-adjusted regression models adjusted for:

Model 1: sequencing batch, age, race, gender, study center

Model 2: Model 1, with education

Model 3: Model 2, with physical activity, diet score, medication use, and smoking

Model 4: Model 3 with BMI

Model 5: Model 4 with hypertension and diabetes

© 2022 Meyer K et al. *JAMA Network Open.*

eTable 45: Multivariable-adjusted regression coefficients, p-values, sex interaction p-values, false discovery rate (FDR)-adjusted p-values for sex-genera interaction with respect to MOCA.

	Model1					Model2					Model3					Model4					Model5				
	beta	p-value	p-val int	FDR	FDR-int	beta	p-value	p-val int	FDR	FDR-int	beta	p-value	p-val int	FDR	FDR-int	beta	p-value	p-val int	FDR	FDR-int	beta	p-value	p-val int	FDR	FDR-int
Acetanaerobacterium	-0.36	0.43	0.88	0.65	0.96	-0.36	0.40	0.52	0.72	0.98	-0.74	0.09	0.16	0.38	0.49	-0.74	0.09	0.16	0.38	0.50	-0.76	0.08	0.16	0.37	0.51
Acidaminococcus	-0.24	0.07	0.61	0.26	0.94	-0.14	0.27	0.62	0.56	0.98	-0.19	0.14	0.84	0.49	0.98	-0.19	0.14	0.84	0.50	0.97	-0.19	0.15	0.76	0.49	0.94
Actinomyces	-0.10	0.80	0.89	0.92	0.96	-0.07	0.85	0.94	0.92	0.99	-0.05	0.89	0.78	0.97	0.96	-0.07	0.86	0.75	0.97	0.94	-0.04	0.92	0.67	0.97	0.90
Adlercreutzia	-0.05	0.82	0.71	0.92	0.94	-0.02	0.93	0.95	0.97	0.99	-0.07	0.74	0.95	0.95	0.98	-0.07	0.75	0.92	0.96	0.98	-0.07	0.73	0.96	0.96	0.98
Agathobacter	-0.10	0.74	0.54	0.91	0.94	-0.24	0.41	0.84	0.72	0.99	-0.26	0.38	0.80	0.72	0.97	-0.26	0.38	0.80	0.73	0.97	-0.25	0.40	0.83	0.76	0.95
Akkermansia	0.09	0.57	0.31	0.78	0.85	-0.05	0.73	0.12	0.87	0.45	-0.17	0.27	0.03	0.58	0.20	-0.17	0.27	0.03	0.59	0.21	-0.17	0.26	0.03	0.57	0.20
Alistipes	0.37	0.18	0.61	0.40	0.94	0.15	0.56	0.90	0.83	0.99	-0.06	0.82	0.52	0.97	0.88	-0.06	0.83	0.52	0.97	0.89	-0.09	0.72	0.47	0.96	0.84
Allisonella	0.11	0.64	0.00	0.82	0.17	0.36	0.12	0.01	0.38	0.14	0.28	0.25	0.03	0.57	0.21	0.28	0.25	0.03	0.56	0.22	0.28	0.25	0.02	0.57	0.18
Anaerofilum	-0.17	0.62	0.22	0.81	0.74	-0.34	0.28	0.34	0.56	0.85	-0.43	0.18	0.23	0.54	0.63	-0.43	0.18	0.23	0.53	0.63	-0.46	0.16	0.19	0.49	0.56
Anaerostipes	0.20	0.59	0.87	0.78	0.96	0.00	1.00	0.64	1.00	0.98	0.09	0.79	0.94	0.97	0.98	0.10	0.78	0.95	0.97	0.98	0.10	0.77	0.87	0.97	0.97
Anaerotruncus	-0.25	0.41	0.05	0.64	0.37	-0.41	0.15	0.02	0.43	0.16	-0.37	0.19	0.01	0.54	0.13	-0.38	0.20	0.01	0.53	0.13	-0.39	0.18	0.01	0.54	0.10
Bacteroides	-0.13	0.78	0.63	0.92	0.94	-0.23	0.60	0.62	0.83	0.98	-0.17	0.69	0.82	0.94	0.98	-0.16	0.71	0.82	0.95	0.97	-0.17	0.70	0.79	0.95	0.94
Barnesiella	0.19	0.18	0.76	0.40	0.94	0.21	0.11	0.61	0.38	0.98	0.19	0.17	0.99	0.52	0.99	0.19	0.17	0.99	0.52	0.99	0.20	0.15	0.99	0.49	0.99
Bifidobacterium	-0.19	0.29	0.59	0.53	0.94	-0.05	0.76	0.59	0.88	0.98	-0.07	0.66	0.51	0.94	0.88	-0.07	0.67	0.51	0.95	0.89	-0.07	0.67	0.56	0.94	0.87
Bilophila	0.07	0.75	0.62	0.91	0.94	-0.10	0.61	0.24	0.83	0.70	-0.31	0.12	0.05	0.46	0.23	-0.30	0.12	0.05	0.47	0.24	-0.30	0.13	0.05	0.49	0.24
Blautia	-0.46	0.49	0.40	0.70	0.94	-0.97	0.12	0.12	0.38	0.45	-0.80	0.20	0.15	0.54	0.49	-0.80	0.20	0.15	0.53	0.50	-0.79	0.21	0.08	0.57	0.33
Butyricicoccus	0.56	0.26	0.52	0.51	0.94	0.10	0.84	0.72	0.92	0.98	-0.05	0.92	0.69	0.97	0.94	-0.05	0.92	0.69	0.97	0.93	-0.08	0.86	0.74	0.97	0.94
Butyricimonas	-0.12	0.47	0.03	0.70	0.37	-0.20	0.18	0.01	0.48	0.16	-0.18	0.24	0.01	0.57	0.13	-0.18	0.23	0.01	0.56	0.13	-0.19	0.21	0.02	0.57	0.18
CAG.56	0.34	0.06	0.06	0.25	0.37	0.23	0.17	0.09	0.46	0.38	0.18	0.29	0.19	0.59	0.57	0.18	0.29	0.19	0.59	0.57	0.18	0.29	0.24	0.61	0.63
Candidatus_Soleaferrea	-0.72	0.05	0.04	0.25	0.37	-0.94	0.01	0.01	0.18	0.16	-1.07	0.00	0.00	0.11	0.05	-1.07	0.00	0.00	0.11	0.05	-1.08	0.00	0.00	0.11	0.04
Caproiciproducens	-0.47	0.16	0.13	0.39	0.57	-0.53	0.09	0.06	0.32	0.29	-0.66	0.04	0.03	0.35	0.20	-0.66	0.04	0.03	0.35	0.21	-0.67	0.04	0.02	0.36	0.18
Christensenellaceae_R.7_group	0.27	0.09	0.91	0.28	0.96	0.18	0.22	0.98	0.53	0.99	0.15	0.34	0.74	0.67	0.96	0.15	0.34	0.74	0.67	0.94	0.14	0.37	0.64	0.74	0.90
Clostridium_sensu_stricto_1	0.13	0.48	0.59	0.70	0.94	0.21	0.22	0.34	0.53	0.85	0.31	0.09	0.13	0.38	0.46	0.31	0.09	0.13	0.38	0.46	0.33	0.07	0.10	0.37	0.38
Colidextribacter	-0.22	0.56	0.86	0.78	0.96	-0.46	0.19	0.52	0.48	0.98	-0.36	0.31	0.56	0.63	0.88	-0.36	0.31	0.57	0.63	0.89	-0.36	0.31	0.45	0.62	0.84
Collinsella	0.02	0.93	0.45	0.97	0.94	0.10	0.57	0.82	0.83	0.99	0.03	0.85	0.93	0.97	0.98	0.03	0.86	0.91	0.97	0.97	0.03	0.89	0.92	0.97	0.98
Coprobacter	0.15	0.42	0.67	0.65	0.94	0.08	0.63	0.58	0.83	0.98	-0.01	0.95	0.88	0.97	0.98	-0.01	0.94	0.88	0.97	0.97	-0.02	0.91	0.95	0.97	0.98
Coprococcus	0.49	0.03	0.01	0.20	0.22	0.30	0.15	0.11	0.43	0.45	0.37	0.09	0.08	0.38	0.33	0.37	0.09	0.08	0.38	0.33	0.35	0.10	0.10	0.42	0.38
Defluviitaleaceae_UCG.011	0.10	0.74	0.31	0.91	0.85	0.11	0.69	0.55	0.87	0.98	0.02	0.93	0.34	0.97	0.75	0.02	0.94	0.34	0.97	0.74	0.01	0.96	0.36	0.97	0.75
Desulfovibrio	-0.12	0.45	0.93	0.68	0.96	-0.11	0.49	0.95	0.78	0.99	-0.11	0.50	0.74	0.83	0.96	-0.12	0.47	0.72	0.80	0.94	-0.12	0.44	0.67	0.81	0.90
Dialister	0.06	0.65	0.26	0.83	0.78	0.07	0.55	0.20	0.83	0.63	0.08	0.56	0.17	0.87	0.51	0.08	0.55	0.17	0.86	0.51	0.08	0.55	0.21	0.89	0.58
DNF00809	-0.02	0.97	0.44	0.99	0.94	-0.10	0.75	0.36	0.88	0.85	0.04	0.91	0.34	0.97	0.75	0.03	0.92	0.34	0.97	0.74	0.03	0.92	0.33	0.97	0.71
Dorea	0.24	0.40	0.32	0.64	0.86	0.14	0.59	0.70	0.83	0.98	0.20	0.45	0.63	0.81	0.88	0.20	0.45	0.64	0.80	0.89	0.20	0.46	0.63	0.81	0.90
DTU089	0.06	0.84	0.61	0.93	0.94	0.03	0.89	0.69	0.96	0.98	-0.07	0.78	0.30	0.97	0.72	-0.08	0.77	0.30	0.97	0.71	-0.09	0.74	0.25	0.96	0.65
Eggerthella	-0.34	0.19	0.37	0.40	0.91	-0.45	0.06	0.19	0.29	0.62	-0.29	0.25	0.42	0.57	0.85	-0.29	0.25	0.42	0.56	0.84	-0.30	0.24	0.31	0.57	0.69
Eisenbergiella	-0.58	0.03	0.00	0.20	0.17	-0.59	0.02	0.00	0.18	0.06	-0.66	0.01	0.00	0.14	0.03	-0.67	0.01	0.00	0.14	0.03	-0.66	0.01	0.00	0.16	0.03
Erysipelatoclostridium	-0.51	0.05	0.04	0.25	0.37	-0.48	0.05	0.04	0.29	0.23	-0.35	0.16	0.10	0.52	0.41	-0.36	0.16	0.10	0.51	0.42	-0.34	0.19	0.10	0.54	0.38
Erysipelotrichaceae_UCG.003	0.17	0.39	0.75	0.64	0.94	0.13	0.49	0.81	0.78	0.99	0.02	0.93	0.86	0.97	0.98	0.02	0.92	0.87	0.97	0.97	0.01	0.95	0.88	0.97	0.97
Escherichia.Shigella	-0.33	0.08	0.21	0.26	0.74	-0.19	0.27	0.30	0.56	0.79	-0.31	0.09	0.11	0.38	0.42	-0.31	0.09	0.11	0.38	0.42	-0.32	0.08	0.10	0.37	0.38
Faecalibacterium	0.44	0.16	0.73	0.39	0.94	0.45	0.13	0.41	0.39	0.91	0.59	0.06	0.15	0.38	0.49	0.59	0.06	0.15	0.38	0.50	0.59	0.06	0.15	0.37	0.50
Family_XIII_AD3011_group	0.56	0.03	0.35	0.20	0.91	0.28	0.26	0.70	0.56	0.98	0.21	0.41	0.97	0.77	0.99	0.21	0.42	0.97	0.78	0.98	0.19	0.47	0.91	0.81	0.98
Family_XIII_UCG.001	0.27	0.37	0.99	0.62	0.99	0.13	0.63	0.95	0.83	0.99	0.09	0.74	0.77	0.95	0.96	0.09	0.75	0.77	0.96	0.96	0.07	0.80	0.75	0.97	0.94
FD2005	-0.27	0.32	0.79	0.55	0.96	-0.19	0.45	0.80	0.77	0.99	-0.12	0.64	0.65	0.93	0.90	-0.11	0.66	0.64	0.95	0.89	-0.13	0.61	0.65	0.93	0.90
Flavonifractor	-0.45	0.05	0.06	0.25	0.37	-0.44	0.04	0.04	0.28	0.23	-0.39	0.09	0.03	0.38	0.22	-0.39	0.09	0.03	0.38	0.22	-0.38	0.10	0.02	0.41	0.18
Fournierella	0.28	0.28	0.36	0.53	0.91	0.26	0.28	0.28	0.56	0.77	0.32	0.20	0.29	0.54	0.70	0.32	0.20	0.28	0.53	0.69	0.31	0.22	0.30	0.57	0.69
Frisingicoccus	0.00	0.98	0.16	0.99	0.62	0.06	0.77	0.45	0.88	0.96	-0.05	0.84	0.35	0.97	0.76	-0.05	0.84	0.36	0.97	0.77	-0.05	0.84	0.31	0.97	0.69
Fusicatenibacter	0.06	0.81	0.71	0.92	0.94	0.01	0.96	0.54	0.99	0.98	-0.08	0.71	0.66	0.94	0.90	-0.08	0.71	0.66	0.95	0.90	-0.09	0.69	0.68	0.95	0.90
Fusobacterium	-0.37	0.14	0.57	0.37	0.94	-0.26	0.26	0.48	0.56	0.98	-0.17	0.47	0.62	0.81	0.88	-0.17	0.46	0.63	0.80	0.89	-0.18	0.45	0.65	0.81	0.90
GCA.900066575	0.34	0.21	0.66	0.43	0.94	0.18	0.49	0.69	0.78	0.98	0.10	0.70	0.61	0.94	0.88	0.10	0.70	0.61	0.95	0.89	0.08	0.76	0.57	0.96	0.87
Gordonibacter	-0.54	0.08	0.03	0.27	0.34	-0.66	0.02	0.01	0.19	0.16	-0.54	0.07	0.04	0.38	0.22	-0.54	0.07	0.04	0.38	0.23	-0.56	0.06	0.03	0.37	0.20
Granulicatella	-0.44	0.27	0.51	0.51	0.94	-0.32	0.39	0.70	0.72	0.98	-0.47	0.23	0.49	0.57	0.88	-0.47	0.23	0.47	0.56	0.86	-0.46	0.24	0.49	0.57	0.84
Haemophilus	0.50	0.01	0.12	0.19	0.57	0.48	0.01	0.04	0.18	0.23	0.39	0.04	0.04	0.35	0.22	0.39	0.04	0.04	0.35	0.23	0.38	0.05	0.05	0.37	0.24
Harryflintia	-0.57	0.08	0.07	0.26	0.37	-0.72	0.02	0.02	0.18	0.16	-0.84	0.01	0.00	0.11	0.06	-0.84	0.01	0.00	0.12	0.07	-0.87	0.01	0.00	0.11	0.06
Holdemanella	0.28	0.14	0.01	0.37	0.17	0.30	0.09	0.01	0.32	0.14	0.37	0.04	0.02	0.35	0.20	0.37	0.04	0.03	0.35	0.21	0.37	0.04	0.04	0.37	0.21
Holdemania	-1.30	0.01	0.02	0.19	0.33	-1.19	0.02	0.03	0.18	0.20	-1.14	0.02	0.05	0.25	0.23	-1.15	0.02	0.05	0.24	0.24	-1.16	0.02	0.05	0.23	0.25
Hungatella	-0.83	0.00	0.01	0.05	0.22	-0.81	0.00	0.00	0.07	0.12	-0.75	0.00	0.00	0.11	0.06	-0.75	0.00	0.00	0.11	0.07	-0.74	0.00	0.00	0.11	0.06
Hydrogenoanaerobacterium	0.44	0.18	0.96	0.40	0.98	0.17	0.57	0.86	0.83	0.99	-0.01	0.97	0.46	0.98	0.88	-0.01	0.97	0.47	0.98	0.86	-0.03	0.93	0.39	0.97	0.79
Incertae_Sedis	-0.76	0.10	0.05	0.30	0.37	-0.65	0.13	0.06	0.39	0.29	-0.57	0.20	0.04	0.54	0.22	-0.57	0.20	0.04	0.53	0.23	-0.56	0.20	0.03	0.57	0.20
Intestinibacter	0.26	0.24	0.48	0.48	0.94	0.37	0.07	0.41	0.29	0.91	0.40	0.06	0.33	0.38	0.75	0.41	0.06	0.32	0.38	0.72	0.42	0.06	0.38	0.37	0.78
Intestinimonas	0.05	0.83	0.22	0.92	0.74	-0.24	0.31	0.04	0.61	0.23	-0.40	0.09	0.01	0.38	0.10	-0.40	0.09	0.01	0.38	0.10	-0.43	0.07	0.00	0.37	0.06
Lachnoclostridium	-1.65	0.00	0.10	0.05	0.56	-1.51	0.00	0.03	0.07	0.20	-1.33	0.01	0.07	0.11	0.31	-1.33	0.01	0.07	0.11	0.31	-1.31	0.01	0.08	0.11	0.33
Lachnospira	0.83	0.00	0.19	0.05	0.72	0.57	0.02	0.21	0.18	0.63	0.27	0.27	0.47	0.58	0.88	0.28	0.26	0.45	0.57	0.86	0.27	0.29	0.56	0.61	0.87
Lachnospiraceae_FCS020_group	0.59	0.02	0.98	0.19	0.98	0.43	0.06	0.71	0.29	0.98	0.45	0.06	0.62	0.38	0.88	0.45	0.06	0.62	0.38	0.89	0.44	0.07	0.69	0.37	0.90
Lachnospiraceae_ND3007_group	0.42	0.02	0.04	0.19	0.37	0.30	0.07	0.03	0.29	0.20	0.33	0.05	0.02	0.37	0.20	0.33	0.05	0.02	0.37	0.21	0.32	0.06	0.03	0.37	0.20
Lachnospiraceae_NK4A136_group	0.44	0.07	0.24	0.26	0.76	0.23	0.33	0.23	0.62	0.69	0.03	0.88	0.24	0.97	0.63	0.03	0.88	0.24	0.97	0.63	0.02	0.94	0.29	0.97	0.69
Lachnospiraceae_UCG.001	0.26	0.14	0.64	0.37	0.94	0.19	0.25	0.65	0.56	0.98	0.09	0.61	0.61	0.92	0.88	0.09	0.61	0.60	0.93	0.89	0.08	0.65	0.69	0.93	0.90
Lachnospiraceae_UCG.004	0.40	0.07	0.41	0.26	0.94	0.24	0.25	0.73	0.56	0.98	0.24	0.25	0.48	0.57	0.88	0.25	0.24	0.47	0.56	0.86	0.26	0.22	0.40	0.57	0.79
Lachnospiraceae_UCG.010	0.07	0.82	0.76	0.92	0.94	-0.10	0.71	0.82	0.87	0.99	-0.11	0.68	0.71	0.94	0.94	-0.12	0.67	0.71	0.95	0.94	-0.13	0.65	0.69	0.93	0.90
Lactobacillus	-0.42	0.11	0.91	0.32	0.96	-0.22	0.37	0.95	0.70	0.99	-0.32	0.21	0.44	0.54	0.87	-0.33	0.20	0.42	0.53	0.84	-0.30	0.24	0.53	0.57	0.87
Lactococcus	0.35	0.28	0.68	0.53	0.94	0.15	0.61	0.95	0.83	0.99	0.23	0.46	0.90	0.81	0.98	0.22	0.46	0.90	0.80	0.97	0.23	0.45	0.96	0.81	0.98
Marvinbryantia	0.26	0.19	0.37	0.40	0.91	0.13	0.47	0.50	0.78	0.98	0.32	0.10	0.26	0.40	0.66	0.32	0.10	0.27	0.41	0.67	0.31	0.11	0.32	0.44	0.70
Megasphaera	-0.38	0.01	0.68	0.19	0.94	-0.25	0.08	0.64	0.32	0.98	-0.17	0.26	0.59	0.57	0.88	-0.17	0.25	0.58	0.56	0.89	-0.17	0.26	0.60	0.57	0.89

© 2022 Meyer K et al. *JAMA Network Open.*

Monoglobus	0.51	0.03	0.86	0.20	0.96	0.40	0.07	0.91	0.29	0.99	0.25	0.29	0.62	0.59	0.88	0.25	0.29	0.62	0.59	0.89	0.26	0.27	0.56	0.58	0.87
Moryella	0.70	0.03	0.16	0.20	0.62	0.57	0.07	0.09	0.29	0.39	0.45	0.15	0.23	0.49	0.63	0.45	0.15	0.24	0.51	0.63	0.45	0.16	0.27	0.49	0.67
Negativibacillus	-0.43	0.05	0.13	0.25	0.57	-0.52	0.01	0.02	0.18	0.18	-0.62	0.00	0.00	0.11	0.06	-0.62	0.00	0.00	0.11	0.07	-0.63	0.00	0.00	0.11	0.04
NK4A214_group	0.25	0.15	0.66	0.39	0.94	0.11	0.48	0.91	0.78	0.99	0.02	0.93	0.61	0.97	0.88	0.01	0.93	0.61	0.97	0.89	-0.01	0.98	0.49	0.98	0.84
Odoribacter	0.09	0.59	0.71	0.78	0.94	0.08	0.63	0.91	0.83	0.99	0.00	1.00	0.60	1.00	0.88	0.00	1.00	0.61	1.00	0.89	-0.01	0.95	0.59	0.97	0.88
Oscillibacter	0.03	0.92	0.28	0.97	0.82	-0.05	0.84	0.27	0.92	0.75	-0.13	0.63	0.13	0.92	0.46	-0.13	0.62	0.13	0.93	0.46	-0.13	0.63	0.11	0.93	0.38
Oscillospira	0.07	0.86	0.89	0.94	0.96	-0.01	0.99	0.63	1.00	0.98	-0.09	0.81	0.32	0.97	0.73	-0.09	0.81	0.32	0.97	0.72	-0.09	0.81	0.23	0.97	0.62
Oxalobacter	0.17	0.55	0.48	0.77	0.94	-0.06	0.83	0.52	0.92	0.98	-0.05	0.86	0.62	0.97	0.88	-0.05	0.86	0.62	0.97	0.89	-0.07	0.78	0.54	0.97	0.87
Paludicola	0.50	0.07	0.65	0.26	0.94	0.17	0.53	0.83	0.83	0.99	-0.01	0.97	0.42	0.98	0.85	-0.01	0.97	0.42	0.98	0.84	-0.02	0.93	0.31	0.97	0.69
Parabacteroides	0.01	0.97	0.92	0.99	0.96	0.12	0.53	0.98	0.83	0.99	0.10	0.62	0.76	0.92	0.96	0.09	0.64	0.73	0.94	0.94	0.09	0.64	0.74	0.93	0.94
Paraprevotella	-0.13	0.36	0.61	0.61	0.94	-0.08	0.56	0.60	0.83	0.98	-0.09	0.49	0.97	0.83	0.99	-0.10	0.48	0.95	0.80	0.98	-0.08	0.55	0.97	0.89	0.98
Parasutterella	0.36	0.02	0.85	0.20	0.96	0.36	0.01	0.62	0.18	0.98	0.22	0.15	0.84	0.49	0.98	0.22	0.14	0.85	0.50	0.97	0.21	0.16	0.84	0.49	0.95
Peptococcus	0.07	0.81	0.14	0.92	0.57	-0.12	0.64	0.08	0.83	0.38	-0.16	0.54	0.05	0.86	0.25	-0.16	0.53	0.05	0.86	0.25	-0.18	0.48	0.04	0.81	0.22
Phascolarctobacterium	0.02	0.89	0.23	0.97	0.75	0.01	0.93	0.13	0.97	0.47	0.02	0.89	0.15	0.97	0.49	0.02	0.90	0.15	0.97	0.50	0.01	0.92	0.19	0.97	0.56
Phocea	-0.51	0.14	0.60	0.37	0.94	-0.70	0.03	0.30	0.23	0.79	-0.94	0.00	0.12	0.11	0.45	-0.94	0.00	0.12	0.11	0.45	-0.94	0.00	0.11	0.11	0.40
Prevotella	-0.13	0.27	0.60	0.51	0.94	-0.09	0.40	0.92	0.72	0.99	-0.09	0.42	0.85	0.77	0.98	-0.09	0.41	0.86	0.78	0.97	-0.09	0.42	0.84	0.79	0.95
Romboutsia	-0.10	0.66	0.59	0.83	0.94	0.00	0.99	0.44	1.00	0.96	0.03	0.89	0.48	0.97	0.88	0.03	0.88	0.48	0.97	0.86	0.04	0.85	0.43	0.97	0.83
Roseburia	0.75	0.01	0.05	0.19	0.37	0.55	0.04	0.16	0.28	0.57	0.21	0.47	0.71	0.81	0.94	0.21	0.46	0.70	0.80	0.94	0.21	0.48	0.77	0.81	0.94
Ruminococcus	0.28	0.11	0.92	0.32	0.96	0.20	0.22	0.68	0.53	0.98	0.10	0.55	0.88	0.87	0.98	0.10	0.56	0.88	0.86	0.97	0.10	0.57	0.79	0.89	0.94
Sellimonas	0.46	0.06	0.13	0.25	0.57	0.51	0.02	0.02	0.19	0.16	0.62	0.01	0.00	0.11	0.06	0.61	0.01	0.00	0.12	0.07	0.62	0.01	0.01	0.11	0.08
Senegalimassilia	-0.06	0.77	0.89	0.92	0.96	-0.07	0.72	0.98	0.87	0.99	-0.02	0.93	0.98	0.97	0.99	-0.02	0.91	0.96	0.97	0.98	-0.02	0.93	0.96	0.97	0.98
Shuttleworthia	0.17	0.54	0.27	0.77	0.79	0.02	0.93	0.46	0.97	0.96	-0.15	0.58	0.21	0.88	0.62	-0.15	0.58	0.22	0.89	0.63	-0.14	0.62	0.20	0.93	0.57
Slackia	-0.02	0.91	0.93	0.97	0.96	0.06	0.76	0.72	0.88	0.98	0.08	0.68	0.78	0.94	0.96	0.07	0.70	0.80	0.95	0.97	0.06	0.74	0.82	0.96	0.95
Streptococcus	0.40	0.19	0.55	0.40	0.94	0.38	0.17	0.54	0.47	0.98	0.43	0.15	0.60	0.49	0.88	0.42	0.16	0.61	0.51	0.89	0.44	0.14	0.56	0.49	0.87
Subdoligranulum	0.46	0.05	0.83	0.25	0.96	0.37	0.09	0.77	0.32	0.99	0.34	0.12	0.94	0.46	0.98	0.33	0.13	0.93	0.47	0.98	0.32	0.15	0.79	0.49	0.94
Sutterella	-0.32	0.02	0.66	0.19	0.94	-0.24	0.05	0.78	0.29	0.99	-0.15	0.24	0.26	0.57	0.66	-0.15	0.23	0.27	0.56	0.67	-0.14	0.26	0.18	0.57	0.56
TM7x	0.00	0.99	0.73	0.99	0.94	-0.12	0.69	0.78	0.87	0.99	0.05	0.88	0.88	0.97	0.98	0.04	0.89	0.88	0.97	0.97	0.04	0.90	0.99	0.97	0.99
Turicibacter	0.30	0.30	0.43	0.53	0.94	0.51	0.06	0.20	0.29	0.63	0.48	0.08	0.58	0.38	0.88	0.49	0.07	0.57	0.38	0.89	0.49	0.07	0.48	0.37	0.84
Tuzzerella	-0.12	0.59	0.56	0.78	0.94	-0.11	0.59	0.37	0.83	0.87	-0.13	0.54	0.42	0.86	0.85	-0.13	0.55	0.43	0.86	0.84	-0.13	0.54	0.47	0.88	0.84
UBA1819	-0.61	0.06	0.01	0.26	0.17	-0.73	0.02	0.00	0.18	0.06	-0.76	0.01	0.00	0.17	0.03	-0.77	0.01	0.00	0.17	0.03	-0.77	0.01	0.00	0.17	0.01
UCG.002	0.18	0.31	0.51	0.54	0.94	0.04	0.79	0.36	0.89	0.85	0.06	0.72	0.52	0.94	0.88	0.06	0.73	0.52	0.95	0.89	0.04	0.80	0.44	0.97	0.84
UCG.003	0.39	0.03	0.13	0.20	0.57	0.24	0.15	0.15	0.43	0.55	0.16	0.34	0.23	0.67	0.63	0.16	0.35	0.24	0.67	0.63	0.15	0.38	0.22	0.75	0.62
UCG.005	0.28	0.16	0.92	0.39	0.96	0.19	0.32	0.99	0.62	0.99	0.12	0.53	0.89	0.86	0.98	0.12	0.54	0.89	0.86	0.97	0.11	0.57	0.82	0.89	0.95
UCG.009	0.25	0.41	0.72	0.64	0.94	0.10	0.73	0.95	0.87	0.99	-0.03	0.90	0.58	0.97	0.88	-0.04	0.90	0.58	0.97	0.89	-0.06	0.83	0.50	0.97	0.85
Veillonella	0.37	0.07	0.05	0.26	0.37	0.40	0.03	0.02	0.24	0.16	0.35	0.07	0.01	0.38	0.13	0.35	0.07	0.01	0.38	0.13	0.35	0.08	0.01	0.37	0.10
Victivallis	0.02	0.92	0.72	0.97	0.94	-0.07	0.70	0.83	0.87	0.99	-0.07	0.72	0.91	0.94	0.98	-0.07	0.72	0.89	0.95	0.97	-0.07	0.70	0.95	0.95	0.98

Multivariable-adjusted regression models adjusted for:

Model 1: sequencing batch, age, race, gender, study center

Model 2: Model 1, with education

Model 3: Model 2, with physical activity, diet score, medication use, and smoking

Model 4: Model 3 with BMI

Model 5: Model 4 with hypertension and diabetes

© 2022 Meyer K et al. *JAMA Network Open.*

eTable 46: Multivariable-adjusted regression coefficients, p-values, sex interaction p-values, false discovery rate (FDR)-adjusted p-values for sex-genera interaction with respect to PC (1st).

	Model1					Model2					Model3					Model4					Model5				
	beta	p-value	p-val int	FDR	FDR-int	beta	p-value	p-val int	FDR	FDR-int	beta	p-value	p-val int	FDR	FDR-int	beta	p-value	p-val int	FDR	FDR-int	beta	p-value	p-val int	FDR	FDR-int
Acetanaerobacterium	0.00	0.98	0.74	0.99	0.91	0.00	0.99	0.86	0.99	0.97	-0.13	0.53	0.50	0.87	0.91	-0.13	0.53	0.52	0.86	0.93	-0.14	0.51	0.55	0.84	0.95
Acidaminococcus	-0.11	0.07	0.49	0.30	0.91	-0.06	0.29	0.50	0.60	0.95	-0.06	0.35	0.84	0.81	0.99	-0.06	0.36	0.86	0.82	0.98	-0.05	0.39	0.81	0.84	0.98
Actinomyces	-0.02	0.90	0.53	0.95	0.91	-0.01	0.97	0.67	0.98	0.95	0.02	0.91	0.97	0.96	0.99	0.00	0.99	0.92	0.99	0.98	0.01	0.94	0.90	0.96	0.98
Adlercreutzia	-0.06	0.57	0.17	0.72	0.80	-0.04	0.65	0.32	0.73	0.80	-0.04	0.69	0.53	0.90	0.93	-0.04	0.72	0.59	0.89	0.97	-0.04	0.70	0.61	0.89	0.97
Agathobacter	0.15	0.31	0.17	0.51	0.80	0.08	0.55	0.32	0.72	0.80	0.01	0.97	0.37	0.99	0.80	0.02	0.90	0.36	0.96	0.79	0.01	0.92	0.43	0.96	0.87
Akkermansia	0.05	0.48	0.29	0.65	0.91	-0.02	0.80	0.10	0.84	0.52	-0.05	0.49	0.06	0.87	0.50	-0.05	0.52	0.07	0.86	0.53	-0.05	0.49	0.07	0.84	0.56
Alistipes	0.22	0.09	0.54	0.31	0.91	0.11	0.36	0.96	0.60	0.97	0.03	0.83	0.85	0.94	0.99	0.03	0.80	0.89	0.94	0.98	0.02	0.87	0.86	0.96	0.98
Allisonella	-0.03	0.81	0.05	0.86	0.74	0.10	0.36	0.08	0.60	0.52	0.09	0.45	0.23	0.87	0.73	0.09	0.46	0.26	0.86	0.75	0.09	0.44	0.25	0.84	0.72
Anaerofilum	0.00	0.99	0.10	0.99	0.75	-0.09	0.54	0.17	0.72	0.67	-0.10	0.52	0.20	0.87	0.73	-0.10	0.51	0.21	0.86	0.74	-0.11	0.49	0.20	0.84	0.72
Anaerostipes	0.07	0.67	0.71	0.78	0.91	-0.02	0.87	0.94	0.90	0.97	0.02	0.89	0.60	0.96	0.99	0.03	0.84	0.54	0.94	0.95	0.03	0.85	0.63	0.96	0.97
Anaerotruncus	-0.11	0.45	0.09	0.64	0.75	-0.19	0.16	0.03	0.50	0.51	-0.21	0.13	0.03	0.60	0.50	-0.22	0.12	0.03	0.54	0.53	-0.22	0.11	0.03	0.53	0.44
Bacteroides	0.06	0.77	0.67	0.84	0.91	0.01	0.95	0.63	0.97	0.95	0.01	0.95	0.62	0.98	0.99	0.03	0.90	0.61	0.96	0.97	0.02	0.92	0.59	0.96	0.97
Barnesiella	0.16	0.01	0.89	0.16	0.93	0.17	0.01	0.95	0.18	0.97	0.14	0.03	0.74	0.40	0.99	0.14	0.03	0.75	0.40	0.98	0.14	0.03	0.70	0.38	0.98
Bifidobacterium	-0.05	0.53	0.72	0.68	0.91	0.02	0.84	0.73	0.88	0.95	-0.01	0.92	0.61	0.96	0.99	0.00	0.96	0.62	0.97	0.97	-0.01	0.94	0.61	0.96	0.97
Bilophila	0.14	0.14	0.90	0.36	0.93	0.06	0.50	0.58	0.72	0.95	0.03	0.72	0.56	0.90	0.97	0.04	0.67	0.62	0.89	0.97	0.04	0.67	0.68	0.89	0.98
Blautia	0.10	0.75	0.77	0.83	0.91	-0.16	0.58	0.70	0.73	0.95	-0.11	0.71	0.69	0.90	0.99	-0.11	0.72	0.72	0.89	0.98	-0.11	0.72	0.55	0.89	0.95
Butyricicoccus	0.35	0.12	0.42	0.35	0.91	0.12	0.57	0.60	0.72	0.95	0.07	0.75	0.47	0.90	0.90	0.08	0.73	0.46	0.89	0.93	0.08	0.73	0.48	0.89	0.92
Butyricimonas	0.00	0.96	0.11	0.98	0.75	-0.04	0.57	0.05	0.72	0.51	-0.04	0.60	0.08	0.87	0.52	-0.04	0.60	0.07	0.86	0.53	-0.04	0.56	0.07	0.84	0.56
CAG.56	0.13	0.12	0.40	0.35	0.91	0.08	0.33	0.52	0.60	0.95	0.04	0.63	0.63	0.87	0.99	0.04	0.64	0.64	0.87	0.98	0.04	0.65	0.71	0.88	0.98
Candidatus_Soleaferrea	-0.12	0.46	0.34	0.64	0.91	-0.24	0.13	0.13	0.49	0.65	-0.27	0.10	0.07	0.55	0.50	-0.27	0.11	0.07	0.54	0.53	-0.27	0.10	0.07	0.51	0.56
Caproiciproducens	-0.16	0.31	0.16	0.51	0.80	-0.19	0.19	0.07	0.56	0.51	-0.26	0.09	0.07	0.52	0.50	-0.25	0.09	0.07	0.53	0.53	-0.25	0.10	0.08	0.51	0.56
Christensenellaceae_R.7_group	0.13	0.08	0.51	0.31	0.91	0.08	0.22	0.55	0.58	0.95	0.06	0.43	0.73	0.87	0.99	0.06	0.44	0.76	0.86	0.98	0.05	0.45	0.70	0.84	0.98
Clostridium_sensu_stricto_1	0.08	0.36	0.57	0.55	0.91	0.12	0.13	0.30	0.49	0.80	0.14	0.12	0.21	0.59	0.73	0.14	0.11	0.20	0.54	0.74	0.15	0.10	0.14	0.51	0.72
Colidextribacter	-0.20	0.25	0.41	0.47	0.91	-0.32	0.05	0.16	0.30	0.67	-0.29	0.08	0.27	0.50	0.75	-0.29	0.09	0.30	0.52	0.75	-0.29	0.09	0.22	0.51	0.72
Collinsella	0.00	0.96	0.27	0.98	0.91	0.05	0.57	0.57	0.72	0.95	0.02	0.79	0.81	0.92	0.99	0.02	0.82	0.87	0.94	0.98	0.02	0.81	0.81	0.95	0.98
Coprobacter	0.13	0.14	0.30	0.36	0.91	0.10	0.23	0.22	0.59	0.71	0.05	0.55	0.24	0.87	0.73	0.05	0.56	0.23	0.86	0.74	0.05	0.58	0.25	0.84	0.72
Coprococcus	0.29	0.01	0.02	0.11	0.54	0.20	0.04	0.18	0.30	0.67	0.19	0.06	0.17	0.45	0.73	0.19	0.06	0.18	0.48	0.74	0.18	0.07	0.23	0.50	0.72
Defluviitaleaceae_UCG.011	0.19	0.20	0.49	0.45	0.91	0.19	0.15	0.83	0.50	0.97	0.20	0.15	0.94	0.65	0.99	0.20	0.15	0.90	0.58	0.98	0.20	0.15	0.87	0.63	0.98
Desulfovibrio	-0.05	0.50	0.76	0.65	0.91	-0.04	0.54	0.72	0.72	0.95	-0.06	0.44	0.65	0.87	0.99	-0.07	0.37	0.60	0.82	0.97	-0.07	0.35	0.54	0.81	0.95
Dialister	0.02	0.76	0.37	0.83	0.91	0.03	0.65	0.30	0.73	0.80	0.03	0.62	0.34	0.87	0.77	0.03	0.60	0.32	0.86	0.75	0.03	0.59	0.35	0.84	0.80
DNF00809	0.11	0.49	0.86	0.65	0.92	0.07	0.65	0.92	0.73	0.97	0.10	0.51	0.77	0.87	0.99	0.10	0.55	0.80	0.86	0.98	0.10	0.53	0.76	0.84	0.98
Dorea	0.16	0.23	0.11	0.45	0.75	0.11	0.37	0.33	0.60	0.80	0.14	0.26	0.22	0.78	0.73	0.14	0.27	0.24	0.80	0.74	0.14	0.28	0.22	0.81	0.72
DTU089	0.04	0.74	0.93	0.83	0.95	0.03	0.78	0.80	0.84	0.97	0.00	0.98	0.98	0.99	0.99	-0.01	0.93	0.95	0.96	0.98	-0.01	0.95	0.86	0.96	0.98
Eggerthella	-0.09	0.48	0.62	0.65	0.91	-0.14	0.21	0.35	0.58	0.80	-0.08	0.51	0.51	0.87	0.91	-0.08	0.49	0.51	0.86	0.93	-0.08	0.48	0.42	0.84	0.87
Eisenbergiella	-0.29	0.02	0.01	0.18	0.53	-0.30	0.01	0.00	0.18	0.11	-0.31	0.01	0.01	0.29	0.18	-0.31	0.01	0.01	0.25	0.20	-0.31	0.01	0.00	0.24	0.13
Erysipelatoclostridium	-0.23	0.06	0.06	0.30	0.74	-0.21	0.06	0.06	0.35	0.51	-0.17	0.16	0.11	0.66	0.69	-0.17	0.15	0.12	0.58	0.72	-0.17	0.15	0.11	0.63	0.63
Erysipelotrichaceae_UCG.003	0.10	0.27	0.66	0.48	0.91	0.08	0.34	0.71	0.60	0.95	0.05	0.60	0.70	0.87	0.99	0.05	0.58	0.65	0.86	0.98	0.05	0.57	0.62	0.84	0.97
Escherichia.Shigella	-0.14	0.10	0.24	0.32	0.91	-0.08	0.35	0.34	0.60	0.80	-0.10	0.25	0.13	0.78	0.72	-0.10	0.26	0.13	0.80	0.72	-0.10	0.23	0.11	0.72	0.64
Faecalibacterium	0.23	0.12	0.82	0.35	0.91	0.23	0.09	0.45	0.40	0.94	0.30	0.05	0.14	0.41	0.72	0.30	0.05	0.15	0.40	0.72	0.30	0.04	0.16	0.40	0.72
Family_XIII_AD3011_group	0.27	0.03	0.50	0.20	0.91	0.13	0.26	0.94	0.59	0.97	0.08	0.54	0.93	0.87	0.99	0.07	0.58	0.94	0.86	0.98	0.06	0.61	0.97	0.86	0.98
Family_XIII_UCG.001	0.14	0.30	0.72	0.51	0.91	0.08	0.55	0.64	0.72	0.95	0.05	0.71	0.76	0.90	0.99	0.04	0.76	0.75	0.91	0.98	0.04	0.76	0.76	0.90	0.98
FD2005	-0.15	0.24	0.33	0.46	0.91	-0.11	0.35	0.27	0.60	0.80	-0.12	0.31	0.22	0.78	0.73	-0.12	0.34	0.24	0.82	0.74	-0.12	0.33	0.22	0.81	0.72
Flavonifractor	-0.16	0.15	0.62	0.36	0.91	-0.16	0.13	0.56	0.49	0.95	-0.11	0.30	0.60	0.78	0.99	-0.11	0.31	0.59	0.80	0.97	-0.11	0.32	0.56	0.81	0.95
Fournierella	0.06	0.62	0.80	0.77	0.91	0.05	0.64	0.69	0.73	0.95	0.03	0.79	0.65	0.92	0.99	0.03	0.82	0.63	0.94	0.97	0.03	0.83	0.74	0.96	0.98
Frisingicoccus	-0.13	0.23	0.06	0.45	0.74	-0.10	0.33	0.22	0.60	0.71	-0.13	0.22	0.27	0.74	0.75	-0.13	0.23	0.30	0.74	0.75	-0.13	0.23	0.27	0.72	0.72
Fusicatenibacter	0.11	0.34	0.59	0.54	0.91	0.08	0.41	0.42	0.62	0.93	0.05	0.66	0.42	0.89	0.89	0.05	0.62	0.42	0.87	0.88	0.05	0.64	0.40	0.88	0.86
Fusobacterium	-0.18	0.13	0.25	0.35	0.91	-0.12	0.26	0.18	0.59	0.67	-0.05	0.63	0.14	0.87	0.72	-0.06	0.60	0.14	0.86	0.72	-0.06	0.57	0.12	0.84	0.65
GCA.900066575	0.13	0.33	0.61	0.52	0.91	0.04	0.72	0.62	0.78	0.95	0.02	0.90	0.88	0.96	0.99	0.01	0.92	0.93	0.96	0.98	0.01	0.95	0.94	0.96	0.98
Gordonibacter	-0.06	0.67	0.16	0.78	0.80	-0.12	0.35	0.09	0.60	0.52	-0.08	0.57	0.30	0.87	0.75	-0.09	0.54	0.31	0.86	0.75	-0.09	0.54	0.32	0.84	0.78
Granulicatella	-0.15	0.43	0.46	0.62	0.91	-0.09	0.62	0.65	0.73	0.95	-0.06	0.74	0.69	0.90	0.99	-0.07	0.70	0.62	0.89	0.97	-0.07	0.72	0.58	0.89	0.96
Haemophilus	0.23	0.01	0.19	0.15	0.82	0.22	0.01	0.07	0.18	0.51	0.20	0.02	0.04	0.40	0.50	0.20	0.03	0.04	0.40	0.53	0.21	0.02	0.05	0.38	0.53
Harryflintia	-0.16	0.28	0.19	0.49	0.82	-0.24	0.09	0.06	0.40	0.51	-0.30	0.04	0.03	0.41	0.50	-0.30	0.04	0.03	0.40	0.53	-0.31	0.04	0.03	0.40	0.44
Holdemanella	0.06	0.49	0.15	0.65	0.80	0.07	0.38	0.19	0.61	0.67	0.09	0.29	0.28	0.78	0.75	0.09	0.30	0.31	0.80	0.75	0.09	0.31	0.33	0.81	0.78
Holdemania	-0.26	0.29	0.07	0.50	0.74	-0.21	0.36	0.09	0.60	0.52	-0.18	0.45	0.18	0.87	0.73	-0.19	0.43	0.17	0.86	0.74	-0.19	0.44	0.23	0.84	0.72
Hungatella	-0.45	0.00	0.01	0.02	0.53	-0.45	0.00	0.00	0.01	0.11	-0.43	0.00	0.00	0.03	0.08	-0.44	0.00	0.00	0.03	0.08	-0.44	0.00	0.00	0.03	0.05
Hydrogenoanaerobacterium	0.33	0.03	0.69	0.22	0.91	0.20	0.17	0.86	0.53	0.97	0.12	0.45	0.91	0.87	0.99	0.11	0.45	0.95	0.86	0.98	0.11	0.47	0.92	0.84	0.98
Incertae_Sedis	0.07	0.73	0.78	0.83	0.91	0.13	0.52	0.92	0.72	0.97	0.12	0.57	0.83	0.87	0.99	0.12	0.57	0.84	0.86	0.98	0.12	0.56	0.83	0.84	0.98
Intestinibacter	0.10	0.36	1.00	0.55	1.00	0.15	0.11	0.95	0.46	0.97	0.14	0.17	0.97	0.66	0.99	0.15	0.14	0.93	0.58	0.98	0.15	0.14	0.95	0.62	0.98
Intestinimonas	0.09	0.42	0.23	0.62	0.91	-0.05	0.63	0.04	0.73	0.51	-0.09	0.43	0.04	0.87	0.50	-0.09	0.43	0.05	0.86	0.53	-0.10	0.40	0.03	0.84	0.44
Lachnoclostridium	-0.66	0.00	0.44	0.11	0.91	-0.59	0.01	0.19	0.18	0.67	-0.42	0.06	0.23	0.45	0.73	-0.41	0.07	0.23	0.49	0.74	-0.41	0.07	0.23	0.50	0.72
Lachnospira	0.38	0.00	0.59	0.08	0.91	0.25	0.03	0.65	0.25	0.95	0.16	0.18	0.75	0.66	0.99	0.17	0.15	0.70	0.58	0.98	0.17	0.17	0.85	0.64	0.98
Lachnospiraceae_FCS020_group	0.31	0.01	0.64	0.12	0.91	0.23	0.03	0.89	0.27	0.97	0.23	0.04	0.82	0.41	0.99	0.23	0.04	0.83	0.40	0.98	0.23	0.04	0.80	0.40	0.98
Lachnospiraceae_ND3007_group	0.23	0.00	0.05	0.11	0.74	0.17	0.02	0.03	0.25	0.51	0.17	0.03	0.01	0.40	0.28	0.18	0.03	0.01	0.40	0.30	0.18	0.03	0.02	0.38	0.33
Lachnospiraceae_NK4A136_group	0.20	0.09	0.32	0.31	0.91	0.09	0.39	0.31	0.61	0.80	-0.01	0.93	0.23	0.96	0.73	-0.01	0.93	0.23	0.96	0.74	-0.01	0.90	0.27	0.96	0.72
Lachnospiraceae_UCG.001	0.17	0.04	0.45	0.25	0.91	0.13	0.08	0.43	0.40	0.94	0.09	0.30	0.46	0.78	0.90	0.09	0.30	0.43	0.80	0.88	0.08	0.32	0.49	0.81	0.92
Lachnospiraceae_UCG.004	0.18	0.09	0.31	0.31	0.91	0.10	0.32	0.60	0.60	0.95	0.09	0.38	0.48	0.84	0.90	0.09	0.36	0.48	0.82	0.93	0.09	0.36	0.39	0.81	0.86
Lachnospiraceae_UCG.010	-0.06	0.68	0.92	0.78	0.95	-0.15	0.26	0.84	0.59	0.97	-0.17	0.19	0.90	0.69	0.99	-0.18	0.18	0.88	0.65	0.98	-0.19	0.16	0.85	0.63	0.98
Lactobacillus	-0.27	0.03	0.57	0.20	0.91	-0.17	0.14	0.58	0.50	0.95	-0.23	0.06	0.28	0.45	0.75	-0.23	0.06	0.24	0.46	0.74	-0.23	0.06	0.26	0.50	0.72
Lactococcus	0.19	0.22	0.55	0.45	0.91	0.09	0.52	0.92	0.72	0.97	0.11	0.47	0.88	0.87	0.99	0.10	0.49	0.88	0.86	0.98	0.10	0.48	0.77	0.84	0.98
Marvinbryantia	0.15	0.10	0.10	0.33	0.75	0.09	0.32	0.14	0.60	0.67	0.09	0.30	0.14	0.78	0.72	0.09	0.32	0.14	0.80	0.72	0.09	0.31	0.17	0.81	0.72
Megasphaera	-0.13	0.07	0.54	0.30	0.91	-0.06	0.35	0.49	0.60	0.95	-0.02	0.73	0.30	0.90	0.75	-0.03	0.70	0.26	0.89	0.75	-0.03	0.70	0.32	0.89	0.78

© 2022 Meyer K et al. *JAMA Network Open.*

Monoglobus	0.25	0.03	0.71	0.20	0.91	0.19	0.06	0.63	0.35	0.95	0.12	0.28	0.48	0.78	0.90	0.12	0.28	0.50	0.80	0.93	0.12	0.28	0.45	0.81	0.88
Moryella	0.31	0.05	0.49	0.25	0.91	0.24	0.09	0.35	0.40	0.80	0.14	0.34	0.46	0.81	0.90	0.14	0.35	0.48	0.82	0.93	0.14	0.36	0.51	0.81	0.95
Negativibacillus	-0.19	0.07	0.12	0.30	0.75	-0.24	0.02	0.02	0.21	0.41	-0.26	0.01	0.01	0.29	0.24	-0.26	0.01	0.01	0.25	0.26	-0.26	0.01	0.01	0.24	0.28
NK4A214_group	0.08	0.32	0.77	0.51	0.91	0.01	0.85	0.48	0.89	0.95	-0.04	0.59	0.37	0.87	0.80	-0.05	0.57	0.38	0.86	0.81	-0.05	0.54	0.32	0.84	0.78
Odoribacter	0.10	0.21	0.86	0.45	0.92	0.09	0.22	0.90	0.58	0.97	0.05	0.56	0.99	0.87	0.99	0.05	0.54	0.96	0.86	0.98	0.04	0.58	0.97	0.84	0.98
Oscillibacter	0.16	0.23	0.72	0.45	0.91	0.12	0.34	0.73	0.60	0.95	0.06	0.66	0.49	0.89	0.90	0.05	0.69	0.50	0.89	0.93	0.06	0.67	0.53	0.89	0.95
Oscillospira	0.11	0.54	0.80	0.69	0.91	0.07	0.67	0.92	0.73	0.97	0.06	0.74	0.72	0.90	0.99	0.05	0.76	0.72	0.91	0.98	0.06	0.74	0.64	0.89	0.97
Oxalobacter	0.33	0.01	0.31	0.14	0.91	0.22	0.07	0.23	0.36	0.71	0.18	0.15	0.18	0.65	0.73	0.18	0.15	0.18	0.58	0.74	0.17	0.18	0.22	0.66	0.72
Paludicola	0.28	0.04	0.46	0.22	0.91	0.11	0.39	0.97	0.61	0.97	0.03	0.82	0.97	0.94	0.99	0.02	0.88	0.99	0.96	0.99	0.02	0.87	0.89	0.96	0.98
Parabacteroides	0.04	0.65	0.82	0.78	0.91	0.10	0.25	0.69	0.59	0.95	0.10	0.26	0.76	0.78	0.99	0.10	0.30	0.85	0.80	0.98	0.10	0.29	0.88	0.81	0.98
Paraprevotella	0.00	0.96	0.35	0.98	0.91	0.03	0.63	0.29	0.73	0.80	0.03	0.59	0.18	0.87	0.73	0.03	0.64	0.20	0.87	0.74	0.04	0.59	0.18	0.84	0.72
Parasutterella	0.17	0.02	0.83	0.19	0.91	0.16	0.01	0.58	0.21	0.95	0.09	0.20	0.97	0.71	0.99	0.09	0.19	0.95	0.66	0.98	0.09	0.21	0.89	0.70	0.98
Peptococcus	-0.03	0.80	0.08	0.85	0.74	-0.13	0.28	0.04	0.60	0.51	-0.19	0.13	0.05	0.60	0.50	-0.19	0.12	0.05	0.54	0.53	-0.20	0.11	0.04	0.53	0.50
Phascolarctobacterium	0.03	0.64	0.35	0.78	0.91	0.03	0.66	0.20	0.73	0.69	0.03	0.61	0.28	0.87	0.75	0.03	0.64	0.28	0.87	0.75	0.03	0.65	0.28	0.88	0.72
Phocea	-0.24	0.14	0.44	0.36	0.91	-0.34	0.02	0.17	0.25	0.67	-0.42	0.01	0.06	0.27	0.50	-0.42	0.01	0.06	0.25	0.53	-0.42	0.01	0.05	0.24	0.53
Prevotella	-0.07	0.23	0.60	0.45	0.91	-0.05	0.35	0.95	0.60	0.97	-0.04	0.43	0.86	0.87	0.99	-0.04	0.41	0.90	0.86	0.98	-0.04	0.42	0.93	0.84	0.98
Romboutsia	0.09	0.40	0.41	0.60	0.91	0.14	0.16	0.26	0.50	0.80	0.12	0.24	0.30	0.78	0.75	0.12	0.23	0.29	0.74	0.75	0.13	0.21	0.27	0.70	0.72
Roseburia	0.35	0.01	0.03	0.14	0.60	0.25	0.05	0.10	0.30	0.52	0.13	0.35	0.33	0.81	0.77	0.13	0.34	0.32	0.82	0.75	0.13	0.35	0.40	0.81	0.86
Ruminococcus	0.10	0.21	0.55	0.45	0.91	0.06	0.41	0.76	0.62	0.97	0.02	0.83	0.68	0.94	0.99	0.02	0.83	0.70	0.94	0.98	0.02	0.82	0.67	0.96	0.98
Sellimonas	0.16	0.15	0.96	0.37	0.97	0.19	0.07	0.45	0.38	0.94	0.19	0.08	0.35	0.50	0.78	0.19	0.08	0.34	0.51	0.77	0.19	0.08	0.35	0.50	0.80
Senegalimassilia	0.05	0.66	0.62	0.78	0.91	0.04	0.67	0.69	0.73	0.95	0.02	0.86	0.85	0.95	0.99	0.01	0.91	0.90	0.96	0.98	0.01	0.91	0.94	0.96	0.98
Shuttleworthia	0.16	0.24	0.74	0.46	0.91	0.08	0.51	0.90	0.72	0.97	0.04	0.76	0.86	0.90	0.99	0.05	0.73	0.79	0.89	0.98	0.05	0.71	0.83	0.89	0.98
Slackia	-0.08	0.39	0.85	0.58	0.92	-0.04	0.64	0.95	0.73	0.97	-0.06	0.49	0.87	0.87	0.99	-0.07	0.45	0.79	0.86	0.98	-0.07	0.43	0.87	0.84	0.98
Streptococcus	0.11	0.45	0.83	0.64	0.91	0.10	0.45	0.83	0.66	0.97	0.14	0.33	0.85	0.80	0.99	0.12	0.38	0.94	0.84	0.98	0.14	0.35	0.91	0.81	0.98
Subdoligranulum	0.19	0.08	0.60	0.31	0.91	0.14	0.15	0.62	0.50	0.95	0.11	0.28	0.74	0.78	0.99	0.11	0.30	0.72	0.80	0.98	0.11	0.30	0.67	0.81	0.98
Sutterella	-0.09	0.16	0.48	0.39	0.91	-0.05	0.39	0.57	0.61	0.95	0.00	1.00	0.17	1.00	0.73	0.00	0.96	0.19	0.97	0.74	0.00	0.99	0.16	0.99	0.72
TM7x	-0.05	0.76	0.84	0.83	0.92	-0.11	0.44	0.90	0.65	0.97	-0.02	0.87	0.74	0.95	0.99	-0.03	0.84	0.77	0.94	0.98	-0.03	0.85	0.85	0.96	0.98
Turicibacter	0.28	0.04	0.25	0.22	0.91	0.39	0.00	0.08	0.09	0.52	0.41	0.00	0.15	0.10	0.72	0.41	0.00	0.13	0.08	0.72	0.42	0.00	0.09	0.07	0.56
Tuzzerella	-0.12	0.23	0.50	0.45	0.91	-0.12	0.21	0.30	0.58	0.80	-0.14	0.18	0.33	0.66	0.77	-0.14	0.18	0.36	0.66	0.79	-0.13	0.19	0.42	0.68	0.87
UBA1819	-0.24	0.12	0.02	0.35	0.54	-0.30	0.04	0.00	0.29	0.11	-0.30	0.04	0.00	0.41	0.11	-0.31	0.04	0.00	0.40	0.11	-0.31	0.04	0.00	0.40	0.08
UCG.002	0.03	0.67	0.26	0.78	0.91	-0.03	0.66	0.14	0.73	0.67	-0.06	0.49	0.31	0.87	0.75	-0.06	0.46	0.31	0.86	0.75	-0.06	0.43	0.27	0.84	0.72
UCG.003	0.16	0.05	0.46	0.26	0.91	0.09	0.25	0.54	0.59	0.95	0.06	0.49	0.47	0.87	0.90	0.05	0.52	0.50	0.86	0.93	0.05	0.54	0.45	0.84	0.88
UCG.005	0.15	0.10	0.81	0.32	0.91	0.11	0.22	0.90	0.58	0.97	0.06	0.52	0.97	0.87	0.99	0.06	0.56	0.97	0.86	0.98	0.06	0.55	0.99	0.84	0.99
UCG.009	0.16	0.26	0.77	0.47	0.91	0.08	0.53	0.50	0.72	0.95	-0.01	0.92	0.73	0.96	0.99	-0.02	0.90	0.74	0.96	0.98	-0.02	0.89	0.78	0.96	0.98
Veillonella	0.16	0.09	0.16	0.31	0.80	0.18	0.04	0.07	0.30	0.51	0.16	0.08	0.07	0.50	0.50	0.16	0.08	0.08	0.51	0.53	0.17	0.08	0.08	0.50	0.56
Victivallis	0.13	0.17	0.81	0.40	0.91	0.08	0.34	0.94	0.60	0.97	0.04	0.69	0.87	0.90	0.99	0.04	0.69	0.82	0.89	0.98	0.04	0.69	0.89	0.89	0.98

Multivariable-adjusted regression models adjusted for:

Model 1: sequencing batch, age, race, gender, study center

Model 2: Model 1, with education

Model 3: Model 2, with physical activity, diet score, medication use, and smoking

Model 4: Model 3 with BMI

Model 5: Model 4 with hypertension and diabetes

© 2022 Meyer K et al. *JAMA Network Open.*

eTable 47: Multivariable-adjusted regression coefficients, p-values, and false discovery rate (FDR)-adjusted p-values for genera among male participants with respect to DSST.

	Model1			Model2			Model3			Model4			Model5		
	beta	p-value	FDR	beta	p-value	FDR	beta	p-value	FDR	beta	p-value	FDR	beta	p-value	FDR
Acetanaerobacterium	0.80	0.70	0.82	0.54	0.77	0.90	0.56	0.77	0.94	0.51	0.79	0.96	0.66	0.73	0.94
Acidaminococcus	0.17	0.77	0.86	0.67	0.23	0.51	0.90	0.12	0.85	0.78	0.18	0.77	0.68	0.25	0.75
Actinomyces	-1.17	0.49	0.66	-1.03	0.51	0.72	-0.51	0.76	0.94	0.02	0.99	1.00	-0.43	0.80	0.97
Adlercreutzia	0.29	0.77	0.86	0.46	0.61	0.81	0.70	0.45	0.89	0.61	0.50	0.84	0.73	0.43	0.81
Agathobacter	0.93	0.50	0.67	0.15	0.90	0.94	-0.57	0.66	0.91	-1.03	0.43	0.81	-1.09	0.40	0.81
Akkermansia	1.68	0.01	0.10	1.04	0.11	0.47	1.03	0.12	0.85	0.97	0.15	0.77	1.03	0.12	0.75
Alistipes	2.51	0.04	0.16	1.54	0.17	0.51	1.19	0.30	0.85	1.12	0.32	0.77	1.50	0.19	0.75
Allisonella	-0.01	0.99	0.99	1.15	0.27	0.54	0.64	0.56	0.91	0.59	0.59	0.90	0.53	0.63	0.91
Anaerofilum	2.34	0.11	0.33	1.47	0.29	0.54	1.62	0.25	0.85	1.56	0.26	0.77	1.63	0.24	0.75
Anaerostipes	2.49	0.13	0.36	1.48	0.34	0.57	1.67	0.29	0.85	1.48	0.34	0.77	1.26	0.42	0.81
Anaerotruncus	1.58	0.24	0.47	0.84	0.50	0.72	1.11	0.39	0.88	1.44	0.26	0.77	1.55	0.22	0.75
Bacteroides	-1.26	0.55	0.70	-1.37	0.48	0.72	0.16	0.94	0.98	-0.24	0.90	0.99	0.05	0.98	0.99
Barnesiella	1.56	0.01	0.10	1.65	0.00	0.31	1.19	0.05	0.71	1.21	0.04	0.61	1.19	0.04	0.51
Bifidobacterium	-0.36	0.65	0.80	0.18	0.81	0.92	0.12	0.87	0.97	0.00	1.00	1.00	-0.08	0.91	0.99
Bilophila	0.47	0.60	0.76	-0.29	0.73	0.88	-0.55	0.53	0.91	-0.83	0.34	0.77	-0.71	0.42	0.81
Blautia	7.43	0.01	0.10	5.27	0.05	0.33	5.97	0.03	0.71	6.05	0.03	0.61	6.13	0.02	0.51
Butyricicoccus	4.88	0.03	0.16	2.51	0.24	0.51	2.53	0.24	0.85	2.57	0.23	0.77	2.40	0.27	0.75
Butyricimonas	1.10	0.15	0.37	0.75	0.29	0.54	0.85	0.23	0.85	0.84	0.23	0.77	0.99	0.16	0.75
CAG.56	0.49	0.54	0.70	0.01	0.99	0.99	-0.24	0.75	0.94	-0.21	0.78	0.96	-0.06	0.94	0.99
Candidatus_Soleaferrea	1.27	0.42	0.66	0.20	0.89	0.94	-0.10	0.95	0.98	-0.04	0.98	1.00	-0.01	0.99	0.99
Caproiciproducens	0.03	0.98	0.99	-0.35	0.80	0.92	0.10	0.94	0.98	0.03	0.98	1.00	-0.15	0.92	0.99
Catenibacterium	1.09	0.16	0.37	1.00	0.16	0.51	0.59	0.42	0.88	0.64	0.38	0.78	0.46	0.53	0.89
Christensenellaceae_R.7_group	1.84	0.01	0.10	1.31	0.05	0.33	0.86	0.21	0.85	0.94	0.17	0.77	0.97	0.15	0.75
Clostridium_sensu_stricto_1	0.60	0.47	0.66	1.00	0.20	0.51	0.56	0.49	0.91	0.60	0.46	0.84	0.32	0.70	0.94
Colidextribacter	-1.64	0.33	0.55	-2.92	0.06	0.35	-1.81	0.25	0.85	-1.87	0.24	0.77	-2.18	0.17	0.75
Collinsella	-0.58	0.48	0.66	-0.24	0.75	0.88	-0.70	0.38	0.88	-0.63	0.42	0.81	-0.63	0.42	0.81
Coprobacter	0.61	0.46	0.66	0.28	0.71	0.88	-0.31	0.69	0.92	-0.20	0.79	0.96	-0.01	0.99	0.99
Coprococcus	2.68	0.01	0.10	1.69	0.07	0.38	1.34	0.16	0.85	1.55	0.10	0.70	1.58	0.10	0.75
Defluviitaleaceae_UCG.011	2.45	0.08	0.27	2.45	0.05	0.33	2.75	0.04	0.71	2.81	0.03	0.61	2.88	0.03	0.51
Desulfovibrio	0.89	0.23	0.46	0.88	0.20	0.51	0.93	0.20	0.85	1.29	0.07	0.67	1.44	0.05	0.51
Dialister	-0.04	0.95	0.97	-0.01	0.99	0.99	0.29	0.62	0.91	0.19	0.74	0.96	-0.03	0.96	0.99
Dorea	1.82	0.15	0.37	1.13	0.34	0.57	1.08	0.36	0.88	1.23	0.30	0.77	1.34	0.25	0.75
DTU089	1.43	0.25	0.48	1.36	0.24	0.51	0.67	0.57	0.91	1.06	0.36	0.78	1.13	0.33	0.79
Eggerthella	-0.86	0.47	0.66	-1.27	0.25	0.51	-0.37	0.75	0.94	-0.25	0.83	0.97	-0.22	0.84	0.99
Eisenbergiella	-1.94	0.15	0.37	-1.80	0.15	0.51	-0.67	0.60	0.91	-0.39	0.76	0.96	-0.46	0.72	0.94
Enterorhabdus	1.96	0.08	0.27	1.38	0.19	0.51	1.03	0.35	0.88	1.11	0.31	0.77	1.03	0.35	0.81
Erysipelatoclostridium	-0.92	0.48	0.66	-0.51	0.67	0.86	0.58	0.64	0.91	0.85	0.49	0.84	0.72	0.55	0.90
Erysipelotrichaceae_UCG.003	0.38	0.68	0.81	0.14	0.87	0.94	-0.20	0.82	0.96	-0.23	0.79	0.96	-0.25	0.77	0.94
Escherichia.Shigella	-0.89	0.32	0.55	-0.09	0.91	0.94	0.28	0.75	0.94	0.15	0.86	0.98	0.28	0.75	0.94
Faecalibacterium	2.59	0.07	0.26	2.77	0.03	0.33	2.79	0.04	0.71	2.87	0.04	0.61	2.72	0.05	0.51
Family_XIII_AD3011_group	3.44	0.00	0.07	2.01	0.07	0.37	1.75	0.12	0.85	2.19	0.05	0.67	2.30	0.04	0.51
Family_XIII_UCG.001	2.46	0.07	0.26	1.79	0.15	0.51	1.32	0.31	0.85	1.79	0.17	0.77	1.89	0.15	0.75
FD2005	0.95	0.43	0.66	1.19	0.28	0.54	0.92	0.42	0.88	0.58	0.61	0.92	0.54	0.64	0.91
Flavonifractor	-0.91	0.41	0.65	-0.69	0.50	0.72	0.64	0.55	0.91	0.57	0.59	0.90	0.42	0.69	0.94
Fournierella	1.40	0.22	0.46	1.24	0.24	0.51	0.12	0.91	0.98	0.32	0.77	0.96	0.36	0.74	0.94
Frisingicoccus	-1.90	0.07	0.26	-1.50	0.12	0.48	-1.15	0.24	0.85	-1.27	0.19	0.77	-1.08	0.27	0.75
Fusicatenibacter	0.28	0.79	0.86	-0.01	0.99	0.99	-0.56	0.58	0.91	-0.69	0.48	0.84	-0.68	0.49	0.84
Fusobacterium	-2.32	0.08	0.27	-1.48	0.24	0.51	-0.96	0.45	0.89	-0.69	0.58	0.90	-0.37	0.77	0.94
GCA.900066575	1.80	0.15	0.37	1.09	0.34	0.57	0.34	0.77	0.94	0.49	0.67	0.95	0.43	0.71	0.94
Gordonibacter	-1.25	0.36	0.60	-1.74	0.17	0.51	-0.72	0.57	0.91	-0.46	0.72	0.96	-0.29	0.82	0.98
Granulicatella	-2.76	0.12	0.35	-1.87	0.26	0.53	-0.96	0.58	0.91	-0.83	0.63	0.92	-1.00	0.57	0.90
Haemophilus	1.44	0.10	0.30	1.41	0.08	0.39	1.29	0.13	0.85	1.36	0.11	0.70	1.21	0.16	0.75
Harryflintia	1.22	0.40	0.65	0.46	0.74	0.88	0.33	0.82	0.96	0.41	0.77	0.96	0.76	0.59	0.90
Holdemanella	1.03	0.23	0.46	0.98	0.21	0.51	0.71	0.39	0.88	0.71	0.38	0.78	0.84	0.30	0.76

© 2022 Meyer K et al. *JAMA Network Open.*

Holdemania	-0.21	0.93	0.95	0.26	0.90	0.94	1.51	0.50	0.91	2.14	0.34	0.77	2.50	0.26	0.75
Howardella	0.11	0.92	0.95	-0.16	0.87	0.94	-0.66	0.51	0.91	-0.44	0.66	0.94	-0.51	0.60	0.91
Hungatella	-2.74	0.03	0.16	-2.44	0.03	0.33	-1.47	0.21	0.85	-1.25	0.29	0.77	-1.48	0.21	0.75
Hydrogenoanaerobacterium	4.14	0.00	0.09	2.86	0.04	0.33	2.36	0.10	0.85	2.55	0.07	0.67	2.44	0.08	0.70
Incertae_Sedis	4.31	0.04	0.16	4.57	0.02	0.33	4.78	0.02	0.71	4.73	0.02	0.61	4.94	0.01	0.51
Intestinibacter	0.28	0.80	0.86	0.82	0.42	0.66	0.46	0.66	0.91	0.15	0.89	0.99	-0.13	0.90	0.99
Intestinimonas	2.74	0.01	0.10	1.36	0.19	0.51	0.90	0.40	0.88	0.88	0.41	0.81	1.12	0.29	0.76
Lachnoclostridium	-4.86	0.03	0.16	-4.20	0.05	0.33	-1.76	0.42	0.88	-2.14	0.32	0.77	-2.11	0.33	0.79
Lachnospira	3.66	0.00	0.07	2.44	0.03	0.33	1.52	0.18	0.85	1.33	0.24	0.77	1.30	0.25	0.75
Lachnospiraceae_FCS020_group	3.49	0.00	0.07	2.67	0.01	0.33	2.26	0.04	0.71	2.33	0.03	0.61	2.30	0.03	0.51
Lachnospiraceae_ND3007_grou	1.03	0.19	0.41	0.40	0.58	0.80	0.05	0.94	0.98	0.03	0.96	1.00	0.04	0.95	0.99
Lachnospiraceae_NK4A136_gro	2.61	0.02	0.11	1.60	0.12	0.48	0.66	0.53	0.91	0.76	0.47	0.84	0.84	0.42	0.81
Lachnospiraceae_UCG.001	2.04	0.01	0.10	1.65	0.02	0.33	1.56	0.05	0.71	1.58	0.04	0.61	1.53	0.05	0.51
Lachnospiraceae_UCG.004	2.65	0.01	0.10	1.88	0.04	0.33	1.60	0.09	0.85	1.57	0.09	0.67	1.41	0.13	0.75
Lachnospiraceae_UCG.010	0.24	0.86	0.91	-0.58	0.63	0.83	-0.54	0.66	0.91	-0.25	0.84	0.97	0.12	0.92	0.99
Lactobacillus	-1.18	0.32	0.55	-0.24	0.83	0.92	-0.62	0.59	0.91	-0.60	0.60	0.90	-1.01	0.38	0.81
Lactococcus	-0.17	0.91	0.95	-0.93	0.48	0.72	-0.36	0.79	0.94	-0.10	0.94	1.00	0.01	0.99	0.99
Marvinbryantia	1.01	0.25	0.48	0.37	0.65	0.85	0.02	0.98	0.98	0.12	0.88	0.99	0.14	0.87	0.99
Megasphaera	-0.22	0.75	0.86	0.38	0.55	0.76	0.44	0.52	0.91	0.43	0.53	0.87	0.32	0.64	0.91
Monoglobus	1.20	0.27	0.51	0.71	0.48	0.72	0.12	0.91	0.98	0.23	0.83	0.97	0.59	0.59	0.90
Moryella	3.34	0.02	0.12	2.60	0.05	0.33	1.43	0.31	0.85	1.72	0.22	0.77	1.60	0.25	0.75
Negativibacillus	-0.15	0.88	0.93	-0.68	0.46	0.72	-0.98	0.30	0.85	-1.03	0.28	0.77	-0.92	0.33	0.79
NK4A214_group	1.29	0.09	0.29	0.59	0.41	0.66	-0.02	0.98	0.98	0.08	0.92	1.00	0.05	0.95	0.99
Odoribacter	0.55	0.46	0.66	0.46	0.51	0.72	0.09	0.90	0.98	0.04	0.95	1.00	0.23	0.75	0.94
Oscillibacter	1.69	0.19	0.41	1.24	0.30	0.54	1.38	0.27	0.85	1.61	0.20	0.77	1.51	0.22	0.75
Oscillospira	0.77	0.67	0.81	0.53	0.75	0.88	1.87	0.27	0.85	2.12	0.20	0.77	2.27	0.17	0.75
Oxalobacter	3.70	0.00	0.07	2.57	0.03	0.33	2.28	0.05	0.74	2.36	0.05	0.61	2.90	0.02	0.51
Paludicola	3.10	0.01	0.10	1.42	0.24	0.51	1.00	0.43	0.88	1.59	0.21	0.77	1.60	0.21	0.75
Parabacteroides	0.44	0.63	0.79	1.00	0.23	0.51	1.16	0.17	0.85	1.42	0.09	0.67	1.57	0.06	0.59
Paraprevotella	0.75	0.23	0.46	0.93	0.11	0.47	0.88	0.15	0.85	1.05	0.08	0.67	0.99	0.11	0.75
Parasutterella	0.86	0.21	0.45	0.86	0.18	0.51	0.40	0.55	0.91	0.32	0.63	0.92	0.51	0.45	0.82
Peptococcus	2.52	0.04	0.16	1.62	0.15	0.51	1.39	0.22	0.85	1.69	0.14	0.77	1.69	0.14	0.75
Phascolarctobacterium	0.19	0.76	0.86	0.20	0.73	0.88	0.18	0.77	0.94	0.34	0.58	0.90	0.53	0.38	0.81
Phocea	1.13	0.49	0.66	0.34	0.82	0.92	0.03	0.98	0.98	0.00	1.00	1.00	0.14	0.93	0.99
Prevotella	-0.25	0.64	0.80	-0.08	0.88	0.94	-0.34	0.50	0.91	-0.34	0.49	0.84	-0.37	0.46	0.82
Romboutsia	0.62	0.54	0.70	0.98	0.29	0.54	0.92	0.34	0.88	0.89	0.35	0.77	0.61	0.52	0.89
Roseburia	3.30	0.01	0.10	2.32	0.05	0.33	1.31	0.31	0.85	1.22	0.34	0.77	1.12	0.37	0.81
Ruminococcus	1.04	0.19	0.41	0.64	0.38	0.63	0.02	0.98	0.98	0.05	0.94	1.00	-0.04	0.96	0.99
Sellimonas	0.82	0.45	0.66	1.01	0.31	0.54	0.73	0.46	0.90	0.85	0.39	0.79	0.88	0.38	0.81
Senegalimassilia	1.39	0.16	0.37	1.27	0.16	0.51	0.35	0.71	0.93	0.53	0.57	0.90	0.53	0.57	0.90
Shuttleworthia	1.84	0.16	0.37	1.24	0.30	0.54	1.32	0.29	0.85	1.20	0.33	0.77	1.28	0.30	0.76
Slackia	0.30	0.73	0.85	0.54	0.50	0.72	0.17	0.84	0.96	0.34	0.68	0.95	0.40	0.63	0.91
Streptococcus	0.37	0.78	0.86	0.47	0.71	0.88	0.62	0.64	0.91	1.21	0.37	0.78	0.82	0.55	0.90
Subdoligranulum	1.03	0.33	0.55	0.52	0.59	0.80	0.21	0.83	0.96	0.36	0.72	0.96	0.36	0.72	0.94
Sutterella	-1.03	0.08	0.27	-0.71	0.19	0.51	-0.86	0.13	0.85	-0.77	0.17	0.77	-0.81	0.15	0.75
TM7x	-1.07	0.44	0.66	-1.63	0.21	0.51	-1.09	0.41	0.88	-0.91	0.49	0.84	-0.96	0.46	0.82
Turicibacter	-0.53	0.69	0.81	0.51	0.68	0.86	-0.04	0.98	0.98	-0.30	0.80	0.96	-0.55	0.65	0.93
Tuzzerella	-1.67	0.09	0.29	-1.57	0.08	0.40	-1.24	0.20	0.85	-1.31	0.17	0.77	-1.28	0.18	0.75
UBA1819	-1.19	0.43	0.66	-1.60	0.25	0.51	-0.61	0.67	0.91	-0.27	0.85	0.97	-0.19	0.89	0.99
UCG.002	0.80	0.30	0.55	0.08	0.91	0.94	-0.38	0.61	0.91	-0.25	0.74	0.96	-0.09	0.90	0.99
UCG.003	1.28	0.11	0.32	0.60	0.42	0.66	0.16	0.84	0.96	0.27	0.72	0.96	0.30	0.70	0.94
UCG.005	1.84	0.04	0.17	1.33	0.11	0.47	0.38	0.67	0.91	0.70	0.43	0.81	0.69	0.44	0.82
UCG.009	3.12	0.02	0.11	2.39	0.05	0.33	1.54	0.22	0.85	1.69	0.17	0.77	1.62	0.19	0.75
Veillonella	0.88	0.33	0.55	1.15	0.17	0.51	0.81	0.35	0.88	0.83	0.34	0.77	0.65	0.45	0.82
Victivallis	2.61	0.00	0.07	2.24	0.01	0.31	1.47	0.08	0.85	1.46	0.08	0.67	1.43	0.08	0.70

Multivariable-adjusted regression models adjusted for:
Model 1: sequencing batch, age, race, gender, study center
Model 2: Model 1, with education

© 2022 Meyer K et al. *JAMA Network Open.*

Model 3: Model 2, with physical activity, diet score, medication use, and smoking
Model 4: Model 3 with BMI
Model 5: Model 4 with hypertension and diabetes

© 2022 Meyer K et al. *JAMA Network Open.*

eTable 48: Multivariable-adjusted regression coefficients, p-values, and false discovery rate (FDR)-adjusted p-values for genera among male participants with respect to Stroop.

	Model1			Model2			Model3			Model4			Model5		
	beta	p-value	FDR	beta	p-value	FDR	beta	p-value	FDR	beta	p-value	FDR	beta	p-value	FDR
Acetanaerobacterium	0.15	0.93	0.97	0.24	0.89	0.98	1.15	0.48	0.85	1.15	0.48	0.84	1.03	0.53	0.89
Acidaminococcus	0.34	0.49	0.76	0.14	0.77	0.97	-0.47	0.35	0.85	-0.45	0.36	0.84	-0.39	0.44	0.89
Actinomyces	0.01	0.99	0.99	-0.06	0.96	0.98	-1.30	0.35	0.85	-1.40	0.32	0.82	-1.06	0.45	0.89
Adlercreutzia	1.93	0.02	0.31	1.85	0.02	0.43	1.86	0.02	0.35	1.87	0.02	0.34	1.81	0.02	0.43
Agathobacter	-1.51	0.19	0.61	-1.21	0.29	0.70	-0.26	0.81	0.96	-0.21	0.85	0.98	-0.12	0.91	0.99
Akkermansia	0.56	0.33	0.68	0.85	0.14	0.58	1.09	0.05	0.52	1.10	0.05	0.51	1.04	0.07	0.61
Alistipes	0.36	0.71	0.94	0.77	0.43	0.80	1.09	0.25	0.85	1.10	0.25	0.82	0.83	0.39	0.89
Allisonella	-0.22	0.81	0.97	-0.69	0.46	0.80	-0.25	0.79	0.96	-0.24	0.79	0.94	-0.24	0.80	0.99
Anaerofilum	2.32	0.06	0.53	2.72	0.03	0.44	3.04	0.01	0.30	3.05	0.01	0.32	2.96	0.01	0.37
Anaerostipes	-0.37	0.80	0.97	0.11	0.94	0.98	0.63	0.65	0.93	0.66	0.64	0.92	0.80	0.57	0.89
Anaerotruncus	1.33	0.24	0.68	1.64	0.14	0.58	2.40	0.02	0.35	2.38	0.03	0.37	2.27	0.04	0.56
Bacteroides	1.65	0.35	0.68	1.66	0.34	0.72	1.62	0.32	0.85	1.68	0.31	0.82	1.57	0.34	0.89
Barnesiella	-0.35	0.51	0.76	-0.38	0.46	0.80	-0.39	0.44	0.85	-0.39	0.43	0.84	-0.34	0.50	0.89
Bifidobacterium	-0.71	0.29	0.68	-0.94	0.16	0.58	-0.60	0.34	0.85	-0.59	0.35	0.84	-0.56	0.38	0.89
Bilophila	-0.14	0.86	0.97	0.18	0.81	0.98	0.50	0.50	0.85	0.55	0.46	0.84	0.50	0.50	0.89
Blautia	0.08	0.97	0.99	0.98	0.69	0.93	1.29	0.58	0.89	1.28	0.58	0.90	1.27	0.59	0.89
Butyricicoccus	-0.57	0.76	0.94	0.39	0.84	0.98	1.41	0.44	0.85	1.40	0.44	0.84	1.40	0.44	0.89
Butyricimonas	0.12	0.85	0.97	0.26	0.67	0.93	0.22	0.71	0.96	0.22	0.71	0.94	0.13	0.83	0.99
CAG.56	-0.35	0.60	0.86	-0.15	0.82	0.98	0.42	0.51	0.85	0.41	0.52	0.84	0.33	0.61	0.89
Candidatus_Soleaferrea	1.26	0.34	0.68	1.74	0.18	0.58	1.62	0.20	0.85	1.61	0.20	0.82	1.52	0.23	0.89
Caproiciproducens	-0.13	0.92	0.97	0.00	1.00	1.00	0.30	0.80	0.96	0.30	0.80	0.94	0.37	0.76	0.97
Catenibacterium	-1.13	0.08	0.58	-1.08	0.09	0.58	-0.48	0.44	0.85	-0.49	0.43	0.84	-0.39	0.53	0.89
Christensenellaceae_R.7_group	-1.28	0.03	0.33	-1.07	0.07	0.58	-0.64	0.27	0.85	-0.65	0.26	0.82	-0.72	0.22	0.89
Clostridium_sensu_stricto_1	0.02	0.98	0.99	-0.15	0.83	0.98	0.00	1.00	1.00	0.00	1.00	1.00	0.24	0.74	0.97
Colidextribacter	0.89	0.53	0.78	1.39	0.32	0.72	0.63	0.64	0.93	0.64	0.63	0.92	0.81	0.55	0.89
Collinsella	-0.81	0.24	0.68	-0.95	0.16	0.58	-0.68	0.31	0.85	-0.69	0.30	0.82	-0.74	0.27	0.89
Coprobacter	-0.22	0.75	0.94	-0.09	0.90	0.98	0.28	0.67	0.93	0.26	0.68	0.94	0.13	0.85	0.99
Coprococcus	-0.92	0.28	0.68	-0.51	0.55	0.86	-0.53	0.52	0.85	-0.56	0.50	0.84	-0.63	0.45	0.89
Defluviitaleaceae_UCG.011	-1.75	0.13	0.61	-1.77	0.12	0.58	-1.10	0.32	0.85	-1.10	0.32	0.82	-1.20	0.28	0.89
Desulfovibrio	-0.59	0.34	0.68	-0.59	0.33	0.72	-0.48	0.42	0.85	-0.54	0.38	0.84	-0.66	0.29	0.89
Dialister	-0.10	0.83	0.97	-0.12	0.80	0.98	-0.15	0.76	0.96	-0.14	0.78	0.94	0.00	0.99	0.99
Dorea	-1.55	0.14	0.61	-1.27	0.22	0.60	-1.28	0.19	0.85	-1.30	0.19	0.82	-1.38	0.16	0.84
DTU089	0.53	0.61	0.86	0.55	0.59	0.90	0.90	0.36	0.85	0.87	0.39	0.84	0.78	0.44	0.89
Eggerthella	1.40	0.16	0.61	1.57	0.11	0.58	1.13	0.24	0.85	1.12	0.25	0.82	1.09	0.26	0.89
Eisenbergiella	0.75	0.51	0.76	0.70	0.53	0.86	0.32	0.77	0.96	0.28	0.80	0.94	0.39	0.72	0.96
Enterorhabdus	-2.23	0.02	0.31	-1.99	0.03	0.44	-1.79	0.05	0.52	-1.80	0.05	0.51	-1.81	0.05	0.61
Erysipelatoclostridium	1.94	0.07	0.58	1.78	0.10	0.58	1.66	0.11	0.67	1.64	0.11	0.69	1.79	0.08	0.61
Erysipelotrichaceae_UCG.003	0.03	0.97	0.99	0.12	0.87	0.98	0.18	0.81	0.96	0.18	0.81	0.94	0.15	0.84	0.99
Escherichia.Shigella	0.36	0.63	0.86	0.04	0.96	0.98	0.03	0.97	1.00	0.05	0.95	1.00	-0.03	0.96	0.99
Faecalibacterium	-3.35	0.00	0.22	-3.42	0.00	0.28	-3.60	0.00	0.18	-3.61	0.00	0.18	-3.61	0.00	0.19
Family_XIII_AD3011_group	-0.77	0.42	0.71	-0.18	0.86	0.98	0.68	0.48	0.85	0.64	0.51	0.84	0.51	0.60	0.89
Family_XIII_UCG.001	-0.25	0.83	0.97	0.06	0.96	0.98	0.05	0.97	1.00	-0.01	0.99	1.00	-0.12	0.91	0.99
FD2005	-1.34	0.18	0.61	-1.42	0.15	0.58	-0.51	0.59	0.90	-0.48	0.63	0.92	-0.50	0.61	0.89
Flavonifractor	0.93	0.32	0.68	0.82	0.37	0.73	-0.10	0.91	1.00	-0.09	0.92	1.00	0.02	0.98	0.99
Fournierella	-1.00	0.29	0.68	-0.93	0.32	0.72	-0.23	0.80	0.96	-0.26	0.78	0.94	-0.34	0.71	0.96
Frisingicoccus	2.04	0.02	0.31	1.86	0.03	0.44	2.22	0.01	0.30	2.24	0.01	0.32	2.13	0.01	0.37
Fusicatenibacter	-0.83	0.37	0.69	-0.69	0.45	0.80	-0.25	0.77	0.96	-0.24	0.79	0.94	-0.22	0.80	0.99
Fusobacterium	0.99	0.37	0.69	0.66	0.55	0.86	0.75	0.48	0.85	0.72	0.50	0.84	0.56	0.60	0.89
GCA.900066575	-1.42	0.17	0.61	-1.15	0.26	0.65	-0.72	0.46	0.85	-0.74	0.45	0.84	-0.80	0.42	0.89
Gordonibacter	1.87	0.10	0.61	2.08	0.06	0.58	2.07	0.05	0.52	2.05	0.06	0.51	1.91	0.08	0.61
Granulicatella	0.14	0.92	0.97	-0.22	0.88	0.98	-1.66	0.26	0.85	-1.68	0.25	0.82	-1.51	0.30	0.89
Haemophilus	-0.97	0.18	0.61	-0.96	0.18	0.58	-0.93	0.20	0.85	-0.94	0.20	0.82	-0.90	0.22	0.89
Harryflintia	1.29	0.29	0.68	1.60	0.19	0.58	2.65	0.03	0.35	2.65	0.03	0.37	2.43	0.04	0.58
Holdemanella	-0.67	0.35	0.68	-0.63	0.37	0.73	-0.02	0.98	1.00	-0.02	0.98	1.00	-0.11	0.87	0.99

© 2022 Meyer K et al. *JAMA Network Open.*

Holdemania	2.93	0.14	0.61	2.75	0.16	0.58	2.37	0.21	0.85	2.33	0.22	0.82	2.05	0.28	0.89
Howardella	-1.19	0.17	0.61	-1.07	0.21	0.59	-0.07	0.94	1.00	-0.10	0.91	1.00	-0.06	0.95	0.99
Hungatella	2.99	0.00	0.22	2.87	0.01	0.28	2.52	0.01	0.30	2.51	0.01	0.32	2.71	0.01	0.37
Hydrogenoanaerobacterium	-1.58	0.20	0.62	-1.05	0.39	0.76	-0.70	0.56	0.87	-0.72	0.55	0.85	-0.72	0.55	0.89
Incertae_Sedis	0.21	0.91	0.97	0.10	0.96	0.98	0.01	1.00	1.00	0.01	0.99	1.00	-0.10	0.95	0.99
Intestinibacter	-0.94	0.31	0.68	-1.16	0.20	0.58	-0.95	0.29	0.85	-0.92	0.30	0.82	-0.84	0.36	0.89
Intestinimonas	0.79	0.39	0.69	1.45	0.12	0.58	1.53	0.09	0.67	1.54	0.09	0.68	1.35	0.14	0.83
Lachnoclostridium	3.58	0.06	0.53	3.29	0.08	0.58	2.01	0.27	0.85	2.07	0.26	0.82	2.19	0.23	0.89
Lachnospira	-2.44	0.01	0.31	-1.99	0.04	0.48	-1.54	0.11	0.67	-1.52	0.11	0.69	-1.56	0.11	0.70
Lachnospiraceae_FCS020_group	-1.56	0.10	0.61	-1.22	0.19	0.58	-0.36	0.70	0.95	-0.37	0.69	0.94	-0.37	0.69	0.95
Lachnospiraceae_ND3007_group	-0.85	0.19	0.61	-0.61	0.35	0.73	-0.22	0.72	0.96	-0.22	0.72	0.94	-0.26	0.68	0.95
Lachnospiraceae_NK4A136_grou	-0.79	0.39	0.69	-0.38	0.68	0.93	0.76	0.39	0.85	0.75	0.40	0.84	0.64	0.48	0.89
Lachnospiraceae_UCG.001	-1.01	0.13	0.61	-0.86	0.19	0.58	-0.28	0.67	0.93	-0.29	0.66	0.94	-0.26	0.69	0.95
Lachnospiraceae_UCG.004	-0.85	0.31	0.68	-0.54	0.51	0.85	-0.22	0.78	0.96	-0.22	0.78	0.94	-0.08	0.92	0.99
Lachnospiraceae_UCG.010	-0.72	0.51	0.76	-0.40	0.71	0.95	0.83	0.42	0.85	0.80	0.44	0.84	0.58	0.58	0.89
Lactobacillus	-1.04	0.30	0.68	-1.45	0.14	0.58	-1.00	0.31	0.85	-1.00	0.31	0.82	-0.71	0.48	0.89
Lactococcus	-0.79	0.51	0.76	-0.50	0.67	0.93	0.20	0.86	0.98	0.17	0.88	0.99	0.11	0.92	0.99
Marvinbryantia	-0.59	0.42	0.71	-0.34	0.65	0.93	0.11	0.88	0.99	0.09	0.90	1.00	0.06	0.94	0.99
Megasphaera	0.08	0.89	0.97	-0.16	0.78	0.97	-0.68	0.24	0.85	-0.68	0.24	0.82	-0.61	0.29	0.89
Monoglobus	-0.76	0.41	0.71	-0.56	0.54	0.86	1.04	0.25	0.85	1.03	0.26	0.82	0.91	0.33	0.89
Moryella	-1.72	0.16	0.61	-1.42	0.24	0.61	-0.16	0.89	0.99	-0.20	0.87	0.98	-0.17	0.89	0.99
Negativibacillus	0.33	0.69	0.94	0.55	0.50	0.84	0.40	0.61	0.92	0.41	0.61	0.92	0.31	0.70	0.95
NK4A214_group	-0.20	0.76	0.94	0.10	0.88	0.98	0.77	0.22	0.85	0.76	0.23	0.82	0.73	0.25	0.89
Odoribacter	0.48	0.45	0.74	0.51	0.41	0.78	0.59	0.33	0.85	0.60	0.32	0.82	0.45	0.46	0.89
Oscillibacter	0.18	0.87	0.97	0.37	0.73	0.95	0.75	0.48	0.85	0.72	0.50	0.84	0.74	0.48	0.89
Oscillospira	1.65	0.27	0.68	1.73	0.24	0.61	1.46	0.30	0.85	1.44	0.31	0.82	1.31	0.35	0.89
Oxalobacter	-1.91	0.06	0.53	-1.46	0.15	0.58	-0.74	0.46	0.85	-0.75	0.45	0.84	-1.13	0.27	0.89
Paludicola	-1.23	0.25	0.68	-0.53	0.63	0.93	0.41	0.70	0.95	0.35	0.74	0.94	0.28	0.80	0.99
Parabacteroides	-0.03	0.97	0.99	-0.25	0.74	0.95	-0.45	0.52	0.85	-0.49	0.49	0.84	-0.58	0.41	0.89
Paraprevotella	-0.60	0.25	0.68	-0.68	0.19	0.58	-0.83	0.11	0.67	-0.87	0.10	0.68	-0.77	0.15	0.84
Parasutterella	-0.70	0.23	0.68	-0.69	0.22	0.60	-0.26	0.65	0.93	-0.25	0.67	0.94	-0.39	0.50	0.89
Peptococcus	-0.89	0.37	0.69	-0.53	0.60	0.90	0.20	0.84	0.97	0.16	0.87	0.98	0.12	0.90	0.99
Phascolarctobacterium	-0.17	0.75	0.94	-0.17	0.74	0.95	-0.11	0.83	0.97	-0.13	0.80	0.94	-0.28	0.59	0.89
Phocea	0.92	0.50	0.76	1.23	0.36	0.73	1.64	0.20	0.85	1.64	0.20	0.82	1.61	0.21	0.89
Prevotella	-0.07	0.88	0.97	-0.14	0.75	0.95	-0.33	0.44	0.85	-0.33	0.44	0.84	-0.33	0.45	0.89
Romboutsia	-0.43	0.61	0.86	-0.58	0.48	0.82	-0.82	0.31	0.85	-0.81	0.31	0.82	-0.66	0.42	0.89
Roseburia	-1.47	0.18	0.61	-1.08	0.32	0.72	-0.97	0.37	0.85	-0.96	0.37	0.84	-0.91	0.40	0.89
Ruminococcus	-0.23	0.72	0.94	-0.08	0.91	0.98	0.42	0.52	0.85	0.41	0.52	0.84	0.45	0.49	0.89
Sellimonas	-0.76	0.39	0.69	-0.85	0.33	0.72	-1.19	0.16	0.85	-1.20	0.15	0.82	-1.19	0.16	0.84
Senegalimassilia	-1.10	0.18	0.61	-1.05	0.20	0.58	-0.47	0.56	0.87	-0.50	0.54	0.85	-0.47	0.56	0.89
Shuttleworthia	-0.12	0.91	0.97	0.11	0.92	0.98	-0.02	0.98	1.00	-0.01	1.00	1.00	-0.01	0.99	0.99
Slackia	-1.62	0.03	0.31	-1.73	0.02	0.43	-1.00	0.15	0.85	-1.04	0.14	0.82	-1.14	0.11	0.70
Streptococcus	-0.41	0.72	0.94	-0.44	0.69	0.93	-0.33	0.77	0.96	-0.41	0.72	0.94	-0.11	0.93	0.99
Subdoligranulum	-0.62	0.48	0.76	-0.41	0.63	0.93	-0.51	0.54	0.86	-0.53	0.52	0.84	-0.59	0.47	0.89
Sutterella	-0.25	0.62	0.86	-0.38	0.44	0.80	-0.85	0.07	0.62	-0.86	0.07	0.59	-0.83	0.08	0.61
TM7x	0.24	0.84	0.97	0.50	0.67	0.93	0.12	0.91	1.00	0.10	0.93	1.00	0.09	0.94	0.99
Turicibacter	-1.06	0.33	0.68	-1.50	0.16	0.58	-2.33	0.02	0.35	-2.32	0.03	0.37	-2.23	0.03	0.56
Tuzzerella	2.09	0.01	0.31	2.04	0.01	0.43	1.54	0.06	0.52	1.56	0.06	0.51	1.51	0.06	0.61
UBA1819	1.97	0.12	0.61	2.13	0.09	0.58	1.49	0.22	0.85	1.46	0.23	0.82	1.40	0.25	0.89
UCG.002	-0.24	0.71	0.94	0.05	0.93	0.98	0.42	0.51	0.85	0.40	0.53	0.84	0.26	0.69	0.95
UCG.003	-1.48	0.02	0.31	-1.23	0.06	0.58	-1.04	0.10	0.67	-1.06	0.10	0.68	-1.12	0.08	0.61
UCG.005	-1.18	0.11	0.61	-0.97	0.19	0.58	-0.14	0.85	0.97	-0.19	0.80	0.94	-0.25	0.75	0.97
UCG.009	-1.16	0.30	0.68	-0.83	0.45	0.80	-0.05	0.96	1.00	-0.07	0.95	1.00	-0.14	0.90	0.99
Veillonella	0.09	0.90	0.97	-0.02	0.98	0.99	-0.05	0.94	1.00	-0.05	0.94	1.00	0.06	0.93	0.99
Victivallis	-1.21	0.10	0.61	-1.05	0.15	0.58	-0.60	0.39	0.85	-0.59	0.40	0.84	-0.59	0.40	0.89

Multivariable-adjusted regression models adjusted for:
Model 1: sequencing batch, age, race, gender, study center
Model 2: Model 1, with education

© 2022 Meyer K et al. *JAMA Network Open.*

Model 3: Model 2, with physical activity, diet score, medication use, and smoking
Model 4: Model 3 with BMI
Model 5: Model 4 with hypertension and diabetes

© 2022 Meyer K et al. *JAMA Network Open.*

eTable 49: Multivariable-adjusted regression coefficients, p-values, and false discovery rate (FDR)-adjusted p-values for genera among male participants with respect to RAVLT.

	Model1			Model2			Model3			Model4			Model5		
	beta	p-value	FDR	beta	p-value	FDR	beta	p-value	FDR	beta	p-value	FDR	beta	p-value	FDR
Acetanaerobacterium	-0.08	0.75	0.88	-0.11	0.65	0.88	-0.23	0.35	0.77	-0.23	0.34	0.78	-0.19	0.42	0.78
Acidaminococcus	-0.18	0.01	0.19	-0.13	0.06	0.47	-0.12	0.09	0.46	-0.13	0.07	0.43	-0.15	0.05	0.40
Actinomyces	-0.17	0.39	0.68	-0.16	0.41	0.78	-0.09	0.68	0.88	-0.05	0.80	0.94	-0.13	0.55	0.84
Adlercreutzia	0.12	0.31	0.62	0.14	0.21	0.59	0.13	0.27	0.77	0.12	0.29	0.77	0.14	0.23	0.68
Agathobacter	0.04	0.82	0.93	-0.04	0.79	0.97	-0.12	0.46	0.84	-0.15	0.35	0.78	-0.18	0.28	0.68
Akkermansia	0.09	0.26	0.58	0.03	0.74	0.95	0.02	0.81	0.93	0.02	0.85	0.94	0.03	0.72	0.89
Alistipes	0.25	0.08	0.45	0.15	0.26	0.61	0.09	0.52	0.84	0.09	0.54	0.86	0.17	0.25	0.68
Allisonella	-0.11	0.40	0.68	0.00	0.99	0.99	-0.01	0.97	0.99	-0.01	0.95	0.99	-0.01	0.95	0.97
Anaerofilum	0.21	0.23	0.56	0.12	0.46	0.82	0.13	0.46	0.84	0.13	0.47	0.86	0.17	0.33	0.74
Anaerostipes	0.19	0.34	0.62	0.09	0.65	0.88	0.16	0.42	0.84	0.15	0.45	0.86	0.15	0.45	0.80
Anaerotruncus	0.04	0.81	0.93	-0.04	0.80	0.97	-0.01	0.95	0.99	0.01	0.94	0.99	0.05	0.77	0.89
Bacteroides	0.43	0.09	0.47	0.41	0.08	0.47	0.40	0.10	0.46	0.37	0.12	0.51	0.40	0.10	0.47
Barnesiella	0.10	0.17	0.51	0.11	0.12	0.47	0.11	0.16	0.62	0.11	0.15	0.58	0.09	0.20	0.66
Bifidobacterium	0.02	0.86	0.94	0.07	0.43	0.78	0.06	0.54	0.84	0.05	0.60	0.87	0.04	0.63	0.88
Bilophila	0.15	0.17	0.51	0.07	0.49	0.85	0.08	0.46	0.84	0.06	0.56	0.86	0.06	0.60	0.88
Blautia	0.46	0.19	0.52	0.24	0.47	0.83	0.24	0.50	0.84	0.24	0.49	0.86	0.24	0.48	0.82
Butyricicoccus	0.12	0.67	0.85	-0.14	0.60	0.88	-0.16	0.57	0.84	-0.15	0.57	0.86	-0.10	0.72	0.89
Butyricimonas	-0.06	0.50	0.75	-0.10	0.25	0.60	-0.09	0.29	0.77	-0.10	0.28	0.77	-0.08	0.36	0.74
CAG.56	0.16	0.10	0.49	0.11	0.24	0.59	0.10	0.29	0.77	0.10	0.28	0.77	0.12	0.21	0.66
Candidatus_Soleaferrea	-0.11	0.57	0.77	-0.22	0.22	0.59	-0.31	0.10	0.46	-0.31	0.10	0.49	-0.27	0.14	0.56
Caproiciproducens	-0.24	0.18	0.51	-0.28	0.10	0.47	-0.33	0.06	0.44	-0.33	0.06	0.43	-0.32	0.07	0.40
Catenibacterium	-0.13	0.17	0.51	-0.13	0.12	0.47	-0.09	0.33	0.77	-0.09	0.35	0.78	-0.10	0.27	0.68
Christensenellaceae_R.7_group	0.03	0.70	0.87	-0.02	0.77	0.96	-0.05	0.53	0.84	-0.05	0.57	0.86	-0.03	0.73	0.89
Clostridium_sensu_stricto_1	0.05	0.62	0.81	0.09	0.34	0.72	0.13	0.22	0.70	0.13	0.21	0.70	0.09	0.39	0.78
Colidextribacter	-0.19	0.35	0.63	-0.32	0.09	0.47	-0.37	0.06	0.44	-0.38	0.06	0.43	-0.39	0.05	0.40
Collinsella	-0.16	0.10	0.49	-0.13	0.18	0.56	-0.17	0.09	0.46	-0.16	0.10	0.49	-0.14	0.14	0.56
Coprobacter	0.02	0.84	0.94	-0.01	0.88	0.97	-0.04	0.65	0.87	-0.04	0.70	0.89	-0.01	0.90	0.93
Coprococcus	0.29	0.01	0.19	0.20	0.09	0.47	0.17	0.16	0.62	0.18	0.13	0.51	0.22	0.07	0.40
Defluviitaleaceae_UCG.011	0.29	0.08	0.45	0.29	0.06	0.47	0.38	0.02	0.42	0.39	0.02	0.41	0.42	0.01	0.35
Desulfovibrio	-0.12	0.16	0.51	-0.12	0.14	0.52	-0.12	0.18	0.65	-0.10	0.26	0.77	-0.08	0.36	0.74
Dialister	0.10	0.15	0.51	0.10	0.11	0.47	0.08	0.26	0.76	0.08	0.29	0.77	0.06	0.42	0.78
Dorea	0.02	0.90	0.96	-0.05	0.72	0.95	-0.08	0.58	0.84	-0.07	0.62	0.88	-0.06	0.71	0.89
DTU089	-0.01	0.94	0.98	-0.02	0.89	0.97	-0.02	0.88	0.96	0.00	0.99	1.00	0.03	0.83	0.89
Eggerthella	0.21	0.13	0.51	0.17	0.20	0.59	0.22	0.13	0.50	0.23	0.11	0.49	0.24	0.09	0.43
Eisenbergiella	-0.10	0.55	0.77	-0.08	0.59	0.88	-0.11	0.49	0.84	-0.09	0.56	0.86	-0.12	0.45	0.80
Enterorhabdus	-0.17	0.20	0.53	-0.23	0.07	0.47	-0.24	0.09	0.46	-0.23	0.10	0.49	-0.21	0.13	0.55
Erysipelatoclostridium	0.22	0.15	0.51	0.27	0.07	0.47	0.31	0.04	0.44	0.33	0.03	0.41	0.30	0.05	0.40
Erysipelotrichaceae_UCG.003	0.01	0.91	0.97	-0.01	0.91	0.97	-0.06	0.59	0.84	-0.06	0.57	0.86	-0.04	0.69	0.89
Escherichia.Shigella	-0.08	0.43	0.69	0.00	0.98	0.99	0.03	0.79	0.93	0.02	0.85	0.94	0.03	0.77	0.89
Faecalibacterium	0.24	0.16	0.51	0.26	0.11	0.47	0.31	0.07	0.45	0.32	0.07	0.43	0.33	0.05	0.40
Family_XIII_AD3011_group	0.22	0.11	0.50	0.07	0.60	0.88	0.04	0.79	0.93	0.07	0.65	0.88	0.11	0.43	0.78
Family_XIII_UCG.001	-0.05	0.74	0.88	-0.12	0.41	0.78	-0.10	0.56	0.84	-0.07	0.68	0.88	-0.03	0.84	0.89
FD2005	-0.04	0.80	0.93	-0.01	0.93	0.97	-0.09	0.53	0.84	-0.12	0.42	0.85	-0.10	0.50	0.82
Flavonifractor	-0.01	0.96	0.99	0.02	0.90	0.97	-0.01	0.92	0.99	-0.02	0.89	0.97	-0.04	0.77	0.89
Fournierella	0.08	0.57	0.77	0.06	0.63	0.88	0.09	0.52	0.84	0.10	0.46	0.86	0.14	0.32	0.74
Frisingicoccus	-0.02	0.85	0.94	0.02	0.88	0.97	-0.05	0.68	0.88	-0.06	0.63	0.88	-0.04	0.73	0.89
Fusicatenibacter	0.04	0.74	0.88	0.01	0.92	0.97	-0.03	0.83	0.93	-0.04	0.77	0.93	-0.03	0.83	0.89
Fusobacterium	0.00	0.98	0.99	0.09	0.57	0.88	0.13	0.41	0.84	0.15	0.34	0.78	0.17	0.28	0.68
GCA.900066575	-0.11	0.45	0.70	-0.18	0.18	0.58	-0.18	0.21	0.70	-0.17	0.24	0.77	-0.14	0.35	0.74
Gordonibacter	0.18	0.27	0.58	0.13	0.39	0.78	0.15	0.35	0.77	0.17	0.30	0.77	0.21	0.19	0.66
Granulicatella	0.04	0.86	0.94	0.13	0.52	0.88	0.15	0.50	0.84	0.16	0.47	0.86	0.11	0.62	0.88
Haemophilus	0.14	0.17	0.51	0.14	0.15	0.53	0.14	0.19	0.66	0.15	0.17	0.63	0.16	0.15	0.56
Harryflintia	-0.01	0.97	0.99	-0.09	0.60	0.88	-0.05	0.80	0.93	-0.04	0.82	0.94	0.01	0.98	0.99
Holdemanella	-0.08	0.44	0.70	-0.08	0.38	0.78	-0.11	0.28	0.77	-0.11	0.28	0.77	-0.09	0.35	0.74

© 2022 Meyer K et al. *JAMA Network Open.*

Holdemania	-0.01	0.98	0.99	0.04	0.88	0.97	-0.10	0.74	0.93	-0.06	0.84	0.94	0.00	1.00	1.00
Howardella	-0.23	0.06	0.45	-0.26	0.02	0.35	-0.24	0.05	0.44	-0.23	0.07	0.43	-0.23	0.06	0.40
Hungatella	-0.10	0.52	0.75	-0.07	0.64	0.88	-0.08	0.61	0.84	-0.06	0.68	0.88	-0.10	0.49	0.82
Hydrogenoanaerobacterium	0.28	0.11	0.50	0.14	0.39	0.78	0.17	0.34	0.77	0.18	0.31	0.77	0.20	0.25	0.68
Incertae_Sedis	0.23	0.36	0.63	0.25	0.28	0.63	0.15	0.56	0.84	0.14	0.57	0.86	0.15	0.55	0.84
Intestinibacter	0.28	0.03	0.29	0.34	0.01	0.34	0.31	0.02	0.42	0.30	0.02	0.41	0.31	0.02	0.40
Intestinimonas	0.20	0.12	0.51	0.06	0.66	0.88	0.06	0.65	0.87	0.06	0.66	0.88	0.11	0.41	0.78
Lachnoclostridium	-0.32	0.24	0.56	-0.25	0.33	0.72	-0.14	0.60	0.84	-0.17	0.54	0.86	-0.22	0.41	0.78
Lachnospira	0.31	0.02	0.23	0.19	0.16	0.53	0.14	0.33	0.77	0.13	0.37	0.79	0.15	0.28	0.68
Lachnospiraceae_FCS020_group	0.24	0.07	0.45	0.15	0.22	0.59	0.14	0.32	0.77	0.14	0.31	0.77	0.15	0.26	0.68
Lachnospiraceae_ND3007_grou	0.22	0.02	0.20	0.15	0.08	0.47	0.15	0.11	0.48	0.15	0.11	0.49	0.17	0.07	0.40
Lachnospiraceae_NK4A136_gro	0.18	0.17	0.51	0.08	0.55	0.88	-0.01	0.96	0.99	0.00	1.00	1.00	0.04	0.76	0.89
Lachnospiraceae_UCG.001	0.25	0.01	0.19	0.21	0.02	0.35	0.22	0.03	0.44	0.22	0.02	0.41	0.22	0.02	0.40
Lachnospiraceae_UCG.004	0.08	0.51	0.75	0.00	0.98	0.99	-0.02	0.87	0.96	-0.02	0.86	0.94	-0.06	0.63	0.88
Lachnospiraceae_UCG.010	-0.05	0.75	0.88	-0.13	0.36	0.76	-0.15	0.33	0.77	-0.13	0.40	0.83	-0.09	0.55	0.84
Lactobacillus	-0.39	0.01	0.19	-0.30	0.03	0.35	-0.36	0.01	0.42	-0.36	0.01	0.41	-0.46	0.00	0.16
Lactococcus	-0.03	0.88	0.95	-0.10	0.52	0.88	-0.05	0.76	0.93	-0.04	0.84	0.94	-0.03	0.85	0.89
Marvinbryantia	0.08	0.45	0.70	0.01	0.89	0.97	0.03	0.80	0.93	0.03	0.75	0.93	0.05	0.61	0.88
Megasphaera	-0.23	0.01	0.19	-0.17	0.03	0.39	-0.16	0.06	0.44	-0.16	0.06	0.43	-0.17	0.04	0.40
Monoglobus	0.33	0.01	0.19	0.28	0.02	0.35	0.29	0.03	0.44	0.30	0.03	0.41	0.31	0.02	0.40
Moryella	0.17	0.33	0.62	0.09	0.57	0.88	0.00	1.00	1.00	0.02	0.92	0.97	0.03	0.88	0.92
Negativibacillus	-0.13	0.26	0.58	-0.19	0.09	0.47	-0.21	0.08	0.46	-0.21	0.08	0.43	-0.19	0.11	0.51
NK4A214_group	0.09	0.33	0.62	0.02	0.86	0.97	0.02	0.82	0.93	0.03	0.76	0.93	0.06	0.53	0.84
Odoribacter	0.11	0.22	0.56	0.10	0.24	0.59	0.09	0.32	0.77	0.09	0.34	0.78	0.12	0.19	0.66
Oscillibacter	0.20	0.19	0.52	0.16	0.28	0.63	0.06	0.72	0.92	0.07	0.65	0.88	0.08	0.62	0.88
Oscillospira	0.13	0.56	0.77	0.10	0.62	0.88	0.03	0.87	0.96	0.05	0.81	0.94	0.08	0.69	0.89
Oxalobacter	0.18	0.21	0.55	0.06	0.65	0.88	0.12	0.43	0.84	0.12	0.41	0.84	0.19	0.21	0.66
Paludicola	0.17	0.28	0.58	-0.01	0.93	0.97	0.01	0.95	0.99	0.05	0.78	0.93	0.09	0.57	0.87
Parabacteroides	-0.11	0.32	0.62	-0.05	0.61	0.88	-0.06	0.59	0.84	-0.04	0.70	0.89	-0.03	0.79	0.89
Paraprevotella	-0.04	0.62	0.81	-0.02	0.80	0.97	0.00	0.98	0.99	0.01	0.92	0.97	-0.03	0.74	0.89
Parasutterella	0.15	0.07	0.45	0.15	0.06	0.47	0.08	0.34	0.77	0.08	0.37	0.79	0.11	0.21	0.66
Peptococcus	-0.09	0.51	0.75	-0.19	0.16	0.53	-0.29	0.04	0.44	-0.28	0.06	0.43	-0.25	0.08	0.43
Phascolarctobacterium	-0.06	0.43	0.69	-0.06	0.41	0.78	-0.06	0.44	0.84	-0.05	0.51	0.86	-0.02	0.78	0.89
Phocea	-0.23	0.24	0.56	-0.31	0.09	0.47	-0.36	0.06	0.44	-0.36	0.06	0.43	-0.36	0.06	0.40
Prevotella	-0.16	0.01	0.19	-0.14	0.02	0.35	-0.13	0.04	0.44	-0.13	0.04	0.43	-0.14	0.03	0.40
Romboutsia	0.10	0.42	0.69	0.13	0.24	0.59	0.08	0.49	0.84	0.08	0.50	0.86	0.06	0.64	0.88
Roseburia	0.15	0.34	0.62	0.05	0.76	0.96	-0.10	0.52	0.84	-0.11	0.49	0.86	-0.10	0.51	0.83
Ruminococcus	0.14	0.13	0.51	0.10	0.25	0.60	0.07	0.46	0.84	0.07	0.44	0.86	0.08	0.42	0.78
Sellimonas	0.13	0.31	0.62	0.15	0.22	0.59	0.15	0.24	0.73	0.16	0.21	0.70	0.15	0.24	0.68
Senegalimassilia	-0.19	0.10	0.49	-0.21	0.06	0.47	-0.22	0.06	0.44	-0.21	0.08	0.43	-0.23	0.05	0.40
Shuttleworthia	0.29	0.06	0.45	0.23	0.12	0.47	0.25	0.12	0.48	0.24	0.13	0.51	0.22	0.15	0.56
Slackia	-0.26	0.01	0.19	-0.23	0.02	0.35	-0.25	0.02	0.42	-0.24	0.02	0.41	-0.22	0.03	0.40
Streptococcus	0.00	0.99	0.99	0.01	0.94	0.97	0.05	0.75	0.93	0.09	0.58	0.86	0.04	0.82	0.89
Subdoligranulum	0.10	0.41	0.68	0.05	0.66	0.88	-0.01	0.95	0.99	0.00	0.99	1.00	0.03	0.81	0.89
Sutterella	-0.14	0.05	0.44	-0.10	0.12	0.47	-0.06	0.39	0.84	-0.05	0.44	0.86	-0.07	0.34	0.74
TM7x	0.08	0.63	0.81	0.02	0.88	0.97	0.09	0.57	0.84	0.11	0.52	0.86	0.11	0.50	0.82
Turicibacter	0.37	0.02	0.20	0.48	0.00	0.11	0.47	0.00	0.26	0.45	0.00	0.36	0.45	0.00	0.19
Tuzzerella	-0.14	0.23	0.56	-0.13	0.24	0.59	-0.21	0.08	0.46	-0.22	0.07	0.43	-0.21	0.09	0.43
UBA1819	-0.09	0.61	0.81	-0.13	0.43	0.78	-0.21	0.26	0.76	-0.19	0.31	0.77	-0.17	0.36	0.74
UCG.002	0.04	0.63	0.81	-0.03	0.74	0.95	-0.06	0.55	0.84	-0.05	0.61	0.88	-0.01	0.91	0.93
UCG.003	0.06	0.53	0.76	-0.01	0.90	0.97	-0.05	0.62	0.85	-0.04	0.68	0.88	-0.02	0.81	0.89
UCG.005	0.04	0.73	0.88	-0.02	0.87	0.97	-0.03	0.82	0.93	-0.01	0.96	0.99	0.02	0.83	0.89
UCG.009	0.16	0.32	0.62	0.08	0.59	0.88	0.01	0.96	0.99	0.02	0.91	0.97	0.06	0.72	0.89
Veillonella	0.12	0.27	0.58	0.15	0.15	0.53	0.14	0.20	0.68	0.14	0.20	0.69	0.13	0.23	0.68
Victivallis	0.04	0.72	0.88	0.00	0.98	0.99	-0.03	0.79	0.93	-0.03	0.78	0.93	-0.03	0.78	0.89

Multivariable-adjusted regression models adjusted for:
Model 1: sequencing batch, age, race, gender, study center
Model 2: Model 1, with education

© 2022 Meyer K et al. *JAMA Network Open.*

Model 3: Model 2, with physical activity, diet score, medication use, and smoking
Model 4: Model 3 with BMI
Model 5: Model 4 with hypertension and diabetes

© 2022 Meyer K et al. *JAMA Network Open.*

eTable 50: Multivariable-adjusted regression coefficients, p-values, and false discovery rate (FDR)-adjusted p-values for genera among male participants with respect to category fluency.

	Model1			Model2			Model3			Model4			Model5		
	beta	p-value	FDR	beta	p-value	FDR	beta	p-value	FDR	beta	p-value	FDR	beta	p-value	FDR
Acetanaerobacterium	-0.14	0.85	0.93	-0.20	0.78	0.88	-0.42	0.58	0.79	-0.42	0.58	0.79	-0.39	0.61	0.81
Acidaminococcus	-0.24	0.26	0.49	-0.13	0.52	0.71	-0.16	0.50	0.73	-0.15	0.52	0.76	-0.17	0.47	0.70
Actinomyces	0.41	0.50	0.69	0.44	0.45	0.64	0.61	0.35	0.59	0.58	0.37	0.63	0.52	0.43	0.67
Adlercreutzia	0.06	0.87	0.93	0.10	0.77	0.88	-0.04	0.91	0.93	-0.03	0.93	0.94	-0.02	0.97	0.97
Agathobacter	0.98	0.04	0.32	0.82	0.09	0.31	0.81	0.11	0.40	0.86	0.09	0.36	0.85	0.10	0.37
Akkermansia	-0.20	0.41	0.64	-0.36	0.14	0.39	-0.48	0.07	0.39	-0.48	0.07	0.36	-0.47	0.08	0.37
Alistipes	0.01	0.98	0.99	-0.22	0.60	0.78	-0.45	0.31	0.55	-0.45	0.32	0.56	-0.40	0.38	0.64
Allisonella	0.06	0.88	0.94	0.32	0.41	0.63	0.24	0.58	0.79	0.24	0.58	0.79	0.23	0.59	0.81
Anaerofilum	-0.33	0.54	0.71	-0.53	0.30	0.55	-0.64	0.25	0.50	-0.64	0.25	0.51	-0.62	0.27	0.52
Anaerostipes	-0.39	0.51	0.69	-0.62	0.28	0.55	-0.57	0.36	0.61	-0.55	0.37	0.63	-0.59	0.35	0.61
Anaerotruncus	-0.76	0.11	0.40	-0.94	0.04	0.29	-0.97	0.05	0.38	-1.00	0.05	0.36	-0.99	0.05	0.37
Bacteroides	0.24	0.74	0.86	0.22	0.76	0.88	0.31	0.68	0.84	0.34	0.65	0.82	0.39	0.61	0.81
Barnesiella	0.58	0.01	0.11	0.60	0.01	0.13	0.56	0.02	0.24	0.56	0.02	0.25	0.56	0.02	0.27
Bifidobacterium	0.40	0.16	0.40	0.53	0.06	0.29	0.47	0.10	0.39	0.48	0.09	0.36	0.47	0.10	0.37
Bilophila	0.87	0.01	0.11	0.71	0.02	0.26	0.68	0.05	0.36	0.71	0.04	0.33	0.74	0.03	0.32
Blautia	-0.67	0.52	0.69	-1.19	0.24	0.49	-1.10	0.31	0.55	-1.10	0.31	0.56	-1.09	0.32	0.58
Butyricicoccus	1.03	0.20	0.45	0.49	0.53	0.71	0.40	0.64	0.83	0.40	0.64	0.82	0.39	0.65	0.81
Butyricimonas	0.34	0.21	0.45	0.26	0.32	0.55	0.30	0.27	0.51	0.30	0.27	0.53	0.33	0.24	0.50
CAG.56	0.50	0.08	0.39	0.39	0.16	0.40	0.38	0.20	0.47	0.38	0.21	0.47	0.41	0.18	0.44
Candidatus_Soleaferrea	-0.63	0.26	0.49	-0.88	0.11	0.35	-0.96	0.10	0.39	-0.96	0.10	0.36	-0.95	0.11	0.37
Caproiciproducens	-1.20	0.02	0.22	-1.29	0.01	0.19	-1.37	0.01	0.24	-1.36	0.01	0.25	-1.40	0.01	0.25
Catenibacterium	0.04	0.87	0.93	0.02	0.93	0.95	0.08	0.77	0.88	0.08	0.78	0.87	0.06	0.85	0.90
Christensenellaceae_R.7_group	0.34	0.18	0.43	0.21	0.39	0.61	0.10	0.71	0.85	0.09	0.73	0.86	0.10	0.71	0.85
Clostridium_sensu_stricto_1	0.42	0.16	0.40	0.51	0.08	0.29	0.59	0.07	0.39	0.58	0.07	0.36	0.56	0.09	0.37
Colidextribacter	-0.58	0.33	0.58	-0.87	0.13	0.39	-0.75	0.23	0.47	-0.75	0.23	0.48	-0.80	0.21	0.47
Collinsella	0.43	0.15	0.40	0.51	0.08	0.29	0.47	0.13	0.42	0.47	0.13	0.41	0.47	0.13	0.40
Coprobacter	0.54	0.07	0.38	0.47	0.10	0.35	0.37	0.22	0.47	0.37	0.23	0.48	0.40	0.19	0.44
Coprococcus	0.67	0.06	0.36	0.46	0.20	0.44	0.42	0.26	0.51	0.41	0.28	0.53	0.43	0.26	0.52
Defluviitaleaceae_UCG.011	-0.17	0.74	0.86	-0.17	0.73	0.88	-0.19	0.71	0.85	-0.19	0.71	0.85	-0.18	0.73	0.86
Desulfovibrio	-0.37	0.16	0.40	-0.37	0.15	0.40	-0.49	0.08	0.39	-0.53	0.06	0.36	-0.51	0.08	0.37
Dialister	-0.04	0.85	0.93	-0.03	0.87	0.93	-0.07	0.75	0.87	-0.07	0.77	0.87	-0.10	0.66	0.81
Dorea	0.49	0.28	0.50	0.34	0.44	0.64	0.31	0.51	0.74	0.30	0.52	0.76	0.32	0.50	0.72
DTU089	-0.53	0.23	0.46	-0.55	0.20	0.44	-0.72	0.11	0.40	-0.77	0.10	0.36	-0.76	0.11	0.37
Eggerthella	-0.70	0.10	0.40	-0.79	0.05	0.29	-0.69	0.12	0.41	-0.70	0.12	0.40	-0.70	0.12	0.39
Eisenbergiella	-1.36	0.00	0.10	-1.32	0.00	0.12	-1.43	0.00	0.16	-1.47	0.00	0.14	-1.49	0.00	0.12
Enterorhabdus	-0.32	0.43	0.64	-0.45	0.24	0.49	-0.52	0.23	0.47	-0.53	0.23	0.48	-0.54	0.22	0.49
Erysipelatoclostridium	-0.96	0.04	0.32	-0.87	0.05	0.29	-0.70	0.14	0.42	-0.73	0.13	0.41	-0.76	0.12	0.39
Erysipelotrichaceae_UCG.003	0.50	0.13	0.40	0.44	0.16	0.40	0.34	0.31	0.55	0.35	0.31	0.56	0.35	0.30	0.56
Escherichia.Shigella	-0.41	0.19	0.44	-0.24	0.45	0.64	-0.24	0.48	0.72	-0.23	0.50	0.74	-0.21	0.53	0.76
Faecalibacterium	0.47	0.35	0.59	0.51	0.30	0.55	0.76	0.16	0.43	0.75	0.17	0.43	0.74	0.18	0.44
Family_XIII_AD3011_group	0.31	0.45	0.65	-0.03	0.94	0.95	-0.14	0.75	0.87	-0.17	0.70	0.85	-0.15	0.74	0.87
Family_XIII_UCG.001	0.17	0.72	0.86	0.02	0.97	0.97	-0.13	0.79	0.88	-0.17	0.75	0.86	-0.15	0.78	0.89
FD2005	-0.61	0.15	0.40	-0.56	0.17	0.41	-0.66	0.14	0.42	-0.65	0.15	0.43	-0.66	0.15	0.42
Flavonifractor	-0.30	0.44	0.65	-0.25	0.50	0.69	-0.11	0.79	0.88	-0.11	0.80	0.88	-0.13	0.75	0.87
Fournierella	0.00	1.00	1.00	-0.04	0.93	0.95	-0.19	0.67	0.84	-0.20	0.64	0.82	-0.19	0.67	0.81
Frisingicoccus	-0.42	0.26	0.49	-0.33	0.36	0.59	-0.33	0.39	0.63	-0.32	0.41	0.65	-0.30	0.45	0.69
Fusicatenibacter	0.59	0.11	0.40	0.53	0.15	0.40	0.53	0.17	0.43	0.54	0.16	0.43	0.55	0.16	0.43
Fusobacterium	-0.31	0.51	0.69	-0.12	0.80	0.88	-0.05	0.93	0.93	-0.07	0.89	0.93	-0.02	0.97	0.97
GCA.900066575	0.61	0.17	0.40	0.45	0.29	0.55	0.37	0.41	0.65	0.36	0.42	0.67	0.37	0.42	0.67
Gordonibacter	-0.36	0.46	0.66	-0.47	0.32	0.55	-0.28	0.58	0.79	-0.30	0.56	0.79	-0.27	0.60	0.81
Granulicatella	-0.34	0.59	0.75	-0.14	0.82	0.88	0.07	0.92	0.93	0.06	0.93	0.94	0.03	0.97	0.97
Haemophilus	0.60	0.05	0.34	0.60	0.05	0.29	0.57	0.09	0.39	0.57	0.09	0.36	0.56	0.10	0.37
Harryflintia	-0.77	0.14	0.40	-0.95	0.06	0.29	-1.19	0.03	0.36	-1.20	0.03	0.33	-1.17	0.04	0.32
Holdemanella	0.44	0.15	0.40	0.43	0.14	0.40	0.54	0.09	0.39	0.54	0.09	0.36	0.56	0.08	0.37

© 2022 Meyer K et al. *JAMA Network Open.*

Holdemania	-1.40	0.09	0.40	-1.30	0.11	0.35	-1.45	0.10	0.39	-1.51	0.09	0.36	-1.46	0.10	0.37
Howardella	-0.30	0.42	0.64	-0.36	0.32	0.55	-0.28	0.47	0.72	-0.30	0.44	0.69	-0.31	0.43	0.67
Hungatella	-1.73	0.00	0.01	-1.67	0.00	0.01	-1.63	0.00	0.02	-1.66	0.00	0.02	-1.71	0.00	0.01
Hydrogenoanaerobacterium	0.34	0.52	0.69	0.04	0.94	0.95	-0.29	0.60	0.81	-0.31	0.58	0.79	-0.32	0.57	0.79
Incertae_Sedis	-0.80	0.28	0.50	-0.74	0.30	0.55	-0.75	0.34	0.59	-0.74	0.35	0.60	-0.72	0.36	0.62
Intestinibacter	0.55	0.16	0.40	0.67	0.08	0.29	0.55	0.18	0.44	0.59	0.16	0.43	0.57	0.18	0.44
Intestinimonas	-0.34	0.38	0.61	-0.69	0.07	0.29	-0.92	0.03	0.36	-0.92	0.03	0.33	-0.90	0.03	0.32
Lachnoclostridium	-1.15	0.16	0.40	-1.01	0.21	0.45	-0.55	0.52	0.74	-0.53	0.54	0.77	-0.54	0.53	0.76
Lachnospira	1.21	0.00	0.10	0.95	0.02	0.24	0.65	0.15	0.43	0.67	0.14	0.41	0.67	0.14	0.40
Lachnospiraceae_FCS020_group	0.56	0.15	0.40	0.37	0.33	0.55	0.37	0.38	0.63	0.37	0.39	0.64	0.37	0.39	0.65
Lachnospiraceae_ND3007_group	0.72	0.01	0.11	0.59	0.03	0.29	0.61	0.04	0.36	0.62	0.04	0.33	0.62	0.03	0.32
Lachnospiraceae_NK4A136_group	0.36	0.36	0.59	0.13	0.74	0.88	-0.20	0.63	0.83	-0.21	0.62	0.82	-0.19	0.65	0.81
Lachnospiraceae_UCG.001	0.02	0.93	0.97	-0.07	0.81	0.88	-0.22	0.47	0.72	-0.22	0.47	0.71	-0.23	0.46	0.69
Lachnospiraceae_UCG.004	0.36	0.31	0.56	0.18	0.60	0.78	0.17	0.65	0.83	0.17	0.64	0.82	0.14	0.70	0.85
Lachnospiraceae_UCG.010	-0.67	0.15	0.40	-0.86	0.06	0.29	-0.87	0.07	0.39	-0.90	0.06	0.36	-0.86	0.08	0.37
Lactobacillus	-0.52	0.21	0.45	-0.32	0.44	0.64	-0.34	0.45	0.70	-0.34	0.45	0.69	-0.42	0.36	0.62
Lactococcus	0.91	0.07	0.38	0.74	0.13	0.39	0.89	0.09	0.39	0.87	0.10	0.36	0.89	0.10	0.37
Marvinbryantia	0.27	0.39	0.61	0.13	0.68	0.85	0.15	0.64	0.83	0.15	0.66	0.82	0.15	0.65	0.81
Megasphaera	-0.22	0.36	0.59	-0.09	0.70	0.86	0.05	0.85	0.91	0.05	0.84	0.91	0.04	0.90	0.93
Monoglobus	0.35	0.37	0.60	0.24	0.52	0.71	0.05	0.91	0.93	0.04	0.92	0.94	0.09	0.84	0.90
Moryella	0.99	0.06	0.36	0.83	0.10	0.35	0.60	0.27	0.51	0.59	0.29	0.54	0.58	0.30	0.56
Negativibacillus	-0.40	0.25	0.49	-0.52	0.13	0.39	-0.63	0.09	0.39	-0.63	0.09	0.36	-0.61	0.11	0.37
NK4A214_group	-0.15	0.59	0.75	-0.32	0.24	0.49	-0.50	0.09	0.39	-0.51	0.08	0.36	-0.52	0.08	0.37
Odoribacter	0.54	0.04	0.32	0.52	0.04	0.29	0.43	0.13	0.42	0.43	0.13	0.41	0.47	0.10	0.37
Oscillibacter	-0.03	0.95	0.98	-0.13	0.77	0.88	-0.19	0.70	0.85	-0.21	0.67	0.82	-0.22	0.66	0.81
Oscillospira	-0.12	0.86	0.93	-0.17	0.78	0.88	-0.19	0.77	0.88	-0.21	0.75	0.86	-0.18	0.78	0.89
Oxalobacter	0.68	0.12	0.40	0.43	0.32	0.55	0.26	0.57	0.79	0.26	0.58	0.79	0.34	0.48	0.70
Paludicola	-0.20	0.67	0.81	-0.62	0.17	0.41	-1.00	0.04	0.36	-1.07	0.03	0.33	-1.08	0.03	0.32
Parabacteroides	0.45	0.16	0.40	0.58	0.06	0.29	0.65	0.05	0.36	0.65	0.05	0.36	0.67	0.04	0.34
Paraprevotella	0.05	0.82	0.93	0.09	0.67	0.85	0.09	0.72	0.85	0.08	0.75	0.86	0.06	0.81	0.90
Parasutterella	0.59	0.01	0.16	0.59	0.01	0.19	0.45	0.09	0.39	0.46	0.08	0.36	0.50	0.06	0.37
Peptococcus	-0.62	0.15	0.40	-0.84	0.04	0.29	-0.95	0.03	0.36	-0.98	0.03	0.33	-0.99	0.03	0.32
Phascolarctobacterium	0.28	0.22	0.45	0.28	0.20	0.44	0.33	0.17	0.43	0.32	0.18	0.44	0.37	0.14	0.40
Phocea	-0.95	0.10	0.40	-1.13	0.04	0.29	-1.51	0.01	0.24	-1.50	0.01	0.25	-1.49	0.01	0.25
Prevotella	0.04	0.84	0.93	0.08	0.67	0.85	0.03	0.87	0.92	0.03	0.87	0.91	0.03	0.89	0.93
Romboutsia	0.53	0.14	0.40	0.61	0.08	0.29	0.49	0.19	0.46	0.49	0.19	0.46	0.46	0.23	0.49
Roseburia	1.31	0.00	0.10	1.10	0.01	0.20	0.75	0.14	0.42	0.75	0.13	0.41	0.75	0.14	0.40
Ruminococcus	0.47	0.10	0.40	0.38	0.16	0.40	0.26	0.39	0.63	0.26	0.40	0.64	0.25	0.42	0.67
Sellimonas	0.35	0.35	0.59	0.40	0.28	0.55	0.42	0.28	0.53	0.41	0.29	0.54	0.42	0.29	0.56
Senegalimassilia	0.44	0.21	0.45	0.41	0.23	0.49	0.48	0.19	0.46	0.47	0.21	0.47	0.47	0.21	0.47
Shuttleworthia	-0.22	0.64	0.79	-0.35	0.43	0.64	-0.61	0.21	0.47	-0.60	0.22	0.48	-0.60	0.23	0.49
Slackia	-0.01	0.97	0.99	0.04	0.89	0.93	0.04	0.91	0.93	0.03	0.94	0.94	0.04	0.91	0.93
Streptococcus	0.32	0.51	0.69	0.34	0.47	0.66	0.60	0.25	0.50	0.58	0.27	0.53	0.53	0.33	0.59
Subdoligranulum	0.78	0.04	0.32	0.66	0.07	0.29	0.54	0.16	0.43	0.53	0.17	0.43	0.54	0.17	0.43
Sutterella	0.09	0.67	0.81	0.16	0.42	0.64	0.27	0.22	0.47	0.26	0.23	0.48	0.26	0.25	0.50
TM7x	0.05	0.92	0.96	-0.07	0.88	0.93	0.12	0.82	0.90	0.11	0.83	0.91	0.10	0.84	0.90
Turicibacter	1.53	0.00	0.05	1.79	0.00	0.01	1.80	0.00	0.02	1.84	0.00	0.02	1.83	0.00	0.01
Tuzzerella	0.07	0.85	0.93	0.09	0.80	0.88	0.06	0.88	0.92	0.06	0.87	0.91	0.07	0.85	0.90
UBA1819	-1.40	0.01	0.11	-1.49	0.00	0.12	-1.54	0.01	0.17	-1.58	0.01	0.14	-1.57	0.01	0.16
UCG.002	-0.10	0.73	0.86	-0.26	0.33	0.55	-0.36	0.22	0.47	-0.37	0.21	0.47	-0.35	0.24	0.50
UCG.003	0.38	0.17	0.41	0.23	0.40	0.62	0.14	0.65	0.83	0.13	0.67	0.82	0.14	0.65	0.81
UCG.005	0.24	0.44	0.65	0.13	0.68	0.85	-0.08	0.83	0.90	-0.10	0.78	0.87	-0.10	0.79	0.89
UCG.009	-0.23	0.62	0.77	-0.41	0.37	0.60	-0.69	0.16	0.43	-0.70	0.15	0.43	-0.72	0.15	0.41
Veillonella	0.58	0.07	0.38	0.64	0.04	0.29	0.65	0.06	0.38	0.65	0.06	0.36	0.63	0.07	0.37
Victivallis	0.17	0.58	0.75	0.09	0.77	0.88	-0.06	0.85	0.91	-0.06	0.85	0.91	-0.06	0.85	0.90

Multivariable-adjusted regression models adjusted for:
Model 1: sequencing batch, age, race, gender, study center
Model 2: Model 1, with education

© 2022 Meyer K et al. *JAMA Network Open.*

Model 3: Model 2, with physical activity, diet score, medication use, and smoking
Model 4: Model 3 with BMI
Model 5: Model 4 with hypertension and diabetes

© 2022 Meyer K et al. *JAMA Network Open.*

eTable 51: Multivariable-adjusted regression coefficients, p-values, and false discovery rate (FDR)-adjusted p-values for genera among male participants with respect to letter fluency.

	Model1			Model2			Model3			Model4			Model5		
	beta	p-value	FDR	beta	p-value	FDR	beta	p-value	FDR	beta	p-value	FDR	beta	p-value	FDR
Acetanaerobacterium	1.59	0.39	0.90	1.36	0.44	0.96	1.21	0.52	0.96	1.21	0.52	0.96	1.21	0.52	0.96
Acidaminococcus	-1.02	0.05	0.64	-0.71	0.16	0.94	-0.54	0.34	0.96	-0.55	0.33	0.96	-0.58	0.31	0.96
Actinomyces	1.15	0.45	0.90	1.22	0.40	0.94	0.95	0.54	0.96	1.01	0.53	0.96	0.94	0.56	0.96
Adlercreutzia	-0.48	0.58	0.90	-0.38	0.65	0.98	-0.38	0.67	0.97	-0.39	0.66	0.97	-0.37	0.67	0.97
Agathobacter	0.77	0.56	0.90	0.26	0.83	0.98	-0.32	0.81	1.00	-0.37	0.78	0.99	-0.34	0.80	1.00
Akkermansia	-0.39	0.53	0.90	-0.84	0.16	0.94	-0.93	0.15	0.96	-0.94	0.14	0.96	-0.93	0.15	0.96
Alistipes	1.83	0.09	0.77	1.18	0.25	0.94	0.74	0.50	0.96	0.74	0.50	0.96	0.81	0.47	0.96
Allisonella	-0.26	0.79	0.97	0.51	0.60	0.98	0.81	0.44	0.96	0.81	0.44	0.96	0.80	0.44	0.96
Anaerofilum	0.65	0.62	0.91	0.06	0.96	0.98	0.13	0.93	1.00	0.12	0.93	0.99	0.12	0.93	1.00
Anaerostipes	1.66	0.28	0.90	1.01	0.49	0.97	2.15	0.17	0.96	2.14	0.17	0.96	2.13	0.18	0.96
Anaerotruncus	-0.76	0.53	0.90	-1.29	0.27	0.94	-0.54	0.66	0.97	-0.52	0.68	0.97	-0.52	0.68	0.97
Bacteroides	1.91	0.30	0.90	1.84	0.30	0.94	2.25	0.22	0.96	2.24	0.22	0.96	2.31	0.21	0.96
Barnesiella	1.11	0.05	0.64	1.17	0.03	0.94	0.73	0.20	0.96	0.73	0.20	0.96	0.73	0.20	0.96
Bifidobacterium	0.03	0.97	0.99	0.38	0.58	0.98	0.19	0.79	1.00	0.18	0.80	0.99	0.16	0.82	1.00
Bilophila	0.99	0.22	0.90	0.49	0.53	0.98	0.81	0.33	0.96	0.81	0.34	0.96	0.84	0.32	0.96
Blautia	0.93	0.72	0.95	-0.55	0.83	0.98	0.11	0.97	1.00	0.11	0.97	0.99	0.12	0.96	1.00
Butyricicoccus	1.46	0.47	0.90	-0.12	0.95	0.98	-0.23	0.91	1.00	-0.23	0.91	0.99	-0.29	0.89	1.00
Butyricimonas	0.38	0.57	0.90	0.15	0.81	0.98	0.17	0.79	1.00	0.17	0.80	0.99	0.20	0.77	1.00
CAG.56	0.18	0.80	0.97	-0.13	0.85	0.98	-0.10	0.89	1.00	-0.10	0.89	0.99	-0.06	0.93	1.00
Candidatus_Soleaferrea	0.56	0.69	0.94	-0.16	0.91	0.98	0.01	1.00	1.00	0.01	0.99	0.99	0.00	1.00	1.00
Caproiciproducens	1.45	0.27	0.90	1.20	0.34	0.94	1.48	0.27	0.96	1.48	0.27	0.96	1.46	0.29	0.96
Catenibacterium	-0.82	0.23	0.90	-0.87	0.18	0.94	-0.70	0.31	0.96	-0.70	0.32	0.96	-0.75	0.29	0.96
Christensenellaceae_R.7_group	0.33	0.60	0.90	-0.03	0.96	0.98	-0.38	0.56	0.96	-0.38	0.56	0.96	-0.38	0.56	0.96
Clostridium_sensu_stricto_1	0.55	0.46	0.90	0.82	0.25	0.94	0.89	0.25	0.96	0.90	0.25	0.96	0.88	0.27	0.96
Colidextribacter	-0.25	0.87	0.98	-1.09	0.45	0.96	-1.16	0.44	0.96	-1.17	0.44	0.96	-1.25	0.42	0.96
Collinsella	0.56	0.44	0.90	0.79	0.26	0.94	0.96	0.20	0.96	0.97	0.20	0.96	0.97	0.20	0.96
Coprobacter	1.78	0.02	0.64	1.56	0.03	0.94	1.35	0.07	0.96	1.36	0.06	0.96	1.41	0.06	0.96
Coprococcus	2.10	0.02	0.64	1.47	0.09	0.94	1.56	0.09	0.96	1.59	0.08	0.96	1.61	0.08	0.96
Defluviitaleaceae_UCG.011	0.54	0.66	0.93	0.52	0.66	0.98	0.90	0.47	0.96	0.90	0.47	0.96	0.90	0.48	0.96
Desulfovibrio	-0.37	0.57	0.90	-0.38	0.55	0.98	-0.45	0.51	0.96	-0.45	0.52	0.96	-0.43	0.54	0.96
Dialister	-0.16	0.76	0.95	-0.14	0.77	0.98	0.08	0.88	1.00	0.08	0.89	0.99	0.03	0.95	1.00
Dorea	0.74	0.52	0.90	0.30	0.79	0.98	0.66	0.57	0.96	0.68	0.56	0.96	0.74	0.53	0.96
DTU089	1.48	0.18	0.90	1.42	0.18	0.94	1.32	0.24	0.96	1.38	0.22	0.96	1.38	0.22	0.96
Eggerthella	0.43	0.68	0.94	0.16	0.88	0.98	0.72	0.51	0.96	0.73	0.50	0.96	0.73	0.51	0.96
Eisenbergiella	-1.59	0.19	0.90	-1.53	0.19	0.94	-1.01	0.42	0.96	-1.00	0.43	0.96	-1.03	0.41	0.96
Enterorhabdus	0.67	0.51	0.90	0.29	0.76	0.98	0.76	0.47	0.96	0.77	0.47	0.96	0.76	0.48	0.96
Erysipelatoclostridium	-0.24	0.83	0.98	0.02	0.98	0.98	-0.04	0.97	1.00	-0.02	0.98	0.99	-0.05	0.97	1.00
Erysipelotrichaceae_UCG.003	0.52	0.52	0.90	0.38	0.63	0.98	-0.02	0.98	1.00	-0.02	0.98	0.99	-0.02	0.98	1.00
Escherichia.Shigella	0.14	0.86	0.98	0.66	0.39	0.94	0.87	0.29	0.96	0.86	0.30	0.96	0.88	0.29	0.96
Faecalibacterium	0.72	0.57	0.90	0.88	0.47	0.96	0.40	0.77	1.00	0.41	0.76	0.99	0.40	0.77	1.00
Family_XIII_AD3011_group	1.19	0.25	0.90	0.19	0.85	0.98	0.06	0.96	1.00	0.09	0.94	0.99	0.09	0.93	1.00
Family_XIII_UCG.001	0.30	0.80	0.97	-0.13	0.91	0.98	-0.13	0.92	1.00	-0.10	0.94	0.99	-0.07	0.95	1.00
FD2005	-2.05	0.05	0.64	-1.89	0.06	0.94	-2.11	0.05	0.96	-2.17	0.05	0.96	-2.19	0.05	0.96
Flavonifractor	0.71	0.47	0.90	0.85	0.37	0.94	1.17	0.25	0.96	1.17	0.25	0.96	1.15	0.26	0.96
Fournierella	0.07	0.95	0.99	-0.03	0.98	0.98	-0.71	0.50	0.96	-0.70	0.51	0.96	-0.70	0.51	0.96
Frisingicoccus	-0.03	0.98	0.99	0.22	0.81	0.98	0.49	0.61	0.96	0.48	0.62	0.96	0.51	0.60	0.96
Fusicatenibacter	0.02	0.98	0.99	-0.17	0.85	0.98	0.06	0.95	1.00	0.05	0.96	0.99	0.05	0.96	1.00
Fusobacterium	-0.07	0.95	0.99	0.50	0.66	0.98	0.64	0.59	0.96	0.67	0.58	0.96	0.76	0.54	0.96
GCA.900066575	1.21	0.27	0.90	0.76	0.48	0.96	0.55	0.62	0.96	0.57	0.61	0.96	0.57	0.62	0.96
Gordonibacter	0.65	0.60	0.90	0.28	0.81	0.98	0.64	0.61	0.96	0.67	0.60	0.96	0.68	0.59	0.96
Granulicatella	-1.57	0.33	0.90	-1.02	0.51	0.98	-0.92	0.59	0.96	-0.91	0.59	0.96	-0.98	0.57	0.96
Haemophilus	0.30	0.70	0.94	0.29	0.70	0.98	-0.31	0.70	0.98	-0.31	0.71	0.99	-0.35	0.68	0.97
Harryflintia	-0.44	0.74	0.95	-1.00	0.43	0.96	-0.76	0.58	0.96	-0.76	0.58	0.96	-0.72	0.61	0.96
Holdemanella	0.18	0.81	0.97	0.15	0.83	0.98	0.57	0.46	0.96	0.57	0.46	0.96	0.61	0.44	0.96

© 2022 Meyer K et al. *JAMA Network Open.*

Holdemania	-2.55	0.23	0.90	-2.29	0.26	0.94	-1.41	0.52	0.96	-1.38	0.53	0.96	-1.36	0.54	0.96
Howardella	-0.95	0.30	0.90	-1.12	0.20	0.94	-0.97	0.30	0.96	-0.96	0.31	0.96	-0.98	0.31	0.96
Hungatella	-2.15	0.05	0.64	-1.95	0.07	0.94	-2.10	0.06	0.96	-2.10	0.06	0.96	-2.15	0.06	0.96
Hydrogenoanaerobacterium	1.97	0.13	0.89	1.13	0.37	0.94	0.29	0.83	1.00	0.30	0.82	0.99	0.28	0.84	1.00
Incertae_Sedis	2.81	0.13	0.89	2.95	0.10	0.94	3.39	0.08	0.96	3.38	0.08	0.96	3.41	0.08	0.96
Intestinibacter	0.70	0.47	0.90	1.06	0.26	0.94	0.86	0.39	0.96	0.85	0.40	0.96	0.82	0.42	0.96
Intestinimonas	0.78	0.42	0.90	-0.19	0.85	0.98	-0.41	0.69	0.98	-0.41	0.69	0.97	-0.38	0.71	0.99
Lachnoclostridium	-1.73	0.40	0.90	-1.26	0.52	0.98	0.23	0.91	1.00	0.20	0.92	0.99	0.24	0.91	1.00
Lachnospira	1.74	0.09	0.77	0.93	0.36	0.94	0.23	0.83	1.00	0.21	0.85	0.99	0.20	0.85	1.00
Lachnospiraceae_FCS020_group	0.51	0.60	0.90	-0.04	0.97	0.98	-0.18	0.86	1.00	-0.17	0.87	0.99	-0.18	0.87	1.00
Lachnospiraceae_ND3007_group	1.17	0.09	0.77	0.78	0.24	0.94	0.69	0.34	0.96	0.68	0.34	0.96	0.70	0.34	0.96
Lachnospiraceae_NK4A136_group	0.70	0.48	0.90	0.03	0.98	0.98	-0.71	0.48	0.96	-0.70	0.49	0.96	-0.70	0.49	0.96
Lachnospiraceae_UCG.001	-0.03	0.96	0.99	-0.29	0.67	0.98	-0.43	0.56	0.96	-0.43	0.57	0.96	-0.43	0.56	0.96
Lachnospiraceae_UCG.004	0.12	0.90	0.99	-0.40	0.64	0.98	-0.63	0.49	0.96	-0.63	0.49	0.96	-0.66	0.47	0.96
Lachnospiraceae_UCG.010	-0.86	0.46	0.90	-1.40	0.21	0.94	-1.27	0.28	0.96	-1.26	0.28	0.96	-1.22	0.31	0.96
Lactobacillus	-2.31	0.03	0.64	-1.73	0.09	0.94	-2.26	0.04	0.96	-2.26	0.04	0.96	-2.42	0.03	0.96
Lactococcus	2.00	0.11	0.87	1.52	0.21	0.94	1.63	0.20	0.96	1.66	0.20	0.96	1.69	0.19	0.96
Marvinbryantia	0.66	0.40	0.90	0.25	0.74	0.98	0.15	0.85	1.00	0.16	0.85	0.99	0.16	0.84	1.00
Megasphaera	-0.31	0.61	0.90	0.07	0.91	0.98	0.41	0.53	0.96	0.41	0.53	0.96	0.39	0.56	0.96
Monoglobus	1.14	0.24	0.90	0.82	0.38	0.94	0.55	0.59	0.96	0.56	0.58	0.96	0.65	0.53	0.96
Moryella	0.75	0.56	0.90	0.27	0.83	0.98	-0.20	0.88	1.00	-0.18	0.89	0.99	-0.21	0.88	1.00
Negativibacillus	-1.22	0.16	0.90	-1.59	0.06	0.94	-1.94	0.03	0.96	-1.94	0.03	0.96	-1.94	0.03	0.96
NK4A214_group	0.41	0.55	0.90	-0.07	0.92	0.98	-0.32	0.65	0.97	-0.31	0.66	0.97	-0.33	0.64	0.96
Odoribacter	0.55	0.41	0.90	0.48	0.45	0.96	-0.06	0.93	1.00	-0.06	0.93	0.99	-0.03	0.96	1.00
Oscillibacter	0.92	0.42	0.90	0.62	0.57	0.98	0.68	0.57	0.96	0.70	0.56	0.96	0.68	0.57	0.96
Oscillospira	3.26	0.04	0.64	3.09	0.04	0.94	2.97	0.06	0.96	3.00	0.06	0.96	3.02	0.06	0.96
Oxalobacter	2.65	0.02	0.64	1.88	0.08	0.94	1.67	0.14	0.96	1.67	0.14	0.96	1.79	0.12	0.96
Paludicola	1.94	0.09	0.77	0.80	0.48	0.96	0.83	0.50	0.96	0.90	0.47	0.96	0.89	0.48	0.96
Parabacteroides	0.34	0.67	0.93	0.70	0.36	0.94	0.76	0.34	0.96	0.79	0.33	0.96	0.82	0.31	0.96
Paraprevotella	-0.36	0.51	0.90	-0.24	0.65	0.98	-0.42	0.47	0.96	-0.41	0.48	0.96	-0.43	0.47	0.96
Parasutterella	0.60	0.33	0.90	0.59	0.31	0.94	0.51	0.42	0.96	0.51	0.43	0.96	0.55	0.40	0.96
Peptococcus	-1.12	0.29	0.90	-1.75	0.09	0.94	-1.98	0.07	0.96	-1.98	0.07	0.96	-2.00	0.07	0.96
Phascolarctobacterium	0.61	0.28	0.90	0.63	0.25	0.94	0.64	0.27	0.96	0.66	0.26	0.96	0.73	0.23	0.96
Phocea	0.07	0.96	0.99	-0.43	0.76	0.98	-0.96	0.52	0.96	-0.96	0.52	0.96	-0.92	0.53	0.96
Prevotella	-0.58	0.22	0.90	-0.46	0.32	0.94	-0.52	0.28	0.96	-0.52	0.29	0.96	-0.52	0.29	0.96
Romboutsia	0.48	0.59	0.90	0.72	0.40	0.94	0.72	0.43	0.96	0.72	0.43	0.96	0.68	0.46	0.96
Roseburia	1.56	0.18	0.90	0.93	0.41	0.94	0.04	0.97	1.00	0.03	0.98	0.99	0.02	0.99	1.00
Ruminococcus	-0.58	0.41	0.90	-0.83	0.22	0.94	-1.02	0.16	0.96	-1.02	0.16	0.96	-1.04	0.15	0.96
Sellimonas	0.34	0.72	0.95	0.48	0.60	0.98	0.07	0.94	1.00	0.08	0.93	0.99	0.09	0.92	1.00
Senegalimassilia	0.09	0.91	0.99	0.03	0.98	0.98	-0.04	0.97	1.00	-0.02	0.98	0.99	-0.02	0.99	1.00
Shuttleworthia	0.53	0.65	0.93	0.14	0.90	0.98	0.26	0.82	1.00	0.26	0.83	0.99	0.28	0.81	1.00
Slackia	-1.08	0.16	0.90	-0.91	0.22	0.94	-0.85	0.29	0.96	-0.84	0.29	0.96	-0.84	0.30	0.96
Streptococcus	-0.13	0.92	0.99	-0.08	0.94	0.98	0.00	1.00	1.00	0.04	0.98	0.99	-0.05	0.97	1.00
Subdoligranulum	0.18	0.85	0.98	-0.16	0.85	0.98	-0.44	0.64	0.97	-0.43	0.65	0.97	-0.44	0.64	0.96
Sutterella	-0.11	0.84	0.98	0.11	0.83	0.98	0.21	0.69	0.98	0.22	0.68	0.97	0.22	0.69	0.97
TM7x	-1.51	0.23	0.90	-1.91	0.11	0.94	-1.38	0.28	0.96	-1.37	0.28	0.96	-1.40	0.27	0.96
Turicibacter	1.10	0.34	0.90	1.79	0.11	0.94	1.76	0.13	0.96	1.76	0.14	0.96	1.73	0.15	0.96
Tuzzerella	0.13	0.88	0.98	0.20	0.81	0.98	-0.32	0.73	1.00	-0.33	0.73	0.99	-0.32	0.73	1.00
UBA1819	-0.02	0.99	0.99	-0.32	0.81	0.98	0.30	0.83	1.00	0.33	0.82	0.99	0.32	0.82	1.00
UCG.002	-0.22	0.75	0.95	-0.71	0.29	0.94	-1.05	0.14	0.96	-1.04	0.15	0.96	-1.04	0.15	0.96
UCG.003	0.61	0.39	0.90	0.17	0.80	0.98	-0.14	0.85	1.00	-0.13	0.85	0.99	-0.13	0.87	1.00
UCG.005	0.99	0.21	0.90	0.66	0.39	0.94	0.52	0.54	0.96	0.55	0.52	0.96	0.55	0.52	0.96
UCG.009	0.39	0.74	0.95	-0.13	0.91	0.98	-0.54	0.65	0.97	-0.54	0.66	0.97	-0.59	0.63	0.96
Veillonella	0.45	0.57	0.90	0.64	0.41	0.94	0.12	0.89	1.00	0.12	0.89	0.99	0.09	0.91	1.00
Victivallis	0.12	0.87	0.98	-0.12	0.87	0.98	-0.41	0.60	0.96	-0.41	0.60	0.96	-0.42	0.60	0.96

Multivariable-adjusted regression models adjusted for:
Model 1: sequencing batch, age, race, gender, study center
Model 2: Model 1, with education

© 2022 Meyer K et al. *JAMA Network Open.*

Model 3: Model 2, with physical activity, diet score, medication use, and smoking
Model 4: Model 3 with BMI
Model 5: Model 4 with hypertension and diabetes

© 2022 Meyer K et al. *JAMA Network Open.*

eTable 52: Multivariable-adjusted regression coefficients, p-values, and false discovery rate (FDR)-adjusted p-values for genera among male participants with respect to MOCA.

	Model1			Model2			Model3			Model4			Model5		
	beta	p-value	FDR	beta	p-value	FDR	beta	p-value	FDR	beta	p-value	FDR	beta	p-value	FDR
Acetanaerobacterium	-0.48	0.33	0.64	-0.54	0.24	0.63	-0.85	0.07	0.37	-0.85	0.07	0.36	-0.84	0.07	0.40
Acidaminococcus	-0.27	0.06	0.29	-0.17	0.22	0.62	-0.24	0.09	0.43	-0.26	0.08	0.36	-0.28	0.06	0.35
Actinomyces	-0.25	0.54	0.83	-0.22	0.57	0.85	-0.24	0.56	0.86	-0.19	0.64	0.88	-0.25	0.54	0.83
Adlercreutzia	0.00	0.99	1.00	0.04	0.87	0.97	-0.03	0.90	1.00	-0.04	0.87	0.99	-0.02	0.93	1.00
Agathobacter	-0.11	0.75	0.90	-0.28	0.38	0.72	-0.23	0.47	0.83	-0.28	0.38	0.75	-0.28	0.38	0.77
Akkermansia	0.06	0.73	0.89	-0.09	0.57	0.85	-0.17	0.29	0.71	-0.18	0.27	0.68	-0.18	0.29	0.67
Alistipes	0.37	0.20	0.51	0.16	0.56	0.85	-0.03	0.90	1.00	-0.04	0.88	0.99	-0.01	0.98	1.00
Allisonella	0.12	0.65	0.89	0.38	0.14	0.54	0.29	0.28	0.71	0.29	0.29	0.69	0.27	0.31	0.67
Anaerofilum	-0.16	0.66	0.89	-0.36	0.29	0.63	-0.44	0.20	0.56	-0.45	0.20	0.57	-0.46	0.19	0.58
Anaerostipes	0.40	0.31	0.64	0.18	0.63	0.86	0.31	0.42	0.79	0.29	0.45	0.81	0.24	0.53	0.83
Anaerotruncus	-0.32	0.32	0.64	-0.49	0.11	0.46	-0.41	0.19	0.56	-0.38	0.23	0.59	-0.38	0.23	0.58
Bacteroides	-0.04	0.93	1.00	-0.07	0.89	0.97	-0.01	0.99	1.00	-0.05	0.92	0.99	0.01	0.99	1.00
Barnesiella	0.18	0.23	0.55	0.20	0.16	0.54	0.19	0.19	0.56	0.19	0.18	0.57	0.20	0.18	0.58
Bifidobacterium	-0.11	0.57	0.85	0.01	0.96	0.99	0.00	0.98	1.00	-0.01	0.96	0.99	-0.02	0.90	1.00
Bilophila	0.05	0.81	0.96	-0.11	0.58	0.85	-0.31	0.14	0.52	-0.35	0.11	0.45	-0.32	0.14	0.54
Blautia	-0.28	0.69	0.89	-0.78	0.24	0.63	-0.61	0.37	0.79	-0.60	0.38	0.75	-0.58	0.39	0.77
Butyricicoccus	0.56	0.30	0.63	0.03	0.95	0.99	-0.05	0.93	1.00	-0.04	0.94	0.99	-0.12	0.82	1.00
Butyricimonas	-0.09	0.61	0.87	-0.17	0.31	0.67	-0.15	0.40	0.79	-0.15	0.39	0.76	-0.13	0.47	0.81
CAG.56	0.30	0.12	0.39	0.20	0.28	0.63	0.16	0.39	0.79	0.16	0.38	0.75	0.19	0.31	0.67
Candidatus_Soleaferrea	-0.72	0.06	0.29	-0.97	0.01	0.16	-1.11	0.00	0.12	-1.10	0.00	0.11	-1.12	0.00	0.19
Caproiciproducens	-0.58	0.11	0.39	-0.66	0.05	0.33	-0.73	0.04	0.30	-0.73	0.03	0.29	-0.79	0.02	0.26
Catenibacterium	-0.08	0.66	0.89	-0.10	0.56	0.85	-0.02	0.93	1.00	-0.01	0.95	0.99	-0.05	0.79	1.00
Christensenellaceae_R.7_group	0.26	0.13	0.41	0.14	0.39	0.73	0.11	0.51	0.83	0.12	0.47	0.81	0.12	0.49	0.83
Clostridium_sensu_stricto_1	0.12	0.57	0.85	0.20	0.29	0.63	0.36	0.07	0.37	0.36	0.07	0.36	0.33	0.10	0.44
Colidextribacter	-0.15	0.71	0.89	-0.42	0.27	0.63	-0.36	0.36	0.79	-0.36	0.35	0.75	-0.42	0.28	0.67
Collinsella	0.00	0.99	1.00	0.07	0.70	0.87	0.00	0.98	1.00	0.00	0.99	0.99	-0.01	0.97	1.00
Coprobacter	0.14	0.47	0.76	0.07	0.70	0.87	-0.02	0.93	1.00	-0.01	0.97	0.99	0.02	0.90	1.00
Coprococcus	0.61	0.01	0.21	0.40	0.08	0.43	0.47	0.04	0.32	0.50	0.04	0.29	0.49	0.04	0.33
Defluviitaleaceae_UCG.011	0.00	1.00	1.00	0.00	1.00	1.00	-0.05	0.88	1.00	-0.04	0.89	0.99	-0.05	0.89	1.00
Desulfovibrio	-0.16	0.38	0.66	-0.16	0.34	0.68	-0.13	0.44	0.81	-0.11	0.55	0.86	-0.09	0.62	0.89
Dialister	0.06	0.69	0.89	0.06	0.64	0.86	0.06	0.68	0.91	0.05	0.72	0.94	0.01	0.93	1.00
Dorea	0.25	0.42	0.71	0.09	0.74	0.87	0.15	0.60	0.89	0.17	0.57	0.86	0.18	0.53	0.83
DTU089	-0.04	0.89	1.00	-0.06	0.84	0.94	-0.15	0.60	0.89	-0.11	0.69	0.92	-0.12	0.69	0.92
Eggerthella	-0.28	0.32	0.64	-0.37	0.16	0.54	-0.24	0.40	0.79	-0.22	0.42	0.78	-0.22	0.42	0.81
Eisenbergiella	-0.59	0.07	0.33	-0.56	0.07	0.38	-0.67	0.03	0.30	-0.65	0.04	0.31	-0.65	0.04	0.33
Enterorhabdus	-0.18	0.50	0.79	-0.31	0.22	0.62	-0.25	0.36	0.79	-0.24	0.38	0.75	-0.28	0.32	0.67
Erysipelatoclostridium	-0.40	0.20	0.51	-0.31	0.29	0.63	-0.21	0.49	0.83	-0.18	0.55	0.86	-0.19	0.52	0.83
Erysipelotrichaceae_UCG.003	0.17	0.44	0.72	0.12	0.57	0.85	0.01	0.98	1.00	0.00	0.99	0.99	-0.01	0.96	1.00
Escherichia.Shigella	-0.24	0.26	0.57	-0.07	0.73	0.87	-0.22	0.30	0.71	-0.24	0.27	0.68	-0.22	0.31	0.67
Faecalibacterium	0.49	0.14	0.42	0.53	0.09	0.44	0.71	0.03	0.30	0.72	0.03	0.29	0.68	0.04	0.35
Family_XIII_AD3011_group	0.50	0.07	0.33	0.18	0.50	0.83	0.18	0.53	0.83	0.22	0.44	0.79	0.22	0.44	0.81
Family_XIII_UCG.001	0.13	0.68	0.89	-0.02	0.96	0.99	0.00	1.00	1.00	0.05	0.89	0.99	0.05	0.89	1.00
FD2005	-0.26	0.36	0.66	-0.21	0.44	0.76	-0.15	0.58	0.88	-0.19	0.50	0.82	-0.21	0.45	0.81
Flavonifractor	-0.42	0.11	0.39	-0.38	0.13	0.53	-0.34	0.20	0.56	-0.34	0.19	0.57	-0.37	0.16	0.57
Fournierella	0.32	0.25	0.57	0.28	0.27	0.63	0.33	0.23	0.60	0.35	0.20	0.57	0.34	0.21	0.58
Frisingicoccus	-0.01	0.96	1.00	0.07	0.75	0.87	-0.03	0.92	1.00	-0.04	0.88	0.99	0.00	1.00	1.00
Fusicatenibacter	0.00	0.99	1.00	-0.06	0.79	0.90	-0.11	0.66	0.91	-0.12	0.62	0.88	-0.12	0.61	0.89
Fusobacterium	0.07	0.84	0.98	0.26	0.40	0.73	0.29	0.35	0.78	0.32	0.30	0.69	0.40	0.21	0.58
GCA.900066575	0.42	0.16	0.43	0.26	0.35	0.68	0.19	0.50	0.83	0.21	0.46	0.81	0.18	0.54	0.83
Gordonibacter	-0.62	0.06	0.29	-0.73	0.02	0.17	-0.58	0.06	0.37	-0.56	0.07	0.36	-0.55	0.08	0.41
Granulicatella	-0.53	0.22	0.53	-0.34	0.40	0.73	-0.54	0.21	0.56	-0.53	0.22	0.58	-0.54	0.21	0.58
Haemophilus	0.48	0.02	0.22	0.48	0.01	0.17	0.41	0.05	0.36	0.42	0.05	0.33	0.38	0.07	0.40
Harryflintia	-0.71	0.04	0.29	-0.88	0.01	0.16	-1.00	0.00	0.12	-1.00	0.00	0.11	-0.96	0.01	0.19
Holdemanella	0.29	0.16	0.43	0.28	0.15	0.54	0.37	0.07	0.37	0.37	0.07	0.36	0.39	0.05	0.35

© 2022 Meyer K et al. *JAMA Network Open.*

Holdemania	-1.56	0.01	0.21	-1.46	0.01	0.16	-1.40	0.01	0.19	-1.36	0.01	0.20	-1.32	0.02	0.20
Howardella	-0.03	0.90	1.00	-0.09	0.70	0.87	-0.01	0.96	1.00	0.01	0.97	0.99	0.00	0.99	1.00
Hungatella	-0.80	0.01	0.21	-0.74	0.01	0.16	-0.70	0.02	0.20	-0.68	0.02	0.23	-0.72	0.01	0.19
Hydrogenoanaerobacterium	0.51	0.15	0.43	0.23	0.50	0.83	0.07	0.84	1.00	0.09	0.80	0.99	0.05	0.89	1.00
Incertae_Sedis	-0.99	0.05	0.29	-0.93	0.05	0.33	-0.86	0.08	0.39	-0.87	0.08	0.36	-0.83	0.09	0.43
Intestinibacter	0.61	0.02	0.22	0.73	0.00	0.16	0.77	0.00	0.12	0.75	0.00	0.11	0.71	0.01	0.19
Intestinimonas	0.00	0.99	1.00	-0.33	0.20	0.61	-0.52	0.04	0.32	-0.53	0.04	0.31	-0.51	0.05	0.35
Lachnoclostridium	-1.47	0.01	0.21	-1.33	0.01	0.16	-1.31	0.01	0.20	-1.35	0.01	0.20	-1.33	0.01	0.19
Lachnospira	1.00	0.00	0.03	0.74	0.01	0.16	0.43	0.12	0.48	0.41	0.14	0.53	0.39	0.16	0.57
Lachnospiraceae_FCS020_group	0.53	0.05	0.29	0.35	0.16	0.54	0.42	0.11	0.47	0.43	0.11	0.45	0.42	0.12	0.48
Lachnospiraceae_ND3007_group	0.42	0.02	0.22	0.29	0.10	0.44	0.34	0.06	0.37	0.34	0.06	0.36	0.34	0.07	0.40
Lachnospiraceae_NK4A136_group	0.46	0.08	0.33	0.24	0.33	0.68	0.11	0.67	0.91	0.12	0.64	0.88	0.12	0.65	0.90
Lachnospiraceae_UCG.001	0.23	0.23	0.56	0.14	0.43	0.75	0.08	0.69	0.92	0.08	0.68	0.92	0.07	0.73	0.96
Lachnospiraceae_UCG.004	0.45	0.06	0.29	0.28	0.21	0.62	0.31	0.18	0.56	0.30	0.19	0.57	0.29	0.22	0.58
Lachnospiraceae_UCG.010	0.05	0.87	1.00	-0.13	0.67	0.87	-0.09	0.77	0.99	-0.06	0.85	0.99	0.00	1.00	1.00
Lactobacillus	-0.49	0.08	0.33	-0.29	0.28	0.63	-0.40	0.15	0.52	-0.40	0.15	0.53	-0.46	0.11	0.44
Lactococcus	0.28	0.42	0.71	0.11	0.73	0.87	0.21	0.53	0.83	0.24	0.47	0.81	0.26	0.43	0.81
Marvinbryantia	0.20	0.34	0.64	0.06	0.75	0.87	0.27	0.19	0.56	0.28	0.17	0.56	0.28	0.18	0.58
Megasphaera	-0.42	0.01	0.21	-0.30	0.05	0.33	-0.26	0.12	0.48	-0.26	0.12	0.47	-0.28	0.10	0.44
Monoglobus	0.44	0.09	0.35	0.34	0.17	0.55	0.23	0.38	0.79	0.24	0.36	0.75	0.33	0.22	0.58
Moryella	0.70	0.04	0.29	0.54	0.10	0.44	0.47	0.17	0.56	0.50	0.15	0.53	0.47	0.17	0.58
Negativibacillus	-0.42	0.08	0.33	-0.54	0.02	0.17	-0.66	0.00	0.12	-0.67	0.00	0.11	-0.66	0.00	0.19
NK4A214_group	0.21	0.26	0.57	0.05	0.76	0.87	-0.03	0.88	1.00	-0.02	0.92	0.99	-0.04	0.83	1.00
Odoribacter	0.08	0.66	0.89	0.06	0.72	0.87	0.02	0.92	1.00	0.01	0.94	0.99	0.04	0.82	1.00
Oscillibacter	-0.18	0.57	0.85	-0.27	0.35	0.68	-0.33	0.29	0.71	-0.31	0.32	0.73	-0.33	0.28	0.67
Oscillospira	0.04	0.93	1.00	-0.01	0.98	0.99	-0.09	0.83	1.00	-0.06	0.88	0.99	-0.05	0.91	1.00
Oxalobacter	0.13	0.66	0.89	-0.13	0.64	0.86	-0.07	0.82	1.00	-0.06	0.84	0.99	0.01	0.98	1.00
Paludicola	0.47	0.12	0.39	0.10	0.74	0.87	-0.01	0.96	1.00	0.04	0.90	0.99	0.02	0.96	1.00
Parabacteroides	-0.02	0.91	1.00	0.10	0.64	0.86	0.09	0.67	0.91	0.11	0.58	0.86	0.14	0.51	0.83
Paraprevotella	-0.14	0.36	0.66	-0.10	0.49	0.83	-0.10	0.51	0.83	-0.08	0.58	0.86	-0.09	0.56	0.84
Parasutterella	0.35	0.03	0.29	0.35	0.02	0.22	0.27	0.10	0.44	0.26	0.11	0.45	0.29	0.08	0.40
Peptococcus	0.04	0.89	1.00	-0.16	0.55	0.85	-0.16	0.56	0.86	-0.14	0.63	0.88	-0.16	0.58	0.86
Phascolarctobacterium	0.05	0.72	0.89	0.06	0.69	0.87	0.07	0.62	0.90	0.09	0.55	0.86	0.12	0.44	0.81
Phocea	-0.35	0.37	0.66	-0.53	0.16	0.54	-0.81	0.03	0.30	-0.82	0.03	0.29	-0.79	0.04	0.33
Prevotella	-0.13	0.30	0.63	-0.10	0.43	0.75	-0.10	0.42	0.79	-0.10	0.41	0.78	-0.11	0.40	0.77
Romboutsia	-0.08	0.73	0.89	0.00	0.98	0.99	0.05	0.82	1.00	0.05	0.83	0.99	0.00	0.99	1.00
Roseburia	0.77	0.01	0.21	0.56	0.06	0.34	0.22	0.48	0.83	0.21	0.50	0.82	0.19	0.55	0.84
Ruminococcus	0.25	0.18	0.49	0.17	0.35	0.68	0.08	0.66	0.91	0.09	0.64	0.88	0.07	0.72	0.96
Sellimonas	0.43	0.09	0.35	0.47	0.05	0.33	0.58	0.02	0.20	0.60	0.01	0.20	0.61	0.01	0.19
Senegalimassilia	-0.09	0.69	0.89	-0.12	0.59	0.86	-0.04	0.86	1.00	-0.02	0.92	0.99	-0.02	0.94	1.00
Shuttleworthia	0.11	0.73	0.89	-0.02	0.93	0.99	-0.23	0.45	0.81	-0.25	0.42	0.78	-0.22	0.47	0.81
Slackia	-0.03	0.90	1.00	0.03	0.90	0.98	0.08	0.70	0.92	0.10	0.63	0.88	0.10	0.63	0.89
Streptococcus	0.31	0.34	0.64	0.33	0.28	0.63	0.34	0.30	0.71	0.41	0.22	0.58	0.35	0.29	0.67
Subdoligranulum	0.37	0.14	0.42	0.26	0.27	0.63	0.24	0.33	0.76	0.25	0.30	0.69	0.24	0.32	0.68
Sutterella	-0.33	0.02	0.22	-0.26	0.05	0.33	-0.18	0.19	0.56	-0.17	0.21	0.58	-0.17	0.21	0.58
TM7x	0.01	0.98	1.00	-0.11	0.73	0.87	0.04	0.90	1.00	0.06	0.85	0.99	0.05	0.88	1.00
Turicibacter	0.27	0.39	0.68	0.50	0.09	0.44	0.44	0.14	0.52	0.42	0.17	0.56	0.38	0.22	0.58
Tuzzerella	-0.13	0.59	0.86	-0.11	0.64	0.86	-0.15	0.52	0.83	-0.16	0.50	0.82	-0.16	0.50	0.83
UBA1819	-0.73	0.04	0.29	-0.82	0.02	0.17	-0.91	0.01	0.19	-0.89	0.01	0.20	-0.88	0.01	0.19
UCG.002	0.14	0.44	0.72	-0.01	0.93	0.99	0.02	0.90	1.00	0.04	0.84	0.99	0.05	0.77	1.00
UCG.003	0.37	0.05	0.29	0.22	0.22	0.62	0.15	0.41	0.79	0.16	0.38	0.75	0.16	0.39	0.77
UCG.005	0.24	0.28	0.60	0.12	0.55	0.85	0.09	0.68	0.91	0.12	0.58	0.86	0.11	0.63	0.89
UCG.009	0.20	0.53	0.82	0.04	0.91	0.98	-0.02	0.94	1.00	-0.01	0.98	0.99	-0.05	0.87	1.00
Veillonella	0.34	0.11	0.39	0.40	0.05	0.33	0.36	0.09	0.43	0.36	0.09	0.42	0.33	0.12	0.49
Victivallis	-0.02	0.93	1.00	-0.10	0.61	0.86	-0.08	0.71	0.92	-0.08	0.70	0.92	-0.08	0.68	0.92

Multivariable-adjusted regression models adjusted for:
Model 1: sequencing batch, age, race, gender, study center
Model 2: Model 1, with education

© 2022 Meyer K et al. *JAMA Network Open.*

Model 3: Model 2, with physical activity, diet score, medication use, and smoking
Model 4: Model 3 with BMI
Model 5: Model 4 with hypertension and diabetes

© 2022 Meyer K et al. *JAMA Network Open.*

eTable 53: Multivariable-adjusted regression coefficients, p-values, and false discovery rate (FDR)-adjusted p-values for genera among male participants with respect to PC (1st).

	Model1			Model2			Model3			Model4			Model5		
	beta	p-value	FDR	beta	p-value	FDR	beta	p-value	FDR	beta	p-value	FDR	beta	p-value	FDR
Acetanaerobacterium	-0.03	0.89	0.93	-0.06	0.76	0.87	-0.16	0.44	0.97	-0.16	0.43	0.97	-0.14	0.49	0.99
Acidaminococcus	-0.12	0.07	0.39	-0.06	0.32	0.75	-0.05	0.44	0.97	-0.06	0.38	0.95	-0.07	0.30	0.87
Actinomyces	-0.05	0.80	0.86	-0.03	0.86	0.89	0.02	0.92	0.97	0.04	0.81	0.97	0.00	0.99	0.99
Adlercreutzia	-0.05	0.67	0.84	-0.02	0.80	0.87	-0.03	0.75	0.97	-0.04	0.72	0.97	-0.03	0.79	0.99
Agathobacter	0.16	0.30	0.61	0.06	0.65	0.85	-0.02	0.90	0.97	-0.04	0.79	0.97	-0.05	0.73	0.99
Akkermansia	0.03	0.73	0.85	-0.06	0.41	0.75	-0.08	0.26	0.74	-0.09	0.24	0.70	-0.08	0.28	0.84
Alistipes	0.21	0.10	0.39	0.09	0.44	0.75	0.01	0.91	0.97	0.01	0.93	0.97	0.05	0.67	0.99
Allisonella	-0.01	0.92	0.95	0.13	0.25	0.67	0.10	0.41	0.97	0.10	0.42	0.97	0.09	0.44	0.96
Anaerofilum	0.03	0.85	0.90	-0.08	0.60	0.84	-0.10	0.51	0.97	-0.10	0.50	0.97	-0.09	0.56	0.99
Anaerostipes	0.14	0.42	0.67	0.02	0.90	0.92	0.07	0.69	0.97	0.06	0.73	0.97	0.05	0.79	0.99
Anaerotruncus	-0.13	0.38	0.66	-0.22	0.10	0.48	-0.19	0.16	0.69	-0.18	0.20	0.69	-0.16	0.24	0.75
Bacteroides	0.08	0.73	0.85	0.07	0.75	0.87	0.11	0.60	0.97	0.09	0.67	0.97	0.11	0.59	0.99
Barnesiella	0.16	0.02	0.27	0.17	0.01	0.19	0.14	0.03	0.54	0.14	0.03	0.53	0.13	0.04	0.53
Bifidobacterium	0.00	1.00	1.00	0.07	0.40	0.75	0.04	0.62	0.97	0.03	0.68	0.97	0.03	0.73	0.99
Bilophila	0.15	0.14	0.45	0.05	0.54	0.81	0.03	0.76	0.97	0.02	0.86	0.97	0.02	0.82	0.99
Blautia	0.18	0.57	0.76	-0.09	0.75	0.87	-0.06	0.84	0.97	-0.06	0.85	0.97	-0.05	0.87	0.99
Butyricicoccus	0.34	0.17	0.49	0.05	0.84	0.88	-0.02	0.95	0.97	-0.01	0.96	0.97	-0.01	0.98	0.99
Butyricimonas	0.03	0.76	0.85	-0.02	0.81	0.87	-0.01	0.86	0.97	-0.01	0.85	0.97	0.00	0.99	0.99
CAG.56	0.10	0.25	0.53	0.04	0.59	0.84	0.02	0.78	0.97	0.02	0.77	0.97	0.04	0.65	0.99
Candidatus_Soleaferrea	-0.12	0.48	0.69	-0.25	0.10	0.48	-0.31	0.06	0.54	-0.30	0.06	0.56	-0.29	0.07	0.53
Caproiciproducens	-0.20	0.22	0.51	-0.25	0.09	0.48	-0.25	0.10	0.59	-0.25	0.10	0.56	-0.26	0.09	0.53
Catenibacterium	-0.02	0.77	0.85	-0.03	0.65	0.85	-0.03	0.75	0.97	-0.02	0.77	0.97	-0.04	0.63	0.99
Christensenellaceae_R.7_group	0.13	0.10	0.39	0.06	0.40	0.75	0.01	0.85	0.97	0.02	0.81	0.97	0.03	0.73	0.99
Clostridium_sensu_stricto_1	0.07	0.47	0.69	0.11	0.16	0.60	0.14	0.12	0.62	0.14	0.11	0.60	0.11	0.21	0.71
Colidextribacter	-0.18	0.32	0.62	-0.34	0.04	0.35	-0.30	0.08	0.54	-0.30	0.08	0.56	-0.33	0.06	0.53
Collinsella	0.01	0.93	0.95	0.05	0.54	0.81	0.02	0.82	0.97	0.02	0.79	0.97	0.03	0.73	0.99
Coprobacter	0.11	0.20	0.51	0.08	0.36	0.75	0.02	0.77	0.97	0.03	0.73	0.97	0.05	0.56	0.99
Coprococcus	0.33	0.00	0.08	0.21	0.03	0.35	0.20	0.06	0.54	0.21	0.04	0.53	0.22	0.03	0.53
Defluviitaleaceae_UCG.011	0.14	0.35	0.64	0.14	0.30	0.75	0.16	0.25	0.74	0.17	0.24	0.70	0.18	0.21	0.71
Desulfovibrio	-0.06	0.48	0.69	-0.06	0.43	0.75	-0.07	0.38	0.97	-0.06	0.48	0.97	-0.04	0.60	0.99
Dialister	0.02	0.75	0.85	0.02	0.67	0.85	0.03	0.68	0.97	0.02	0.74	0.97	0.00	0.97	0.99
Dorea	0.17	0.22	0.51	0.09	0.49	0.79	0.09	0.50	0.97	0.09	0.47	0.97	0.11	0.41	0.94
DTU089	0.00	0.99	1.00	-0.01	0.96	0.96	-0.05	0.71	0.97	-0.03	0.81	0.97	-0.02	0.89	0.99
Eggerthella	-0.06	0.67	0.84	-0.11	0.36	0.75	-0.03	0.83	0.97	-0.02	0.87	0.97	-0.01	0.90	0.99
Eisenbergiella	-0.28	0.06	0.36	-0.26	0.05	0.35	-0.24	0.08	0.54	-0.23	0.10	0.56	-0.25	0.08	0.53
Enterorhabdus	0.04	0.72	0.85	-0.03	0.80	0.87	-0.02	0.89	0.97	-0.01	0.92	0.97	-0.01	0.93	0.99
Erysipelatoclostridium	-0.18	0.19	0.51	-0.14	0.29	0.75	-0.08	0.55	0.97	-0.07	0.62	0.97	-0.09	0.52	0.99
Erysipelotrichaceae_UCG.003	0.08	0.40	0.66	0.06	0.54	0.81	0.01	0.92	0.97	0.01	0.93	0.97	0.01	0.89	0.99
Escherichia.Shigella	-0.09	0.35	0.64	0.01	0.95	0.96	0.02	0.83	0.97	0.01	0.88	0.97	0.03	0.79	0.99
Faecalibacterium	0.24	0.12	0.41	0.26	0.06	0.37	0.30	0.05	0.54	0.30	0.04	0.53	0.30	0.05	0.53
Family_XIII_AD3011_group	0.24	0.05	0.34	0.06	0.59	0.84	0.02	0.85	0.97	0.04	0.73	0.97	0.06	0.61	0.99
Family_XIII_UCG.001	0.08	0.60	0.78	-0.01	0.96	0.96	-0.01	0.93	0.97	0.01	0.95	0.97	0.03	0.85	0.99
FD2005	-0.12	0.35	0.64	-0.09	0.43	0.75	-0.14	0.25	0.74	-0.16	0.19	0.69	-0.16	0.20	0.71
Flavonifractor	-0.10	0.40	0.66	-0.07	0.49	0.79	0.00	0.99	0.99	0.00	0.97	0.98	-0.02	0.86	0.99
Fournierella	0.09	0.47	0.69	0.07	0.53	0.81	0.02	0.89	0.97	0.03	0.83	0.97	0.04	0.74	0.99
Frisingicoccus	-0.14	0.21	0.51	-0.10	0.35	0.75	-0.11	0.30	0.83	-0.12	0.27	0.76	-0.10	0.34	0.88
Fusicatenibacter	0.06	0.57	0.76	0.03	0.78	0.87	-0.01	0.96	0.97	-0.01	0.91	0.97	-0.01	0.93	0.99
Fusobacterium	-0.06	0.68	0.84	0.04	0.74	0.87	0.09	0.53	0.97	0.10	0.47	0.97	0.13	0.36	0.90
GCA.900066575	0.16	0.24	0.53	0.07	0.55	0.81	0.03	0.83	0.97	0.03	0.78	0.97	0.04	0.73	0.99
Gordonibacter	-0.11	0.47	0.69	-0.17	0.22	0.63	-0.10	0.46	0.97	-0.09	0.52	0.97	-0.07	0.62	0.99
Granulicatella	-0.20	0.31	0.62	-0.09	0.61	0.84	-0.04	0.82	0.97	-0.04	0.84	0.97	-0.06	0.74	0.99
Haemophilus	0.20	0.03	0.27	0.20	0.02	0.26	0.16	0.08	0.54	0.17	0.07	0.56	0.16	0.08	0.53
Harryflintia	-0.20	0.20	0.51	-0.30	0.04	0.35	-0.33	0.03	0.54	-0.33	0.03	0.53	-0.30	0.05	0.53
Holdemanella	0.08	0.41	0.66	0.07	0.40	0.75	0.07	0.41	0.97	0.07	0.41	0.97	0.09	0.33	0.88

© 2022 Meyer K et al. *JAMA Network Open.*

Holdemania	-0.44	0.09	0.39	-0.38	0.10	0.48	-0.37	0.13	0.62	-0.35	0.16	0.68	-0.31	0.20	0.71
Howardella	-0.10	0.39	0.66	-0.13	0.21	0.63	-0.14	0.19	0.70	-0.13	0.22	0.70	-0.14	0.20	0.71
Hungatella	-0.44	0.00	0.05	-0.41	0.00	0.06	-0.38	0.00	0.18	-0.37	0.00	0.23	-0.40	0.00	0.16
Hydrogenoanaerobacterium	0.34	0.03	0.27	0.18	0.21	0.63	0.09	0.57	0.97	0.10	0.53	0.97	0.10	0.53	0.99
Incertae_Sedis	0.05	0.83	0.89	0.08	0.70	0.86	0.08	0.73	0.97	0.07	0.74	0.97	0.09	0.69	0.99
Intestinibacter	0.24	0.04	0.30	0.31	0.00	0.13	0.28	0.01	0.38	0.27	0.02	0.52	0.26	0.02	0.53
Intestinimonas	0.07	0.54	0.74	-0.11	0.33	0.75	-0.16	0.16	0.68	-0.17	0.15	0.68	-0.14	0.23	0.74
Lachnoclostridium	-0.55	0.03	0.27	-0.47	0.04	0.35	-0.29	0.21	0.70	-0.31	0.19	0.69	-0.33	0.16	0.71
Lachnospira	0.42	0.00	0.05	0.27	0.02	0.26	0.16	0.21	0.70	0.15	0.24	0.70	0.15	0.22	0.71
Lachnospiraceae_FCS020_group	0.27	0.03	0.27	0.17	0.13	0.56	0.15	0.22	0.70	0.15	0.21	0.70	0.15	0.20	0.71
Lachnospiraceae_ND3007_group	0.22	0.01	0.21	0.15	0.05	0.35	0.14	0.09	0.54	0.14	0.09	0.56	0.14	0.08	0.53
Lachnospiraceae_NK4A136_group	0.20	0.10	0.39	0.07	0.50	0.80	-0.06	0.62	0.97	-0.05	0.65	0.97	-0.04	0.75	0.99
Lachnospiraceae_UCG.001	0.15	0.08	0.39	0.10	0.18	0.63	0.07	0.42	0.97	0.07	0.41	0.97	0.07	0.43	0.96
Lachnospiraceae_UCG.004	0.18	0.09	0.39	0.09	0.38	0.75	0.06	0.58	0.97	0.05	0.59	0.97	0.03	0.74	0.99
Lachnospiraceae_UCG.010	-0.06	0.70	0.84	-0.16	0.22	0.64	-0.19	0.16	0.68	-0.18	0.19	0.69	-0.15	0.28	0.84
Lactobacillus	-0.28	0.03	0.27	-0.16	0.16	0.60	-0.22	0.07	0.54	-0.22	0.07	0.56	-0.28	0.03	0.53
Lactococcus	0.13	0.39	0.66	0.04	0.76	0.87	0.08	0.57	0.97	0.10	0.51	0.97	0.10	0.48	0.99
Marvinbryantia	0.12	0.22	0.51	0.04	0.65	0.85	0.04	0.65	0.97	0.05	0.61	0.97	0.05	0.56	0.99
Megasphaera	-0.13	0.08	0.39	-0.06	0.37	0.75	-0.02	0.75	0.97	-0.02	0.74	0.97	-0.04	0.63	0.99
Monoglobus	0.19	0.10	0.39	0.14	0.21	0.63	0.07	0.55	0.97	0.08	0.52	0.97	0.10	0.40	0.94
Moryella	0.30	0.05	0.34	0.22	0.13	0.56	0.10	0.51	0.97	0.12	0.45	0.97	0.11	0.46	0.99
Negativibacillus	-0.17	0.11	0.39	-0.24	0.01	0.26	-0.27	0.01	0.30	-0.28	0.01	0.28	-0.26	0.01	0.40
NK4A214_group	0.07	0.43	0.67	-0.02	0.78	0.87	-0.08	0.32	0.87	-0.08	0.35	0.90	-0.07	0.39	0.94
Odoribacter	0.10	0.23	0.52	0.09	0.23	0.65	0.05	0.56	0.97	0.04	0.58	0.97	0.06	0.42	0.94
Oscillibacter	0.08	0.55	0.75	0.03	0.82	0.87	-0.02	0.86	0.97	-0.01	0.92	0.97	-0.02	0.90	0.99
Oscillospira	0.10	0.60	0.78	0.07	0.68	0.85	0.08	0.67	0.97	0.09	0.62	0.97	0.11	0.55	0.99
Oxalobacter	0.32	0.02	0.27	0.18	0.15	0.59	0.16	0.21	0.70	0.17	0.20	0.69	0.22	0.10	0.53
Paludicola	0.24	0.08	0.39	0.03	0.82	0.87	-0.04	0.78	0.97	-0.01	0.93	0.97	0.00	0.99	0.99
Parabacteroides	0.03	0.77	0.85	0.10	0.28	0.74	0.11	0.21	0.70	0.13	0.16	0.68	0.14	0.12	0.61
Paraprevotella	0.00	0.98	1.00	0.02	0.70	0.86	0.02	0.76	0.97	0.03	0.67	0.97	0.01	0.85	0.99
Parasutterella	0.17	0.03	0.27	0.16	0.01	0.26	0.11	0.13	0.63	0.11	0.15	0.68	0.13	0.08	0.53
Peptococcus	-0.05	0.70	0.84	-0.17	0.16	0.60	-0.23	0.07	0.54	-0.22	0.08	0.56	-0.21	0.10	0.53
Phascolarctobacterium	0.05	0.51	0.71	0.05	0.45	0.76	0.06	0.40	0.97	0.06	0.34	0.90	0.09	0.20	0.71
Phocea	-0.15	0.41	0.66	-0.25	0.13	0.56	-0.35	0.04	0.54	-0.35	0.04	0.53	-0.34	0.04	0.53
Prevotella	-0.07	0.25	0.53	-0.05	0.38	0.75	-0.05	0.33	0.89	-0.05	0.33	0.90	-0.06	0.31	0.87
Romboutsia	0.09	0.41	0.66	0.13	0.17	0.61	0.12	0.24	0.74	0.12	0.24	0.70	0.10	0.35	0.88
Roseburia	0.35	0.01	0.25	0.24	0.07	0.40	0.09	0.52	0.97	0.08	0.54	0.97	0.08	0.56	0.99
Ruminococcus	0.08	0.32	0.62	0.04	0.63	0.85	-0.02	0.83	0.97	-0.02	0.85	0.97	-0.02	0.81	0.99
Sellimonas	0.16	0.17	0.49	0.18	0.08	0.46	0.19	0.09	0.54	0.19	0.08	0.56	0.19	0.08	0.53
Senegalimassilia	0.04	0.70	0.84	0.03	0.78	0.87	0.01	0.94	0.97	0.02	0.88	0.97	0.01	0.91	0.99
Shuttleworthia	0.11	0.45	0.68	0.04	0.78	0.87	0.01	0.96	0.97	0.00	1.00	1.00	0.00	0.99	0.99
Slackia	-0.06	0.49	0.70	-0.04	0.68	0.85	-0.06	0.54	0.97	-0.05	0.60	0.97	-0.04	0.68	0.99
Streptococcus	0.06	0.70	0.84	0.07	0.61	0.84	0.10	0.50	0.97	0.13	0.38	0.95	0.09	0.54	0.99
Subdoligranulum	0.14	0.21	0.51	0.08	0.44	0.75	0.03	0.75	0.97	0.04	0.70	0.97	0.05	0.64	0.99
Sutterella	-0.09	0.17	0.49	-0.05	0.40	0.75	-0.01	0.82	0.97	-0.01	0.87	0.97	-0.02	0.80	0.99
TM7x	-0.04	0.78	0.85	-0.11	0.42	0.75	-0.03	0.83	0.97	-0.02	0.88	0.97	-0.02	0.87	0.99
Turicibacter	0.29	0.04	0.30	0.42	0.00	0.06	0.42	0.00	0.18	0.41	0.00	0.23	0.40	0.00	0.16
Tuzzerella	-0.13	0.22	0.51	-0.12	0.21	0.63	-0.14	0.17	0.70	-0.15	0.16	0.68	-0.14	0.18	0.71
UBA1819	-0.25	0.12	0.42	-0.30	0.04	0.35	-0.28	0.07	0.54	-0.27	0.09	0.56	-0.26	0.10	0.54
UCG.002	0.02	0.77	0.85	-0.06	0.40	0.75	-0.10	0.20	0.70	-0.10	0.23	0.70	-0.08	0.34	0.88
UCG.003	0.16	0.07	0.39	0.07	0.34	0.75	0.03	0.76	0.97	0.03	0.71	0.97	0.04	0.64	0.99
UCG.005	0.14	0.16	0.49	0.07	0.41	0.75	0.01	0.92	0.97	0.03	0.80	0.97	0.03	0.73	0.99
UCG.009	0.14	0.31	0.62	0.05	0.68	0.85	-0.04	0.77	0.97	-0.03	0.81	0.97	-0.03	0.84	0.99
Veillonella	0.14	0.14	0.45	0.18	0.05	0.35	0.15	0.12	0.62	0.15	0.12	0.60	0.14	0.15	0.71
Victivallis	0.11	0.26	0.55	0.06	0.49	0.79	0.01	0.90	0.97	0.01	0.91	0.97	0.01	0.92	0.99

Multivariable-adjusted regression models adjusted for:
Model 1: sequencing batch, age, race, gender, study center
Model 2: Model 1, with education

© 2022 Meyer K et al. *JAMA Network Open.*

Model 3: Model 2, with physical activity, diet score, medication use, and smoking
Model 4: Model 3 with BMI
Model 5: Model 4 with hypertension and diabetes

© 2022 Meyer K et al. *JAMA Network Open.*

eTable 54: Multivariable-adjusted regression coefficients, p-values, and false discovery rate (FDR)-adjusted p-values for genera among female participants with respect to DSST.

	Model1			Model2			Model3			Model4			Model5		
	beta	p-value	FDR	beta	p-value	FDR	beta	p-value	FDR	beta	p-value	FDR	beta	p-value	FDR
Acetanaerobacterium	-0.05	0.98	0.98	0.77	0.65	0.92	0.75	0.67	0.99	0.42	0.81	1.00	0.17	0.92	0.99
Acidaminococcus	-1.28	0.03	0.35	-0.94	0.11	0.61	-0.57	0.37	0.94	-0.55	0.38	0.99	-0.76	0.24	0.97
Actinomyces	-0.14	0.93	0.98	0.28	0.85	0.97	1.20	0.46	0.94	1.43	0.37	0.99	1.81	0.26	0.97
Adlercreutzia	2.26	0.02	0.23	1.95	0.04	0.52	1.59	0.11	0.91	1.38	0.17	0.99	1.24	0.21	0.97
Agathobacter	-0.35	0.76	0.91	-0.23	0.83	0.97	-0.69	0.55	0.96	-0.43	0.71	1.00	-0.31	0.78	0.97
Akkermansia	1.44	0.01	0.23	1.30	0.02	0.48	1.34	0.03	0.87	1.28	0.03	0.99	1.37	0.02	0.56
Alistipes	-0.13	0.89	0.98	-0.11	0.91	0.99	-0.37	0.70	0.99	-0.43	0.66	1.00	-0.47	0.63	0.97
Anaerofilum	2.40	0.07	0.39	1.47	0.26	0.75	1.27	0.34	0.94	1.02	0.44	0.99	1.37	0.31	0.97
Anaerostipes	-0.60	0.66	0.88	-0.78	0.56	0.90	-0.62	0.65	0.98	-0.68	0.62	1.00	-0.83	0.55	0.97
Anaerotruncus	2.05	0.09	0.41	1.88	0.11	0.61	1.63	0.19	0.91	1.20	0.34	0.99	0.85	0.50	0.97
Angelakisella	-1.64	0.35	0.69	-2.27	0.18	0.68	-1.56	0.40	0.94	-1.90	0.30	0.99	-1.98	0.28	0.97
Bacteroides	-2.72	0.26	0.64	-3.27	0.17	0.68	-2.98	0.21	0.91	-2.63	0.27	0.99	-2.41	0.31	0.97
Barnesiella	1.34	0.02	0.23	1.30	0.02	0.43	1.34	0.02	0.87	1.34	0.02	0.99	1.54	0.01	0.56
Bifidobacterium	0.13	0.86	0.98	0.53	0.48	0.88	0.08	0.92	0.99	0.07	0.93	1.00	0.17	0.83	0.97
Bilophila	-0.17	0.83	0.95	-0.14	0.85	0.97	-0.48	0.52	0.96	-0.52	0.48	0.99	-0.54	0.48	0.97
Blautia	2.30	0.39	0.73	2.42	0.35	0.80	3.30	0.21	0.91	3.07	0.24	0.99	3.20	0.24	0.97
Butyricicoccus	1.41	0.41	0.73	0.62	0.71	0.92	0.40	0.82	0.99	0.62	0.72	1.00	0.55	0.75	0.97
Butyricimonas	0.22	0.73	0.91	0.05	0.94	0.99	-0.09	0.89	0.99	0.00	1.00	1.00	-0.03	0.96	0.99
CAG.56	0.66	0.40	0.73	0.54	0.48	0.88	0.07	0.93	0.99	0.11	0.89	1.00	0.38	0.64	0.97
Candidatus_Soleaferrea	2.06	0.20	0.60	1.98	0.21	0.72	2.06	0.21	0.91	1.94	0.23	0.99	1.88	0.25	0.97
Caproiciproducens	1.51	0.24	0.63	1.65	0.18	0.68	0.54	0.68	0.99	0.35	0.79	1.00	0.08	0.95	0.99
Christensenellaceae_R.7_group	1.62	0.01	0.23	1.33	0.03	0.52	0.91	0.19	0.91	0.75	0.28	0.99	0.89	0.20	0.97
Clostridium_sensu_stricto_1	-0.30	0.73	0.91	-0.37	0.66	0.92	-0.58	0.50	0.96	-0.57	0.50	0.99	-0.59	0.49	0.97
Colidextribacter	-1.62	0.34	0.69	-1.73	0.29	0.75	-1.26	0.47	0.95	-1.63	0.35	0.99	-1.38	0.44	0.97
Collinsella	-1.06	0.13	0.47	-0.46	0.51	0.88	-0.16	0.82	0.99	0.00	1.00	1.00	0.21	0.77	0.97
Coprobacter	-0.38	0.63	0.88	-0.70	0.37	0.82	-0.89	0.29	0.94	-0.95	0.25	0.99	-0.75	0.37	0.97
Coprococcus	0.37	0.65	0.88	0.67	0.40	0.84	0.71	0.39	0.94	0.78	0.35	0.99	0.96	0.25	0.97
Defluviitaleaceae_UCG.011	2.89	0.02	0.23	2.44	0.04	0.52	1.28	0.31	0.94	0.96	0.44	0.99	0.51	0.69	0.97
Desulfovibrio	0.30	0.69	0.89	0.40	0.58	0.90	0.42	0.58	0.96	0.35	0.64	1.00	0.29	0.70	0.97
Dialister	0.22	0.69	0.89	0.21	0.70	0.92	0.23	0.69	0.99	0.24	0.67	1.00	0.32	0.58	0.97
DNF00809	1.82	0.19	0.60	1.72	0.20	0.71	1.64	0.24	0.93	1.58	0.26	0.99	1.52	0.28	0.97
Dorea	-1.07	0.31	0.68	-0.67	0.52	0.88	-0.80	0.45	0.94	-0.61	0.57	1.00	-0.67	0.53	0.97
DTU089	0.44	0.70	0.89	0.21	0.85	0.97	0.14	0.90	0.99	0.05	0.97	1.00	-0.03	0.98	0.99
Eggerthella	1.19	0.27	0.64	1.14	0.27	0.75	1.42	0.20	0.91	1.24	0.26	0.99	1.44	0.19	0.97
Eisenbergiella	1.73	0.10	0.43	1.67	0.10	0.61	1.59	0.13	0.91	1.24	0.24	0.99	1.26	0.23	0.97
Erysipelatoclostridium	0.53	0.65	0.88	0.47	0.68	0.92	0.72	0.54	0.96	0.48	0.68	1.00	0.58	0.62	0.97
Erysipelotrichaceae_UCG.003	0.80	0.34	0.69	0.78	0.34	0.80	0.17	0.85	0.99	0.09	0.92	1.00	0.20	0.82	0.97
Escherichia.Shigella	0.88	0.24	0.63	1.04	0.15	0.68	1.23	0.11	0.91	1.30	0.09	0.99	1.15	0.15	0.97
Faecalibacterium	1.09	0.30	0.66	0.60	0.56	0.90	0.07	0.95	0.99	0.23	0.83	1.00	0.44	0.68	0.97
Family_XIII_AD3011_group	2.02	0.05	0.38	1.79	0.08	0.61	1.36	0.22	0.92	1.17	0.29	0.99	1.08	0.33	0.97
Family_XIII_UCG.001	0.91	0.49	0.81	0.68	0.60	0.91	0.20	0.88	0.99	-0.07	0.96	1.00	-0.28	0.83	0.97
Flavonifractor	-0.81	0.49	0.81	-0.84	0.46	0.88	-0.55	0.64	0.98	-0.57	0.63	1.00	-0.65	0.59	0.97
Frisingicoccus	0.02	0.98	0.98	-0.40	0.63	0.92	-0.50	0.57	0.96	-0.76	0.39	0.99	-0.64	0.47	0.97
Fusicatenibacter	-0.04	0.96	0.98	-0.29	0.71	0.92	-0.17	0.84	0.99	0.08	0.92	1.00	0.10	0.91	0.99
Fusobacterium	0.03	0.98	0.98	0.29	0.83	0.97	1.15	0.42	0.94	0.95	0.50	0.99	0.80	0.57	0.97
GCA.900066575	0.34	0.78	0.92	-0.22	0.85	0.97	-1.29	0.30	0.94	-1.59	0.20	0.99	-1.50	0.24	0.97
GCA.900066755	0.88	0.64	0.88	1.02	0.58	0.90	1.03	0.58	0.96	0.52	0.78	1.00	1.25	0.51	0.97
Gordonibacter	1.39	0.22	0.63	1.16	0.30	0.75	0.20	0.86	0.99	-0.16	0.89	1.00	-0.28	0.81	0.97
Granulicatella	-0.13	0.93	0.98	-0.20	0.89	0.99	-0.73	0.63	0.98	-0.49	0.75	1.00	-0.09	0.95	0.99
Haemophilus	0.58	0.53	0.81	0.22	0.81	0.97	-0.34	0.71	0.99	-0.36	0.70	1.00	-0.09	0.92	0.99
Harryflintia	-0.03	0.98	0.98	-0.01	1.00	1.00	0.29	0.83	0.99	-0.03	0.98	1.00	0.07	0.96	0.99

© 2022 Meyer K et al. *JAMA Network Open.*

Holdemania	1.68	0.40	0.73	1.50	0.44	0.88	1.53	0.45	0.94	1.29	0.52	0.99	1.32	0.51	0.97
Hungatella	0.97	0.34	0.69	1.20	0.23	0.75	1.72	0.10	0.91	1.70	0.10	0.99	1.72	0.10	0.97
Hydrogenoanaerobacterium	0.74	0.49	0.81	0.17	0.87	0.98	-0.22	0.84	0.99	-0.64	0.57	1.00	-0.54	0.63	0.97
Incertae_Sedis	2.61	0.13	0.47	2.59	0.12	0.61	2.52	0.15	0.91	2.37	0.18	0.99	2.40	0.17	0.97
Intestinibacter	1.39	0.20	0.60	1.62	0.12	0.61	1.66	0.13	0.91	1.93	0.07	0.99	2.34	0.03	0.56
Intestinimonas	1.26	0.18	0.60	0.99	0.28	0.75	0.62	0.52	0.96	0.24	0.81	1.00	0.36	0.72	0.97
Lachnoclostridium	-4.13	0.04	0.36	-3.02	0.13	0.61	-1.87	0.38	0.94	-1.61	0.44	0.99	-1.84	0.38	0.97
Lachnospira	2.98	0.00	0.11	2.35	0.02	0.43	1.80	0.08	0.91	1.98	0.05	0.99	2.20	0.03	0.56
Lachnospiraceae_FCS020_group	3.82	0.00	0.04	3.14	0.00	0.35	2.78	0.01	0.87	2.85	0.01	0.99	2.70	0.02	0.56
Lachnospiraceae_ND3007_group	-0.47	0.51	0.81	-0.78	0.26	0.75	-1.13	0.12	0.91	-0.96	0.18	0.99	-0.80	0.27	0.97
Lachnospiraceae_NK4A136_grou	0.49	0.64	0.88	-0.09	0.93	0.99	-0.98	0.38	0.94	-0.82	0.46	0.99	-0.67	0.55	0.97
Lachnospiraceae_UCG.001	1.43	0.07	0.39	1.26	0.10	0.61	1.23	0.12	0.91	1.07	0.18	0.99	1.30	0.10	0.97
Lachnospiraceae_UCG.004	0.53	0.51	0.81	0.42	0.59	0.90	0.11	0.89	0.99	0.21	0.79	1.00	0.22	0.79	0.97
Lachnospiraceae_UCG.010	0.38	0.75	0.91	-0.07	0.95	0.99	-0.37	0.76	0.99	-0.47	0.70	1.00	-0.27	0.83	0.97
Lactobacillus	-1.21	0.20	0.60	-0.67	0.47	0.88	-1.00	0.31	0.94	-0.65	0.51	0.99	-0.45	0.66	0.97
Lactococcus	0.56	0.68	0.89	0.51	0.70	0.92	0.07	0.96	0.99	-0.02	0.99	1.00	0.20	0.89	0.98
Marvinbryantia	0.92	0.25	0.63	0.79	0.31	0.75	0.38	0.65	0.98	0.38	0.64	1.00	0.54	0.51	0.97
Megasphaera	-0.14	0.86	0.98	0.28	0.71	0.92	0.70	0.37	0.94	0.77	0.33	0.99	0.70	0.39	0.97
Monoglobus	3.01	0.00	0.16	2.78	0.01	0.37	2.27	0.04	0.91	2.21	0.04	0.99	2.57	0.02	0.56
Moryella	1.46	0.29	0.66	0.90	0.51	0.88	0.35	0.81	0.99	0.56	0.70	1.00	0.61	0.67	0.97
Negativibacillus	-0.54	0.52	0.81	-0.25	0.76	0.94	0.04	0.97	0.99	-0.04	0.97	1.00	0.01	0.99	0.99
NK4A214_group	0.66	0.32	0.68	0.50	0.43	0.88	0.38	0.57	0.96	0.27	0.69	1.00	0.46	0.50	0.97
Odoribacter	0.38	0.55	0.81	0.18	0.78	0.95	0.00	1.00	1.00	-0.03	0.97	1.00	-0.09	0.89	0.98
Oscillibacter	2.12	0.05	0.38	1.85	0.09	0.61	1.49	0.18	0.91	1.18	0.29	0.99	1.20	0.29	0.97
Oscillospira	-0.09	0.95	0.98	0.09	0.95	0.99	0.14	0.93	0.99	0.07	0.97	1.00	0.33	0.83	0.97
Oxalobacter	1.70	0.13	0.47	0.91	0.41	0.84	0.89	0.45	0.94	0.85	0.47	0.99	0.54	0.65	0.97
Paludicola	2.19	0.06	0.38	1.89	0.10	0.61	0.96	0.42	0.94	0.59	0.62	1.00	0.64	0.60	0.97
Parabacteroides	-0.90	0.27	0.64	-0.66	0.41	0.84	-0.46	0.57	0.96	-0.18	0.83	1.00	0.01	0.99	0.99
Paraprevotella	-1.30	0.04	0.36	-1.21	0.05	0.59	-0.98	0.14	0.91	-1.01	0.12	0.99	-0.87	0.18	0.97
Parasutterella	0.50	0.41	0.73	0.37	0.54	0.89	0.44	0.49	0.96	0.48	0.45	0.99	0.36	0.57	0.97
Peptococcus	0.82	0.53	0.81	0.40	0.76	0.94	0.35	0.80	0.99	0.22	0.87	1.00	0.53	0.69	0.97
Phascolarctobacterium	-0.09	0.87	0.98	0.00	1.00	1.00	-0.08	0.89	0.99	-0.19	0.74	1.00	-0.36	0.54	0.97
Phocea	-0.08	0.96	0.98	-0.16	0.91	0.99	0.12	0.93	0.99	0.34	0.82	1.00	0.69	0.64	0.97
Prevotella	-0.91	0.12	0.47	-0.59	0.31	0.75	-0.50	0.40	0.94	-0.43	0.47	0.99	-0.52	0.39	0.97
Romboutsia	-0.07	0.95	0.98	0.04	0.97	1.00	-0.14	0.89	0.99	0.02	0.98	1.00	0.34	0.74	0.97
Roseburia	0.64	0.55	0.81	0.69	0.50	0.88	0.13	0.90	0.99	0.34	0.76	1.00	0.40	0.72	0.97
Ruminococcus	1.73	0.01	0.23	1.32	0.06	0.59	1.01	0.17	0.91	0.97	0.18	0.99	1.16	0.12	0.97
Sellimonas	1.64	0.12	0.47	1.12	0.28	0.75	1.08	0.31	0.94	0.80	0.45	0.99	0.84	0.43	0.97
Shuttleworthia	0.87	0.38	0.73	-0.02	0.99	1.00	-0.34	0.74	0.99	-0.58	0.57	1.00	-0.32	0.76	0.97
Slackia	-1.52	0.09	0.41	-1.45	0.10	0.61	-1.40	0.13	0.91	-1.21	0.18	0.99	-1.44	0.11	0.97
Streptococcus	0.77	0.54	0.81	0.82	0.50	0.88	0.16	0.90	0.99	0.27	0.83	1.00	0.84	0.52	0.97
Subdoligranulum	1.78	0.05	0.38	1.49	0.09	0.61	0.73	0.44	0.94	0.79	0.40	0.99	0.83	0.38	0.97
Sutterella	-0.96	0.08	0.41	-0.64	0.24	0.75	-0.66	0.24	0.93	-0.61	0.27	0.99	-0.51	0.37	0.97
TM7x	1.50	0.24	0.63	1.11	0.37	0.82	0.96	0.45	0.94	1.00	0.43	0.99	1.64	0.20	0.97
Turicibacter	0.45	0.75	0.91	0.60	0.67	0.92	0.62	0.66	0.99	0.55	0.70	1.00	0.23	0.87	0.98
Tuzzerella	-1.13	0.19	0.60	-0.90	0.29	0.75	-0.69	0.45	0.94	-0.85	0.35	0.99	-0.92	0.31	0.97
UBA1819	1.47	0.27	0.64	1.46	0.26	0.75	2.01	0.14	0.91	1.56	0.26	0.99	1.25	0.38	0.97
UCG.002	1.07	0.09	0.41	0.83	0.18	0.68	0.57	0.40	0.94	0.48	0.48	0.99	0.56	0.41	0.97
UCG.003	0.58	0.44	0.76	0.32	0.66	0.92	0.01	0.99	1.00	0.06	0.94	1.00	0.15	0.85	0.97
UCG.005	1.46	0.07	0.40	1.10	0.17	0.68	0.41	0.64	0.98	0.25	0.77	1.00	0.27	0.75	0.97
UCG.009	0.29	0.82	0.95	-0.53	0.68	0.92	-0.75	0.57	0.96	-0.86	0.51	0.99	-0.78	0.55	0.97
Veillonella	0.43	0.65	0.88	0.30	0.75	0.94	0.04	0.97	0.99	0.22	0.82	1.00	0.67	0.51	0.97

Multivariable-adjusted regression models adjusted for:
Model 1: sequencing batch, age, race, gender, study center
Model 2: Model 1, with education

© 2022 Meyer K et al. *JAMA Network Open.*

Model 3: Model 2, with physical activity, diet score, medication use, and smoking
Model 4: Model 3 with BMI
Model 5: Model 4 with hypertension and diabetes

© 2022 Meyer K et al. *JAMA Network Open.*

eTable 55: Multivariable-adjusted regression coefficients, p-values, and false discovery rate (FDR)-adjusted p-values for genera among female participants with respect to Stroop.

	Model1			Model2			Model3			Model4			Model5		
	beta	p-value	FDR	beta	p-value	FDR	beta	p-value	FDR	beta	p-value	FDR	beta	p-value	FDR
Acetanaerobacterium	-0.27	0.81	0.90	-0.47	0.69	0.83	-0.43	0.73	0.85	-0.29	0.81	0.91	-0.41	0.75	0.87
Acidaminococcus	0.45	0.26	0.53	0.36	0.38	0.66	0.22	0.62	0.82	0.21	0.63	0.84	0.24	0.59	0.79
Actinomyces	-0.19	0.86	0.94	-0.30	0.77	0.86	-0.71	0.53	0.75	-0.83	0.47	0.73	-0.86	0.45	0.71
Adlercreutzia	-1.60	0.01	0.14	-1.53	0.02	0.18	-1.45	0.04	0.30	-1.37	0.05	0.32	-1.35	0.06	0.30
Agathobacter	0.26	0.72	0.85	0.24	0.75	0.86	0.42	0.60	0.81	0.31	0.69	0.84	0.27	0.74	0.87
Akkermansia	-1.03	0.01	0.09	-1.00	0.01	0.13	-0.95	0.02	0.24	-0.92	0.03	0.29	-0.93	0.03	0.24
Alistipes	-0.58	0.36	0.64	-0.58	0.36	0.64	-0.53	0.43	0.70	-0.51	0.45	0.71	-0.51	0.46	0.71
Anaerofilum	-1.73	0.05	0.21	-1.53	0.08	0.29	-1.68	0.07	0.31	-1.58	0.09	0.39	-1.79	0.06	0.30
Anaerostipes	0.31	0.74	0.85	0.36	0.69	0.83	0.37	0.70	0.85	0.40	0.68	0.84	0.41	0.68	0.86
Anaerotruncus	-1.58	0.05	0.21	-1.54	0.06	0.26	-1.66	0.06	0.30	-1.50	0.09	0.39	-1.59	0.08	0.34
Angelakisella	-1.55	0.18	0.42	-1.41	0.22	0.50	-1.49	0.24	0.57	-1.37	0.29	0.64	-1.47	0.26	0.56
Bacteroides	-3.58	0.03	0.19	-3.43	0.03	0.24	-3.28	0.05	0.30	-3.45	0.04	0.31	-3.58	0.03	0.24
Barnesiella	-0.84	0.02	0.19	-0.84	0.02	0.20	-0.95	0.02	0.23	-0.95	0.02	0.29	-1.06	0.01	0.23
Bifidobacterium	-0.06	0.90	0.98	-0.16	0.76	0.86	-0.09	0.87	0.92	-0.09	0.87	0.94	-0.08	0.88	0.94
Bilophila	-0.60	0.23	0.50	-0.61	0.22	0.50	-0.55	0.30	0.60	-0.53	0.32	0.64	-0.50	0.35	0.66
Blautia	-1.86	0.29	0.58	-1.88	0.28	0.57	-2.48	0.17	0.49	-2.39	0.19	0.55	-2.93	0.13	0.47
Butyricicoccus	-0.87	0.45	0.69	-0.66	0.57	0.80	-0.30	0.81	0.90	-0.41	0.74	0.87	-0.27	0.83	0.91
Butyricimonas	-1.24	0.00	0.09	-1.21	0.00	0.12	-1.11	0.02	0.23	-1.16	0.01	0.29	-1.15	0.01	0.23
CAG.56	-0.60	0.25	0.52	-0.57	0.28	0.57	-0.44	0.44	0.70	-0.46	0.43	0.71	-0.59	0.31	0.61
Candidatus_Soleaferrea	-0.85	0.43	0.69	-0.85	0.43	0.70	-1.16	0.31	0.61	-1.12	0.33	0.64	-1.26	0.28	0.58
Caproiciproducens	-1.33	0.12	0.34	-1.35	0.11	0.35	-1.20	0.19	0.51	-1.12	0.22	0.56	-1.14	0.22	0.54
Christensenellaceae_R.7_group	-1.24	0.00	0.09	-1.18	0.01	0.12	-1.16	0.02	0.23	-1.09	0.02	0.29	-1.19	0.02	0.23
Clostridium_sensu_stricto_1	0.45	0.44	0.69	0.46	0.43	0.70	0.47	0.43	0.70	0.47	0.44	0.71	0.47	0.44	0.71
Colidextribacter	-0.99	0.38	0.65	-0.94	0.40	0.68	-1.32	0.28	0.59	-1.19	0.33	0.64	-1.62	0.20	0.53
Collinsella	0.36	0.44	0.69	0.21	0.66	0.83	0.17	0.73	0.85	0.11	0.83	0.92	0.12	0.82	0.91
Coprobacter	0.32	0.55	0.76	0.40	0.46	0.73	0.45	0.44	0.70	0.48	0.41	0.71	0.45	0.45	0.71
Coprococcus	0.34	0.53	0.75	0.25	0.64	0.83	0.11	0.85	0.92	0.09	0.88	0.94	-0.01	0.98	0.98
Defluviitaleaceae_UCG.011	-2.29	0.00	0.09	-2.19	0.01	0.12	-2.23	0.01	0.23	-2.12	0.02	0.29	-2.17	0.02	0.23
Desulfovibrio	0.01	0.99	0.99	-0.01	0.98	0.99	0.02	0.97	0.98	0.05	0.93	0.96	-0.02	0.98	0.98
Dialister	0.34	0.36	0.64	0.35	0.35	0.64	0.48	0.24	0.57	0.47	0.25	0.59	0.42	0.31	0.61
DNF00809	-0.53	0.56	0.76	-0.52	0.57	0.80	-0.66	0.50	0.73	-0.63	0.52	0.76	-0.63	0.52	0.76
Dorea	-0.52	0.46	0.69	-0.62	0.38	0.66	-0.62	0.41	0.70	-0.71	0.34	0.64	-0.67	0.37	0.66
DTU089	-0.91	0.24	0.51	-0.86	0.26	0.56	-0.93	0.25	0.57	-0.90	0.27	0.61	-1.02	0.21	0.54
Eggerthella	0.05	0.95	0.98	0.06	0.93	0.98	-0.08	0.91	0.95	0.00	1.00	1.00	-0.11	0.89	0.94
Eisenbergiella	-1.12	0.11	0.33	-1.11	0.11	0.36	-1.30	0.08	0.31	-1.17	0.11	0.44	-1.16	0.12	0.47
Erysipelatoclostridium	-1.46	0.06	0.21	-1.45	0.06	0.26	-1.50	0.07	0.31	-1.41	0.09	0.39	-1.45	0.08	0.35
Erysipelotrichaceae_UCG.003	-1.08	0.05	0.21	-1.06	0.06	0.26	-0.91	0.13	0.42	-0.88	0.15	0.49	-0.95	0.12	0.47
Escherichia.Shigella	0.04	0.94	0.98	0.00	1.00	1.00	-0.12	0.83	0.92	-0.15	0.79	0.90	-0.14	0.80	0.90
Faecalibacterium	-1.17	0.09	0.29	-1.05	0.13	0.36	-0.83	0.26	0.57	-0.90	0.22	0.56	-0.98	0.20	0.53
Family_XIII_AD3011_group	-1.32	0.06	0.21	-1.25	0.07	0.28	-1.07	0.17	0.49	-0.99	0.20	0.55	-1.00	0.21	0.53
Family_XIII_UCG.001	-1.64	0.06	0.21	-1.57	0.08	0.28	-1.59	0.10	0.37	-1.49	0.12	0.44	-1.48	0.13	0.47
Flavonifractor	-0.33	0.67	0.82	-0.32	0.68	0.83	-0.45	0.59	0.81	-0.45	0.59	0.81	-0.54	0.52	0.76
Frisingicoccus	-0.82	0.15	0.38	-0.72	0.21	0.50	-0.58	0.34	0.65	-0.49	0.43	0.71	-0.51	0.41	0.69
Fusicatenibacter	-0.30	0.57	0.76	-0.25	0.65	0.83	-0.47	0.43	0.70	-0.59	0.33	0.64	-0.56	0.36	0.66
Fusobacterium	1.71	0.06	0.21	1.64	0.07	0.28	1.49	0.13	0.42	1.58	0.11	0.44	1.50	0.14	0.47
GCA.900066575	-1.31	0.10	0.32	-1.17	0.14	0.38	-0.84	0.33	0.64	-0.73	0.40	0.70	-0.97	0.28	0.58
GCA.900066755	-1.02	0.41	0.68	-1.05	0.40	0.68	-1.48	0.26	0.57	-1.28	0.33	0.64	-1.58	0.24	0.56
Gordonibacter	-1.07	0.16	0.39	-1.02	0.18	0.46	-0.70	0.39	0.70	-0.56	0.49	0.74	-0.54	0.52	0.76
Granulicatella	-0.05	0.96	0.98	-0.05	0.96	0.99	0.02	0.98	0.98	-0.08	0.94	0.96	-0.12	0.91	0.94
Haemophilus	0.14	0.82	0.90	0.23	0.71	0.84	0.46	0.48	0.73	0.47	0.47	0.73	0.33	0.61	0.80
Harryflintia	-1.64	0.06	0.21	-1.65	0.06	0.26	-1.89	0.04	0.30	-1.78	0.06	0.32	-2.07	0.03	0.24
Holdemania	-2.02	0.13	0.34	-1.99	0.13	0.36	-2.29	0.10	0.37	-2.19	0.12	0.44	-2.37	0.10	0.41

© 2022 Meyer K et al. *JAMA Network Open.*

Hungatella	-0.29	0.67	0.82	-0.34	0.62	0.83	-0.91	0.21	0.56	-0.90	0.22	0.56	-0.86	0.24	0.56
Hydrogenoanaerobacterium	-1.33	0.06	0.21	-1.21	0.09	0.30	-1.27	0.10	0.37	-1.12	0.15	0.49	-1.17	0.14	0.47
Incertae_Sedis	-1.42	0.22	0.49	-1.42	0.22	0.50	-1.30	0.29	0.60	-1.24	0.32	0.64	-1.41	0.26	0.56
Intestinibacter	-0.34	0.64	0.82	-0.39	0.58	0.80	-0.32	0.68	0.83	-0.43	0.57	0.81	-0.49	0.53	0.76
Intestinimonas	-1.77	0.00	0.09	-1.72	0.01	0.12	-1.68	0.01	0.23	-1.56	0.02	0.29	-1.62	0.02	0.24
Lachnoclostridium	2.60	0.05	0.21	2.36	0.08	0.29	2.00	0.17	0.49	1.90	0.20	0.55	2.08	0.16	0.50
Lachnospira	-1.00	0.13	0.34	-0.84	0.20	0.50	-0.79	0.27	0.58	-0.87	0.23	0.56	-1.06	0.15	0.49
Lachnospiraceae_FCS020_group	-1.47	0.04	0.21	-1.31	0.07	0.28	-0.98	0.22	0.56	-1.01	0.20	0.55	-0.95	0.24	0.56
Lachnospiraceae_ND3007_group	0.18	0.70	0.82	0.26	0.58	0.80	0.36	0.48	0.73	0.29	0.57	0.81	0.21	0.69	0.86
Lachnospiraceae_NK4A136_group	0.63	0.37	0.64	0.80	0.26	0.56	1.19	0.13	0.42	1.13	0.15	0.49	1.01	0.21	0.53
Lachnospiraceae_UCG.001	-0.21	0.69	0.82	-0.17	0.74	0.86	-0.06	0.91	0.95	0.01	0.99	1.00	-0.07	0.90	0.94
Lachnospiraceae_UCG.004	0.33	0.53	0.75	0.37	0.48	0.74	0.47	0.41	0.70	0.43	0.45	0.71	0.41	0.48	0.74
Lachnospiraceae_UCG.010	0.02	0.98	0.99	0.15	0.85	0.91	0.27	0.75	0.86	0.32	0.71	0.84	0.28	0.74	0.87
Lactobacillus	0.78	0.21	0.49	0.64	0.31	0.61	0.53	0.45	0.70	0.38	0.58	0.81	0.47	0.51	0.76
Lactococcus	0.36	0.69	0.82	0.37	0.68	0.83	0.44	0.65	0.83	0.49	0.62	0.82	0.60	0.55	0.76
Marvinbryantia	0.53	0.31	0.59	0.58	0.27	0.57	0.57	0.32	0.62	0.57	0.32	0.64	0.48	0.41	0.69
Megasphaera	0.26	0.61	0.79	0.15	0.77	0.86	-0.25	0.65	0.83	-0.28	0.61	0.82	-0.20	0.73	0.87
Monoglobus	-2.48	0.00	0.04	-2.43	0.00	0.05	-2.28	0.00	0.14	-2.26	0.00	0.22	-2.49	0.00	0.14
Moryella	-0.58	0.53	0.75	-0.43	0.65	0.83	-0.05	0.96	0.98	-0.13	0.90	0.95	-0.29	0.78	0.88
Negativibacillus	-0.75	0.17	0.41	-0.83	0.13	0.36	-1.17	0.05	0.30	-1.14	0.05	0.32	-1.21	0.05	0.29
NK4A214_group	-0.95	0.03	0.20	-0.91	0.04	0.24	-0.97	0.04	0.30	-0.93	0.05	0.32	-1.03	0.03	0.24
Odoribacter	-0.33	0.44	0.69	-0.28	0.51	0.75	-0.19	0.68	0.83	-0.18	0.70	0.84	-0.25	0.60	0.79
Oscillibacter	-2.00	0.01	0.09	-1.93	0.01	0.12	-1.87	0.02	0.23	-1.76	0.03	0.29	-1.81	0.02	0.24
Oscillospira	-2.10	0.04	0.21	-2.15	0.03	0.24	-2.29	0.03	0.30	-2.27	0.03	0.31	-2.70	0.02	0.23
Oxalobacter	-0.34	0.65	0.82	-0.13	0.87	0.92	0.35	0.67	0.83	0.37	0.66	0.84	0.52	0.54	0.76
Paludicola	-1.56	0.04	0.21	-1.49	0.05	0.26	-1.33	0.11	0.38	-1.19	0.16	0.49	-1.15	0.18	0.52
Parabacteroides	-0.31	0.57	0.76	-0.37	0.50	0.75	-0.39	0.50	0.73	-0.52	0.37	0.68	-0.57	0.33	0.64
Paraprevotella	0.81	0.06	0.21	0.79	0.06	0.27	0.89	0.05	0.30	0.90	0.05	0.32	0.87	0.06	0.30
Parasutterella	-0.84	0.04	0.21	-0.80	0.05	0.26	-0.81	0.07	0.31	-0.83	0.06	0.32	-0.85	0.06	0.30
Peptococcus	-0.62	0.47	0.70	-0.53	0.55	0.79	-0.45	0.64	0.83	-0.39	0.68	0.84	-0.36	0.71	0.87
Phascolarctobacterium	-0.58	0.14	0.35	-0.61	0.12	0.36	-0.67	0.11	0.37	-0.63	0.13	0.46	-0.57	0.18	0.52
Phocea	0.89	0.36	0.64	0.92	0.34	0.64	1.17	0.26	0.57	1.08	0.30	0.64	0.89	0.40	0.68
Prevotella	1.04	0.01	0.09	0.97	0.01	0.16	0.97	0.02	0.23	0.94	0.02	0.29	0.93	0.03	0.24
Romboutsia	-0.48	0.47	0.69	-0.52	0.43	0.70	-0.53	0.45	0.70	-0.60	0.39	0.70	-0.69	0.34	0.65
Roseburia	0.22	0.76	0.87	0.20	0.77	0.86	0.29	0.71	0.85	0.20	0.79	0.90	0.10	0.90	0.94
Ruminococcus	-0.53	0.26	0.53	-0.44	0.36	0.64	-0.45	0.38	0.70	-0.44	0.39	0.70	-0.48	0.36	0.66
Sellimonas	-0.60	0.39	0.66	-0.47	0.50	0.75	-0.40	0.59	0.81	-0.28	0.71	0.84	-0.37	0.63	0.80
Shuttleworthia	-1.57	0.02	0.14	-1.40	0.04	0.24	-1.05	0.15	0.45	-0.97	0.19	0.55	-1.01	0.18	0.52
Slackia	0.05	0.94	0.98	0.02	0.97	0.99	-0.02	0.97	0.98	-0.11	0.86	0.94	-0.08	0.90	0.94
Streptococcus	-1.27	0.13	0.34	-1.28	0.12	0.36	-1.65	0.07	0.31	-1.70	0.06	0.32	-1.70	0.07	0.31
Subdoligranulum	-1.26	0.04	0.21	-1.18	0.05	0.26	-1.32	0.04	0.30	-1.35	0.04	0.31	-1.54	0.02	0.24
Sutterella	0.57	0.12	0.34	0.49	0.18	0.46	0.46	0.24	0.57	0.44	0.26	0.61	0.46	0.26	0.56
TM7x	-0.86	0.31	0.59	-0.78	0.36	0.64	-0.59	0.51	0.73	-0.61	0.49	0.74	-0.80	0.38	0.67
Turicibacter	0.11	0.91	0.98	0.06	0.95	0.99	0.35	0.73	0.85	0.37	0.71	0.84	0.41	0.69	0.86
Tuzzerella	-0.26	0.65	0.82	-0.32	0.59	0.80	-0.11	0.86	0.92	-0.05	0.94	0.96	-0.03	0.97	0.98
UBA1819	-2.36	0.01	0.09	-2.35	0.01	0.12	-2.88	0.00	0.14	-2.74	0.00	0.22	-2.98	0.00	0.14
UCG.002	-0.25	0.55	0.76	-0.18	0.66	0.83	-0.10	0.84	0.92	-0.06	0.91	0.95	-0.15	0.76	0.88
UCG.003	0.03	0.95	0.98	0.09	0.85	0.91	0.15	0.78	0.89	0.13	0.81	0.91	0.16	0.77	0.88
UCG.005	-1.40	0.01	0.11	-1.31	0.02	0.16	-1.13	0.06	0.31	-1.07	0.07	0.36	-1.20	0.05	0.30
UCG.009	-0.80	0.36	0.64	-0.61	0.48	0.74	-0.46	0.62	0.82	-0.42	0.65	0.84	-0.55	0.56	0.76
Veillonella	0.15	0.81	0.90	0.18	0.78	0.86	0.46	0.51	0.73	0.38	0.58	0.81	0.38	0.59	0.79

Multivariable-adjusted regression models adjusted for:
Model 1: sequencing batch, age, race, gender, study center
Model 2: Model 1, with education
Model 3: Model 2, with physical activity, diet score, medication use, and smoking
Model 4: Model 3 with BMI

© 2022 Meyer K et al. *JAMA Network Open.*

Model 5: Model 4 with hypertension and diabetes

© 2022 Meyer K et al. *JAMA Network Open.*

eTable 56: Multivariable-adjusted regression coefficients, p-values, and false discovery rate (FDR)-adjusted p-values for genera among female participants with respect to RAVLT.

	Model1			Model2			Model3			Model4			Model5		
	beta	p-value	FDR	beta	p-value	FDR	beta	p-value	FDR	beta	p-value	FDR	beta	p-value	FDR
Acetanaerobacterium	-0.05	0.79	0.95	0.05	0.78	0.95	-0.05	0.81	0.93	-0.07	0.72	0.91	-0.10	0.60	0.91
Acidaminococcus	-0.10	0.12	0.44	-0.06	0.37	0.78	-0.02	0.76	0.92	-0.02	0.77	0.91	-0.02	0.76	0.92
Actinomyces	-0.08	0.65	0.89	-0.02	0.88	0.98	-0.01	0.94	0.99	0.00	1.00	1.00	0.03	0.85	0.93
Adlercreutzia	0.21	0.05	0.40	0.17	0.10	0.66	0.13	0.23	0.87	0.12	0.28	0.89	0.11	0.34	0.91
Agathobacter	-0.19	0.12	0.44	-0.18	0.14	0.66	-0.19	0.14	0.87	-0.17	0.18	0.89	-0.16	0.21	0.91
Akkermansia	0.09	0.16	0.48	0.07	0.24	0.71	0.06	0.38	0.87	0.05	0.42	0.89	0.05	0.47	0.91
Alistipes	0.13	0.22	0.54	0.13	0.20	0.66	0.06	0.55	0.87	0.06	0.57	0.89	0.05	0.63	0.91
Anaerofilum	0.27	0.07	0.42	0.15	0.30	0.78	0.14	0.36	0.87	0.12	0.42	0.89	0.15	0.31	0.91
Anaerostipes	-0.09	0.54	0.87	-0.12	0.43	0.79	-0.15	0.34	0.87	-0.15	0.32	0.89	-0.16	0.29	0.91
Anaerotruncus	0.03	0.85	0.96	0.00	0.98	0.99	-0.09	0.52	0.87	-0.12	0.39	0.89	-0.13	0.35	0.91
Angelakisella	0.24	0.22	0.54	0.16	0.39	0.78	0.13	0.52	0.87	0.11	0.59	0.89	0.09	0.65	0.91
Bacteroides	-0.27	0.32	0.67	-0.34	0.20	0.66	-0.29	0.28	0.87	-0.27	0.31	0.89	-0.24	0.38	0.91
Barnesiella	0.11	0.09	0.44	0.10	0.10	0.66	0.07	0.30	0.87	0.07	0.30	0.89	0.08	0.21	0.91
Bifidobacterium	0.00	0.99	1.00	0.05	0.55	0.88	0.03	0.73	0.92	0.03	0.73	0.91	0.02	0.80	0.92
Bilophila	0.13	0.11	0.44	0.14	0.09	0.66	0.12	0.16	0.87	0.11	0.17	0.89	0.10	0.21	0.91
Blautia	-0.21	0.47	0.79	-0.20	0.49	0.86	-0.12	0.69	0.92	-0.13	0.65	0.90	-0.10	0.74	0.92
Butyricicoccus	0.06	0.75	0.95	-0.04	0.82	0.96	-0.13	0.49	0.87	-0.12	0.54	0.89	-0.14	0.48	0.91
Butyricimonas	0.11	0.13	0.44	0.09	0.20	0.66	0.09	0.20	0.87	0.10	0.17	0.89	0.09	0.21	0.91
CAG.56	-0.01	0.86	0.97	-0.03	0.71	0.92	-0.08	0.38	0.87	-0.08	0.40	0.89	-0.05	0.55	0.91
Candidatus_Soleaferrea	-0.01	0.94	0.98	-0.02	0.89	0.98	0.02	0.89	0.95	0.02	0.92	0.96	0.00	0.98	0.98
Caproiciproducens	0.16	0.26	0.59	0.18	0.19	0.66	0.11	0.46	0.87	0.09	0.51	0.89	0.08	0.59	0.91
Christensenellaceae_R.7_group	0.14	0.05	0.40	0.10	0.14	0.66	0.04	0.59	0.89	0.03	0.69	0.91	0.04	0.61	0.91
Clostridium_sensu_stricto_1	0.18	0.06	0.42	0.17	0.07	0.66	0.15	0.12	0.87	0.15	0.12	0.89	0.14	0.15	0.91
Colidextribacter	-0.27	0.15	0.47	-0.29	0.11	0.66	-0.36	0.06	0.87	-0.39	0.04	0.89	-0.37	0.07	0.91
Collinsella	-0.20	0.01	0.31	-0.13	0.10	0.66	-0.11	0.18	0.87	-0.10	0.22	0.89	-0.10	0.23	0.91
Coprobacter	-0.04	0.67	0.89	-0.08	0.36	0.78	-0.12	0.18	0.87	-0.13	0.17	0.89	-0.12	0.20	0.91
Coprococcus	-0.08	0.37	0.72	-0.04	0.62	0.88	-0.07	0.47	0.87	-0.06	0.50	0.89	-0.04	0.67	0.91
Defluviitaleaceae_UCG.011	0.23	0.09	0.44	0.17	0.19	0.66	0.09	0.50	0.87	0.07	0.60	0.89	0.04	0.79	0.92
Desulfovibrio	-0.04	0.62	0.87	-0.03	0.72	0.92	-0.07	0.43	0.87	-0.07	0.40	0.89	-0.07	0.43	0.91
Dialister	-0.05	0.45	0.79	-0.05	0.42	0.78	-0.07	0.27	0.87	-0.07	0.28	0.89	-0.06	0.36	0.91
DNF00809	0.01	0.94	0.98	0.00	0.99	0.99	-0.02	0.89	0.95	-0.02	0.87	0.96	-0.03	0.85	0.93
Dorea	-0.12	0.30	0.62	-0.07	0.54	0.88	-0.07	0.52	0.87	-0.06	0.59	0.89	-0.07	0.56	0.91
DTU089	-0.05	0.68	0.89	-0.08	0.51	0.87	-0.10	0.42	0.87	-0.11	0.40	0.89	-0.09	0.47	0.91
Eggerthella	0.07	0.58	0.87	0.06	0.60	0.88	0.02	0.85	0.93	0.01	0.93	0.96	0.04	0.73	0.92
Eisenbergiella	-0.03	0.82	0.95	-0.03	0.76	0.94	-0.04	0.70	0.92	-0.07	0.55	0.89	-0.06	0.59	0.91
Erysipelatoclostridium	0.00	1.00	1.00	-0.01	0.95	0.99	-0.04	0.74	0.92	-0.06	0.65	0.90	-0.06	0.63	0.91
Erysipelotrichaceae_UCG.003	0.02	0.83	0.95	0.02	0.85	0.97	-0.02	0.83	0.93	-0.03	0.79	0.92	-0.04	0.71	0.92
Escherichia.Shigella	0.01	0.94	0.98	0.03	0.74	0.93	0.04	0.63	0.92	0.05	0.59	0.89	0.06	0.53	0.91
Faecalibacterium	0.16	0.17	0.50	0.09	0.40	0.78	0.07	0.53	0.87	0.08	0.47	0.89	0.08	0.49	0.91
Family_XIII_AD3011_group	0.08	0.48	0.79	0.05	0.64	0.88	-0.04	0.72	0.92	-0.06	0.65	0.90	-0.07	0.55	0.91
Family_XIII_UCG.001	0.22	0.13	0.44	0.19	0.17	0.66	0.13	0.38	0.87	0.12	0.44	0.89	0.10	0.52	0.91
Flavonifractor	-0.07	0.57	0.87	-0.08	0.53	0.88	-0.07	0.57	0.88	-0.08	0.56	0.89	-0.07	0.59	0.91
Frisingicoccus	0.05	0.60	0.87	0.00	0.97	0.99	-0.07	0.50	0.87	-0.08	0.40	0.89	-0.07	0.48	0.91
Fusicatenibacter	-0.04	0.66	0.89	-0.07	0.41	0.78	-0.07	0.43	0.87	-0.06	0.53	0.89	-0.07	0.44	0.91
Fusobacterium	-0.25	0.11	0.44	-0.22	0.15	0.66	-0.10	0.54	0.87	-0.11	0.48	0.89	-0.12	0.46	0.91
GCA.900066575	0.02	0.88	0.97	-0.05	0.68	0.90	-0.15	0.29	0.87	-0.17	0.23	0.89	-0.18	0.21	0.91
GCA.900066755	0.26	0.22	0.54	0.27	0.17	0.66	0.24	0.25	0.87	0.21	0.32	0.89	0.25	0.23	0.91
Gordonibacter	0.34	0.01	0.31	0.31	0.01	0.66	0.21	0.10	0.87	0.19	0.14	0.89	0.19	0.14	0.91
Granulicatella	0.10	0.57	0.87	0.09	0.60	0.88	0.05	0.77	0.92	0.07	0.70	0.91	0.10	0.58	0.91
Haemophilus	0.13	0.21	0.54	0.08	0.41	0.78	0.04	0.69	0.92	0.04	0.70	0.91	0.06	0.54	0.91
Harryflintia	0.08	0.59	0.87	0.08	0.56	0.88	0.07	0.66	0.92	0.05	0.76	0.91	0.05	0.75	0.92

© 2022 Meyer K et al. *JAMA Network Open.*

Holdemania	0.24	0.28	0.60	0.22	0.31	0.78	0.15	0.51	0.87	0.13	0.55	0.89	0.09	0.70	0.92
Hungatella	-0.23	0.04	0.40	-0.20	0.07	0.66	-0.13	0.27	0.87	-0.13	0.26	0.89	-0.12	0.29	0.91
Hydrogenoanaerobacterium	0.25	0.03	0.40	0.18	0.12	0.66	0.14	0.26	0.87	0.12	0.36	0.89	0.12	0.33	0.91
Incertae_Sedis	0.03	0.87	0.97	0.03	0.88	0.98	-0.07	0.73	0.92	-0.08	0.70	0.91	-0.07	0.73	0.92
Intestinibacter	0.07	0.55	0.87	0.10	0.38	0.78	0.08	0.53	0.87	0.09	0.44	0.89	0.11	0.35	0.91
Intestinimonas	0.25	0.02	0.35	0.22	0.03	0.66	0.19	0.08	0.87	0.17	0.12	0.89	0.19	0.08	0.91
Lachnoclostridium	-0.36	0.11	0.44	-0.21	0.34	0.78	-0.05	0.82	0.93	-0.04	0.87	0.96	-0.04	0.87	0.93
Lachnospira	0.20	0.07	0.43	0.11	0.32	0.78	0.06	0.60	0.89	0.07	0.53	0.89	0.08	0.48	0.91
Lachnospiraceae_FCS020_group	0.25	0.04	0.40	0.15	0.20	0.66	0.07	0.57	0.88	0.08	0.54	0.89	0.06	0.65	0.91
Lachnospiraceae_ND3007_grou	0.00	0.96	0.98	-0.04	0.64	0.88	-0.09	0.27	0.87	-0.08	0.33	0.89	-0.07	0.38	0.91
Lachnospiraceae_NK4A136_gro	-0.03	0.83	0.95	-0.10	0.37	0.78	-0.21	0.09	0.87	-0.20	0.11	0.89	-0.19	0.12	0.91
Lachnospiraceae_UCG.001	0.06	0.47	0.79	0.04	0.62	0.88	0.02	0.82	0.93	0.01	0.91	0.96	0.03	0.78	0.92
Lachnospiraceae_UCG.004	0.01	0.93	0.98	-0.01	0.94	0.99	0.00	0.99	0.99	0.00	0.96	0.97	-0.01	0.95	0.96
Lachnospiraceae_UCG.010	-0.19	0.16	0.48	-0.25	0.05	0.66	-0.26	0.05	0.87	-0.27	0.05	0.89	-0.26	0.05	0.91
Lactobacillus	-0.16	0.12	0.44	-0.09	0.36	0.78	-0.12	0.27	0.87	-0.10	0.36	0.89	-0.09	0.42	0.91
Lactococcus	0.17	0.26	0.59	0.16	0.26	0.73	0.14	0.37	0.87	0.13	0.38	0.89	0.13	0.41	0.91
Marvinbryantia	-0.12	0.18	0.50	-0.14	0.11	0.66	-0.10	0.27	0.87	-0.10	0.28	0.89	-0.08	0.39	0.91
Megasphaera	0.03	0.69	0.89	0.09	0.28	0.75	0.15	0.08	0.87	0.16	0.07	0.89	0.17	0.07	0.91
Monoglobus	0.16	0.18	0.50	0.13	0.26	0.73	0.10	0.41	0.87	0.10	0.43	0.89	0.11	0.39	0.91
Moryella	0.12	0.46	0.79	0.04	0.78	0.95	0.01	0.96	0.99	0.02	0.90	0.96	0.05	0.78	0.92
Negativibacillus	-0.08	0.38	0.72	-0.04	0.63	0.88	-0.02	0.87	0.94	-0.02	0.83	0.95	-0.02	0.87	0.93
NK4A214_group	0.02	0.76	0.95	0.00	0.98	0.99	-0.03	0.69	0.92	-0.04	0.62	0.90	-0.02	0.79	0.92
Odoribacter	0.09	0.21	0.54	0.06	0.37	0.78	0.05	0.53	0.87	0.04	0.54	0.89	0.04	0.58	0.91
Oscillibacter	0.18	0.14	0.46	0.15	0.22	0.67	0.08	0.55	0.87	0.06	0.66	0.90	0.05	0.69	0.92
Oscillospira	-0.04	0.83	0.95	-0.01	0.93	0.99	-0.05	0.75	0.92	-0.06	0.73	0.91	-0.02	0.89	0.93
Oxalobacter	0.26	0.03	0.40	0.16	0.18	0.66	0.04	0.75	0.92	0.04	0.76	0.91	0.03	0.84	0.93
Paludicola	0.05	0.70	0.91	0.01	0.95	0.99	-0.14	0.29	0.87	-0.17	0.21	0.89	-0.16	0.25	0.91
Parabacteroides	-0.03	0.78	0.95	0.01	0.94	0.99	0.07	0.47	0.87	0.09	0.35	0.89	0.10	0.28	0.91
Paraprevotella	0.02	0.81	0.95	0.03	0.67	0.90	0.02	0.74	0.92	0.02	0.76	0.91	0.03	0.66	0.91
Parasutterella	0.16	0.02	0.35	0.14	0.03	0.66	0.11	0.11	0.87	0.12	0.10	0.89	0.10	0.16	0.91
Peptococcus	0.29	0.05	0.40	0.23	0.10	0.66	0.19	0.22	0.87	0.18	0.23	0.89	0.20	0.18	0.91
Phascolarctobacterium	0.07	0.26	0.59	0.09	0.17	0.66	0.10	0.14	0.87	0.09	0.17	0.89	0.08	0.25	0.91
Phocea	-0.13	0.41	0.75	-0.14	0.36	0.78	-0.19	0.25	0.87	-0.18	0.28	0.89	-0.15	0.35	0.91
Prevotella	-0.06	0.37	0.72	-0.02	0.80	0.96	0.00	0.98	0.99	0.01	0.92	0.96	0.01	0.90	0.93
Romboutsia	0.01	0.96	0.98	0.02	0.85	0.97	0.00	0.98	0.99	0.01	0.95	0.97	0.01	0.94	0.96
Roseburia	-0.06	0.59	0.87	-0.06	0.62	0.88	-0.05	0.70	0.92	-0.03	0.78	0.91	-0.02	0.87	0.93
Ruminococcus	0.09	0.27	0.59	0.03	0.68	0.90	0.02	0.84	0.93	0.01	0.86	0.96	0.02	0.83	0.93
Sellimonas	0.21	0.08	0.43	0.14	0.22	0.67	0.15	0.22	0.87	0.13	0.28	0.89	0.15	0.22	0.91
Shuttleworthia	0.28	0.01	0.31	0.18	0.10	0.66	0.11	0.34	0.87	0.10	0.41	0.89	0.13	0.28	0.91
Slackia	-0.05	0.58	0.87	-0.05	0.64	0.88	-0.02	0.82	0.93	-0.01	0.92	0.96	-0.03	0.75	0.92
Streptococcus	0.18	0.19	0.51	0.19	0.16	0.66	0.21	0.14	0.87	0.22	0.13	0.89	0.24	0.11	0.91
Subdoligranulum	0.16	0.11	0.44	0.12	0.21	0.66	0.07	0.50	0.87	0.08	0.47	0.89	0.09	0.38	0.91
Sutterella	-0.11	0.09	0.44	-0.06	0.29	0.76	-0.05	0.40	0.87	-0.05	0.43	0.89	-0.05	0.43	0.91
TM7x	-0.04	0.77	0.95	-0.09	0.49	0.86	-0.16	0.25	0.87	-0.16	0.26	0.89	-0.12	0.40	0.91
Turicibacter	0.31	0.05	0.40	0.33	0.03	0.66	0.34	0.03	0.87	0.34	0.03	0.89	0.30	0.06	0.91
Tuzzerella	-0.08	0.38	0.72	-0.05	0.57	0.88	-0.10	0.34	0.87	-0.11	0.29	0.89	-0.13	0.21	0.91
UBA1819	0.07	0.65	0.89	0.07	0.64	0.88	0.01	0.97	0.99	-0.03	0.87	0.96	-0.02	0.90	0.93
UCG.002	0.13	0.06	0.42	0.10	0.15	0.66	0.06	0.44	0.87	0.05	0.48	0.89	0.06	0.45	0.91
UCG.003	0.01	0.95	0.98	-0.03	0.72	0.92	-0.07	0.38	0.87	-0.07	0.40	0.89	-0.08	0.34	0.91
UCG.005	0.07	0.47	0.79	0.02	0.85	0.97	-0.07	0.45	0.87	-0.08	0.39	0.89	-0.09	0.37	0.91
UCG.009	0.07	0.61	0.87	-0.03	0.82	0.96	-0.04	0.79	0.93	-0.05	0.75	0.91	-0.03	0.84	0.93
Veillonella	0.10	0.37	0.72	0.08	0.44	0.79	0.04	0.70	0.92	0.05	0.62	0.90	0.07	0.54	0.91

Multivariable-adjusted regression models adjusted for:
Model 1: sequencing batch, age, race, gender, study center
Model 2: Model 1, with education

© 2022 Meyer K et al. *JAMA Network Open.*

Model 3: Model 2, with physical activity, diet score, medication use, and smoking
Model 4: Model 3 with BMI
Model 5: Model 4 with hypertension and diabetes

© 2022 Meyer K et al. *JAMA Network Open.*

eTable 57: Multivariable-adjusted regression coefficients, p-values, and false discovery rate (FDR)-adjusted p-values for genera among female participants with respect to category fluency.

	Model1			Model2			Model3			Model4			Model5		
	beta	p-value	FDR	beta	p-value	FDR	beta	p-value	FDR	beta	p-value	FDR	beta	p-value	FDR
Acetanaerobacterium	-0.20	0.71	0.87	0.07	0.89	0.98	0.15	0.79	0.96	0.11	0.85	0.99	0.09	0.87	1.00
Acidaminococcus	-0.36	0.06	0.43	-0.25	0.19	0.79	-0.20	0.35	0.95	-0.19	0.35	0.94	-0.22	0.29	1.00
Actinomyces	-0.92	0.06	0.43	-0.79	0.10	0.71	-0.77	0.14	0.89	-0.74	0.16	0.94	-0.69	0.19	1.00
Adlercreutzia	-0.04	0.89	0.92	-0.15	0.61	0.94	-0.15	0.64	0.96	-0.18	0.58	0.99	-0.22	0.51	1.00
Agathobacter	-0.27	0.45	0.72	-0.23	0.51	0.90	-0.35	0.34	0.95	-0.32	0.39	0.94	-0.27	0.47	1.00
Akkermansia	0.21	0.25	0.72	0.17	0.36	0.86	0.17	0.39	0.95	0.16	0.42	0.94	0.14	0.49	1.00
Alistipes	0.06	0.85	0.92	0.07	0.82	0.95	-0.06	0.84	0.96	-0.07	0.82	0.99	-0.08	0.80	1.00
Anaerofilum	0.79	0.06	0.43	0.47	0.25	0.85	0.49	0.26	0.95	0.45	0.30	0.94	0.50	0.26	1.00
Anaerostipes	0.07	0.87	0.92	0.01	0.98	1.00	-0.10	0.83	0.96	-0.10	0.81	0.99	-0.11	0.81	1.00
Anaerotruncus	0.63	0.10	0.50	0.57	0.13	0.79	0.60	0.14	0.89	0.55	0.18	0.94	0.55	0.19	1.00
Angelakisella	0.47	0.40	0.72	0.26	0.63	0.94	0.32	0.60	0.96	0.27	0.65	0.99	0.26	0.67	1.00
Bacteroides	-0.76	0.32	0.72	-0.94	0.21	0.79	-0.93	0.23	0.94	-0.89	0.25	0.94	-0.78	0.32	1.00
Barnesiella	0.51	0.00	0.12	0.50	0.00	0.28	0.62	0.00	0.10	0.62	0.00	0.10	0.64	0.00	0.07
Bifidobacterium	-0.37	0.12	0.53	-0.24	0.30	0.85	-0.28	0.27	0.95	-0.28	0.27	0.94	-0.31	0.23	1.00
Bilophila	0.41	0.08	0.47	0.42	0.07	0.67	0.43	0.08	0.89	0.43	0.08	0.94	0.40	0.11	0.99
Blautia	-0.40	0.64	0.82	-0.36	0.66	0.94	-0.42	0.62	0.96	-0.46	0.59	0.99	-0.43	0.63	1.00
Butyricicoccus	0.47	0.39	0.72	0.20	0.70	0.94	0.21	0.72	0.96	0.24	0.68	0.99	0.19	0.74	1.00
Butyricimonas	0.33	0.11	0.51	0.27	0.17	0.79	0.32	0.13	0.89	0.34	0.11	0.94	0.35	0.11	0.99
CAG.56	-0.02	0.94	0.95	-0.06	0.80	0.95	-0.09	0.73	0.96	-0.08	0.75	0.99	-0.05	0.84	1.00
Candidatus_Soleaferrea	0.53	0.30	0.72	0.50	0.31	0.85	0.60	0.26	0.95	0.58	0.27	0.94	0.57	0.29	1.00
Caproiciproducens	-0.04	0.93	0.95	0.01	0.98	1.00	0.09	0.83	0.96	0.07	0.87	0.99	-0.01	0.98	1.00
Christensenellaceae_R.7_group	0.37	0.07	0.47	0.26	0.19	0.79	0.29	0.20	0.94	0.27	0.23	0.94	0.27	0.24	1.00
Clostridium_sensu_stricto_1	-0.18	0.51	0.75	-0.21	0.44	0.89	-0.27	0.34	0.95	-0.27	0.34	0.94	-0.31	0.26	1.00
Colidextribacter	0.49	0.36	0.72	0.45	0.39	0.88	0.44	0.44	0.95	0.39	0.49	0.99	0.54	0.36	1.00
Collinsella	0.18	0.42	0.72	0.41	0.06	0.67	0.41	0.08	0.89	0.44	0.06	0.94	0.43	0.07	0.99
Coprobacter	0.16	0.53	0.75	0.05	0.83	0.95	-0.07	0.80	0.96	-0.08	0.78	0.99	-0.08	0.77	1.00
Coprococcus	-0.17	0.52	0.75	-0.07	0.79	0.95	-0.10	0.72	0.96	-0.09	0.74	0.99	-0.03	0.93	1.00
Defluviitaleaceae_UCG.011	0.82	0.03	0.43	0.67	0.08	0.67	0.80	0.05	0.89	0.76	0.06	0.94	0.69	0.10	0.99
Desulfovibrio	0.00	1.00	1.00	0.03	0.89	0.98	0.03	0.89	0.96	0.03	0.92	0.99	0.02	0.95	1.00
Dialister	-0.04	0.82	0.92	-0.04	0.80	0.95	-0.01	0.97	1.00	-0.01	0.98	1.00	0.03	0.87	1.00
DNF00809	0.06	0.89	0.92	0.02	0.96	0.99	0.01	0.99	1.00	0.00	1.00	1.00	-0.01	0.99	1.00
Dorea	-0.30	0.37	0.72	-0.16	0.62	0.94	-0.19	0.58	0.96	-0.17	0.63	0.99	-0.20	0.57	1.00
DTU089	-0.30	0.42	0.72	-0.37	0.29	0.85	-0.33	0.38	0.95	-0.34	0.36	0.94	-0.27	0.48	1.00
Eggerthella	-0.14	0.68	0.86	-0.16	0.64	0.94	-0.17	0.62	0.96	-0.20	0.57	0.99	-0.14	0.69	1.00
Eisenbergiella	0.25	0.46	0.72	0.23	0.49	0.89	0.20	0.56	0.96	0.15	0.66	0.99	0.17	0.62	1.00
Erysipelatoclostridium	-0.14	0.71	0.87	-0.16	0.66	0.94	-0.18	0.64	0.96	-0.21	0.58	0.99	-0.21	0.59	1.00
Erysipelotrichaceae_UCG.003	-0.07	0.80	0.92	-0.08	0.77	0.95	0.00	1.00	1.00	-0.01	0.97	1.00	-0.05	0.85	1.00
Escherichia.Shigella	-0.21	0.39	0.72	-0.15	0.51	0.90	-0.08	0.76	0.96	-0.07	0.78	0.99	-0.05	0.84	1.00
Faecalibacterium	0.05	0.89	0.92	-0.12	0.70	0.94	-0.23	0.50	0.95	-0.21	0.54	0.99	-0.21	0.54	1.00
Family_XIII_AD3011_group	0.16	0.64	0.82	0.08	0.81	0.95	0.07	0.85	0.96	0.04	0.91	0.99	-0.02	0.95	1.00
Family_XIII_UCG.001	0.78	0.06	0.43	0.70	0.08	0.67	0.70	0.11	0.89	0.67	0.13	0.94	0.61	0.17	1.00
Flavonifractor	-0.44	0.23	0.72	-0.46	0.20	0.79	-0.46	0.23	0.94	-0.46	0.23	0.94	-0.44	0.26	1.00
Frisingicoccus	0.48	0.08	0.47	0.35	0.20	0.79	0.26	0.36	0.95	0.23	0.42	0.94	0.25	0.38	1.00
Fusicatenibacter	0.26	0.31	0.72	0.18	0.48	0.89	0.05	0.87	0.96	0.08	0.77	0.99	0.00	1.00	1.00
Fusobacterium	-0.08	0.85	0.92	0.00	0.99	1.00	0.19	0.68	0.96	0.16	0.72	0.99	0.19	0.69	1.00
GCA.900066575	0.05	0.89	0.92	-0.14	0.71	0.94	-0.06	0.88	0.96	-0.10	0.81	0.99	-0.11	0.78	1.00
GCA.900066755	-0.45	0.45	0.72	-0.40	0.48	0.89	-0.42	0.48	0.95	-0.50	0.41	0.94	-0.45	0.47	1.00
Gordonibacter	0.52	0.15	0.62	0.44	0.21	0.79	0.54	0.15	0.89	0.50	0.19	0.94	0.49	0.21	1.00
Granulicatella	0.09	0.85	0.92	0.07	0.88	0.98	0.01	0.98	1.00	0.04	0.93	0.99	0.16	0.75	1.00
Haemophilus	0.14	0.63	0.82	0.02	0.95	0.99	-0.17	0.58	0.96	-0.17	0.58	0.99	-0.12	0.69	1.00
Harryflintia	0.36	0.39	0.72	0.36	0.37	0.86	0.33	0.45	0.95	0.29	0.51	0.99	0.30	0.49	1.00

© 2022 Meyer K et al. *JAMA Network Open.*

Holdemania	1.20	0.06	0.43	1.14	0.06	0.67	1.14	0.08	0.89	1.11	0.09	0.94	1.04	0.11	0.99
Hungatella	-0.17	0.59	0.80	-0.09	0.76	0.95	-0.08	0.82	0.96	-0.08	0.82	0.99	-0.04	0.90	1.00
Hydrogenoanaerobacterium	0.32	0.35	0.72	0.13	0.70	0.94	0.18	0.63	0.96	0.13	0.73	0.99	0.09	0.82	1.00
Incertae_Sedis	0.30	0.59	0.80	0.29	0.58	0.94	0.33	0.57	0.96	0.31	0.60	0.99	0.29	0.61	1.00
Intestinibacter	-0.53	0.12	0.53	-0.46	0.17	0.79	-0.50	0.16	0.89	-0.47	0.18	0.94	-0.45	0.21	1.00
Intestinimonas	0.88	0.00	0.12	0.79	0.01	0.28	0.80	0.01	0.59	0.77	0.02	0.84	0.83	0.01	0.50
Lachnoclostridium	-0.66	0.31	0.72	-0.27	0.67	0.94	-0.09	0.90	0.96	-0.05	0.94	0.99	-0.03	0.96	1.00
Lachnospira	0.52	0.09	0.48	0.29	0.34	0.86	0.26	0.42	0.95	0.29	0.38	0.94	0.33	0.33	1.00
Lachnospiraceae_FCS020_group	0.83	0.02	0.41	0.59	0.08	0.67	0.64	0.08	0.89	0.66	0.08	0.94	0.59	0.11	0.99
Lachnospiraceae_ND3007_group	0.24	0.29	0.72	0.14	0.53	0.91	0.04	0.87	0.96	0.06	0.79	0.99	0.07	0.76	1.00
Lachnospiraceae_NK4A136_grou	0.43	0.19	0.71	0.24	0.46	0.89	0.12	0.74	0.96	0.14	0.69	0.99	0.17	0.64	1.00
Lachnospiraceae_UCG.001	0.51	0.04	0.43	0.45	0.06	0.67	0.39	0.13	0.89	0.37	0.16	0.94	0.39	0.14	1.00
Lachnospiraceae_UCG.004	0.15	0.56	0.78	0.11	0.65	0.94	-0.03	0.91	0.97	-0.02	0.95	0.99	-0.05	0.85	1.00
Lachnospiraceae_UCG.010	-0.13	0.73	0.87	-0.29	0.44	0.89	-0.31	0.44	0.95	-0.32	0.42	0.94	-0.27	0.50	1.00
Lactobacillus	-0.22	0.45	0.72	-0.04	0.90	0.98	-0.08	0.80	0.96	-0.03	0.92	0.99	0.00	0.99	1.00
Lactococcus	0.31	0.47	0.72	0.29	0.48	0.89	0.25	0.57	0.96	0.24	0.59	0.99	0.22	0.63	1.00
Marvinbryantia	-0.26	0.29	0.72	-0.31	0.21	0.79	-0.25	0.34	0.95	-0.25	0.34	0.94	-0.18	0.50	1.00
Megasphaera	-0.15	0.52	0.75	-0.01	0.95	0.99	0.02	0.94	0.99	0.03	0.91	0.99	0.01	0.98	1.00
Monoglobus	0.73	0.03	0.43	0.65	0.05	0.67	0.66	0.06	0.89	0.66	0.06	0.94	0.67	0.07	0.99
Moryella	0.37	0.40	0.72	0.18	0.67	0.94	0.17	0.72	0.96	0.20	0.67	0.99	0.22	0.65	1.00
Negativibacillus	0.13	0.61	0.82	0.23	0.36	0.86	0.27	0.34	0.95	0.26	0.35	0.94	0.25	0.36	1.00
NK4A214_group	0.46	0.03	0.43	0.41	0.05	0.67	0.37	0.09	0.89	0.36	0.10	0.94	0.39	0.08	0.99
Odoribacter	0.18	0.38	0.72	0.11	0.58	0.94	0.06	0.79	0.96	0.05	0.80	0.99	0.03	0.89	1.00
Oscillibacter	0.45	0.20	0.72	0.35	0.30	0.85	0.46	0.21	0.94	0.42	0.25	0.94	0.39	0.29	1.00
Oscillospira	-0.39	0.41	0.72	-0.33	0.48	0.89	-0.22	0.66	0.96	-0.23	0.65	0.99	-0.14	0.78	1.00
Oxalobacter	0.46	0.19	0.71	0.19	0.59	0.94	0.28	0.46	0.95	0.28	0.47	0.99	0.23	0.55	1.00
Paludicola	0.14	0.71	0.87	0.03	0.93	0.99	0.05	0.90	0.96	0.00	0.99	1.00	-0.01	0.98	1.00
Parabacteroides	0.18	0.49	0.74	0.26	0.30	0.85	0.22	0.42	0.95	0.26	0.34	0.94	0.32	0.24	1.00
Paraprevotella	-0.18	0.37	0.72	-0.15	0.45	0.89	-0.15	0.49	0.95	-0.15	0.48	0.99	-0.14	0.50	1.00
Parasutterella	0.23	0.23	0.72	0.19	0.32	0.85	0.17	0.41	0.95	0.18	0.39	0.94	0.11	0.60	1.00
Peptococcus	0.94	0.02	0.43	0.80	0.05	0.67	0.68	0.12	0.89	0.66	0.13	0.94	0.72	0.10	0.99
Phascolarctobacterium	0.15	0.43	0.72	0.18	0.32	0.85	0.21	0.28	0.95	0.20	0.31	0.94	0.17	0.39	1.00
Phocea	-0.36	0.44	0.72	-0.38	0.39	0.88	-0.34	0.48	0.95	-0.31	0.51	0.99	-0.22	0.65	1.00
Prevotella	0.03	0.89	0.92	0.14	0.43	0.89	0.16	0.41	0.95	0.17	0.38	0.94	0.17	0.40	1.00
Romboutsia	0.07	0.82	0.92	0.11	0.72	0.94	0.06	0.86	0.96	0.08	0.80	0.99	0.09	0.80	1.00
Roseburia	-0.11	0.74	0.88	-0.09	0.78	0.95	-0.06	0.87	0.96	-0.03	0.93	0.99	0.05	0.90	1.00
Ruminococcus	0.38	0.09	0.48	0.24	0.28	0.85	0.16	0.50	0.95	0.16	0.51	0.99	0.13	0.58	1.00
Sellimonas	0.33	0.32	0.72	0.15	0.65	0.94	0.09	0.80	0.96	0.05	0.89	0.99	0.05	0.88	1.00
Shuttleworthia	0.33	0.29	0.72	0.03	0.92	0.99	0.09	0.80	0.96	0.06	0.87	0.99	0.11	0.74	1.00
Slackia	-0.11	0.69	0.87	-0.09	0.75	0.95	-0.07	0.81	0.96	-0.05	0.88	0.99	-0.11	0.71	1.00
Streptococcus	0.09	0.81	0.92	0.11	0.78	0.95	0.11	0.79	0.96	0.13	0.76	0.99	0.15	0.73	1.00
Subdoligranulum	0.84	0.00	0.12	0.74	0.01	0.28	0.66	0.03	0.89	0.67	0.03	0.94	0.73	0.02	0.63
Sutterella	-0.20	0.25	0.72	-0.09	0.61	0.94	-0.17	0.37	0.95	-0.16	0.38	0.94	-0.14	0.46	1.00
TM7x	-0.36	0.37	0.72	-0.50	0.20	0.79	-0.43	0.30	0.95	-0.42	0.31	0.94	-0.37	0.38	1.00
Turicibacter	-0.49	0.28	0.72	-0.44	0.32	0.85	-0.35	0.45	0.95	-0.36	0.44	0.96	-0.46	0.33	1.00
Tuzzerella	0.22	0.43	0.72	0.30	0.26	0.85	0.30	0.30	0.95	0.28	0.34	0.94	0.27	0.36	1.00
UBA1819	0.52	0.23	0.72	0.51	0.21	0.79	0.59	0.18	0.93	0.54	0.23	0.94	0.59	0.20	1.00
UCG.002	0.27	0.18	0.70	0.19	0.33	0.85	0.15	0.50	0.95	0.14	0.53	0.99	0.16	0.49	1.00
UCG.003	0.24	0.32	0.72	0.15	0.52	0.91	0.12	0.64	0.96	0.13	0.62	0.99	0.09	0.72	1.00
UCG.005	0.49	0.06	0.43	0.37	0.15	0.79	0.35	0.21	0.94	0.33	0.24	0.94	0.30	0.29	1.00
UCG.009	0.28	0.50	0.75	0.00	1.00	1.00	-0.03	0.95	0.99	-0.04	0.92	0.99	-0.02	0.95	1.00
Veillonella	-0.32	0.28	0.72	-0.37	0.21	0.79	-0.43	0.18	0.93	-0.41	0.20	0.94	-0.37	0.26	1.00

Multivariable-adjusted regression models adjusted for:
Model 1: sequencing batch, age, race, gender, study center
Model 2: Model 1, with education

© 2022 Meyer K et al. *JAMA Network Open.*

Model 3: Model 2, with physical activity, diet score, medication use, and smoking
Model 4: Model 3 with BMI
Model 5: Model 4 with hypertension and diabetes

© 2022 Meyer K et al. *JAMA Network Open.*

eTable 58: Multivariable-adjusted regression coefficients, p-values, and false discovery rate (FDR)-adjusted p-values for genera among female participants with respect to letter fluency.

	Model1			Model2			Model3			Model4			Model5		
	beta	p-value	FDR	beta	p-value	FDR	beta	p-value	FDR	beta	p-value	FDR	beta	p-value	FDR
Acetanaerobacterium	0.05	0.97	0.98	0.53	0.69	0.99	0.91	0.51	0.98	0.78	0.57	0.98	0.56	0.68	0.99
Acidaminococcus	-0.46	0.33	0.82	-0.25	0.59	0.92	-0.08	0.87	0.98	-0.07	0.88	0.99	-0.09	0.85	0.99
Actinomyces	-1.08	0.37	0.85	-0.82	0.48	0.91	0.18	0.88	0.98	0.28	0.83	0.99	0.48	0.71	0.99
Adlercreutzia	-0.82	0.28	0.82	-1.03	0.17	0.91	-1.15	0.14	0.94	-1.26	0.11	0.83	-1.38	0.08	0.84
Agathobacter	-0.20	0.82	0.95	-0.12	0.89	0.99	-0.37	0.68	0.98	-0.26	0.77	0.99	-0.11	0.90	0.99
Akkermansia	0.26	0.57	0.87	0.17	0.71	0.99	0.12	0.80	0.98	0.09	0.85	0.99	0.02	0.96	0.99
Alistipes	0.18	0.80	0.95	0.18	0.80	0.99	-0.07	0.92	0.99	-0.09	0.90	0.99	-0.19	0.80	0.99
Anaerofilum	0.97	0.34	0.82	0.38	0.71	0.99	0.23	0.83	0.98	0.12	0.91	0.99	-0.01	0.99	0.99
Anaerostipes	-1.79	0.09	0.78	-1.92	0.07	0.69	-1.89	0.08	0.94	-1.91	0.07	0.83	-1.90	0.08	0.84
Anaerotruncus	0.10	0.91	0.98	-0.02	0.98	0.99	-0.13	0.89	0.98	-0.34	0.73	0.99	-0.40	0.68	0.99
Angelakisella	1.30	0.33	0.82	0.94	0.48	0.91	1.70	0.23	0.94	1.57	0.27	0.94	1.31	0.36	0.96
Bacteroides	-1.50	0.42	0.85	-1.88	0.30	0.91	-2.12	0.25	0.94	-1.97	0.29	0.94	-2.01	0.28	0.96
Barnesiella	0.95	0.03	0.71	0.93	0.03	0.60	1.03	0.02	0.94	1.03	0.02	0.83	1.05	0.02	0.84
Bifidobacterium	-0.18	0.77	0.95	0.09	0.88	0.99	-0.01	0.99	1.00	-0.01	0.99	0.99	-0.05	0.94	0.99
Bilophila	1.16	0.04	0.71	1.16	0.04	0.63	0.86	0.14	0.94	0.84	0.15	0.83	0.69	0.24	0.96
Blautia	-3.99	0.05	0.71	-3.96	0.05	0.63	-3.86	0.06	0.94	-3.97	0.05	0.83	-3.99	0.06	0.84
Butyricicoccus	-0.37	0.78	0.95	-0.90	0.50	0.91	-1.92	0.16	0.94	-1.84	0.18	0.83	-1.76	0.20	0.96
Butyricimonas	0.54	0.28	0.82	0.45	0.36	0.91	0.38	0.46	0.98	0.42	0.41	0.95	0.40	0.43	0.96
CAG.56	0.48	0.43	0.85	0.42	0.48	0.91	0.63	0.32	0.94	0.64	0.31	0.94	0.68	0.29	0.96
Candidatus_Soleaferrea	-0.89	0.48	0.85	-0.99	0.42	0.91	-0.80	0.53	0.98	-0.85	0.50	0.95	-1.02	0.42	0.96
Caproiciproducens	-0.12	0.90	0.98	-0.03	0.98	0.99	-0.70	0.49	0.98	-0.78	0.44	0.95	-0.97	0.34	0.96
Christensenellaceae_R.7_group	0.72	0.15	0.80	0.54	0.27	0.91	0.21	0.70	0.98	0.13	0.81	0.99	0.08	0.88	0.99
Clostridium_sensu_stricto_1	-0.10	0.88	0.97	-0.13	0.85	0.99	-0.15	0.82	0.98	-0.15	0.82	0.99	-0.33	0.62	0.99
Colidextribacter	0.81	0.53	0.86	0.70	0.58	0.92	0.30	0.82	0.98	0.16	0.91	0.99	0.38	0.79	0.99
Collinsella	-0.69	0.20	0.82	-0.30	0.58	0.92	-0.49	0.39	0.95	-0.42	0.45	0.95	-0.47	0.41	0.96
Coprobacter	0.15	0.81	0.95	-0.09	0.89	0.99	-0.60	0.37	0.95	-0.63	0.35	0.94	-0.66	0.33	0.96
Coprococcus	-0.12	0.85	0.96	0.06	0.92	0.99	0.00	1.00	1.00	0.03	0.96	0.99	0.10	0.88	0.99
Defluviitaleaceae_UCG.011	0.21	0.83	0.95	-0.11	0.91	0.99	-0.49	0.61	0.98	-0.65	0.51	0.95	-0.94	0.35	0.96
Desulfovibrio	-0.19	0.75	0.95	-0.10	0.86	0.99	-0.22	0.71	0.98	-0.26	0.67	0.99	-0.26	0.67	0.99
Dialister	-0.49	0.26	0.82	-0.50	0.24	0.91	-0.41	0.37	0.95	-0.40	0.37	0.95	-0.38	0.41	0.96
DNF00809	-1.01	0.34	0.82	-1.08	0.30	0.91	-1.46	0.18	0.94	-1.48	0.18	0.83	-1.54	0.16	0.96
Dorea	-0.69	0.39	0.85	-0.46	0.57	0.92	-0.87	0.29	0.94	-0.80	0.33	0.94	-0.85	0.30	0.96
DTU089	-0.73	0.41	0.85	-0.87	0.32	0.91	-1.18	0.19	0.94	-1.22	0.18	0.83	-1.04	0.25	0.96
Eggerthella	-1.70	0.04	0.71	-1.78	0.03	0.60	-1.41	0.10	0.94	-1.49	0.09	0.83	-1.40	0.11	0.96
Eisenbergiella	-0.46	0.57	0.87	-0.51	0.53	0.92	-0.81	0.32	0.94	-0.99	0.23	0.94	-0.83	0.32	0.96
Erysipelatoclostridium	-0.38	0.67	0.95	-0.45	0.61	0.94	-0.40	0.66	0.98	-0.51	0.58	0.98	-0.53	0.56	0.99
Erysipelotrichaceae_UCG.003	0.44	0.50	0.86	0.42	0.51	0.91	0.15	0.83	0.98	0.11	0.87	0.99	-0.02	0.98	0.99
Escherichia.Shigella	-1.11	0.06	0.71	-1.00	0.08	0.71	-0.88	0.14	0.94	-0.86	0.16	0.83	-0.90	0.15	0.96
Faecalibacterium	0.50	0.54	0.86	0.20	0.80	0.99	0.17	0.84	0.98	0.24	0.77	0.99	0.17	0.84	0.99
Family_XIII_AD3011_group	0.31	0.71	0.95	0.19	0.82	0.99	0.05	0.95	0.99	-0.04	0.96	0.99	-0.12	0.89	0.99
Family_XIII_UCG.001	1.43	0.16	0.80	1.28	0.20	0.91	1.20	0.26	0.94	1.10	0.30	0.94	1.05	0.32	0.96
Flavonifractor	-2.05	0.02	0.71	-2.08	0.02	0.60	-2.04	0.03	0.94	-2.04	0.03	0.83	-2.11	0.02	0.84
Frisingicoccus	0.20	0.76	0.95	-0.10	0.88	0.99	-0.38	0.58	0.98	-0.49	0.48	0.95	-0.47	0.50	0.96
Fusicatenibacter	0.34	0.58	0.87	0.18	0.77	0.99	-0.30	0.65	0.98	-0.20	0.77	0.99	-0.36	0.59	0.99
Fusobacterium	1.70	0.12	0.80	1.77	0.10	0.73	2.33	0.04	0.94	2.26	0.05	0.83	2.17	0.05	0.84
GCA.900066575	1.04	0.26	0.82	0.72	0.44	0.91	0.21	0.83	0.98	0.08	0.93	0.99	-0.18	0.86	0.99
GCA.900066755	-0.84	0.56	0.87	-0.80	0.57	0.92	-0.93	0.53	0.98	-1.18	0.43	0.95	-1.22	0.41	0.96
Gordonibacter	0.64	0.47	0.85	0.48	0.58	0.92	0.21	0.82	0.98	0.06	0.95	0.99	-0.01	0.99	0.99
Granulicatella	0.90	0.44	0.85	0.87	0.45	0.91	1.48	0.22	0.94	1.60	0.18	0.83	1.87	0.12	0.96
Haemophilus	0.51	0.47	0.85	0.32	0.65	0.98	0.34	0.63	0.98	0.34	0.64	0.99	0.39	0.59	0.99
Harryflintia	-1.21	0.23	0.82	-1.17	0.24	0.91	-1.39	0.18	0.94	-1.54	0.14	0.83	-1.83	0.08	0.84
Holdemania	1.63	0.29	0.82	1.48	0.33	0.91	1.24	0.43	0.98	1.14	0.47	0.95	0.56	0.73	0.99

© 2022 Meyer K et al. *JAMA Network Open.*

Hungatella	-0.86	0.27	0.82	-0.74	0.34	0.91	-0.46	0.57	0.98	-0.46	0.57	0.98	-0.23	0.77	0.99
Hydrogenoanaerobacterium	1.01	0.22	0.82	0.68	0.40	0.91	0.87	0.32	0.94	0.72	0.41	0.95	0.67	0.45	0.96
Incertae_Sedis	-1.10	0.41	0.85	-1.08	0.41	0.91	-0.78	0.57	0.98	-0.85	0.53	0.97	-0.97	0.48	0.96
Intestinibacter	-0.47	0.58	0.87	-0.32	0.70	0.99	-0.39	0.64	0.98	-0.28	0.74	0.99	-0.17	0.84	0.99
Intestinimonas	0.19	0.79	0.95	0.02	0.97	0.99	0.14	0.85	0.98	-0.02	0.98	0.99	0.16	0.83	0.99
Lachnoclostridium	-2.21	0.16	0.80	-1.58	0.31	0.91	-0.86	0.60	0.98	-0.75	0.65	0.99	-0.59	0.72	0.99
Lachnospira	0.61	0.42	0.85	0.18	0.81	0.99	0.03	0.97	1.00	0.11	0.89	0.99	0.07	0.93	0.99
Lachnospiraceae_FCS020_group	2.28	0.01	0.70	1.88	0.03	0.60	1.69	0.06	0.94	1.71	0.05	0.83	1.58	0.08	0.84
Lachnospiraceae_ND3007_group	0.35	0.52	0.86	0.18	0.74	0.99	0.01	0.99	1.00	0.08	0.88	0.99	0.01	0.99	0.99
Lachnospiraceae_NK4A136_group	0.07	0.93	0.98	-0.28	0.73	0.99	-0.71	0.42	0.98	-0.64	0.46	0.95	-0.70	0.43	0.96
Lachnospiraceae_UCG.001	-0.03	0.96	0.98	-0.13	0.83	0.99	-0.18	0.77	0.98	-0.26	0.68	0.99	-0.24	0.70	0.99
Lachnospiraceae_UCG.004	0.52	0.40	0.85	0.47	0.44	0.91	0.62	0.33	0.94	0.67	0.29	0.94	0.46	0.47	0.96
Lachnospiraceae_UCG.010	0.48	0.60	0.89	0.21	0.82	0.99	-0.08	0.94	0.99	-0.12	0.90	0.99	-0.08	0.94	0.99
Lactobacillus	0.05	0.95	0.98	0.42	0.56	0.92	0.46	0.55	0.98	0.64	0.42	0.95	0.79	0.32	0.96
Lactococcus	-0.09	0.93	0.98	-0.10	0.92	0.99	0.06	0.95	0.99	0.02	0.98	0.99	-0.16	0.89	0.99
Marvinbryantia	-0.58	0.34	0.82	-0.68	0.26	0.91	-0.87	0.17	0.94	-0.87	0.17	0.83	-0.80	0.21	0.96
Megasphaera	-0.28	0.63	0.90	-0.01	0.98	0.99	0.37	0.54	0.98	0.40	0.52	0.95	0.36	0.57	0.99
Monoglobus	0.92	0.27	0.82	0.76	0.35	0.91	0.84	0.33	0.94	0.81	0.34	0.94	0.73	0.40	0.96
Moryella	1.01	0.34	0.82	0.70	0.51	0.91	0.30	0.79	0.98	0.40	0.73	0.99	0.40	0.73	0.99
Negativibacillus	0.93	0.15	0.80	1.10	0.08	0.71	0.93	0.16	0.94	0.90	0.17	0.83	0.83	0.22	0.96
NK4A214_group	-0.12	0.82	0.95	-0.20	0.69	0.99	-0.40	0.46	0.98	-0.45	0.40	0.95	-0.43	0.43	0.96
Odoribacter	1.20	0.01	0.71	1.11	0.02	0.60	0.90	0.08	0.94	0.89	0.08	0.83	0.78	0.13	0.96
Oscillibacter	-0.42	0.62	0.90	-0.61	0.47	0.91	-0.45	0.61	0.98	-0.60	0.50	0.95	-0.75	0.40	0.96
Oscillospira	-0.15	0.90	0.98	-0.08	0.94	0.99	-0.20	0.87	0.98	-0.23	0.85	0.99	-0.20	0.87	0.99
Oxalobacter	-0.56	0.52	0.86	-1.08	0.21	0.91	-1.39	0.13	0.94	-1.41	0.13	0.83	-1.40	0.13	0.96
Paludicola	0.02	0.98	0.98	-0.20	0.82	0.99	-0.70	0.45	0.98	-0.88	0.34	0.94	-0.70	0.46	0.96
Parabacteroides	1.01	0.11	0.80	1.15	0.07	0.69	1.03	0.11	0.94	1.18	0.07	0.83	1.25	0.05	0.84
Paraprevotella	0.49	0.32	0.82	0.56	0.25	0.91	0.46	0.37	0.95	0.45	0.38	0.95	0.45	0.37	0.96
Parasutterella	0.70	0.14	0.80	0.62	0.18	0.91	0.49	0.32	0.94	0.51	0.30	0.94	0.34	0.49	0.96
Peptococcus	1.34	0.18	0.81	1.10	0.27	0.91	0.24	0.82	0.98	0.18	0.86	0.99	0.35	0.74	0.99
Phascolarctobacterium	0.82	0.07	0.74	0.89	0.05	0.63	0.95	0.04	0.94	0.91	0.05	0.83	0.93	0.05	0.84
Phocea	0.24	0.83	0.95	0.19	0.86	0.99	0.37	0.75	0.98	0.47	0.68	0.99	0.46	0.69	0.99
Prevotella	0.12	0.80	0.95	0.34	0.46	0.91	0.21	0.66	0.98	0.23	0.62	0.99	0.24	0.60	0.99
Romboutsia	-0.03	0.97	0.98	0.07	0.93	0.99	0.06	0.93	0.99	0.13	0.87	0.99	0.06	0.94	0.99
Roseburia	-0.27	0.75	0.95	-0.25	0.75	0.99	-0.34	0.69	0.98	-0.26	0.76	0.99	-0.14	0.87	0.99
Ruminococcus	0.49	0.38	0.85	0.22	0.69	0.99	0.09	0.87	0.98	0.08	0.89	0.99	0.01	0.99	0.99
Sellimonas	1.18	0.15	0.80	0.87	0.28	0.91	0.58	0.49	0.98	0.46	0.58	0.98	0.48	0.57	0.99
Shuttleworthia	-0.23	0.76	0.95	-0.80	0.30	0.91	-1.02	0.21	0.94	-1.13	0.16	0.83	-1.03	0.21	0.96
Slackia	0.73	0.29	0.82	0.81	0.24	0.91	0.75	0.29	0.94	0.84	0.24	0.94	0.67	0.35	0.96
Streptococcus	-1.63	0.09	0.78	-1.57	0.10	0.73	-0.80	0.43	0.98	-0.76	0.45	0.95	-0.72	0.48	0.96
Subdoligranulum	1.12	0.11	0.80	0.93	0.18	0.91	0.24	0.74	0.98	0.27	0.72	0.99	0.21	0.78	0.99
Sutterella	-0.13	0.75	0.95	0.09	0.84	0.99	-0.11	0.80	0.98	-0.10	0.83	0.99	-0.07	0.87	0.99
TM7x	-1.05	0.28	0.82	-1.31	0.17	0.91	-0.95	0.34	0.95	-0.93	0.35	0.94	-0.89	0.37	0.96
Turicibacter	0.70	0.53	0.86	0.80	0.46	0.91	1.09	0.33	0.94	1.06	0.34	0.94	0.73	0.52	0.96
Tuzzerella	-0.12	0.86	0.96	-0.01	0.99	0.99	-0.20	0.78	0.98	-0.27	0.70	0.99	-0.43	0.55	0.99
UBA1819	0.73	0.48	0.85	0.70	0.49	0.91	0.93	0.38	0.95	0.74	0.49	0.95	0.77	0.48	0.96
UCG.002	0.66	0.17	0.81	0.53	0.27	0.91	0.11	0.84	0.98	0.07	0.90	0.99	0.02	0.97	0.99
UCG.003	1.04	0.07	0.74	0.89	0.12	0.81	0.72	0.23	0.94	0.74	0.22	0.94	0.62	0.31	0.96
UCG.005	0.75	0.24	0.82	0.55	0.39	0.91	0.28	0.68	0.98	0.21	0.76	0.99	0.09	0.89	0.99
UCG.009	-0.28	0.78	0.95	-0.76	0.44	0.91	-1.31	0.20	0.94	-1.36	0.19	0.83	-1.35	0.19	0.96
Veillonella	-0.53	0.47	0.85	-0.58	0.43	0.91	-0.12	0.88	0.98	-0.04	0.95	0.99	0.03	0.97	0.99

Multivariable-adjusted regression models adjusted for:
Model 1: sequencing batch, age, race, gender, study center
Model 2: Model 1, with education
Model 3: Model 2, with physical activity, diet score, medication use, and smoking
Model 4: Model 3 with BMI

© 2022 Meyer K et al. *JAMA Network Open.*

Model 5: Model 4 with hypertension and diabetes

© 2022 Meyer K et al. *JAMA Network Open.*

eTable 59: Multivariable-adjusted regression coefficients, p-values, and false discovery rate (FDR)-adjusted p-values for genera among female participants with respect to MOCA.

	Model1			Model2			Model3			Model4			Model5		
	beta	p-value	FDR	beta	p-value	FDR	beta	p-value	FDR	beta	p-value	FDR	beta	p-value	FDR
Acetanaerobacterium	-0.17	0.63	0.89	0.09	0.79	0.92	0.14	0.69	0.89	0.12	0.73	0.92	0.13	0.71	0.93
Acidaminococcus	-0.31	0.01	0.15	-0.20	0.09	0.50	-0.11	0.37	0.65	-0.11	0.38	0.67	-0.11	0.37	0.67
Actinomyces	-0.07	0.83	0.94	0.07	0.82	0.92	0.27	0.39	0.67	0.28	0.37	0.67	0.40	0.21	0.58
Adlercreutzia	0.03	0.88	0.95	-0.07	0.69	0.92	-0.14	0.47	0.72	-0.16	0.43	0.71	-0.15	0.44	0.72
Agathobacter	-0.34	0.14	0.41	-0.30	0.16	0.63	-0.36	0.10	0.49	-0.35	0.12	0.53	-0.32	0.16	0.50
Akkermansia	0.30	0.01	0.15	0.25	0.03	0.37	0.26	0.03	0.36	0.25	0.03	0.38	0.24	0.04	0.40
Alistipes	0.16	0.43	0.71	0.16	0.38	0.70	0.13	0.50	0.74	0.12	0.51	0.75	0.11	0.55	0.81
Anaerofilum	0.36	0.18	0.47	0.05	0.86	0.94	0.06	0.83	0.96	0.04	0.87	0.98	0.09	0.74	0.94
Anaerostipes	0.12	0.68	0.89	0.06	0.82	0.92	0.01	0.98	0.98	0.00	0.99	0.99	0.05	0.84	0.99
Anaerotruncus	0.61	0.01	0.15	0.56	0.02	0.37	0.66	0.01	0.23	0.64	0.01	0.30	0.64	0.01	0.32
Angelakisella	0.43	0.24	0.52	0.23	0.50	0.77	0.44	0.22	0.60	0.42	0.25	0.64	0.41	0.26	0.62
Bacteroides	0.13	0.79	0.93	-0.04	0.93	0.94	-0.13	0.78	0.94	-0.11	0.82	0.95	-0.03	0.94	1.00
Barnesiella	0.14	0.22	0.50	0.13	0.23	0.70	0.18	0.11	0.49	0.18	0.11	0.53	0.21	0.07	0.41
Bifidobacterium	-0.10	0.54	0.83	0.03	0.82	0.92	0.02	0.88	0.98	0.02	0.89	0.98	0.01	0.95	1.00
Bilophila	0.20	0.20	0.48	0.21	0.15	0.63	0.20	0.17	0.57	0.20	0.18	0.58	0.22	0.14	0.50
Blautia	0.10	0.86	0.94	0.14	0.78	0.92	0.29	0.57	0.80	0.27	0.59	0.83	0.58	0.28	0.62
Butyricicoccus	0.14	0.69	0.89	-0.12	0.72	0.92	-0.33	0.34	0.63	-0.32	0.36	0.67	-0.32	0.35	0.65
Butyricimonas	0.29	0.03	0.17	0.23	0.06	0.41	0.26	0.04	0.36	0.27	0.04	0.38	0.24	0.07	0.41
CAG.56	-0.10	0.52	0.83	-0.14	0.34	0.70	-0.12	0.44	0.71	-0.12	0.45	0.73	-0.08	0.63	0.86
Candidatus_Soleaferrea	0.30	0.37	0.66	0.28	0.38	0.70	0.45	0.16	0.56	0.44	0.16	0.56	0.50	0.12	0.43
Caproiciproducens	0.24	0.36	0.66	0.29	0.24	0.70	0.24	0.34	0.63	0.23	0.36	0.67	0.24	0.34	0.65
Christensenellaceae_R.7_group	0.30	0.02	0.17	0.21	0.10	0.50	0.21	0.12	0.49	0.20	0.14	0.53	0.23	0.10	0.42
Clostridium_sensu_stricto_1	0.00	1.00	1.00	-0.02	0.88	0.94	-0.07	0.68	0.89	-0.07	0.68	0.90	-0.07	0.69	0.92
Colidextribacter	-0.14	0.68	0.89	-0.18	0.58	0.81	-0.10	0.76	0.94	-0.13	0.71	0.92	0.00	1.00	1.00
Collinsella	-0.12	0.42	0.71	0.09	0.52	0.77	0.09	0.52	0.75	0.10	0.47	0.73	0.11	0.46	0.72
Coprobacter	0.05	0.78	0.93	-0.05	0.74	0.92	-0.08	0.64	0.87	-0.08	0.62	0.85	-0.05	0.78	0.98
Coprococcus	-0.30	0.08	0.29	-0.20	0.21	0.66	-0.18	0.27	0.63	-0.17	0.28	0.67	-0.15	0.36	0.65
Defluviitaleaceae_UCG.011	0.54	0.03	0.17	0.40	0.09	0.50	0.42	0.08	0.42	0.41	0.10	0.49	0.35	0.16	0.50
Desulfovibrio	-0.13	0.41	0.71	-0.09	0.52	0.77	-0.01	0.94	0.98	-0.02	0.91	0.98	-0.03	0.87	1.00
Dialister	-0.13	0.25	0.53	-0.14	0.20	0.66	-0.17	0.14	0.52	-0.17	0.14	0.53	-0.13	0.24	0.62
DNF00809	0.39	0.17	0.46	0.36	0.18	0.63	0.49	0.08	0.42	0.48	0.08	0.47	0.49	0.07	0.41
Dorea	-0.11	0.60	0.86	0.02	0.92	0.94	0.03	0.87	0.98	0.05	0.83	0.95	0.03	0.90	1.00
DTU089	0.32	0.17	0.46	0.25	0.26	0.70	0.35	0.12	0.49	0.34	0.13	0.53	0.37	0.11	0.42
Eggerthella	-0.05	0.82	0.94	-0.07	0.75	0.92	-0.06	0.77	0.94	-0.07	0.73	0.92	0.00	1.00	1.00
Eisenbergiella	0.40	0.07	0.29	0.38	0.06	0.41	0.38	0.07	0.42	0.36	0.08	0.47	0.39	0.06	0.41
Erysipelatoclostridium	0.07	0.76	0.93	0.05	0.82	0.92	0.02	0.93	0.98	0.00	0.99	0.99	0.05	0.84	0.99
Erysipelotrichaceae_UCG.003	0.09	0.60	0.86	0.08	0.61	0.85	0.04	0.81	0.95	0.04	0.83	0.95	0.04	0.81	0.98
Escherichia.Shigella	-0.09	0.57	0.85	-0.04	0.79	0.92	0.01	0.92	0.98	0.02	0.90	0.98	-0.02	0.90	1.00
Faecalibacterium	0.26	0.23	0.52	0.10	0.63	0.87	-0.02	0.92	0.98	-0.01	0.96	0.99	0.01	0.98	1.00
Family_XIII_AD3011_group	0.28	0.19	0.48	0.21	0.30	0.70	0.26	0.23	0.60	0.25	0.25	0.64	0.24	0.28	0.62
Family_XIII_UCG.001	0.31	0.26	0.53	0.23	0.37	0.70	0.24	0.37	0.65	0.22	0.41	0.69	0.20	0.47	0.72
Flavonifractor	0.15	0.54	0.83	0.14	0.54	0.77	0.23	0.33	0.63	0.23	0.33	0.67	0.30	0.20	0.55
Frisingicoccus	0.44	0.01	0.15	0.31	0.06	0.41	0.23	0.19	0.58	0.21	0.22	0.64	0.25	0.16	0.50
Fusicatenibacter	-0.04	0.79	0.93	-0.12	0.42	0.72	-0.17	0.29	0.63	-0.16	0.33	0.67	-0.16	0.33	0.65
Fusobacterium	-0.63	0.03	0.17	-0.55	0.04	0.37	-0.48	0.08	0.42	-0.50	0.07	0.46	-0.54	0.05	0.41
GCA.900066575	0.40	0.11	0.33	0.22	0.35	0.70	0.18	0.45	0.72	0.17	0.50	0.74	0.21	0.40	0.68
GCA.900066755	-0.20	0.60	0.86	-0.16	0.66	0.89	-0.07	0.86	0.97	-0.10	0.79	0.94	0.04	0.92	1.00
Gordonibacter	0.40	0.09	0.30	0.33	0.14	0.63	0.24	0.30	0.63	0.22	0.35	0.67	0.23	0.31	0.65
Granulicatella	-0.07	0.81	0.94	-0.10	0.74	0.92	-0.10	0.75	0.94	-0.08	0.79	0.94	-0.05	0.88	1.00
Haemophilus	0.09	0.64	0.89	-0.03	0.87	0.94	-0.16	0.39	0.67	-0.16	0.39	0.67	-0.13	0.47	0.72
Harryflintia	0.31	0.25	0.53	0.31	0.21	0.66	0.46	0.08	0.42	0.44	0.09	0.49	0.47	0.08	0.41
Holdemania	0.33	0.43	0.71	0.27	0.49	0.77	0.21	0.60	0.82	0.19	0.63	0.85	0.21	0.60	0.86

© 2022 Meyer K et al. *JAMA Network Open.*

Hungatella	-0.04	0.84	0.94	0.03	0.88	0.94	0.15	0.47	0.72	0.15	0.47	0.73	0.14	0.50	0.76
Hydrogenoanaerobacterium	0.38	0.08	0.29	0.20	0.33	0.70	0.22	0.32	0.63	0.20	0.37	0.67	0.24	0.28	0.62
Incertae_Sedis	0.46	0.20	0.48	0.45	0.17	0.63	0.69	0.05	0.36	0.68	0.05	0.40	0.75	0.03	0.35
Intestinibacter	-0.20	0.36	0.66	-0.13	0.53	0.77	-0.16	0.44	0.71	-0.15	0.49	0.74	-0.08	0.73	0.94
Intestinimonas	0.47	0.02	0.15	0.38	0.04	0.37	0.42	0.03	0.36	0.40	0.04	0.38	0.45	0.02	0.32
Lachnoclostridium	-0.75	0.07	0.29	-0.38	0.34	0.70	-0.31	0.46	0.72	-0.29	0.48	0.74	-0.30	0.47	0.72
Lachnospira	0.29	0.15	0.44	0.06	0.75	0.92	-0.08	0.68	0.89	-0.07	0.72	0.92	-0.02	0.93	1.00
Lachnospiraceae_FCS020_group	0.60	0.01	0.15	0.36	0.09	0.50	0.30	0.18	0.57	0.31	0.17	0.56	0.30	0.18	0.50
Lachnospiraceae_ND3007_grou	-0.06	0.68	0.89	-0.16	0.25	0.70	-0.16	0.27	0.63	-0.15	0.30	0.67	-0.13	0.38	0.67
Lachnospiraceae_NK4A136_gro	-0.02	0.93	0.96	-0.21	0.30	0.70	-0.43	0.05	0.36	-0.42	0.05	0.40	-0.39	0.08	0.41
Lachnospiraceae_UCG.001	0.15	0.34	0.65	0.10	0.50	0.77	-0.02	0.91	0.98	-0.03	0.85	0.96	0.01	0.96	1.00
Lachnospiraceae_UCG.004	0.15	0.37	0.67	0.11	0.47	0.77	0.01	0.95	0.98	0.02	0.92	0.98	-0.01	0.97	1.00
Lachnospiraceae_UCG.010	-0.03	0.92	0.96	-0.17	0.46	0.77	-0.24	0.32	0.63	-0.24	0.31	0.67	-0.24	0.32	0.65
Lactobacillus	-0.34	0.08	0.29	-0.16	0.37	0.70	-0.05	0.80	0.95	-0.03	0.90	0.98	-0.05	0.80	0.98
Lactococcus	0.19	0.50	0.81	0.17	0.51	0.77	0.24	0.37	0.65	0.24	0.38	0.67	0.26	0.35	0.65
Marvinbryantia	0.03	0.86	0.94	-0.01	0.93	0.94	0.05	0.76	0.94	0.05	0.76	0.94	0.09	0.58	0.84
Megasphaera	-0.26	0.09	0.30	-0.13	0.37	0.70	-0.01	0.97	0.98	0.00	0.99	0.99	-0.03	0.87	1.00
Monoglobus	0.50	0.02	0.17	0.42	0.04	0.37	0.45	0.04	0.36	0.44	0.04	0.38	0.51	0.02	0.32
Moryella	0.10	0.73	0.92	-0.09	0.74	0.92	-0.02	0.95	0.98	0.00	0.99	0.99	0.00	1.00	1.00
Negativibacillus	0.04	0.82	0.94	0.14	0.40	0.71	0.26	0.12	0.49	0.25	0.13	0.53	0.30	0.08	0.41
NK4A214_group	0.17	0.21	0.48	0.12	0.33	0.70	0.16	0.23	0.60	0.15	0.26	0.64	0.18	0.17	0.50
Odoribacter	0.18	0.17	0.46	0.11	0.35	0.70	0.13	0.32	0.63	0.13	0.33	0.67	0.11	0.39	0.68
Oscillibacter	0.49	0.03	0.17	0.41	0.06	0.41	0.46	0.03	0.36	0.45	0.04	0.38	0.50	0.02	0.32
Oscillospira	0.13	0.67	0.89	0.19	0.52	0.77	0.37	0.22	0.60	0.36	0.23	0.64	0.50	0.10	0.42
Oxalobacter	0.47	0.04	0.21	0.21	0.34	0.70	0.15	0.52	0.75	0.15	0.53	0.76	0.11	0.63	0.86
Paludicola	0.40	0.09	0.30	0.30	0.18	0.63	0.29	0.21	0.60	0.27	0.24	0.64	0.33	0.17	0.50
Parabacteroides	0.07	0.68	0.89	0.15	0.35	0.70	0.21	0.18	0.57	0.24	0.14	0.53	0.26	0.11	0.42
Paraprevotella	0.01	0.93	0.96	0.04	0.72	0.92	-0.06	0.65	0.87	-0.06	0.64	0.86	-0.03	0.81	0.98
Parasutterella	0.33	0.01	0.15	0.28	0.02	0.37	0.26	0.03	0.36	0.27	0.03	0.38	0.25	0.04	0.40
Peptococcus	0.66	0.01	0.15	0.53	0.04	0.37	0.57	0.03	0.36	0.57	0.03	0.38	0.62	0.02	0.32
Phascolarctobacterium	0.20	0.10	0.31	0.23	0.04	0.37	0.23	0.04	0.36	0.23	0.05	0.40	0.19	0.11	0.42
Phocea	-0.33	0.27	0.54	-0.36	0.20	0.66	-0.39	0.18	0.57	-0.38	0.19	0.60	-0.33	0.26	0.62
Prevotella	-0.22	0.07	0.29	-0.11	0.33	0.70	-0.13	0.27	0.63	-0.12	0.29	0.67	-0.15	0.22	0.58
Romboutsia	-0.22	0.28	0.54	-0.19	0.32	0.70	-0.16	0.42	0.69	-0.15	0.45	0.73	-0.12	0.54	0.80
Roseburia	0.00	1.00	1.00	0.02	0.93	0.94	0.04	0.84	0.96	0.06	0.79	0.94	0.10	0.66	0.89
Ruminococcus	0.25	0.08	0.29	0.12	0.40	0.71	0.14	0.32	0.63	0.14	0.33	0.67	0.17	0.25	0.62
Sellimonas	0.04	0.85	0.94	-0.14	0.49	0.77	-0.23	0.28	0.63	-0.25	0.24	0.64	-0.21	0.33	0.65
Shuttleworthia	0.59	0.00	0.15	0.31	0.10	0.51	0.30	0.14	0.52	0.29	0.15	0.55	0.35	0.09	0.42
Slackia	-0.02	0.89	0.95	0.00	1.00	1.00	0.04	0.80	0.95	0.06	0.75	0.93	0.04	0.81	0.98
Streptococcus	0.20	0.44	0.72	0.21	0.38	0.70	0.30	0.23	0.60	0.31	0.22	0.64	0.35	0.17	0.50
Subdoligranulum	0.42	0.02	0.17	0.33	0.06	0.41	0.38	0.04	0.36	0.38	0.04	0.38	0.42	0.02	0.32
Sutterella	-0.38	0.00	0.07	-0.28	0.01	0.37	-0.34	0.00	0.18	-0.34	0.00	0.21	-0.36	0.00	0.11
TM7x	0.16	0.54	0.83	0.03	0.90	0.94	-0.01	0.98	0.98	0.00	0.99	0.99	0.09	0.72	0.93
Turicibacter	0.00	0.99	1.00	0.04	0.87	0.94	0.27	0.34	0.63	0.26	0.35	0.67	0.22	0.44	0.72
Tuzzerella	0.06	0.74	0.92	0.14	0.41	0.72	0.11	0.54	0.76	0.10	0.58	0.81	0.09	0.61	0.86
UBA1819	0.61	0.03	0.17	0.61	0.02	0.37	0.78	0.00	0.18	0.76	0.00	0.24	0.85	0.00	0.11
UCG.002	0.33	0.01	0.15	0.26	0.03	0.37	0.21	0.12	0.49	0.20	0.13	0.53	0.22	0.10	0.42
UCG.003	0.05	0.76	0.93	-0.04	0.79	0.92	-0.10	0.51	0.75	-0.10	0.52	0.76	-0.12	0.45	0.72
UCG.005	0.34	0.04	0.21	0.22	0.16	0.63	0.18	0.29	0.63	0.17	0.32	0.67	0.18	0.30	0.65
UCG.009	0.48	0.07	0.29	0.22	0.38	0.70	0.27	0.29	0.63	0.26	0.31	0.67	0.29	0.27	0.62
Veillonella	-0.22	0.27	0.54	-0.26	0.15	0.63	-0.36	0.06	0.42	-0.35	0.07	0.46	-0.36	0.07	0.41

Multivariable-adjusted regression models adjusted for:
Model 1: sequencing batch, age, race, gender, study center
Model 2: Model 1, with education
Model 3: Model 2, with physical activity, diet score, medication use, and smoking
Model 4: Model 3 with BMI

© 2022 Meyer K et al. *JAMA Network Open.*

Model 5: Model 4 with hypertension and diabetes

© 2022 Meyer K et al. *JAMA Network Open.*

eTable 60: Multivariable-adjusted regression coefficients, p-values, and false discovery rate (FDR)-adjusted p-values for genera among female participants with respect to PC (1st).

	Model1			Model2			Model3			Model4			Model5		
	beta	p-value	FDR	beta	p-value	FDR	beta	p-value	FDR	beta	p-value	FDR	beta	p-value	FDR
Acetanaerobacterium	-0.04	0.82	0.91	0.08	0.62	0.84	0.08	0.64	0.91	0.05	0.76	0.99	0.04	0.83	0.94
Acidaminococcus	-0.16	0.01	0.08	-0.11	0.05	0.29	-0.07	0.23	0.74	-0.07	0.23	0.83	-0.08	0.18	0.80
Actinomyces	-0.16	0.30	0.62	-0.10	0.49	0.76	0.00	1.00	1.00	0.02	0.91	0.99	0.05	0.73	0.94
Adlercreutzia	0.13	0.18	0.43	0.08	0.36	0.69	0.05	0.60	0.91	0.03	0.74	0.99	0.02	0.84	0.94
Agathobacter	-0.11	0.32	0.62	-0.10	0.36	0.69	-0.14	0.20	0.74	-0.12	0.28	0.83	-0.10	0.36	0.87
Akkermansia	0.16	0.01	0.08	0.13	0.01	0.20	0.13	0.02	0.51	0.12	0.03	0.59	0.12	0.04	0.64
Alistipes	0.09	0.34	0.63	0.09	0.30	0.69	0.05	0.63	0.91	0.04	0.67	0.99	0.03	0.73	0.94
Anaerofilum	0.33	0.01	0.09	0.19	0.12	0.44	0.19	0.15	0.74	0.17	0.20	0.83	0.19	0.15	0.80
Anaerostipes	-0.07	0.60	0.81	-0.10	0.45	0.76	-0.11	0.39	0.76	-0.12	0.37	0.83	-0.12	0.37	0.87
Anaerotruncus	0.25	0.04	0.16	0.22	0.05	0.29	0.21	0.08	0.58	0.17	0.15	0.83	0.16	0.18	0.80
Angelakisella	0.24	0.16	0.40	0.15	0.35	0.69	0.22	0.21	0.74	0.19	0.27	0.83	0.18	0.31	0.87
Bacteroides	-0.08	0.75	0.88	-0.15	0.49	0.76	-0.15	0.51	0.86	-0.12	0.60	0.96	-0.09	0.70	0.94
Barnesiella	0.18	0.00	0.04	0.17	0.00	0.10	0.18	0.00	0.13	0.18	0.00	0.13	0.20	0.00	0.05
Bifidobacterium	-0.04	0.57	0.79	0.01	0.85	0.95	-0.01	0.89	1.00	-0.01	0.88	0.99	-0.02	0.83	0.94
Bilophila	0.12	0.10	0.27	0.13	0.07	0.34	0.10	0.16	0.74	0.10	0.18	0.83	0.09	0.23	0.87
Blautia	-0.09	0.74	0.88	-0.07	0.78	0.95	0.01	0.98	1.00	-0.01	0.96	0.99	0.04	0.87	0.94
Butyricicoccus	0.13	0.44	0.72	0.02	0.92	0.95	-0.08	0.63	0.91	-0.06	0.71	0.99	-0.08	0.66	0.94
Butyricimonas	0.14	0.03	0.13	0.12	0.06	0.30	0.11	0.08	0.58	0.12	0.06	0.67	0.12	0.07	0.80
CAG.56	0.03	0.66	0.84	0.02	0.83	0.95	-0.01	0.94	1.00	0.00	0.97	0.99	0.02	0.82	0.94
Candidatus_Soleaferrea	0.12	0.45	0.72	0.11	0.47	0.76	0.16	0.31	0.74	0.15	0.33	0.83	0.15	0.34	0.87
Caproiciproducens	0.16	0.21	0.48	0.18	0.13	0.45	0.11	0.38	0.75	0.10	0.45	0.87	0.07	0.56	0.92
Christensenellaceae_R.7_group	0.20	0.00	0.04	0.16	0.01	0.15	0.12	0.07	0.58	0.11	0.11	0.74	0.11	0.09	0.80
Clostridium_sensu_stricto_1	0.03	0.70	0.85	0.02	0.78	0.95	0.01	0.95	1.00	0.01	0.95	0.99	-0.01	0.93	0.96
Colidextribacter	-0.02	0.92	0.96	-0.03	0.84	0.95	-0.04	0.83	1.00	-0.07	0.69	0.99	-0.01	0.93	0.96
Collinsella	-0.10	0.16	0.40	-0.01	0.90	0.95	0.00	0.98	1.00	0.01	0.86	0.99	0.01	0.84	0.94
Coprobacter	0.02	0.81	0.90	-0.03	0.74	0.93	-0.07	0.36	0.74	-0.08	0.32	0.83	-0.07	0.38	0.87
Coprococcus	-0.05	0.52	0.74	-0.01	0.90	0.95	-0.01	0.86	1.00	-0.01	0.92	0.99	0.01	0.87	0.94
Defluviitaleaceae_UCG.011	0.34	0.01	0.08	0.27	0.02	0.22	0.23	0.06	0.58	0.20	0.10	0.74	0.17	0.17	0.80
Desulfovibrio	-0.02	0.78	0.88	-0.01	0.93	0.95	-0.01	0.87	1.00	-0.02	0.81	0.99	-0.02	0.81	0.94
Dialister	-0.05	0.41	0.69	-0.05	0.36	0.69	-0.05	0.37	0.74	-0.05	0.37	0.83	-0.04	0.51	0.92
DNF00809	0.09	0.49	0.74	0.08	0.54	0.80	0.07	0.59	0.91	0.07	0.62	0.96	0.06	0.63	0.94
Dorea	-0.11	0.31	0.62	-0.05	0.64	0.84	-0.07	0.51	0.86	-0.05	0.61	0.96	-0.06	0.54	0.92
DTU089	0.05	0.67	0.84	0.02	0.88	0.95	0.02	0.88	1.00	0.01	0.94	0.99	0.03	0.78	0.94
Eggerthella	-0.01	0.89	0.94	-0.02	0.83	0.95	0.00	0.97	1.00	-0.02	0.85	0.99	0.01	0.93	0.96
Eisenbergiella	0.11	0.28	0.59	0.10	0.29	0.69	0.09	0.35	0.74	0.06	0.53	0.96	0.08	0.46	0.92
Erysipelatoclostridium	0.02	0.83	0.91	0.02	0.89	0.95	0.01	0.91	1.00	-0.01	0.94	0.99	0.00	0.98	0.99
Erysipelotrichaceae_UCG.003	0.07	0.37	0.63	0.07	0.36	0.69	0.03	0.70	0.94	0.03	0.76	0.99	0.02	0.82	0.94
Escherichia.Shigella	-0.04	0.58	0.79	-0.02	0.79	0.95	0.01	0.85	1.00	0.02	0.80	0.99	0.02	0.84	0.94
Faecalibacterium	0.16	0.11	0.31	0.09	0.34	0.69	0.04	0.66	0.92	0.06	0.57	0.96	0.06	0.54	0.92
Family_XIII_AD3011_group	0.18	0.09	0.26	0.14	0.14	0.46	0.10	0.33	0.74	0.09	0.41	0.83	0.07	0.50	0.92
Family_XIII_UCG.001	0.24	0.07	0.23	0.20	0.10	0.42	0.17	0.21	0.74	0.14	0.27	0.83	0.13	0.34	0.87
Flavonifractor	-0.10	0.37	0.63	-0.11	0.32	0.69	-0.08	0.48	0.86	-0.08	0.47	0.89	-0.07	0.53	0.92
Frisingicoccus	0.15	0.09	0.26	0.09	0.28	0.69	0.04	0.63	0.91	0.02	0.81	0.99	0.03	0.71	0.94
Fusicatenibacter	0.04	0.64	0.84	0.00	0.98	0.99	-0.02	0.80	1.00	0.00	0.99	1.00	-0.02	0.84	0.94
Fusobacterium	-0.16	0.25	0.55	-0.12	0.35	0.69	-0.03	0.84	1.00	-0.05	0.74	0.99	-0.05	0.71	0.94
GCA.900066575	0.17	0.16	0.40	0.09	0.43	0.76	0.02	0.88	1.00	-0.01	0.96	0.99	0.00	0.97	0.99
GCA.900066755	0.06	0.76	0.88	0.08	0.66	0.86	0.10	0.60	0.91	0.05	0.77	0.99	0.10	0.58	0.94
Gordonibacter	0.24	0.03	0.14	0.21	0.05	0.29	0.14	0.20	0.74	0.12	0.31	0.83	0.11	0.33	0.87
Granulicatella	0.01	0.94	0.96	0.00	0.99	0.99	0.00	0.99	1.00	0.02	0.90	0.99	0.06	0.71	0.94
Haemophilus	0.06	0.50	0.74	0.01	0.91	0.95	-0.05	0.60	0.91	-0.05	0.59	0.96	-0.03	0.77	0.94
Harryflintia	0.13	0.32	0.62	0.13	0.28	0.69	0.16	0.23	0.74	0.13	0.31	0.83	0.14	0.30	0.87
Holdemania	0.40	0.04	0.17	0.37	0.05	0.29	0.35	0.08	0.58	0.33	0.09	0.74	0.30	0.12	0.80

© 2022 Meyer K et al. *JAMA Network Open.*

Hungatella	-0.08	0.43	0.71	-0.05	0.62	0.84	0.03	0.78	1.00	0.03	0.79	0.99	0.04	0.71	0.94
Hydrogenoanaerobacterium	0.24	0.03	0.13	0.16	0.12	0.44	0.15	0.18	0.74	0.12	0.29	0.83	0.12	0.28	0.87
Incertae_Sedis	0.17	0.33	0.62	0.17	0.31	0.69	0.19	0.28	0.74	0.17	0.31	0.83	0.18	0.29	0.87
Intestinibacter	0.00	1.00	1.00	0.03	0.74	0.93	0.02	0.86	1.00	0.04	0.70	0.99	0.07	0.53	0.92
Intestinimonas	0.29	0.00	0.04	0.25	0.00	0.12	0.24	0.01	0.36	0.21	0.02	0.59	0.24	0.01	0.39
Lachnoclostridium	-0.50	0.01	0.09	-0.34	0.08	0.35	-0.21	0.31	0.74	-0.19	0.36	0.83	-0.19	0.35	0.87
Lachnospira	0.24	0.01	0.09	0.15	0.12	0.44	0.10	0.32	0.74	0.11	0.25	0.83	0.13	0.18	0.80
Lachnospiraceae_FCS020_group	0.39	0.00	0.03	0.29	0.01	0.12	0.24	0.03	0.55	0.24	0.03	0.59	0.22	0.04	0.65
Lachnospiraceae_ND3007_group	0.01	0.88	0.94	-0.03	0.62	0.84	-0.07	0.31	0.74	-0.06	0.42	0.83	-0.05	0.48	0.92
Lachnospiraceae_NK4A136_grou	0.01	0.89	0.94	-0.07	0.48	0.76	-0.18	0.09	0.58	-0.17	0.12	0.74	-0.16	0.14	0.80
Lachnospiraceae_UCG.001	0.09	0.25	0.55	0.07	0.37	0.69	0.03	0.67	0.92	0.02	0.80	0.99	0.03	0.66	0.94
Lachnospiraceae_UCG.004	0.04	0.61	0.81	0.02	0.74	0.93	0.00	0.97	1.00	0.01	0.94	0.99	-0.01	0.91	0.96
Lachnospiraceae_UCG.010	-0.03	0.77	0.88	-0.10	0.37	0.69	-0.13	0.26	0.74	-0.14	0.23	0.83	-0.13	0.27	0.87
Lactobacillus	-0.17	0.07	0.23	-0.09	0.30	0.69	-0.09	0.33	0.74	-0.06	0.51	0.93	-0.06	0.55	0.92
Lactococcus	0.09	0.49	0.74	0.09	0.49	0.76	0.08	0.54	0.89	0.07	0.58	0.96	0.07	0.62	0.94
Marvinbryantia	-0.06	0.46	0.73	-0.08	0.29	0.69	-0.07	0.35	0.74	-0.07	0.35	0.83	-0.05	0.53	0.92
Megasphaera	-0.07	0.35	0.63	-0.01	0.87	0.95	0.06	0.46	0.85	0.06	0.42	0.83	0.05	0.50	0.92
Monoglobus	0.33	0.00	0.04	0.30	0.00	0.12	0.28	0.01	0.36	0.28	0.01	0.42	0.30	0.01	0.27
Moryella	0.18	0.20	0.46	0.10	0.46	0.76	0.06	0.69	0.93	0.07	0.60	0.96	0.09	0.53	0.92
Negativibacillus	0.04	0.64	0.84	0.08	0.30	0.69	0.11	0.18	0.74	0.10	0.20	0.83	0.11	0.19	0.80
NK4A214_group	0.12	0.06	0.22	0.10	0.10	0.43	0.08	0.21	0.74	0.07	0.26	0.83	0.09	0.17	0.80
Odoribacter	0.12	0.06	0.22	0.09	0.14	0.46	0.07	0.29	0.74	0.06	0.30	0.83	0.06	0.37	0.87
Oscillibacter	0.25	0.02	0.12	0.21	0.04	0.29	0.20	0.06	0.58	0.18	0.10	0.74	0.17	0.11	0.80
Oscillospira	0.06	0.68	0.84	0.09	0.54	0.80	0.11	0.45	0.84	0.11	0.47	0.89	0.15	0.31	0.87
Oxalobacter	0.17	0.11	0.31	0.06	0.58	0.84	0.00	0.98	1.00	-0.01	0.95	0.99	-0.03	0.81	0.94
Paludicola	0.20	0.09	0.26	0.15	0.16	0.50	0.07	0.52	0.86	0.04	0.72	0.99	0.06	0.63	0.94
Parabacteroides	0.04	0.65	0.84	0.07	0.35	0.69	0.09	0.25	0.74	0.12	0.14	0.83	0.14	0.09	0.80
Paraprevotella	-0.06	0.37	0.63	-0.04	0.46	0.76	-0.06	0.37	0.74	-0.06	0.34	0.83	-0.05	0.42	0.91
Parasutterella	0.14	0.02	0.11	0.12	0.03	0.29	0.10	0.09	0.58	0.11	0.08	0.74	0.09	0.15	0.80
Peptococcus	0.30	0.02	0.11	0.24	0.05	0.29	0.19	0.14	0.74	0.18	0.16	0.83	0.21	0.11	0.80
Phascolarctobacterium	0.09	0.13	0.35	0.10	0.06	0.33	0.10	0.07	0.58	0.10	0.09	0.74	0.08	0.15	0.80
Phocea	-0.09	0.52	0.74	-0.10	0.44	0.76	-0.10	0.47	0.85	-0.09	0.55	0.96	-0.06	0.69	0.94
Prevotella	-0.10	0.08	0.26	-0.05	0.34	0.69	-0.05	0.35	0.74	-0.05	0.40	0.83	-0.05	0.37	0.87
Romboutsia	0.01	0.94	0.96	0.02	0.81	0.95	0.01	0.89	1.00	0.03	0.78	0.99	0.04	0.72	0.94
Roseburia	-0.03	0.75	0.88	-0.03	0.79	0.95	-0.04	0.71	0.94	-0.02	0.83	0.99	0.00	1.00	1.00
Ruminococcus	0.16	0.02	0.11	0.10	0.12	0.44	0.09	0.23	0.74	0.08	0.25	0.83	0.09	0.23	0.87
Sellimonas	0.21	0.04	0.16	0.14	0.17	0.51	0.11	0.30	0.74	0.08	0.42	0.83	0.10	0.36	0.87
Shuttleworthia	0.23	0.02	0.10	0.11	0.23	0.68	0.07	0.50	0.86	0.05	0.63	0.97	0.07	0.47	0.92
Slackia	-0.06	0.51	0.74	-0.05	0.56	0.83	-0.04	0.66	0.92	-0.02	0.80	0.99	-0.05	0.60	0.94
Streptococcus	0.05	0.68	0.84	0.06	0.63	0.84	0.09	0.45	0.84	0.10	0.40	0.83	0.13	0.32	0.87
Subdoligranulum	0.27	0.00	0.04	0.23	0.01	0.12	0.18	0.05	0.58	0.19	0.04	0.59	0.20	0.03	0.59
Sutterella	-0.14	0.01	0.09	-0.09	0.08	0.37	-0.11	0.05	0.58	-0.10	0.06	0.67	-0.10	0.06	0.80
TM7x	0.00	0.98	0.99	-0.06	0.61	0.84	-0.07	0.56	0.90	-0.07	0.58	0.96	-0.03	0.81	0.94
Turicibacter	0.10	0.48	0.74	0.12	0.37	0.69	0.16	0.24	0.74	0.16	0.26	0.83	0.12	0.39	0.88
Tuzzerella	-0.02	0.84	0.91	0.02	0.83	0.95	0.00	0.96	1.00	-0.02	0.84	0.99	-0.03	0.73	0.94
UBA1819	0.27	0.04	0.16	0.27	0.03	0.29	0.31	0.02	0.44	0.28	0.04	0.59	0.30	0.03	0.59
UCG.002	0.15	0.02	0.10	0.11	0.05	0.29	0.07	0.29	0.74	0.06	0.35	0.83	0.07	0.29	0.87
UCG.003	0.07	0.32	0.62	0.04	0.61	0.84	0.00	0.96	1.00	0.00	1.00	1.00	-0.01	0.88	0.95
UCG.005	0.20	0.01	0.09	0.15	0.05	0.29	0.09	0.28	0.74	0.08	0.35	0.83	0.07	0.37	0.87
UCG.009	0.12	0.36	0.63	0.00	0.99	0.99	-0.03	0.84	1.00	-0.04	0.78	0.99	-0.02	0.86	0.94
Veillonella	-0.05	0.57	0.79	-0.07	0.41	0.74	-0.10	0.31	0.74	-0.08	0.39	0.83	-0.07	0.49	0.92

Multivariable-adjusted regression models adjusted for:

Model 1: sequencing batch, age, race, gender, study center

Model 2: Model 1, with education

Model 3: Model 2, with physical activity, diet score, medication use, and smoking

Model 4: Model 3 with BMI

© 2022 Meyer K et al. *JAMA Network Open.*

Model 5: Model 4 with hypertension and diabetes

© 2022 Meyer K et al. *JAMA Network Open.*

eTable 61: Descriptive statistics for phyla

Phylum	Min.	25th %	Median	75th %	Max.	Mean	SD	Missing, n
**Actinobacteriota**	51	1284	2401	4384	35951	3643	4202.29	0
**Bacteroidota**	75	42818	54298	69215	135415	56826	20544.51	0
**Cyanobacteria**	0	0	0	33	5629	147.1	473.16	380
**Desulfobacterota**	0	54.5	282	611	3605	446.8	544.63	95
**Firmicutes**	12890	39809	51938	64752	132185	54006	18641.44	0
**Fusobacteriota**	0	0	0	9	12808	241.1	1035.03	396
**Patescibacteria**	0	3	10	26	1826	25.34	83.04	115
**Proteobacteria**	0	1689	3038	5296	26977	4167	4000.37	1
**Verrucomicrobiota**	0	3	148	1230	23786	1242	2639.92	135

© 2022 Meyer K et al. *JAMA Network Open.*

ETable 62: Multivariable-adjusted regression coefficients, p-values, and false discovery rate (FDR)-adjusted p-values for phyla with respect to DSST.

	Model1			Model2			Model3			Model4			Model5		
	**beta**	**p-value**	**FDR**	**beta**	**p-value**	**FDR**	**beta**	**p-value**	**FDR**	**beta**	**p-value**	**FDR**	**beta**	**p-value**	**FDR**
Actinobacteriota	-2.02	0.15	0.41	-0.35	0.80	0.94	-0.35	0.80	0.94	-0.35	0.80	0.95	-0.19	0.90	0.97
Bacteroidota	-1.56	0.66	0.74	-1.37	0.68	0.94	-0.24	0.94	0.94	-0.23	0.95	0.95	-0.27	0.94	0.97
Cyanobacteria	0.74	0.20	0.41	0.20	0.73	0.94	-0.06	0.92	0.94	-0.06	0.92	0.95	-0.02	0.97	0.97
Desulfobacterota	0.11	0.86	0.86	-0.03	0.95	0.95	-0.39	0.52	0.94	-0.39	0.52	0.95	-0.40	0.51	0.97
Firmicutes	18.61	0.00	0.00	13.94	0.01	0.04	10.91	0.05	0.22	10.91	0.05	0.23	11.21	0.05	0.21
Fusobacteriota	-1.15	0.23	0.41	-0.62	0.49	0.94	0.15	0.88	0.94	0.14	0.88	0.95	0.07	0.94	0.97
Patescibacteria	-0.61	0.54	0.71	-1.11	0.24	0.72	-1.03	0.29	0.87	-1.03	0.29	0.87	-0.81	0.41	0.97
Proteobacteria	-0.67	0.55	0.71	-0.22	0.83	0.94	0.19	0.86	0.94	0.19	0.86	0.95	0.04	0.97	0.97
Verrucomicrobiota	1.65	0.00	0.00	1.37	0.00	0.02	1.30	0.00	0.04	1.30	0.01	0.05	1.34	0.00	0.04

Multivariable-adjusted regression models adjusted for:
Model 1: sequencing batch, age, race, gender, study center
Model 2: Model 1, with education
Model 3: Model 2, with physical activity, diet score, medication use, and smoking
Model 4: Model 3 with BMI
Model 5: Model 4 with hypertension and diabetes

© 2022 Meyer K et al. *JAMA Network Open.*

eTable 63: Multivariable-adjusted regression coefficients, p-values, and false discovery rate (FDR)-adjusted p-values for phyla with respect to Stroop.

	Model1			Model2			Model3			Model4			Model5		
	**beta**	**p-value**	**FDR**	**beta**	**p-value**	**FDR**	**beta**	**p-value**	**FDR**	**beta**	**p-value**	**FDR**	**beta**	**p-value**	**FDR**
ctinobacterio	-0.72	0.49	0.55	-1.32	0.21	0.37	-1.57	0.13	0.30	-1.59	0.13	0.29	-1.57	0.14	0.31
Bacteroidota	2.12	0.41	0.52	2.06	0.41	0.53	1.56	0.53	0.68	1.54	0.54	0.69	1.32	0.60	0.77
Cyanobacteria	-0.39	0.36	0.52	-0.22	0.61	0.69	-0.06	0.89	0.89	-0.04	0.92	0.92	-0.09	0.83	0.83
esulfobactero	-0.54	0.22	0.43	-0.48	0.26	0.39	-0.34	0.45	0.67	-0.33	0.45	0.68	-0.38	0.40	0.60
Firmicutes	-10.80	0.01	0.07	-9.26	0.02	0.19	-7.95	0.05	0.30	-7.92	0.06	0.29	-7.61	0.07	0.31
Fusobacteriota	1.56	0.02	0.11	1.38	0.04	0.20	1.17	0.09	0.30	1.19	0.09	0.29	1.14	0.10	0.31
Patescibacteri	-0.28	0.70	0.70	-0.13	0.86	0.86	-0.29	0.68	0.77	-0.29	0.69	0.78	-0.28	0.70	0.79
Proteobacteria	-0.98	0.24	0.43	-1.13	0.17	0.37	-1.24	0.13	0.30	-1.27	0.12	0.29	-1.26	0.13	0.31
rrucomicrobio	-0.68	0.04	0.12	-0.59	0.08	0.23	-0.41	0.23	0.42	-0.41	0.24	0.42	-0.41	0.24	0.44

Multivariable-adjusted regression models adjusted for:
Model 1: sequencing batch, age, race, gender, study center
Model 2: Model 1, with education
Model 3: Model 2, with physical activity, diet score, medication use, and smoking
Model 4: Model 3 with BMI
Model 5: Model 4 with hypertension and diabetes

© 2022 Meyer K et al. *JAMA Network Open.*

eTable 64: Multivariable-adjusted regression coefficients, p-values, and false discovery rate (FDR)-adjusted p-values for phyla with respect to RAVLT

	Model1			Model2			Model3			Model4			Model5		
	**beta**	**p-value**	**FDR**	**beta**	**p-value**	**FDR**	**beta**	**p-value**	**FDR**	**beta**	**p-value**	**FDR**	**beta**	**p-value**	**FDR**
Actinobacteriota	-0.29	0.07	0.24	-0.11	0.48	0.70	-0.08	0.62	0.84	-0.08	0.63	0.84	-0.07	0.66	0.86
Bacteroidota	-0.21	0.60	0.67	-0.19	0.62	0.70	-0.01	0.97	0.97	-0.01	0.98	0.98	0.00	0.99	0.99
Cyanobacteria	0.11	0.11	0.24	0.05	0.47	0.70	0.03	0.65	0.84	0.03	0.69	0.84	0.04	0.61	0.86
Desulfobacterota	0.04	0.55	0.67	0.03	0.70	0.70	0.02	0.74	0.84	0.02	0.75	0.84	0.02	0.77	0.86
Firmicutes	1.95	0.00	0.02	1.43	0.02	0.17	1.19	0.07	0.48	1.18	0.07	0.47	1.20	0.07	0.57
Fusobacteriota	-0.19	0.08	0.24	-0.13	0.21	0.63	-0.04	0.73	0.84	-0.04	0.71	0.84	-0.04	0.71	0.86
Patescibacteria	-0.08	0.46	0.67	-0.14	0.20	0.63	-0.18	0.11	0.48	-0.18	0.10	0.47	-0.18	0.13	0.57
Proteobacteria	0.00	0.99	0.99	0.05	0.70	0.70	0.05	0.69	0.84	0.06	0.67	0.84	0.05	0.73	0.86
Verrucomicrobiota	0.07	0.17	0.30	0.04	0.42	0.70	0.02	0.67	0.84	0.02	0.68	0.84	0.02	0.68	0.86

Multivariable-adjusted regression models adjusted for:
Model 1: sequencing batch, age, race, gender, study center
Model 2: Model 1, with education
Model 3: Model 2, with physical activity, diet score, medication use, and smoking
Model 4: Model 3 with BMI
Model 5: Model 4 with hypertension and diabetes

© 2022 Meyer K et al. *JAMA Network Open.*

eTable 65: Multivariable-adjusted regression coefficients, p-values, and false discovery rate (FDR)-adjusted p-values for phyla with respect to category fluency.

	Model1			Model2			Model3			Model4			Model5		
	beta	p-value	FDR	beta	p-value	FDR	beta	p-value	FDR	beta	p-value	FDR	beta	p-value	FDR
Actinobacteriota	-0.07	0.89	0.89	0.39	0.39	0.61	0.51	0.29	0.61	0.53	0.27	0.57	0.53	0.27	0.60
Bacteroidota	0.88	0.45	0.67	0.93	0.40	0.61	0.96	0.40	0.61	1.01	0.38	0.57	1.10	0.34	0.60
Cyanobacteria	0.49	0.01	0.09	0.35	0.06	0.18	0.38	0.06	0.18	0.35	0.08	0.24	0.36	0.08	0.23
Desulfobacterota	0.46	0.02	0.09	0.42	0.03	0.12	0.42	0.04	0.18	0.42	0.04	0.19	0.40	0.05	0.23
Firmicutes	2.18	0.23	0.51	0.90	0.61	0.72	0.84	0.66	0.74	0.79	0.68	0.76	0.73	0.70	0.79
Fusobacteriota	-0.28	0.36	0.66	-0.14	0.64	0.72	0.01	0.97	0.97	-0.02	0.96	0.96	0.00	1.00	1.00
Patescibacteria	-0.18	0.58	0.74	-0.32	0.32	0.61	-0.28	0.40	0.61	-0.29	0.38	0.57	-0.28	0.40	0.60
Proteobacteria	0.76	0.04	0.12	0.88	0.01	0.12	1.05	0.01	0.05	1.09	0.00	0.03	1.08	0.00	0.04
Verrucomicrobiota	0.03	0.84	0.89	-0.05	0.74	0.74	-0.07	0.65	0.74	-0.08	0.63	0.76	-0.10	0.53	0.69

Multivariable-adjusted regression models adjusted for:
Model 1: sequencing batch, age, race, gender, study center
Model 2: Model 1, with education
Model 3: Model 2, with physical activity, diet score, medication use, and smoking
Model 4: Model 3 with BMI
Model 5: Model 4 with hypertension and diabetes

© 2022 Meyer K et al. *JAMA Network Open.*

eTable 66: Multivariable-adjusted regression coefficients, p-values, and false discovery rate (FDR)-adjusted p-values for phyla with respect to letter fluency.

	Model1			Model2			Model3			Model4			Model5		
	beta	p-value	FDR	beta	p-value	FDR	beta	p-value	FDR	beta	p-value	FDR	beta	p-value	FDR
Actinobacteriota	-1.37	0.23	0.66	-0.32	0.78	0.98	-0.25	0.83	0.91	-0.22	0.85	0.92	-0.18	0.88	0.88
Bacteroidota	2.11	0.45	0.66	2.17	0.43	0.77	1.50	0.59	0.91	1.57	0.57	0.91	1.59	0.57	0.88
Cyanobacteria	1.00	0.03	0.31	0.67	0.15	0.45	0.34	0.49	0.91	0.30	0.54	0.91	0.35	0.49	0.88
Desulfobacterota	0.85	0.08	0.35	0.76	0.11	0.45	0.74	0.13	0.87	0.74	0.14	0.91	0.67	0.18	0.88
Firmicutes	2.97	0.51	0.66	0.10	0.98	0.98	0.50	0.91	0.91	0.44	0.92	0.92	0.79	0.87	0.88
Fusobacteriota	0.51	0.51	0.66	0.79	0.29	0.66	1.02	0.19	0.87	1.00	0.21	0.91	1.00	0.21	0.88
Patescibacteria	-0.83	0.30	0.66	-1.15	0.14	0.45	-0.71	0.38	0.91	-0.73	0.37	0.91	-0.71	0.38	0.88
Proteobacteria	-0.26	0.78	0.87	0.07	0.94	0.98	0.29	0.75	0.91	0.34	0.71	0.91	0.27	0.77	0.88
Verrucomicrobiota	0.06	0.87	0.87	-0.12	0.74	0.98	-0.15	0.69	0.91	-0.16	0.67	0.91	-0.21	0.59	0.88

Multivariable-adjusted regression models adjusted for:
Model 1: sequencing batch, age, race, gender, study center
Model 2: Model 1, with education
Model 3: Model 2, with physical activity, diet score, medication use, and smoking
Model 4: Model 3 with BMI
Model 5: Model 4 with hypertension and diabetes

© 2022 Meyer K et al. *JAMA Network Open.*

eTable 67: Multivariable-adjusted regression coefficients, p-values, and false discovery rate (FDR)-adjusted p-values for phyla with respect to MOCA.

	Model1			Model2			Model3			Model4			Model5		
	beta	p-value	FDR	beta	p-value	FDR	beta	p-value	FDR	beta	p-value	FDR	beta	p-value	FDR
Actinobacteriota	-0.50	0.10	0.27	-0.08	0.78	1.00	0.00	1.00	1.00	0.00	0.99	0.99	-0.01	0.96	0.96
Bacteroidota	0.16	0.84	0.97	0.21	0.77	1.00	0.34	0.64	0.97	0.34	0.63	0.99	0.34	0.63	0.96
Cyanobacteria	0.19	0.13	0.27	0.06	0.63	1.00	0.04	0.76	0.97	0.03	0.78	0.99	0.03	0.82	0.96
Desulfobacterota	0.02	0.89	0.97	-0.02	0.89	1.00	-0.04	0.75	0.97	-0.04	0.75	0.99	-0.04	0.73	0.96
Firmicutes	2.35	0.05	0.27	1.13	0.32	1.00	1.16	0.32	0.97	1.16	0.32	0.99	1.25	0.29	0.96
Fusobacteriota	-0.37	0.07	0.27	-0.24	0.22	1.00	-0.18	0.36	0.97	-0.18	0.35	0.99	-0.19	0.34	0.96
Patescibacteria	0.01	0.97	0.97	-0.12	0.56	1.00	-0.10	0.63	0.97	-0.10	0.62	0.99	-0.08	0.69	0.96
Proteobacteria	-0.11	0.65	0.97	0.00	1.00	1.00	-0.02	0.93	1.00	-0.01	0.95	0.99	-0.04	0.87	0.96
Verrucomicrobiota	0.14	0.15	0.27	0.07	0.46	1.00	0.08	0.41	0.97	0.08	0.41	0.99	0.07	0.50	0.96

Multivariable-adjusted regression models adjusted for:
Model 1: sequencing batch, age, race, gender, study center
Model 2: Model 1, with education
Model 3: Model 2, with physical activity, diet score, medication use, and smoking
Model 4: Model 3 with BMI
Model 5: Model 4 with hypertension and diabetes

© 2022 Meyer K et al. *JAMA Network Open.*

eTable 68: Multivariable-adjusted regression coefficients, p-values, and false discovery rate (FDR)-adjusted p-values for phyla with respect to PC (1st).

	Model1			Model2			Model3			Model4			Model5		
	beta	p-value	FDR	beta	p-value	FDR	beta	p-value	FDR	beta	p-value	FDR	beta	p-value	FDR
Actinobacteriota	-0.25	0.09	0.19	-0.04	0.79	0.89	0.00	0.99	0.99	0.00	0.98	0.98	0.01	0.96	0.96
Bacteroidota	0.02	0.97	0.97	0.04	0.90	0.90	0.12	0.73	0.93	0.13	0.70	0.90	0.14	0.67	0.86
Cyanobacteria	0.14	0.02	0.08	0.07	0.20	0.46	0.05	0.44	0.71	0.04	0.50	0.75	0.05	0.44	0.79
Desulfobacterota	0.08	0.20	0.30	0.06	0.29	0.50	0.04	0.47	0.71	0.04	0.48	0.75	0.04	0.53	0.80
Firmicutes	1.60	0.00	0.04	1.00	0.06	0.46	0.81	0.14	0.66	0.80	0.15	0.59	0.82	0.14	0.72
Fusobacteriota	-0.15	0.11	0.20	-0.09	0.33	0.50	-0.01	0.89	0.99	-0.02	0.85	0.95	-0.02	0.85	0.96
Patescibacteria	-0.07	0.51	0.65	-0.13	0.16	0.46	-0.12	0.22	0.66	-0.12	0.21	0.59	-0.11	0.25	0.72
Proteobacteria	0.03	0.79	0.88	0.09	0.41	0.52	0.11	0.29	0.66	0.12	0.26	0.59	0.11	0.32	0.72
Verrucomicrobiota	0.10	0.03	0.08	0.07	0.12	0.46	0.06	0.21	0.66	0.06	0.22	0.59	0.05	0.25	0.72

Multivariable-adjusted regression models adjusted for:
Model 1: sequencing batch, age, race, gender, study center
Model 2: Model 1, with education
Model 3: Model 2, with physical activity, diet score, medication use, and smoking
Model 4: Model 3 with BMI
Model 5: Model 4 with hypertension and diabetes

© 2022 Meyer K et al. *JAMA Network Open.*

eTable 69: Coefficients and p-values for associations between Bacteroidota:Firmicutes ratio with measures of cognitive function.

	**DSST**		**Stroop**		**RAVLT**		**Category fluency**		**Letter fluency**		**MOCA**		**PC (1st)**	
	beta	p-value	beta	p-value	beta	p-value	beta	p-value	beta	p-value	beta	p-value	beta	p-value
**Model 1**	11.15	0.06	8.14	0.07	1.27	0.08	3.66	0.92	9.02	0.85	2.43	0.43	1.14	0.19
**Model 2**	10.65	0.13	8.08	0.10	1.22	0.16	3.56	0.79	8.80	0.61	2.27	0.77	1.05	0.41
**Model 3**	10.98	0.32	8.15	0.19	1.28	0.39	3.76	0.76	9.12	0.75	2.31	0.86	1.10	0.64
**Model 4**	11.00	0.33	8.16	0.19	1.28	0.40	3.76	0.74	9.13	0.73	2.31	0.87	1.10	0.66
**Model 5**	11.06	0.31	8.22	0.23	1.29	0.41	3.77	0.69	9.14	0.75	2.32	0.85	1.10	0.68

Multivariable-adjusted regression models adjusted for:
Model 1: sequencing batch, age, race, gender, study center
Model 2: Model 1, with education
Model 3: Model 2, with physical activity, diet score, medication use, and smoking
Model 4: Model 3 with BMI
Model 5: Model 4 with hypertension and diabetes

© 2022 Meyer K et al. *JAMA Network Open.*

eFigure 1: Beta-diversity (PCoA) differences for each cognitive function assessment

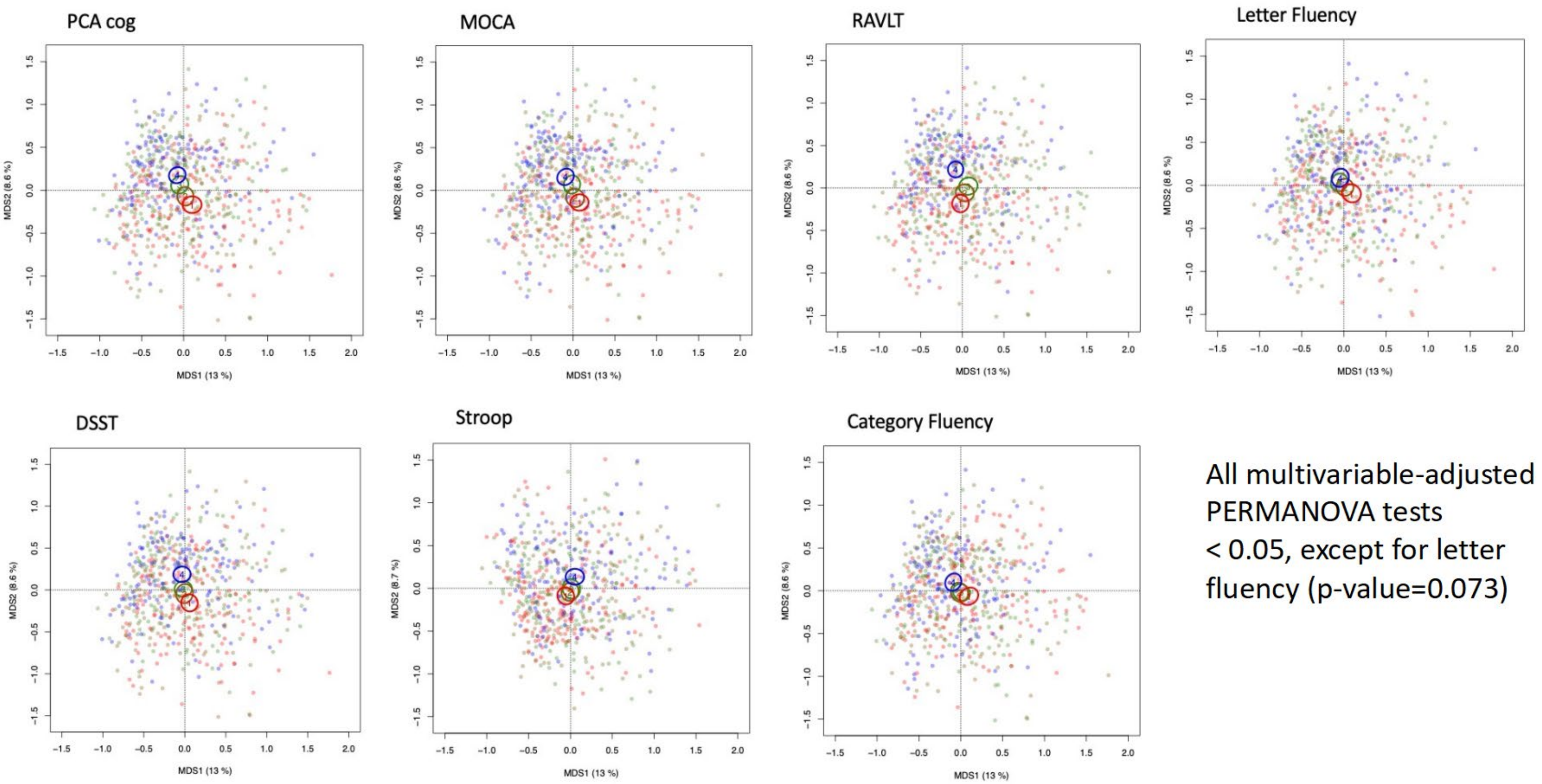

PCoA biplots of associations between cognition measures and microbial dissimilarity: CARDIA microbiome study. Biplots based on Bray Curtis dissimilarity matrix (non-Euclidean) for genera abundance in study participants with respect to quartiles (Q1: red; Q2: brown; Q3: green; and Q4: blue) of the first principal component (PC) of cognitive assessments (factor 1), MoCA, DSST, Stroop, RAVLT, letter fluency, and category fluency. Biplots shown for first two PCoA axes. Centroids illustrate the 95% CI for the mean location of each population subgroup. Permutational multivariate ANOVA (PERMANOVA) *P* values were less than 0.05 for all cognitive measures except letter fluency (p value = 0.073) in multivariable-adjusted analysis including: sequencing batch, age, race, sex, study center, education, smoking, physical activity, diet quality score, medication use, BMI, diabetes, and hypertension. MDS: multidimensional scaling.

© 2022 Meyer K et al. *JAMA Network Open.*
